# Visible light-driven dearomative ring expansion of (aza)arenes to access dihydrofuran-based polycyclic compounds†

**DOI:** 10.1039/d4sc00748d

**Published:** 2024-05-07

**Authors:** Linghong Zhang, Mengdi You, Xu Ban, Xiaowei Zhao, Yanli Yin, Shanshan Cao, Zhiyong Jiang

**Affiliations:** a Key Laboratory of Natural Medicine and Immuno-Engineering of Henan Province, Henan University Kaifeng Henan P. R. China 475004 chmjzy@henu.edu.cn; b School of Chemistry and Chemical Engineering, Pingyuan Laboratory, Henan Normal University Xinxiang Henan P. R. China 453007 caoshanshan@htu.edu.cn

## Abstract

The dearomative expansion of aromatic rings has long been pursued by chemists due to its potential to provide tractable approaches for synthesizing valuable non-aromatic molecules. To circumvent the conventional use of hazardous and unstable diazo compounds, photochemical synthesis has recently emerged as a promising platform. However, protocols that can effectively handle both arenes and azaarenes remain scarce. Herein, we introduce a generic strategy that efficiently converts β-(aza)aryl-β-substituted enones into biologically significant cycloheptatriene derivatives, including their aza-variants. This method allows for the easy modulation of diverse functional groups on the product and demonstrates a wide substrate scope, evidenced by its excellent tolerance to various drug motifs and good compatibility with five-membered azaarenes undergoing ring expansion. Moreover, DFT calculations of plausible mechanisms have motivated the implementation of an important cascade diradical recombination strategy for 1,3-dienones, thus facilitating the synthesis of valuable 2-oxabicyclo[3.1.0]hex-3-ene derivatives.

## Abstract 2

A visible light-mediated dearomative ring expansion strategy that is general as the good compatibility of diverse arenes and azaarenes have been developed.

## Graphical abstract

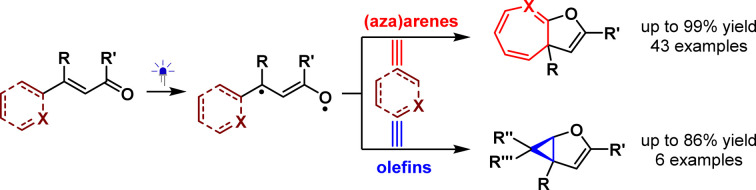

## Introduction

**Scheme 1:**
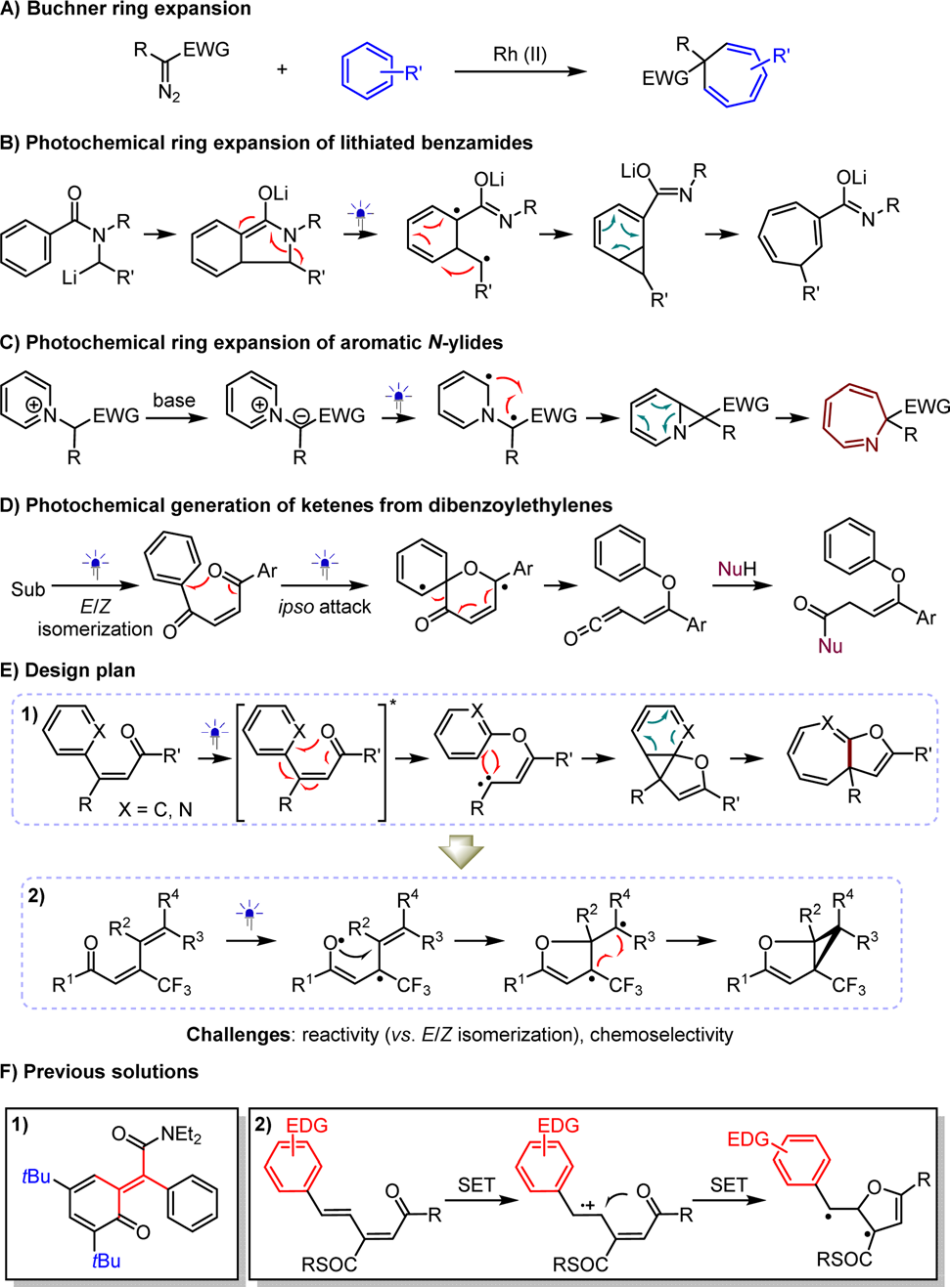
Development of photochemical ring expansion of (aza)arenes.

The direct construction of value-added compounds from simple chemical feedstocks is a perpetual goal in the development of synthetic methodologies.^1^ The Buchner ring expansion,^2^ discovered in 1883, has attracted considerable attention due to its cost-effective approach to synthesizing biologically and synthetically important cycloheptatriene derivatives from petroleum-derived arenes (Scheme 1A).^2–7^ Nonetheless, the use of hazardous and unstable diazo compounds as reaction partners constitutes a frustrating dilemma, stimulating continuous exploration of alternative protocols. To this end, the Clayden group in 2003 devised a photochemical ring expansion for lithiated benzamides (Scheme 1B),^8^ which recently demonstrated its robustness^9^ by successfully preparing enantioenriched products through the pairing of chiral anions with lithium salts. Furthermore, Beeler and coworkers in 2021 described a photochemical rearrangement of aromatic *N*-ylides to furnish azepines (Scheme 1C).^10^ Central to the success of these strategies is the generation of diradical species from substrates through absorbing photon energy, which leads to the formation of crucial cyclopropane intermediates, effectively avoiding the use of diazo substrates as carbene precursors. These elegant works reveal that leveraging excited states as a sustainable platform is highly promising for exploring complementary strategies, due to its ability to transfuse sufficient chemical energy from photons and the high reactivity of the radicals, leading to mild reaction conditions and good functional group tolerance.

**Scheme 2:**
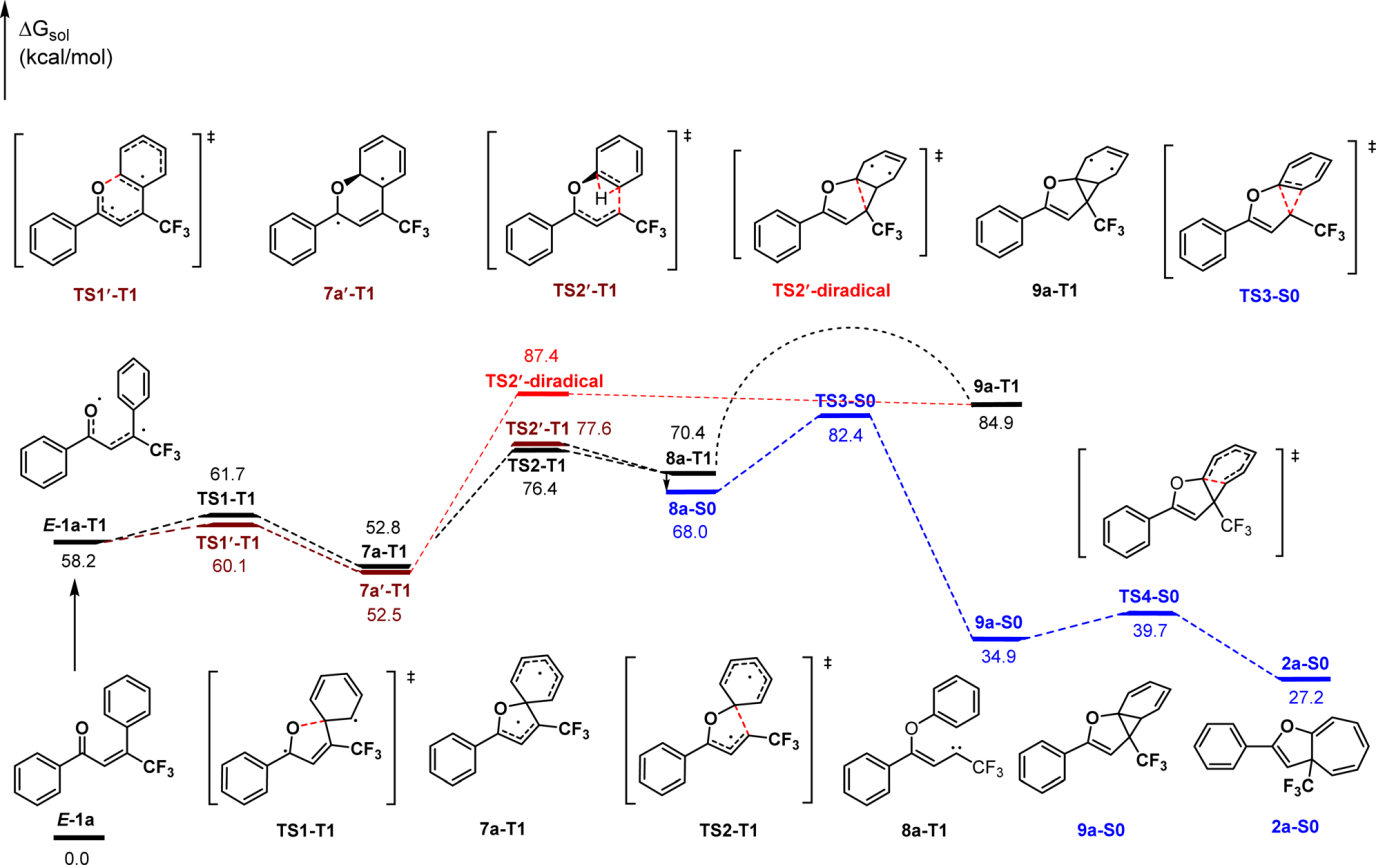
Plausible reaction pathway.

In recent years, our group has devoted considerable effort to visible light-driven photochemical synthesis,^11–16^ where β-substituted enones were used in the catalytic asymmetric reductive azaarylation.^15^ In addition to experiencing single-electron reduction, these olefins are prone to *E*/*Z* isomerization and homocoupling, triggered by the formation of triplet species *via* energy transfer or direct excitation. Notably, such an event of producing formal diradicals has been extensively used since 1962 in accomplishing attractive reactions of dibenzoylethylenes with diverse nucleophiles (Scheme 1D).^17–21^ The mechanism profile entails the production of key ketene intermediates triggered by intramolecular *ipso*-addition of oxygen radicals to arenes, resulting in six-membered ring diradical species, which then undergo a final rearrangement process. It is tempting to assume that if photoexcited β-aryl-β-substituted enones can undergo *O*-radical addition and recombination of diradicals, cyclopropane intermediates might be produced *via* intramolecular [1 + 2] cycloaddition of the resulting carbenes with arenes. This could offer a useful ring expansion approach to cycloheptatriene variants bearing the valuable dihydrofuran moiety^22–24^ (Scheme 1E-1). If it is feasible, we wondered whether such intramolecular radical addition to olefins, instead of aza(arenes), could initiate a five-membered ring transition state, thereby facilitating the synthesis of the medically valuable dihydrofuran-based cyclopropanes^25,26^ from 1,3-dienones *via* a predicable cascade diradical recombination pathway, with diradicals favored over carbenes as intermediates (Scheme 1E-2). The challenge of reactivity in both scenarios stems from the release of energy by the *E*/*Z* isomerization of the triplet enones,^27^ a long-standing dilemma in developing practical synthetic methods. To prevent isomerization, the Bos group devised *ortho*-quinone methides stabilized by bulky substituents (Scheme 1F-1).^28^ Although this synthetic issue was subsequently solved by Adam and co-workers using dimethyldioxirane oxidation of benzofurans, the requirement for specific *tetra*-substituted olefins remains unavoidable.^29^ In addition, we hypothesized that the exothermal aromatization process might be another crucial point for the success of these chemical transformations. It is worth mentioning that cycloheptatrienes are prone to undergo 4π electrocyclization after absorption of photon energy,^30^ which further compromises the yield and represents a challenge for chemoselectivity. On the other hand, to avoid the isomerization of dienes, which the Das group demonstrated considerably deteriorates yields,^31^ a redox method was developed. This method relies on the use of electron-rich aryl groups to enable the oxidation of olefins *via* single electron transfer (SET), indispensable for facilitating the reaction (Scheme 1F-2). Unfortunately, this dependency limits the method's wider applicability.^32^

Herein, we report the successful implementation of visible light-driven dearomative ring expansion on both arenes and azaarenes, using readily accessible β-(aza)aryl-β-substituted enones as feedstocks. A variety of desired cycloheptatrienes and their aza-variants were obtained in high yields. The wide range of substrates is validated not only by the flexible modulation of diverse functional groups on the substrates but also by the good efficiency of various six- and even five-membered azaarenes in experiencing ring expansion. To our delight, such a direct photoexcitation method is also feasible for 1,3-dienones, providing a general approach to constructing 2-oxabicyclo[3.1.0]hex-3-ene derivatives.

## Results and discussion

Given the importance of trifluoromethyl (CF_3_) in drug discovery, our study commenced with (*E*)-4,4,4-trifluoro-1,3-diphenylbut-2-en-1-one (1a) as the model substrate (Table 1A). The transformation was initially tested in toluene at 25 °C and irradiated with a 3 W blue LED (*λ* = 447 nm). To our delight, while the (*Z*)-isomer of 1a (*i.e.* (*Z*)-1a) was achieved in 40% yield as a major byproduct, the targeted 2a could be obtained in 32% yield (entry 1, Table 1A). After screening solvents (entries 2–5, Table 1A), THF was found to prominently improve the reactivity and chemoselectivity, leading to 2a in 85% yield. The influence of the light source's wavelength on the concentration of triplet species, which is crucial for the transformation's success, was subsequently examined (Table 1B). A 3 W LED with *λ* = 419 nm was found to increase the yield of 2a to 93%, with roughly no detection of (*Z*)-1a (entry 3, Table 1B). The results summarized in entries 4–5 further elucidated that an appropriate emission wavelength of LED is critical for the efficient occurrence of the reaction. Finally, we observed that slightly raising the temperature up to 30 °C could render 2a in 95% yield as the best result (entry 2, Table 1C). The control experiment performed in the dark confirmed the indispensability of the light source in the transformation (entry 1, Table 1D). It is worth mentioning that the ambient atmosphere is able to generate product 2a with a slightly reduced yield (85%) due to the slower reaction rate.

**Table 1:** Optimization of the reaction conditions and control experiments^a^

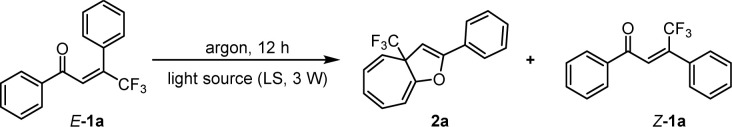							
(A) *T* = 25 °C, LED: *λ*^max^_em_ = 447 nm				(B) *T* = 25 °C, THF			
Entry	Solvent	Yield of 2a	Yield of *Z*-1a	Entry	LS (*λ*^max^_em_)	Yield of 2a	Yield of *Z*-1a
1	Toluene	32%	40%	1	370 nm	N.R.	N.R.
2	CH_2_Cl_2_	23%	52%	2	393 nm	43%	23%
3	CHCl_3_	53%	21%	3	419 nm	93%	0%
4	THF	85%	5%	4	493 nm	N.R.	N.R.
5	Et_2_O	56%	27%	5	520 nm	N.R.	N.R.
(C) THF, LED: *λ*^max^_em_ = 419 nm				(D) *T* = 30 °C, THF, *λ*^max^_em_ = 419 nm			
Entry	*T* (°C)	Yield of 2a	Yield of *Z*-1a	Entry	Conditions	Yield of 2a	Yield of *Z*-1a
1	35	93%	0%	1	No light	N.R.	N.R.
2	30	95%	0%	2	Under air	85%	0%
3	10	90%	0%	THF = tetrahydrofuran		N.R. = no reaction	
4	−10	85%	5%	LED = light-emitting diode			

a The reaction was performed on a 0.1 mmol scale. The yield was isolated by flash column chromatography on silica gel.

With the optimal reaction conditions in hand, the substrate scope for the dearomative ring expansion of arenes was investigated (Table 2). A variety of β-CF_3_–β-phenyl-substituted enones featuring diverse electron-withdrawing or electron-donating groups on the aromatic rings as the carbonyl substituents were first examined, resulting in the corresponding products 2b–h in 76 to 99% yields. Among them, the good tolerance of OBoc (2f) and OH (2g) encouraged us to assemble pharmaceutically important molecules or even drugs onto such a valuable cycloheptatriene derivative. As a result, products 2i–m containing different esters derived from deoxycholic acid (2i), dehydrocholic acid (2j), abietic acid (2k), gemfibrozil (2l) and oleic acid (2m) were obtained in good to excellent yields. The robustness of the method is further underscored by the convenient introduction of other useful functional groups, such as fused aromatic rings (2n), electron-rich heteroaromatic rings (2o–p), 2*H*-chromen-2-one (2q) and a series of electron-deficient azaarenes (2r–v). Next, the viability of alkyls as the ketone substituents was explored, and positive results were supported by the production of 2w–y in 73 to 79% yields, indicating the capability of the method to tolerate both acyclic and cyclic alkyls on the molecule using readily accessible enones. It was found that this method enables not only other simple arenes (2za) but also fused arenes (*e.g.*, naphthalene and anthracene, 2zb–zc) to undergo efficient ring expansion. The unique 2za obtained in the reaction indicates the high viability of the *ipso*-substitution pathway to render the carbene intermediate (*vide infra*). When the 3-arene was not symmetric, featuring substituents at the 3- and/or 2-positions, the ring expansion was less selective (*e.g.*, 2zd-1/2zd-2 = 1 : 3). We then replaced CF_3_ of 1 with other meaningful functional groups, including different polyfluoroalkyls, esters, nitriles, and notably, the electron-neutral methyl, furnishing the corresponding products 2ze–zj in 65 to 98% yields, where the transformation into 2zj was considerably slower and had to be prolonged to 72 h. Amazingly, two enone moieties in a molecule were found to be capable of efficiently experiencing ring expansion individually, furnishing complex molecules (*i.e.*, 2zk) containing two cycloheptatrienes. Finally, the study revealed that dearomative ring expansion of enones featuring cyclic ketones can pave the way to cycloheptatriene-containing polycyclic compounds (2zl–zn). Notably, although the photo-activated enones could potentially undergo [2 + 2] cycloaddition,^33^ no corresponding side products were observed in these reactions, likely due to the high steric hindrance.

**Table 2:** Substrate scope with respect to ring expansion of arenes^a^

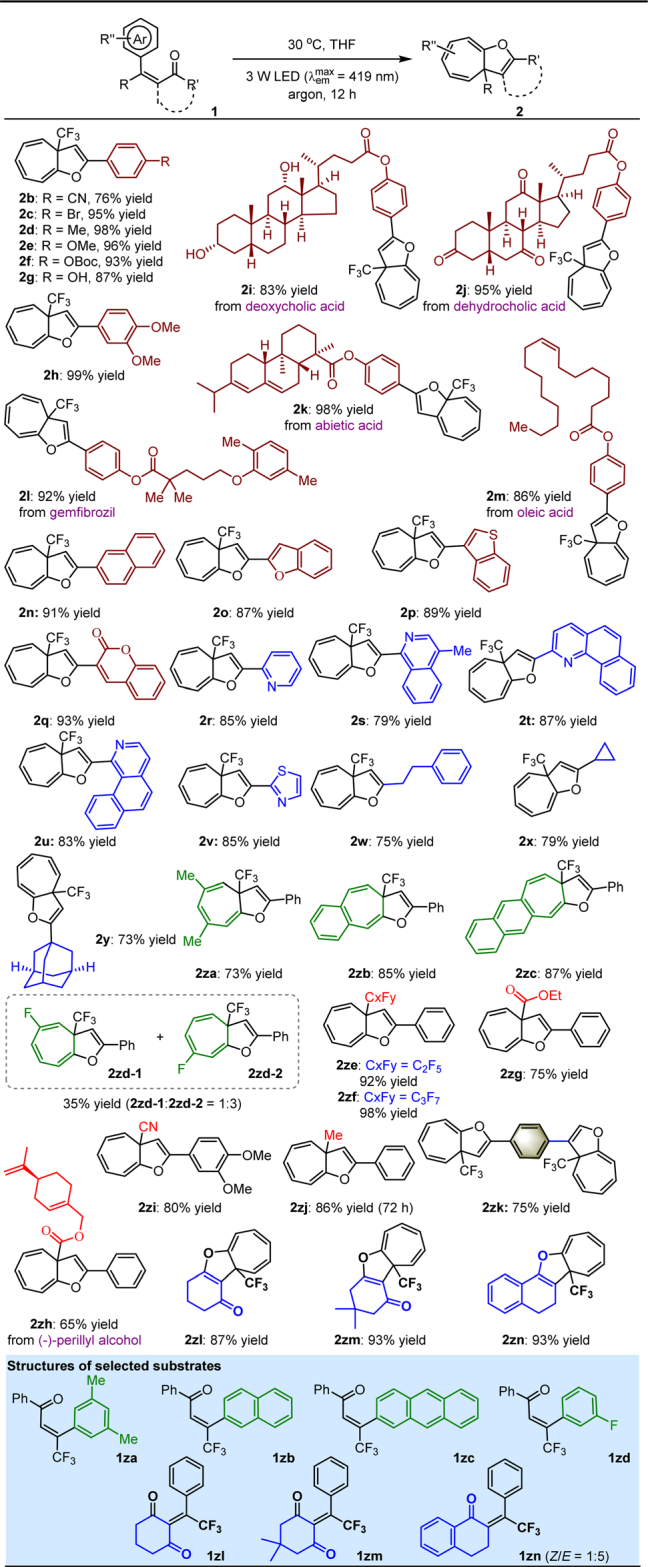

a Reaction was performed on a 0.1 mmol scale. Yield was isolated by flash column chromatography on silica gel.

The success motivated us to further test the feasibility of this method for the attractive ring expansion of azaarenes. As depicted in Table 3, when diverse imine-containing azaaryls, such as 2-pyridyls (3a–c), 2-benzothiazolyl (3d), and 2-benzoxazolyl (3e), were assembled on the β-position of enones instead of aryls, the transformations could still undergo the established approach, affording a series of valuable aza-cycloheptatriene, 1,4-thiazine and 1,4-oxazine variants 4a–e in 43 to 76% yields.

**Table 3:** Substrate scope with respect to ring expansion of azaarenes^a^

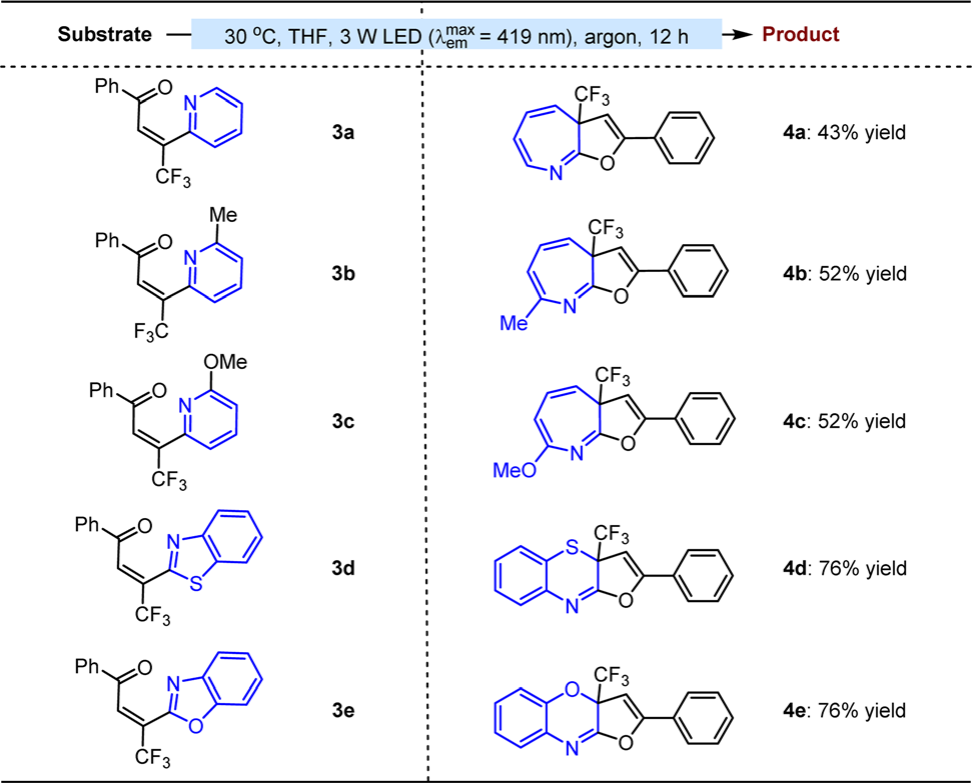

a Reaction was performed on a 0.1 mmol scale. Yield was isolated by flash column chromatography on silica gel.

In view of the product structure, the formation of the cyclopropane intermediates should be reasonable. To further confirm this deduction and importantly, to gain insights on the possible pathway for generating the carbene intermediates prior to the cyclopropylation (*i.e.*, *ipso*-addition or *ortho*-addition of *O*-radical), we performed density functional theory (DFT) calculations at the M06-2X level of theory on the reaction of 1a. As shown in Scheme 2, visible light first initiates the excitation of *E*-1a to its triplet state E-1a-T1. Spin density analysis indicates that the two unpaired electrons mainly locate on the oxygen atom and β-carbon atom of the carbonyl group respectively.^34^ From *E*-1a-T1, the *ipso*-addition of *O*-radical (TS1-T1, 61.7 kcal mol^−1^) followed by C–C bond cleavage (TS2-T1, 76.4 kcal mol^−1^) leads to generating the triplet state carbene intermediate 8a-T1. This process is reversible and endothermic by 12.2 kcal mol^−1^. By comparison, the *ortho*-addition pathway was also considered, which proceeds through transition states TS1′-T1 and TS2′-T1 with energy values of 60.1 kcal mol^−1^ and 77.6 kcal mol^−1^ respectively. The calculation results indicate that the *ipso*-addition pathway for the generation of carbene intermediate 8a-T1 is slightly more favorable than the *ortho*-addition case due to a 1.2 kcal mol^−1^ lower energy of TS2-T1 than TS2′-T1. In addition, the diradical cycloaddition *via* the transition state TS2′-diradical from the diradical intermediate 7a′-T1 was also considered, which is 11.0 kcal mol^−1^ higher in energy than the transition state TS2-T1. Consequently, the diradical mechanism is disfavored thermodynamically and kinetically, and can be excluded.

Then, the formed triplet state carbene intermediate 8a-T1 must transition to the S0 state *via* a T1 → S0 intersystem crossing to finish the dearomative ring expansion process. The first cyclopropanation of the aromatic ring occurs *via* the concerted transition state TS3-S0 (82.4 kcal mol^−1^) leading to the formation of the norcaradiene intermediate 9a-S0, which is the rate-determining step and exothermic by 33.1 kcal mol^−1^. Subsequently, 9a-S0 rearranges by 6π electrocyclization through the transition state TS4-S0 (39.7 kcal mol^−1^) to form the final cycloheptatriene product 2a-S0. In contrast, the dearomative ring expansion process cannot be finished adiabatically in the T1 state because it is thermodynamically unfavorable compared to the S0 state.

**Scheme 3:**
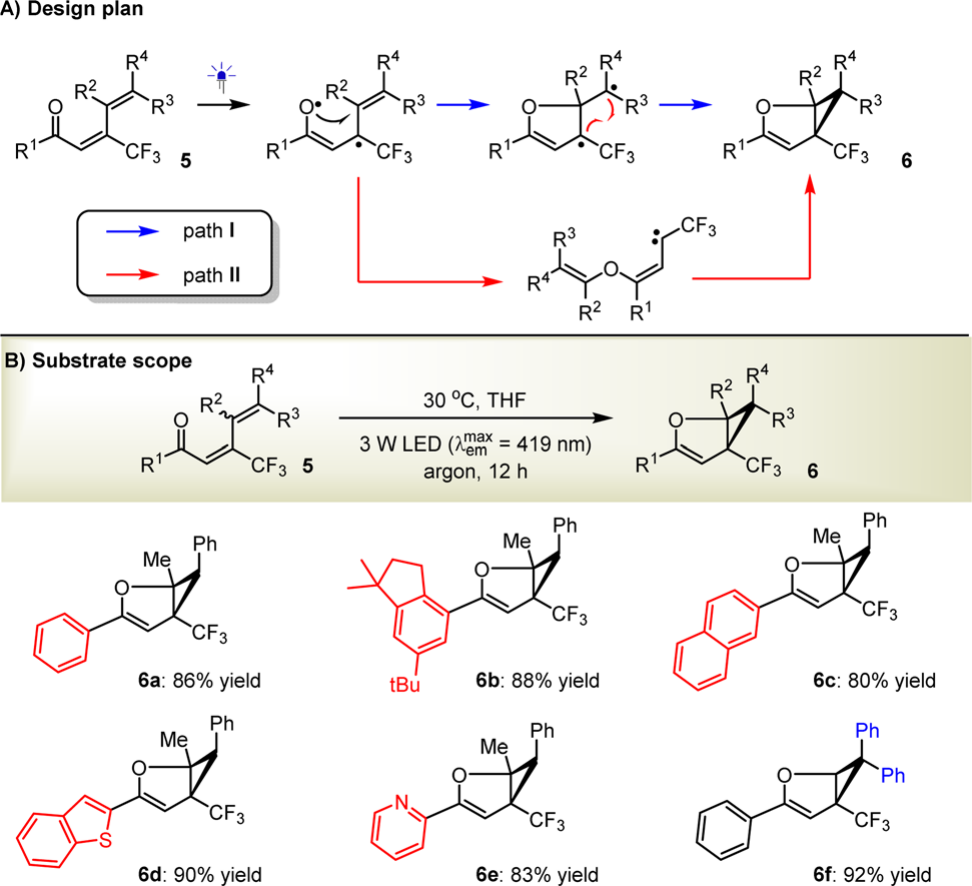
From olefins to cyclopropanes.

In light of the successful application of the enones and the elucidation of plausible mechanisms, which reveal that the compatibility of the direct photoexcitation platform can be addressed by carefully modulating the light source, solvent, temperature, *etc.*, we were inspired to challenge the synthesis of dihydrofuran-based cyclopropanes (6) from 1,3-dienones (5) (Scheme 3A). We considered this to be viable since the photoexcited 5, as a formal diradical, will be able to readily undergo sequential radical addition and intramolecular radical coupling (path I, Scheme 3A). In addition, the formation of carbene intermediates, similar to the transformations of those enones, would also be an alternative way to access 6 (path II, Scheme 3A). Accordingly, (2*E*,4*E*)-1,5-diphenyl-3-(trifluoromethyl)hexa-2,4-dien-1-one (5a) was selected as the model substrate to be examined under the standard reaction conditions. We were pleased to find that the desired product 6a was obtained in 86% yield (Scheme 3B). Satisfactory results for products 6b–e can convincingly support the capability of this strategy to readily modulate different functional groups on the dihydrofuran rings, such as functionalized aryls (6b), fused aromatic (6c) and heteroaromatic rings (6d), and azaarenes (6e). The broad substrate scope is further validated by the convenient assembly of diaryls (6f) on the cyclopropane moiety. DFT calculations were also performed, and diradicals but not carbenes were demonstrated to represent the favored intermediates (Scheme S1 in the ESI†).

**Scheme 4:**
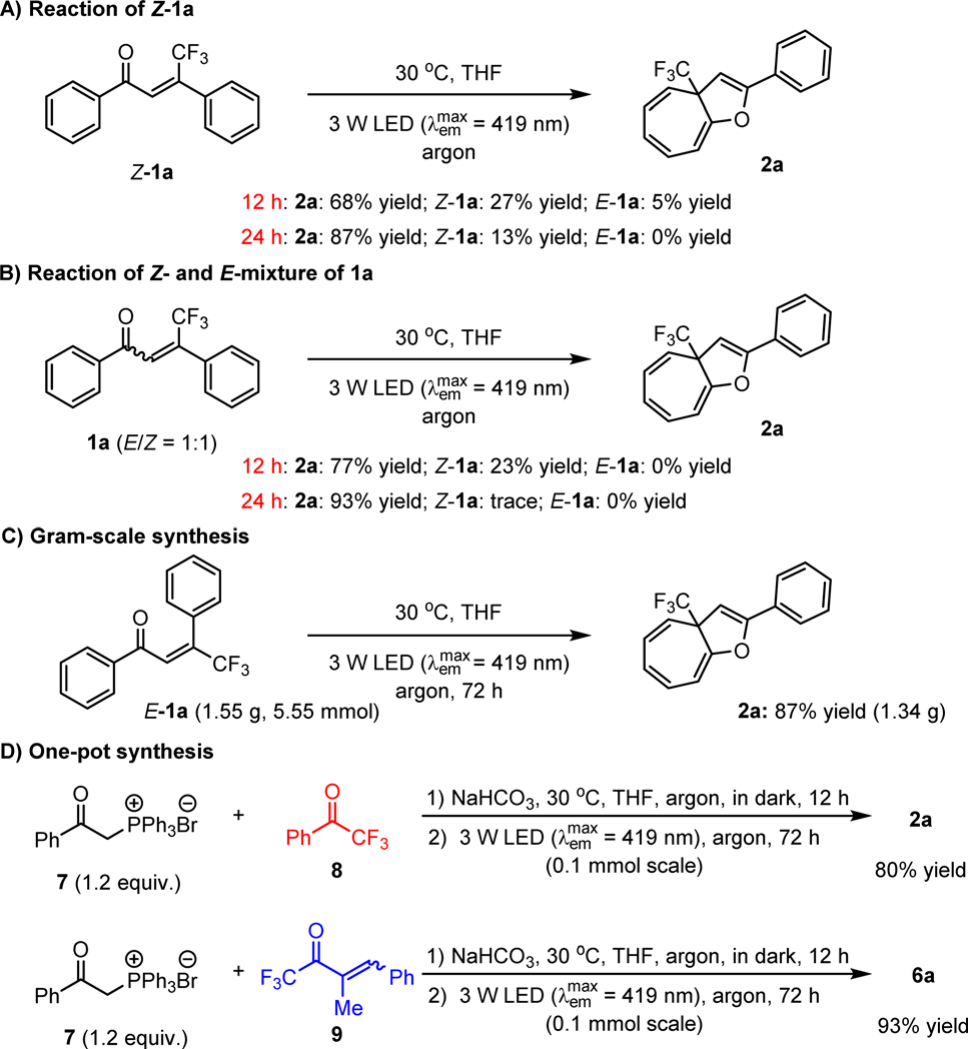
Synthetic potential.

It is worth mentioning that enones 1 can be conveniently prepared, and importantly, only *E*-configurations are obtained, revealing the excellent diastereoselectivity of the employed method. To further explore the utility of this dearomative ring expansion approach, *Z*-1a was attempted as a representative of *Z*-olefins under established reaction conditions, given that photo-activated *Z*-olefins should be able to conveniently form *E*-olefins. As a result, the transformation became slightly sluggish in view of only 68% yield of product 2a obtained (Scheme 4A). After 24 h, the yield of 2a could be enhanced to 87%. Given that the potential enones should concurrently possess *E*- and *Z*-isomers which are difficult to isolate efficiently, the transformation of 1a with a 1 : 1 *E*/*Z* ratio was performed, leading to 2a in 77% yield after 12 h (Scheme 4B). After 24 h, the yield of 2a can increase to 93%. These results clearly reveal that *E*-olefins can react more rapidly than *Z*-olefins. In addition to high yields with a wide range of substrates, large-scale preparation is of considerable importance for the application of the method in industry. As such, we then attempted the reaction of accessing 2a on a 5.55 mmol scale (Scheme 4C). It was found that after prolonging the reaction time to 72 h, the desired product could be obtained in 87% yield. Furthermore, given that these olefin feedstocks are readily synthesized *via* the Wittig reaction,^31^ we wondered whether these compounds could be directly used as starting substrates to undergo a one-pot sequential reaction involving the Wittig reaction followed by photoactivated ring expansion. Gratifyingly, the investigations revealed the viability of such a more convenient synthetic mode. As shown in Scheme 4D, the transformations of ylide 7 with ketone 8 or 9 were first carried out in the presence of NaHCO_3_ in THF at 30 °C for 12 h in the dark. Subsequently, the reaction mixtures were directly irradiated with a 3 W blue LED for another 72 h, leading to products 2a and 6a in high yields. The longer reaction time is due to the reduced efficiency of the photon absorption, stemming from the formation of insoluble side products during the initial step.

## Conclusions

In summary, we have successfully developed a general dearomative ring expansion strategy for both arenes and azaarenes using a sustainable visible light-driven platform. Key to the success of this method is the convenient formation of carbene intermediates through triplet enones undergoing diradical recombination *via* five-membered ring transition states. The high yields, broad substrate scope involving the good tolerance of diverse complex drugs and bioactive molecule motifs, and the valuable cycloheptatriene products strongly disclose the synthetic ubiquity and importance of this method. Additionally, it has prompted the development of a useful cascade diradical recombination approach for 1,3-dienones, providing a significant tool for the synthesis of 2-oxabicyclo[3.1.0]hex-3-ene derivatives. We anticipate that this work will inspire the pursuit of novel diradical recombination protocols for a variety of non-saturated functional groups, creating versatile and highly atom-economical platforms for the efficient preparation of complex molecules through subsequent intramolecular or intermolecular chemical transformations.

## Data availability

General information, optimization of reaction conditions, general procedures, mechanistic studies, DFT calculations, synthetic applications, characterization data, X-ray of products, and NMR spectra.

## Author contributions

Z. J. conceived and designed the experiments. L. Z., M. Y., X. Z., and Y. Y. performed the experiments and prepared the ESI.† L. Z. and X. B. helped with isolating the new compounds and analyzing the data. S. C. performed DFT calculations; Z. J., X. B. and S. C. wrote the paper. L. Z. and M. Y. contributed equally to this work. All authors discussed the results and commented on the manuscript.

## Conflicts of interest

There are no conflicts to declare.

## Supplementary Material

SC-015-D4SC00748D-s001

SC-015-D4SC00748D-s002
