# Supplementary material for: Visible light-driven dearomative ring expansion of (aza)arenes to access dihydrofuran-based polycyclic compounds
Source: Chem Sci. 2024 May 7;15(23):8828–34. doi: 10.1039/d4sc00748d (PMC11168080; doi:10.1039/d4sc00748d)
Supplement: SC-015-D4SC00748D-s001 [file SC-015-D4SC00748D-s001.pdf]

Electronic Supplementary Material (ESI) for Chemical Science.
This journal is © The Royal Society of Chemistry 2024

Supporting Information

Visible Light-Driven Dearomative Ring Expansion of (Aza)arenes to Access Dihydrofuran-based Polycyclic Compounds

Linghong Zhang,[1] Mengdi You,[2] Xu Ban,[2] Xiaowei Zhao,[1] Yanli Yin,[2] Shanshan Cao,[2,*] and Zhiyong Jiang[1,2,*]

[1]*Key Laboratory of Natural Medicine and Immuno-Engineering of Henan Province, Henan University, Kaifeng, Henan, P. R. China 475004*

[2]*School of Chemistry and Chemical Engineering, Henan Normal University; Pingyuan Laboratory, Xinxiang, Henan, P. R. China 453007*

**E-mail: caoshanshan@htu.edu.cn; jiangzhiyong@htu.edu.cn*

Table of Contents

1. General information

S3-S4

2. Synthetic procedures for starting materials

S5-S8

3. General experimental procedures

S9-S10

4. Experimental equipment

S11-S13

5. Mechanism studies

S14-S16

6. DFT calculation

S17-S48

7. The structural determination of **2za** and **2zd**

S49-S53

8. X-Ray crystallographic data of **2c**, **2za** and **6b**

S54-S76

9. Characterization of some starting substrates

S77-S88

10. Characterization of product

S89-S103

11. Copies of NMR spectra

S104-S237

12. References

S238

1. General information

(1) General procedures and methods

Experiments involving moisture and/or air sensitive components were performed under a positive pressure of argon in oven-dried glassware equipped with a rubber septum inlet. Dried solvents and liquid reagents were transferred by oven-dried syringes or hypodermic syringe cooled to ambient temperature in a desiccator. Moisture in non-volatile reagents/compounds was removed in high *vacuo* by means of an oil pump and subsequent purging with nitrogen. Solvents were removed *in vacuo* under ~30 mmHg and heated with a water bath at 30–35 ºC using rotary evaporator with aspirator. The condenser was cooled with running water at 0 ºC.

All experiments were monitored by analytical thin layer chromatography (TLC). TLC was performed on pre-coated plates, 60 F_{254}. After elution, plate was visualized under UV illumination at 254 nm for UV active material. Further visualization was achieved by staining $Ce(SO_4)_2$ and phosphomolybdic acid solution. For those using the aqueous stains, the TLC plates were heated on a hot plate.

Columns for flash chromatography (FC) contained *silica gel* 200–300 mesh. Columns were packed as slurry of *silica gel* in petroleum ether and equilibrated solution using the appropriate solvent system. The elution was assisted by applying pressure of about 2 atm with an air pump.

(2) Instrumentations

Proton nuclear magnetic resonance (^{1}H NMR), carbon NMR (^{13}C NMR), and fluorine NMR (^{19}F NMR) were recorded in $CDCl_3$ otherwise stated. Chemical shifts are reported in parts per million (ppm), using the residual solvent signal as an internal standard: $CDCl_3$ (^{1}H NMR: δ 7.26, singlet; ^{13}C NMR: δ 77.0, triplet). Multiplicities were given as: *s* (singlet), *d* (doublet), *t* (triplet), *q* (quartet), *quintet*, *m* (multiplets), *dd* (doublet of doublets), *dt* (doublet of triplets), and *br* (broad). Coupling constants (*J*) were recorded in Hertz (Hz). The number of proton atoms (*n*) for a given resonance was indicated by *n*H. The number of carbon atoms (*n*) for a given resonance was indicated by *n*C. HRMS (Analyzer: TOF) was reported in units of mass of charge ratio (m/z). Mass samples were dissolved in CH_3CN (HPLC Grade) unless otherwise stated. Melting points were determined on a melting point apparatus.

(3) Materials

Starting materials were purchased commercially from J&K, Adamas, Energy Chemical, Sigma-

Aldrich, Alfa Aesar, Acros Organics, and other commercial suppliers with reagent grade quality. They were used without further purification unless specified. All solvents used, mainly petroleum ether (PE) and ethyl acetate (EtOAc) were distilled. Anhydrous dichloromethane (DCM) was freshly distilled from CaH_2 and stored under N_2 atmosphere. THF, Et_2O and toluene were freshly distilled from sodium/benzophenone before use. All synthesized compounds were stored in a -20 °C freezer.

2. Synthetic procedures for starting materials.

(1) Synthesis of 1a-zf, 1zk, 3a-e and 5a-f[10]

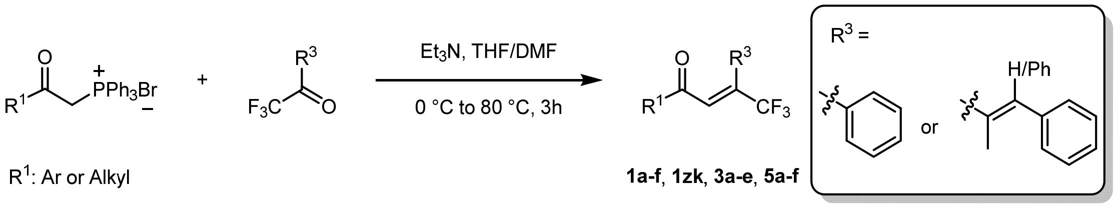

1a-f, **1zk**, **3a-e**, **5a-f**

To a cooled solution (0 °C) of (benzoylmethyl)triphenylphosphonium bromide (7.5 mmol) and Et_3N (7.5 mmol) in THF (20.0 mL) was added trifluoromethyl ketone (7.5 mmol). The mixture was allowed to warm to room temperature and stirred for 15 min. Then it was heat to 80 °C for 3 h with the reaction progress monitored by TLC. After the reaction was completed, it was quenched with saturated NH_4Cl (aq.), extracted with EA. The organic phase was dried with Na_2SO_4 and concentrated under reduced pressure. The residue was purified by column chromatography to offer the corresponding *E*-*β*-CF_3-chalcones. See previously reported methods for the synthesis of trifluoromethyl aryl ketones.[11]

(2) Synthesis of 1zg, 1zh, 1zi[12]

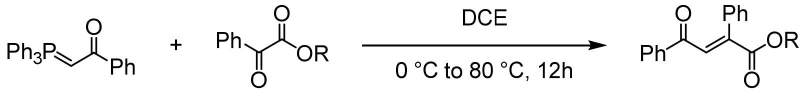

1zg, 1zh

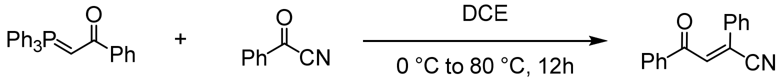

1zi

To a 20 mL vial in air containing a magnetic stir bar, ethyl benzoylformate (5.00 mmol, 1.00 equiv), (phenacylidene)triphenylphosphorane (6.00 mmol, 1.20 equiv), and 10 mL DCE were added and heated at 80 °C for 24 h. The vial was cooled to room temperature, and the contents of the vial were filtered through a plug of silica. The solid that remained on the silica was washed with ether. The filtrate was concentrated under vacuum and the crude product was purified by automated silica gel chromatography, eluting with a mixture of ethyl acetate and hexanes (gradient 0:100 → 20:80 EtOAc:Hexanes).

(3) Synthesis of starting materials 1zj[13]

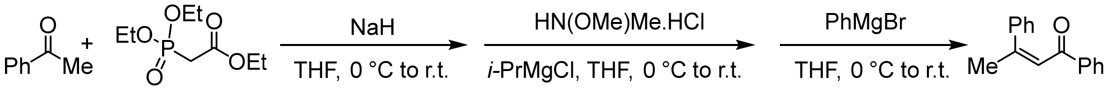

***Z*-1zj**

Triethyl-2-phosphonopropionate (1.2 equiv.) was slowly added towards a stirring suspension of NaH (60% in mineral oil, 1.2 equiv.) in THF at 0 °C. After 30 min, acetophenone (1.0 equiv.) was added dropwise and the mixture was allowed to stir over-night at room temperature. Thereafter, the mixture was quenched with NH_4Cl and extracted with Et_2O (3x). The combined organic phases were washed with brine, dried over Na_2SO_4 and evaporated in vacuo to dryness to give the crude. The crude was purified by column chromatography (pentane/Et_2O, 20:1) to obtain the product as an *E*/*Z* mixture, which was used in the next step without removal of the undesired configurational isomer. i-PrMgCl (3.0M in THF, 4.0 equiv.) was slowly added towards a stirring solution of α, β-unsaturated ester (1.0 equiv.) and HN(OMe)Me.HCl (2.0 equiv.) in THF at 0 °C over a time period of 30 min. The reaction mixture was allowed to stir at room temperature and was quenched after 1 h with NH_4Cl followed by extraction with Et_2O (3x). The combined organic phases were washed with brine, dried over Na_2SO_4 and evaporated in vacuo to dryness to give the crude. The crude was purified by column chromatography (pentane/Et_2O, 3:1) wherein the undesired configurational isomer was separated from the mixture. Suitable Grignard reagent (PhMgBr) (1.1 equiv.) was added dropwise to a stirring solution of Weinreb amide (1.0 equiv.) in THF at 0 °C. The reaction mixture was allowed to stir at room temperature and was quenched after 1 h with NH_4Cl followed by extraction with Et_2O (3x). The combined organic phases were washed with brine, dried over Na_2SO_4 and evaporated in vacuo to dryness to give the crude. The Z/E ratio of the product was determined to be 1:1 through crude NMR analysis. Through TLC and NMR analysis, the Rf value for the *Z*-*α*,*β*-unsaturated ketone. was found to be 0.6, while the Rf value for the *E*-*α*,*β*-unsaturated ketone. was 0.5. The crude was purified by column chromatography (pentane/Et_2O, 100:0→50:1→25:1) to yield the desired *Z*-*α*,*β*-unsaturated ketone.

(4) Synthesis of 1zi and 1zm[14]

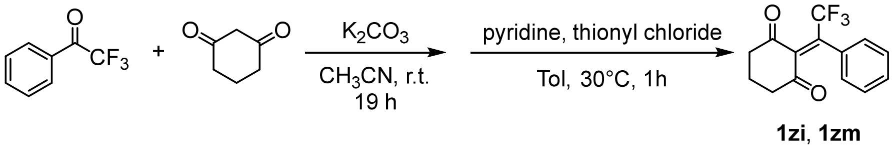

1zi, **1zm**

To a solution of 2,2,2-trifluoro-1-phenyl-ethanone (5 mmol, 1.0 equiv.) in acetonitrile (50.0 mL) was added cyclohexanedione derivative (5 mmol, 1.0 equiv.) and potassium carbonate (1.0 equiv.) at room temperature. The reaction was stirred for 19 h at room temperature, then reaction mass was filtered and concentrated under reduced pressure to get crude adduct. This crude adduct (1.0 equiv.) was suspended in toluene (80.0 mL) and pyridine (3.0 equiv.). To this reaction mixture thionyl chloride (1.3 equiv.) was added dropwise maintaining temperature below 30 °C. The reaction was stirred 1 h at room temperature. After completion of the reaction the reaction was quenched with cold water and layers were separated. The combined organic phases were washed with brine, dried over Na_2SO_4 and evaporated in vacuo to dryness to give the crude. The crude was purified by column chromatography (pentane/Et_2O, 95:5) to yield the desired α,β-unsaturated ketone.

(5) Synthesis of 1n.[13]

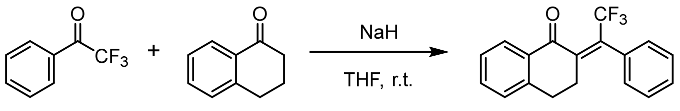

1n

3,4-Dihydronaphthalen-1(2*H*)-one (1.2 equiv.) and 2,2,2-trifluoro-1-phenylethan-1-one (1.0 equiv.) were stirred in THF at room temperature and a solution of NaH (1.2 equiv.) in THF was added after which the mixture was stirred overnight. Thereafter, the mixture was carefully quenched with HCl (4M) until neutral pH was reached. The mixture was concentrated and dissolved in DCM. The organic phase was washed with $NaHCO_3$, washed with brine, dried over Na_2SO_4, and evaporated in vacuo to dryness to give the crude. The crude was purified by column chromatography (pentane/Et_2O, 95:5) to yield **1n**.

(6) Synthesis of 1i–m

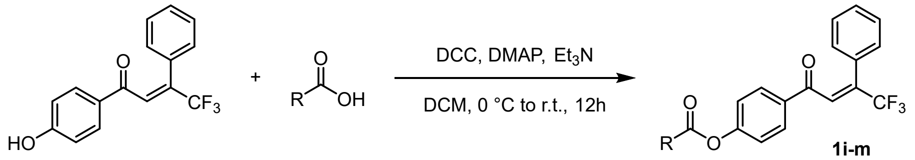

1i-m

To a cooled solution (0 °C) of the ketones (1.0 mmol) in DCM (5 mL) was added acid (1.0 mmol), DMAP (0.10 mmol) and DCC (1.0 mmol). The mixture was degassed and back-filled with argon (3x). Then it was stirred at 0 °C for 1 h and allowed to warm to room temperature and stirred overnight with the reaction progress monitored by TLC. After the reaction was completed, the solution was washed with water, and brine, dried over Na_2SO_4 and evaporated.

The product corresponding product was purified by flash column chromatography.

The method to access **1c**, **1d**, and **1e** has been reported.[15] The method to access **5** has been reported.[16]

(7) Synthesis of *Z*-1a

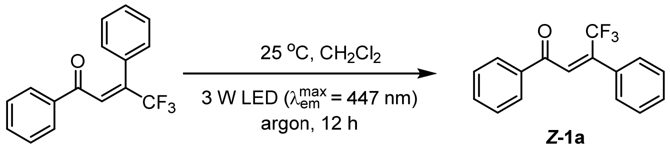

E-**1a** (0.10 mmol, 1.0 equiv.) and DCM (2.0 mL) were sequentially added into the Schlenk tube, and the reaction was degassed three times by freeze-pump-thaw method. The reaction mixture was stirred under an argon atmosphere at 25 °C, then irradiated by a 3 W 447 nm LED at a distance of about 0 cm for another 12 h. The solvent was removed under reduced pressure, and the residue was purified by flash chromatography on *silica gel* (PE/EA = 100/1–50/1 ratio) to give the desired products *Z*-**1a**.

3. General experimental procedures

(1) General procedure for the preparation of cycloheptatrienes 2 and 4

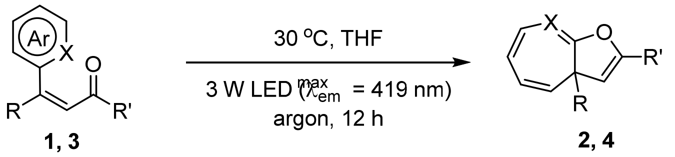

E-**1** (0.10 mmol, 1.0 equiv.) or *E*-**3** (0.1 mmol, 1.0 equiv.) and THF (2.0 mL) were sequentially added into the Schlenk tube, and the reaction was degassed three times by freeze-pump-thaw method. The reaction mixture was stirred under an argon atmosphere at 30 °C, then irradiated by a 3 W 419 nm LED at a distance of about 2 cm for another 12 h. The solvent was removed under reduced pressure, and the residue was purified by flash chromatography on *silica gel* (PE/EA = 100/1–50/1 ratio) to give the desired products **2** or **4**.

(2) General procedure for 2-oxabicyclo [3.1.0] hex-3-ene 6a-6f

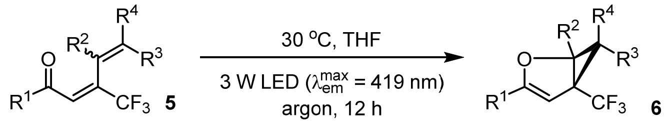

5 (0.1 mmol, 1.0 equiv.) and THF (2.0 mL) were sequentially added into the Schlenk tube, and the reaction was degassed three times by freeze-pump-thaw method. The reaction mixture was stirred under an argon atmosphere at 30 °C, then irradiated by a 3 W 419 nm LED at a distance of about 2 cm for another 12 h. The solvent was removed under reduced pressure, and the residue was purified by flash chromatography on *silica gel* (PE) to give the desired products **6a-6f**.

(3) Gram-scale synthesis

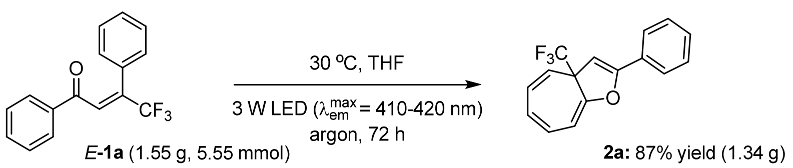

E-**1a** (5.55 mmol, 1.0 equiv.) and THF (12.0 mL) were sequentially added into the 25 mL Schlenk tube, and the reaction was degassed three times by freeze-pump-thaw method. The reaction mixture was stirred under an argon atmosphere at 30 °C, then irradiated by a 3 W 410-420 nm LED at a distance of about 0 cm for another 72 h. The solvent was removed under reduced pressure, and the residue was purified by flash chromatography on *silica gel* (PE/EA =

100/1–50/1 ratio) to give the desired product **2a** (1.34g) in 87% yield.

(4) One-pot synthesis

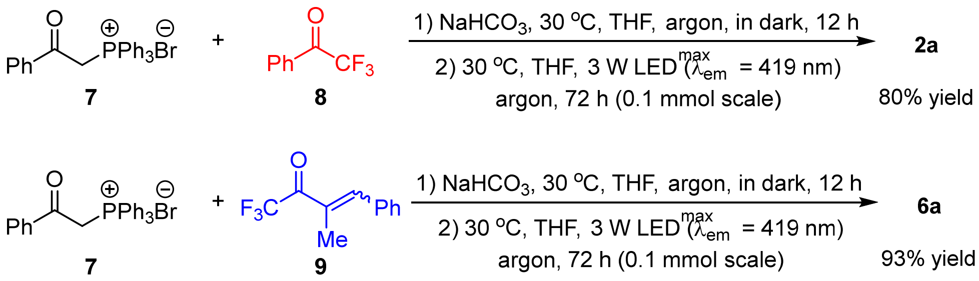

7 (0.12 mmol, 1.2 equiv.) and **8** (0.1 mmol, 1.0 equiv.) or **9** (0.1 mmol, 1.0 equiv.) in THF (2.0 mL) were sequentially added into the Schlenk tube, and the reaction was degassed three times by freeze-pump-thaw method. The reaction mixture was stirred under an argon atmosphere at 30 °C for 12 h in dark. Then irradiated by a 3 W 419 nm LED at a distance of about 0 cm for another 72 h. The solvent was removed under reduced pressure, and the residue was purified by flash chromatography on *silica gel* (PE/EA = 100/1) to give the desired products **2a** (22.2 mg) in 80% yield or **6a** (29.4 mg) in 93% yield.

4. Experimental equipment

(1) Incubator and setup of reactions

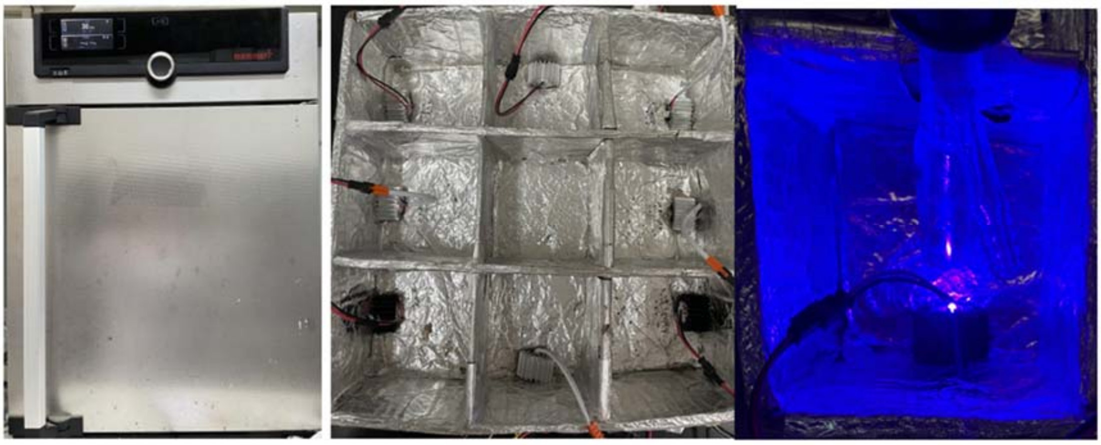

Fig. S1. Incubator and setup of reactions.

(2) Emission spectrum of the LED light

The following spectrum was recorded by a QE Pro fiber-optic spectrometer which was placed at a fixed distance of 0.5 cm from the used 3 W LED light.

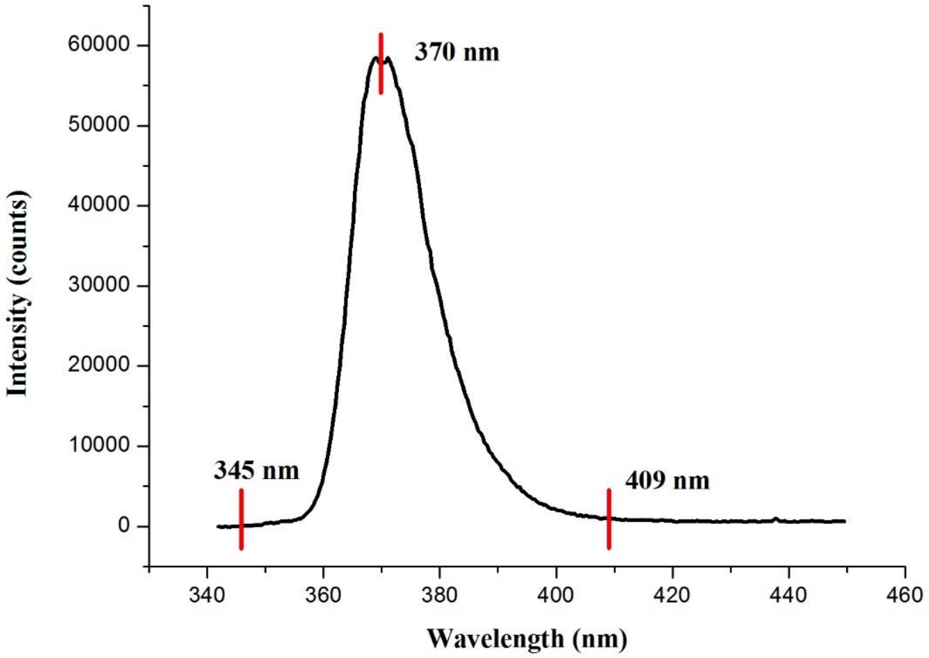

Fig. S2. Emission spectrum of the 3 W LED light (370 nm)

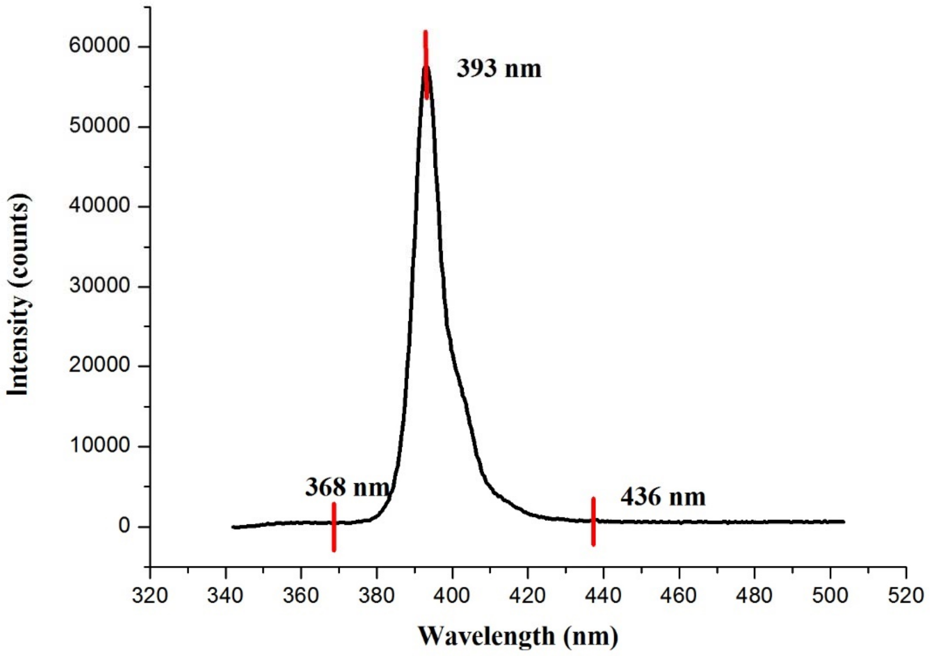

Fig. S3. Emission spectrum of the 3 W LED light (393 nm)

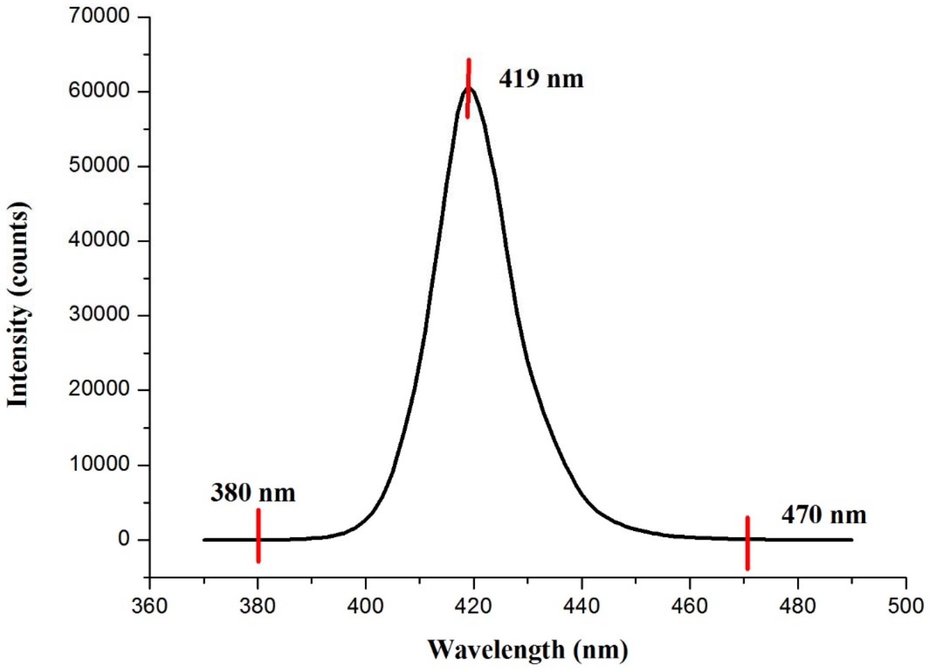

Fig. S4. Emission spectrum of the 3 W LED light (419 nm)

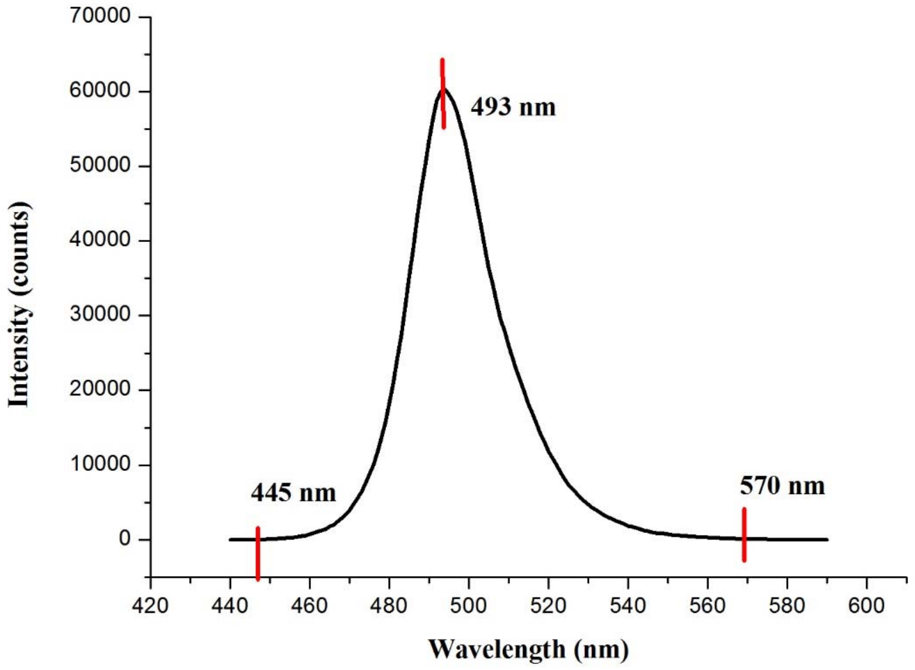

Fig. S5. Emission spectrum of the 3 W LED light (493 nm)

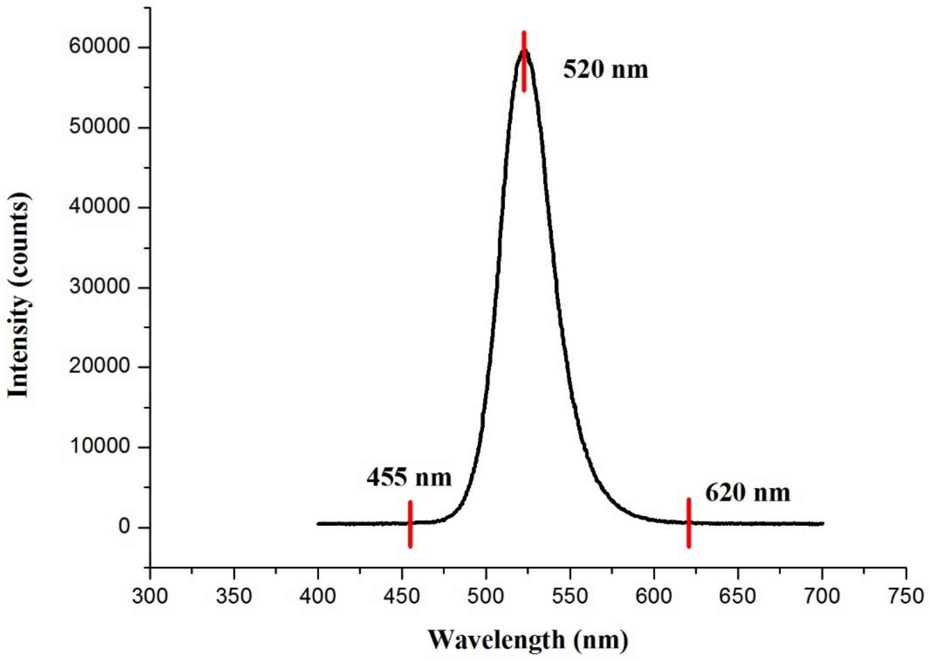

Fig. S6. Emission spectrum of the 3 W LED light (520 nm)

5. Mechanism studies

(1) Radical trapping experiments

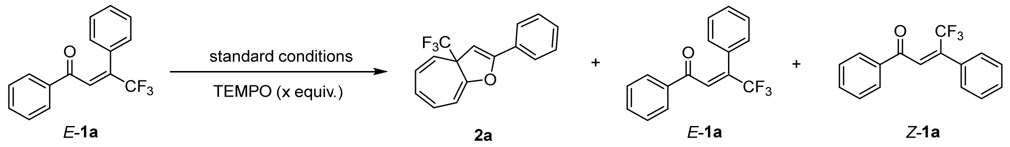

E-**1a** (0.1 mmol, 1.0 equiv.), TEMPO (x mmol, x equiv.) in THF (2.0 mL) were sequentially added into the 25 mL Schlenk tube, and the reaction was degassed three times by freeze-pump-thaw method. The reaction mixture was stirred under an argon atmosphere at 30 °C, then irradiated by a 3 W 410-420 nm LED at a distance of about 2 cm for another 12 h. The solvent was removed under reduced pressure, and the residue was purified by flash chromatography on *silica gel* (PE/EA = 100/1–50/1 ratio) to give the desired products **2a**, *E*-**1a** and *Z*-**1a**.

Table S1. Effect of TMEPO in reaction

TEMPO (x equiv.)	yield of **2a**	recovery of *E*-**1a**	yield of *Z*-**1a**
0	95%	trace	trace
1.0	87%	9%	4%
2.0	78%	14%	8%
3.0	53%	35%	12%
4.0	38%	43%	19%
5.0	13%	60%	27%

Comments: 5.0 equiv. of TEMPO was added to the standard conditions as a radical scavenger, and the experimental results confirmed the existence of a radical process.

(2) UV-vis absorption spectra

UV-vis absorption spectroscopy was performed using a spectrophotometer, equipped with a temperature control unit at 25 °C. The samples were measured in 3.0 mL quartz cuvettes fitted with a PTFE stopper. **1a** and **5a** were prepared as a 0.05 mM solution with fresh THF as the solvent for measurement.

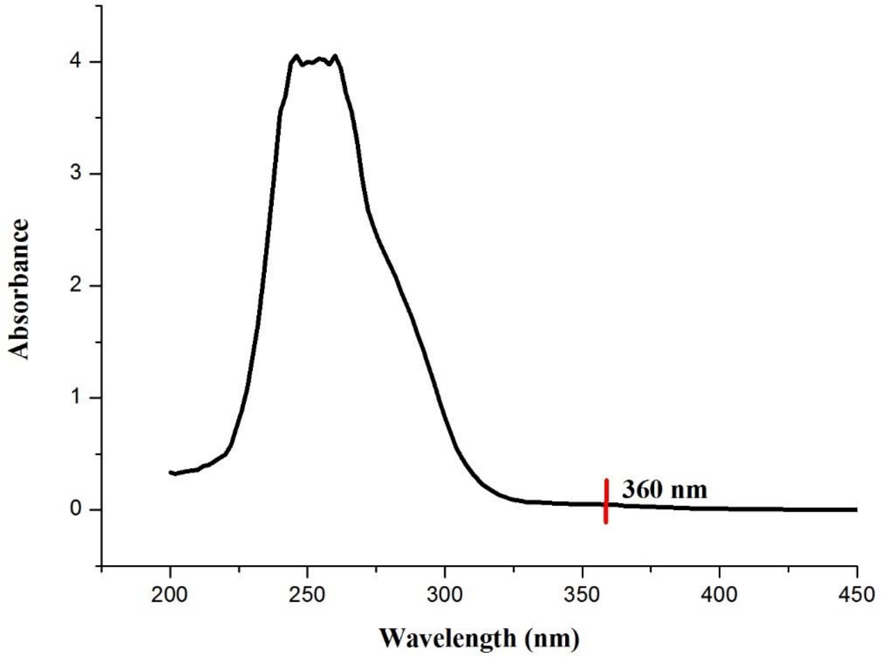

Fig. S7. UV-vis absorption spectra of **1**.

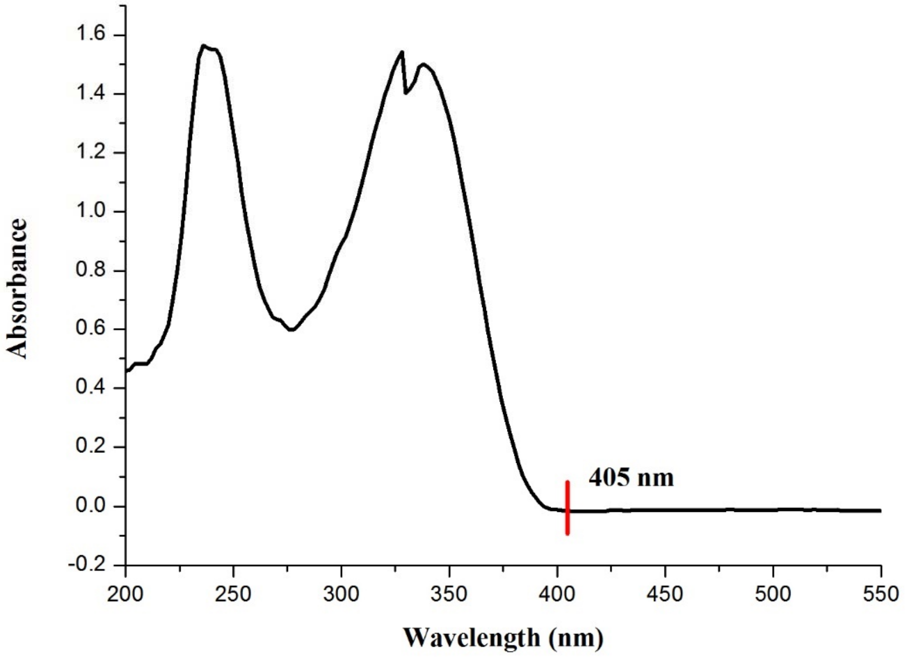

Fig. S8. UV-vis absorption spectra of **5**.

(3) Concentration effect for the transformation of *E*-1a

Table S2. Concentration effect for the transformation of *E*-**1a**

conc. of **1a** (mol/L)	yield of **2a**	recovery of *E*-**1a**	yield of *Z*-**1a**
2.0	92%	N.D.	N.D.
1.0	93%	N.D.	N.D.
0.5	95%	N.D.	N.D.
0.3	83%	trace	12%
0.1	78%	8%	14%

0.1 mmol scale. N.D. = not detected.

Comments: Concentration is critical for high yields. When the concentration is reduced, such as 0.1 mol/L, **2a** is obtained in 78% yield. At the same time, *Z*-**1a** was isolated in 14% yield, demonstrating the viability of such a transformation by direct photoexcitation.

6. DFT caculation

(1) Computation Details

All the calculations in this work were performed on the basis of density functional theory (DFT) in the Gaussian G16 package (Revision B.01).[1] All the reactants, complexes, transition states, intermediates and products were fully optimized at the M06-2X[2]/6-31+G(d, p)[3-5] level of theory. The nature of the local minima was established with analytical frequencies calculations and the single point energies were subsequently obtained at the same functional and basis set with SMD (tetrahydrofuran) solvent model[6] and the temperature was set to 303.15 k in terms of the experiment temperature. Intrinsic reaction coordinate (IRC)[7-8] calculations were carried out to ascertain the true nature of the transition states. Three dimensional diagrams of the computed species were generated by using CYL view visualization software[9].

(2) Plausible reaction pathways for the reaction with alkenes

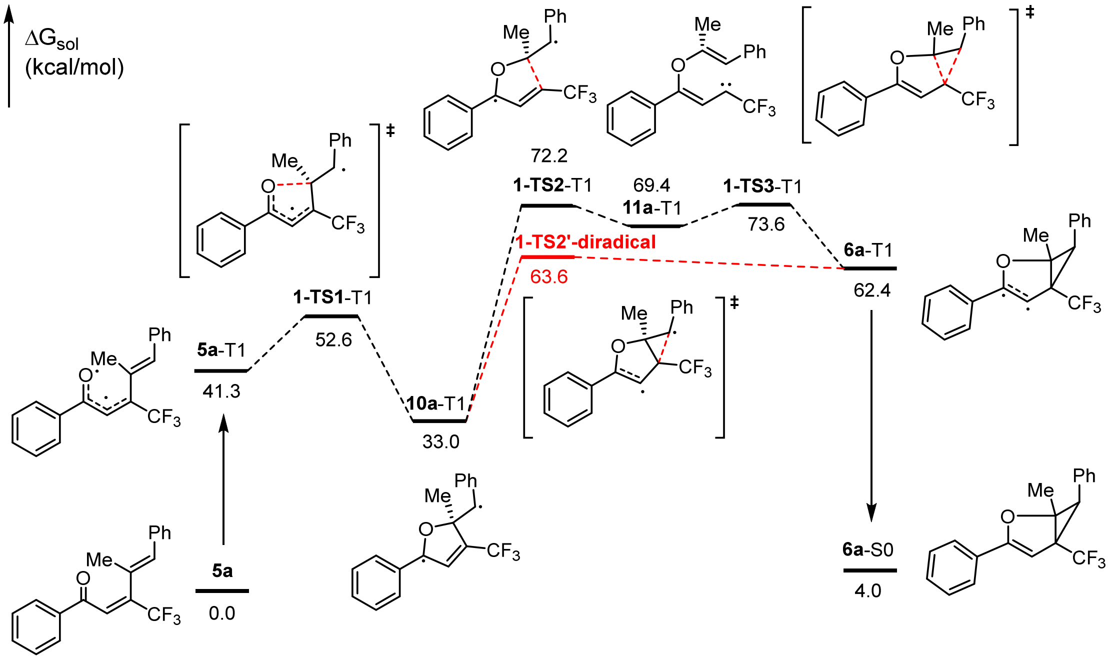

Scheme S1. The energy profiles for the reaction with alkenes.

We noticed that the dihydrofuran-based cyclopropanes become the major product when the alkenes substrates are performed. We further examined whether the proposed mechanism is capable to rationalize this intriguing phenomenon. The calculated energy profiles for the formation of cyclopropanes are shown in Scheme S1. Starting from the excitation of **5a** to **5a**-T1, it undergoes the radical addition via transition states **1-TS1**-T1 with an activation barrier of 11.3 kcal/mol,

resulting in a diradical intermediate **1-10a**-T1. Then two pathways were considered, i.e. carbene mechanism and diradical mechanism. Along the pathway shown in black, the C-C bond cleavage occurs through transition states **1-TS2**-T1 by crossing an activation barrier of 39.2 kcal/mol, leading to the generation of carbene intermediate **1-11a**-T1. Alternatively, the diradical recombination could undergo via transition state **1-TS2′-diradical** with an activation barrier of 30.6 kcal/mol, giving to **6a-T1** (the pathway shown in red). The calculated results show that diradical recombination pathway is more favored than the carbene pathway by 8.6 kcal/mol.

(3) Cartesian coordination and energies (in Hartree) for the calculated species were performed at the M06-2X/6-31+G level in solvent**

E-**1a**

Sum of electronic and thermal Free Energies = -990.567872

C	-4.04734000	0.83656300	1.23158700
C	-2.77197500	0.83539200	0.66936900
C	-2.48295100	-0.01248000	-0.40601000
C	-3.47637300	-0.85836600	-0.91512900
C	-4.74947600	-0.84994200	-0.35694300
C	-5.03515300	-0.00243800	0.71709000
H	-4.26883900	1.49106000	2.06862700
H	-2.00456000	1.48764700	1.07720900
H	-3.23637500	-1.51244100	-1.74763400
H	-5.52071900	-1.50169500	-0.75543700
H	-6.02995800	0.00234100	1.15250500
C	-1.12157400	-0.05515400	-1.01524100
C	-0.14770100	1.01846000	-0.62158900
H	-0.50006400	2.04394000	-0.70302900
O	-0.80909200	-0.90025200	-1.83436000
C	1.10112300	0.76522100	-0.22062600
C	1.65861900	-0.58804500	0.04943800
C	2.83611800	-1.03245500	-0.56341600
C	0.98914800	-1.43034200	0.94545900

C	3.32537000	-2.30853100	-0.29066700
H	3.35878200	-0.39491700	-1.26896700
C	1.48223100	-2.70412600	1.21647400
H	0.08371700	-1.07991800	1.43505900
C	2.65186800	-3.14583500	0.59824800
H	4.23328500	-2.64927400	-0.77877700
H	0.95508800	-3.34837700	1.91343500
H	3.03754100	-4.13866100	0.80906900
C	2.01579500	1.92851000	0.07492500
F	2.50464700	1.86265900	1.32843500
F	1.41291900	3.12177000	-0.04523000
F	3.08226200	1.95154200	-0.75042600

E-**1a**-T1

Sum of electronic and thermal Free Energies= -990.495892

C	-4.61250400	-0.01775300	1.31849400
C	-3.26149900	0.22331200	1.07448300
C	-2.69103900	-0.15417000	-0.14705100
C	-3.48765900	-0.77146200	-1.11935100
C	-4.83278300	-1.02194600	-0.86872300
C	-5.39738500	-0.64495400	0.35158800
H	-5.05149000	0.28754000	2.26320900
H	-2.67003300	0.72803000	1.83204500
H	-3.03792100	-1.05311200	-2.06631100
H	-5.44215800	-1.50911500	-1.62366400
H	-6.44828500	-0.83678400	0.54644000
C	-1.25153300	0.09295900	-0.47573400
C	-0.29670100	0.30339100	0.60107400
H	-0.61265400	0.27798800	1.64007800
O	-0.86266400	0.09663000	-1.64712800
C	1.11903600	0.53871400	0.28816300
C	2.07382700	-0.52170800	0.21926200

C	3.44726500	-0.29612800	-0.05834500
C	1.64836400	-1.85740000	0.43746900
C	4.33735300	-1.35768800	-0.11325100
H	3.81571500	0.70930300	-0.22956700
C	2.54904900	-2.90915800	0.37805500
H	0.60178200	-2.05824700	0.65028500
C	3.89838500	-2.66762000	0.10297500
H	5.38389100	-1.16442500	-0.32727800
H	2.20059100	-3.92360800	0.54496000
H	4.60198700	-3.49280100	0.05695700
C	1.52574000	1.95303500	0.01316900
F	2.44942600	2.41369300	0.88945100
F	0.47767700	2.79293400	0.08690900
F	2.06760700	2.11711100	-1.21233800

TS1-T1

Sum of electronic and thermal Free Energies = -990.469478

C	-4.69577100	-1.11285300	0.06205600
C	-3.30734900	-1.16955100	0.06740000
C	-2.54623000	0.01318000	0.00239600
C	-3.21773400	1.24767900	-0.06191000
C	-4.60853900	1.29489600	-0.06605400
C	-5.35529000	0.11766300	-0.00527100
H	-5.26802800	-2.03437100	0.11287300
H	-2.81606500	-2.13543300	0.12741600
H	-2.63919400	2.16444400	-0.11076000
H	-5.11160000	2.25595500	-0.11799600
H	-6.44022100	0.15624000	-0.00885600
C	-1.08449900	-0.01751600	0.00273500
C	-0.27434200	-1.17009400	-0.00534300
H	-0.67058100	-2.17791100	-0.01712300
O	-0.44222000	1.13258900	0.00416500

C	1.06786500	-0.88557700	-0.00649200
C	1.45941600	0.55368700	0.00551500
C	1.86255400	1.16525600	1.24295700
C	1.87296400	1.18299900	-1.21940600
C	2.48561200	2.39310600	1.24546600
H	1.62444200	0.65291600	2.17038900
C	2.49587600	2.41082700	-1.19882200
H	1.64279700	0.68414100	-2.15611500
C	2.79281400	3.02715200	0.02909700
H	2.75196500	2.86764600	2.18419000
H	2.77006400	2.89897700	-2.12827600
H	3.28288900	3.99560200	0.03814700
C	2.13036500	-1.91178600	-0.01804500
F	2.95998900	-1.80164700	-1.08819900
F	1.63993300	-3.16402200	-0.04034700
F	2.94952600	-1.83692300	1.06355900

7a-T1

Sum of electronic and thermal Free Energies = -990.483720

C	4.79391900	1.03739600	-0.01074700
C	3.41209600	1.17198800	-0.01278000
C	2.58680000	0.02956600	0.00136900
C	3.18545500	-1.24476200	0.01784700
C	4.57094900	-1.36755200	0.01958800
C	5.38172800	-0.23139800	0.00527100
H	5.41817000	1.92574700	-0.02155500
H	2.96906100	2.16323700	-0.02504600
H	2.55774400	-2.12949600	0.02854900
H	5.02030400	-2.35598700	0.03193000
H	6.46262500	-0.33145900	0.00659000
C	1.14952300	0.15694300	-0.00139600
C	0.32670400	1.29306600	-0.02191700

H	0.67073300	2.31919300	-0.03537000
O	0.40902300	-0.97816000	0.01854800
C	-0.97601100	0.87225200	-0.02124100
C	-1.02948100	-0.64882100	-0.00052600
C	-1.65349500	-1.19518800	1.25211600
C	-1.61914100	-1.23005600	-1.25490900
C	-2.67994700	-2.08983200	1.22265500
H	-1.24582900	-0.82655900	2.18944600
C	-2.64355500	-2.12705200	-1.23025600
H	-1.18515800	-0.88577100	-2.18969500
C	-3.20392300	-2.56986100	-0.00507500
H	-3.10323400	-2.44671700	2.15701900
H	-3.03847800	-2.51196000	-2.16588800
H	-4.02072700	-3.28240700	-0.00615700
C	-2.17903200	1.72740200	-0.00348000
F	-3.06011200	1.41467100	-0.98147600
F	-1.87185900	3.02920600	-0.15595400
F	-2.88057700	1.63200200	1.15328300

TS2-T1

Sum of electronic and thermal Free Energies = -990.446073

C	4.73899000	1.17077500	-0.10362500
C	3.35116000	1.20972700	-0.09001300
C	2.60526700	0.01810900	0.00890900
C	3.29358100	-1.20742300	0.08949400
C	4.68453300	-1.23515500	0.07528900
C	5.41467300	-0.05019500	-0.02041000
H	5.29852700	2.09797400	-0.18134700
H	2.84626700	2.16778000	-0.16139300
H	2.73269800	-2.13252400	0.16466900
H	5.20054500	-2.18833800	0.13996000
H	6.49988800	-0.07532100	-0.03138900

C	1.15673600	0.04524900	0.02712300
C	0.30978300	1.15645000	0.01139500
H	0.71196600	2.16684300	-0.01367500
O	0.55391200	-1.17475400	0.09436900
C	-1.02197700	0.89002800	0.04260800
C	-0.84019000	-1.24202800	0.02687600
C	-1.53338000	-1.57763500	1.21541600
C	-1.42655900	-1.50654500	-1.23668200
C	-2.86816100	-1.93969800	1.13310100
H	-1.01757100	-1.48848100	2.16627600
C	-2.76171700	-1.86920100	-1.29263900
H	-0.82985500	-1.36409500	-2.13243500
C	-3.49379300	-2.06247700	-0.11373700
H	-3.42483100	-2.14446700	2.04218500
H	-3.23656800	-2.01982000	-2.25704400
H	-4.53921400	-2.34846000	-0.16798000
C	-2.20624500	1.74709600	0.03400000
F	-2.97950900	1.56477700	-1.06308400
F	-1.90088700	3.06879700	0.06129300
F	-3.01842100	1.52692300	1.09452500

TS1′-T1

Sum of electronic and thermal Free Energies= -990.472097

C	-4.57322400	-1.20640900	0.22550700
C	-3.18348700	-1.14323900	0.21970800
C	-2.52896000	0.09123400	0.06850800
C	-3.30328200	1.25671100	-0.05489700
C	-4.69330300	1.18670400	-0.04953400
C	-5.33524400	-0.04429700	0.08833200
H	-5.06365200	-2.16774700	0.34543700
H	-2.61507700	-2.05829500	0.35069200
H	-2.80332900	2.21330000	-0.16332800

H	-5.27671000	2.09669700	-0.15358500
H	-6.41955500	-0.09873000	0.09463000
C	-1.05363500	0.20522400	0.06198800
C	-0.21183400	-0.90057100	-0.18338200
H	-0.66627500	-1.86990000	-0.34925400
O	-0.56832500	1.39444500	0.28573900
C	1.17357400	-0.79480500	-0.17919500
C	1.82318000	0.49998000	-0.11586400
C	2.98863400	0.76947100	0.61281600
C	1.16382100	1.58406800	-0.79262600
C	3.44077900	2.07831400	0.73541600
H	3.51128900	-0.02957400	1.12865400
C	1.64429700	2.90931900	-0.64699200
H	0.53125700	1.35214100	-1.64351700
C	2.75723000	3.15567300	0.12518500
H	4.32704000	2.27969500	1.32942200
H	1.14107300	3.71326200	-1.17431800
H	3.13556100	4.16580500	0.24201500
C	2.00219700	-2.02935800	-0.11457500
F	3.11989900	-1.94715000	-0.87092500
F	1.33564400	-3.12435800	-0.52571500
F	2.44229200	-2.31297500	1.14162500

7a′-T1

Sum of electronic and thermal Free Energies = -990.484259

C	-4.53952200	-1.32488300	0.19724500
C	-3.15448300	-1.25375100	0.12921400
C	-2.50836300	-0.01064400	-0.03055300
C	-3.30016500	1.15061300	-0.13110800
C	-4.68693800	1.06800800	-0.06644000
C	-5.31511700	-0.16652400	0.09987200
H	-5.01820900	-2.29136200	0.32178700

H	-2.57855600	-2.17046500	0.19491200
H	-2.82185900	2.11534200	-0.25501900
H	-5.27972400	1.97441700	-0.14370800
H	-6.39767300	-0.22798000	0.15111200
C	-1.06140600	0.08719300	-0.09919300
C	-0.14947400	-0.96016700	0.08792200
H	-0.52896200	-1.93025400	0.38508700
O	-0.60691300	1.34516800	-0.37458800
C	1.19377000	-0.78103100	-0.11678700
C	1.70010900	0.55516000	-0.62694300
C	3.11742500	0.93109800	-0.28364900
C	0.73291400	1.61527400	-0.19594600
C	3.44528600	2.16361000	0.19859900
H	3.89430200	0.20544300	-0.50147200
C	1.09145100	2.83101900	0.29093800
H	1.64284500	0.47927600	-1.73579500
C	2.46155100	3.14591900	0.47722300
H	4.49033300	2.39718400	0.38123900
H	0.31430700	3.54364100	0.55044500
H	2.74602400	4.11798000	0.86159800
C	2.14970800	-1.90479300	0.05255700
F	2.93266600	-2.07058200	-1.04115300
F	1.54229900	-3.08337200	0.27648200
F	3.00636900	-1.71839900	1.08565100

TS2′-T1

Sum of electronic and thermal Free Energies = -990.444132

C	4.56451400	-1.31970100	0.03755800
C	3.17723000	-1.24716600	0.06198400
C	2.52535600	0.00090600	0.02480400
C	3.31045600	1.16855400	-0.01682700
C	4.69910700	1.08648500	-0.03687600

C	5.33423700	-0.15521100	-0.01360900
H	5.04812300	-2.29121800	0.06862700
H	2.60467700	-2.16621700	0.12851600
H	2.82689600	2.13841700	-0.03990700
H	5.28734300	1.99842300	-0.07281800
H	6.41794000	-0.21669600	-0.02924100
C	1.07019600	0.09850000	0.03796100
C	0.19172300	-0.97370800	-0.15501200
H	0.63586200	-1.93373900	-0.41443400
O	0.62388900	1.37544000	0.21540700
C	-1.16530600	-0.87731700	-0.04068300
C	-1.59363400	0.93814000	1.05702100
C	-2.96893500	1.25848700	1.02106700
C	-0.72919800	1.63977000	0.18486100
C	-3.45501000	2.15615100	0.07978000
H	-3.64046600	0.77924700	1.72618800
C	-1.20379600	2.54978700	-0.74145100
H	-1.17983800	0.42138700	1.91847000
C	-2.57772400	2.80533400	-0.79604500
H	-4.51736200	2.37508300	0.03925800
H	-0.50833200	3.03714300	-1.41708100
H	-2.96016200	3.51512300	-1.52240900
C	-2.18376400	-1.92311100	-0.16156300
F	-2.80109200	-2.17758000	1.01921400
F	-1.69171000	-3.11076700	-0.59151300
F	-3.17147600	-1.58897200	-1.02395100

TS2'-diradical

Sum of electronic and thermal Free Energies = -990.428623

C	-4.69428100	-1.09404300	0.40587900

C	-3.30548600	-1.09305600	0.46943300
C	-2.58165700	0.04307200	0.07449800
C	-3.27341600	1.18181000	-0.36451200
C	-4.66453300	1.17584000	-0.41871100
C	-5.37876400	0.03964400	-0.03797600
H	-5.24517300	-1.97769300	0.71289000
H	-2.78174400	-1.97001300	0.83818000
H	-2.72004200	2.06500500	-0.66568600
H	-5.19166800	2.06119700	-0.76066400
H	-6.46361800	0.03814000	-0.08077600
C	-1.12159800	0.03114200	0.11043500
C	-0.26037500	-1.01827000	0.22200800
H	-0.57227700	-2.05275600	0.13407500
O	-0.51731600	1.23455800	-0.09652800
C	1.10794600	-0.60728700	0.31001400
C	0.86464300	1.19011500	0.00153200
C	1.43746500	0.57668600	1.20803200
C	1.61093700	1.93917900	-0.89029500
C	2.84542900	0.93716100	1.48796400
H	0.78488100	0.58250000	2.08457100
C	3.02133000	2.17779400	-0.60025800
H	1.14697500	2.31702000	-1.79401900
C	3.58547700	1.71286600	0.55830900
H	3.31770200	0.50725100	2.36305100
H	3.60295100	2.76667900	-1.30098100
H	4.62581200	1.93564700	0.77713600
C	2.11438700	-1.52122900	-0.32053200
F	3.39422100	-1.23081800	-0.05827500
F	1.99200100	-1.54046800	-1.66793500
F	1.92374300	-2.80404400	0.07838000

8a-T1

Sum of electronic and thermal Free Energies= -990.455733

C	-4.55590900	1.25828100	-0.07772900
C	-3.17462900	1.25381800	0.07373200
C	-2.46486000	0.03824400	0.10106300
C	-3.18288300	-1.16700500	-0.00572100
C	-4.56597100	-1.15349700	-0.15522900
C	-5.25881600	0.05671400	-0.19555500
H	-5.08878900	2.20401600	-0.09312300
H	-2.65308900	2.19783800	0.19365000
H	-2.64804600	-2.11016000	0.01980000
H	-5.10397500	-2.09246500	-0.24319100
H	-6.33815600	0.06531800	-0.31160700
C	-1.01303500	0.00283600	0.23974100
C	-0.17082300	1.10016000	0.03875500
H	-0.63182300	2.01983300	-0.32141800
O	-0.53306600	-1.20079500	0.65557400
C	1.17337500	1.10985100	0.24878300
C	0.72892400	-1.62415800	0.26718800
C	1.07033400	-1.68495900	-1.08264500
C	1.60333800	-2.04555400	1.26288700
C	2.33311200	-2.15554400	-1.43326000
H	0.36123500	-1.35451500	-1.83664900
C	2.86208200	-2.52041600	0.89581200
H	1.29741100	-1.98705500	2.30262800
C	3.23170400	-2.57050400	-0.44810300
H	2.61404400	-2.19969900	-2.48098000
H	3.55538900	-2.84582700	1.66515600
H	4.21486100	-2.93461300	-0.72879300
C	2.21336300	2.11373300	0.01393000
F	2.96753500	2.34188400	1.11058100
F	1.70069700	3.30819800	-0.35886700

F	3.07728200	1.74677200	-0.95971100

9a-T1

Sum of electronic and thermal Free Energies = -990.432504

C	4.75576300	1.04062900	0.39857300
C	3.36698100	1.09629800	0.42799600
C	2.60806100	-0.02042000	0.04878600
C	3.26144000	-1.19341600	-0.34889100
C	4.65414100	-1.24410600	-0.37398800
C	5.40450900	-0.12983100	-0.00272900
H	5.33445500	1.91049500	0.69376800
H	2.87117500	2.00708200	0.75074000
H	2.67910200	-2.06208700	-0.63722400
H	5.15209000	-2.15772700	-0.68408800
H	6.48928500	-0.17150400	-0.02162500
C	1.14201600	0.04251300	0.05959100
C	0.30413800	1.07177700	0.28758000
H	0.58428800	2.10046600	0.46991100
O	0.49448200	-1.12921500	-0.23673000
C	-1.08213000	0.57115800	0.23001500
C	-0.89907500	-0.93584000	-0.10093300
C	-1.42159400	-0.55280300	1.24498300
C	-1.72041300	-1.74069200	-0.99526400
C	-2.81123200	-0.93501700	1.53253600
H	-0.71936700	-0.56203300	2.07673500
C	-3.01726400	-2.21924400	-0.54755900
H	-1.34747500	-1.95004700	-1.99075600
C	-3.53084800	-1.83949500	0.64854300
H	-3.30788800	-0.49596600	2.39002200
H	-3.56803900	-2.89242600	-1.19663300
H	-4.50283400	-2.20423200	0.96696700
C	-2.13274100	1.47532300	-0.33180800

F	-3.26921100	0.84741200	-0.66627500
F	-1.69746400	2.10910200	-1.43924000
F	-2.46789800	2.44245100	0.55122900

8a-S0

Sum of electronic and thermal Free Energies = -990.459585

C	4.45115100	0.92565600	-0.10421700
C	3.06320500	0.97819600	-0.14087700
C	2.31546000	-0.20595900	-0.02076600
C	2.97654300	-1.43784500	0.13253800
C	4.36348600	-1.47880300	0.18074700
C	5.10081500	-0.29874300	0.06114000
H	5.02697400	1.83901900	-0.21014700
H	2.56637800	1.92983000	-0.29471500
H	2.40024700	-2.35144200	0.23067200
H	4.87124900	-2.42864500	0.31183400
H	6.18556000	-0.33416000	0.09385300
C	0.85325800	-0.16684800	-0.06431700
C	0.11536400	1.02228300	0.20347200
H	0.68642400	1.79461200	0.71903500
O	0.33265300	-1.31953800	-0.39564000
C	-1.15341600	1.18263300	-0.28346900
C	-1.00328500	-1.70158300	-0.17344000
C	-1.47531300	-1.78606700	1.12815500
C	-1.73655400	-2.11463900	-1.27237000
C	-2.77111200	-2.25781200	1.32399600
H	-0.85324800	-1.47354800	1.96135100
C	-3.03053500	-2.58610700	-1.05722100
H	-1.31265400	-2.04081400	-2.26828800
C	-3.55135200	-2.65076800	0.23520000
H	-3.16783400	-2.32170300	2.33226900
H	-3.63166200	-2.89935800	-1.90483800

H	-4.56102800	-3.01495100	0.39554500
C	-1.69518900	2.55297700	-0.03267400
F	-1.95258700	3.18317300	-1.20319400
F	-0.93804300	3.44337500	0.68074900
F	-2.86567900	2.48434700	0.64501800

TS3-S0

Sum of electronic and thermal Free Energies = -990.436558

C	-4.52483600	1.14166200	0.38080900
C	-3.13820800	1.08169700	0.45017400
C	-2.46126700	-0.09372100	0.08539700
C	-3.20175700	-1.21169000	-0.33130400
C	-4.58957300	-1.14434100	-0.39864300
C	-5.25481400	0.03049600	-0.04635000
H	-5.03813000	2.05411500	0.66733300
H	-2.57941700	1.94436000	0.80096600
H	-2.69061900	-2.12845200	-0.60411200
H	-5.15314100	-2.01207300	-0.72679800
H	-6.33811700	0.07912700	-0.09886700
C	-0.99935700	-0.15225000	0.17996700
C	-0.16428700	0.98161200	0.15542300
H	-0.64557600	1.83052500	-0.33600700
O	-0.50908400	-1.37998200	0.00067900
C	1.13759700	1.10535000	0.56131500
C	0.83176900	-1.62643600	0.12510800
C	1.42454700	-2.43381800	-0.85247900
C	1.55543300	-1.05408600	1.17958800
C	2.78239000	-2.68001100	-0.77301000
H	0.81489800	-2.83293100	-1.65695300
C	2.93927900	-1.36000200	1.25578400
H	1.01626000	-0.69543800	2.04729100
C	3.53957900	-2.13523000	0.28705900

H	3.26566100	-3.29091000	-1.52829100
H	3.51825300	-0.96116900	2.08233500
H	4.60270100	-2.34679600	0.34759500
C	1.97757100	2.06427700	-0.22024900
F	2.73043000	2.85518300	0.57883100
F	1.33956400	2.92917300	-1.05800100
F	2.83839400	1.36213800	-0.99099100

9a-S0

Sum of electronic and thermal Free Energies = -990.512294

C	4.74710900	1.08301300	0.36321100
C	3.35804600	1.12414000	0.39561800
C	2.61031700	-0.01075400	0.04989900
C	3.27553400	-1.18636100	-0.31806500
C	4.66874300	-1.22201600	-0.34792100
C	5.40800700	-0.09022000	-0.00953200
H	5.31680100	1.96723900	0.63219800
H	2.85325400	2.03869400	0.69303000
H	2.70231700	-2.06929300	-0.58032100
H	5.17556000	-2.13808000	-0.63577800
H	6.49310500	-0.11992800	-0.03218100
C	1.14376900	0.03665900	0.06132100
C	0.29212400	1.04581600	0.29501000
H	0.54975000	2.07355400	0.51016600
O	0.50564500	-1.14541500	-0.23209700
C	-1.09637700	0.52669800	0.21448500
C	-0.88082200	-0.94463900	-0.10992600
C	-1.42705800	-0.53721200	1.26316300
C	-1.69663100	-1.82054700	-0.95622300
C	-2.80743700	-0.97627400	1.53400800
H	-0.74529700	-0.51720100	2.10934300
C	-2.95711800	-2.13526300	-0.61165800

H	-1.21995500	-2.21981900	-1.84633300
C	-3.53411200	-1.67988500	0.64347500
H	-3.20533000	-0.75957500	2.52128300
H	-3.54802100	-2.77942200	-1.25515100
H	-4.54631400	-1.98402300	0.89115600
C	-2.15174600	1.43752900	-0.33664900
F	-3.12629800	0.82697900	-0.99150800
F	-1.63806000	2.33011400	-1.18348200
F	-2.74509900	2.15035800	0.62699800

TS4-S0

Sum of electronic and thermal Free Energies = -990.504647

C	4.64251900	-1.26681300	-0.32515500
C	3.24918500	-1.22620600	-0.30485100
C	2.58820000	-0.04525600	0.05231800
C	3.33604100	1.09262100	0.38641400
C	4.72504200	1.04536700	0.36580200
C	5.38285400	-0.13486300	0.00999700
H	5.14824600	-2.18651100	-0.60273700
H	2.67507600	-2.10971000	-0.56309900
H	2.83055000	2.01486500	0.65746000
H	5.29679300	1.93106400	0.62499900
H	6.46794500	-0.16863800	-0.00609400
C	1.12242900	0.00730400	0.07514100
C	0.26767300	0.95950400	0.47269600
H	0.51219900	1.93072700	0.87947500
O	0.47605200	-1.12766700	-0.36555700
C	-1.12993000	0.43886300	0.30142300
C	-0.88658100	-0.91445900	-0.24993100
C	-1.59402500	-0.42409400	1.44289600
C	-1.75154100	-1.77048500	-0.96183100
C	-2.86268300	-1.02497500	1.60167200

H	-0.93604900	-0.38437500	2.30647000
C	-3.07569100	-1.96841400	-0.63689000
H	-1.29035400	-2.35137800	-1.75574800
C	-3.62883900	-1.59642700	0.60605100
H	-3.19438700	-1.13943100	2.63169000
H	-3.67259900	-2.58931700	-1.29852300
H	-4.62363200	-1.95474100	0.85282500
C	-2.05002300	1.43787100	-0.36309800
F	-3.29526500	1.00253400	-0.57537100
F	-1.55935400	1.82199900	-1.55699400
F	-2.14871100	2.55008300	0.39479000

2a-S0

Sum of electronic and thermal Free Energies = -990.524567

C	4.68521300	0.90197600	0.70501100
C	3.29588400	0.93318500	0.73530500
C	2.55563300	-0.09092700	0.12792000
C	3.22519700	-1.14426200	-0.50557500
C	4.61868200	-1.16954600	-0.53319100
C	5.35128700	-0.14929800	0.07008400
H	5.25074700	1.69847500	1.17854000
H	2.78582800	1.75221200	1.23370100
H	2.65722400	-1.93948300	-0.97634700
H	5.13026000	-1.98933900	-1.02789800
H	6.43659600	-0.17077000	0.04800700
C	1.08927900	-0.05352900	0.15430800
C	0.22418600	0.84599100	0.63720800
H	0.45888000	1.78275300	1.12310400
O	0.45921100	-1.14620000	-0.40593400
C	-1.18315000	0.36720700	0.35891300
C	-0.89482400	-0.99913600	-0.23417100
C	-2.03201500	0.24766700	1.60687400

C	-1.74010500	-1.99086600	-0.56440400
C	-3.16781700	-0.47097400	1.71509500
H	-1.67777600	0.80879500	2.46765700
C	-3.14983800	-2.02661600	-0.25359800
H	-1.31279800	-2.85221100	-1.07167100
C	-3.77935900	-1.35847000	0.74604400
H	-3.69671700	-0.38744000	2.66303500
H	-3.73838300	-2.73863700	-0.82666600
H	-4.83208500	-1.57679300	0.90563000
C	-1.82742800	1.30713100	-0.66918300
F	-3.06255700	0.93467500	-1.03525900
F	-1.09620100	1.38529900	-1.79643100
F	-1.92573300	2.55612000	-0.17380500

5a

Sum of electronic and thermal Free Energies = -1107.181972

C	-1.75928800	0.01016200	1.05306400
O	-1.23272700	-0.33748800	2.09569500
C	-2.74817500	-0.85215100	0.34408900
C	-3.16566100	-0.55749200	-0.95884000
C	-3.24276100	-1.98932000	0.99537500
C	-4.07186300	-1.39568800	-1.60596200
H	-2.77764200	0.31566000	-1.47576100
C	-4.15273200	-2.81897900	0.35050200
H	-2.90788500	-2.20717600	2.00475600
C	-4.56697000	-2.52268500	-0.95102100
H	-4.38939700	-1.16937100	-2.61889000
H	-4.54033600	-3.69693900	0.85774700
H	-5.27639200	-3.17299200	-1.45410700
C	-1.44265100	1.34805100	0.45177700
H	-2.28137400	1.98360100	0.17768700

C	-0.18517900	1.75087100	0.25872900
C	1.03000400	0.92242500	0.52781000
C	1.37548000	0.01361500	-0.39989400
H	0.78606000	-0.02381300	-1.31627500
C	0.06460200	3.10614200	-0.35277800
F	0.57516000	2.99000500	-1.59617200
F	0.95699400	3.82336200	0.35709100
F	-1.04344200	3.85712600	-0.45953600
C	2.49886700	-0.94303800	-0.33636900
C	3.21021400	-1.23320000	-1.51073900
C	2.85214800	-1.61204500	0.84468600
C	4.27006000	-2.13614100	-1.49851700
H	2.92990400	-0.73708900	-2.43675300
C	3.90780400	-2.52252700	0.85507800
H	2.28144300	-1.44014700	1.75236100
C	4.62495700	-2.78169800	-0.31281300
H	4.81572000	-2.34066500	-2.41494800
H	4.16432400	-3.03658900	1.77673200
H	5.44791500	-3.49007700	-0.30215900
C	1.74506200	1.19411400	1.82541400
H	1.70804400	2.25885300	2.07028200
H	1.24851200	0.65736300	2.64182100
H	2.79099800	0.88235900	1.78195900

5a-T1

Sum of electronic and thermal Free Energies = -1107.116127

C	-1.39489300	-0.23770700	0.45233600
O	-0.52131800	-0.83367900	1.10040400
C	-2.72328600	-0.88569400	0.20552100
C	-3.57486700	-0.48221300	-0.83040900
C	-3.10438300	-1.94997300	1.03213400

C	-4.79282400	-1.13034500	-1.02981000
H	-3.28575300	0.32236600	-1.50031700
C	-4.32401500	-2.59058500	0.83695600
H	-2.43479000	-2.26172000	1.82764300
C	-5.17138300	-2.18127500	-0.19484100
H	-5.44310100	-0.81687600	-1.84074600
H	-4.61529200	-3.40915500	1.48811700
H	-6.12210800	-2.68276000	-0.34938800
C	-1.17452900	1.10140200	-0.04554900
H	-2.04624300	1.68424600	-0.32026700
C	0.10306500	1.75545900	-0.06553200
C	1.32402900	1.14062300	-0.26368300
C	1.38759700	-0.25852900	-0.66168900
H	0.57385100	-0.62260000	-1.28266600
C	0.05840600	3.25897600	0.05132100
F	0.42641000	3.89319200	-1.08829100
F	0.87048200	3.73592300	1.01862200
F	-1.17446600	3.71458300	0.34121900
C	2.43197300	-1.18931800	-0.37694400
C	2.47058700	-2.40616900	-1.10361600
C	3.40629800	-0.99123400	0.63265900
C	3.45153000	-3.35521600	-0.85991100
H	1.71797200	-2.58456400	-1.86727300
C	4.38074800	-1.95008400	0.87422100
H	3.37552900	-0.09792100	1.24734600
C	4.41716200	-3.13158100	0.12740800
H	3.46560400	-4.27559500	-1.43590200
H	5.11329600	-1.78226100	1.65809800
H	5.18358100	-3.87566500	0.32044900
C	2.61771300	1.91921900	-0.21453700
H	2.51874400	2.89233200	-0.69921400

H	2.91740000	2.10184900	0.82356900
H	3.41645600	1.36993700	-0.71283300

1-TS1-T1

Sum of electronic and thermal Free Energies = -1107.098157

C	-1.41779500	-0.11142100	0.11209400
O	-0.35266800	-0.57273900	0.69581900
C	-2.60381800	-0.98084700	0.02510400
C	-3.77276900	-0.58331800	-0.64667500
C	-2.57027300	-2.24516500	0.63581700
C	-4.87649800	-1.42829800	-0.69753800
H	-3.82495700	0.38257400	-1.13861300
C	-3.67836900	-3.08573600	0.58293500
H	-1.66757200	-2.55750600	1.15053900
C	-4.83628900	-2.68191900	-0.08280900
H	-5.77165900	-1.10822300	-1.22233000
H	-3.63741800	-4.05951300	1.06184300
H	-5.70013600	-3.33828600	-0.12523400
C	-1.35632400	1.21627100	-0.35911800
H	-2.20772400	1.73751300	-0.77889400
C	-0.14172900	1.83717100	-0.11831300
C	0.97149100	0.99257100	0.32440000
C	1.55946900	0.12706700	-0.64066600
H	1.19676600	0.22619600	-1.66129500
C	-0.02539300	3.31542600	-0.10006000
F	1.20056700	3.74528100	-0.48011300
F	-0.23262400	3.87384800	1.12498100
F	-0.91807000	3.90248500	-0.92077800
C	2.53620400	-0.90742100	-0.40753300
C	3.27094300	-1.38880600	-1.51252100
C	2.76005800	-1.49390700	0.85692300

C	4.22837100	-2.38200300	-1.35234200
H	3.08615300	-0.96193700	-2.49482600
C	3.70866000	-2.49755600	1.00745600
H	2.15840700	-1.19134000	1.70717400
C	4.45397400	-2.93701100	-0.09033900
H	4.79417600	-2.73091100	-2.21062400
H	3.86223800	-2.94874700	1.98294100
H	5.19660100	-3.71904800	0.03523200
C	1.68196700	1.36113000	1.59873500
H	1.67360700	2.44686700	1.72883800
H	1.14652000	0.93568800	2.45412800
H	2.71619800	1.01667400	1.61445700

10a-T1

Sum of electronic and thermal Free Energies = -1107.129403

C	-1.50617500	0.24408000	0.03376600
O	-0.37576000	-0.28030900	0.56543500
C	-2.66965800	-0.60463500	-0.05591800
C	-3.89243300	-0.10088200	-0.54262900
C	-2.59674200	-1.95232900	0.34457400
C	-5.00548700	-0.92643400	-0.62629800
H	-3.96779600	0.93734500	-0.85179500
C	-3.71853900	-2.77009200	0.25712700
H	-1.65792300	-2.34884500	0.71733100
C	-4.92596900	-2.26444700	-0.22740300
H	-5.94190500	-0.52635500	-1.00300400
H	-3.64959900	-3.80838200	0.56739000
H	-5.79915700	-2.90589200	-0.29420800
C	-1.31253800	1.58315000	-0.33219900
H	-2.06689400	2.23213000	-0.75720800
C	-0.01400700	1.91516600	-0.04730200

C	0.69643700	0.71200100	0.53794300
C	1.81388000	0.21130500	-0.34303000
H	2.22073700	0.91680100	-1.06010200
C	0.56746500	3.26845500	-0.13836500
F	1.83455100	3.26911300	-0.61309400
F	0.63973300	3.89732400	1.06459400
F	-0.15348800	4.07642200	-0.93760900
C	2.42447200	-1.07170000	-0.26049500
C	3.52569900	-1.35206400	-1.11364200
C	1.99959300	-2.10173900	0.62064500
C	4.16537600	-2.58118700	-1.08393200
H	3.86565900	-0.57871100	-1.79768100
C	2.64985000	-3.32810100	0.64344700
H	1.15163800	-1.93666500	1.27565400
C	3.73376500	-3.57977200	-0.20359800
H	5.00515100	-2.76687400	-1.74707200
H	2.30695500	-4.10016900	1.32609300
H	4.23469200	-4.54243600	-0.17967400
C	1.16166800	0.94436900	1.98167700
H	1.94712700	1.70550600	1.99379100
H	0.32182200	1.28629200	2.59322900
H	1.56847900	0.02213000	2.40254100

1-TS2-T1

Sum of electronic and thermal Free Energies = -1107.066937

C	-1.88487300	-0.02514400	-0.07575300
O	-1.02473600	-1.08131400	-0.05834600
C	-3.29298800	-0.37965000	-0.07474900
C	-4.29343800	0.59369500	-0.26681800
C	-3.68140800	-1.71872300	0.11904300
C	-5.63499900	0.23516200	-0.25675900

H	-4.02476600	1.63204600	-0.43088600
C	-5.02811500	-2.06857800	0.12464600
H	-2.92216000	-2.47846200	0.26809400
C	-6.01138500	-1.09665200	-0.06120400
H	-6.39269900	0.99832400	-0.40610400
H	-5.31014800	-3.10603900	0.27681200
H	-7.06142000	-1.37190500	-0.05578900
C	-1.32769500	1.25373100	-0.15900200
H	-1.97587300	2.12749100	-0.20175000
C	0.02817800	1.37452300	-0.18822100
C	0.28093800	-0.84874800	0.37623400
C	1.24948300	-1.24567300	-0.49539100
H	0.90756800	-1.57122700	-1.47554200
C	0.90372600	2.54551700	-0.17116300
F	0.25567300	3.69725500	-0.47544800
F	1.92617300	2.44084600	-1.04861500
F	1.48119900	2.75383500	1.04022900
C	2.69101000	-1.25817900	-0.26882300
C	3.48378500	-2.08388900	-1.08772300
C	3.33288000	-0.46209600	0.69681400
C	4.86305800	-2.14389500	-0.92167900
H	3.00213800	-2.68638200	-1.85361900
C	4.71542600	-0.51836300	0.85504600
H	2.75892700	0.22881300	1.30496300
C	5.48495200	-1.36368800	0.05511200
H	5.45501300	-2.79511800	-1.55781100
H	5.19404800	0.10975200	1.60041700
H	6.56232500	-1.40474100	0.18316000
C	0.41066600	-0.65524000	1.86922000
H	0.71176600	0.35535100	2.15182000
H	-0.55680000	-0.86153400	2.33452900

H	1.14736300	-1.35793200	2.27000400

11a-T1

Sum of electronic and thermal Free Energies = -1107.071332

C	-1.78008800	-0.11744700	-0.16727100
O	-0.91733900	-1.14664600	-0.37354400
C	-3.18029300	-0.52403800	-0.09598100
C	-4.22866200	0.39807500	-0.27626300
C	-3.50065500	-1.87086500	0.15430600
C	-5.55248000	-0.01433400	-0.18450800
H	-4.01386400	1.43575600	-0.51039900
C	-4.82880500	-2.27620000	0.24193800
H	-2.70210300	-2.59262200	0.28730000
C	-5.86033900	-1.35153400	0.07734100
H	-6.34875700	0.70941800	-0.32876400
H	-5.05823700	-3.31841800	0.44197700
H	-6.89603700	-1.66968000	0.14549300
C	-1.33284900	1.20451000	-0.10057900
H	-2.07666600	1.97380400	0.10699200
C	-0.04218100	1.60748300	-0.26902400
C	0.36638900	-1.08320800	0.17109400
C	1.38024200	-1.27207200	-0.68390200
H	1.11961200	-1.43699700	-1.72759000
C	0.61461200	2.90900500	-0.12720800
F	-0.26831700	3.93309300	-0.09575500
F	1.47113500	3.16462000	-1.13754300
F	1.34454200	3.00396800	1.01044200
C	2.81244700	-1.28913200	-0.34150800
C	3.66967900	-2.16417700	-1.02694600
C	3.36427500	-0.42279500	0.61462600
C	5.03120500	-2.20072200	-0.73810300

H	3.25751200	-2.82381000	-1.78642700
C	4.72790600	-0.45780900	0.90142000
H	2.72874100	0.30226900	1.11607900
C	5.56473700	-1.35075000	0.23222400
H	5.67732300	-2.89065800	-1.27284200
H	5.13826700	0.22350900	1.64088700
H	6.62710800	-1.37527800	0.45525200
C	0.40032300	-0.96502600	1.66473800
H	0.34341400	0.07440400	2.00425900
H	-0.45541400	-1.50388400	2.08412400
H	1.31879900	-1.40356100	2.05934300

1-TS3-T1

Sum of electronic and thermal Free Energies = -1107.064742

C	-1.78474600	-0.12244800	0.15068200
O	-1.21838200	-1.33216300	0.42735800
C	-3.22457000	-0.21007200	-0.06830000
C	-3.97067600	0.90274000	-0.50399000
C	-3.89973700	-1.42680800	0.14783400
C	-5.34250800	0.80151900	-0.69873100
H	-3.48167300	1.84846600	-0.71076600
C	-5.27337100	-1.51952200	-0.05163400
H	-3.34381500	-2.29646100	0.47876000
C	-6.00345900	-0.40801100	-0.47249300
H	-5.89798900	1.67080400	-1.03718000
H	-5.77559300	-2.46595500	0.12458000
H	-7.07514100	-0.48290400	-0.62848100
C	-1.04631800	1.06789900	0.17614100
H	-1.60867700	1.99533700	0.07942600
C	0.31152500	1.11883900	0.31460200
C	0.14560900	-1.50107400	0.51434800

C	0.94749000	-0.91847100	-0.42102000
H	0.48176500	-0.60218000	-1.35231000
C	1.16496200	2.31145200	0.25065400
F	2.00223800	2.28602200	-0.81404800
F	1.95848700	2.44664000	1.33981200
F	0.47196000	3.47627500	0.15250900
C	2.42204800	-0.98483600	-0.41993600
C	3.09679800	-1.22964100	-1.62376300
C	3.17236200	-0.76083000	0.74261500
C	4.48814300	-1.28772000	-1.65795100
H	2.52344100	-1.37982300	-2.53502600
C	4.56403600	-0.81646100	0.70733000
H	2.66367400	-0.51680000	1.67170200
C	5.22617100	-1.08547600	-0.49111300
H	4.99622300	-1.48560500	-2.59706100
H	5.13209500	-0.63600500	1.61508200
H	6.31094800	-1.12452400	-0.51782200
C	0.52490100	-2.30187400	1.70939000
H	1.53121000	-2.71042900	1.61386900
H	0.47313900	-1.68882500	2.61816200
H	-0.18867500	-3.12344000	1.82576100

6a-T1

Sum of electronic and thermal Free Energies = -1107.082469

C	-1.87894500	0.18123600	0.26864600
O	-1.17791700	-0.43264300	1.26316200
C	-3.17958900	-0.20049500	-0.03796000
C	-3.89443800	0.47375200	-1.07911400
C	-3.84520000	-1.25989100	0.65890700
C	-5.18258800	0.09008500	-1.40631700
H	-3.41547300	1.28993600	-1.61231400

C	-5.13516700	-1.61464200	0.31745400
H	-3.32223000	-1.78146300	1.45391500
C	-5.82039300	-0.95119200	-0.71614800
H	-5.70651900	0.60925900	-2.20389600
H	-5.62558200	-2.42124400	0.85529600
H	-6.83434700	-1.23912500	-0.97349600
C	-1.02361100	1.22397900	-0.37303000
H	-1.34983800	2.24932500	-0.51656200
C	0.34919800	0.90162000	0.03473100
C	0.21463400	-0.15569800	1.12136800
C	0.78309700	-0.56795400	-0.19679900
H	0.07613700	-1.08875400	-0.84310400
C	1.31646800	2.03697700	0.13088800
F	1.54466900	2.60035200	-1.07359100
F	2.51067700	1.68902400	0.63309300
F	0.83656700	3.01982400	0.92218800
C	2.20738800	-0.98452200	-0.39506300
C	2.95080900	-0.48131300	-1.46792200
C	2.78217800	-1.94462100	0.44271200
C	4.25425100	-0.91969800	-1.68987700
H	2.50639000	0.25954200	-2.12795900
C	4.08700900	-2.38484200	0.21995800
H	2.20517200	-2.35312900	1.26799100
C	4.82688300	-1.87103000	-0.84412400
H	4.82311600	-0.51777500	-2.52291000
H	4.52327900	-3.13009000	0.87831000
H	5.84358000	-2.21135100	-1.01581500
C	0.92125600	-0.15556500	2.44267000
H	1.99340600	-0.00239400	2.31864400
H	0.51678500	0.63698000	3.07838900
H	0.76280100	-1.11849700	2.93711600

1-TS2'-diradical

Sum of electronic and thermal Free Energies= -1107.080682

C	0.53485400	-1.08464400	0.10836900
O	0.03156400	-0.88877000	1.36264000
C	1.90227200	-1.25719200	-0.07752700
C	2.43952300	-1.48016800	-1.38640700
C	2.81798000	-1.19113000	1.02249100
C	3.80273400	-1.62041800	-1.56823300
H	1.77100300	-1.54022700	-2.23991900
C	4.17509500	-1.33618800	0.81081500
H	2.43222100	-1.02722400	2.02364300
C	4.68793500	-1.55186300	-0.48042700
H	4.19103700	-1.78699400	-2.56911900
H	4.85450200	-1.28214900	1.65706200
H	5.75589700	-1.66573500	-0.63426900
C	-0.57057700	-1.01507900	-0.86431500
H	-0.49154100	-1.17618600	-1.92943100
C	-1.69558900	-0.58774700	-0.15917900
C	-1.31999900	-0.44447400	1.29107600
C	-1.47893100	0.94731500	0.79597700
H	-2.47538400	1.36400400	0.91435800
C	-3.08043200	-0.79151400	-0.67634900
F	-4.01571800	-0.07931400	-0.02184700
F	-3.45755200	-2.08432700	-0.57912800
F	-3.17354100	-0.46154300	-1.97735500
C	-0.40169000	1.86775100	0.40460500
C	-0.61451400	2.72983100	-0.68371800
C	0.82289800	1.93230500	1.09009200
C	0.37061900	3.63028000	-1.07926400
H	-1.55793000	2.68118700	-1.22235700

C	1.80566900	2.83494300	0.69144100
H	0.99963800	1.27961200	1.93887000
C	1.58542600	3.68462600	-0.39385200
H	0.19156700	4.28733400	-1.92509900
H	2.74648000	2.87417200	1.23241800
H	2.35504400	4.38548200	-0.70252500
C	-2.14446600	-0.98528700	2.42594500
H	-3.17191500	-0.62247900	2.35658800
H	-2.14505400	-2.07774200	2.40039200
H	-1.71961800	-0.64433600	3.37399100

6a-S0

Sum of electronic and thermal Free Energies= -1107.175655

C	-1.82641100	0.03387100	0.00908200
O	-1.15384900	-0.84829600	0.81851100
C	-3.25588100	-0.24175300	-0.18477800
C	-4.03087000	0.58714900	-1.00875200
C	-3.86011400	-1.32748200	0.46005300
C	-5.38778200	0.33602600	-1.17657700
H	-3.57081400	1.42815500	-1.51914400
C	-5.22129600	-1.57543400	0.28756100
H	-3.26375600	-1.97411000	1.09484000
C	-5.98847700	-0.74635500	-0.52849100
H	-5.97922700	0.98426400	-1.81595700
H	-5.68094900	-2.41958300	0.79255700
H	-7.04814800	-0.94121400	-0.66261300
C	-1.04596600	1.01972600	-0.46593600
H	-1.35422300	1.84550700	-1.09236700
C	0.34393100	0.77591200	0.00323900
C	0.21461100	-0.47290000	0.85992300
C	0.94927000	-0.55745700	-0.44650900

H	0.37808600	-0.99733300	-1.26309200
C	1.15210100	1.96694900	0.40355500
F	1.46538700	2.74346900	-0.65755800
F	2.30847500	1.65575200	1.01198600
F	0.46847500	2.75860300	1.25662600
C	2.41981000	-0.83848200	-0.51763900
C	3.26283200	-0.03742400	-1.29479500
C	2.95241100	-1.96373700	0.11983500
C	4.61622000	-0.34567400	-1.41991300
H	2.85649400	0.83404200	-1.80192800
C	4.30621100	-2.27488600	-0.00509000
H	2.30228100	-2.60046400	0.71429200
C	5.14212500	-1.46430600	-0.77270500
H	5.25967000	0.28822100	-2.02277800
H	4.70663200	-3.15057300	0.49710000
H	6.19692900	-1.70395600	-0.86807700
C	0.81763100	-0.69801200	2.21526700
H	1.88080300	-0.45702500	2.22275400
H	0.30603900	-0.07614300	2.95549000
H	0.69781500	-1.74821500	2.49737200

7. The structural determination of 2za and 2zd

(1) The structural determination of 2za

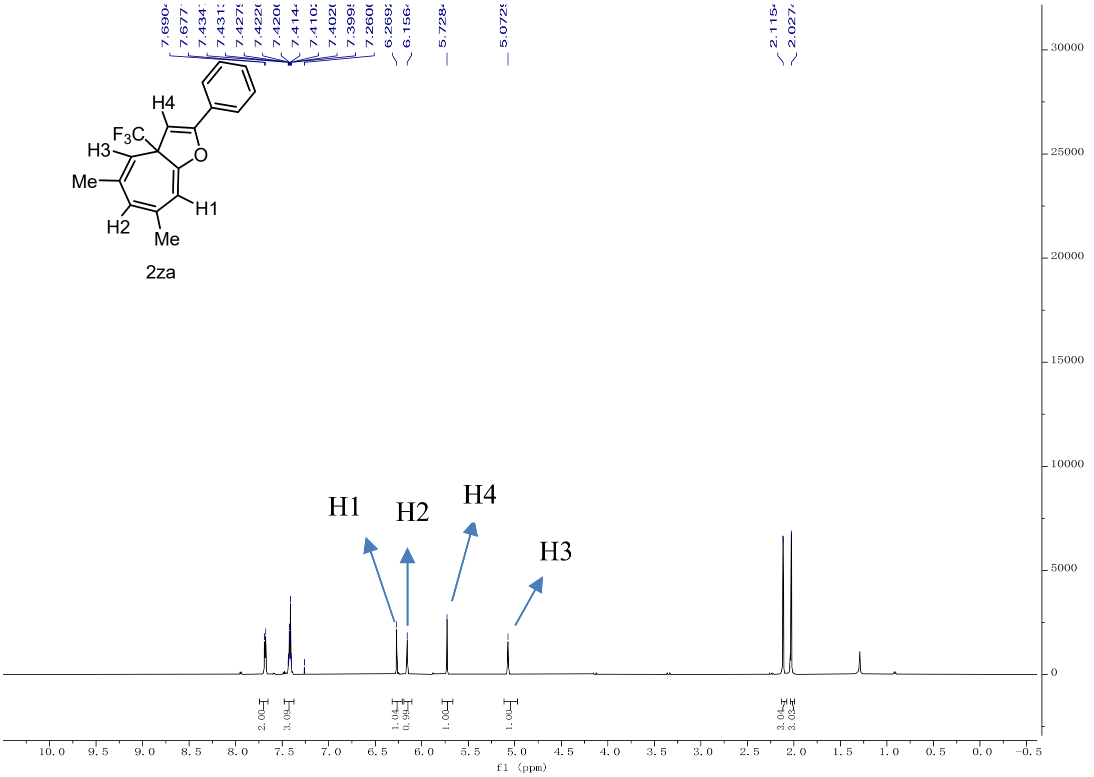

Fig. S9. The ^{1}H-NMR of compound **2a** after decoupling from F (400 MHz, $CDCl_3$).

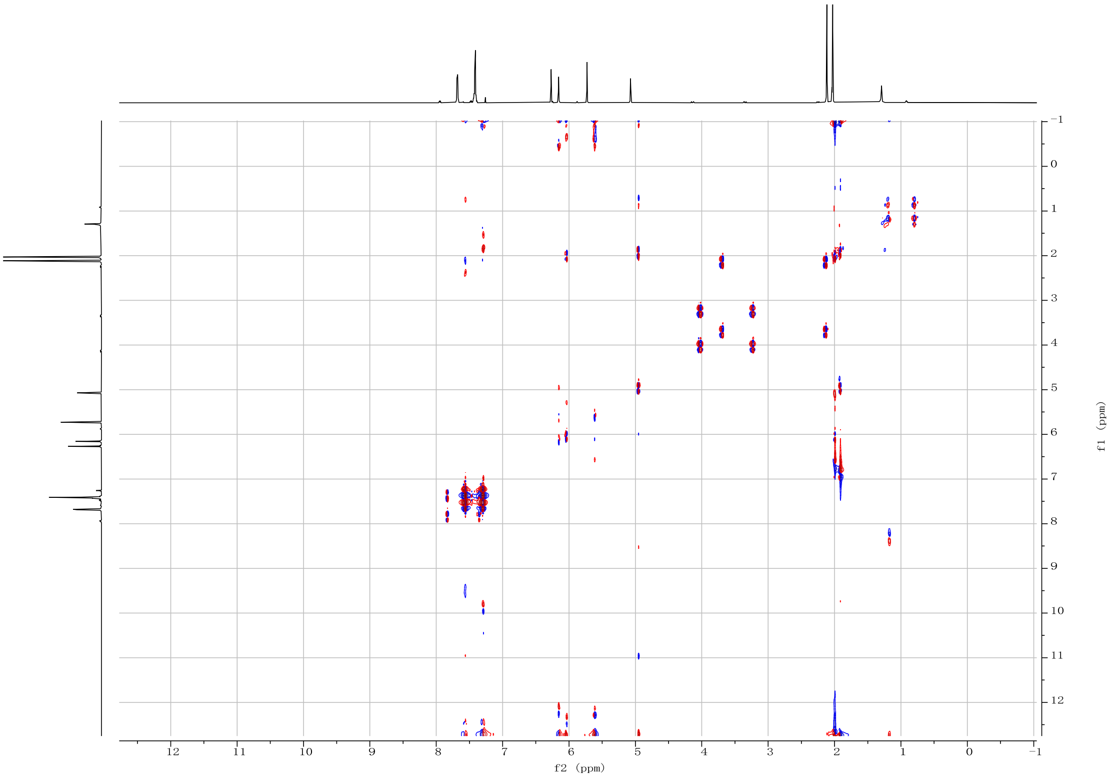

Fig. S10. ^{1}H-^{1}H COSY of **2za** (400 MHz, $CDCl_3$)

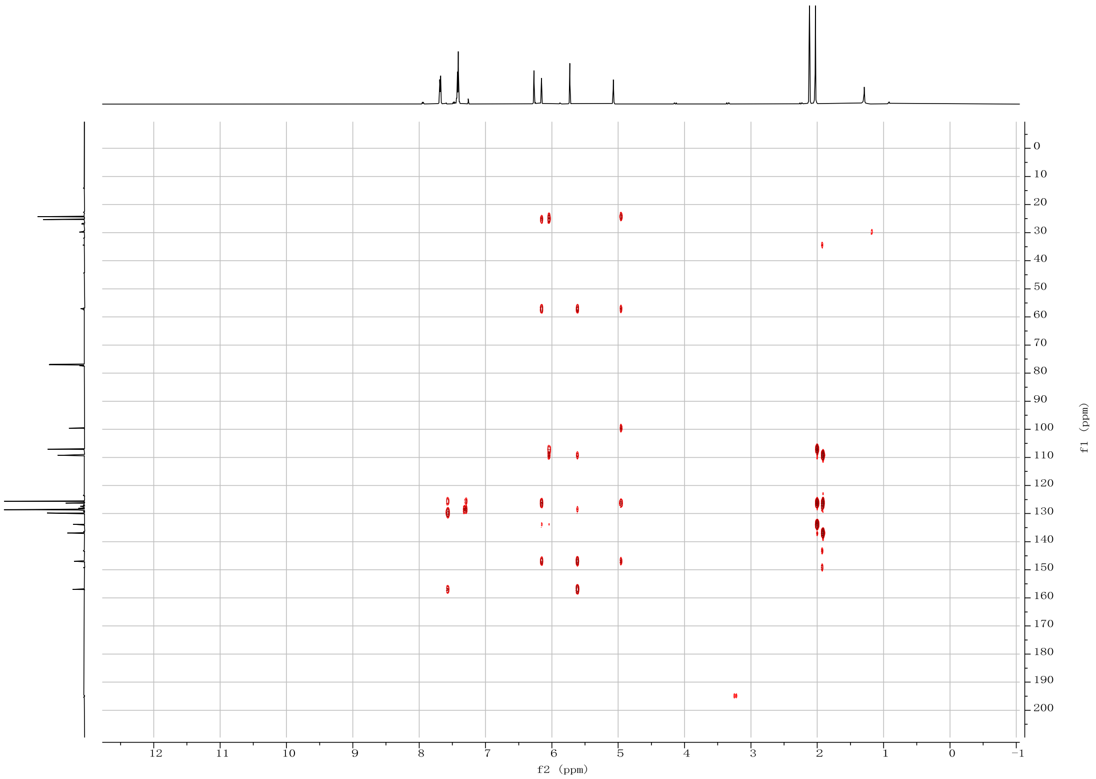

Fig. S11. ^{1}H-^{13}C HMBC of **2za** (400 MHz, $CDCl_3$)

(2) The structural determination of 2zd

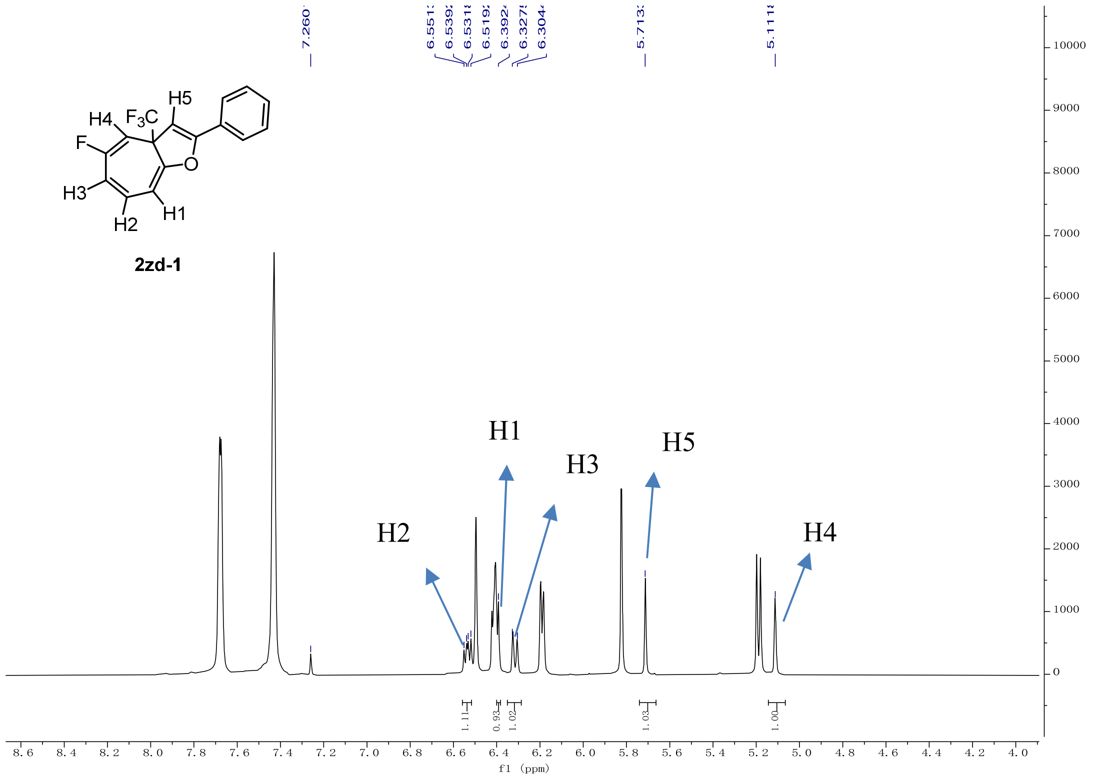

Fig. S12. The ^{1}H-NMR of compound **2zd-1** after decoupling from F (400 MHz, $CDCl_3$).

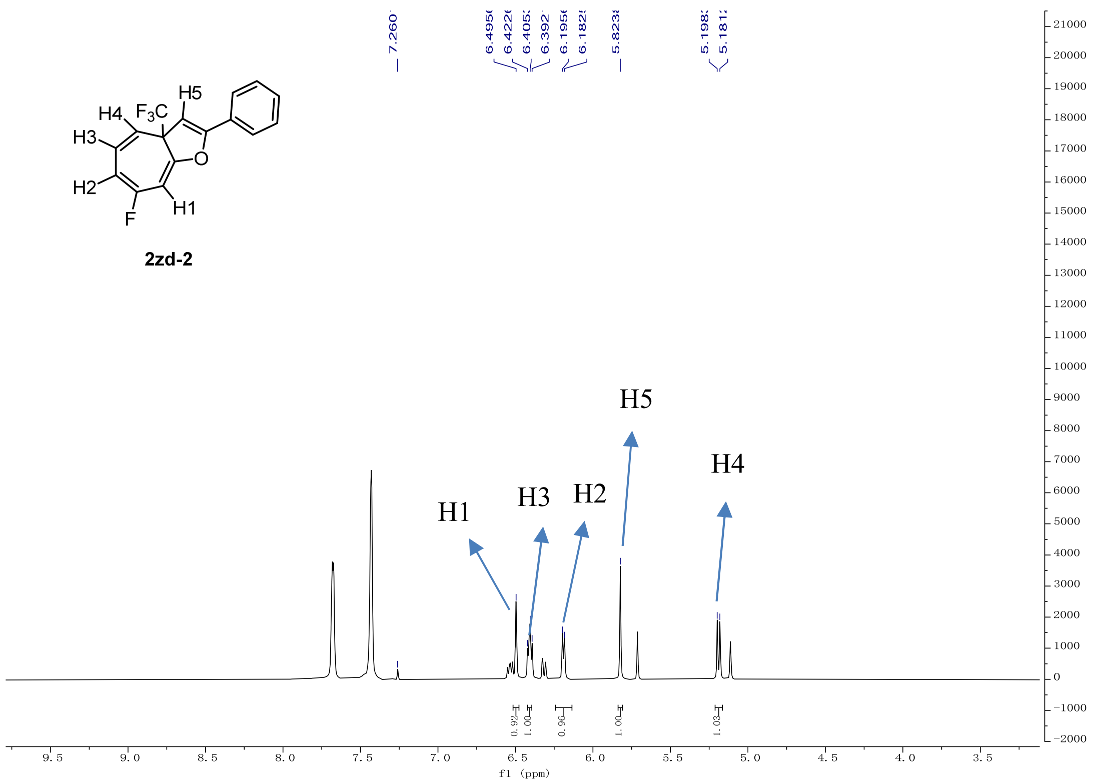

Fig. S13. The ^{1}H-NMR of compound **2zd-2** after decoupling from F (400 MHz, $CDCl_3$).

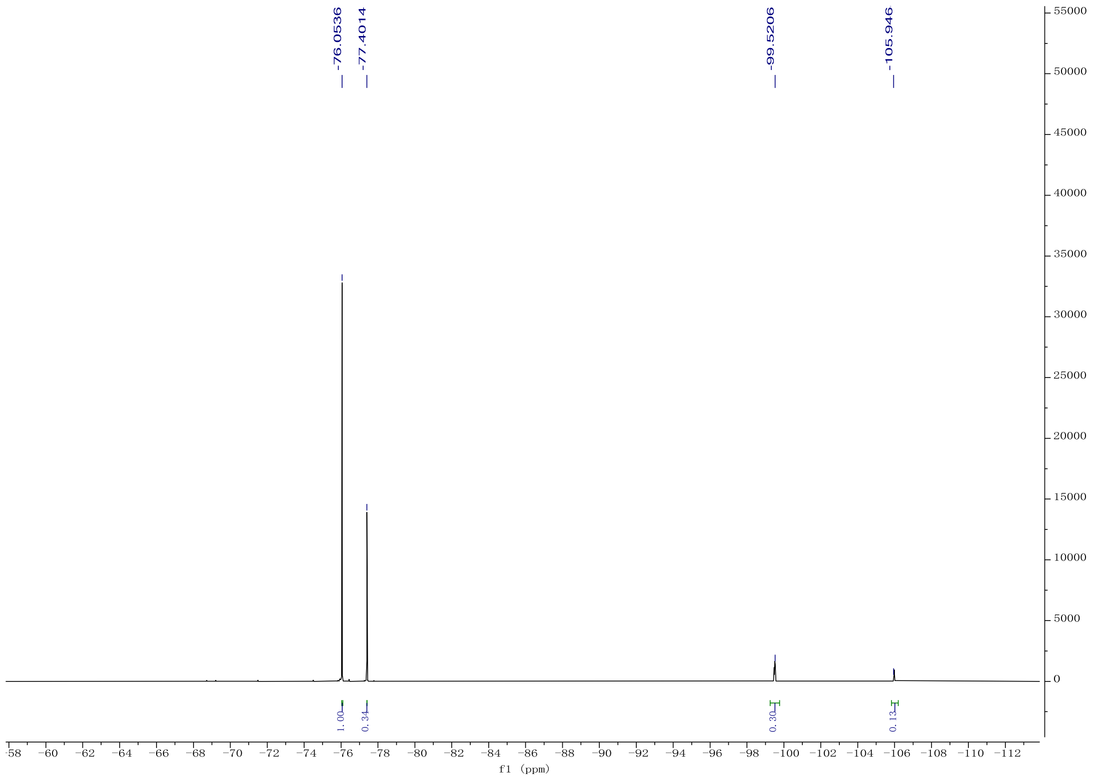

Fig. S14. ^{19}F NMR (565 MHz, CDCl3) of **2zd**.

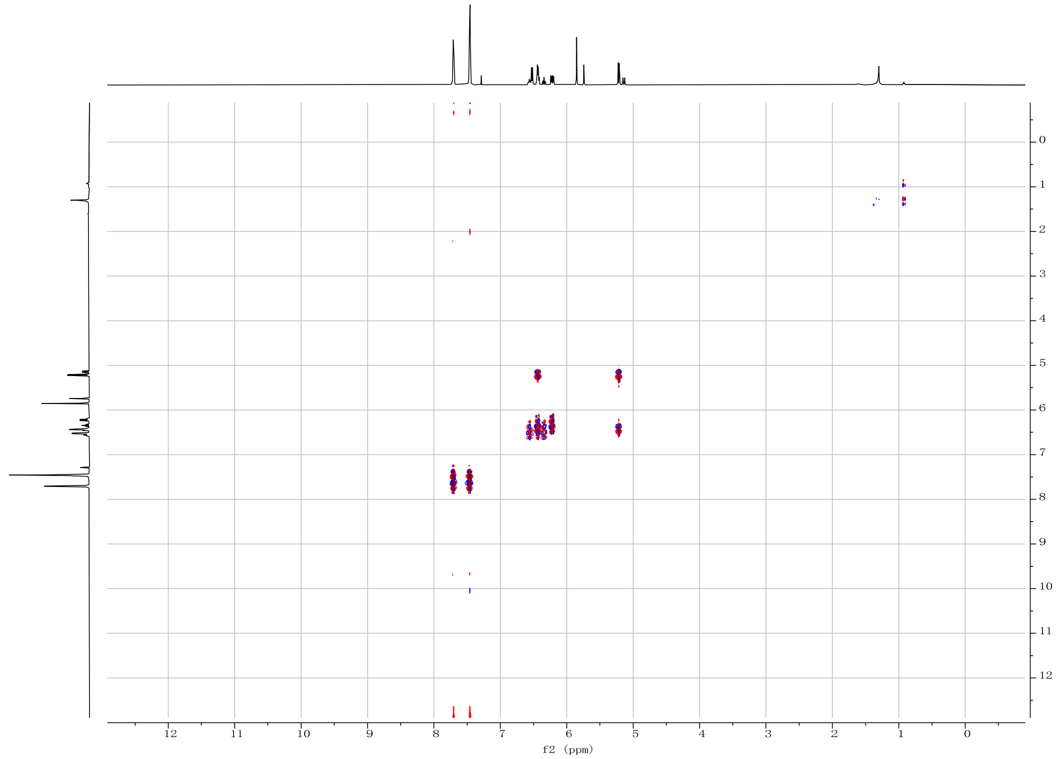

Fig. S15. ^{1}H-^{1}H COSY of **2zd** (400 MHz, $CDCl_3$)

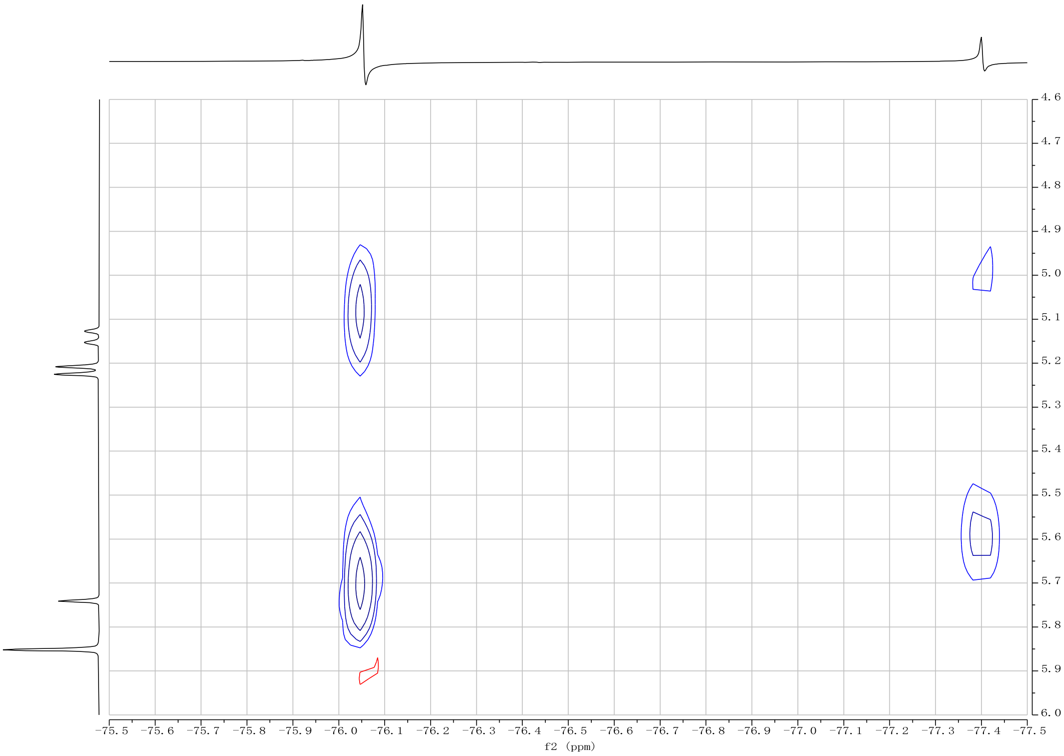

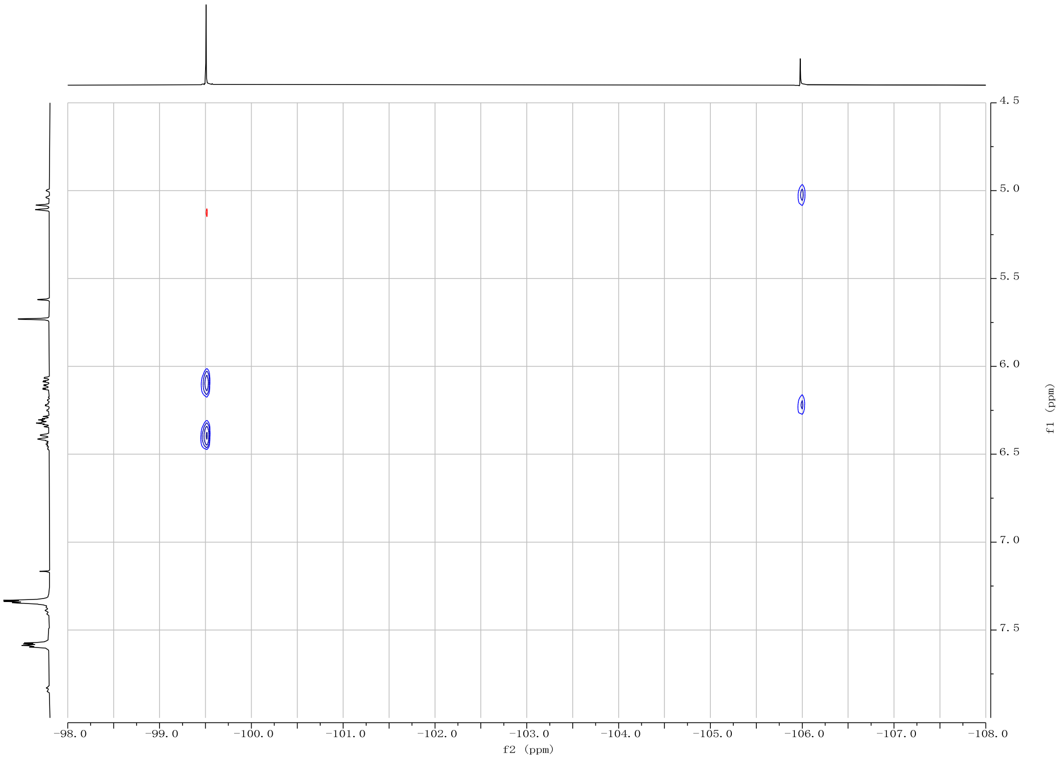

Fig. S16. ^{19}F-^{1}H HONESY of **2zd** (400 MHz, $CDCl_3$)

Note: According to the NMR data analysis, **2zd-1:2zd-2** = 1:3.

8. X-Ray crystallographic data of 2c, 2za and 6b

(1) X-ray crystallographic analysis of a single crystal of compound **2c** (CCDC2285183).

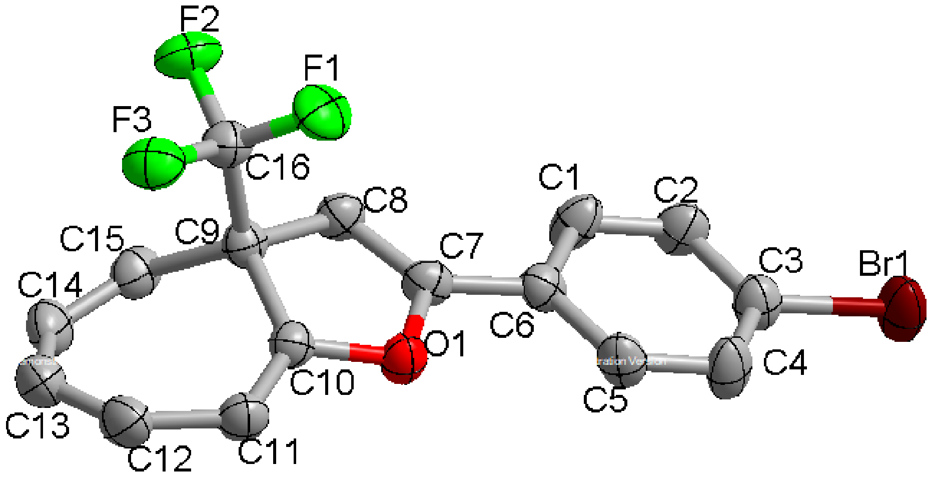

Fig. S17. Relative configuration of product **2c**

Displacement ellipsoids are drawn at the 30% probability level.

(Solvents: dichloromethane/petroleum ether = 1:10)

Table **S3** Crystal data and structure refinement for compound **2c** (**CCDC2285183**).

dentification code	
Empirical formula	$C_{16}H_{10}BrF_3O$
Formula weight	355.15
Temperature/K	293(2)
Crystal system	Monoclinic
Space group	$P2_1/c$
a/Å	16.291(2)
b/Å	6.2878(6)
c/Å	15.4937(18)
α/°	90
β/°	116.475(15)
γ/°	90
Volume/Å3	1420.7(3)
Z	4
ρcalcg/cm3	1.660
μ/mm-1	4.235
F(000)	704.0
Crystal size/mm3	0.339 × 0.137 × 0.078
Radiation	CuKα (λ = 1.54184)

2Θ range for data collection/°	11.428 to 134.124
Index ranges	-15 ⩽ h ⩽ 19, -7 ⩽ k ⩽ 5, -17 ⩽ l ⩽ 18
Reflections collected	5416
Independent reflections	2534 [R_{int} = 0.0355, R_{sigma} = 0.0445]
Data/restraints/parameters	2534/1/200
Goodness-of-fit on F2	1.021
Final R indexes [I>=2σ (I)]	R_1 = 0.0561, wR_2 = 0.1482
Final R indexes [all data]	R_1 = 0.0776, wR_2 = 0.1725
Largest diff. peak/hole / e Å-3	1.10/-0.35

Table **S4** Fractional Atomic Coordinates (×10^4) and Equivalent Isotropic Displacement Parameters ($Å_2$×10^3) for **2c**. U_{eq} is defined as 1/3 of of the trace of the orthogonalised UIJ tensor.

Atom	**x**	**y**	**Z**	**U(eq)**
Br1	9673(3)	11366(6)	11719(3)	97.2(7)
Br1A	9772(8)	11230(20)	11575(8)	97.2(7)
F1	5949(2)	1950(6)	7937(2)	88.8(9)
F2	4931.4(19)	3334(5)	6639(2)	83.1(9)
F3	5587(2)	454(5)	6583(2)	79.5(8)
O1	7973(2)	3621(5)	8211(2)	63.6(7)
C1	7690(6)	8720(20)	9247(11)	70(2)
C2	8178(7)	10020(20)	10016(11)	71(2)
C3	9031(7)	9418(15)	10689(6)	68(2)
C3A	9000(19)	9700(50)	10510(20)	68(2)
C4A	9483(13)	8170(40)	10276(16)	83(3)
C5A	9018(16)	6770(30)	9520(16)	71(2)
C6A	8070(16)	6890(50)	9000(20)	56.3(18)
C1A	7586(13)	8420(70)	9240(30)	70(2)
C2A	8051(19)	9820(70)	9990(30)	71(2)
C4	9393(5)	7501(15)	10609(6)	83(3)
C5	8899(5)	6188(13)	9831(6)	71(2)
C6	8036(6)	6779(17)	9129(7)	56.3(18)
C7	7519(3)	5442(7)	8274(3)	57.4(9)
C8	6674(3)	5582(7)	7551(3)	60.2(10)
C9	6475(3)	3664(7)	6914(3)	55.0(9)
C10	7405(3)	2575(7)	7363(3)	55.3(9)

C11	7722(3)	941(8)	7057(3)	67.3(11)
C12	7257(4)	-101(9)	6143(4)	77.8(13)
C13	6585(4)	726(10)	5332(4)	80.4(14)
C14	6210(4)	2832(10)	5207(3)	77.0(13)
C15	6185(3)	4171(8)	5861(3)	67.4(11)
C16	5740(3)	2341(7)	7020(3)	60.6(10)

Table **S5** Anisotropic Displacement Parameters (Å^2×10^3) for **2c**. The Anisotropic displacement factor exponent takes the form: $-2\pi^2[h^2a^{*2}U_{11}+2hka^*b^*U_{12}+\ldots]$.

**Atom**	**U_{11}**	**U_{22}**	**U_{33}**	**U_{23}**	**U_{13}**	**U_{12}**
Br1	95.5(10)	92.3(7)	79.9(11)	-32.7(7)	17.6(6)	22.1(6)
Br1A	95.5(10)	92.3(7)	79.9(11)	-32.7(7)	17.6(6)	22.1(6)
F1	95(2)	108(2)	75.8(17)	21.5(17)	49.5(15)	-1.9(18)
F2	57.1(14)	84(2)	110(2)	15.4(16)	39.1(14)	12.1(13)
F3	77.6(17)	65.1(16)	104(2)	-3.3(15)	47.8(15)	-8.2(14)
O1	57.9(15)	64.1(18)	58.6(15)	-2.7(13)	16.8(12)	12.8(13)
C1	55(3)	53(5)	81(3)	-3(3)	11(3)	5(3)
C2	68(4)	56(4)	81(3)	-2(3)	27(3)	12(3)
C3	74(3)	63(4)	58(4)	3(3)	21(3)	14(3)
C3A	74(3)	63(4)	58(4)	3(3)	21(3)	14(3)
C4A	77(4)	89(6)	55(4)	-2(3)	3(3)	32(4)
C5A	76(4)	69(4)	61(4)	-3(3)	24(3)	27(3)
C6A	62(2)	58(3)	51(3)	9(3)	27(2)	10(2)
C1A	55(3)	53(5)	81(3)	-3(3)	11(3)	5(3)
C2A	68(4)	56(4)	81(3)	-2(3)	27(3)	12(3)
C4	77(4)	89(6)	55(4)	-2(3)	3(3)	32(4)
C5	76(4)	69(4)	61(4)	-3(3)	24(3)	27(3)
C6	62(2)	58(3)	51(3)	9(3)	27(2)	10(2)
C7	59(2)	53(2)	61(2)	8.4(18)	27.0(18)	11.6(18)
C8	57(2)	59(2)	62(2)	6.8(19)	23.8(17)	12.1(19)
C9	57(2)	56(2)	49.0(19)	7.2(16)	21.7(16)	3.0(18)
C10	55(2)	59(2)	52(2)	4.5(17)	24.0(16)	4.3(18)
C11	58(2)	70(3)	69(3)	-1(2)	24.6(19)	11(2)
C12	75(3)	79(3)	83(3)	-19(3)	39(2)	0(3)
C13	75(3)	103(4)	67(3)	-14(3)	35(2)	-3(3)

C14	80(3)	98(4)	51(2)	6(2)	28(2)	-4(3)
C15	63(2)	74(3)	61(2)	18(2)	23.6(19)	2(2)
C16	59(2)	61(2)	65(2)	10.4(19)	30.6(19)	6.3(19)

Table **S6** Bond Lengths for **2c**.

**Atom**	**Atom**	**Length/Å**		**Atom**	**Atom**	**Length/Å**
Br1	C3	1.911(8)		C6A	C7	1.416(17)
Br1A	C3A	1.838(18)		C1A	C2A	1.3900
F1	C16	1.327(5)		C4	C5	1.385(10)
F2	C16	1.335(5)		C5	C6	1.390(10)
F3	C16	1.333(6)		C6	C7	1.475(9)
O1	C7	1.389(5)		C7	C8	1.336(6)
O1	C10	1.389(5)		C8	C9	1.499(6)
C1	C2	1.369(9)		C9	C10	1.519(6)
C1	C6	1.393(9)		C9	C15	1.517(6)
C2	C3	1.368(10)		C9	C16	1.526(6)
C3	C4	1.372(10)		C10	C11	1.330(6)
C3A	C4A	1.3900		C11	C12	1.432(7)
C3A	C2A	1.3900		C12	C13	1.350(8)
C4A	C5A	1.3900		C13	C14	1.435(8)
C5A	C6A	1.3900		C14	C15	1.331(7)
C6A	C1A	1.3900				

Table **S7** Bond Angles for **2c**.

**Atom**	**Atom**	**Atom**	**Angle/°**		**Atom**	**Atom**	**Atom**	**Angle/°**
C10	O1	C7	107.8(3)		C8	C7	C6A	132.5(13)
C2	C1	C6	121.7(6)		C8	C7	C6	132.7(5)
C3	C2	C1	119.7(6)		C7	C8	C9	109.8(4)
C2	C3	Br1	116.7(7)		C8	C9	C10	100.5(3)
C2	C3	C4	120.5(6)		C8	C9	C15	114.3(4)
C4	C3	Br1	122.8(6)		C8	C9	C16	108.5(4)
C4A	C3A	Br1A	110.9(16)		C10	C9	C16	112.0(3)
C4A	C3A	C2A	120.0		C15	C9	C10	110.9(4)
C2A	C3A	Br1A	129.0(16)		C15	C9	C16	110.3(4)

C3A	C4A	C5A	120.0		O1	C10	C9	109.1(3)
C4A	C5A	C6A	120.0		C11	C10	O1	120.2(4)
C5A	C6A	C7	125(2)		C11	C10	C9	130.7(4)
C1A	C6A	C5A	120.0		C10	C11	C12	126.0(4)
C1A	C6A	C7	115(2)		C13	C12	C11	126.6(5)
C6A	C1A	C2A	120.0		C12	C13	C14	126.9(5)
C1A	C2A	C3A	120.0		C15	C14	C13	129.5(5)
C3	C4	C5	119.8(6)		C14	C15	C9	125.8(5)
C4	C5	C6	120.9(6)		F1	C16	F2	107.0(4)
C1	C6	C7	121.0(7)		F1	C16	F3	106.2(4)
C5	C6	C1	117.4(6)		F1	C16	C9	112.2(4)
C5	C6	C7	121.6(8)		F2	C16	C9	111.4(4)
O1	C7	C6A	114.8(13)		F3	C16	F2	105.5(4)
O1	C7	C6	115.1(5)		F3	C16	C9	114.1(4)
C8	C7	O1	112.2(4)					

Table **S8** Torsion Angles for **2c**.

**A**	**B**	**C**	**D**	**Angle/°**		**A**	**B**	**C**	**D**	**Angle/°**
Br1	C3	C4	C5	-178.3(7)		C7	O1	C10	C11	170.5(4)
Br1A	C3A	C4A	C5A	177(2)		C7	C6A	C1A	C2A	175(3)
Br1A	C3A	C2A	C1A	-176(3)		C7	C8	C9	C10	-5.8(5)
O1	C7	C8	C9	1.8(5)		C7	C8	C9	C15	-124.6(4)
O1	C10	C11	C12	-171.5(5)		C7	C8	C9	C16	111.8(4)
C1	C2	C3	Br1	178.5(9)		C8	C9	C10	O1	7.9(4)
C1	C2	C3	C4	-1.2(13)		C8	C9	C10	C11	-169.6(5)
C1	C6	C7	O1	175.4(7)		C8	C9	C15	C14	160.7(5)
C1	C6	C7	C8	-7.6(12)		C8	C9	C16	F1	-51.1(5)
C2	C1	C6	C5	0.6(11)		C8	C9	C16	F2	68.8(4)
C2	C1	C6	C7	-177.2(10)		C8	C9	C16	F3	-171.9(3)
C2	C3	C4	C5	1.3(15)		C9	C10	C11	C12	5.9(9)
C3	C4	C5	C6	-0.4(15)		C10	O1	C7	C6A	-168.6(19)
C3A	C4A	C5A	C6A	0.0		C10	O1	C7	C6	-178.8(6)
C4A	C3A	C2A	C1A	0.0		C10	O1	C7	C8	3.6(5)
C4A	C5A	C6A	C1A	0.0		C10	C9	C15	C14	47.9(6)
C4A	C5A	C6A	C7	-174(3)		C10	C9	C16	F1	59.0(5)

C5A	C6A	C1A	C2A	0.0		C10	C9	C16	F2	178.8(4)
C5A	C6A	C7	O1	10(4)		C10	C9	C16	F3	-61.9(5)
C5A	C6A	C7	C8	-159.8(19)		C10	C11	C12	C13	22.8(9)
C6A	C1A	C2A	C3A	0.0		C11	C12	C13	C14	1.5(10)
C6A	C7	C8	C9	172(2)		C12	C13	C14	C15	-24.7(10)
C1A	C6A	C7	O1	-164.2(16)		C13	C14	C15	C9	-6.8(9)
C1A	C6A	C7	C8	26(3)		C15	C9	C10	O1	129.2(4)
C2A	C3A	C4A	C5A	0.0		C15	C9	C10	C11	-48.4(7)
C4	C5	C6	C1	-0.5(12)		C15	C9	C16	F1	-177.0(4)
C4	C5	C6	C7	177.3(7)		C15	C9	C16	F2	-57.1(5)
C5	C6	C7	O1	-2.3(12)		C15	C9	C16	F3	62.2(5)
C5	C6	C7	C8	174.7(8)		C16	C9	C10	O1	-107.1(4)
C6	C1	C2	C3	0.2(12)		C16	C9	C10	C11	75.3(6)
C6	C7	C8	C9	-175.2(7)		C16	C9	C15	C14	-76.8(6)
C7	O1	C10	C9	-7.4(5)						

Table **S9** Hydrogen Atom Coordinates (Å×10^4) and Isotropic Displacement Parameters (Å^2×10^3) for **2c**.

**Atom**	**x**	**y**	**z**	**U(eq)**
H1	7112	9153	8791	84
H2	7931	11315	10080	85
H4A	10118	8090	10624	100
H5A	9342	5748	9362	85
H1A	6952	8503	8889	84
H2A	7728	10845	10150	85
H4	9968	7084	11076	100
H5	9149	4894	9776	85
H8	6269	6701	7456	72
H11	8300	428	7474	81
H12	7435	-1488	6105	93
H13	6338	-164	4797	97
H14	5946	3337	4577	92
H15	5970	5538	5653	81

Table **S10** Atomic Occupancy for **2c**.

Atom	Occupancy		Atom	Occupancy		Atom	Occupancy
Br1	0.752(15)		Br1A	0.248(15)		C1	0.752(15)
H1	0.752(15)		C2	0.752(15)		H2	0.752(15)
C3	0.752(15)		C3A	0.248(15)		C4A	0.248(15)
H4A	0.248(15)		C5A	0.248(15)		H5A	0.248(15)
C6A	0.248(15)		C1A	0.248(15)		H1A	0.248(15)
C2A	0.248(15)		H2A	0.248(15)		C4	0.752(15)
H4	0.752(15)		C5	0.752(15)		H5	0.752(15)
C6	0.752(15)						

(2) X-ray crystallographic analysis of a single crystal of compound **2za** (CCDC 2348513).

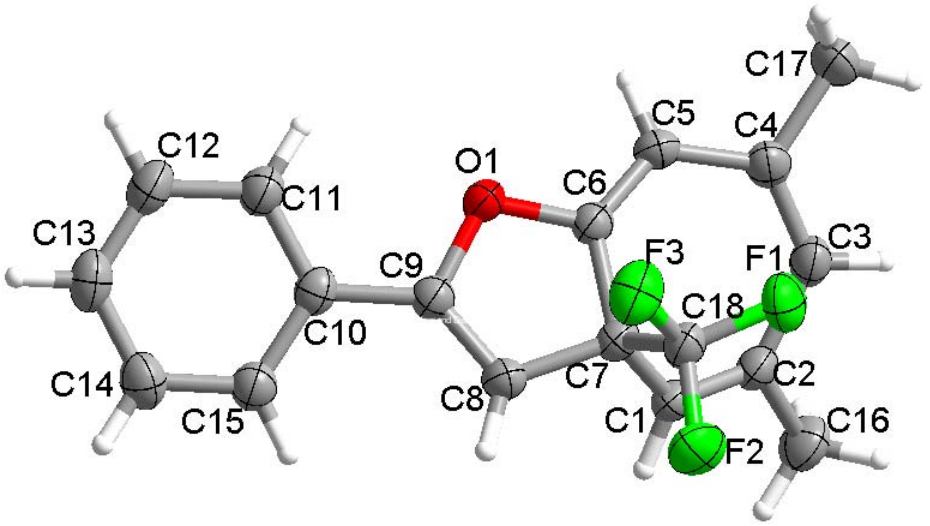

Fig. S18. Relative configuration of product **2za**

Displacement ellipsoids are drawn at the 30% probability level.

(Solvents: diethyl ether/petroleum ether = 1:10)

Table **S11** Crystal data and structure refinement for **2za (CCDC 2348513).**

Identification code	
Empirical formula	C18H15F3O
Formula weight	304.30
Temperature/K	293(2)
Crystal system	triclinic
Space group	P-1
a/Å	6.9554(4)

b/Å	13.0085(7)
c/Å	17.4007(13)
α/°	75.775(6)
β/°	80.036(6)
γ/°	89.708(5)
Volume/Å^3	1501.95(17)
Z	4
ρ_{calc}g/cm^3	1.346
μ/mm^{-1}	0.912
F(000)	632.0
Crystal size/mm^3	0.19 × 0.11 × 0.08
Radiation	CuKα (λ = 1.54184)
2Θ range for data collection/°	7.016 to 134.16
Index ranges	-8 ≤ h ≤ 7, -15 ≤ k ≤ 15, -20 ≤ l ≤ 20
Reflections collected	8516
Independent reflections	8516 [Rint = ?, Rsigma = 0.0416
Data/restraints/parameters	8516/0/402
Goodness-of-fit on F^2	1.003
Final R indexes [I>=2σ (I)]	R_1 = 0.0513, wR_2 = 0.1190
Final R indexes [all data]	R_1 = 0.0797, wR_2 = 0.1277
Largest diff. peak/hole / e Å^{-3}	0.24/-0.28

Table **S12** Fractional Atomic Coordinates (×104) and Equivalent Isotropic Displacement Parameters (Å2×103) for **2za**. Ueq is defined as 1/3 of of the trace of the orthogonalised UIJ tensor.

Atom	x	y	z	U(eq)
C1	806(5)	2112(3)	7811.9(19)	56.6(7)
C2	-547(5)	1872(3)	8477.7(19)	59.6(7)
C3	-1146(5)	811(3)	8978.3(19)	63.7(8)
C4	-1131(5)	-113(3)	8765(2)	61.2(8)
C5	-412(4)	-216(3)	7959.3(19)	56.1(7)
C6	922(4)	411(2)	7403.3(18)	50.9(7)
C7	2108(4)	1359(3)	7457.8(18)	52.8(7)
C8	2967(4)	1793(3)	6579.5(19)	55.9(7)

C9	2490(4)	1150(2)	6148.4(18)	52.8(7)
C10	2927(4)	1193(3)	5291.6(18)	54.5(7)
C11	2512(5)	326(3)	5003(2)	66.2(8)
C12	2951(5)	387(3)	4188(2)	75.3(10)
C13	3798(5)	1282(3)	3656(2)	73.0(10)
C14	4217(5)	2150(3)	3932(2)	73.5(10)
C15	3790(5)	2108(3)	4741(2)	64.5(8)
C16	-1666(6)	2756(4)	8762(3)	81.6(11)
C17	-2016(6)	-1111(4)	9365(2)	83.1(11)
C18	3772(5)	1037(3)	7922(2)	60.4(8)
F1	3210(3)	542.9(18)	8694.3(12)	76.0(6)
F2	4884(3)	1886(2)	7897.8(15)	88.6(7)
F3	4982(3)	377(2)	7606.0(14)	84.3(6)
O1	1313(3)	285.9(17)	6626.4(12)	58.6(5)
C1'	8343(4)	2887(3)	2178.7(18)	55.5(7)
C2'	7581(5)	3142(3)	1503.2(19)	60.4(8)
C3'	7448(5)	4195(3)	999.5(19)	61.7(8)
C4'	7281(5)	5125(3)	1207.0(18)	59.0(8)
C5'	7269(4)	5235(3)	2014.8(19)	56.8(7)
C6'	8088(4)	4596(2)	2581.5(17)	50.2(7)
C7'	9329(4)	3646(2)	2535.4(17)	50.3(7)
C8'	9402(4)	3210(3)	3418.7(18)	54.5(7)
C9'	8534(4)	3860(3)	3840.6(18)	53.2(7)
C10'	8185(4)	3816(3)	4701.7(18)	53.8(7)
C11'	7520(5)	4689(3)	4985(2)	66.0(8)
C12'	7218(5)	4635(3)	5801(2)	75.8(10)
C13'	7567(5)	3723(3)	6337(2)	73.5(10)
C14'	8215(5)	2847(3)	6066(2)	73.7(10)
C15'	8532(5)	2896(3)	5254(2)	67.2(9)
C16'	6726(6)	2259(3)	1225(3)	77.5(10)
C17'	7001(7)	6137(4)	592(2)	89.0(12)
C18'	11412(4)	3957(3)	2081(2)	59.1(8)
F1'	12327(3)	4599(2)	2405.4(14)	85.3(7)
F2'	12482(3)	3092(2)	2106.9(16)	91.0(7)
F3'	11545(3)	4448.9(19)	1308.9(12)	78.1(6)

O1'	7776(3)	4726.0(17)	3357.0(12)	57.7(5)

Table **S13** Anisotropic Displacement Parameters (Å2×103) for **2za**. The Anisotropic displacement factor exponent takes the form: -2π2[h2a*2U11+2hka*b*U12+…].

Atom	U11	U22	U33	U23	U13	U12
C1	74(2)	49.1(19)	51.5(17)	-18.1(13)	-16.3(13)	-2.5(13)
C2	73(2)	59(2)	53.4(18)	-24.9(15)	-14.4(13)	3.1(14)
C3	67(2)	77(3)	48.3(17)	-22.1(16)	-5.6(13)	-5.5(15)
C4	58.4(19)	69(2)	55.7(18)	-13.4(16)	-10.3(13)	-11.4(14)
C5	62(2)	49.9(19)	58.7(18)	-17.4(14)	-11.1(13)	-7.4(12)
C6	55.0(18)	51.0(19)	49.5(16)	-15.9(13)	-11.7(12)	2.1(12)
C7	58.8(19)	52(2)	52.4(18)	-19.9(14)	-13.1(12)	-0.6(12)
C8	60.4(19)	53.6(19)	54.3(17)	-15.8(14)	-8.7(12)	-6.4(13)
C9	51.6(18)	55.3(19)	52.5(17)	-15.8(14)	-8.3(12)	0.7(12)
C10	48.4(17)	66(2)	53.5(17)	-21.0(14)	-11.0(11)	5.0(12)
C11	72(2)	72(2)	58.0(19)	-24.0(16)	-9.2(14)	-0.8(15)
C12	82(3)	90(3)	65(2)	-40(2)	-14.0(17)	4.6(19)
C13	68(2)	102(3)	52.8(19)	-26.9(19)	-9.8(14)	12.6(18)
C14	68(2)	89(3)	57(2)	-11.8(19)	-2.9(14)	-1.5(17)
C15	66(2)	70(2)	58.1(19)	-19.0(16)	-10.1(14)	-1.8(15)
C16	91(3)	84(3)	76(3)	-39(2)	-5.8(17)	9.5(18)
C17	100(3)	79(3)	63(2)	-12.6(19)	-0.3(17)	-27.3(19)
C18	68(2)	66(2)	50.5(19)	-20.8(16)	-11.6(13)	-5.3(15)
F1	76.9(13)	95.9(16)	54.6(12)	-12.1(10)	-19.6(8)	3.0(9)
F2	86.1(15)	93.0(18)	95.8(16)	-26.4(13)	-36.2(12)	-21.7(11)
F3	75.1(14)	105.7(19)	83.1(15)	-37.6(13)	-24.1(10)	24.8(11)
O1	67.6(14)	59.5(14)	51.9(12)	-22.7(10)	-6.4(9)	-8.7(9)
C1'	66(2)	47.6(19)	53.1(18)	-19.4(13)	-2.5(12)	6.7(12)
C2'	67(2)	63(2)	54.0(18)	-24.2(15)	-3.5(13)	4.0(13)
C3'	73(2)	72(2)	45.2(16)	-21.2(15)	-13.4(13)	8.2(14)
C4'	67(2)	61(2)	49.2(17)	-13.3(14)	-10.7(12)	11.3(13)
C5'	65(2)	50.6(19)	55.9(18)	-15.0(14)	-10.4(12)	10.4(12)
C6'	50.8(17)	52.8(19)	48.2(16)	-17.9(13)	-4.5(11)	3.3(11)
C7'	52.4(18)	51.0(19)	47.7(17)	-15.9(13)	-4.5(11)	7.9(11)
C8'	60.4(19)	53.7(19)	51.9(17)	-18.0(14)	-10.2(12)	12.9(13)

C9'	50.5(18)	59(2)	52.0(17)	-17.1(14)	-10.3(12)	5.0(12)
C10'	45.8(17)	71(2)	48.5(16)	-21.7(14)	-8.7(11)	2.0(12)
C11'	72(2)	75(2)	55.9(19)	-23.9(16)	-12.3(14)	9.5(15)
C12'	81(3)	91(3)	62(2)	-36(2)	-5.9(16)	7.0(18)
C13'	72(2)	103(3)	48.1(18)	-27.1(19)	-5.9(14)	-5.1(18)
C14'	77(2)	87(3)	57(2)	-15.0(19)	-15.4(15)	4.9(17)
C15'	70(2)	77(3)	58(2)	-21.8(17)	-13.6(14)	8.6(16)
C16'	102(3)	73(3)	69(2)	-38.5(19)	-14.2(17)	0.4(18)
C17'	127(4)	75(3)	64(2)	-11(2)	-25(2)	32(2)
C18'	55.0(19)	67(2)	56.1(19)	-19.5(16)	-6.4(12)	11.3(14)
F1'	66.0(13)	110(2)	85.8(15)	-39.6(13)	-5.6(10)	-12.9(11)
F2'	69.8(14)	98.7(19)	99.4(17)	-26.8(14)	1.5(10)	31.1(11)
F3'	75.0(13)	99.2(17)	51.5(11)	-11.8(10)	2.3(8)	3.1(9)
O1'	67.6(13)	58.9(14)	52.3(12)	-23.7(10)	-12.1(9)	15.7(9)

Table **S14** Bond Lengths for **2za**.

Atom	Atom	Length/Å		Atom	Atom	Length/Å
C1	C2	1.332(5)		C1'	C2'	1.340(5)
C1	C7	1.501(5)		C1'	C7'	1.513(4)
C2	C3	1.458(5)		C2'	C3'	1.446(5)
C2	C16	1.520(5)		C2'	C16'	1.512(5)
C3	C4	1.342(5)		C3'	C4'	1.344(5)
C4	C5	1.443(5)		C4'	C5'	1.446(5)
C4	C17	1.509(5)		C4'	C17'	1.515(5)
C5	C6	1.331(4)		C5'	C6'	1.332(4)
C6	C7	1.518(4)		C6'	C7'	1.515(4)
C6	O1	1.382(4)		C6'	O1'	1.381(4)
C7	C8	1.508(4)		C7'	C8'	1.512(4)
C7	C18	1.523(4)		C7'	C18'	1.529(4)
C8	C9	1.330(4)		C8'	C9'	1.328(4)
C9	C10	1.456(4)		C9'	C10'	1.463(4)
C9	O1	1.393(4)		C9'	O1'	1.392(4)
C10	C11	1.392(5)		C10'	C11'	1.392(5)
C10	C15	1.394(5)		C10'	C15'	1.388(5)
C11	C12	1.381(5)		C11'	C12'	1.383(5)

C12	C13	1.360(6)		C12'	C13'	1.367(6)
C13	C14	1.381(6)		C13'	C14'	1.381(6)
C14	C15	1.376(5)		C14'	C15'	1.378(5)
C18	F1	1.330(4)		C18'	F1'	1.334(4)
C18	F2	1.340(4)		C18'	F2'	1.342(4)
C18	F3	1.346(4)		C18'	F3'	1.326(4)

Table **S15** Bond Angles for **2za.**

Atom	Atom	Atom	Angle/°		Atom	Atom	Atom	Angle/°
C2	C1	C7	127.1(3)		C2'	C1'	C7'	126.2(3)
C1	C2	C3	126.4(3)		C1'	C2'	C3'	126.9(3)
C1	C2	C16	119.5(3)		C1'	C2'	C16'	118.4(3)
C3	C2	C16	114.1(3)		C3'	C2'	C16'	114.8(3)
C4	C3	C2	128.9(3)		C4'	C3'	C2'	129.0(3)
C3	C4	C5	123.8(3)		C3'	C4'	C5'	123.9(3)
C3	C4	C17	120.3(3)		C3'	C4'	C17'	120.6(3)
C5	C4	C17	115.8(3)		C5'	C4'	C17'	115.5(3)
C6	C5	C4	126.9(3)		C6'	C5'	C4'	126.5(3)
C5	C6	C7	129.8(3)		C5'	C6'	C7'	129.7(3)
C5	C6	O1	120.5(3)		C5'	C6'	O1'	120.3(3)
O1	C6	C7	109.6(2)		O1'	C6'	C7'	109.9(2)
C1	C7	C6	110.6(2)		C1'	C7'	C6'	110.5(2)
C1	C7	C8	113.7(3)		C1'	C7'	C18'	111.4(3)
C1	C7	C18	111.2(3)		C6'	C7'	C18'	112.9(3)
C6	C7	C18	112.6(3)		C8'	C7'	C1'	113.2(3)
C8	C7	C6	99.9(2)		C8'	C7'	C6'	99.9(2)
C8	C7	C18	108.5(2)		C8'	C7'	C18'	108.5(2)
C9	C8	C7	110.2(3)		C9'	C8'	C7'	109.9(3)
C8	C9	C10	132.7(3)		C8'	C9'	C10'	132.5(3)
C8	C9	O1	111.8(3)		C8'	C9'	O1'	112.1(3)
O1	C9	C10	115.5(3)		O1'	C9'	C10'	115.4(3)
C11	C10	C9	121.2(3)		C11'	C10'	C9'	121.2(3)
C11	C10	C15	118.5(3)		C15'	C10'	C9'	120.1(3)
C15	C10	C9	120.3(3)		C15'	C10'	C11'	118.7(3)
C12	C11	C10	119.9(4)		C12'	C11'	C10'	120.3(4)

C13	C12	C11	121.1(4)		C13'	C12'	C11'	120.3(4)
C12	C13	C14	119.7(3)		C12'	C13'	C14'	120.1(3)
C15	C14	C13	120.3(4)		C15'	C14'	C13'	120.0(4)
C14	C15	C10	120.5(3)		C14'	C15'	C10'	120.6(4)
F1	C18	C7	114.7(3)		F1'	C18'	C7'	112.2(3)
F1	C18	F2	106.9(3)		F1'	C18'	F2'	106.6(3)
F1	C18	F3	105.6(3)		F2'	C18'	C7'	110.3(3)
F2	C18	C7	110.9(3)		F3'	C18'	C7'	114.8(3)
F2	C18	F3	105.9(3)		F3'	C18'	F1'	106.0(3)
F3	C18	C7	112.3(3)		F3'	C18'	F2'	106.4(3)
C6	O1	C9	108.0(2)		C6'	O1'	C9'	107.7(2)

Table **S16** Torsion Angles for **2za**.

A	B	C	D	Angle/˚		A	B	C	D	Angle/˚
C1	C2	C3	C4	-28.9(5)		C1'	C2'	C3'	C4'	-29.5(5)
C1	C7	C8	C9	-122.7(3)		C1'	C7'	C8'	C9'	-122.6(3)
C1	C7	C18	F1	60.6(4)		C1'	C7'	C18'	F1'	-177.9(3)
C1	C7	C18	F2	-60.6(4)		C1'	C7'	C18'	F2'	-59.2(4)
C1	C7	C18	F3	-178.8(3)		C1'	C7'	C18'	F3'	61.0(4)
C2	C1	C7	C6	48.8(4)		C2'	C1'	C7'	C6'	48.9(4)
C2	C1	C7	C8	160.2(3)		C2'	C1'	C7'	C8'	160.0(3)
C2	C1	C7	C18	-77.1(4)		C2'	C1'	C7'	C18'	-77.4(4)
C2	C3	C4	C5	2.4(6)		C2'	C3'	C4'	C5'	2.2(5)
C2	C3	C4	C17	-173.6(3)		C2'	C3'	C4'	C17'	-175.1(3)
C3	C4	C5	C6	26.3(5)		C3'	C4'	C5'	C6'	26.2(5)
C4	C5	C6	C7	2.9(6)		C4'	C5'	C6'	C7'	4.2(5)
C4	C5	C6	O1	-172.8(3)		C4'	C5'	C6'	O1'	-172.6(3)
C5	C6	C7	C1	-49.0(4)		C5'	C6'	C7'	C1'	-50.5(4)
C5	C6	C7	C8	-169.0(3)		C5'	C6'	C7'	C8'	-169.9(3)
C5	C6	C7	C18	76.0(4)		C5'	C6'	C7'	C18'	75.1(4)
C5	C6	O1	C9	169.7(3)		C5'	C6'	O1'	C9'	170.7(3)
C6	C7	C8	C9	-4.9(3)		C6'	C7'	C8'	C9'	-5.1(3)
C6	C7	C18	F1	-64.1(4)		C6'	C7'	C18'	F1'	57.1(4)
C6	C7	C18	F2	174.7(3)		C6'	C7'	C18'	F2'	175.7(3)
C6	C7	C18	F3	56.5(4)		C6'	C7'	C18'	F3'	-64.1(4)

C7	C1	C2	C3	-4.4(5)		C7'	C1'	C2'	C3'	-3.6(5)
C7	C1	C2	C16	177.4(3)		C7'	C1'	C2'	C16'	177.5(3)
C7	C6	O1	C9	-6.8(3)		C7'	C6'	O1'	C9'	-6.7(3)
C7	C8	C9	C10	180.0(3)		C7'	C8'	C9'	C10'	179.4(3)
C7	C8	C9	O1	1.3(4)		C7'	C8'	C9'	O1'	1.6(4)
C8	C7	C18	F1	-173.7(3)		C8'	C7'	C18'	F1'	-52.6(4)
C8	C7	C18	F2	65.1(4)		C8'	C7'	C18'	F2'	66.1(4)
C8	C7	C18	F3	-53.1(4)		C8'	C7'	C18'	F3'	-173.8(3)
C8	C9	C10	C11	169.5(3)		C8'	C9'	C10'	C11'	169.1(3)
C8	C9	C10	C15	-10.1(5)		C8'	C9'	C10'	C15'	-10.3(5)
C8	C9	O1	C6	3.5(3)		C8'	C9'	O1'	C6'	3.2(3)
C9	C10	C11	C12	-179.7(3)		C9'	C10'	C11'	C12'	-179.3(3)
C9	C10	C15	C14	179.6(3)		C9'	C10'	C15'	C14'	179.8(3)
C10	C9	O1	C6	-175.5(2)		C10'	C9'	O1'	C6'	-175.0(2)
C10	C11	C12	C13	0.3(6)		C10'	C11'	C12'	C13'	-0.1(6)
C11	C10	C15	C14	0.1(5)		C11'	C10'	C15'	C14'	0.3(5)
C11	C12	C13	C14	-0.4(6)		C11'	C12'	C13'	C14'	-0.3(6)
C12	C13	C14	C15	0.3(5)		C12'	C13'	C14'	C15'	0.7(5)
C13	C14	C15	C10	-0.2(5)		C13'	C14'	C15'	C10'	-0.7(5)
C15	C10	C11	C12	-0.1(5)		C15'	C10'	C11'	C12'	0.1(5)
C16	C2	C3	C4	149.4(4)		C16'	C2'	C3'	C4'	149.5(3)
C17	C4	C5	C6	-157.6(3)		C17'	C4'	C5'	C6'	-156.3(3)
C18	C7	C8	C9	113.1(3)		C18'	C7'	C8'	C9'	113.1(3)
O1	C6	C7	C1	127.0(3)		O1'	C6'	C7'	C1'	126.6(3)
O1	C6	C7	C8	7.0(3)		O1'	C6'	C7'	C8'	7.1(3)
O1	C6	C7	C18	-107.9(3)		O1'	C6'	C7'	C18'	-107.9(3)
O1	C9	C10	C11	-11.9(4)		O1'	C9'	C10'	C11'	-13.1(4)
O1	C9	C10	C15	168.6(3)		O1'	C9'	C10'	C15'	167.5(3)

Table **S17** Hydrogen Atom Coordinates (Å×104) and Isotropic Displacement Parameters (Å2×103) for **2za**.

Atom	x	y	z	U(eq)
H1	962	2824	7536	68
H3	-1601	769	9521	76
H5	-936	-782	7813	67

H8	3722	2419	6370	67
H11	1939	-294	5360	79
H12	2663	-194	4000	90
H13	4093	1310	3109	88
H14	4789	2765	3569	88
H15	4080	2695	4922	77
H16A	-3038	2639	8783	122
H16B	-1236	3426	8393	122
H16C	-1432	2762	9288	122
H17A	-3316	-1238	9284	125
H17B	-2063	-1031	9902	125
H17C	-1232	-1701	9292	125
H1'	8256	2174	2455	67
H3'	7483	4235	456	74
H5'	6625	5810	2152	68
H8'	9970	2580	3635	65
H11'	7277	5312	4624	79
H12'	6776	5222	5985	91
H13'	7367	3692	6885	88
H14'	8437	2225	6432	88
H15'	8983	2307	5074	81
H16D	6824	1593	1602	116
H16E	7434	2239	704	116
H16F	5378	2384	1192	116
H17D	5847	6468	790	133
H17E	6862	5972	96	133
H17F	8115	6612	501	133

(3) X-ray crystallographic analysis of a single crystal of compound **6b** (CCDC 2286090).

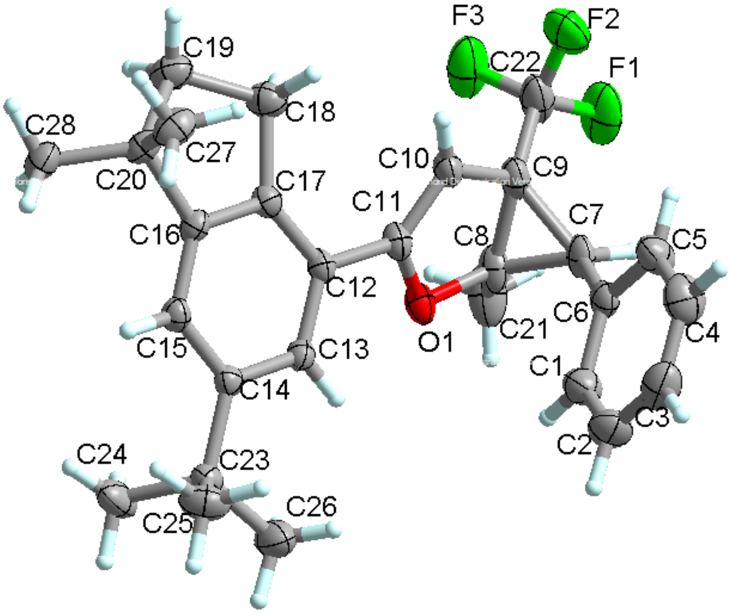

Fig. S18. Relative configuration of product **6b**

Displacement ellipsoids are drawn at the 30% probability level.

(Solvents: dichloromethane/petroleum ether = 1:10)

Table **S18** Crystal data and structure refinement for **6b** (**CCDC 2286090**).

Identification code	
Empirical formula	C28H31F3O
Formula weight	440.53
Temperature/K	293(2)
Crystal system	triclinic
Space group	P-1
a/Å	9.1987(6)
b/Å	11.0072(6)
c/Å	12.2093(6)
α/°	90.074(4)
β/°	92.871(4)
γ/°	96.049(5)
Volume/Å^3	1227.76(12)
Z	2
ρ_{calc}g/cm^3	1.192
μ/mm^{-1}	0.706
F(000)	468.0
Crystal size/mm^3	0.16 × 0.11 × 0.1

Radiation	CuKα (λ = 1.54184)
2Θ range for data collection/°	7.25 to 134.138
Index ranges	-10 ≤ h ≤ 10, -13 ≤ k ≤ 13, -14 ≤ l ≤ 9
Reflections collected	8391
Independent reflections	4357 [R_{int} = 0.0295, R_{sigma} = 0.0382]
Data/restraints/parameters	4357/0/295
Goodness-of-fit on F^2	1.036
Final R indexes [I>=2σ (I)]	R_1 = 0.0731, wR_2 = 0.2102
Final R indexes [all data]	R_1 = 0.0858, wR_2 = 0.2280
Largest diff. peak/hole / e $Å^{-3}$	0.67/-0.26

Table **S19** Fractional Atomic Coordinates (×10^4) and Equivalent Isotropic Displacement Parameters ($Å_2$×10^3) for **6b**. U_{eq} is defined as 1/3 of of the trace of the orthogonalised U_{IJ} tensor.

Atom	x	y	z	U(eq)
F1	6396(4)	3806(2)	4144.7(19)	129.6(11)
F2	8724(4)	3850(3)	4284.1(19)	125.8(10)
F3	7646(4)	5040(2)	5265(2)	134.7(12)
O1	6664.5(19)	2530.4(19)	7525.7(14)	61.3(5)
C1	6290(4)	-166(3)	6657(3)	75.4(8)
C2	6628(5)	-1337(3)	6842(3)	88.3(10)
C3	7577(5)	-1832(3)	6208(3)	88.9(11)
C4	8214(5)	-1150(4)	5393(3)	94.7(11)
C5	7894(4)	39(3)	5207(3)	77.8(9)
C6	6905(3)	546(2)	5839.0(19)	55.2(6)
C7	6452(3)	1763(3)	5589(2)	62.8(7)
C8	6122(3)	2675(3)	6426(2)	66.6(8)
C9	7452(4)	2964(3)	5743(2)	64.2(7)
C10	8694(3)	2954(3)	6563.8(19)	58.0(6)
C11	8169(3)	2698(2)	7545.5(19)	51.3(6)
C12	8916(2)	2587(2)	8631.2(17)	45.7(5)
C13	8238(2)	1878(2)	9434.9(18)	44.9(5)
C14	8897(3)	1763(2)	10476.4(17)	45.2(5)
C15	10281(3)	2389(2)	10704.2(18)	48.1(5)
C16	10960(2)	3115(2)	9918.0(19)	46.3(5)
C17	10288(2)	3221(2)	8882.4(18)	46.4(5)

C18	11262(3)	4127(3)	8230(3)	72.1(8)
C19	12345(4)	4794(3)	9117(3)	73.9(8)
C20	12447(3)	3863(2)	10042(2)	55.8(6)
C21	4713(5)	3293(4)	6389(3)	107.7(16)
C22	7547(6)	3909(3)	4873(3)	91.6(12)
C23	8105(3)	993(2)	11354.0(19)	51.4(6)
C24	7786(5)	1836(3)	12285(3)	89.6(11)
C25	9029(4)	28(3)	11782(3)	84.5(10)
C26	6630(4)	368(4)	10899(3)	91.2(11)
C27	13648(3)	3032(3)	9853(3)	74.8(8)
C28	12730(3)	4471(3)	11171(3)	70.9(8)

Table **S20** Anisotropic Displacement Parameters ($Å^2 \times 10^3$) for **6b**. The Anisotropic displacement factor exponent takes the form: $-2\pi^2[h^2a^{*2}U_{11}+2hka^*b^*U_{12}+\ldots]$.

Atom	U11	U22	U33	U23	U13	U12
F1	202(3)	101.0(16)	80.9(14)	15.2(12)	-66.3(17)	27.7(17)
F2	189(3)	115.4(19)	68.6(13)	20.9(12)	10.4(16)	-4.6(18)
F3	242(4)	64.7(13)	94.7(16)	-1.5(11)	-39.8(19)	25.8(16)
O1	54.8(10)	85.1(13)	45.1(9)	-10.0(8)	-10.0(7)	19.1(9)
C1	76.7(19)	85(2)	64.3(17)	4.8(15)	9.8(14)	4.5(16)
C2	108(3)	77(2)	77(2)	22.4(17)	-0.6(19)	-1.2(19)
C3	109(3)	66.3(19)	89(2)	2.7(18)	-28(2)	13.4(18)
C4	103(3)	94(3)	93(3)	-14(2)	7(2)	34(2)
C5	98(2)	78(2)	60.1(16)	1.9(14)	17.4(16)	16.7(17)
C6	61.0(14)	61.9(15)	41.1(11)	-3.9(10)	-10.2(10)	5.0(11)
C7	74.7(17)	69.9(17)	42.8(12)	-5.6(11)	-18.5(11)	13.3(13)
C8	71.0(17)	76.9(18)	53.6(14)	-12.1(13)	-21.9(12)	27.4(14)
C9	90(2)	60.3(15)	41.9(12)	-3.6(11)	-11.3(12)	14.0(13)
C10	67.4(16)	65.6(16)	40.1(12)	-5.8(11)	-3.0(11)	5.4(12)
C11	51.9(13)	60.4(14)	42.8(12)	-7.5(10)	-4.4(10)	14.2(10)
C12	46.9(12)	53.0(12)	38.1(11)	-4.2(9)	-2.9(9)	11.2(9)
C13	40.6(11)	49.9(12)	43.3(11)	-6.9(9)	-1.0(8)	2.5(9)
C14	50.5(12)	44.1(11)	41.3(11)	-4.2(9)	3.7(9)	5.5(9)
C15	53.3(13)	49.3(12)	41.1(11)	-0.6(9)	-7.1(9)	6.7(10)
C16	44.2(11)	43.0(11)	51.1(12)	-2.7(9)	-5.1(9)	5.1(9)

C17	46.2(12)	48.8(12)	44.9(12)	1.3(9)	-0.3(9)	8.8(9)
C18	70.6(18)	76.3(19)	69.0(18)	14.7(14)	2.8(14)	6.5(14)
C19	68.4(17)	67.1(18)	84(2)	17.0(15)	-3.3(15)	-1.4(14)
C20	46.5(13)	50.1(13)	68.6(15)	-0.1(11)	-6.0(11)	-0.7(10)
C21	109(3)	127(3)	94(3)	-33(2)	-40(2)	67(3)
C22	152(4)	66(2)	54.4(17)	-2.6(14)	-15(2)	12(2)
C23	57.6(14)	53.2(13)	43.2(12)	1.2(10)	6.4(10)	2.2(10)
C24	126(3)	80(2)	66.7(19)	-2.5(16)	41(2)	10(2)
C25	85(2)	78(2)	94(2)	33.9(18)	17.1(18)	17.3(17)
C26	83(2)	107(3)	77(2)	8.2(19)	1.1(17)	-20(2)
C27	50.2(15)	73.1(19)	100(2)	-2.2(16)	-1.1(14)	5.9(13)
C28	69.3(17)	60.4(16)	78.3(19)	-8.5(14)	-15.3(14)	-6.5(13)

Table **S21** Bond Lengths for **6b**.

Atom	Atom	Length/Å		Atom	Atom	Length/Å
F1	C22	1.343(5)		C10	C11	1.334(4)
F2	C22	1.337(5)		C11	C12	1.473(3)
F3	C22	1.325(4)		C12	C13	1.387(3)
O1	C8	1.423(3)		C12	C17	1.396(3)
O1	C11	1.376(3)		C13	C14	1.393(3)
C1	C2	1.375(5)		C14	C15	1.397(3)
C1	C6	1.377(4)		C14	C23	1.532(3)
C2	C3	1.351(6)		C15	C16	1.381(3)
C3	C4	1.365(6)		C16	C17	1.390(3)
C4	C5	1.388(5)		C16	C20	1.521(3)
C5	C6	1.382(4)		C17	C18	1.522(4)
C6	C7	1.472(4)		C18	C19	1.563(5)
C7	C8	1.495(4)		C19	C20	1.532(4)
C7	C9	1.534(4)		C20	C27	1.532(4)
C8	C9	1.521(5)		C20	C28	1.530(4)
C8	C21	1.525(4)		C23	C24	1.526(4)
C9	C10	1.483(4)		C23	C25	1.507(4)
C9	C22	1.487(5)		C23	C26	1.533(4)

Table **S22** Bond Angles for **6b**.

Atom	Atom	Atom	Angle/°		Atom	Atom	Atom	Angle/°
C11	O1	C8	108.2(2)		C12	C13	C14	121.9(2)
C2	C1	C6	121.9(3)		C13	C14	C15	118.2(2)
C3	C2	C1	120.3(3)		C13	C14	C23	120.7(2)
C2	C3	C4	119.4(3)		C15	C14	C23	121.1(2)
C3	C4	C5	120.8(4)		C16	C15	C14	120.5(2)
C6	C5	C4	120.2(3)		C15	C16	C17	120.6(2)
C1	C6	C5	117.3(3)		C15	C16	C20	126.9(2)
C1	C6	C7	121.6(3)		C17	C16	C20	112.6(2)
C5	C6	C7	120.9(3)		C12	C17	C18	131.9(2)
C6	C7	C8	124.9(2)		C16	C17	C12	119.9(2)
C6	C7	C9	124.4(2)		C16	C17	C18	108.1(2)
C8	C7	C9	60.2(2)		C17	C18	C19	104.1(2)
O1	C8	C7	117.9(2)		C20	C19	C18	104.6(2)
O1	C8	C9	106.6(2)		C16	C20	C19	102.0(2)
O1	C8	C21	111.2(3)		C16	C20	C27	109.2(2)
C7	C8	C9	61.13(19)		C16	C20	C28	112.7(2)
C7	C8	C21	123.3(2)		C19	C20	C27	111.2(3)
C9	C8	C21	127.9(3)		C28	C20	C19	112.5(2)
C8	C9	C7	58.62(19)		C28	C20	C27	109.1(2)
C10	C9	C7	116.3(2)		F1	C22	C9	113.6(4)
C10	C9	C8	103.0(2)		F2	C22	F1	105.6(3)
C10	C9	C22	118.3(3)		F2	C22	C9	111.7(3)
C22	C9	C7	121.5(2)		F3	C22	F1	106.0(3)
C22	C9	C8	123.8(3)		F3	C22	F2	106.0(4)
C11	C10	C9	108.9(3)		F3	C22	C9	113.2(3)
O1	C11	C12	115.5(2)		C14	C23	C26	111.3(2)
C10	C11	O1	113.2(2)		C24	C23	C14	108.6(2)
C10	C11	C12	131.3(2)		C24	C23	C26	107.1(3)
C13	C12	C11	120.1(2)		C25	C23	C14	110.6(2)
C13	C12	C17	118.9(2)		C25	C23	C24	110.3(3)
C17	C12	C11	121.0(2)		C25	C23	C26	108.8(3)

Table **S22** Torsion Angles for **6b**.

A	B	C	D	Angle/°		A	B	C	D	Angle/°

O1	C8	C9	C7	113.2(2)		C10	C11	C12	C13	-155.0(3)
O1	C8	C9	C10	0.1(3)		C10	C11	C12	C17	27.1(4)
O1	C8	C9	C22	-137.7(3)		C11	O1	C8	C7	65.2(3)
O1	C11	C12	C13	26.2(3)		C11	O1	C8	C9	-0.4(3)
O1	C11	C12	C17	-151.7(2)		C11	O1	C8	C21	-143.9(3)
C1	C2	C3	C4	1.1(6)		C11	C12	C13	C14	-179.0(2)
C1	C6	C7	C8	39.8(4)		C11	C12	C17	C16	179.0(2)
C1	C6	C7	C9	115.0(3)		C11	C12	C17	C18	2.6(4)
C2	C1	C6	C5	-0.5(5)		C12	C13	C14	C15	0.1(3)
C2	C1	C6	C7	175.2(3)		C12	C13	C14	C23	178.8(2)
C2	C3	C4	C5	-0.3(6)		C12	C17	C18	C19	161.4(3)
C3	C4	C5	C6	-0.9(6)		C13	C12	C17	C16	1.2(3)
C4	C5	C6	C1	1.2(5)		C13	C12	C17	C18	-175.3(3)
C4	C5	C6	C7	-174.4(3)		C13	C14	C15	C16	1.0(3)
C5	C6	C7	C8	-144.7(3)		C13	C14	C23	C24	-114.7(3)
C5	C6	C7	C9	-69.5(4)		C13	C14	C23	C25	124.1(3)
C6	C1	C2	C3	-0.7(6)		C13	C14	C23	C26	3.0(4)
C6	C7	C8	O1	18.9(5)		C14	C15	C16	C17	-0.9(3)
C6	C7	C8	C9	113.3(3)		C14	C15	C16	C20	179.2(2)
C6	C7	C8	C21	-128.2(4)		C15	C14	C23	C24	63.9(3)
C6	C7	C9	C8	-114.1(3)		C15	C14	C23	C25	-57.2(3)
C6	C7	C9	C10	-24.5(4)		C15	C14	C23	C26	-178.4(3)
C6	C7	C9	C22	133.0(3)		C15	C16	C17	C12	-0.2(3)
C7	C8	C9	C10	-113.1(2)		C15	C16	C17	C18	177.1(2)
C7	C8	C9	C22	109.1(3)		C15	C16	C20	C19	-159.8(3)
C7	C9	C10	C11	-61.0(3)		C15	C16	C20	C27	82.4(3)
C7	C9	C22	F1	20.2(5)		C15	C16	C20	C28	-39.0(3)
C7	C9	C22	F2	-99.2(4)		C16	C17	C18	C19	-15.3(3)
C7	C9	C22	F3	141.3(4)		C17	C12	C13	C14	-1.2(3)
C8	O1	C11	C10	0.6(3)		C17	C16	C20	C19	20.3(3)
C8	O1	C11	C12	179.6(2)		C17	C16	C20	C27	-97.4(3)
C8	C7	C9	C10	89.6(3)		C17	C16	C20	C28	141.1(2)
C8	C7	C9	C22	-112.9(4)		C17	C18	C19	C20	27.5(3)
C8	C9	C10	C11	0.2(3)		C18	C19	C20	C16	-28.4(3)
C8	C9	C22	F1	-50.8(4)		C18	C19	C20	C27	87.9(3)

C8	C9	C22	F2	-170.2(3)		C18	C19	C20	C28	-149.3(3)
C8	C9	C22	F3	70.2(5)		C20	C16	C17	C12	179.7(2)
C9	C7	C8	O1	-94.4(3)		C20	C16	C17	C18	-3.1(3)
C9	C7	C8	C21	118.5(4)		C21	C8	C9	C7	-111.5(3)
C9	C10	C11	O1	-0.5(3)		C21	C8	C9	C10	135.5(3)
C9	C10	C11	C12	-179.4(2)		C21	C8	C9	C22	-2.3(5)
C10	C9	C22	F1	177.3(3)		C22	C9	C10	C11	140.8(3)
C10	C9	C22	F2	57.9(4)		C23	C14	C15	C16	-177.7(2)
C10	C9	C22	F3	-61.7(5)						

Table **S23** Hydrogen Atom Coordinates (Å×104) and Isotropic Displacement Parameters (Å2×103) for **6b**.

Atom	x	y	z	U(eq)
H1	5626	156	7097	90
H2	6202	-1791	7406	106
H3	7794	-2629	6325	107
H4	8871	-1486	4956	114
H5	8347	497	4656	93
H7	5802	1762	4928	75
H10	9680	3102	6418	70
H13	7316	1467	9274	54
H15	10748	2317	11391	58
H18A	10690	4701	7847	86
H18B	11786	3707	7700	86
H19A	13296	5016	8824	89
H19B	11970	5528	9379	89
H21A	3966	2783	6741	162
H21B	4401	3419	5639	162
H21C	4885	4066	6763	162
H24A	8690	2179	12641	134
H24B	7205	1378	12806	134
H24C	7260	2482	11993	134
H25A	9257	-475	11184	127
H25B	8499	-469	12306	127
H25C	9920	413	12129	127

H26A	6033	976	10624	137
H26B	6144	-82	11473	137
H26C	6789	-180	10316	137
H27A	13558	2359	10353	112
H27B	14590	3488	9974	112
H27C	13546	2727	9113	112
H28A	11905	4890	11342	106
H28B	13591	5044	11165	106
H28C	12870	3858	11714	106

9. Characterization of some starting substrates

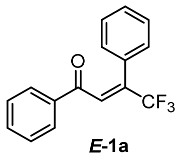

***E*-1a**

(*E*)-4,4,4-trifluoro-1,3-diphenylbut-2-en-1-one ((E)-1a)[15]: yellow oil; 83% yield; ^{1}H NMR (600 MHz, $CDCl_3$) δ 7.66 (d, J = 7.8 Hz, 2H), 7.31 (t, *J* = 7.5 Hz, 1H), 7.19 (t, *J* = 7.7 Hz, 2H), 7.13 (d, *J* = 7.5 Hz, 3H), 7.06 (d, *J* = 7.1 Hz, 3H); ^{13}C NMR (151 MHz, $CDCl_3$) δ 191.8, 138.6 (q, *J* = 30.8 Hz), 135.9, 133.7, 130.9 (q, *J* = 5.2 Hz), 130.7, 129.2, 128.9, 128.7, 128.5, 128.2, 122.8 (q, *J* = 274.8 Hz); ^{19}F NMR (565 MHz, $CDCl_3$) δ -66.1; HRMS (ESI) m/z 277.0835 (M+H$^+$), calc. for $C_{16}H_{11}F_3O$ 277.0837.

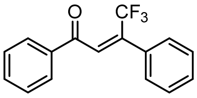

***Z*-1a**

(*Z*)-4,4,4-trifluoro-1,3-diphenylbut-2-en-1-one ((Z-)1a): yellow oil; 43% yield; ^{1}H NMR (300 MHz, $CDCl_3$) δ 8.00 (d, *J* = 8.3 Hz, 2H), 7.65 (t, *J* = 7.4 Hz, 1H), 7.58 – 7.49 (m, 4H), 7.48 – 7.42 (m, 3H), 6.84 (s, 1H); ^{13}C NMR (75 MHz, $CDCl_3$) δ 192.5, 136.1, 135.6, 134.4 (q, *J* = 3.6 Hz), 134.2, 133.5, 129. 6, 129.2, 128.9, 128.8, 127.8, 122.6 (q, *J* = 276.2 Hz); ^{19}F NMR (565 MHz, $CDCl_3$) δ -66.0; HRMS (ESI) m/z 277.0835 (M+H$^+$), calc. for $C_{16}H_{11}F_3O$ 277.0834.

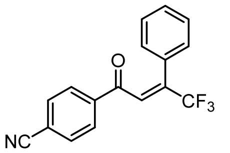

1b

***(E)*-4-(4,4,4-trifluoro-3-phenylbut-2-enoyl)benzonitrile (1b):** yellow oil; 78% yield; ^{1}H NMR (300 MHz, $CDCl_3$) δ 7.81 (d, *J* = 8.4 Hz, 2H), 7.61 (d, *J* = 8.4 Hz, 2H), 7.31 – 7.09 (m, 6H); ^{13}C NMR (75 MHz, $CDCl_3$) δ 191.0, 140.5 (q, *J* = 31.1 Hz), 138.9, 132.4, 130.4, 129.9, 129.6 (q, *J* = 5.0 Hz), 129.1, 129.0, 128.5, 122.6 (q, *J* = 275.1 Hz), 117.6, 116.8; ^{19}F NMR (376 MHz, $CDCl_3$) δ -66.0; HRMS (ESI) m/z 302.0787 (M+H$^+$), calc. for $C_{16}H_{11}F_3O$ 302.0783.

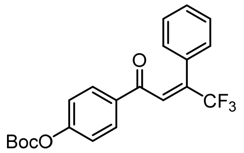

1f

***(E)*-tert-butyl (4-(4,4,4-trifluoro-3-phenylbut-2-enoyl)phenyl) carbonate (1f):** yellow solid; Mp 75.6 – 76.3 ºC; 85% yield; ^{1}H NMR (300 MHz, $CDCl_3$) δ 7.89 (d, *J* = 6.8 Hz, 2H), 7.35 – 7.25 (m, 67.30 – 7.15 (m, 2H), 1.60 (s, 9H); ^{13}C NMR (75 MHz, $CDCl_3$) δ 190.6, 155.2, 150.7, 138.9 (q, *J* = 30.8 Hz), 133.3, 130.6 (q, *J* = 5.5 Hz), 130.4, 129.4, 128.9, 128.3, 122.8 (q, *J* = 274.8 Hz), 121.3, 84.2, 27.5; ^{19}F NMR (376 MHz, $CDCl_3$) δ -65.6; HRMS (ESI) m/z 393.1308 (M+H$^+$), calc. for $C_{16}H_{11}F_3O$ 393.1310.

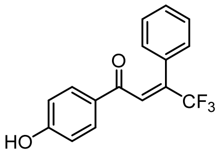

1g

***(E)*-4,4,4-trifluoro-1-(4-hydroxyphenyl)-3-phenylbut-2-en-1-one (1g):** yellow solid; Mp 98.0 – 99.2 °C; 79% yield; ^{1}H NMR (300 MHz, $CDCl_3$) δ 7.80 (d, J = 8.5 Hz, 2H), 7.41 – 6.91 (m, 6H), 6.81 (d, J = 2.0 Hz, 2H), 6.33 – 5.95 (m, 1H); ^{13}C NMR (75 MHz, $CDCl_3$) δ 191.0, 161.0, 131.8, 131.0, 130.9 (q, J = 3.9 Hz), 129.4, 129.1, 129.0, 128.4, 120.4 (q, J = 273.9 Hz), 115.6; ^{19}F NMR (376 MHz, $CDCl_3$) δ -66.0; HRMS (ESI) m/z 293.0784 ($M+H^+$), calc. for $C_{16}H_{11}F_3O$ 293.0788.

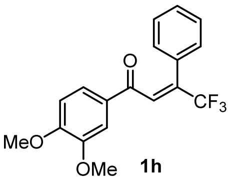

1h

***(E)*-1-(3,4-dimethoxyphenyl)-4,4,4-trifluoro-3-phenylbut-2-en-1-one (1h):** yellow solid; Mp 76.3 – 77.8 °C; 83% yield; ^{1}H NMR (300 MHz, $CDCl_3$) δ 7.45 (d, J = 2.2 Hz, 1H), 7.34 (d, J = 2.2 Hz, 1H), 7.29 – 7.18 (m, 7H), 6.80 (d, J = 2.5 Hz, 1H), 3.90 (s, 3H), 3.84 (s, 3H); ^{13}C NMR (75 MHz, $CDCl_3$) δ 190.4, 154.1, 149.3, 138.1 (d, J = 30.3 Hz), 131.0, 130.9 (q, J = 5.0 Hz), 129.3, 129.0, 128.3, 124.6,122.9 (q, J = 273.0 Hz) 110.2, 110.0, 56.1, 55.9; ^{19}F NMR (376 MHz, $CDCl_3$) δ -65.4; HRMS (ESI) m/z 337.1046 ($M+H^+$), calc. for $C_{16}H_{11}F_3O$ 337.1040.

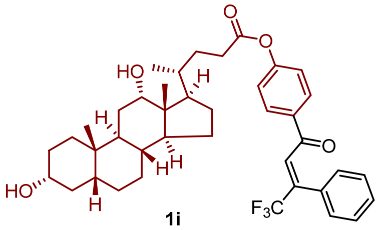

1i

4-((*E*)-4,4,4-trifluoro-3-phenylbut-2-enoyl)phenyl *(R)*-4-((3*R*,5*R*,8*R*,9*S*,10*S*,12*S*,13*R*,14*S*,17*R*)-3,12-dihydroxy-10,13-dimethylhexadecahydro-1H-cyclopenta[a]phenanthren-17-yl)pentanoate (1i): yellow solid; Mp 89.3 – 91.0 °C; 83% yield; ^{1}H NMR (300 MHz, $CDCl_3$) δ 7.86 (d, J = 8.7 Hz, 2H), 7.35 – 7.24 (m, 6H), 7.14 (d, J = 8.7 Hz, 2H), 3.68 – 3.54 (m, 2H), 2.69 – 2.45 (m, 2H), 2.07 – 1.76 (m, 8H), 1.74 – 1.42 (m, 15H), 1.34 – 1.13 (m, 6H), 0.96 (s, 3H), 0.71 (s, 3H); ^{13}C NMR (75 MHz, $CDCl_3$) δ 190.7, 171.9, 155.0, 139.1 (q, J = 31.1 Hz), 133.5, 130.7, 130.5, 129.5, 129.0, 128.4, 121.9, 71.3, 55.7, 54.9, 43.8, 42.4, 40.1, 39.2, 37.3, 36.9, 35.2, 34.9, 34.1, 33.8, 31.4, 30.8, 30.3, 28.6, 26.9, 24.8, 23.4, 21.2, 18.4, 12.1; ^{19}F NMR (565 MHz, $CDCl_3$) δ -66.8; HRMS (ESI) m/z 667.3605 ($M+H^+$), calc. for $C_{16}H_{11}F_3O$ 667.3610.

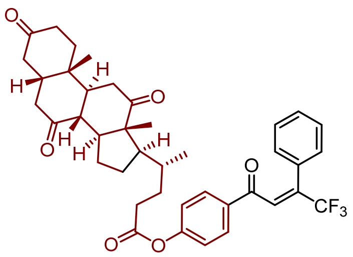

1j

4-((*E*)-4,4,4-trifluoro-3-phenylbut-2-enoyl)phenyl (*R*)-4-((5*S*,8*R*,9*S*,10*S*,13*R*,14*S*,17*R*)-10,13-dimethyl-3,7,12-trioxohexadecahydro-1H-cyclopenta[a]phenanthren-17-yl)pentanoate (1j): yellow solid; Mp 103.3 – 104.9 °C; 83% yield; ^{1}H NMR (300 MHz, $CDCl_3$) δ 7.89 (d, *J* = 8.7 Hz, 2H), 7.31 (s, 6H), 7.17 (d, *J* = 6.7 Hz, 2H), 3.72 – 3.67 (m, 1H), 3.67 – 3.58 (m, 2H), 3.55 – 3.45 (m, 1H), 2.72 – 2.59 (m, 1H), 2.59 – 2.47 (m, 1H), 2.42 – 2.21 (m, 1H), 2.02 – 1.78 (m, 10H), 1.71 – 1.52 (m, 10H), 1.36 – 1.14 (m, 11H), 1.00 – 0.97 (m, 3H), 0.73 (s, 3H); ^{13}C NMR (75 MHz, DMSO) δ 212.0, 209.5, 190.8, 171.7, 156.7, 154.9, 133.2, 130.6, 129.5, 128.9, 128.6, 123.1 (q, *J* = 274.0 Hz), 122.5, 56.3, 51.3, 48.0, 47.6, 46.1, 45.4, 44.6, 44.1, 42.6, 38.4, 36.2, 35.7, 35.0, 34.64, 33.4, 31.0, 30.1, 27.3, 25.4, 24.7, 24.5, 21.2, 11.5; ^{19}F NMR (376 MHz, $CDCl_3$) δ -66.1; HRMS (ESI) m/z 677.3085 (M+H^{+}), calc. for $C_{16}H_{11}F_3O$ 677.3080.

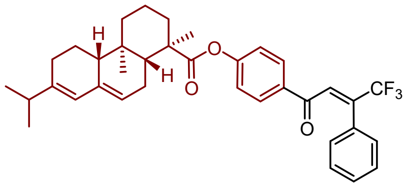

1k

4-((*E*)-4,4,4-trifluoro-3-phenylbut-2-enoyl)phenyl (1R,4aR,4bR,10aR)-7-isopropyl-1,4a-dimethyl-1,2,3,4,4a,4b,5,6,10,10a-decahydrophenanthrene-1-carboxylate (1k): yellow solid; Mp 103.3 – 104.6 °C; 75% yield; ^{1}H NMR (300 MHz, $CDCl_3$) δ 7.88 – 7.79 (m, 3H), 7.26 (q, *J* = 3.7 Hz, 5H), 7.20 – 7.06 (m, 2H), 6.25 (d, *J* = 16.0 Hz, 1H), 2.46 (d, *J* = 17.1 Hz, 2H), 2.28 (d, *J* = 17.4 Hz, 2H), 2.13 – 2.05 (m, 4H), 2.00 – 1.97 (m, 1H), 1.92 – 1.84 (m, 3H), 1.74 – 1.62 (m, 1H), 1.61 – 1.52 (m, 2H), 1.58 – 1.49 (m, 1H), 1.37 – 1.20 (m, 2H), 1.19 – 1.04 (m, 3H), 1.05 – 0.89 (m, 3H), 0.92 – 0.76 (m, 2H); ^{13}C NMR (75 MHz, $CDCl_3$) δ 197.4, 190.7, 163.2, 161.9, 155.0, 153.1, 138.0, 135.7 (q, *J* = 30.5 Hz), 133.5, 130.7, 130.6 (d, *J* = 5.2 Hz), 130.5, 129.5, 128.97, 129.0, 127.8, 127.2, 122.8 (d, *J* = 274.8 Hz), 122.0, 116.7, 49.7, 49.4, 41.5, 33.7, 25.5, 24.8, 24.3, 23.0, 21.4, 18.8; ^{19}F NMR (376 MHz, $CDCl_3$) δ -66.1; HRMS (ESI) m/z 577.2924 (M+H^{+}), calc. for $C_{16}H_{11}F_3O$ 577.2930.

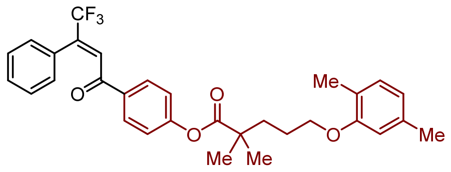

1l

(*E*)-4-(4,4,4-trifluoro-3-phenylbut-2-enoyl)phenyl 5-(2,5-dimethylphenoxy)-2,2-dimethylpentanoate (2l): yellow solid; Mp 93.5 – 94.6 °C; 83% yield; ^{1}H NMR (300 MHz, $CDCl_3$)

δ 7.92 – 7.78 (m, 2H), 7.35 – 7.29 (m, 4H), 7.12 – 7.05 (m, 2H), 7.02 (d, *J* = 7.4 Hz, 1H), 6.77 – 6.55 (m, 2H), 4.00 (d, *J* = 5.3 Hz, 2H), 2.32 (s, 3H), 2.19 (s, 3H), 1.90 (s, 4H), 1.39 (s, 6H); ^{13}C NMR (75 MHz, $CDCl_3$) δ 190.6, 175.5, 156.7, 155.3, 138.7 (q, *J* = 30.8 Hz), 136.4, 133.3, 130.6 (q, *J* = 5.4 Hz), 130.4, 130.3, 129.4, 128.9, 128.3, 123.4, 122.8 (q, *J* = 274.8 Hz), 121.9, 120.7, 111.8, 67.5, 42.5, 36.9, 25.1, 24.9, 21.3, 15.7; ^{19}F NMR (376 MHz, $CDCl_3$) δ -65.6; HRMS (ESI) m/z 525.2247 ($M+H^+$), calc. for $C_{16}H_{11}F_3O$ 525.2240.

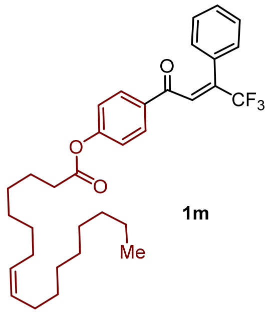

1m

4-((*E*)-4,4,4-trifluoro-3-phenylbut-2-enoyl)phenyl (Z)-heptadec-8-enoate (1m): yellow oil; 87% yield; ^{1}H NMR (300 MHz, $CDCl_3$) δ 7.90 (d, *J* = 8.3 Hz, 2H), 7.31 (d, *J* = 4.2 Hz, 6H), 7.21 – 7.12 (m, 2H), 5.41 (d, *J* = 5.3 Hz, 2H), 2.62 (d, *J* = 7.6 Hz, 2H), 2.45 – 2.34 (m, 2H), 2.08 (p, *J* = 6.4 Hz, 7H), 1.78 (d, *J* = 7.2 Hz, 2H), 1.68 – 1.65 (m, 1H), 1.38 (dd, *J* = 22.1, 13.4 Hz, 18H); ^{13}C NMR (75 MHz, $CDCl_3$) δ 190.9, 179.7, 171.5, 155.1, 139.1 (q, *J* = 31.2 Hz), 133.4, 131.7, 130.5, 130.0, 130.0 (q, *J* = 4.7 Hz), 130.0, 129.7, 129.6, 129.5, 129.23 128.94 128.4, 128.3, 122.8 (q, *J* = 274.8 Hz), 121.9, 115.6, 34.3, 34.0, 31.9, 29.7, 29.6, 29.5, 29.3, 29.0, 27.2, 27.1, 24.7, 24.6, 22.6, 14.1; ^{19}F NMR (376 MHz, $CDCl_3$) δ -66.1; HRMS (ESI) m/z 543.3081 ($M+H^+$), calc. for $C_{16}H_{11}F_3O$ 543.3077.

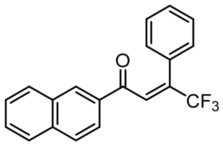

1n

(*E*)-4,4,4-trifluoro-1-(naphthalen-2-yl)-3-phenylbut-2-en-1-one (1n)[15]: yellow oil; 87% yield; ^{1}H NMR (600 MHz, $CDCl_3$) δ 8.19 (s, 1H), 7.77 – 7.71 (m, 2H), 7.64 (t, *J* = 7.9 Hz, 2H), 7.42 (t, *J* = 7.5 Hz, 1H), 7.37 (t, *J* = 7.5 Hz, 1H), 7.21 – 7.15 (m, 2H), 7.06 (dd, *J* = 4.6, 2.6 Hz, 3H); ^{13}C NMR (151 MHz, $CDCl_3$) δ 191.7, 138.7 (q, *J* = 30.8 Hz), 135.8, 133.3, 132.2, 131.4, 131.0 (q, *J* = 5.0 Hz), 130.8, 129.6, 129.3, 128.9, 128.3, 127.7, 126.9, 123.6, 122.9 (q, *J* = 274.5 Hz); ^{19}F NMR (565 MHz, $CDCl_3$) δ -66.0; HRMS (ESI) m/z 327.0991 ($M+H^+$), calc. for $C_{20}H_{13}F_3O$ 329.0992.

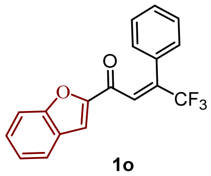

1o

***(E)*-1-(benzofuran-2-yl)-4,4,4-trifluoro-3-phenylbut-2-en-1-one (1o):** yellow solid; Mp 55.5 – 55.9 °C; 83% yield; ^{1}H NMR (600 MHz, $CDCl_3$) δ 7.68 (d, *J* = 1.0 Hz, 1H), 7.55 (d, *J* = 0.9 Hz, 1H), 7.51 – 7.46 (m, 2H), 7.42 (q, *J* = 1.4 Hz, 1H), 7.38 – 7.33 (m, 5H), 7.33 – 7.28 (m, 1H); ^{13}C NMR (151 MHz, $CDCl_3$) δ 179.9, 155.8, 152.2, 141.4 (q, *J* = 30.8 Hz), 130.7, 129.5, 128.9,

128.8, 128.3, 127.7 (q, J = 5.0 Hz), 126.8, 124.1, 123.5, 22.7 (q, J = 275.1 Hz), 115.0, 112.5; ^{19}F NMR (565 MHz, $CDCl_3$) δ -66.8; HRMS (ESI) m/z 317.0784 (M+H$^+$), calc. for $C_{20}H_{13}F_3O$ 317.0784.

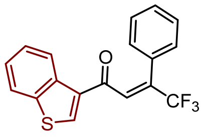

1p

***(E)*-1-(benzo[b]thiophen-3-yl)-4,4,4-trifluoro-3-phenylbut-2-en-1-one (2p):** yellow solid; Mp 63.7 – 64.5 °C; 83% yield; ^{1}H NMR (300 MHz, $CDCl_3$) δ 8.76 (d, J = 7.2 Hz, 2H), 8.35 (s, 1H), 7.94 (d, J = 7.0 Hz, 1H), 7.72 – 7.52 (m, 2H), 7.50 – 7.41 (m, 4H); ^{13}C NMR (75 MHz, $CDCl_3$) δ 186.0,139.9, 139.8, 138.5 (q, J = 30.5 Hz), 135.9, 134.6, 131.1 (q, J = 5.0 Hz), 130.9, 129.4, 128.9, 128.4, 126.0, 125.8, 125.3, 122.9 (q, J = 275.1 Hz), 122.2; ^{19}F NMR (376 MHz, $CDCl_3$) δ -66.1; HRMS (ESI) m/z 333.0556 (M+H$^+$), calc. for $C_{20}H_{13}F_3O$ 333.0560.

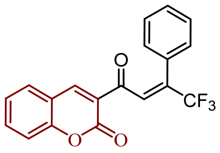

1q

***(E)*-3-(4,4,4-trifluoro-3-phenylbut-2-enoyl)-2H-chromen-2-one (2q):** yellow solid; Mp 68.9 – 70.3 °C; 72% yield; ^{1}H NMR (600 MHz, $CDCl_3$) δ 8.20 (s, 1H), 7.67 – 7.62 (m, 1H), 7.55 (d, J = 7.8 Hz, 1H), 7.48 (d, J = 2.9 Hz, 1H), 7.35 – 7.30 (m, 2H), 7.29 (s, 5H); ^{13}C NMR (151 MHz, $CDCl_3$) δ 188.0, 158.6, 155.2, 147.9, 139.2 (q, J = 30.8 Hz), 134.8, 131.4 (q, J = 5.5 Hz), 131.1, 130.1, 129.3, 129.2, 128.2, 125.1, 124.5, 122.7 (q, J = 274.5 Hz), 118.0, 116.6; ^{19}F NMR (565 MHz, $CDCl_3$) δ -67.1; HRMS (ESI) m/z 345.0733 (M+H$^+$), calc. for $C_{20}H_{13}F_3O$ 345.0737.

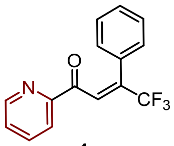

1r

***(E)*-4,4,4-trifluoro-3-phenyl-1-(pyridin-2-yl)but-2-en-1-one (1r):** yellow solid; Mp 73.8 – 74.3 °C; 89% yield; ^{1}H NMR (300 MHz, $CDCl_3$) δ 8.75 (d, J = 3.8 Hz, 1H), 8.13 – 8.06 (m, 1H), 7.96 (d, J = 7.8 Hz, 1H), 7.84 (t, J = 1.7 Hz, 1H), 7.57 – 7.48 (m, 1H), 7.47 – 7.32 (m, 5H); ^{13}C NMR (75 MHz, $CDCl_3$) δ 190.3, 153.5, 149.1, 141.3 (q, J = 30.8 Hz), 137.0, 131.5, 129.1, 128.9, 128.4 (q, J = 5.8 Hz), 128.2, 127.5, 122.8 (q, J = 275.1 Hz), 122.6; ^{19}F NMR (376 MHz, $CDCl_3$) δ -67.0; HRMS (ESI) m/z 278.0787 (M+H$^+$), calc. for $C_{20}H_{13}F_3O$278.0790.

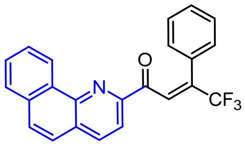

1t

***(E)*-1-(benzo[h]quinolin-2-yl)-4,4,4-trifluoro-3-phenylbut-2-en-1-one (1t):** yellow solid; Mp 85.6 – 86.3 °C; 73% yield; ^{1}H NMR (300 MHz, $CDCl_3$) δ 9.34 (d, J = 8.2 Hz, 1H), 8.43 (s, 1H), 8.24 (d, J = 3.0 Hz, 1H), 8.18 – 8.09 (m, 1H), 8.02 – 7.90 (m, 2H), 7.87 – 7.65 (m, 2H), 7.36 (s, 8H); ^{13}C NMR (75 MHz, $CDCl_3$) δ 190.5, 151.5, 145.5, 140.8 (q, J = 33.6 Hz), 136.8, 133.8, 131.7, 131.4, 130.7, 129.0, 128.9, 128.7, 128.2, 128.1, 127.9, 124.8, 124.5, 122.3 (q, J

= 273.9 Hz), 122.3 (q, J = 4.7 Hz), 121.2, 119.6; ^{19}F NMR (376 MHz, $CDCl_3$) δ -66.9; HRMS (ESI) m/z 378.1100 (M+H$^+$), calc. for $C_{20}H_{13}F_3O$ 378.1108.

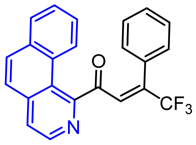

1u

***(E)*-1-(benzo[h]isoquinolin-1-yl)-4,4,4-trifluoro-3-phenylbut-2-en-1-one (1u):** yellow solid; Mp 77.9 – 78.3 °C; 88% yield; ^{1}H NMR (300 MHz, $CDCl_3$) δ 9.34 (d, J = 8.3 Hz, 1H), 8.43 (s, 1H), 8.29 – 8.20 (m, 1H), 8.17 – 8.09 (m, 1H), 8.01 – 7.90 (m, 1H), 7.88 – 7.83 (m, 2H), 7.82 – 7.75 (m, 2H), 7.76 – 7.67 (m, 1H), 7.36 (s, 5H); ^{13}C NMR (75 MHz, $CDCl_3$) δ 190.5, 151.4, 145.4, 142.3 (q, J = 30.4 Hz), 136.8, 133.8, 131.6, 131.4, 130.7, 129.1, 128.9, 128.8, 128.6, 128.3 (q, J = 2.9 Hz), 128.2, 128.1, 127.9, 124.8, 124.5, 122.7 (q, J = 274.0 Hz) 119.5; ^{19}F NMR (376 MHz, $CDCl_3$) δ -66.9; HRMS (ESI) m/z 378.1100 (M+H$^+$), calc. for $C_{20}H_{13}F_3O$ 378.1103.

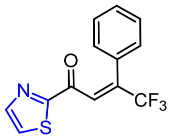

1v

***(E)*-4,4,4-trifluoro-3-phenyl-1-(thiazol-2-yl)but-2-en-1-one (1v):** yellow solid; Mp 83.5 – 83.9 °C; 93% yield; ^{1}H NMR (600 MHz, $CDCl_3$) δ 8.05 (d, J = 3.0 Hz, 1H), 7.93 (d, J = 1.4 Hz, 1H), 7.70 (d, J = 3.0 Hz, 1H), 7.44 – 7.38 (m, 3H), 7.35 – 7.30 (m, 2H); ^{13}C NMR (151 MHz, $CDCl_3$) δ 181.4, 167.18,145.1, 143.3 (q, J = 31.1 Hz), 131.0, 129.4, 128.7, 128.3, 127.4, 126.2 (q, J = 5.2 Hz), 122.5 (q, J = 275.1 Hz); ^{19}F NMR (565 MHz, $CDCl_3$) δ -67.3; HRMS (ESI) m/z 284.0352 (M+H$^+$), calc. for $C_{20}H_{13}F_3O$ 284.0355.

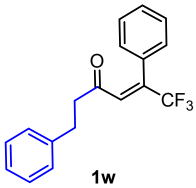

1w

***(E)*-6,6,6-trifluoro-1,5-diphenylhex-4-en-3-one (1w):** yellow oil; 87% yield; ^{1}H NMR (300 MHz, $CDCl_3$) δ 7.51 – 7.40 (m, 4H), 7.37 – 7.25 (m, 4H), 7.10 – 6.97 (m, 2H), 6.85 – 6.67 (m, 1H), 2.81 (t, J = 7.4 Hz, 2H), 2.58 (t, J = 7.5 Hz, 2H); ^{13}C NMR (75 MHz, $CDCl_3$) δ 200.6, 140.2, 138.4 (q, J = 30.9 Hz), 131.9 (p, J = 5.0 Hz), 130.8, 129.6, 128.9, 128.6, 128.3, 128.1, 126.1, 122.7 (d, J = 274.8 Hz), 44.5, 29.3; ^{19}F NMR (376 MHz, $CDCl_3$) δ -66.8; HRMS (ESI) m/z 305.1148 (M+H$^+$), calc. for $C_{20}H_{13}F_3O$ 305.1150.

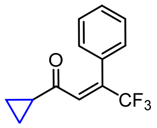

1x

***(E)*-1-cyclopropyl-4,4,4-trifluoro-3-phenylbut-2-en-1-one (1x):** yellow oil; 74% yield; ^{1}H NMR (300 MHz, $CDCl_3$) δ 7.38 – 7.25 (m, 5H), 6.71 (s, 1H), 1.64 – 1.48 (m, 1H), 0.95 – 0.82 (m, 2H), 0.64 – 0.53 (m, 2H); ^{13}C NMR (75 MHz, $CDCl_3$) δ 201.8, 138.3 (q, J = 30.8 Hz), 132.6 (q, J = 5.0 Hz), 131.1, 129.6, 129.2, 128.4, 122.9 (q, J = 274.8 Hz), 22.1, 12.8; ^{19}F NMR (376 MHz, $CDCl_3$) δ -65.6; HRMS (ESI) m/z 241.0835 (M+H$^+$), calc. for $C_{20}H_{13}F_3O$ 241.0840.

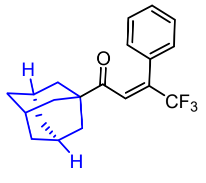

1y

***(E)*-1-(adamantan-1-yl)-4,4,4-trifluoro-3-phenylbut-2-en-1-one (1y):** yellow oil; 76% yield; ^{1}H NMR (300 MHz, $CDCl_3$) δ 7.43 – 7.39 (m, 2H), 7.35 – 7.25 (m, 2H), 7.15 – 7.09 (m, 1H), 2.07 (s, 2H), 1.86 – 1.64 (m, 9H); ^{1}3C NMR (75 MHz, CDC13) δ 205.3, 138.5 (q, J = 30.5 Hz), 131.1, 129.1 (q, J = 4.7 Hz), 129.0, 128.0, 122.7 (q, J = 274.8 Hz), 46.5, 37.6, 36.2; ^{19}F NMR (376 MHz, $CDCl_3$) δ -66.6; HRMS (ESI) m/z 335.1617 ($M+H^+$), calc. for $C_{20}H_{13}F_3O$ 335.1620.

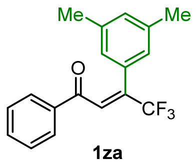

1za

(*E*)-3-(3,5-dimethylphenyl)-4,4,4-trifluoro-1-phenylbut-2-en-1-one (1za): yellow oil; 83% yield; ^{1}H NMR (300 MHz, $CDCl_3$) δ 7.80 – 7.73 (m, 2H), 7.44 – 7.35 (m, 1H), 7.28 (t, J = 7.4 Hz, 2H), 7.17 (s, 1H), 6.82 (d, J = 13.0 Hz, 3H), 2.11 (s, 6H); ^{13}C NMR (75 MHz, $CDCl_3$) δ 192.0, 138.9 (q, J = 30.7 Hz), 137.7, 136.0, 133.5, 130.9, 130.4 (q, J = 5.1 Hz), 128.6, 128.4, 126.6, 122.9 (q, J = 274.8 Hz), 21.1; ^{19}F NMR (376 MHz, $CDCl_3$) δ -66.0; HRMS (ESI) m/z 305.1148 ($M+H^+$), calc. for $C_{18}H_{15}F_3O$ 305.1148.

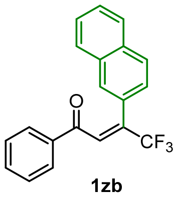

1zb

(*E*)-4,4,4-trifluoro-3-(naphthalen-2-yl)-1-phenylbut-2-en-1-one (1zb)[15]: yellow solid; Mp 73.6 – 74.3 °C; 93% yield; ^{1}H NMR (300 MHz, $CDCl_3$) δ 7.85 (d, J = 7.2 Hz, 2H), 7.81 – 7.70 (m, 4H), 7.48 (q, J = 5.6 Hz, 3H), 7.37 (t, J = 7.6 Hz, 4H); ^{13}C NMR (75 MHz, $CDCl_3$) δ 192.1, 139.0 (q, J = 30.9 Hz), 136.1, 134.0, 133.3, 132.7, 131.1 (q, J = 5.0 Hz), 129.2, 128.9, 128.7, 128.4, 128.3, 128.2, 127.7, 127.1, 126.6, 126.1, 123.0 (q, J = 275.1 Hz); ^{19}F NMR (376 MHz, $CDCl_3$) δ -65.6; HRMS (ESI) m/z 327.0991 ($M+H^+$), calc. for $C_{20}H_{13}F_3O$ 327.0989.

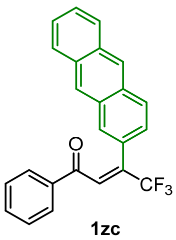

1zc

(*E*)-3-(anthracen-2-yl)-4,4,4-trifluoro-1-phenylbut-2-en-1-one (1zc): yellow solid; Mp 77.3 – 78.2 °C; 93% yield; ^{1}H NMR (300 MHz, $CDCl_3$) δ 8.34 (s, 2H), 7.96 (d, J = 12.4 Hz, 3H), 7.91 – 7.80 (m, 3H), 7.45 (t, J = 6.7 Hz, 3H), 7.40 – 7.27 (m, 4H); ^{13}C NMR (75 MHz, $CDCl_3$) δ 192.2, 139.1 (q, J = 31.0 Hz), 136.0, 133.9, 132.3, 131.9, 131.1 (q, J = 5.0 Hz), 130.9, 130.5, 129.8, 128.8, 128.7, 128.5, 128.2, 128.1, 127.8, 127.2, 126.1, 126.0, 125.7, 125.2, 123.1 (q, J = 275.1 Hz); ^{19}F NMR (376 MHz, $CDCl_3$) δ -66.0; HRMS (ESI) m/z 377.1148 ($M+H^+$), calc. for $C_{24}H_{15}F_3O$ 377.1150.

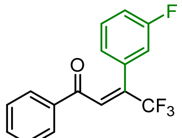

1zd

(*E*)-4,4,4-trifluoro-3-(3-fluorophenyl)-1-phenylbut-2-en-1-one (1zd): yellow oil; 88% yield; ^{1}H NMR (300 MHz, $CDCl_3$) δ 7.85 (d, *J* = 7.5 Hz, 2H), 7.59 – 7.49 (m, 1H), 7.45 – 7.33 (m, 3H), 7.26 – 7.16 (m, 1H), 7.08 (d, *J* = 7.7 Hz, 2H), 7.05 – 6.92 (m, 1H); ^{13}C NMR (75 MHz, $CDCl_3$) δ 191.3, 162.3 (d, *J* = 247.3 Hz), 137.6 (q, *J* = 31.2 Hz), 136.0, 134.2, 132.8 (d, *J* = 8.3 Hz), 131.7 (d, *J* = 5.2 Hz), 128.9, 128.8, 125.1 (d, *J* = 3.3 Hz), 122.7 (q, *J* = 274.7 Hz), 116.7, 116.37, 116.09; ^{19}F NMR (376 MHz, $CDCl_3$) δ -66.3, -112.2; HRMS (ESI) m/z 295.0741 ($M+H^+$), calc. for $C_{16}H_{10}F_4O$ 295.0743.

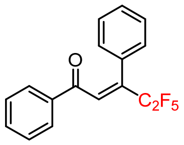

1ze

(*E*)-4,4,5,5,5-pentafluoro-1,3-diphenylpent-2-en-1-one (1ze): yellow oil; 65% yield; ^{1}H NMR (600 MHz, $CDCl_3$) δ 7.85 (d, *J* = 7.7 Hz, 2H), 7.57 (t, *J* = 7.5 Hz, 1H), 7.45 (t, *J* = 7.7 Hz, 2H), 7.37 (s, 1H), 7.33 – 7.25 (m, 5H); ^{13}C NMR (151 MHz, $CDCl_3$) δ 191.8, 138.1 (t, *J* = 21.5 Hz), 136.1, 134.4 (t, *J* = 7.7 Hz), 133.9, 130.9, 129.4, 129.3, 128.8, 128.7, 128.2, 118.9 (dt, *J* = 287.4, 38.1 Hz), 112.4 (tq, *J* = 256.5, 38.0Hz); 19F NMR (565 MHz, CDCl3) δ -82.2, -114.5; HRMS (ESI) m/z 327.0803 ($M+H^+$), calc. for $C_{17}H_{11}F_5O$ 327.0804.

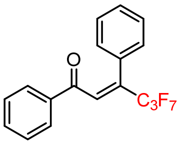

1zf

***(E)*-4,4,5,5,6,6,6-heptafluoro-1,3-diphenylhex-2-en-1-one (1zf):** yellow oil; 64% yield; ^{1}H NMR (300 MHz, $CDCl_3$) δ 7.80 – 7.72 (m, 2H), 7.48 (t, *J* = 7.6 Hz, 2H), 7.38 – 7.30 (m, 3H), 7.20 (s, 4H); ^{13}C NMR (75 MHz, $CDCl_3$) δ 191.9, 138.1 (t, *J* = 22.0 Hz), 136.1, 134.9 (t, *J* = 8.1 Hz), 133.9, 131.1, 129.6, 129.3, 128.8, 128.7, 128.2, 128.1, 126.4, 112.4 (tq, *J* = 262.2, 30.3 Hz); ^{19}F NMR (376 MHz, $CDCl_3$) δ -81.1, -116.5 – -117.3 (m, 2F), -121.8 – -125. 2(m, 2F); HRMS (ESI) m/z 377.0771 ($M+H^+$), calc. for $C_{17}H_{11}F_5O$ 377.0780.

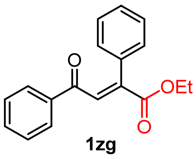

1zg

Ethyl *(E)*-4-oxo-2,4-diphenylbut-2-enoate (1zg)[12]: yellow oil; 53% yield; ^{1}H NMR (300 MHz, $CDCl_3$) δ 7.92 – 7.79 (m, 2H), 7.74 (d, *J* = 2.9 Hz, 1H), 7.56 – 7.50 (m, 1H), 7.41 (t, *J* = 7.2 Hz, 2H), 7.27 (d, *J* = 2.8 Hz, 5H), 4.38 (q, *J* = 7.2 Hz, 2H), 1.39 (t, *J* = 7.2 Hz, 3H); 13C NMR (75 MHz, CDCl3) δ 193.8, 166.4, 140.7, 136.1, 136.0, 133.7, 133.5, 129.4, 128.9, 128.5, 127.8, 61.9, 14.1; HRMS (ESI) m/z 280.1172 ($M+H^+$), calc. for $C_{18}H_{16}O_3$ 280.1175;

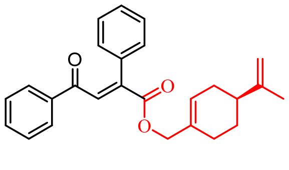

1zh

***(S)*-(4-(prop-1-en-2-yl)cyclohex-1-en-1-yl)methyl *(E)*-4-oxo-2,4-diphenylbut-2-enoate (1zh)[12]:** yellow oil; 57% yield; ^{1}H NMR (300 MHz, $CDCl_3$) δ 7.91 – 7.83 (m, 2H), 7.73 (s, 1H), 7.53 (t, J = 7.5 Hz, 1H), 7.40 (t, J = 7.5 Hz, 2H), 7.26 (s, 5H), 5.83 (d, J = 4.8 Hz, 1H), 4.78 (s, 1H), 4.77 (s, 1H), 4.71 (s, 2H), 2.30 – 2.07 (m, 4H), 2.10 – 1.98 (m, 1H), 1.96 – 1.86 (m, 1H), 1.79 (s, 3H), 1.61 – 1.47 (m, 1H), 1.40 – 1.25 (m, 1H); ^{13}C NMR (75 MHz, $CDCl_3$) δ 193.6, 166.2, 149.3, 140.5, 136.1, 136.0, 133.7, 133.5, 132.0, 129.3, 128.8, 128.4, 127.7, 126.1, 108.8, 69.6, 40.6, 30.3, 27.1, 26.3, 20.6; HRMS (ESI) m/z 387.1955 (M+H$^+$), calc. for $C_{18}H_{16}O_3$ 387.1969;

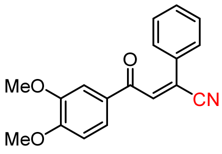

1zi

***(E)*-4-(3,4-dimethoxyphenyl)-4-oxo-2-phenylbut-2-enenitrile (1zi)[12]:** yellow solid; Mp 83.3 – 84.7 ºC; 93% yield; ^{1}H NMR (300 MHz, $CDCl_3$) δ 7.90 (s, 1H), 7.82 – 7.75 (m, 2H), 7.68 – 7.59 (m, 2H), 7.55 – 7.45 (m, 3H), 6.92 (d, J = 8.2 Hz, 1H), 3.97 (s, 3H), 3.95 (s, 3H); ^{13}C NMR (75 MHz, $CDCl_3$) δ 185.2, 154.4, 149.6, 133.3, 132.6, 131.3, 129.9, 129.3, 127.1, 124.1, 123.5, 116.1, 110.6, 110.0, 56.2, 56.0; HRMS (ESI) m/z 294.1125 (M+H$^+$), calc. for $C_{18}H_{16}O_3$ 294.1130;

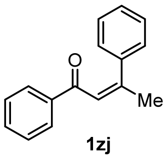

1zj

(*Z*)-1,3-diphenylbut-2-en-1-one (1zj)[13]: yellow oil; 63% yield; ^{1}H NMR (300 MHz, CDCl3) δ 8.11 – 8.01 (m, 2H), 7.72 – 7.59 (m, 3H), 7.59 – 7.43 (m, 5H), 7.34 – 7.19 (m, 1H), 2.69 (s, 3H); ^{13}C NMR (75 MHz, CDCl3) δ 191.7, 155.0, 142.6, 139.2, 132.4, 129.0, 128.5, 128.4, 128.2, 126.4, 122.0, 18.8; HRMS (ESI) m/z 223.1118 (M+H$^+$), calc. for $C_{16}H_{14}O$ 223.1118.

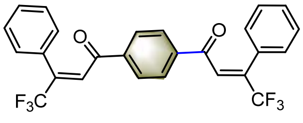

1zk

***(2E,2'E)*-1,1'-(1,4-phenylene)bis(4,4,4-trifluoro-3-phenylbut-2-en-1-one) (1zk):** yellow solid; Mp 75.3 – 77.0 ºC; 78% yield; ^{1}H NMR (300 MHz, $CDCl_3$) δ 7.82 (s, 4H), 7.39 – 7.19 (m, 12H); ^{13}C NMR (75 MHz, $CDCl_3$) δ 191.4, 139.9 (q, J = 31.1 Hz), 139.4, 130.5, 130.0 (q, J = 5.2 Hz), 129.6,128.9, 128.9, 128.4, 122.7 (q, J = 274.8 Hz); ^{19}F NMR (376 MHz, $CDCl_3$) δ -66.3; HRMS (ESI) m/z 474.1054 (M+H$^+$), calc. for $C_{16}H_{14}O$ 474.1060.

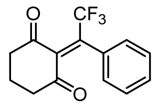

1zl

2-(2,2,2-trifluoro-1-phenylethylidene)cyclohexane-1,3-dione (1zl)[14]: yellow solid; Mp 130.2 – 131.8 °C; 47% yield; ^{1}H NMR (300 MHz, $CDCl_3$) δ 7.44 (s, 3H), 7.25 (s, 2H), 2.89 (t, J = 6.7 Hz, 2H), 2.68 (t, J = 6.7 Hz, 2H), 2.21 – 1.98 (m, 2H); ^{13}C NMR (75 MHz, $CDCl_3$) δ 198.0, 196.8, 147.9, 136.4 (q, J = 32.9 Hz), 130.7, 129.3, 128.3, 128.2, 121.6 (q, J = 277.6 Hz), 42.0, 41.20, 17.7; ^{19}F NMR (376 MHz, $CDCl_3$) δ -57.8; HRMS (ESI) m/z 269.0784 (M+H$^+$), calc. for $C_{14}H_{12}F_3O_2$ 269.0788.

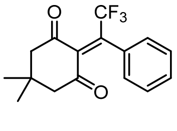

1zm

5,5-dimethyl-2-(2,2,2-trifluoro-1-phenylethylidene)cyclohexane-1,3-dione (1zm)[14]: yellow solid; Mp 93.0 – 91.5 °C; 70% yield; ^{1}H NMR (300 MHz, $CDCl_3$) δ 7.44 – 7.34 (m, 3H), 7.20 (d, J = 6.5 Hz, 2H), 2.82 (s, 2H), 2.61 (s, 2H), 1.10 (s, 6H); ^{13}C NMR (75 MHz, $CDCl_3$) δ 197.1, 196.1, 147.1, 136.6 (q, J = 33.0 Hz), 129.4, 128.4, 128.3, 123.5 (q, J = 268.1 Hz), 118.8, 56.6, 55.8, 30.8, 28.4; ^{19}F NMR (376 MHz, $CDCl_3$) δ -56.5; HRMS (ESI) m/z 297.1097 (M+H$^+$), calc. for $C_{16}H_{16}F_3O_2$ 297.1100.

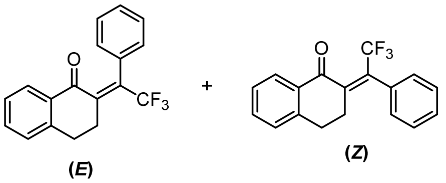

1zn

(*E*)-2-(2,2,2-trifluoro-1-phenylethylidene)-3,4-dihydronaphthalen-1(2H)-one and (*Z*)-2-(2,2,2-trifluoro-1-phenylethylidene)-3,4-dihydronaphthalen-1(2H)-one (1zn): yellow solid; 63% yield, 5:1 Z/E; Mixed substrate; ^{1}H NMR (400 MHz, $CDCl_3$) δ 7.75 (d, J = 8.5 Hz, 1H), 7.41 – 7.32 (m, 2H), 7.23 – 7.21 (m, 2H), 7.19 – 7.15 (m, 2H), 7.07 – 6.98 (m, 2H), 3.14 – 3.09 (m, 2H), 3.08 – 3.02 (m, 2H); ^{13}C NMR (101 MHz, $CDCl_3$) δ 189.0, 145.0 (q, J = 2.5 Hz), 143.1, 134.0 (q, J = 35.0 Hz), 133.8, 133.6, 129.0, 128.5, 128.3, 128.0 128.0, 127.1, 123.3 (q, J = 277.0 Hz), 30.6, 30.2; ^{19}F NMR (376 MHz, $CDCl_3$) δ -56.7; HRMS (ESI) m/z 303.0991 (M+H$^+$), calc. for $C_{16}H_{16}F_3O_2$ 303.0988.

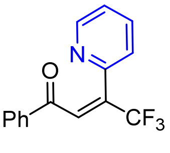

3a

***(E)*-4,4,4-trifluoro-1-phenyl-3-(pyridin-2-yl)but-2-en-1-one (3a):** yellow solid; Mp 77.3 – 78.1 °C; 83% yield; ^{1}H NMR (300 MHz, $CDCl_3$) δ 8.32 (d, J = 4.0 Hz, 1H), 7.89 (d, J = 7.0 Hz, 2H), 7.64 (t, J = 6.9 Hz, 1H), 7.56 – 7.46 (m, 2H), 7.46 – 7.35 (m, 2H), 7.24 (d, J = 1.4 Hz, 1H), 7.16 – 7.09 (m, 1H); ^{13}C NMR (75 MHz, $CDCl_3$) δ 192.4, 148.9, 148.7, 136.4, 136.1, 135.6 (q, J = 30.3 Hz), 133.6 (q, J = 5.2 Hz), 133.2, 128.5, 128.3, 123.6, 122.8 (q, J = 274.3 Hz), 122.8 (q, J = 2.2 Hz); ^{19}F NMR (376 MHz, $CDCl_3$) δ -67.1; HRMS (ESI) m/z 278.0787 (M+H$^+$), calc. for

$C_{16}H_{16}F_3O_2$ 278.0789.

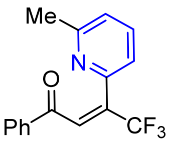

3b

***(E)*-4,4,4-trifluoro-3-(6-methylpyridin-2-yl)-1-phenylbut-2-en-1-one (3b):** yellow solid; Mp 73.3 – 73.7 °C; 86% yield; ^{1}H NMR (300 MHz, $CDCl_3$) δ 7.87 (d, J = 7.2 Hz, 2H), 7.49 (q, J = 7.4 Hz, 2H), 7.43 – 7.33 (m, 2H), 7.29 (d, J = 7.8 Hz, 1H), 7.12 – 7.06 (m, 1H), 6.92 (d, J = 7.7 Hz, 1H), 2.07 (s, 3H); ^{13}C NMR (75 MHz, $CDCl_3$) δ 193.0, 157.9, 147.4, 136.9, 136.5, 134.8 (q, J = 30.1 Hz), 133.8 (q, J = 5.5 Hz), 132.9, 128.4, 128.3, 123.4, 123.0 (q, J = 274.0 Hz), 119.3, 22.9; ^{19}F NMR (376 MHz, $CDCl_3$) δ -63.3; HRMS (ESI) m/z 292.0944 (M+H$^+$), calc. for $C_{16}H_{16}F_3O_2$ 292.0947.

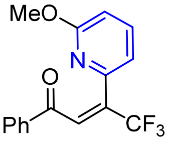

3c

***(E)*-4,4,4-trifluoro-3-(6-methoxypyridin-2-yl)-1-phenylbut-2-en-1-one (3c):** yellow solid; Mp 75.6 – 77.9 °C; 80% yield; ^{1}H NMR (300 MHz, $CDCl_3$) δ 7.91 (d, J = 8.6 Hz, 2H), 7.59 – 7.48 (m, 2H), 7.47 – 7.36 (m, 2H), 7.15 (d, J = 7.6 Hz, 1H), 7.08 – 7.01 (m, 1H), 6.60 (d, J = 8.3 Hz, 1H), 3.28 (s, 3H); ^{13}C NMR (75 MHz, $CDCl_3$) δ 193.3, 163.2, 145.7, 139.0, 135.8, 134.5 (q, J = 30.0 Hz), 133.6, 133.5, 128.9, 128.6, 123.0 (q, J = 274.3 Hz), 115.6 (q, J = 2.8 Hz), 112.4, 53.4; ^{19}F NMR (376 MHz, $CDCl_3$) δ -63.9; HRMS (ESI) m/z 309.0893 (M+H$^+$), calc. for $C_{16}H_{16}F_3O_2$ 309.0899.

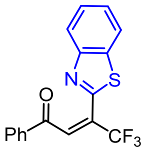

3d

***(Z)*-3-(benzo[d]thiazol-2-yl)-4,4,4-trifluoro-1-phenylbut-2-en-1-one (3d):** yellow solid; Mp 66.3 – 66.9 °C; 83% yield; ^{1}H NMR (300 MHz, $CDCl_3$) δ 7.93 – 7.87 (m, 2H), 7.84 – 7.78 (m, 1H), 7.75 – 7.69 (m, 1H), 7.46 (dd, J = 10.0, 4.7 Hz, 1H), 7.41 – 7.27 (m, 5H); ^{13}C NMR (75 MHz, $CDCl_3$) δ 191.7, 155.7, 152.3, 135.8 (q, J = 4.8 Hz), 135.5, 135.4, 133.8, 130.1 (q, J = 32.2 Hz), 128.6, 128.5, 126.4, 126.2, 123.8, 121.2, 118.3 (q, J = 274.8 Hz); ^{19}F NMR (376 MHz, $CDCl_3$) δ -65.6; HRMS (ESI) m/z 334.0508 (M+H$^+$), calc. for $C_{16}H_{16}F_3O_2$ 334.0500.

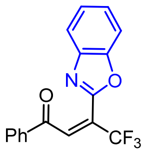

3e

***(E)*-3-(benzo[d]oxazol-2-yl)-4,4,4-trifluoro-1-phenylbut-2-en-1-one (3e):** yellow solid; Mp 73.3 – 75.0 °C; 83% yield; ^{1}H NMR (300 MHz, $CDCl_3$) δ 7.99 – 7.93 (m, 2H), 7.70 (td, J = 5.2, 2.2 Hz, 1H), 7.64 – 7.56 (m, 1H), 7.51 – 7.43 (m, 4H), 7.36 – 7.29 (m, 2H); ^{13}C NMR (75 MHz, $CDCl_3$) δ 191.4, 154.6, 150.2, 140.8, 138.11 (q, J = 4.7 Hz), 134.4, 129.0, 128.8, 126.7, 125.2, 121.40 (q, J = 274.5 Hz), 124.14 (d, J = 33.0 Hz), 121.1, 110.8; ^{19}F NMR (376 MHz, $CDCl_3$) δ -66.1; HRMS (ESI) m/z

318.0737 ($M+H^+$), calc. for $C_{16}H_{16}F_3O_2$ 318.0736.

10. Characterization of adducts

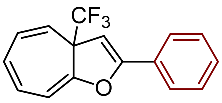

2a

2-phenyl-3a-(trifluoromethyl)-3a*H*-cyclohepta[b]furan (2a): white solid; Mp 42.1 – 42.4 °C; 26.2 mg, 95% yield; ^{1}H NMR (400 MHz, $CDCl_3$) δ 7.70 (d, *J* = 3.2 Hz, 2H), 7.42 (t, *J* = 3.4 Hz, 3H), 6.58 – 6.38 (m, 4H), 5.82 (s, 1H), 5.30 (d, *J* = 9.9 Hz, 1H); ^{13}C NMR (101 MHz, $CDCl_3$) δ 157.3, 146.0, 129.9, 128.9, 128.6, 128.2, 126.3 (q, *J* = 286.5 Hz), 125.9, 125.6, 125.4, 112.5, 104.9, 99.2, 58.6 (q, *J* = 31.1 Hz); ^{19}F NMR (376 MHz, $CDCl_3$) δ -76.0; HRMS (ESI) m/z 277.0835 (M+H$^+$), calc. for $C_{16}H_{11}F_3O$ 277.0837.

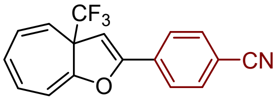

2b

4-(3a-(trifluoromethyl)-3a*H*-cyclohepta[b]furan-2-yl)benzonitrile (2b): white solid; Mp 107.6 – 108.3 °C; 22.9 mg, 76% yield; ^{1}H NMR (600 MHz, $CDCl_3$) δ 7.77 (d, *J* = 8.5 Hz, 2H), 7.70 (d, *J* = 8.5 Hz, 2H), 6.55 – 6.43 (m, 4H), 5.97 – 5.94 (m, 1H), 5.29 (d, *J* = 10.2 Hz, 1H); ^{13}C NMR (151 MHz, $CDCl_3$) δ 155.5, 145.5, 132.6, 132.4, 129.4, 126.3, 126.2 (q, *J* = 286.9 Hz), 126.0, 126.0, 118.5, 113.5, 112.2, 105.7, 102.6, 58.9 (q, *J* = 31.3 Hz); ^{19}F NMR (565 MHz, $CDCl_3$) δ -75.9; HRMS (ESI) m/z 302.0787 (M+H$^+$), calc. for $C_{17}H_{10}F_3NO$ 302.0789.

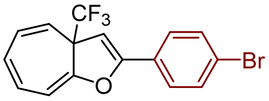

2c

2-(4-bromophenyl)-3a-(trifluoromethyl)-3a*H*-cyclohepta[b]furan (2c): white solid; Mp 104.4 – 105.9 °C; 33.7 mg, 95% yield; ^{1}H NMR (300 MHz, $CDCl_3$) δ 7.55 (s, 4H), 6.48 (ddd, *J* = 8.5, 6.2, 3.0 Hz, 4H), 5.81 (s, 1H), 5.28 (d, *J* = 10.2Hz, 1H); ^{13}C NMR (75 MHz, $CDCl_3$) δ 156.4, 145.8, 131.9, 129.0, 127.2, 126.2 (d, *J* = 286.8 Hz), 125.9, 125.6, 124.2, 112.4, 105.1, 99.9, 58.7 (q, *J* = 31.2 Hz); ^{19}F NMR (565 MHz, $CDCl_3$) δ -76.1; HRMS (ESI) m/z 354.9940 (M+H$^+$), calc. for $C_{16}H_{10}BrF_3O$ 354.9946.

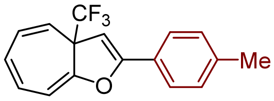

2d

2-(p-tolyl)-3a-(trifluoromethyl)-3a*H*-cyclohepta[*b*]furan (2d): white solid; Mp 110.8 – 111.7 °C; 28.4 mg, 98% yield; ^{1}H NMR (300 MHz, $CDCl_3$) δ 7.58 (d, *J* = 8.2 Hz, 2H), 7.23 (d, *J* = 8.1 Hz, 2H), 6.60 – 6.33 (m, 4H), 5.75 (s, 1H), 5.29 (d, *J* = 10.2 Hz, 1H), 2.40 (s, 3H); ^{13}C NMR (75 MHz, $CDCl_3$) δ 157.4, 146.1, 140.1, 129.3, 128.8, 126.3 (q, *J* = 286.7 Hz), 125.9, 125.7, 112.7, 104.7, 98.3, 77.4, 77.0, 76.6, 58.6 (q, *J* = 31.2 Hz), 24.4; ^{19}F NMR (565 MHz, $CDCl_3$) δ -76.2; HRMS (ESI) m/z 291.0991 (M+H$^+$), calc. for $C_{18}H_{22}NO_2$ 291.0996.

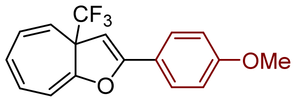
2e

2-(4-methoxyphenyl)-3a-(trifluoromethyl)-3a*H*-cyclohepta[*b*]furan(2e): yellow oil; 29.4mg, 96% yield; ^{1}H NMR (300 MHz, $CDCl_3$) δ 7.62 (d, *J* = 8.8 Hz, 2H), 6.93 (d, *J* = 8.8 Hz, 2H), 6.55 – 6.36 (m, 4H), 5.66 (s, 1H), 5.28 (d, *J* = 10.1 Hz, 1H), 3.85 (s, 3H); ^{13}C NMR (75 MHz, $CDCl_3$) δ 160.9, 157.1, 146.2, 128.8, 127.2, 126.4 (q, *J* = 286.6 Hz), 125.9, 125.4, 120.9, 114.0, 112.9, 104.7, 97.3(d, *J* = 2.1 Hz), 55.4; ^{19}F NMR (376 MHz, CD_2Cl_2) δ -76.4; HRMS (ESI) m/z 307.0941 (M+H$^+$), calc. for $C_{17}H_{13}F_3O_2$ 307.0940.

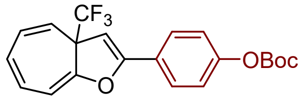
2f

tert-butyl (4-(3a-(trifluoromethyl)-3a*H*-cyclohepta[b]furan-2-yl)phenyl) carbonate (2f): white solid; Mp 70.0 – 71.2 °C; 36.5 mg, 93% yield; ^{1}H NMR (600 MHz, $CDCl_3$) δ 7.63 – 7.51 (m, 2H), 7.18 – 7.07 (m, 2H), 6.41 (d, *J* = 7.1 Hz, 1H), 6.38 – 6.34 (m, 2H), 6.31 (dd, *J* = 10.7, 6.9 Hz, 1H), 5.66 (s, 1H), 5.17 (d, *J* = 10.3 Hz, 1H), 1.47 (s, 9H); ^{13}C NMR (151 MHz, $CDCl_3$) δ 156.5, 156.5, 152.2, 152.2, 151.4, 151.4, 147.0, 146.0, 128.9, 126.9, 126.3 (q, *J* = 286.9 Hz), 125.9, 125.5, 121.5, 112.6, 105.0, 99.3, 83.9, 58.7 (q, *J* = 31.3 Hz), 27.7; ^{19}F NMR (565 MHz, $CDCl_3$) δ -76.1; HRMS (ESI) m/z 393.1308 (M+H$^+$), calc. for $C_{21}H_{19}F_3O_4$ 393.1312.

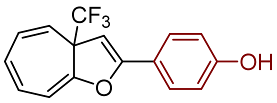
2g

4-(3a-(trifluoromethyl)-3a*H*-cyclohepta[b]furan-2-yl) phenol (2g): yellow oil; 25.4 mg, 87% yield; ^{1}H NMR (400 MHz, $CDCl_3$) δ 7.60 – 7.54 (m, 2H), 6.89 – 6.85 (m, 2H), 6.51 – 6.40 (m, 4H), 5.65 (s, 1H), 5.28 (d, *J* = 10.3 Hz, 1H); ^{13}C NMR (101 MHz, $CDCl_3$) δ 157.0, 146.1, 128.8, 127.4, 126.4 (q, *J* = 286.6 Hz), 125.8, 125.3, 121.1, 115.5, 112.9, 104.6, 97.3, 58.6 (q, *J* = 31.1 Hz); ^{19}F NMR (376 MHz, $CDCl_3$) δ -76.1; HRMS (ESI) m/z 293.0784 (M+H$^+$), calc. for $C_{16}H_{11}F_3O_2$ 293.0782.

2-(3,4-dimethoxyphenyl)-3a-(trifluoromethyl)-3a*H*-cyclohepta[*b*]furan (2h): white solid;

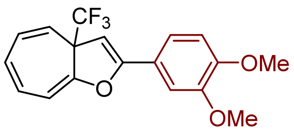
2h

Mp 94.7 – 95.5 °C; 33.3 mg, 99% yield; ^{1}H NMR (600 MHz, $CDCl_3$) δ 7.28 (dd, *J* = 8.4, 1.9 Hz, 1H), 7.15 (d, *J* = 1.9 Hz, 1H), 6.88 (d, *J* = 8.4 Hz, 1H), 6.51 (d, *J* = 7.1 Hz, 1H), 6.49 – 6.44 (m, 2H), 6.41 (dd, *J* = 10.8, 6.7 Hz, 1H), 5.67 (s, 1H), 5.28 (d, *J* = 10.4 Hz, 1H), 3.94 (s, 3H), 3.91 (s, 3H); ^{13}C NMR (151 MHz, $CDCl_3$) δ 157.1, 150.6, 149.0, 146.1, 128.8, 126.4 (q, *J* = 286.9 Hz), 125.9, 125.4, 121.1, 118.9, 112.9, 111.0, 108.5, 104.7, 97.6, 56.0, 58.7 (q, *J* = 31.0 Hz); ^{19}F NMR (565 MHz, $CDCl_3$) δ -76.1; HRMS (ESI) m/z 337.1046 (M+H$^+$), calc. for $C_{18}H_{15}F_3O_3$ 337.1045.

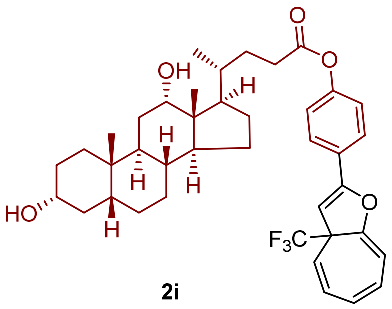

2i

4-(3a-(trifluoromethyl)-3a*H*-cyclohepta[b]furan-2-yl)phenyl (4*R*)-4-((3*R*,5*R*,8*R*,9*S*,10*S*,12*S*,13*R*,14*S*,17*R*)-3,12-dihydroxy-10,13-dimethylhexadecahydro-1*H*-cyclopenta[*a*]phenanthren-17-yl)pentanoate (2i): white solid; Mp 90.4 – 92.4 °C; 55.3 mg, 83% yield; ^{1}H NMR (600 MHz, $CDCl_3$) δ 7.71 – 7.63 (m, 2H), 7.15 – 7.10 (m, 2H), 6.51 – 6.36 (m, 4H), 5.75 (s, 1H), 5.26 (d, *J* = 10.3 Hz, 1H), 3.61 – 3.51 (m, 2H), 2.64 – 2.57 (m, 1H), 2.51 – 2.44 (m, 1H), 2.05 – 1.88 (m, 4H), 1.87 – 1.73 (m, 4H), 1.71 – 1.51 (m, 5H), 1.54 – 1.33 (m, 9H), 1.32 – 1.18 (m, 5H), 1.17 – 1.06 (m, 2H), 0.99 (d, *J* = 6.4 Hz, 4H), 0.93 (s, 3H), 0.69 (s, 3H); ^{13}C NMR (151 MHz, $CDCl_3$) δ 172.3, 156.4, 151.8, 145.8, 128.8, 126.8, 126.2 (q, *J* = 286.7 Hz), 125.8, 125.7, 125.4, 121.8, 112.5, 104.8, 99.2, 99.1, 71.2, 71.2, 67.8, 58.5 (q, *J* = 31.1 Hz), 55.7, 54.9, 43.7, 43.6, 42.4, 40.1, 39.2, 37.2, 37.0, 35.2, 34.8, 34.0, 33.8, 31.3, 30.8, 30.2, 28.6, 26.8, 25.5, 25.5, 24.9, 23.3, 21.1, 18.4, 14.0, 12.1; ^{19}F NMR (565 MHz, $CDCl_3$) δ -76.1; HRMS (ESI) m/z 667.3605 (M+H^{+}), calc. for $C_{40}H_{49}F_3O_5$ 667.3602.

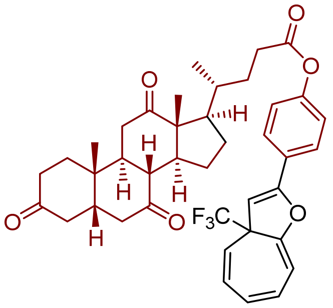

2j

4-(3a-(trifluoromethyl)-3aH-cyclohepta[*b*]furan-2-yl)phenyl (4*R*)-4-((5*S*,8*R*,9*S*,10*S*,13*R*,14*S*,17*R*)-10,13-dimethyl-3,7,12-trioxohexadecahydro-1*H*-cyclopenta[*a*]phenanthren-17-yl)pentanoate (2j): white solid; Mp 136.4 – 137.4 °C; 64.2 mg, 95% yield; ^{1}H NMR (400 MHz, $CDCl_3$) δ 7.68 (d, *J* = 8.7 Hz, 2H), 7.18 – 7.07 (m, 2H), 6.57 – 6.34 (m, 4H), 5.76 (d, *J* = 0.9 Hz, 1H), 5.27 (d, *J* = 10.3 Hz, 1H), 2.98 – 2.76 (m, 3H), 2.72 – 2.59 (m, 1H), 2.58 – 2.47 (m, 1H), 2.39 – 2.26 (m, 4H), 2.25 – 2.17 (m, 2H), 2.17 – 2.11 (m, 2H), 2.09 – 1.98 (m, 4H), 1.96 – 1.80 (m, 3H), 1.60 (d, *J* = 4.9 Hz, 4H), 1.39 (s, 3H), 1.08 (s, 3H), 0.91 (d, *J* = 6.6 Hz, 3H); ^{13}C NMR (101 MHz, $CDCl_3$) δ 211.9, 209.0, 208.6, 172.2, 156.5, 151.9, 145.9, 128.9, 126.8, 126.2 (d, *J* = 286.6 Hz), 125.8, 125.4, 121.8, 112.5, 104.9, 99.2, 58.6 (d, *J* = 31.2 Hz), 56.9, 51.7, 49.1, 48.9, 46.8, 45.6, 45.5, 44.9, 42.7, 38.6, 36.4, 36.0, 35.4, 35.2, 33.9, 31.5, 30.3, 27.6, 25.6, 25.1, 24.9, 21.8, 18.6, 11.8; ^{19}F NMR (376 MHz, $CDCl_3$) δ -76.1; HRMS (ESI) m/z 677.3085 (M+H^{+}), calc. for $C_{40}H_{43}F_3O6$ 667.3086.

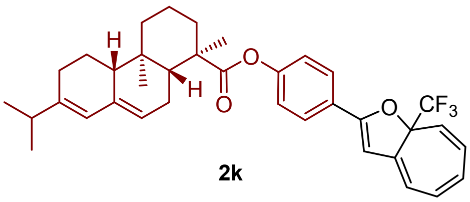

2k

4-(8a-(trifluoromethyl)-8a*H*-cyclohepta[b]furan-2-yl)phenyl (1*R*,4a*R*,4b*R*,10a*R*)-7-isopropyl-1,4a-dimethyl-1,2,3,4,4a,4b,5,6,10,10a-decahydrophenanthrene-1-carboxylate (2k): white solid; Mp 98.7 – 99.7 °C; 56.4 mg, 98% yield; ^{1}H NMR (400 MHz, $CDCl_3$) δ 7.69 (d, *J* = 8.6 Hz, 2H), 7.09 (d, *J* = 8.7 Hz, 2H), 6.56 – 6.41 (m, 4H), 5.89 – 5.71 (m, 2H), 5.45 (d, *J* = 1.9 Hz, 1H), 5.29 (d, *J* = 10.3 Hz, 1H), 2.37 – 2.17 (m, 3H), 2.04 – 1.94 (m, 3H), 1.90 – 1.81 (m, 2H), 1.69 (dd, *J* = 13.2, 8.1 Hz, 2H), 1.42 (d, *J* = 4.7 Hz, 3H), 1.37 – 1.18 (m, 5H), 1.05 (dd, *J* = 6.8, 3.8 Hz, 5H), 0.92 (d, *J* = 3.2 Hz, 3H); ^{13}C NMR (101 MHz, $CDCl_3$) δ 176.7, 156.5, 152.4, 145.9, 145.5, 135.6, 128.8, 126.7, 126.2 (q, *J* = 287.1 Hz), 125.8, 125.7, 125.4, 122.3, 121.8, 120.3, 112.5, 104.9, 99.1, 58.6 (q, *J* = 31.2 Hz). 50.9, 46.9, 45.0, 38.2, 37.0, 34.9, 34.6, 31.6, 27.4, 25.8, 22.6, 22.4, 21.4, 20.8, 18.1, 17.1, 14.1, 14.0; ^{19}F NMR (376 MHz, $CDCl_3$) δ -76.0; HRMS (ESI) m/z 577.2924 (M+H$^+$), calc. for $C_{36}H_{39}F_3O_3$ 577.2929.

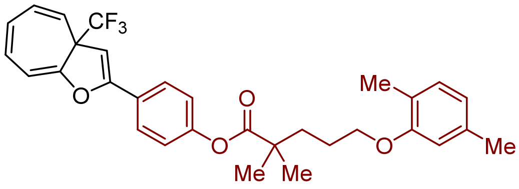

2l

4-(3a-(trifluoromethyl)-3a*H*-cyclohepta[b]furan-2-yl)phenyl-5-(2,5-dimethylphenoxy)-2,2-dimethylpentanoate (2l): yellow oil; 48.2 mg, 92% yield; ^{1}H NMR (400 MHz, $CDCl_3$) δ 7.69 (d, *J* = 8.7 Hz, 2H), 7.10 (d, *J* = 8.7 Hz, 2H), 7.02 (d, *J* = 7.5 Hz, 1H), 6.69 (d, *J* = 7.5 Hz, 1H), 6.64 (s, 1H), 6.54 – 6.41 (m, 4H), 5.78 (s, 1H), 5.29 (d, *J* = 10.3 Hz, 1H), 4.01 (t, *J* = 5.1 Hz, 2H), 2.32 (s, 3H), 2.19 (s, 3H), 1.91 (s, 4H), 1.40 (s, 6H); ^{13}C NMR (101 MHz, $CDCl_3$) δ 176.1, 156.8, 156.6, 152.3, 145.9, 136.5, 130.4, 128.9, 126.8, 126.2 (d, *J* = 286.8 Hz), 125.8, 125.5, 123.6, 121.9, 120.8, 112.6, 111.9, 104.9, 99.2, 67.7, 58.6 (d, *J* = 31.2 Hz), 42.5, 37.1, 25.2, 21.4, 15.8; ^{19}F NMR (376 MHz, $CDCl_3$) δ -76.1; HRMS (ESI) m/z 525.2247 (M+H$^+$), calc. for $C_{31}H_{31}F_3O_4$ 525.2248.

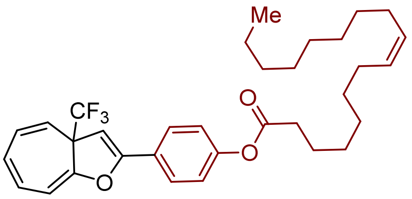

2m

4-(3a-(trifluoromethyl)-3a*H*-cyclohepta[b]furan-2-yl)phenyl (*Z*)-heptadec-8-enoate (2m): yellow oil; 46.6 mg, 86% yield; ^{1}H NMR (600 MHz, $CDCl_3$) δ 7.69 (d, *J* = 8.7 Hz, 2H), 7.15 (d, *J* = 8.7 Hz, 2H), 6.57 – 6.37 (m, 4H), 5.77 (s, 1H), 5.42 – 5.33 (m, 2H), 5.28 (d, *J* = 11.1 Hz, 1H), 2.57 (t, *J* = 7.5 Hz, 2H), 2.06 – 2.01 (m, 3H), 1.80 – 1.74 (m, 2H), 1.45 – 1.24 (m, 22H), 0.89 (t, *J* = 7.0 Hz, 3H); ^{13}C NMR

(151 MHz, $CDCl_3$) δ 171.9, 156.6, 151.9, 145.9, 130.0, 129.7, 128.9, 126.8, 126.2 (q, J = 286.9 Hz), 125.8, 125.5, 121.9, 112.6, 104.9, 99.2, 58.6 (q, J = 31.2 Hz), 34.4, 31.9, 29.8, 29.7, 29.5, 29.3, 29.1, 27.2, 27.1, 24.8, 22.7, 14.1; ^{19}F NMR (565 MHz, $CDCl_3$) δ -76.1; HRMS (ESI) m/z 543.3081 ($M+H^+$), calc. for $C_{33}H_{41}F_3O_3$ 543.3078.

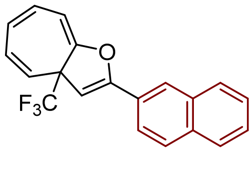

2n

2-(naphthalen-1-yl)-3a-(trifluoromethyl)-3a*H*-cyclohepta[b]furan (2n): white solid; Mp 105.3 – 106.9 °C; 29.7 mg, 91% yield; ^1H NMR (300 MHz, $CDCl_3$) δ 8.20 (s, 1H), 7.94 – 7.83 (m, 3H), 7.72 (dd, J = 8.6, 1.6 Hz, 1H), 7.58 – 7.51 (m, 2H), 6.62 – 6.43 (m, 4H), 5.94 (s, 1H), 5.34 (d, J = 10.4 Hz, 1H); ^{13}C NMR (75 MHz, $CDCl_3$) δ 133.8, 132.9, 128.9, 128.6, 128.4, 127.7, 126.3 (q, J = 286.7 Hz), 127.1, 126.7, 125.9, 125.4, 125.4, 125.3, 122.7, 112.6, 104.7, 99.8, 58.7 (q, J = 31.2 Hz); ^{19}F NMR (565 MHz, $CDCl_3$) δ -76.0; HRMS (ESI) m/z 327.0991 ($M+H^+$), calc. for $C_{20}H_{13}F_3O$ 329.0992.

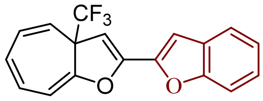

2o

2-(benzofuran-2-yl)-3a-(trifluoromethyl)-3a*H*-cyclohepta[*b*]furan(2o): white solid; Mp 94.3 – 95.0 °C; 27.5 mg, 87% yield; ^1H NMR (600 MHz, $CDCl_3$) δ 7.63 (d, J = 7.7 Hz, 1H), 7.52 (d, J = 8.3 Hz, 1H), 7.37 (t, J = 7.7 Hz, 1H), 7.28 (t, J = 7.5 Hz, 1H), 7.06 (s, 1H), 6.56 – 6.45 (m, 4H), 5.99 (s, 1H), 5.33 (d, J = 10.1 Hz, 1H); ^{13}C NMR (151 MHz, $CDCl_3$) δ 155.3, 149.2, 145.6, 145.4, 129.1, 127.7, 126.1 (q, J = 286.6 Hz), 125.9, 125.8, 125.7, 123.5, 121.9, 112.6, 111.4, 106.4, 105.3, 101.4, 58.4 (q, J = 31.7 Hz); ^{19}F NMR (565 MHz, $CDCl_3$) δ -75.9; HRMS (ESI) m/z 317.0784 ($M+H^+$), calc. for $C_{18}H_{11}F_3O_2$ 317.0783.

2-(benzo[b]thiophen-3-yl)-3a-(trifluoromethyl)-3a*H*-cyclohepta[*b*]furan (2p): white solid;

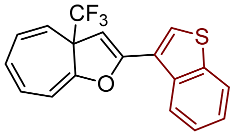

2p

29.5 mg, 89% yield; ^1H NMR (400 MHz, $CDCl_3$) δ 8.08 (d, J = 8.0 Hz, 1H), 7.89 (s, 1H), 7.51 – 7.39 (m, 2H), 6.57 – 6.32 (m, 4H), 5.88 (s, 1H), 5.33 (d, J = 10.2 Hz, 1H); ^{13}C NMR (151 MHz, $CDCl_3$) δ 152.8, 145.3, 140.5, 135.7, 129.0, 128.0, 126.3 (q, J = 286.7 Hz), 125.9, 125.5, 125.1, 125.0, 124.7, 123.0, 122.9, 112.7, 104.7, 100.2, 58.7 (q, J = 31.1 Hz); ^{19}F NMR (565 MHz, $CDCl_3$) δ -76.1; HRMS (ESI) m/z 333.0555 ($M+H^+$), calc. for $C_{18}H_{11}F_3OS$ 333.0558.

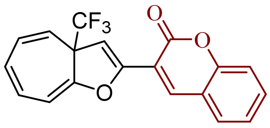

2q

3-(3a-(trifluoromethyl)-3a*H*-cyclohepta[*b*]furan-2-yl)-2H-chromen-2-one (2q): white solid; Mp 162.3 – 163.5 °C; 32.0 mg, 93% yield; ^{1}H NMR (600 MHz, $CDCl_3$) δ 7.63 (d, *J* = 7.7 Hz, 1H), 7.52 (d, *J* = 8.3 Hz, 1H), 7.37 (t, *J* = 7.7 Hz, 1H), 7.28 (t, *J* = 7.5 Hz, 1H), 7.06 (s, 1H), 6.56 – 6.45 (m, 4H), 5.99 (s, 1H), 5.33 (d, *J* = 10.1 Hz, 1H); ^{13}C NMR (151 MHz, $CDCl_3$) δ 155.3, 149.2, 145.6, 145.4, 129.1, 127.7, 126.1 (q, *J* = 286.6 Hz), 125.9, 125.8, 125.7, 123.5, 121.9, 112.6, 111.4, 106.4, 105.3, 101.4, 58.43 (q, *J* = 31.7 Hz), 29.7; ^{19}F NMR (565 MHz, $CDCl_3$) δ -75.9; HRMS (ESI) m/z 327.0991 (M+H$^+$), calc. for $C_{19}H_{11}F_3O_3$ 327.0992.

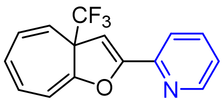

2r

2-(3a-(trifluoromethyl)-3a*H*-cyclohepta[*b*]furan-2-yl)pyridine (2r): yellow oil; 23.5 mg, 85% yield; ^{1}H NMR (400 MHz, $CDCl_3$) δ 8.64 (d, *J* = 4.2 Hz, 1H), 7.74 (td, *J* = 7.7, 1.7 Hz, 1H), 7.65 (d, *J* = 7.9 Hz, 1H), 7.29 – 7.23 (m, 1H), 6.54 (d, *J* = 6.9 Hz, 1H), 6.50 – 6.36 (m, 3H), 6.27 (s, 1H), 5.31 (d, *J* = 10.4 Hz, 1H); ^{13}C NMR (101 MHz, $CDCl_3$) δ 156.6, 149.9, 147.3, 146.0, 136.8, 129.0, 127.6, 125.8 (d, *J* = 12.4 Hz), 124.3, 120.6, 112.3, 105.4, 103.2, 58.6 (q, *J* = 31.4 Hz); ^{19}F NMR (376 MHz, $CDCl_3$) δ -75.8; HRMS (ESI) m/z 278.0787 (M+H$^+$), calc. for $C_{15}H_{10}F_3NO$ 278.0784.

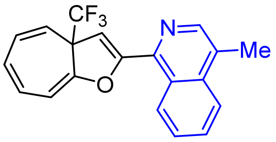

2s

4-methyl-1-(3a-(trifluoromethyl)-3a*H*-cyclohepta[*b*]furan-2-yl)isoquinoline (2s): black solid; 27.0 mg, 79% yield; ^{1}H NMR (600 MHz, CD_2Cl_2) δ 9.23 (d, *J* = 5.5 Hz, 1H), 9.16 (d, *J* = 8.8 Hz, 1H), 8.38 – 8.34 (m, 2H), 8.23 – 8.20 (m, 1H), 7.34 (d, *J* = 7.0 Hz, 1H), 7.30 – 7.19 (m, 3H), 6.90 (s, 1H), 6.13 (d, *J* = 10.2 Hz, 1H), 6.03 (t, *J* = 1.1 Hz, 2H), 3.26 (s, 3H); ^{13}C NMR (151 MHz, CD_2Cl_2) δ 157.5, 147.3, 146.2, 142.3, 141.1, 137.3, 130.5, 129.2, 126.4 (d, *J* = 287.2 Hz), 126.1, 125.9, 125.8, 125.0, 121.8, 112.1, 106.7, 105.5, 58.3 (d, *J* = 31.4 Hz), 21.7; ^{19}F NMR (565 MHz, CD_2Cl_2) δ -75.6; HRMS (ESI) m/z 342.1100 (M+H$^+$), calc. for $C_{20}H_{14}F_3NO$ 342.1099.

2-(3a-(trifluoromethyl)-3a*H*-cyclohepta[*b*]furan-2-yl)benzo[*h*]quinoline (2t): white solid;

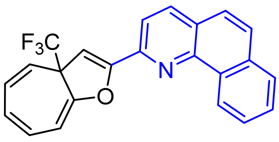

2t

Mp 158.9 – 159.2 °C; 32.8 mg, 87% yield; ^{1}H NMR (300 MHz, $CDCl_3$) δ 9.39 (dd, *J* = 7.7, 1.7 Hz, 1H), 8.22 (d, *J* = 8.3 Hz, 1H), 7.97 – 7.63 (m, 6H), 6.78 – 6.31 (m, 5H), 5.43 (d, *J* = 10.3 Hz, 1H); ^{13}C

NMR (151 MHz, CD_2Cl_2) δ 157.4, 146.4, 146.2, 145.7, 134.0, 131.3, 129.2, 128.7, 128.7, 128.0, 127.3, 126.8 (q, J = 286.1 Hz), 125.9, 125.8, 125.1, 124.5, 118.6, 112.4, 105.5, 103.4, 77.0, 58.8 (q, J = 31.1 Hz); ^{19}F NMR (565 MHz, CD_2Cl_2) δ -76.0; HRMS (ESI) m/z 378.1100 (M+H$^+$), calc. for $C_{23}H_{14}F_3NO$ 378.1104.

1-(3a-(trifluoromethyl)-3a*H*-cyclohepta[*b*]furan-2-yl) benzo[*h*]isoquinoline (2u): white solid; 31.3 mg, 83% yield; ^{1}H NMR (300 MHz, CD_2Cl_2) δ 8.76 (d, J = 8.4 Hz, 1H), 8.68 (d, J = 7.5 Hz, 1H), 8.66 – 8.57 (m, 1H), 8.25 (dd, J = 8.0, 1.7 Hz, 1H), 7.97 (t, J = 7.0 Hz, 1H), 7.87 – 7.72 (m, 3H), 6.88 – 6.41 (m, 4H), 6.27 (d, J = 1.1 Hz, 1H), 5.52 (d, J = 10.0 Hz, 1H), 5.39 – 5.29 (m, 1H); ^{13}C NMR (75 MHz, CD_2Cl_2) δ 156.9, 148.7, 146.0, 143.4, 133.3, 131.2, 130.6, 129.3, 129.2, 128.2, 127.9, 127.2, 125.9, 124.4, 122.4, 122.2, 112.0, 107.4, 105.7, 58.5 (d, J = 31.4 Hz); ^{19}F NMR (565 MHz, CD_2Cl_2) δ -76.0; HRMS (ESI) m/z 378.1100 (M+H$^+$), calc. for $C_{23}H_{14}F_3NO$ 378.1104.

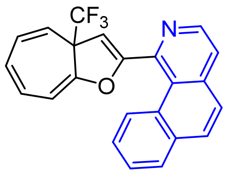

2u

2-(3a-(trifluoromethyl)-3a*H*-cyclohepta[*b*]furan-2-yl)thiazole(2v): white solid; 24.1 mg, 85% yield; ^{1}H NMR (600 MHz, $CDCl_3$) δ 7.94 (d, J = 3.1 Hz, 1H), 7.47 (d, J = 3.1 Hz, 1H), 6.56 (d, J = 6.6 Hz, 1H), 6.52 – 6.45 (m, 3H), 6.20 (s, 1H), 5.32 (d, J = 10.1 Hz, 1H); ^{13}C NMR (151 MHz, $CDCl_3$) δ 155.9, 151.5, 145.1, 144.4, 129.2, 126.0, 125.9 (q, J = 287.0 Hz), 125.7, 120.8, 112.3, 105.8, 102.6, 58.6 (q, J = 31.6 Hz); ^{19}F NMR (565 MHz, $CDCl_3$) δ -75.8; HRMS (ESI) m/z 284.0451 (M+H$^+$), calc. for $C_{13}H_8F_3NOS$ 284.0451.

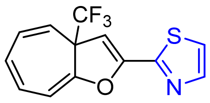

2v

2-phenethyl-3a-(trifluoromethyl)-3a*H*-cyclohepta[*b*]furan(2w): yellow oil; 22.8 mg, 75% yield; ^{1}H NMR (300 MHz, $CDCl_3$) δ 7.39 – 7.28 (m, 3H), 7.25 (t, J = 5.5 Hz, 2H), 6.52 – 6.39 (m, 4H), 5.20 (d, J = 8.5 Hz, 2H), 3.00 – 2.92 (m, 2H), 2.72 – 2.63 (m, 2H); ^{13}C NMR (75 MHz, $CDCl_3$) δ 159.9, 146.5, 140.4, 128.7, 128.5, 128.4, 126.3, 126.1 (q, J = 12.6 Hz), 125.9, 125.2, 112.5, 104.2, 101.0, 58.1 (q, J = 31.0 Hz); 32.5, 29.5; ^{19}F NMR (565 MHz, $CDCl_3$) δ -76.4; HRMS (ESI) m/z 305.1148 (M+H$^+$), calc. for $C_{18}H_{15}F_3O$ 305.1148.

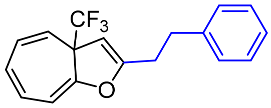

2w

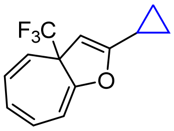
2x

2-cyclopropyl-3a-(trifluoromethyl)-3a*H*-cyclohepta[*b*]furan (2x): yellow oil; 22.8 mg, 75% yield; ^{1}H NMR (600 MHz, $CDCl_3$) δ 6.43 – 6.38 (m, 2H), 6.37 – 6.33 (m, 1H), 6.30 (dt, *J* = 7.2, 1.0 Hz, 1H), 5.17 (d, *J* = 10.0 Hz, 1H), 5.14 (s, 1H), 1.67 – 1.53 (m, 1H), 0.91 – 0.76 (m, 4H); ^{13}C NMR (151 MHz, $CDCl_3$) δ 161.5, 146.4, 128.5, 126.3 (q, *J* = 286.4 Hz), 125.7, 125.1, 112.9, 104.1, 98.3, 98., 98.3, 58.1 (q, *J* = 31.1 Hz), 8.3, 6.1, 5.8; ^{19}F NMR (565 MHz, $CDCl_3$) δ -76.6; HRMS (ESI) m/z 241.0835 (M+H$^+$), calc. for $C_{13}H_{11}F_3O$ 241.0836.

2-((1r,3R,5S,7s)-adamantan-1-yl)-3a-(trifluoromethyl)-3a*H*-cyclohepta[*b*]furan(2y):

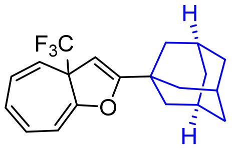
2y

white solid; Mp 116.7 – 117.1 °C; 24.4 mg, 73% yield; ^{1}H NMR (300 MHz, $CDCl_3$) δ 6.46 – 6.32 (m, 4H), 5.18 (d, *J* = 10.2 Hz, 1H), 5.04 (s, 1H), 2.04 (s, 3H), 1.84 (s, 6H), 1.77 (d, *J* = 14.9 Hz, 6H); ^{13}C NMR (75 MHz, $CDCl_3$) δ 168.6, 146.9, 128.5, 126.4 (d, *J* = 286.5 Hz), 125.9, 124.7, 112.5, 103.9, 97.1, 57.9 (q, *J* = 30.6 Hz), 39.5, 36.6, 34.1, 27.8; ^{19}F NMR (565 MHz, $CDCl_3$) δ -76.70; HRMS (ESI) m/z 335.1617 (M+H$^+$), calc. for $C_{20}H_{21}F_3O$ 335.1621.

6,8-dimethyl-2-phenyl-3a-(trifluoromethyl)-3a*H*-cyclohepta[*b*]furan(2za): white solid; Mp

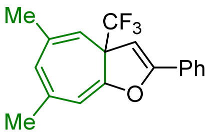
2za

98.3 – 99.7 °C; 22.2 mg, 73% yield; ^{1}H NMR (400 MHz, $CDCl_3$) δ 7.66 (dd, *J* = 7.3, 2.3 Hz, 2H), 7.45 – 7.36 (m, 3H), 6.25 (s, 1H), 6.14 (s, 1H), 5.71 (s, 1H), 5.05 (s, 1H), 2.10 (s, 3H), 2.01 (s, 3H); ^{13}C NMR (101 MHz, $CDCl_3$) δ 156.9, 146.9, 136.9, 133.8, 129.8, 128.5, 128.4, 126.5 (q, *J* = 287.0 Hz), 126.2, 125.6, 109.1, 107.0, 99.6, 57.2 (q, *J* = 30.7 Hz), 25.3, 24.3; ^{19}F NMR (376 MHz, $CDCl_3$) δ -75.8; HRMS (ESI) m/z 305.1148 (M+H$^+$), calc. for $C_{18}H_{15}F_3O$ 305.1148.

2-phenyl-3a-(trifluoromethyl)-3a*H*-benzo[5,6]cyclohepta[1,2-*b*]furan (2zb): yellow solid;

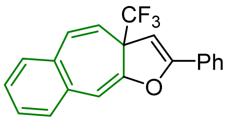
2zb

Mp 117.6 – 119.6 °C; 27.7 mg, 85% yield; ^{1}H NMR (600 MHz, $CDCl_3$) δ 8.20 (s, 1H), 7.91 (dt, *J* = 6.8, 3.5 Hz, 1H), 7.86 (dd, *J* = 10.4, 6.1 Hz, 2H), 7.72 (dd, *J* = 8.6, 1.5 Hz, 1H), 7.54 (dd, *J* = 6.2, 3.2 Hz, 2H), 6.61 (d, *J* = 7.2 Hz, 1H), 5.94 (s, 1H), 5.35 (d, *J* = 10.3 Hz, 1H); ^{13}C NMR (151 MHz, $CDCl_3$) δ 157.3, 146.0, 133.9, 132.9, 128.9, 128.6, 128.4, 127.8, 127.1, 126.7, 126.3 (q, *J* = 287.0 Hz), 125.9, 125.4, 125.4, 125.3, 122.8, 112.6, 104.9, 99.8, 58.7 (q, *J* = 31.2 Hz); ^{19}F NMR (565 MHz, $CDCl_3$) δ -75.9; HRMS (ESI) m/z 272.1443 (M+H$^+$), calc. for $C_{17}H_{19}FNO$ 272.1445.

2-phenyl-3a-(trifluoromethyl)-3a*H*-naphtho[2',3':5,6]cyclohepta[1,2-b]furan (2zc):

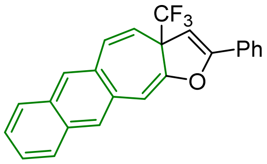

2zc

yellow solid; Mp 98.8 – 100.2 °C; 32.7 mg, 87% yield; ^{1}H NMR (300 MHz, $CDCl_3$) δ 7.94 (s, 1H), 7.89 – 7.77 (m, 2H), 7.75 (s, 1H), 7.67 – 7.58 (m, 2H), 7.53 – 7.45 (m, 2H), 7.44 – 7.34 (m, 3H), 6.88 (d, J = 9.9 Hz, 1H), 6.46 (dd, J = 9.9, 1.3 Hz, 1H), 6.04 (s, 1H), 2.89 (s, 1H); ^{13}C NMR (151 MHz, $CDCl_3$) δ 157.7, 133.3, 132.6, 130.0, 129.4, 129.3, 129.1, 128.5, 128.0, 127.6, 127.4, 127.2, 126.5, 126.1, 125.1, 123.2 (q, J = 275.1 Hz), 119.2, 99.0, 71.7, 33.7 (q, J = 36.3 Hz), 32.0; ^{19}F NMR (565 MHz, $CDCl_3$) δ -62.1; HRMS (ESI) m/z 377.1148 ($M+H^+$), calc. for $C_{24}H_{15}F_3O$ 377.1150.

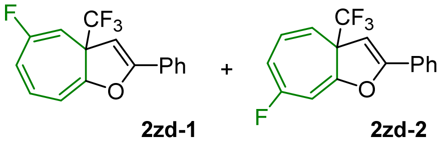

2zd-1 **2zd-2**

6-fluoro-2-phenyl-3a-(trifluoromethyl)-3a*H*-cyclohepta[b]furan (2zd-1) and 7-fluoro-2-phenyl-3a-(trifluoromethyl)-3a*H*-cyclohepta[b]furan (2zd-2): white solid; Mp 117.6 – 119.6 °C; 10.3 mg, 35% yield; **2zd-1:** ^{1}H NMR (600 MHz, $CDCl_3$) δ 7.68 (s, 2H), 7.48 – 7.44 (m, 3H), 6.54 (td, J = 7.6, 3.7 Hz, 1H), 6.42 (d, J = 5.3 Hz, 1H), 6.31 (td, J = 11.6, 2.0 Hz, 1H), 5.71 (s, 1H), 5.11 (d, J = 17.8 Hz, 1H); **2zd-2:** ^{1}H NMR (600 MHz, $CDCl_3$) δ 7.69 – 7.66 (m, 2H), 7.44 – 7.42 (m, 3H), 6.49 (dt, J = 9.0, 1.6 Hz, 1H), 6.42 – 6.38 (m, 1H), 6.23 – 6.13 (m, 1H), 5.82 (d, J = 1.1 Hz, 1H), 5.19 (d, J = 10.3 Hz, 1H).^{13}C NMR (151 MHz, $CDCl_3$) δ 160.7, 159.8, 158.2, 157.6, 157.5, 149.2, 147.7, 147.5, 130.2, 128.8, 128.7, 128.7, 127.8, 127.7, 126.7, 126.1, 126.0, 125.7, 125.6, 124.8, 118.6, 118.4, 109.7, 106.4, 106.2, 103.9, 100.3, 100.1, 99.3, 98.6, 94.5, 94.3, 58.8 (q, J = 31.1 Hz); ^{19}F NMR (565 MHz, $CDCl_3$) δ -76.1, -77.4; HRMS (ESI) m/z 295.0741 ($M+H^+$), calc. for $C_{16}H_{10}F_4O$ 295.0744.

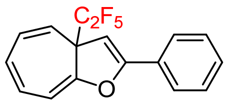

2ze

3a-(perfluoroethyl)-2-phenyl-3a*H*-cyclohepta[*b*]furan(2ze): white solid; Mp 68.2 – 70.0 °C 27.7 mg, 92% yield; ^{1}H NMR (600 MHz, $CDCl_3$) δ 7.72 – 7.64 (m, 2H), 7.48 – 7.37 (m, 3H), 6.59 (d, J = 7.2 Hz, 1H), 6.49 (dd, J = 16.8, 9.9 Hz, 2H), 6.44 (dd, J = 11.0, 6.6 Hz, 1H), 5.78 (s, 1H), 5.27 (d, J = 10.2 Hz, 1H); 13C NMR (151 MHz, CDCl3) δ 157.0, 145.2, 130.0, 128.8, 128.6, 128.3, 126.1, 125.7, 121.3 (qt, J = 290.5, 35.1 Hz), 111.7, 114.30 (td, J = 234.7, 31.2 Hz), 105.7, 99.0, 57.8 (t, J = 24.2 Hz); ^{19}F NMR (565 MHz, $CDCl_3$) δ -79.09, -119.66 – -121.66 (m, 2F); HRMS (ESI) m/z 327.0803 ($M+H^+$), calc. for $C_{17}H_{11}F_5O$ 327.0804.

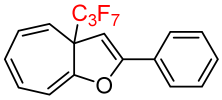

2zf

3a-(perfluoropropyl)-2-phenyl-3a*H*-cyclohepta[b]furan (3zf): white solid; Mp 67.2 – 67.6 °C; 27.7 mg, 98% yield; ^{1}H NMR (400 MHz, $CDCl_3$) δ 7.72 – 7.67 (m, 2H), 7.47 – 7.38 (m, 3H), 6.59 (d, *J* = 7.2 Hz, 1H), 6.55 – 6.40 (m, 3H), 5.87 – 5.79 (m, 1H), 5.30 (dd, *J* = 10.2, 1.7 Hz, 1H); 13C NMR (101 MHz, CDCl3) δ 157.0, 145.1, 130.0, 128.7, 128.6, 128.3, 126.1, 125.6, 118.4 (qt, *J* = 290.5, 35.1 Hz), 113.1 (tq, *J* = 136.4, 6.04 Hz), 108.3 (tq, *J* = 167.7, 39.5 Hz), 105.9, 98.9 (q, *J* = 3.2 Hz), 59.0 (t, *J* = 24.8 Hz); ^{19}F NMR (376 MHz, $CDCl_3$) δ -81.2, -116.7 – -117.8 (m, 2F), -121.3 – -125.3 (m, 2F); HRMS (ESI) m/z 377.0771 ($M+H^+$), calc. for $C_{18}H_{11}F_7O$ 377.0771.

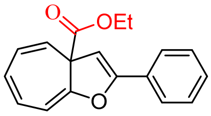

2zg

Ethyl-2-phenyl-3a*H*-cyclohepta[*b*]furan-3a-carboxylate (2zg): yellow oil; 20.4 mg, 73% yield; ^{1}H NMR (600 MHz, $CDCl_3$) δ 7.68 – 7.65 (m, 2H), 7.41 – 7.36 (m, 3H), 6.57 – 6.49 (m, 1H), 6.41 – 6.31 (m, 3H), 5.91 (d, *J* = 1.1 Hz, 1H), 5.45 – 5.39 (m, 1H), 4.07 – 3.96 (m, 2H), 1.13 (t, *J* = 7.1 Hz, 3H); ^{13}C NMR (151 MHz, $CDCl_3$) δ 170.0, 156.6, 151.7, 129.6, 128.5, 126.8, 125.6, 124.6, 116.8, 102.5, 101.4, 61.4, 59.9, 14.1; HRMS (ESI) m/z 267.1016 ($M+H^+$), calc. for $C_{18}H_{16}O_3$ 267.1016; HRMS (ESI) m/z 377.0771 ($M+H^+$), calc. for $C_{18}H_{11}F_7O$ 377.0771.

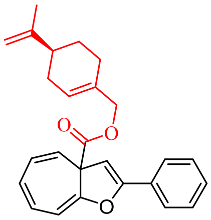

2zh

((*S*)-4-(prop-1-en-2-yl)cyclohex-1-en-1-yl)methyl-2-phenyl-3a*H*-cyclohepta[*b*]furan-3a-carboxylate (2zh): yellow oil; 25.1 mg, 65% yield; ^{1}H NMR (400 MHz, $CDCl_3$) δ 7.68 – 7.64 (m, 2H), 7.39 (qd, *J* = 3.8, 1.9 Hz, 3H), 6.56 – 6.49 (m, 1H), 6.41 – 6.31 (m, 3H), 5.92 (d, *J* = 1.1 Hz, 1H), 5.65 (d, *J* = 4.1 Hz, 1H), 5.48 – 5.39 (m, 1H), 4.71 (dq, *J* = 9.7, 1.3 Hz, 2H), 4.36 (d, *J* = 4.2 Hz, 2H), 2.18 – 2.05 (m, 3H), 1.97 – 1.90 (m, 3H), 1.73 (t, *J* = 1.1 Hz, 3H), 1.59 (s, 1H), 1.43 (s, 1H), 1.34 (s, 1H), 1.29 (s, 2H), 1.26 (d, *J* = 2.1 Hz, 1H); ^{13}C NMR (151 MHz, CD_2Cl_2) δ 169.7, 151.8, 149.9, 132.6, 129.8, 128.7, 126.8, 125.5, 124.7, 117.0, 108.5, 103.0, 101.4, 69.0, 60.2, 40.9, 30.5, 27.4, 26.1, 25.2, 20.6; HRMS (ESI) m/z 387.1955 ($M+H^+$), calc. for $C_{26}H_{26}O_3$ 387.1956.

2-(3,4-dimethoxyphenyl)-3a*H*-cyclohepta[*b*]furan-3a-carbonitrile (3zi): white solid; Mp

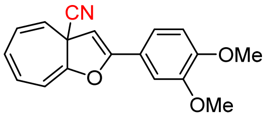

2zi

147.0 – 148.1 °C; 23.4 mg, 80% yield; ^{1}H NMR (400 MHz, $CDCl_3$) δ 7.41 (dd, *J* = 8.4, 1.9 Hz, 1H), 7.37 – 7.31 (m, 3H), 7.24 – 7.17 (m, 4H), 6.75 (d, *J* = 8.4 Hz, 1H), 3.84 (s, 3H), 3.81 (s, 3H); ^{13}C NMR (151 MHz, $CDCl_3$) δ 189.8, 154.5, 149.4, 139.7, 131.2, 130.3, 128.8, 128.4, 128.4, 124.8, 122.1,

118.0, 110.2, 110.0, 56.1, 55.9; HRMS (ESI) m/z 294.1125 (M+H$^+$), calc. for $C_{18}H_{15}NO_3$ 294.1125.

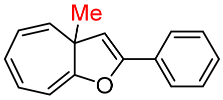

2zj

3a-methyl-2-phenyl-3a*H*-cyclohepta[*b*]furan (3zj): grey solid; Mp 39.0 – 40.6 ºC; 19.1 mg, 86% yield; ^{1}H NMR (300 MHz, $CDCl_3$) δ 7.66 (d, *J* = 6.7 Hz, 2H), 7.44 – 7.33 (m, 3H), 6.46 (dd, *J* = 10.9, 6.9 Hz, 1H), 6.32 (dd, *J* = 10.8, 6.4 Hz, 1H), 6.26 – 6.14 (m, 2H), 5.78 (s, 1H), 5.39 (d, *J* = 9.8 Hz, 1H), 0.88 (s, 3H); ^{13}C NMR (75 MHz, $CDCl_3$) δ 156.6, 153.1, 129.4, 128.9, 128.4, 125.9, 125.1, 124.1, 123.8, 120.4, 108.0, 99.0, 49.6, 18.7; HRMS (ESI) m/z 223.1118 (M+H$^+$), calc. for $C_{16}H_{14}O$ 223.1118.

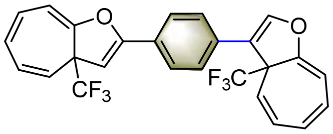

2zk

3a-(trifluoromethyl)-3-(4-(3a-(trifluoromethyl)-3aH-cyclohepta[b]furan-2-yl)phenyl)-3a*H*-cyclohepta[*b*]furan (2zk): yellow solid; 35.6 mg, 75% yield; ^{1}H NMR (400 MHz, $CDCl_3$) δ 7.72 (s, 4H), 6.57 – 6.37 (m, 8H), 5.87 (s, 2H), 5.29 (d, *J* = 10.3 Hz, 2H); ^{13}C NMR (101 MHz, $CDCl_3$) δ 156.5, 145.8, 129.5, 129.0, 127.6, 126.2 (q, *J* = 286.6 Hz), 125.9, 125.8, 125.6, 124.8, 112.4, 105.1, 100.4, 58.6 (q, *J* = 31.2 Hz); ^{19}F NMR (376 MHz, $CDCl_3$) δ -76.0; HRMS (ESI) m/z 474.1127 (M+H$^+$), calc. for $C_{26}H_{16}F_6O_2$ 474.1123.

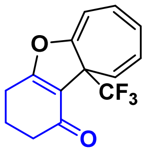

2zl

10a-(trifluoromethyl)-2,3,4,10a-tetrahydro-1*H*-cyclohepta[*b*]benzofuran-1-one (2zl): white solid; Mp 78.5 – 80.1 ºC; 23.3 mg, 87% yield; ^{1}H NMR (400 MHz, $CDCl_3$) δ 6.50 – 6.43 (m, 3H), 6.40 – 6.33 (m, 1H), 5.73 (dd, *J* = 8.6, 6.0 Hz, 1H), 2.76 – 2.68 (m, 1H), 2.63 – 2.50 (m, 2H), 2.42 (ddd, *J* = 16.6, 8.6, 5.7 Hz, 1H), 2.20 – 2.12 (m, 2H); ^{13}C NMR (75 MHz, $CDCl_3$) δ 192.3, 175.6, 145.4, 129.1, 127.5, 126.0 (q, *J* = 288.4 Hz), 124.5, 114.52, 114.2, 107.2, 67.9, 56.0 (q, *J* = 32.3 Hz), 37.4, 25.6, 23.5, 21.3; ^{19}F NMR (376 MHz, $CDCl_3$) δ -74.8; HRMS (ESI) m/z 269.0784 (M+H$^+$), calc. for $C_{14}H_{11}F_3O_2$ 269.0783.

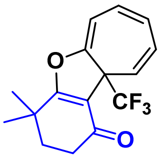

2zm

4,4-dimethyl-10a-(trifluoromethyl)-2,3,4,10a-tetrahydro-1*H*-cyclohepta[*b*]benzofuran-1-one (2zm): white solid; Mp 83.4 – 85.0 ºC; 27.5 mg, 93% yield; ^{1}H NMR (600 MHz, $CDCl_3$) δ 6.50 – 6.43 (m, 3H), 6.37 (tt, *J* = 10.0, 3.2 Hz, 1H), 5.78 – 5.59 (m, 1H), 2.51 (dd, *J* = 42.1, 17.9 Hz, 2H), 2.43

– 2.25 (m, 2H), 1.17 (s, 3H), 1.14 (s, 3H); ^{13}C NMR (101 MHz, $CDCl_3$) δ 191.7, 174.6, 145.7, 129.1, 127.5, 126.07 (q, *J* = 288.1 Hz), 124.5, 114.5, 113.2, 107.3, 56.0 (q, *J* = 32.2 Hz), 51.7, 37.2, 34.1, 29.0, 27.7; ^{19}F NMR (376 MHz, $CDCl_3$) δ -74.7; HRMS (ESI) m/z 277.0714 ($M+H^+$), calc. for $C_{16}H_{15}F_3O_2$ 277.0720.

6b-(trifluoromethyl)-5,6b-dihydro-6*H*-cyclohepta[*b*]naphtho[2,1-*d*]furan (2zn): white

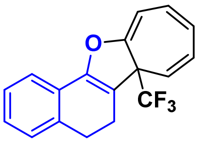

2zn

solid; Mp 88.7 – 89.2 °C; 28.1 mg, 93% yield; ^{1}H NMR (400 MHz, $CDCl_3$) δ 7.46 – 7.36 (m, 1H), 7.26 – 7.20 (m, 3H), 6.59 – 6.35 (m, 4H), 5.27 (d, *J* = 10.1 Hz, 1H), 3.10 – 2.97 (m, 2H), 2.72 – 2.60 (m, 2H); ^{13}C NMR (101 MHz, $CDCl_3$) δ 151.9, 146.8, 136.2, 129.0, 128.9, 127.7, 126.6, 126.37 (q, *J* = 288.5 Hz), 126.0 (q, *J* = 9.3 Hz), 125.4, 121.3, 111.4, 111.0, 105.1, 58.8 (q, *J* = 30.6 Hz), 28.6, 19.4; ^{19}F NMR (376 MHz, $CDCl_3$) δ -74.3; HRMS (ESI) m/z 303.0991 ($M+H^+$), calc. for $C_{18}H_{13}F_3O$ 303.0995.

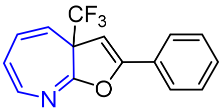

4a

2-phenyl-3a-(trifluoromethyl)-3a*H*-furo[2,3-*b*]azepine (4a): white solid; 11.9 mg, 43% yield; ^{1}H NMR (400 MHz, $CDCl_3$) δ 8.64 (d, *J* = 4.2 Hz, 1H), 7.74 (td, *J* = 7.7, 1.8 Hz, 1H), 7.65 (d, *J* = 7.9 Hz, 1H), 7.29 – 7.23 (m, 1H), 6.54 (d, *J* = 6.9 Hz, 1H), 6.50 – 6.36 (m, 3H), 6.27 (s, 1H), 5.31 (d, *J* = 10.4 Hz, 1H); ^{13}C NMR (151 MHz, $CDCl_3$) δ 156.2, 147.5, 134.9, 130.5, 130.3, 128.8, 127.4, 125.6, 124.8 (q, *J* = 286.1 Hz), 115.8, 110.4, 100.1, 59.6 (d, *J* = 32.5 Hz); ^{19}F NMR (565 MHz, $CDCl_3$) δ -71.2; HRMS (ESI) m/z 278.0787 ($M+H^+$), calc. for $C_{15}H_{10}F_3NO$ 278.0786.

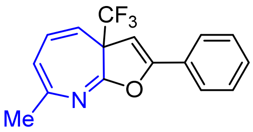

4b

7-methyl-2-phenyl-3a-(trifluoromethyl)-3a*H*-furo[2,3-*b*]azepine (4b): white solid; Mp 72.9 – 74.6 °C; 15.1 mg, 52% yield; ^{1}H NMR (600 MHz, $CDCl_3$) δ 7.74 – 7.67 (m, 2H), 7.47 – 7.38 (m, 3H), 6.46 (dd, *J* = 9.8, 7.3 Hz, 1H), 6.01 (d, *J* = 7.2 Hz, 1H), 5.97 (s, 1H), 5.07 (d, *J* = 9.9 Hz, 1H), 2.21 (s, 3H); ^{13}C NMR (151 MHz, $CDCl_3$) δ 156.0, 145.8, 144.1, 130.4, 130.3, 128.7, 127.5, 125.5, 124.8 (q, *J* = 285.6 Hz), 112.5, 107.7, 100.1, 58.9 (q, *J* = 32.6 Hz), 24.6; ^{19}F NMR (565 MHz, $CDCl_3$) δ -72.0; HRMS (ESI) m/z 292.0944 ($M+H^+$), calc. for $C_{16}H_{12}F_3NO$ 292.0946.

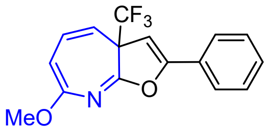

4c

7-methoxy-2-phenyl-3a-(trifluoromethyl)-3a*H*-furo[2,3-*b*] azepine (4c): white solid; Mp 83.6 – 84.6 °C;15.1 mg, 52% yield; ^{1}H NMR (400 MHz, $CDCl_3$) δ 7.83 (d, *J* = 7.2 Hz, 2H), 7.46 (t, *J* = 7.9 Hz, 2H), 7.34 (t, *J* = 7.7 Hz, 2H), 7.07 (d, *J* = 7.4 Hz, 1H), 6.97 (d, *J* = 1.2 Hz, 1H), 6.52 (d, *J* = 8.3 Hz, 1H), 3.19 (s, 3H); ^{13}C NMR (151 MHz, CD_2Cl_2) δ 155.5, 145.0, 143.8, 130.0, 129.9, 128.3, 127.2, 125.0, 124.6 (q, *J* = 285.6 Hz), 111.9, 107.3, 99.8, 58.5 (q, *J* = 32.2 Hz), 23.9; ^{19}F NMR (565 MHz, CD_2Cl_2) δ -72.0; HRMS (ESI) m/z 308.0893 (M+H$^+$), calc. for $C_{16}H_{12}F_3NO_2$ 308.0894.

2-phenyl-3a-(trifluoromethyl)-3a*H*-benzo[*b*]furo[2,3-e][1,4]thiazine (4d): white solid; Mp 91.3 – 93.2 °C; 25.3 mg, 76% yield; ^{1}H NMR (400 MHz, $CDCl_3$) δ 7.81 – 7.71 (m, 2H), 7.48 (dd, *J* = 5.3, 2.0 Hz, 3H), 7.44 (dd, *J* = 7.9, 1.4 Hz, 1H), 7.39 (dd, *J* = 7.7, 1.4 Hz, 1H), 7.30 (td, *J* = 7.7, 1.5 Hz, 1H), 7.18 (td, *J* = 7.6, 1.4 Hz, 1H), 5.81 (s, 1H); ^{13}C NMR (101 MHz, $CDCl_3$) δ 159.1, 154.6, 140.0, 130.2, 127.9, 126.7, 126.0, 125.8, 125.1, 124.9, 122.8 (q, *J* = 285.9 Hz), 116.0, 93.3, 48.3 (d, *J* = 33.4 Hz); ^{19}F NMR (376 MHz, $CDCl_3$) δ -74.3; HRMS (ESI) m/z 334.0508 (M+H$^+$), calc. for $C_{17}H_{10}F_3NOS$ 334.0508.

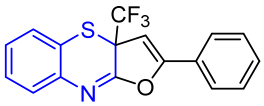

4d

2-phenyl-3a-(trifluoromethyl)-3a*H*-benzo[*b*]furo[2,3-e] [1,4]oxazine (4e): white solid; Mp 94.5 – 95.1 °C; 24.1 mg, 76% yield; ^{1}H NMR (600 MHz, $CDCl_3$) δ 7.80 – 7.73 (m, 2H), 7.55 – 7.46 (m, 3H), 7.39 (dd, *J* = 8.0, 1.6 Hz, 1H), 7.24 – 7.18 (m, 1H), 7.12 – 7.07 (m, 2H), 5.96 (s, 1H). ^{13}C NMR (151 MHz, $CDCl_3$) δ 162.1, 156.9, 142.8, 131.6, 131.5, 128.9, 128.6, 127.0, 126.7, 126.1, 123.6, 122.5 (q, *J* = 291.6 Hz), 115.7, 94.2, 78.0 (d, *J* = 33.6 Hz); ^{19}F NMR (565 MHz, $CDCl_3$) δ -77.5; HRMS (ESI) m/z 318.0737 (M+H$^+$), calc. for $C_{17}H_{10}F_3NO_2$ 318.0740.

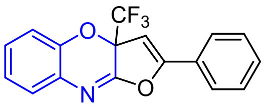

4e

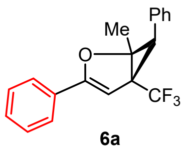

6a

1-methyl-3,6-diphenyl-5-(trifluoromethyl)-2-oxabicyclo[3.1.0]hex-3-ene (6a): white solid; Mp 117.6 – 119.6 °C; 27.2 mg, 86% yield; ^{1}H NMR (600 MHz, $CDCl_3$) δ 7.32 (dd, *J* = 6.4, 2.8 Hz, 2H), 7.28 (d, *J* = 7.6 Hz, 2H), 7.25 – 7.22 (m, 3H), 7.20 (t, *J* = 7.6 Hz, 2H), 7.13 (t, *J* = 7.3 Hz, 1H), 5.48 (s, 1H), 2.64 (s, 1H), 1.98 (s, 3H); ^{13}C NMR (151 MHz, $CDCl_3$) δ 157.2, 132.1, 130.3, 129.3, 128.8, 128.1, 128.0, 126.9, 125.0, 124.7 (q, *J* = 272.5 Hz), 94.7, 73.2, 44.0 (q, *J* = 37.1 Hz), 25.8, 17.3; ^{19}F NMR (565 MHz, $CDCl_3$) δ -65.3; HRMS (ESI) m/z 317.1148 (M+H$^+$), calc. for $C_{19}H_{15}F_3O$

317.1149.

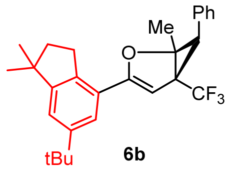
6b

3-(6-(*tert*-butyl)-1,1-dimethyl-2,3-dihydro-1*H*-inden-4-yl)-1-methyl-6-phenyl-5-(trifluoromethyl)-2-oxabicyclo[3.1.0]hex-3-ene (6b): white solid; Mp 108.7 – 110.5°C; 38.7 mg, 88% yield; ^{1}H NMR (600 MHz, $CDCl_3$) δ 7.29 (dq, *J* = 7.0, 1.1 Hz, 2H), 7.21 (t, *J* = 7.5 Hz, 2H), 7.14 (t, *J* = 7.4 Hz, 1H), 7.06 – 7.03 (m, 2H), 5.24 (s, 1H), 2.72 (ddd, *J* = 15.9, 8.0, 6.3 Hz, 1H), 2.60 – 2.52 (m, 2H), 1.94 (s, 3H), 1.81 (h, *J* = 5.7 Hz, 2H), 1.27 (s, 9H), 1.19 (s, 6H); ^{13}C NMR (151 MHz, $CDCl_3$) δ 157.4, 152.8, 149.4, 137.5, 132.3, 130.57, 128.0, 126.9, 124.8 (q, *J* = 273.2 Hz), 121.6, 119.3, 96.8, 72.7, 44.1 (d, *J* = 36.9 Hz), 43.8, 41.3, 34.6, 31.4, 29.7, 28.7, 28.6, 25.5, 17.3; ^{19}F NMR (565 MHz, $CDCl_3$) δ -65.3; HRMS (ESI) m/z 441.2400 (M+H$^+$), calc. for $C_{28}H_{31}F_3O$ 441.2395.

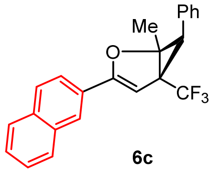
6c

1-methyl-3-(naphthalen-1-yl)-6-phenyl-5-(trifluoromethyl)-2-oxabicyclo[3.1.0]hex-3-ene (6c): white solid; Mp 80.4 – 81.4 °C 29.3 mg, 80% yield; ^{1}H NMR (600 MHz, $CDCl_3$) δ 7.82 (s, 1H), 7.80 – 7.77 (m, 1H), 7.76 – 7.71 (m, 1H), 7.66 (d, *J* = 8.6 Hz, 1H), 7.47 – 7.42 (m, 2H), 7.35 (dd, *J* = 8.6, 1.6 Hz, 1H), 7.29 (d, *J* = 7.8 Hz, 2H), 7.15 (t, *J* = 7.7 Hz, 2H), 7.07 (t, *J* = 7.4 Hz, 1H), 5.59 (s, 1H), 2.66 (s, 1H), 2.01 (s, 3H); ^{13}C NMR (151 MHz, $CDCl_3$) δ 157.1, 133.3, 132.9, 132.1, 130.3, 128.3, 128.0, 127.8, 127.6, 126.9, 126.5, 126.4, 126.3, 124.0, 124.7 (q, *J* = 272.5 Hz), 122.7, 95.4, 73.3, 44.1 (q, *J* = 37.1 Hz), 26.1, 17.3; ^{19}F NMR (565 MHz, $CDCl_3$) δ -65.2 HRMS (ESI) m/z 367.1304 (M+H$^+$), calc. for $C_{23}H_{17}F_3O$ 367.1308.

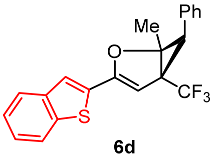
6d

3-(benzo[b]thiophen-2-yl)-1-methyl-6-phenyl-5-(trifluoromethyl)-2-oxabicyclo[3.1.0]hex-3-ene (6d): yellow solid; Mp 83.5 – 85.2 °C; 33.5 mg, 90% yield; ^{1}H NMR (600 MHz, $CDCl_3$) δ 7.80 – 7.75 (m, 1H), 7.73 – 7.69 (m, 1H), 7.36 – 7.27 (m, 5H), 7.19 (dd, *J* = 8.4, 6.9 Hz, 2H), 7.13 – 7.09 (m, 1H), 5.51 (s, 1H), 2.66 (s, 1H), 1.99 (s, 3H); ^{13}C NMR (151 MHz, $CDCl_3$) δ 152.6, 140.1, 136.0, 132.0, 130.4, 128.1, 127.0, 125.9, 125.7, 124.7 (q, *J* = 271.8 Hz), 124.5, 122.9, 122.6, 96.3, 72.6, 43.8 (q, *J* = 37.0 Hz), 25.7, 17.3; ^{19}F NMR (565 MHz, $CDCl_3$) δ -65.2; HRMS (ESI) m/z 378.0868 (M+H$^+$), calc. for $C_{18}H_{14}F_3NO$ 378.0869.

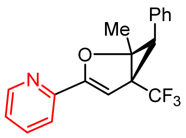

6e

2-(1-methyl-6-phenyl-5-(trifluoromethyl)-2-oxabicyclo[3.1.0]hex-3-en-3-yl)pyridine (6e): white solid; Mp 108.7 – 110.5 °C; 26.3 mg, 83% yield; ^{1}H NMR (600 MHz, $CDCl_3$) δ 8.45 (d, *J* = 4.7 Hz, 1H), 7.54 (td, *J* = 7.8, 1.7 Hz, 1H), 7.28 (dd, *J* = 5.6, 4.7 Hz, 2H), 7.16 (t, *J* = 7.6 Hz, 2H), 7.09 (ddd, *J* = 7.7, 4.6, 3.5 Hz, 2H), 5.96 (s, 1H), 2.67 (s, 1H), 1.98 (s, 3H); ^{13}C NMR (151 MHz, $CDCl_3$) δ 156.2, 149.3, 147.8, 136.2, 131.7, 130.2, 128.0, 126.9, 124.5 (q, *J* = 272.6 Hz), 123.2, 119.4, 98.7, 73.9, 44.0 (q, *J* = 37.1 Hz), 25.9, 17.3; ^{19}F NMR (565 MHz, $CDCl_3$) δ -65.2; HRMS (ESI) m/z 318.1100 ($M+H^+$), calc. for $C_{18}H_{14}F_3NO$ 318.1099.

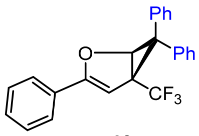

6f

3,6,6-triphenyl-5-(trifluoromethyl)-2-oxabicyclo[3.1.0]hex-3-ene (6f): yellow solid; Mp 110.9 – 111.9 °C 34.8 mg, 92% yield; ^{1}H NMR (600 MHz, $CDCl_3$) δ 7.60 (d, *J* = 7.4 Hz, 2H), 7.42 (dd, *J* = 8.1, 1.0 Hz, 2H), 7.31 (t, *J* = 7.7 Hz, 2H), 7.27 – 7.24 (m, 2H), 7.21 (t, *J* = 6.6 Hz, 4H), 7.15 (t, *J* = 7.8 Hz, 2H), 7.03 – 6.99 (m, 1H), 5.62 (s, 1H), 5.62 (s, 1H); ^{13}C NMR (151 MHz, $CDCl_3$) δ 158.7, 138.2, 137.1, 130.1, 129.1, 130.0, 129.0, 128.7, 128.2, 128.1, 127.3, 126.7, 125.1, 124.5 (q, *J* = 273.6 Hz), 97.6, 69.1, 47.3 (q, *J* = 36.7 Hz); 38.1; ^{19}F NMR (565 MHz, $CDCl_3$) δ -60.4; HRMS (ESI) m/z 379.1304 ($M+H^+$), calc. for $C_{24}H_{17}F_3O$ 379.1310.

11. Copies of NMR spectra

^{1}H NMR (600 MHz, $CDCl_3$) of compound (*E*)-**1a**

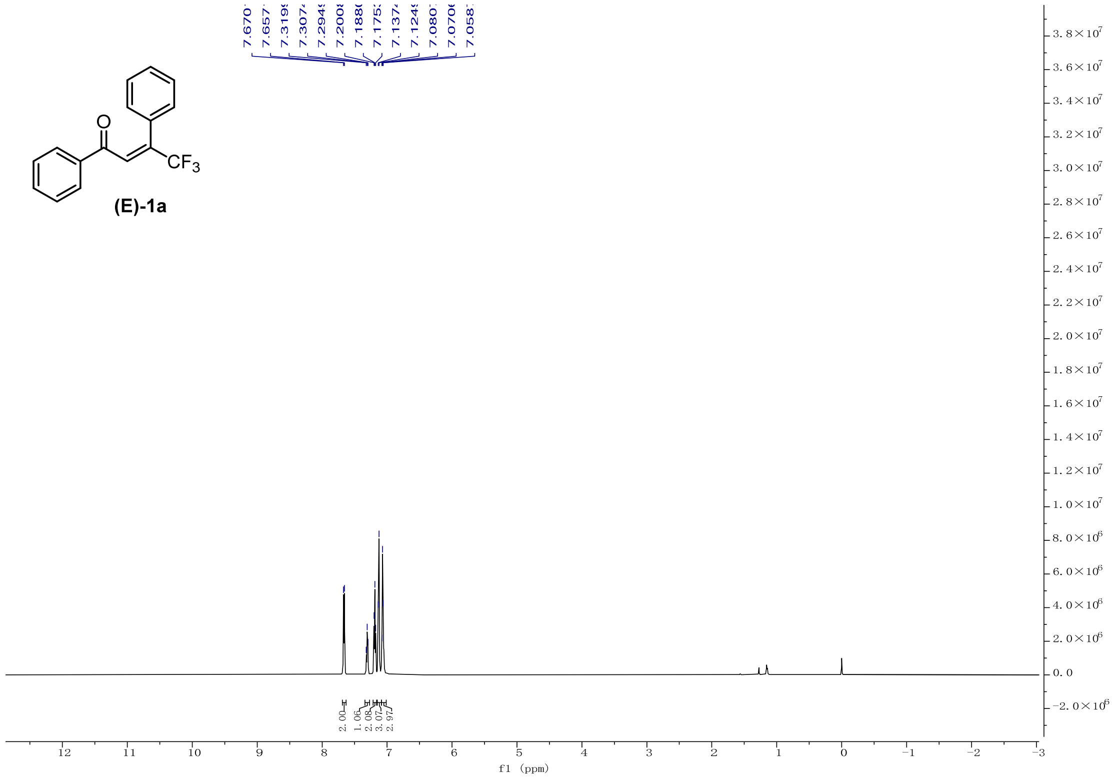

^{13}C NMR (151 MHz, $CDCl_3$) of compound (*E*)-**1a**

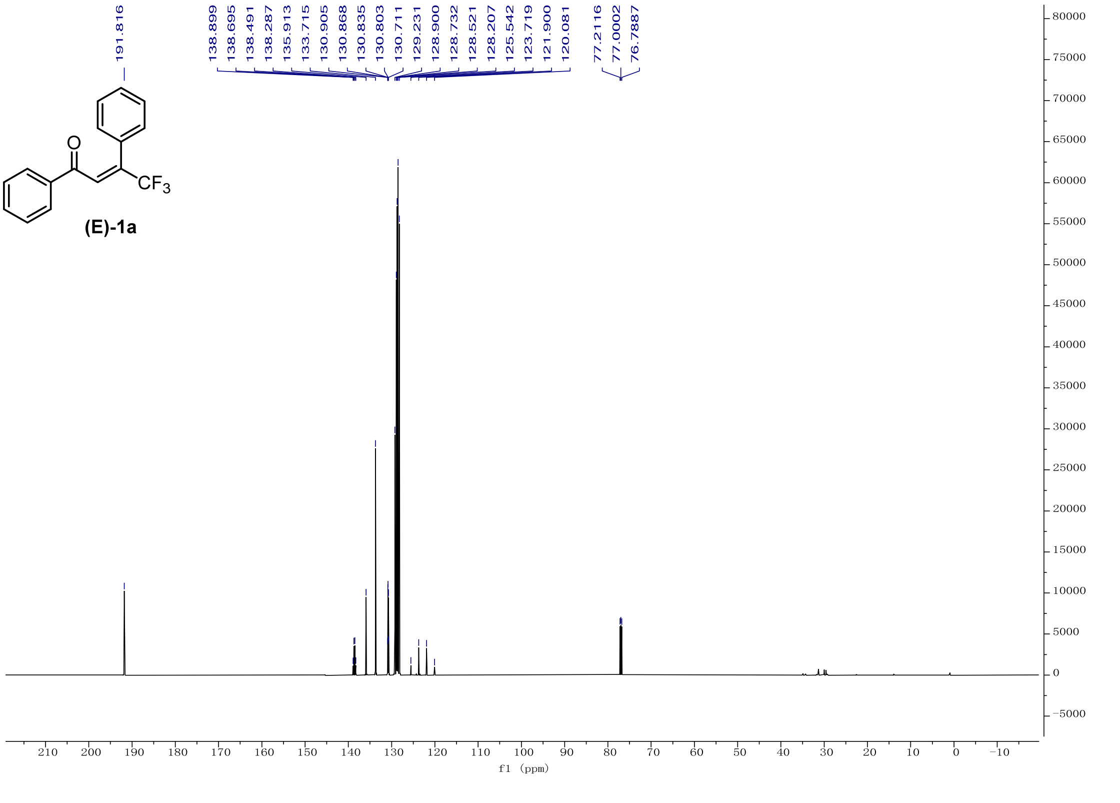

^{19}F NMR (565 MHz, $CDCl_3$) of compound (*E*)-**1a**

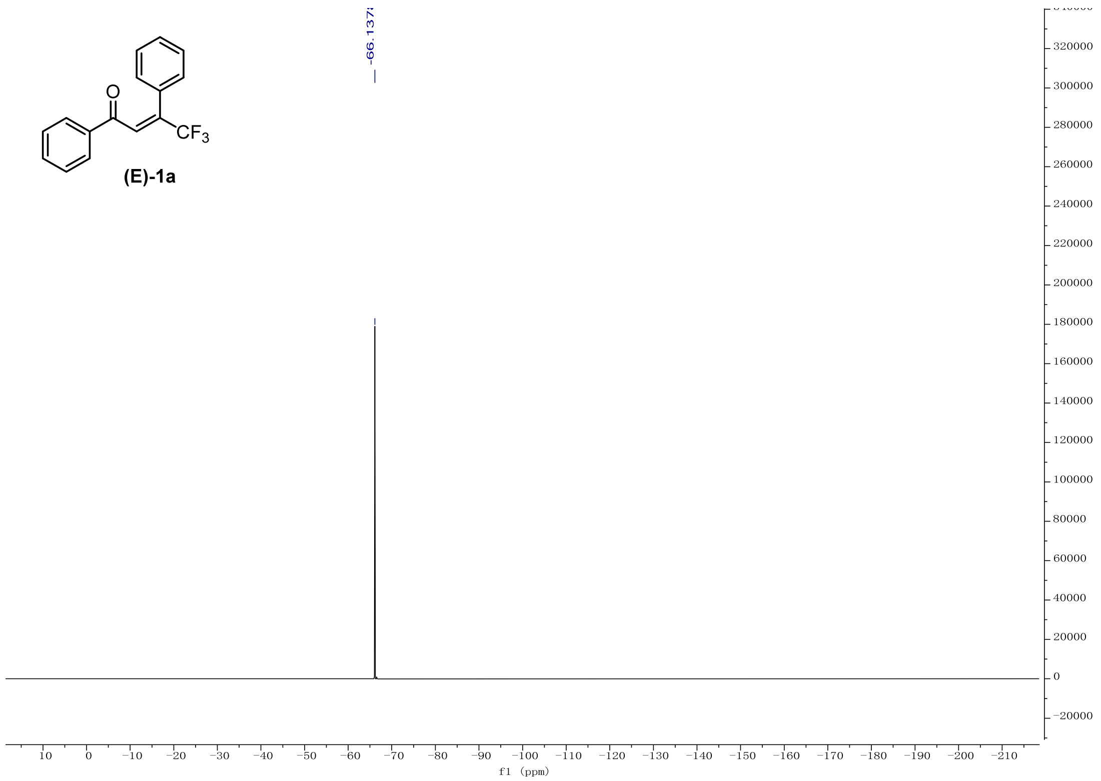

^{1}H NMR (300 MHz, $CDCl_3$) of compound (*Z*)-**1a**

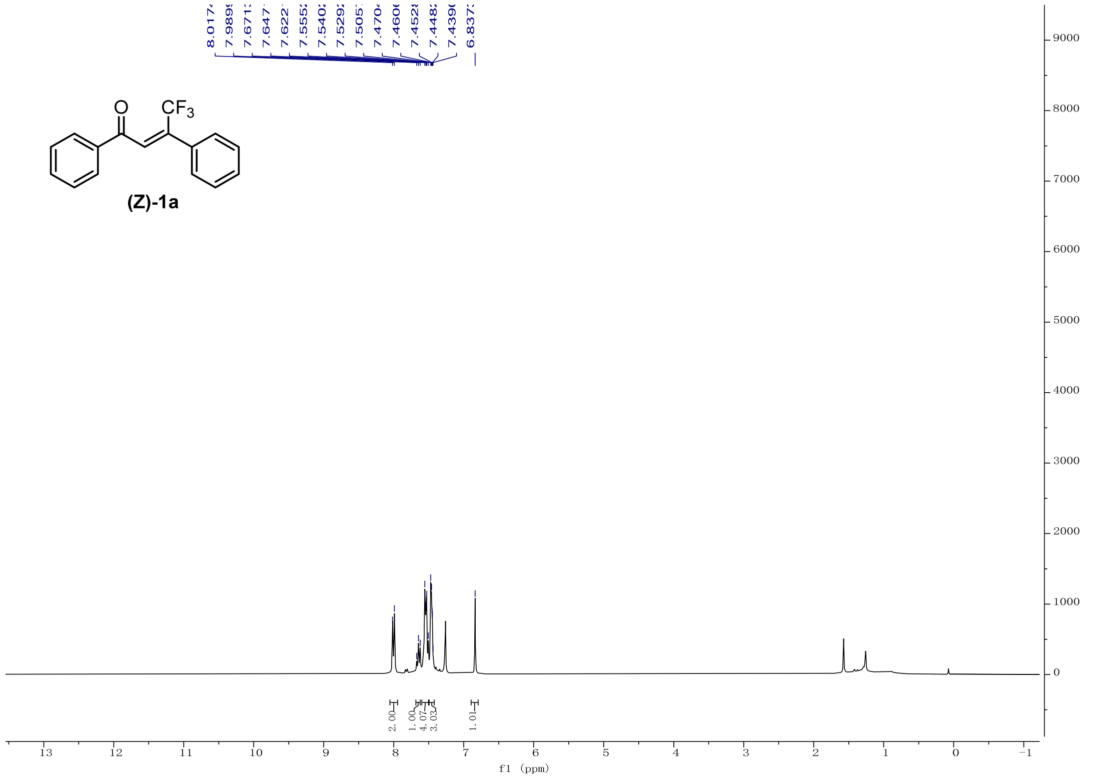

^{13}C NMR (75 MHz, $CDCl_3$) of compound (*Z*)-**1a**

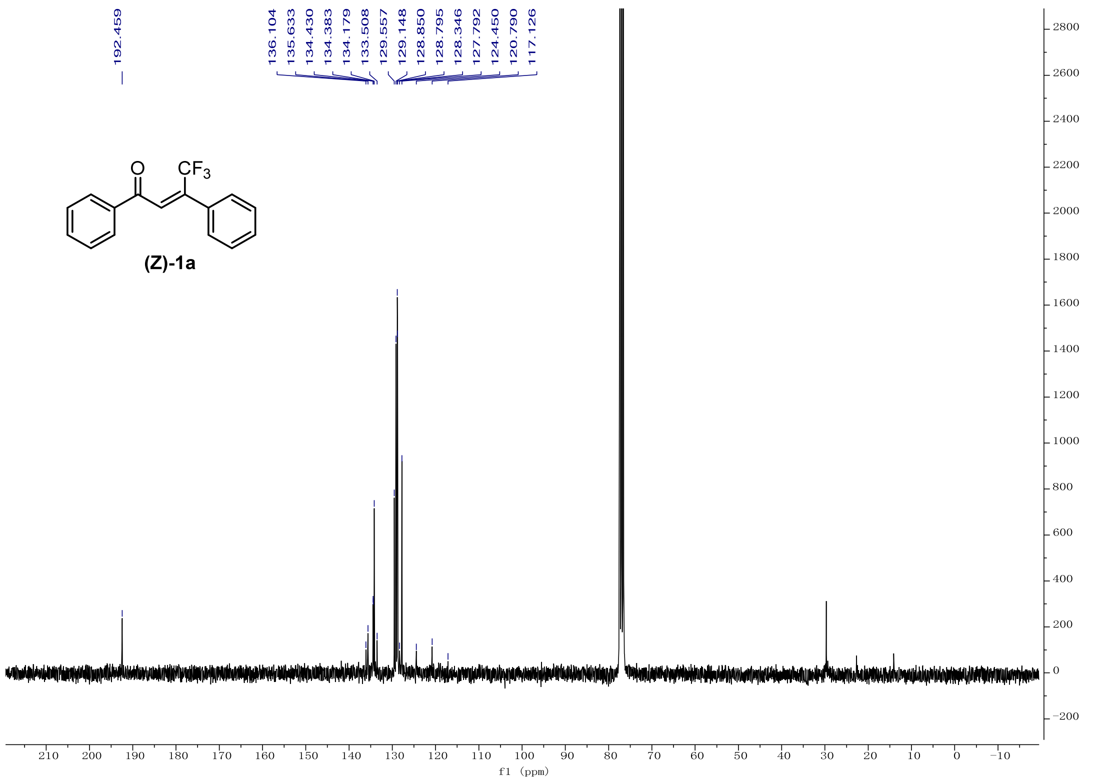

^{19}F NMR (376 MHz, $CDCl_3$) of compound (*Z*)-**1a**

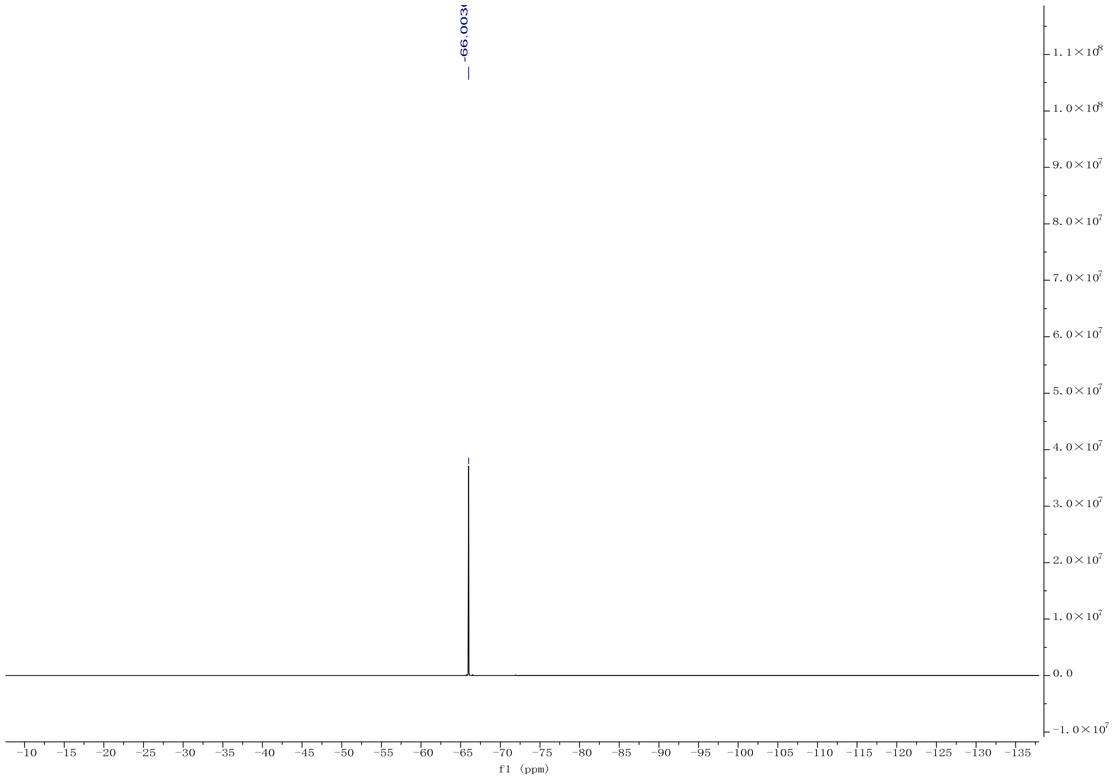

^{1}H NMR (300 MHz, $CDCl_3$) of compound **1b**

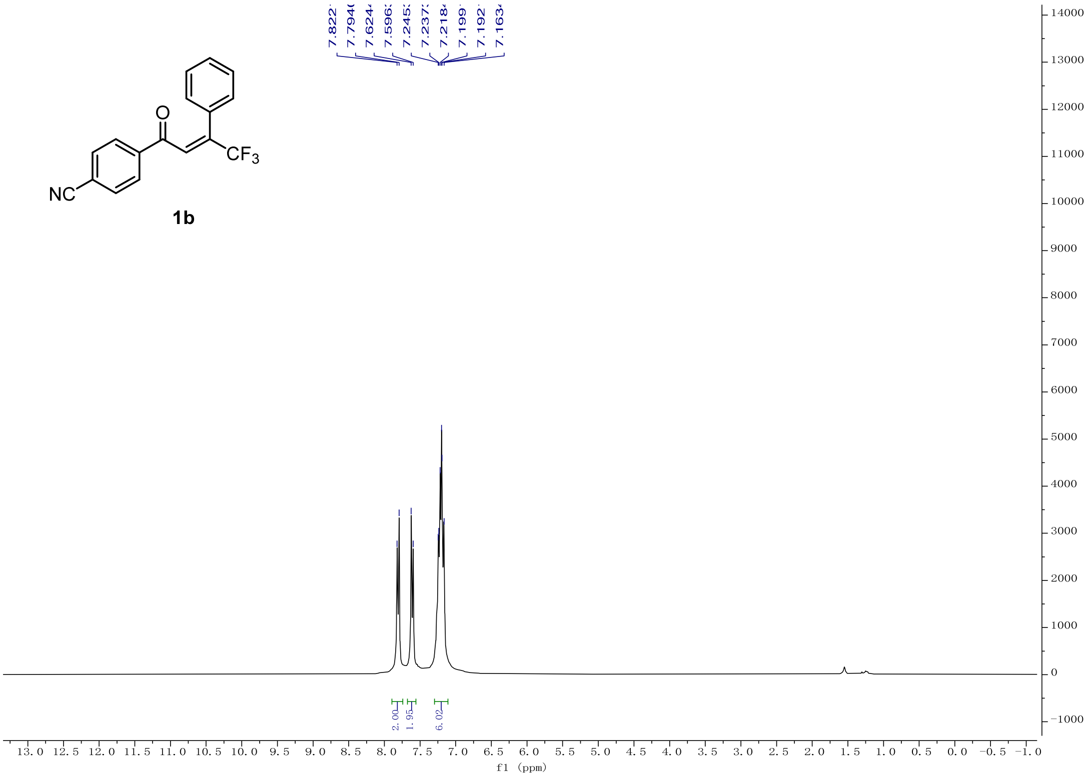

^{13}C NMR (75 MHz, $CDCl_3$) of compound **1b**

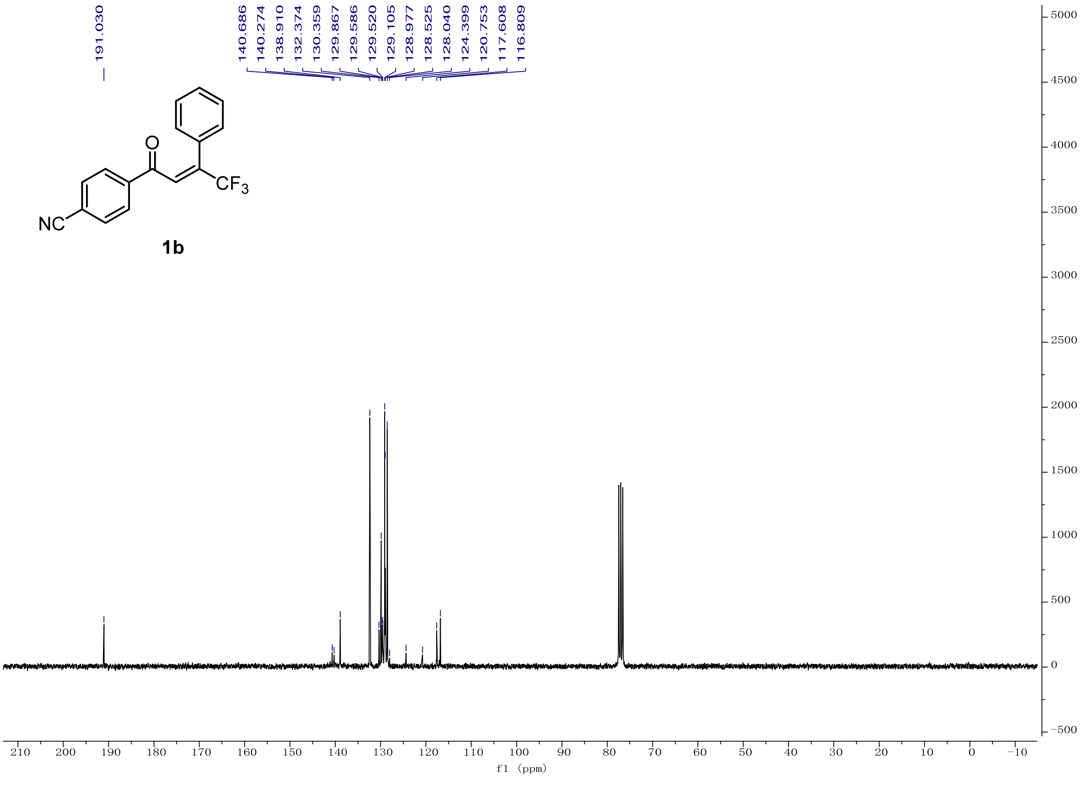

^{19}F NMR (376 MHz, $CDCl_3$) of compound **2b**

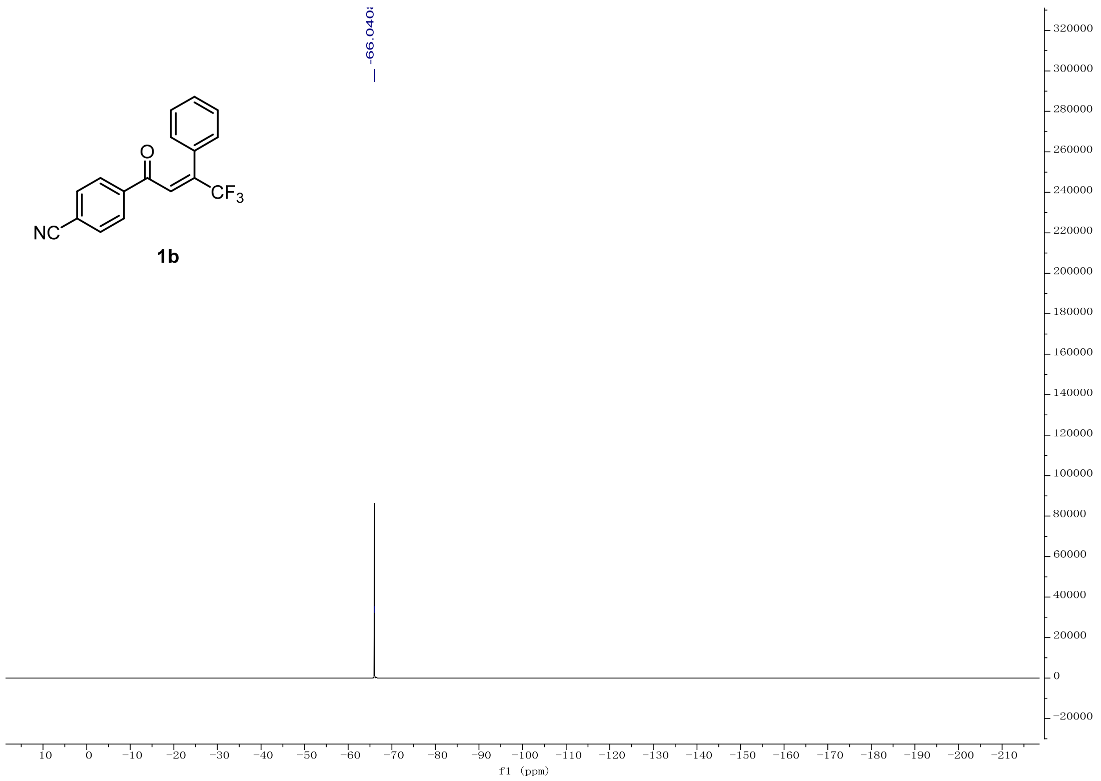

^{1}H NMR (300 MHz, $CDCl_3$) of compound **1f**

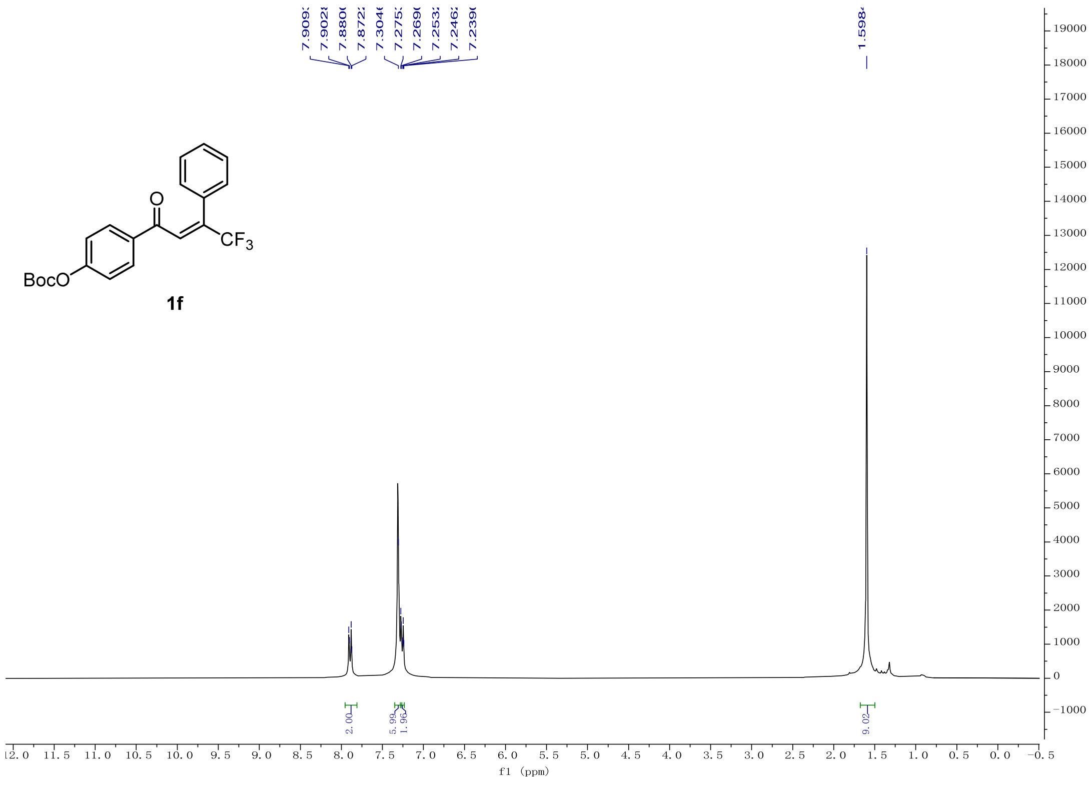

^{13}C NMR (75 MHz, $CDCl_3$) of compound **1f**

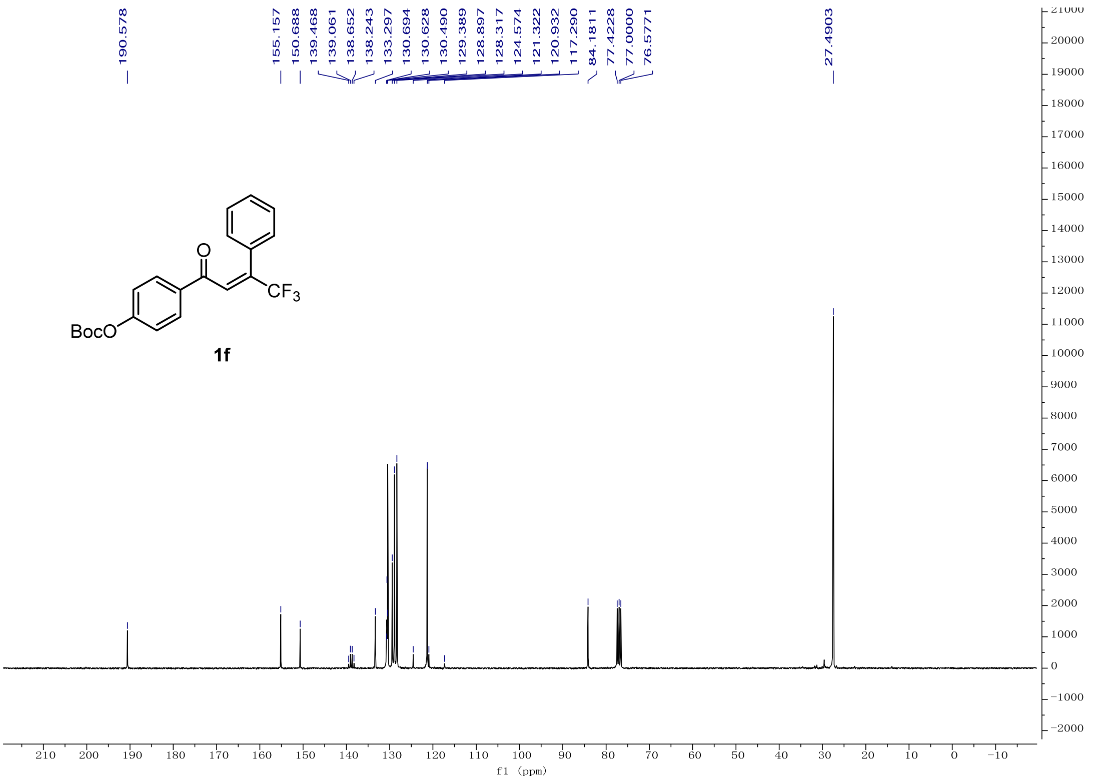

^{19}F NMR (376 MHz, $CDCl_3$) of compound **1f**

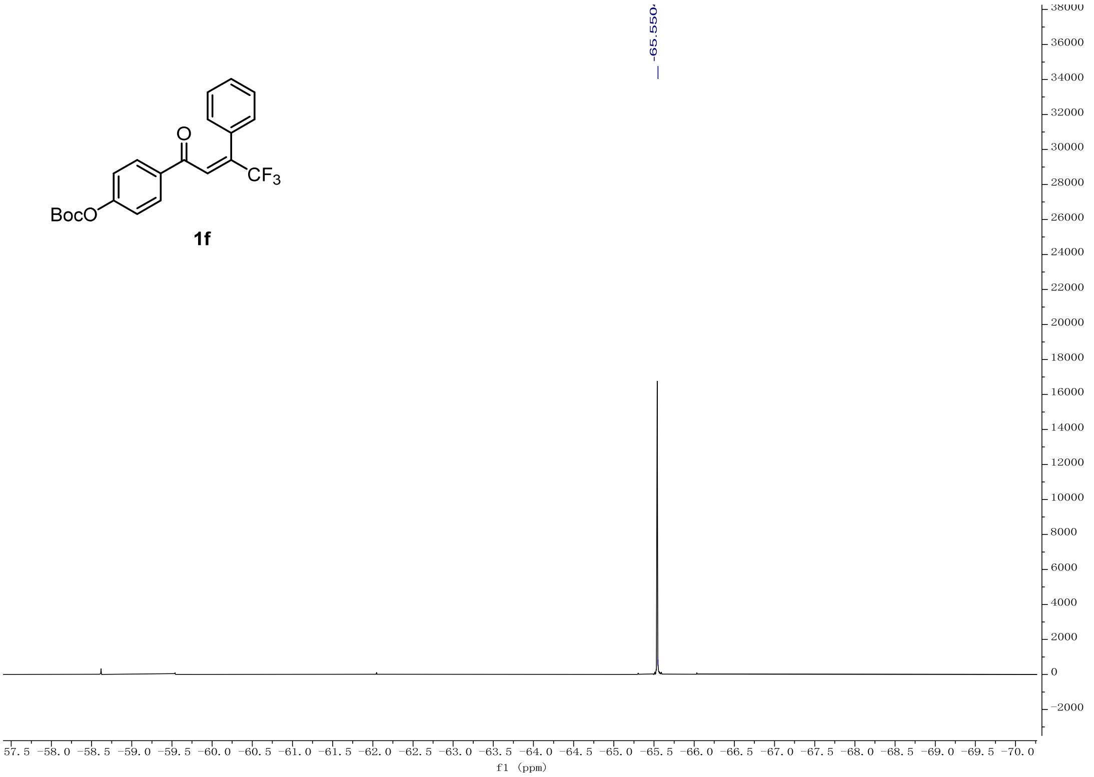

^{1}H NMR (300 MHz, $CDCl_3$) of compound **1g**

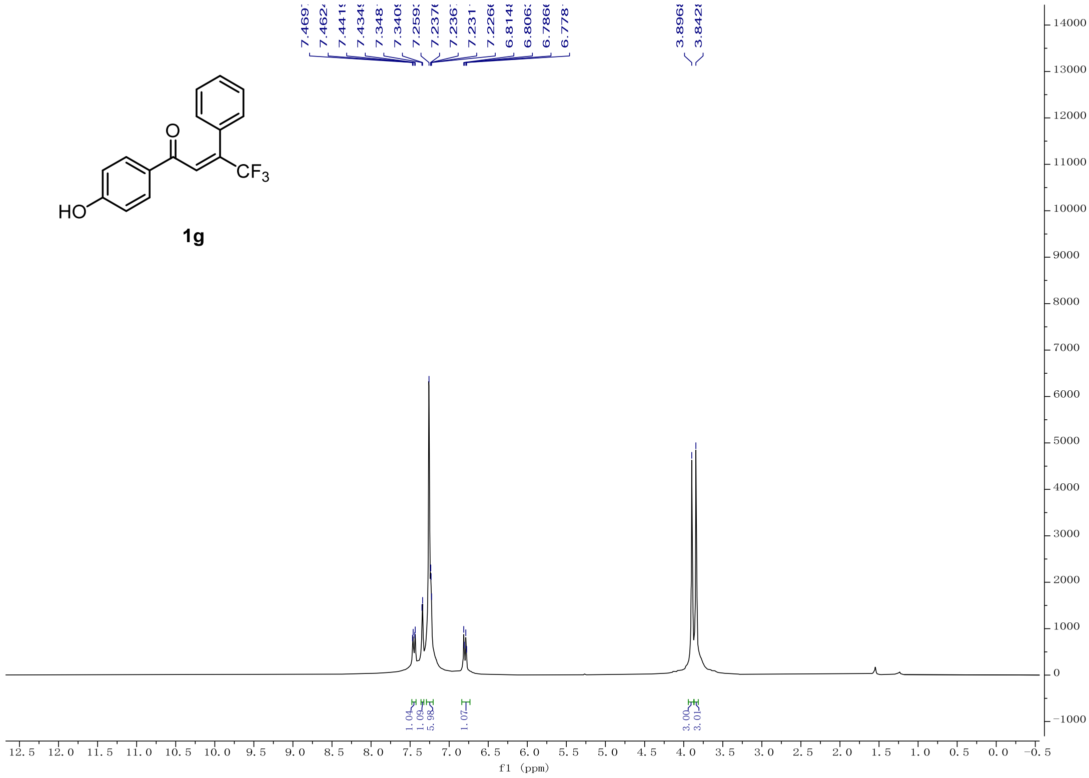

^{13}C NMR (75 MHz, $CDCl_3$) of compound **1g**

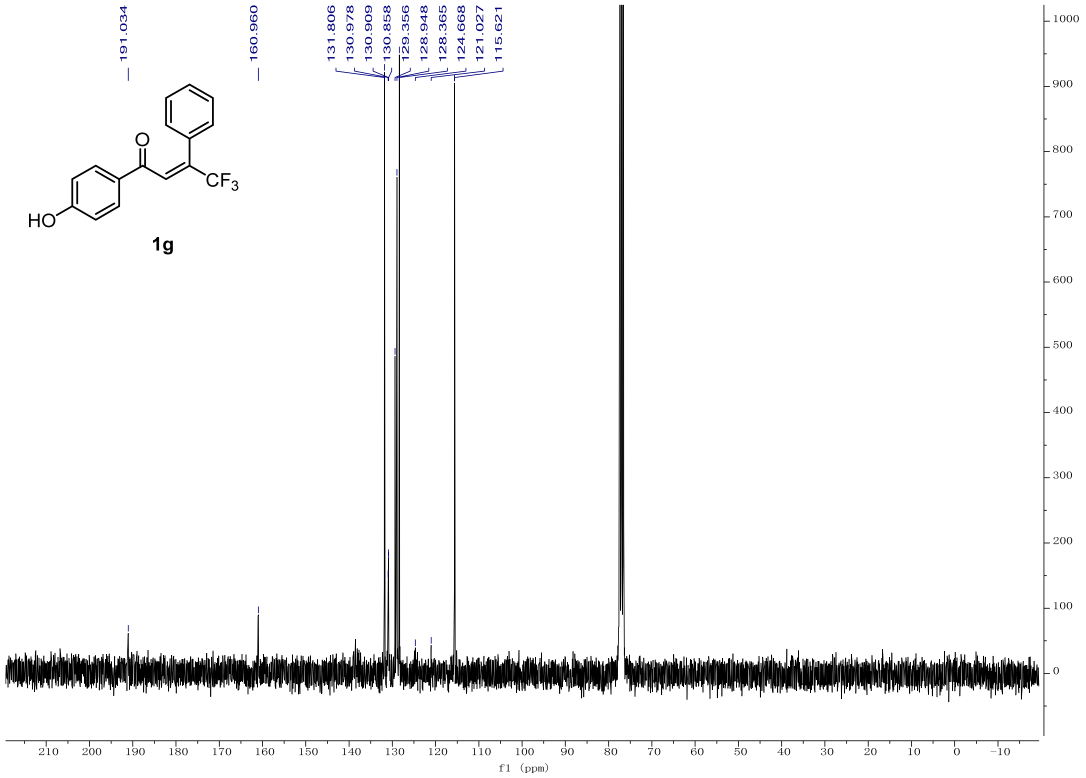

^{19}F NMR (376 MHz, $CDCl_3$) of compound **1g**

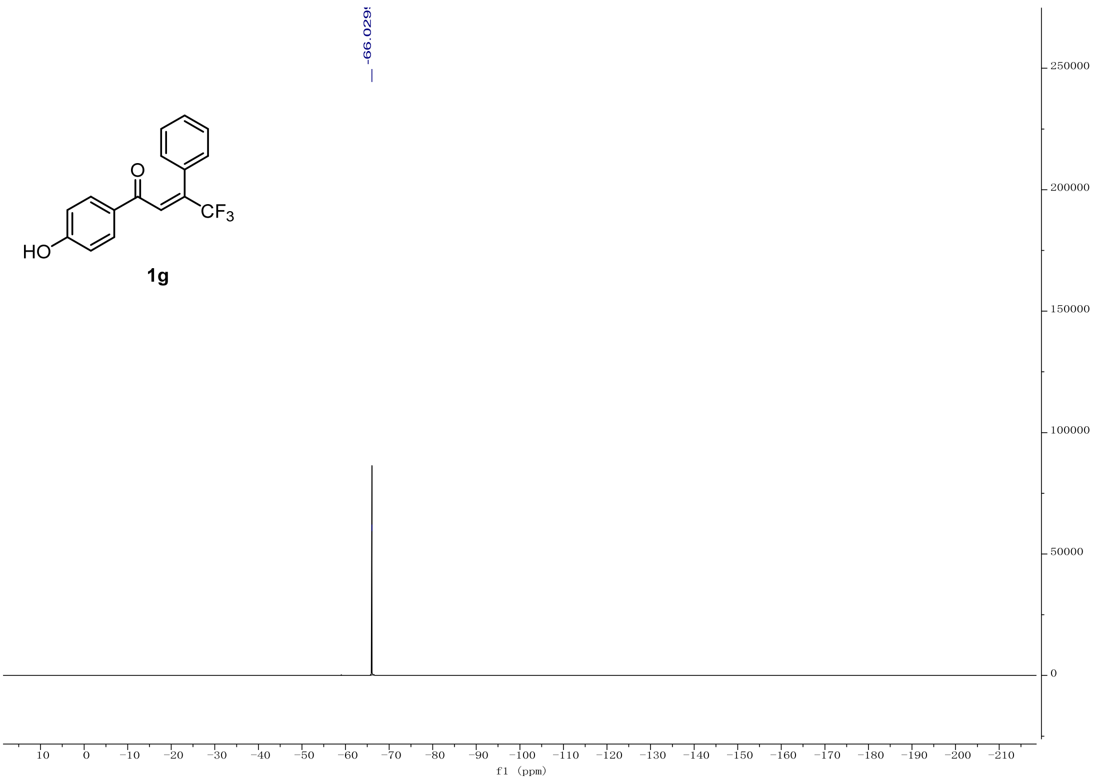

^{1}H NMR (300 MHz, $CDCl_3$) of compound **1h**

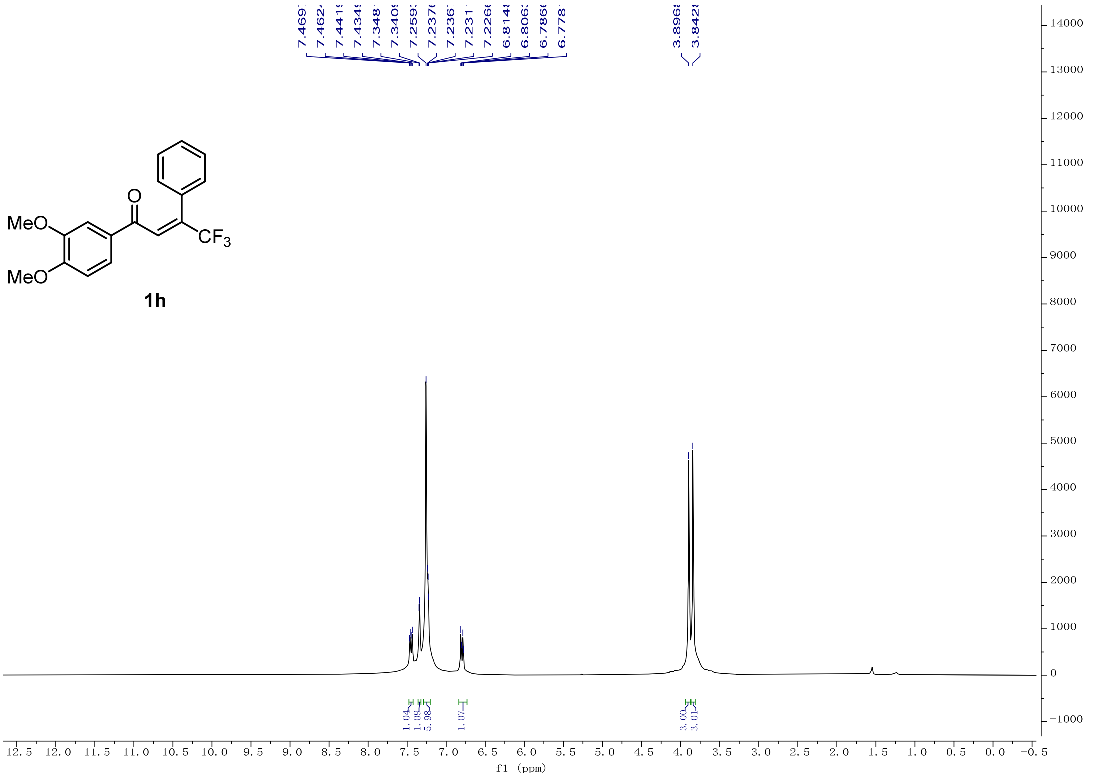

^{13}C NMR (75 MHz, $CDCl_3$) of compound **1h**

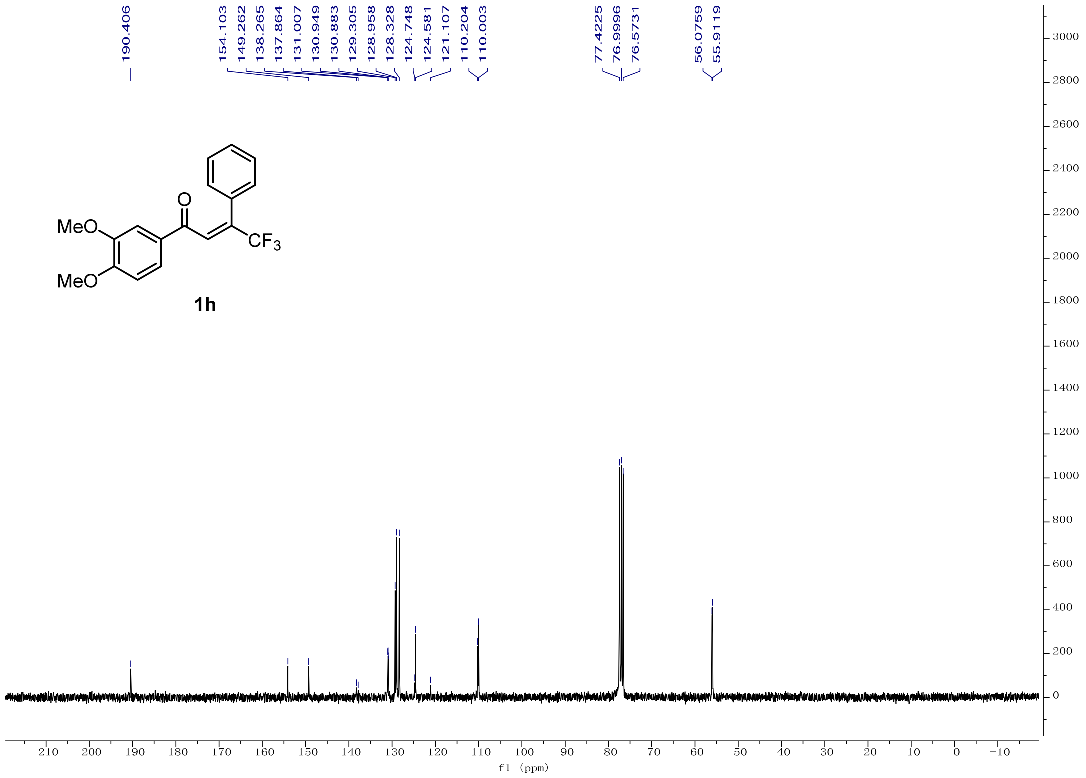

^{19}F NMR (376 MHz, $CDCl_3$) of compound **1h**

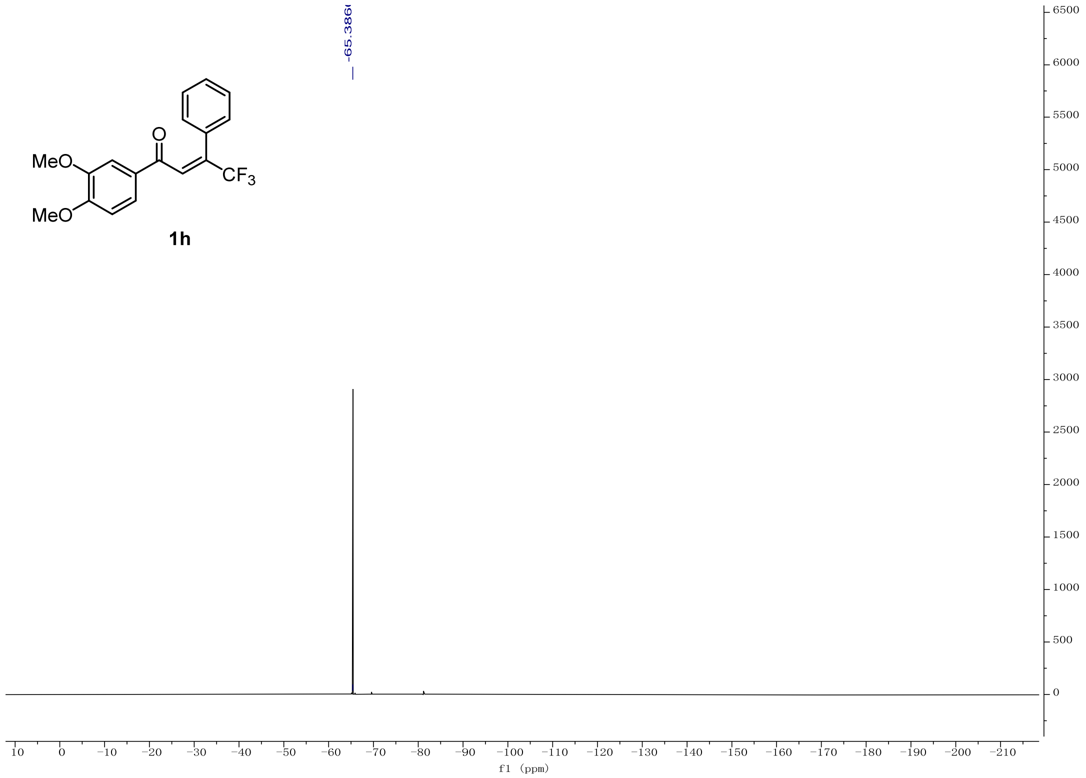

^{1}H NMR (300 MHz, $CDCl_3$) of compound **1i**

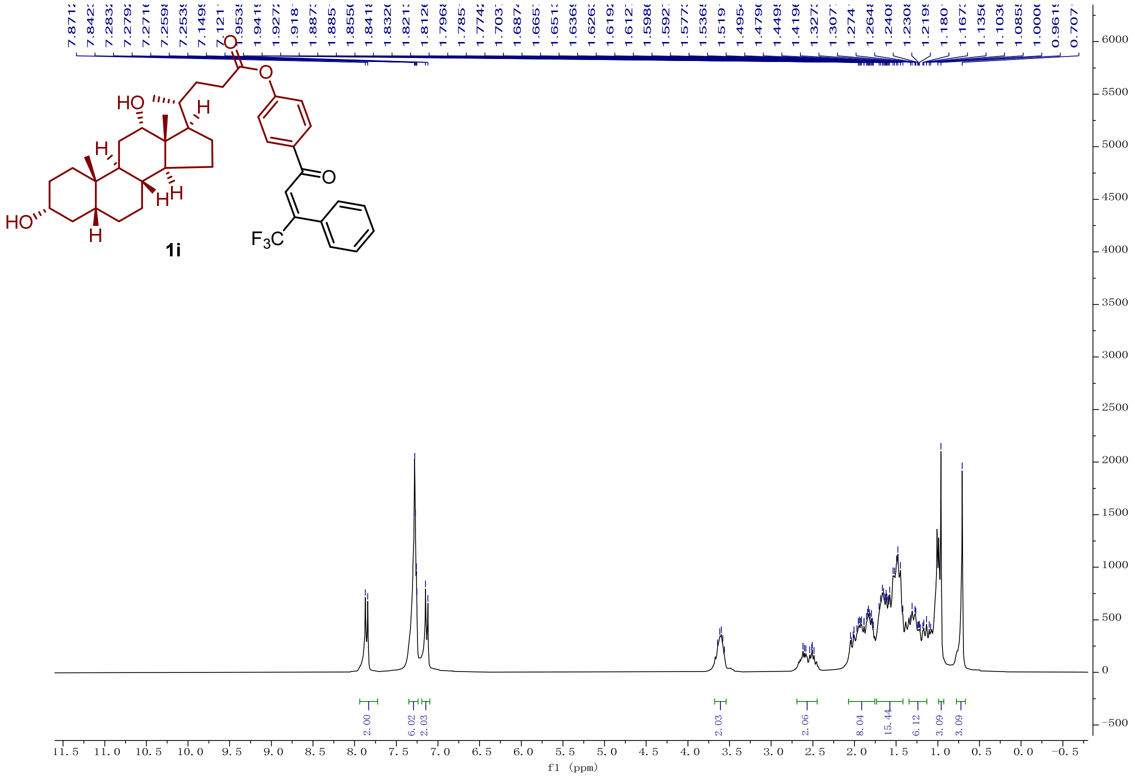

^{13}C NMR (75 MHz, $CDCl_3$) of compound **1i**

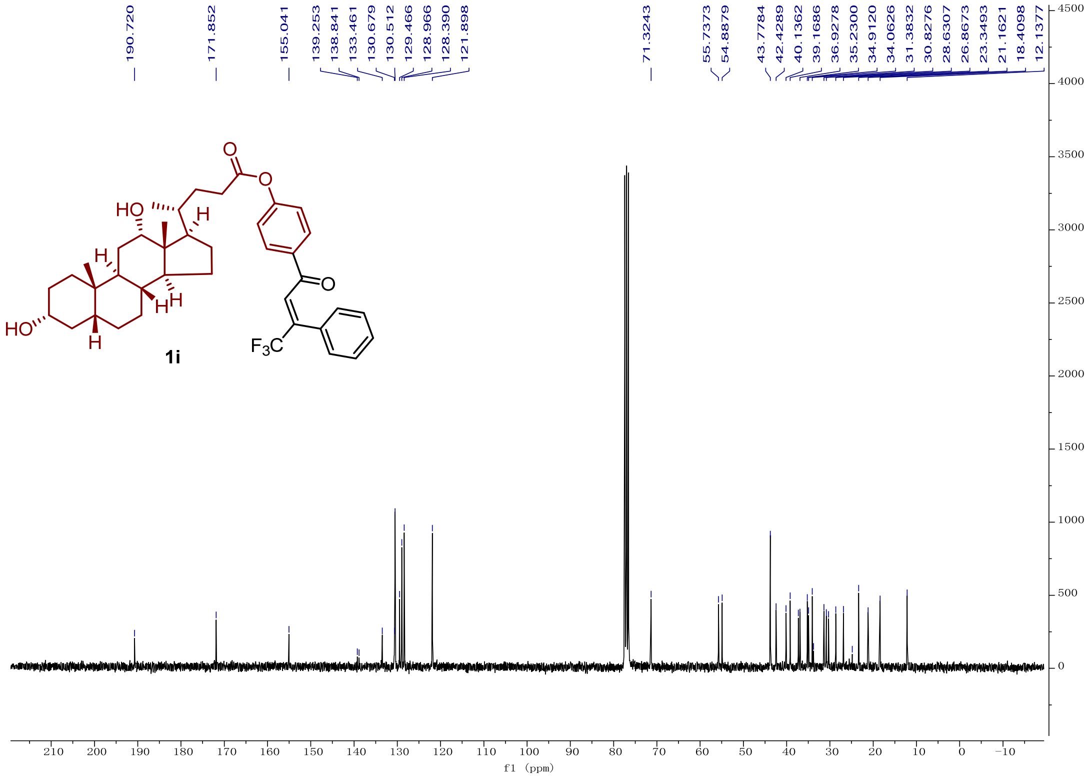

^{19}F NMR (376 MHz, $CDCl_3$) of compound **1i**

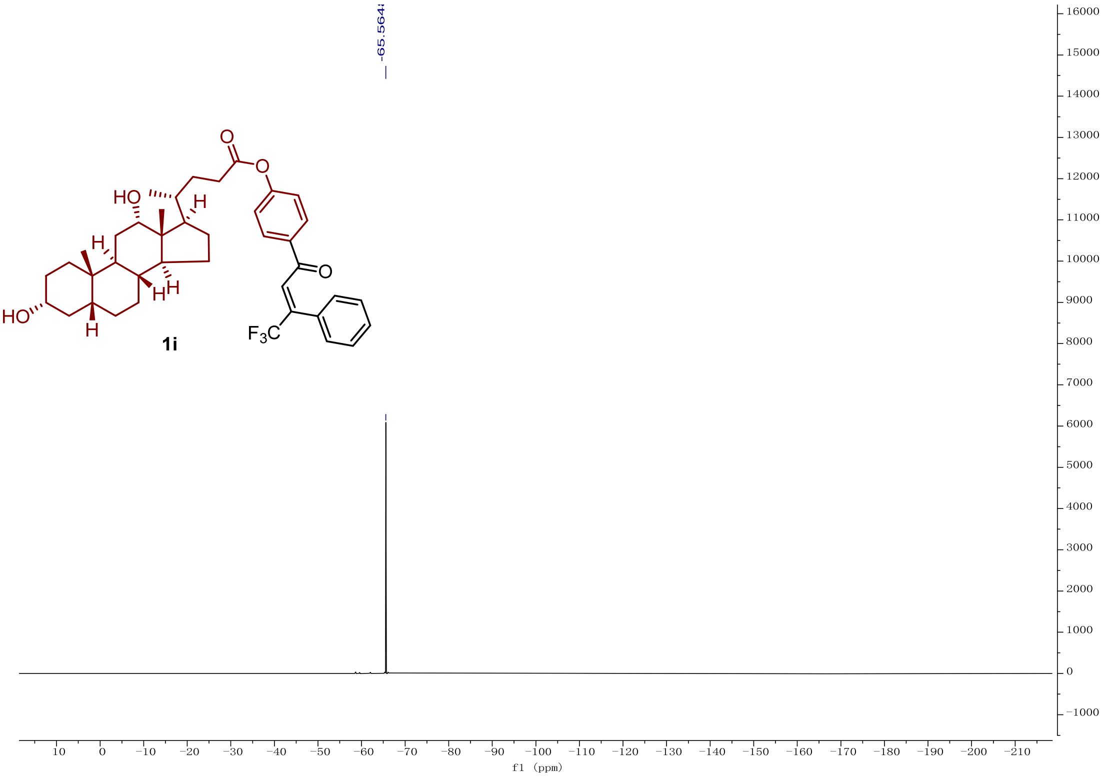

^{1}H NMR (300 MHz, $CDCl_3$) of compound **1j**

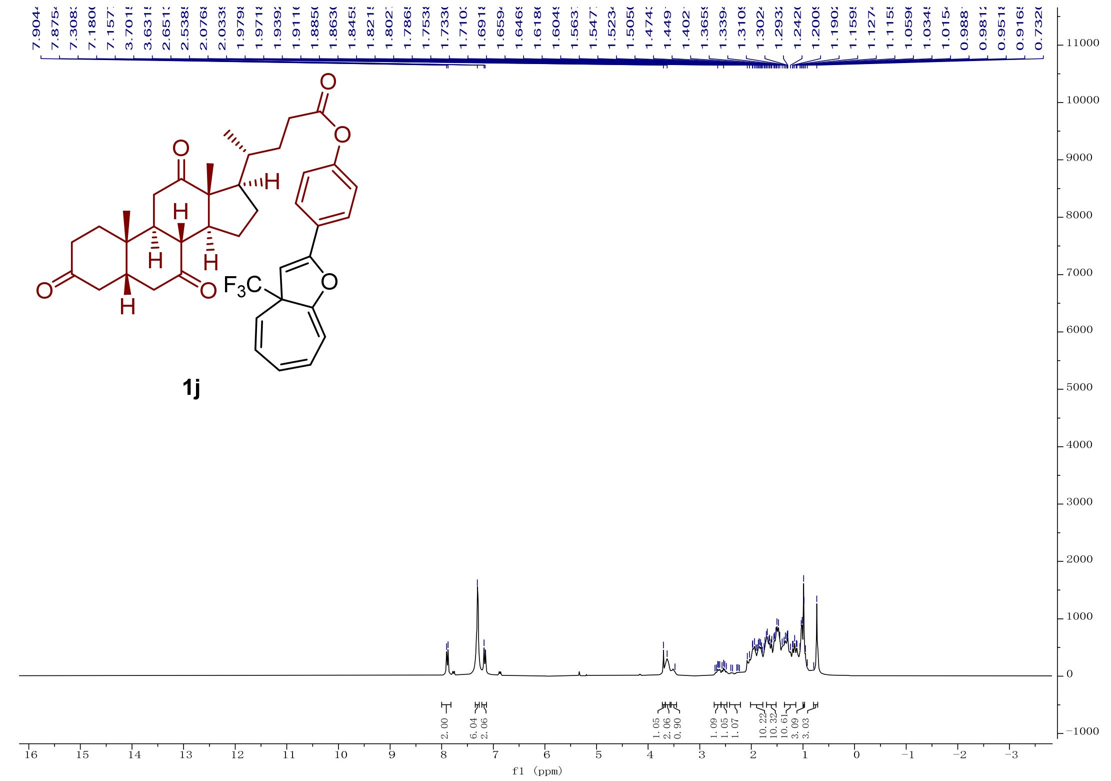

[13] C NMR (75 MHz, DMSO) of compound **1j**

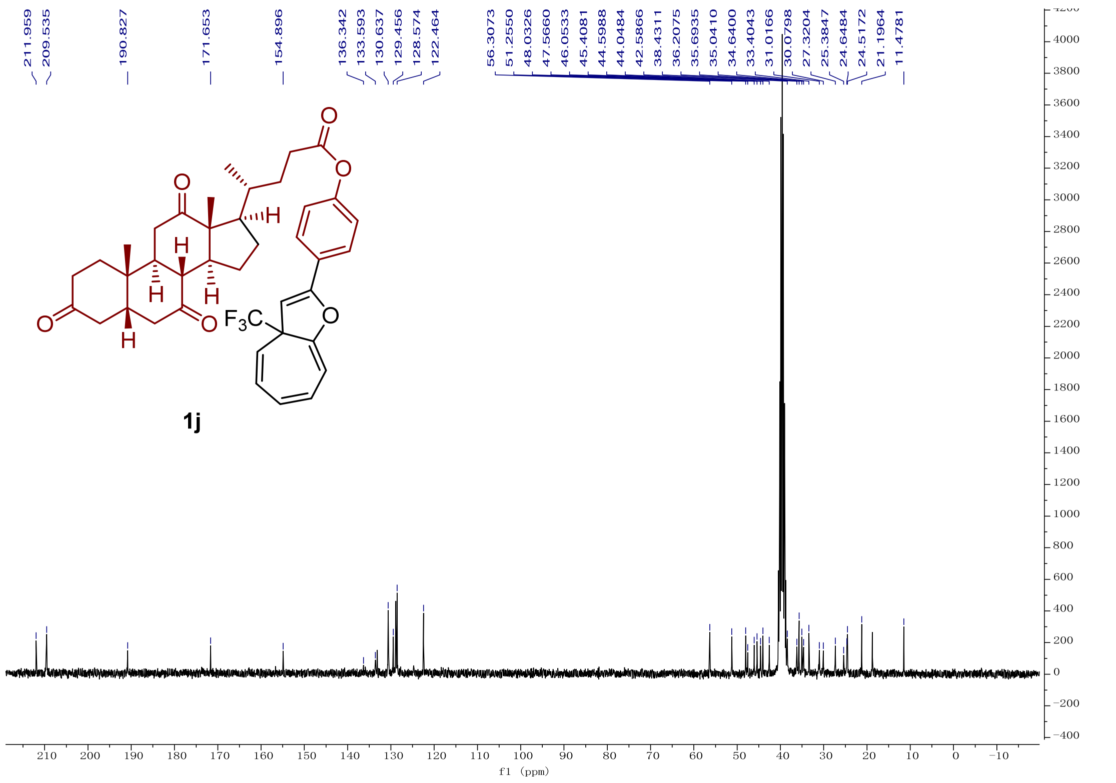

[19] F NMR (376 MHz, $CDCl_3$) of compound **1j**

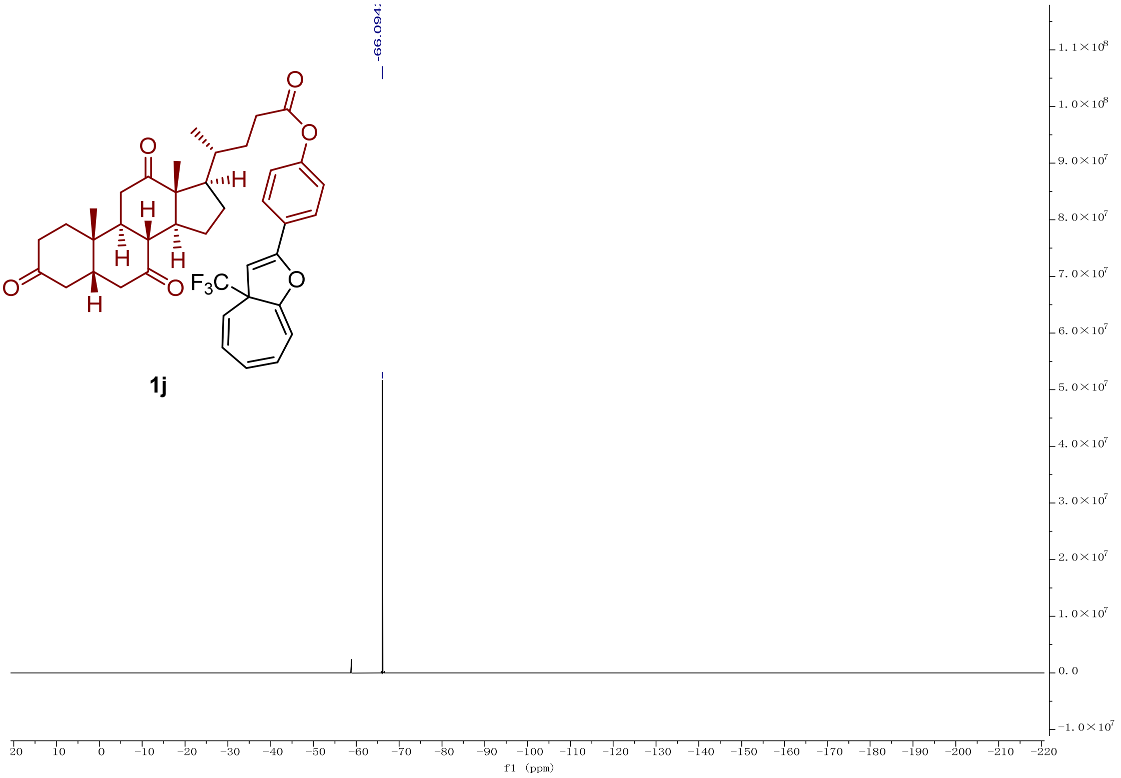

^{1}H NMR (300 MHz, $CDCl_3$) of compound **1k**

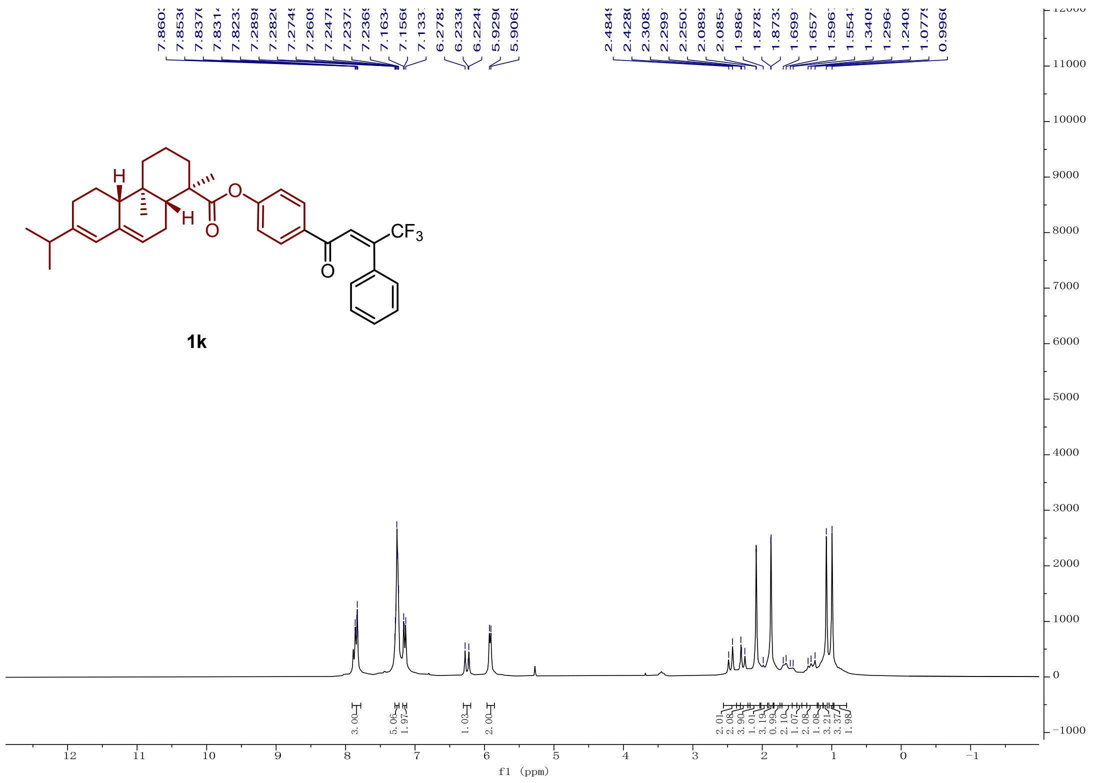

^{13}C NMR (75 MHz, $CDCl_3$) of compound **1k**

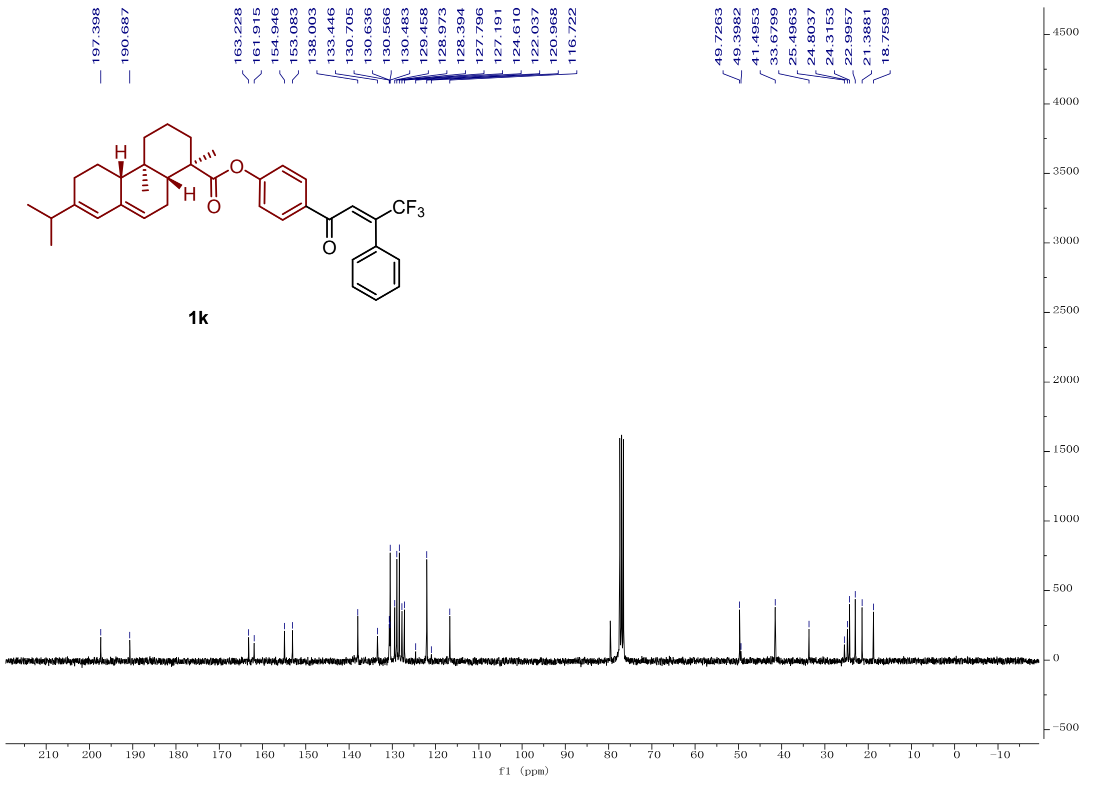

^{19}F NMR (376 MHz, $CDCl_3$) of compound **1k**

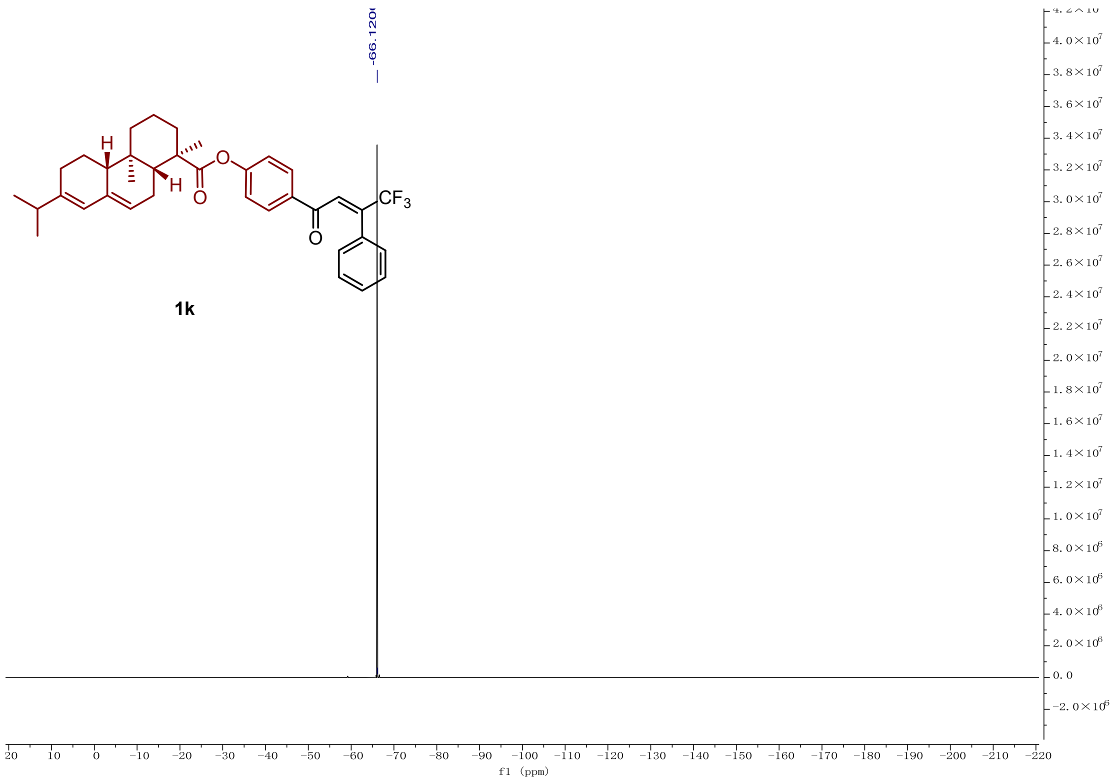

^{1}H NMR (300 MHz, $CDCl_3$) of compound **1l**

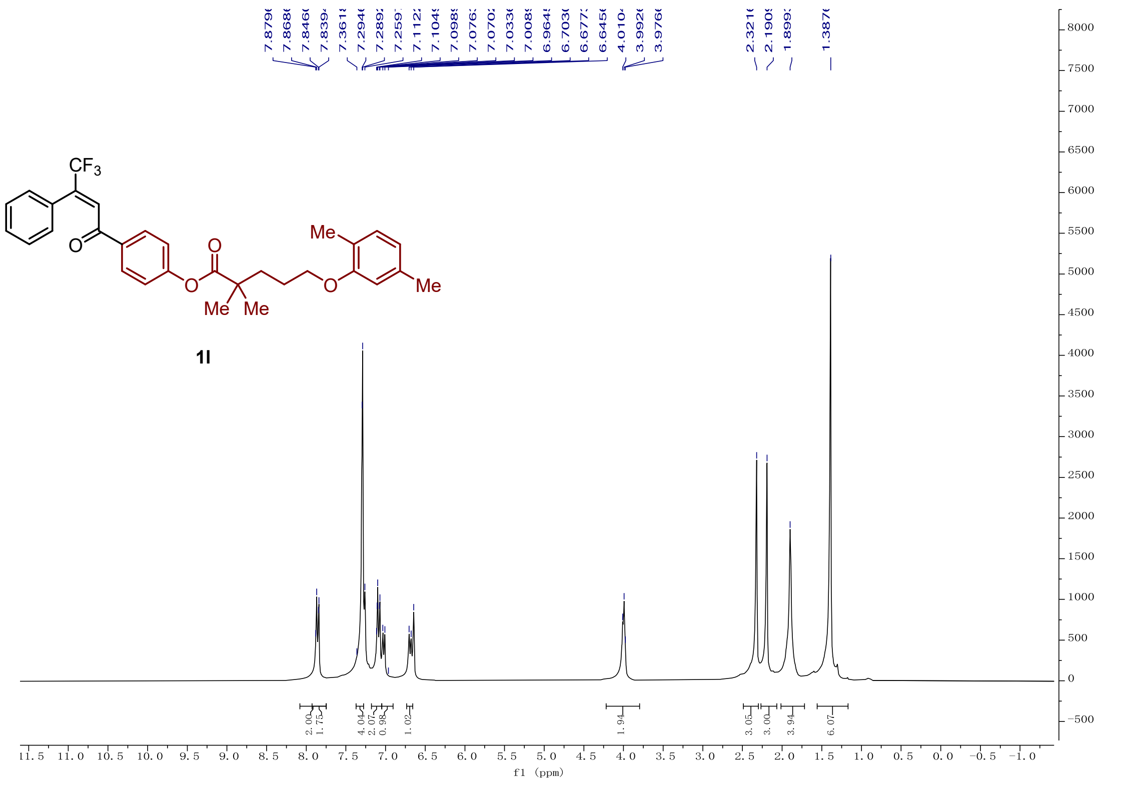

^{13}C NMR (75 MHz, $CDCl_3$) of compound **1l**

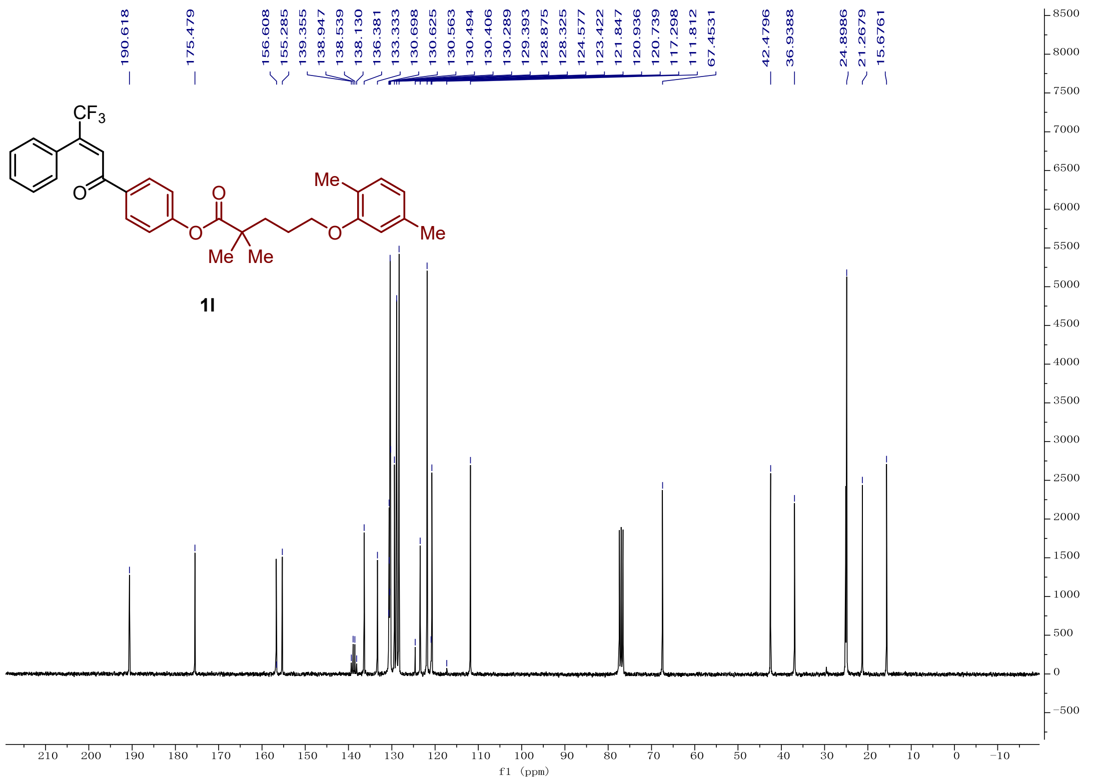

^{19}F NMR (376 MHz, $CDCl_3$) of compound **1l**

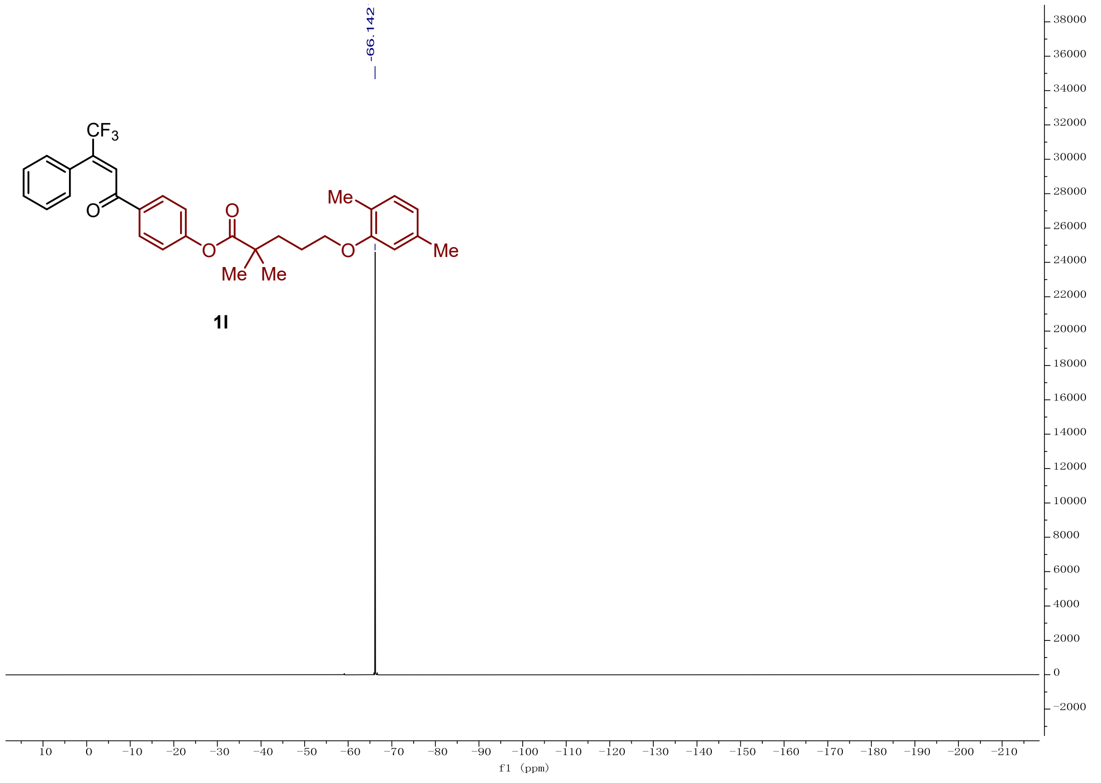

^{1}H NMR (300 MHz, $CDCl_3$) of compound **1m**

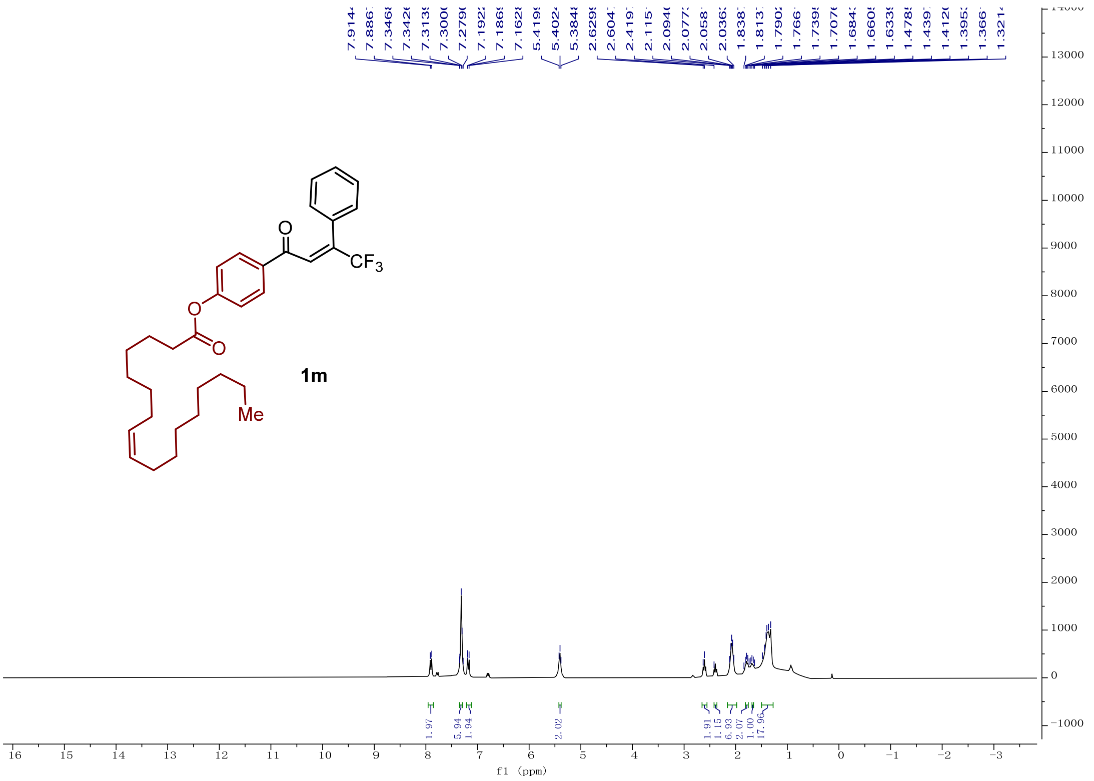

^{13}C NMR (75 MHz, $CDCl_3$) of compound **1m**

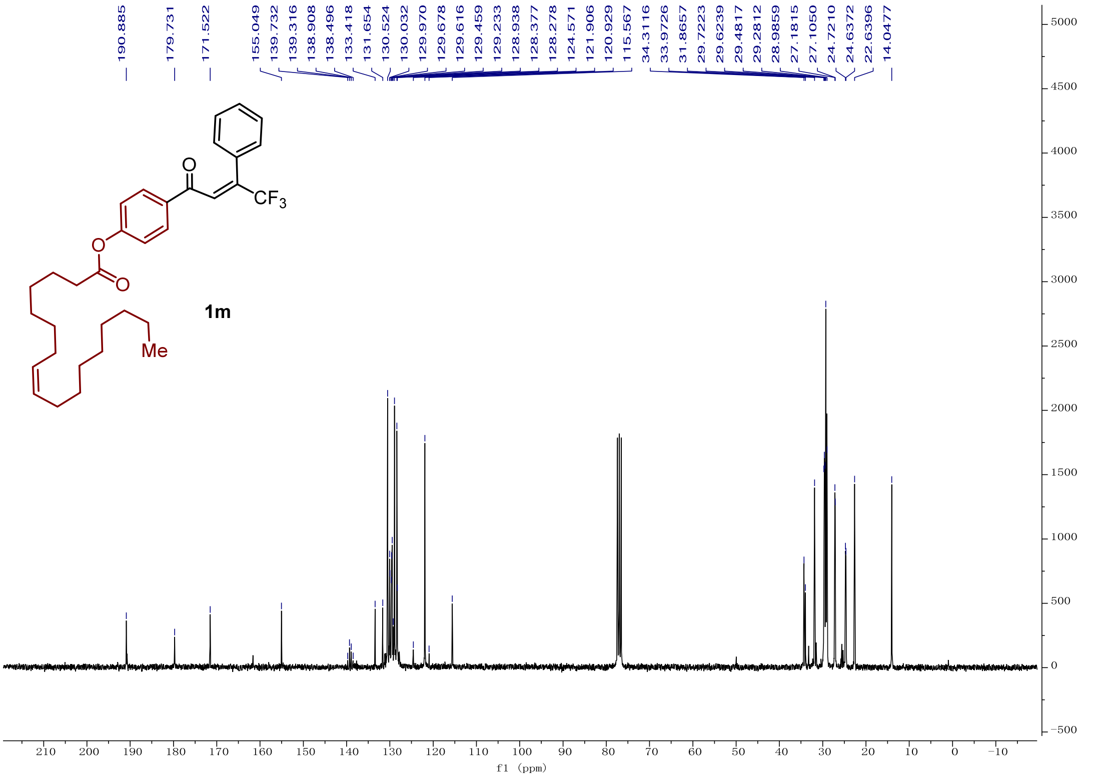

^{19}F NMR (376 MHz, $CDCl_3$) of compound **1m**

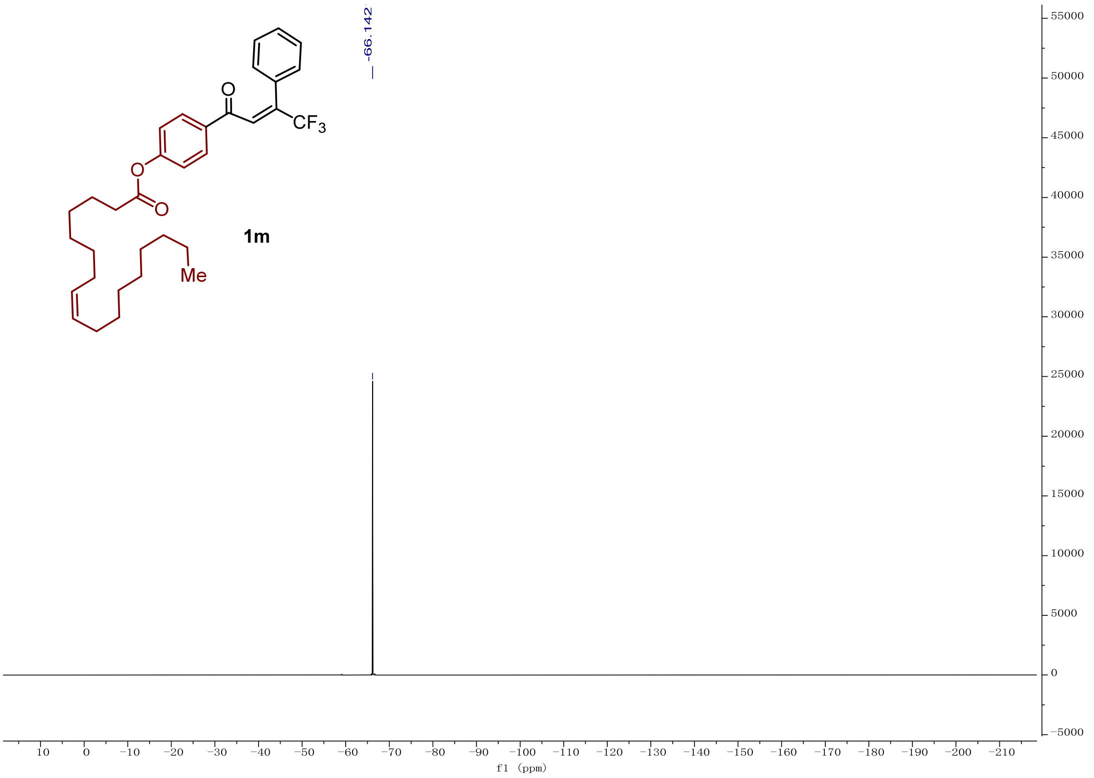

^{1}H NMR (600 MHz, $CDCl_3$) of compound **1n**

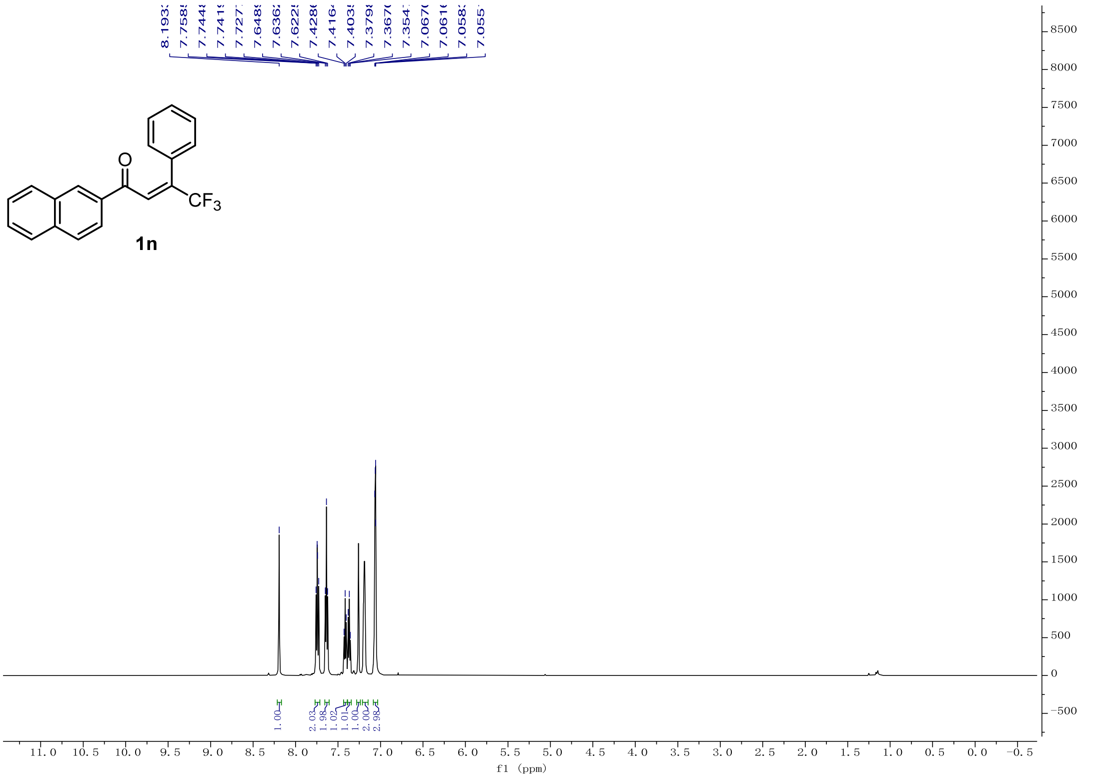

^{13}C NMR (151 MHz, $CDCl_3$) of compound **1n**

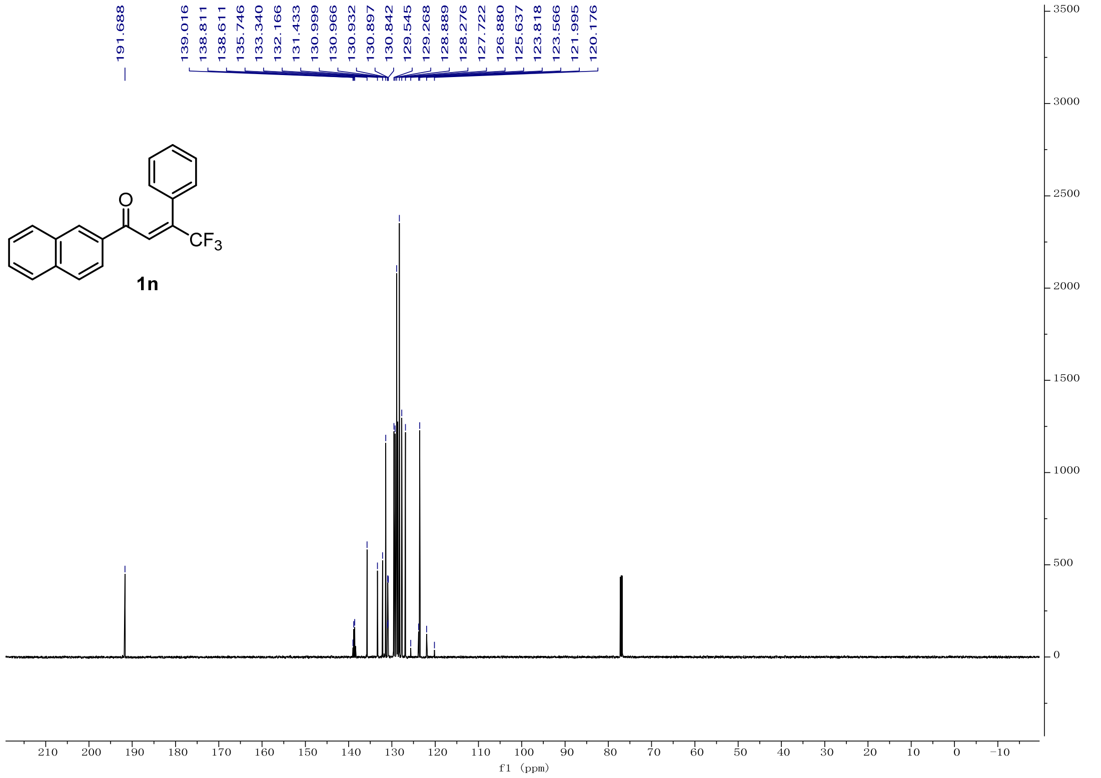

^{19}F NMR (565 MHz, $CDCl_3$) of compound **1n**

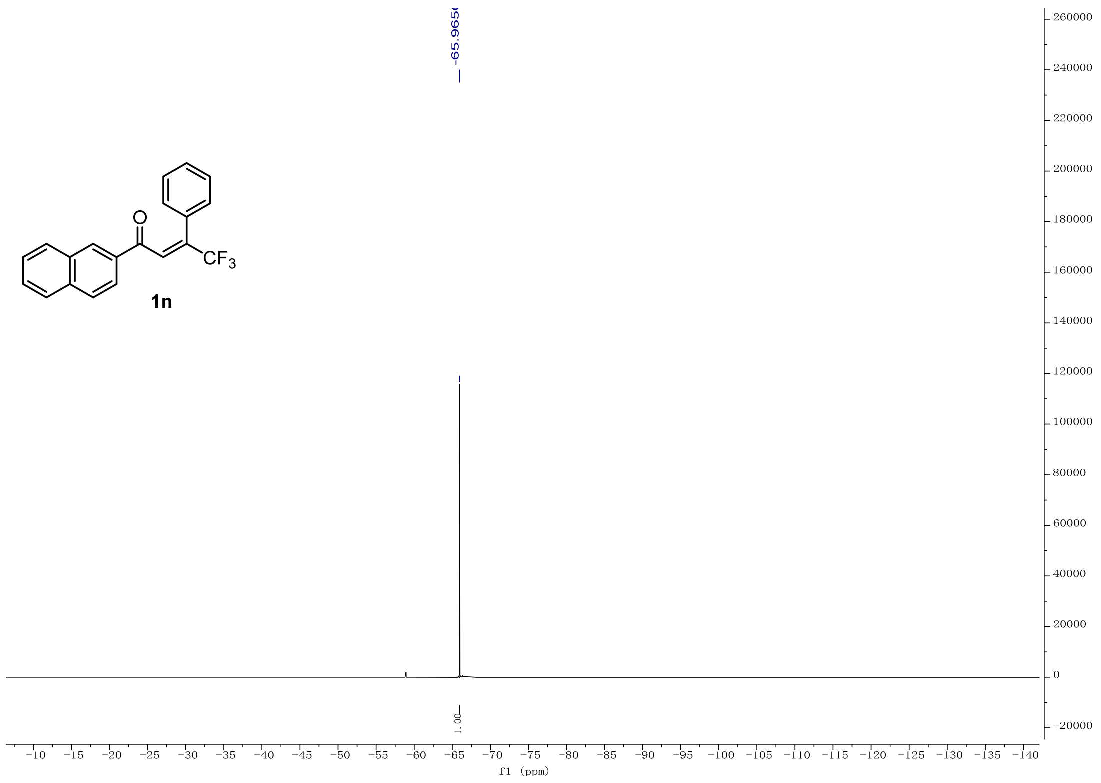

^{1}H NMR (600 MHz, $CDCl_3$) of compound **1o**

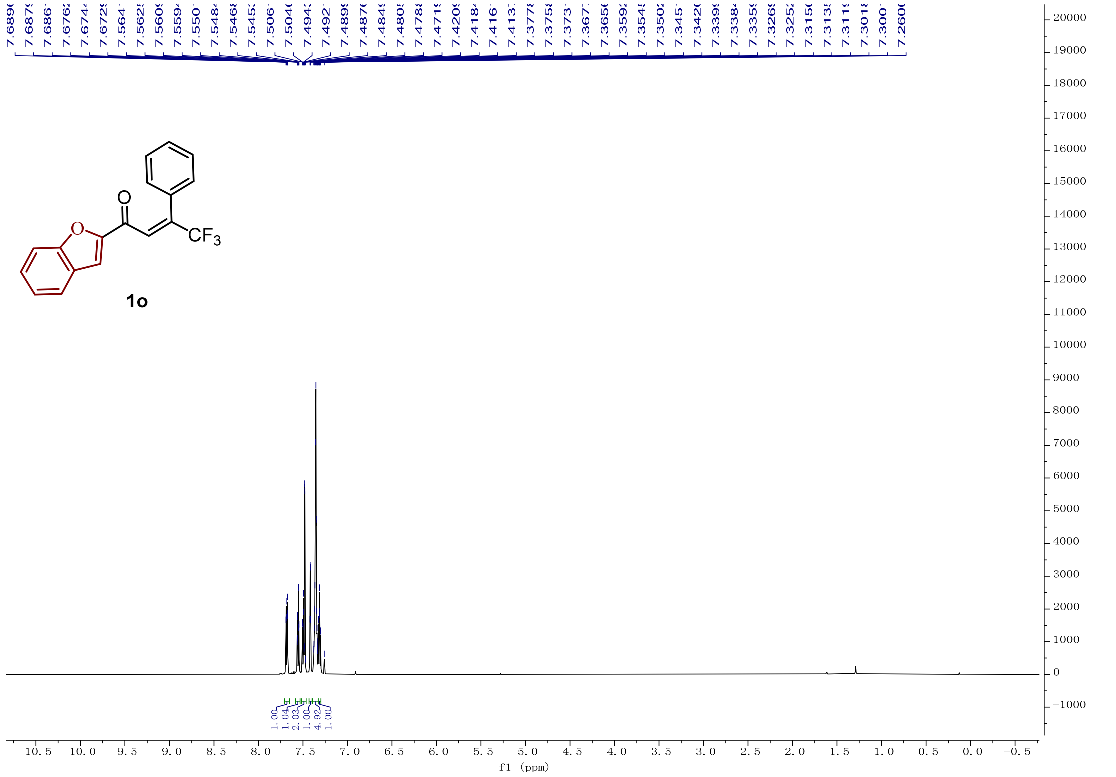

^{13}C NMR (151 MHz, $CDCl_3$) of compound **2o**

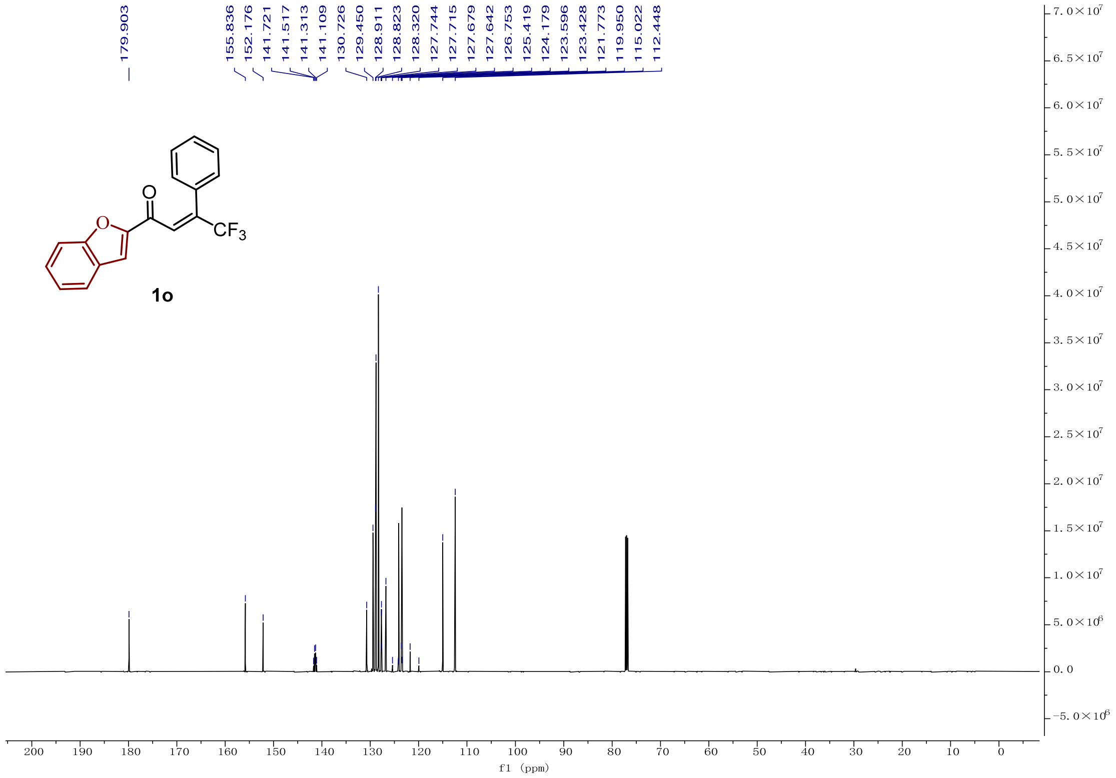

^{19}F NMR (565 MHz, $CDCl_3$) of compound **1o**

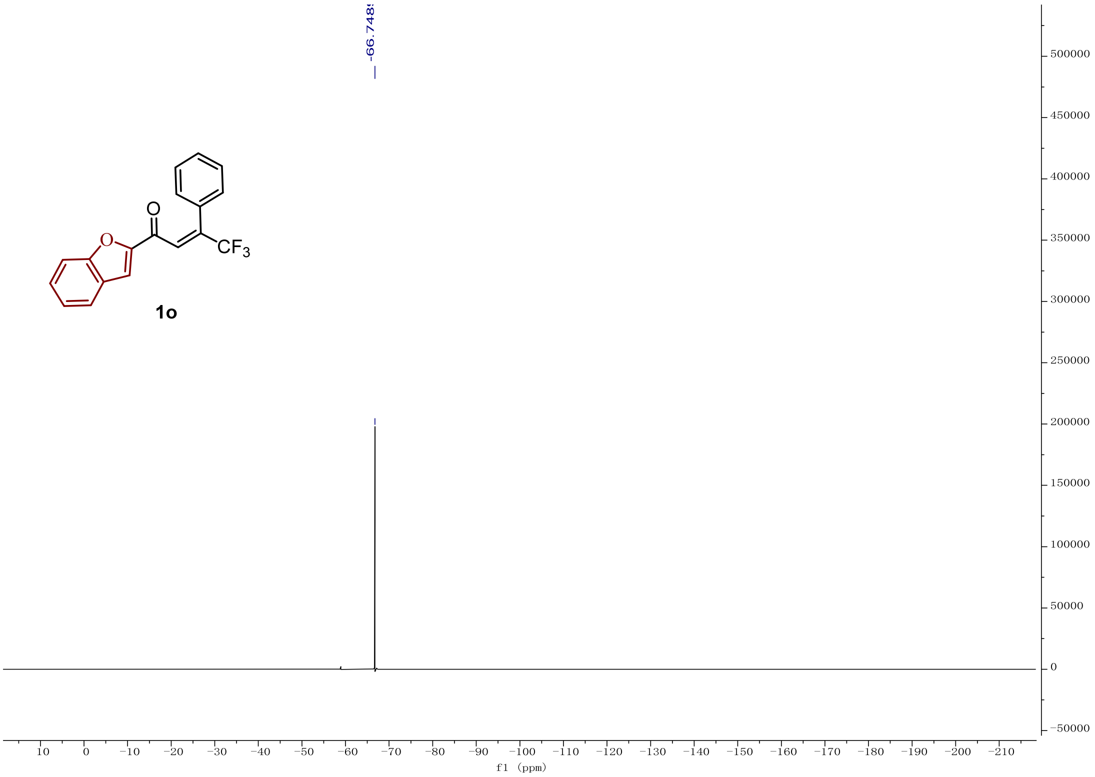

^{1}H NMR (300 MHz, $CDCl_3$) of compound **1p**

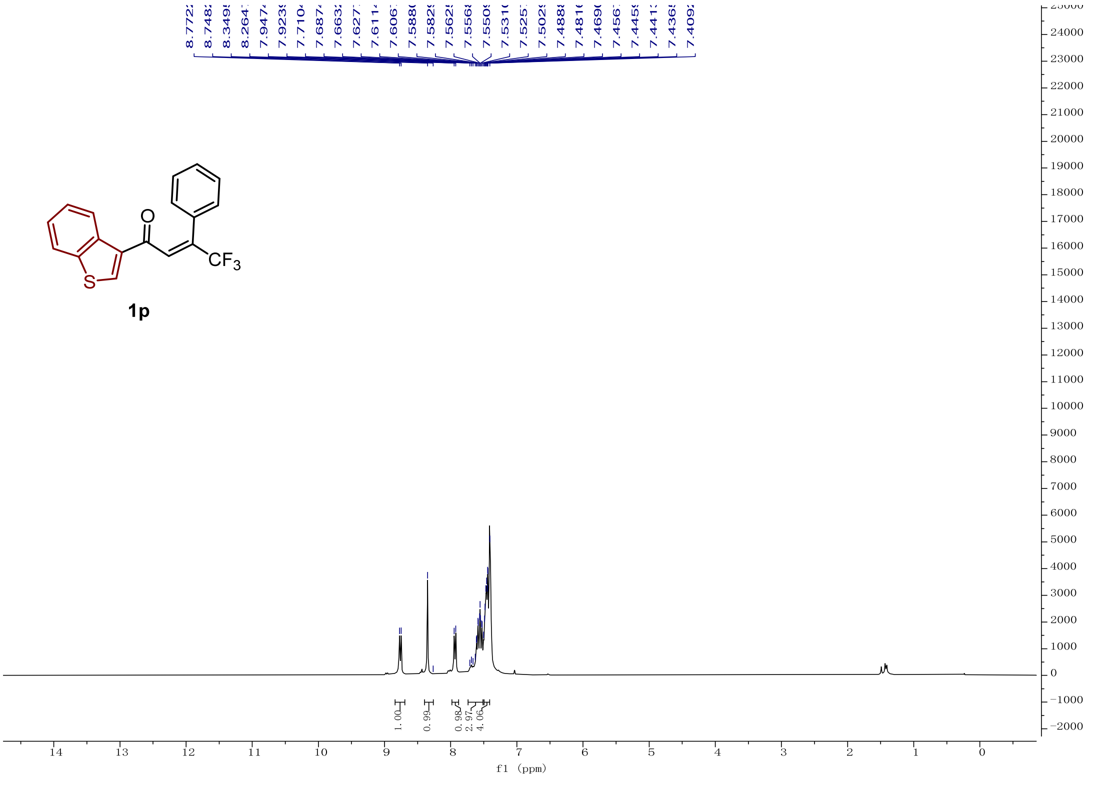

^{13}C NMR (75 MHz, $CDCl_3$) of compound **1p**

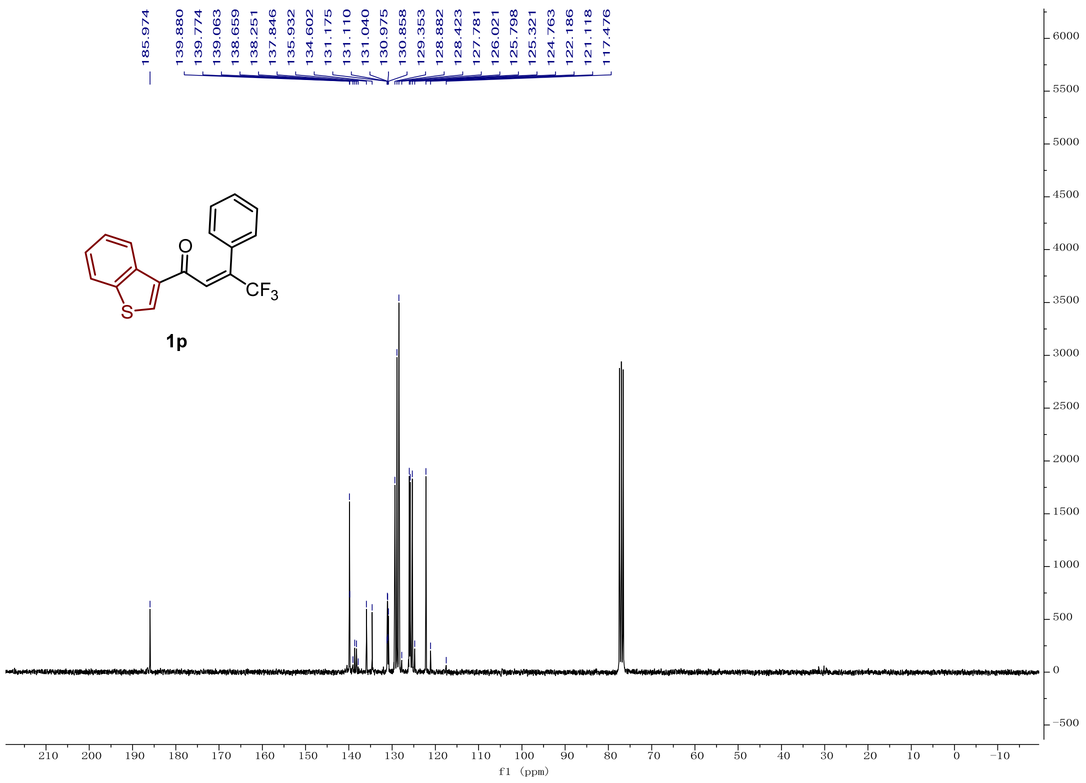

^{19}F NMR (376 MHz, $CDCl_3$) of compound **1p**

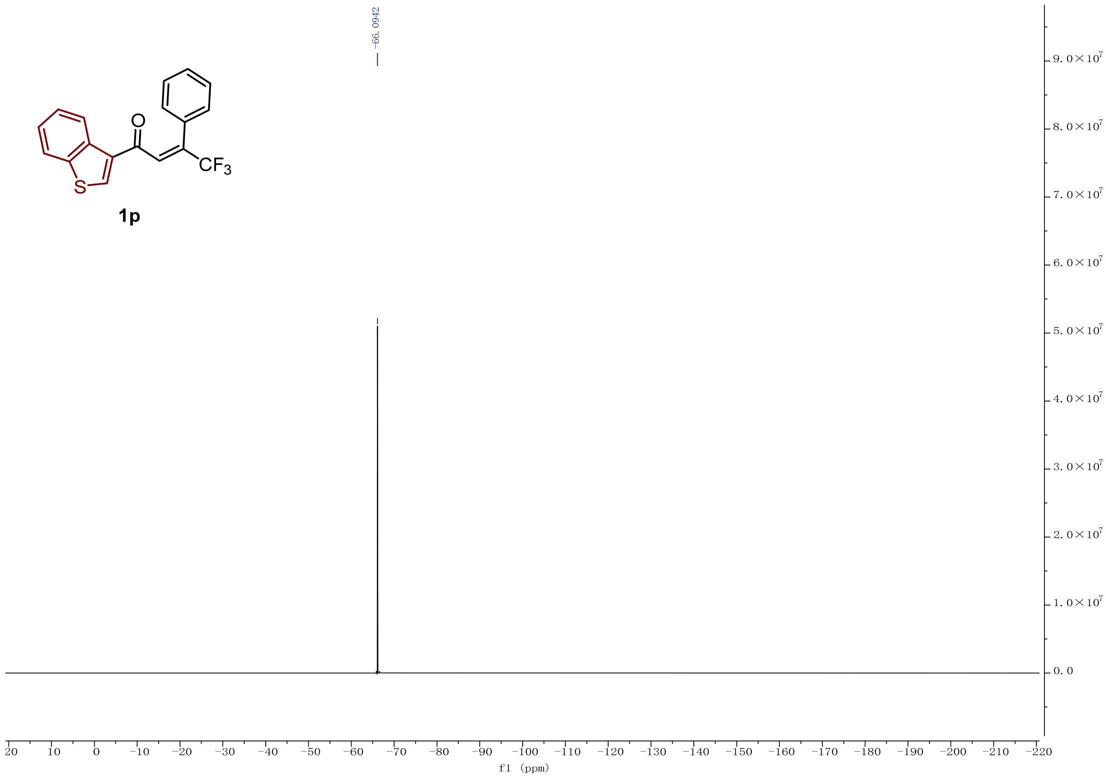

^{1}H NMR (600 MHz, $CDCl_3$) of compound **1q**

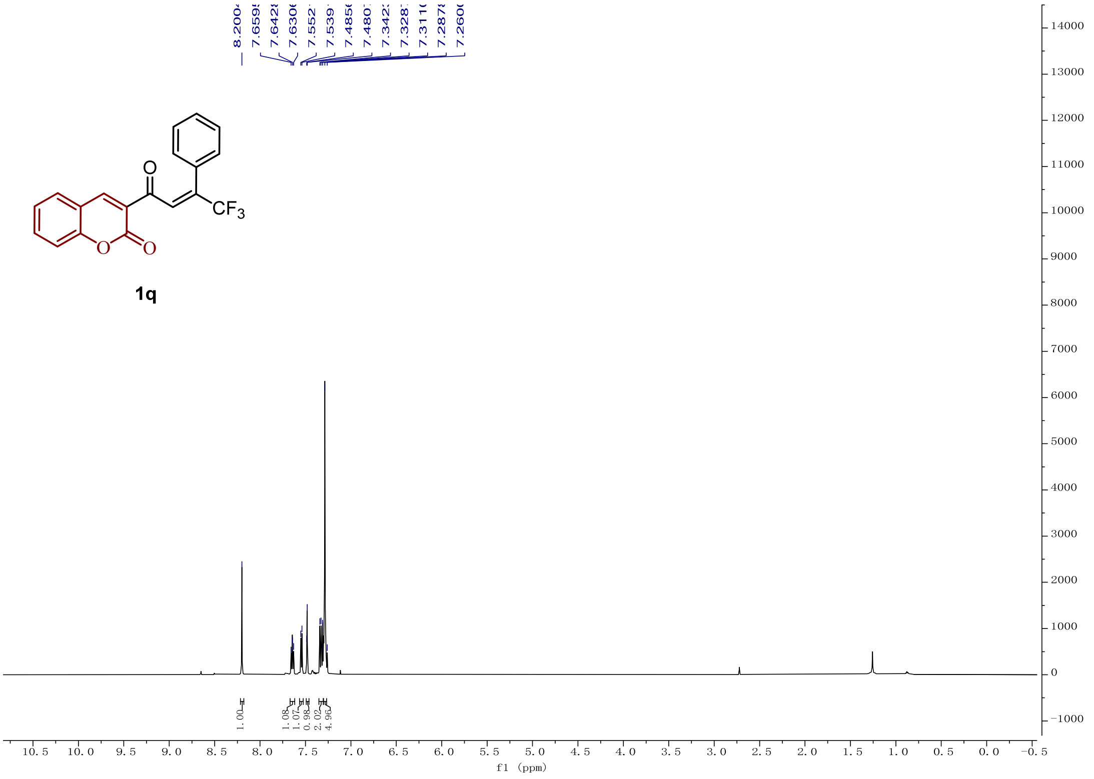

^{13}C NMR (151 MHz, $CDCl_3$) of compound **1q**

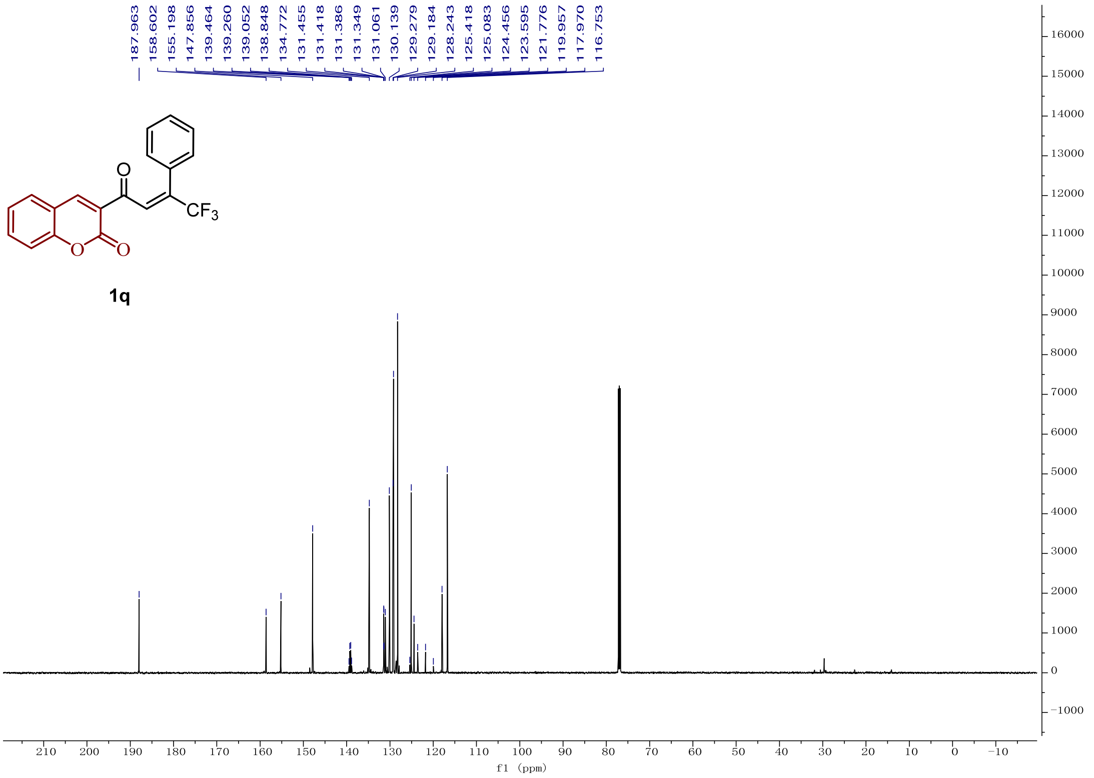

^{19}F NMR (565 MHz, $CDCl_3$) of compound **1q**

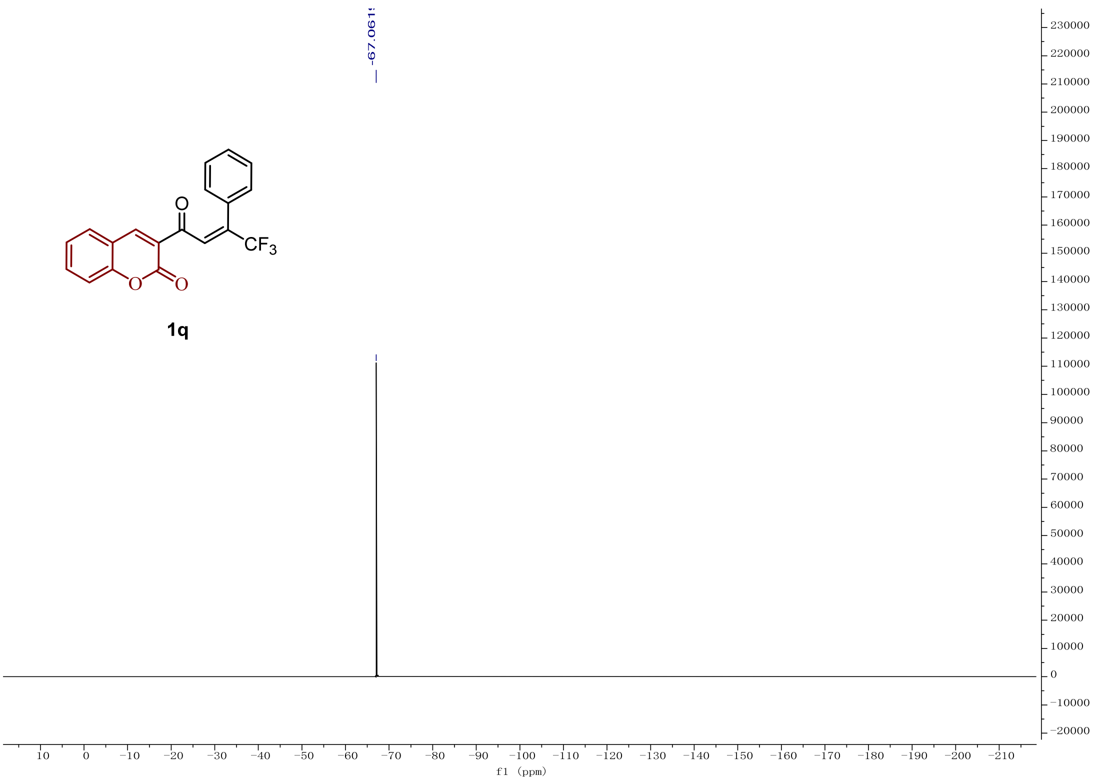

^{1}H NMR (300 MHz, $CDCl_3$) of compound **1r**

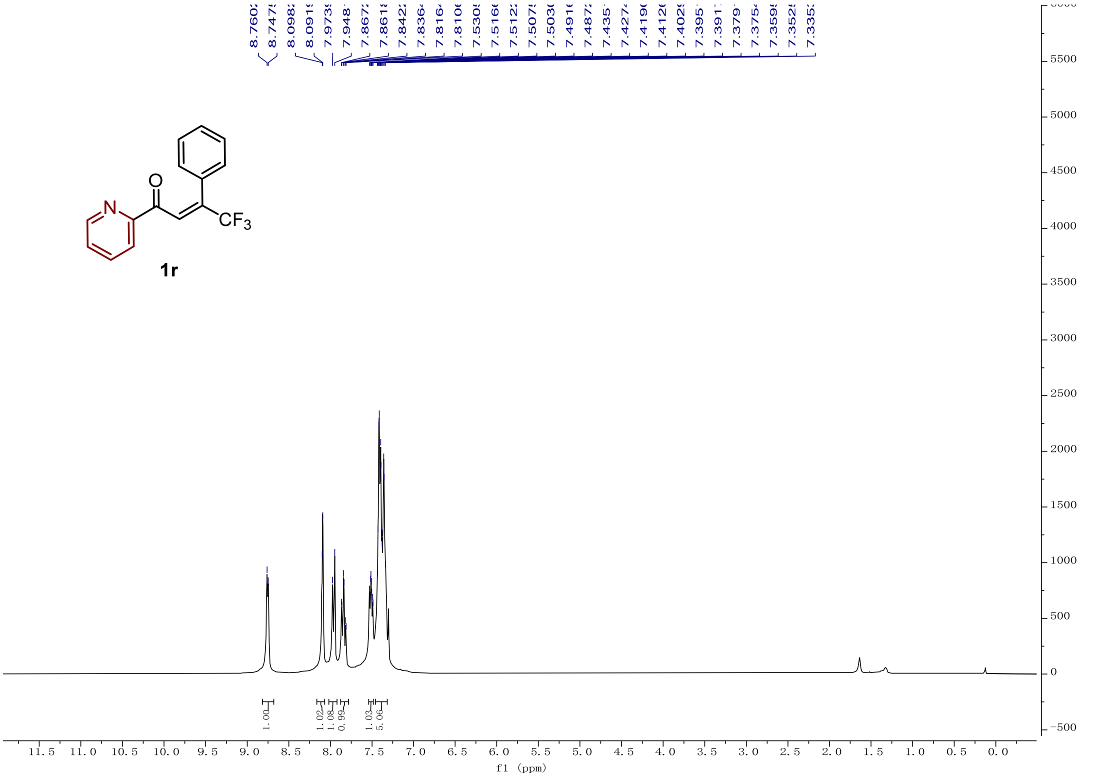

^{13}C NMR (75 MHz, $CDCl_3$) of compound **1r**

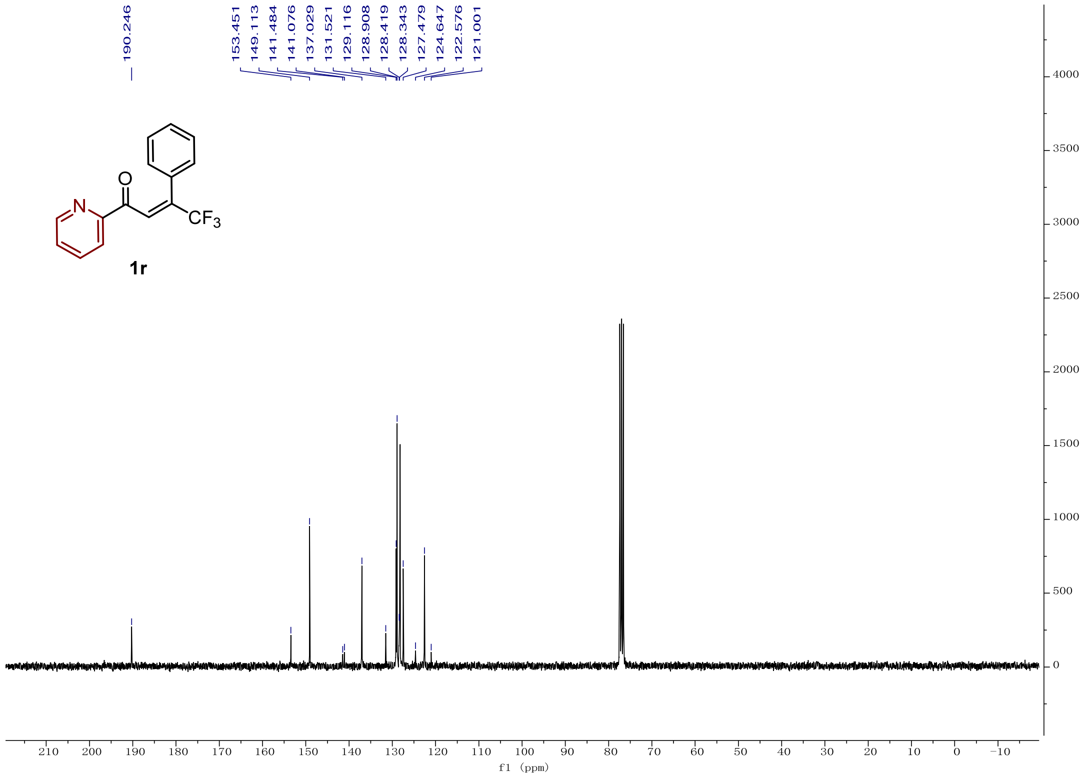

^{19}F NMR (376 MHz, $CDCl_3$) of compound **1r**

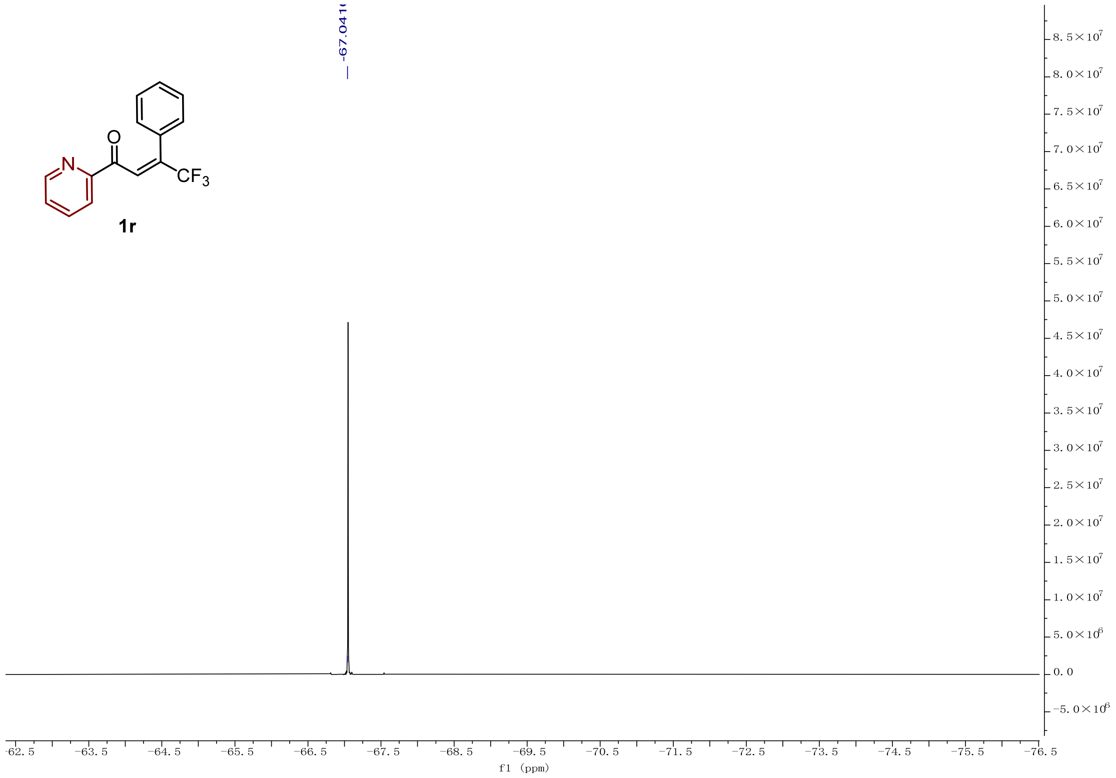

^{1}H NMR (300 MHz, $CDCl_3$) of compound **1t**

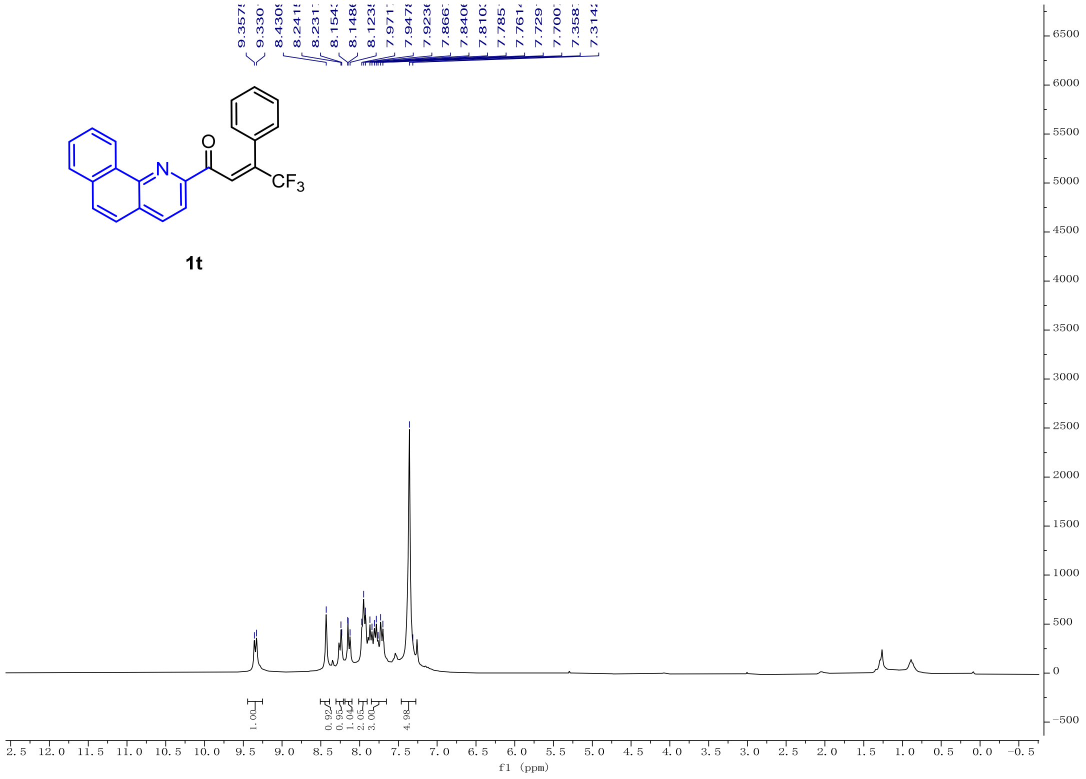

^{13}C NMR (75 MHz, $CDCl_3$) of compound **1t**

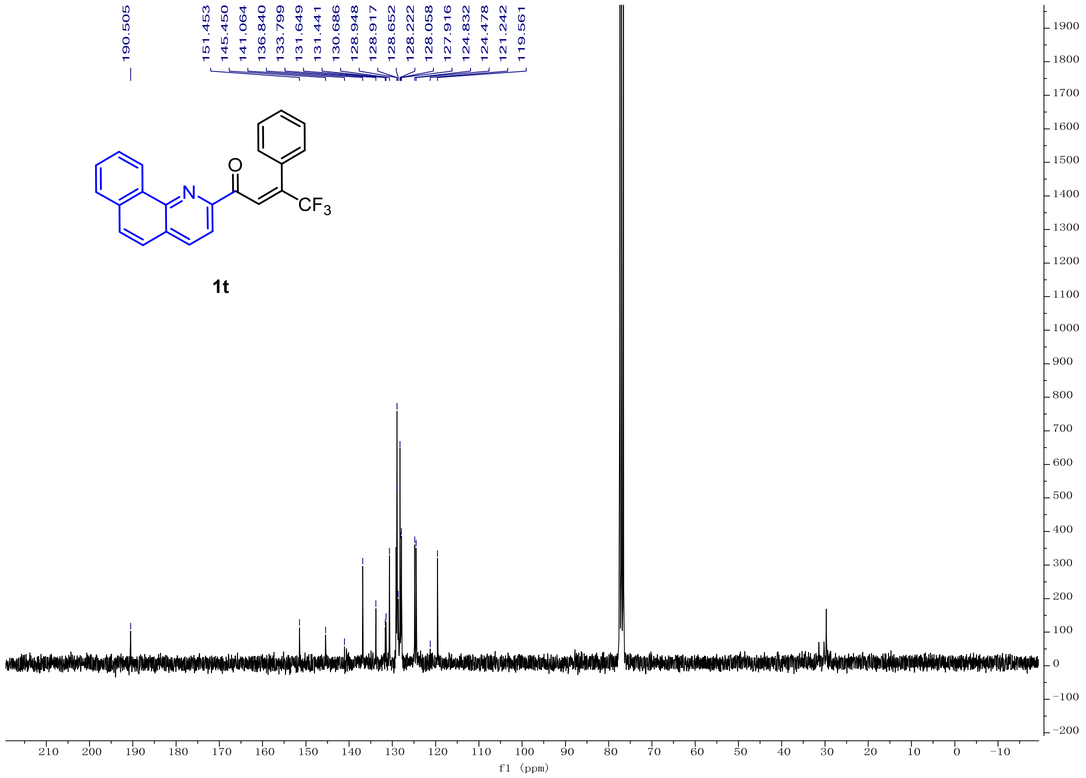

^{19}F NMR (376 MHz, $CDCl_3$) of compound **1t**

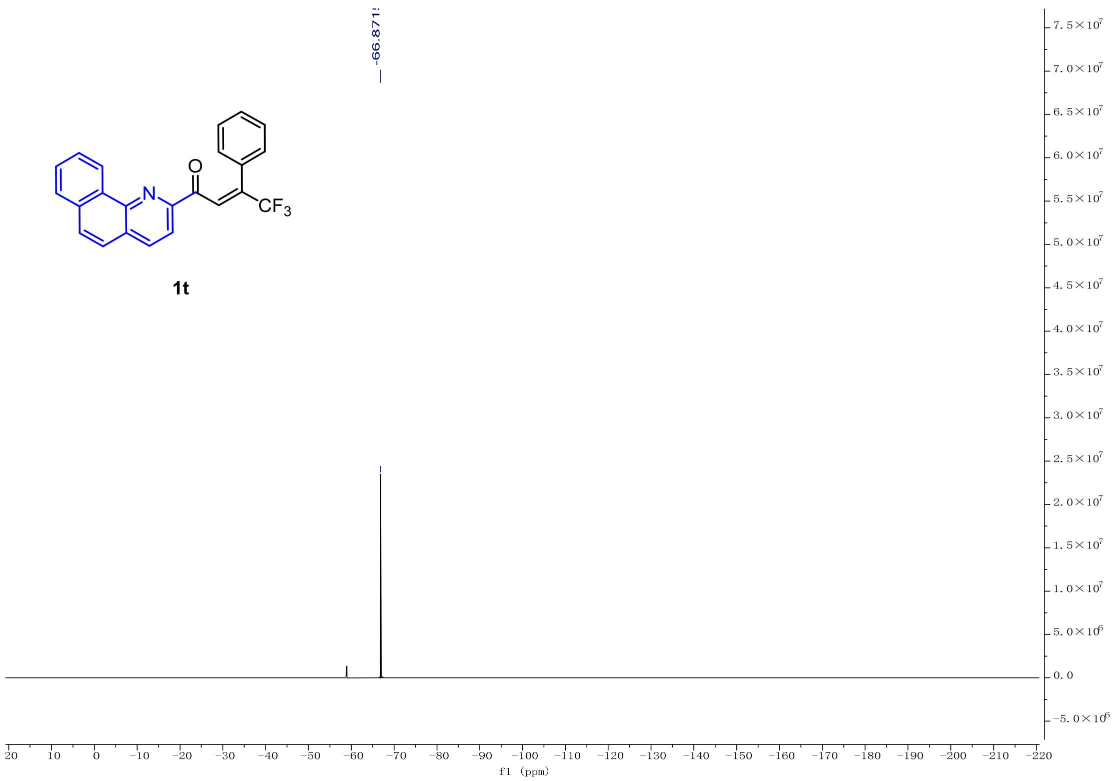

^{1}H NMR (300 MHz, $CDCl_3$) of compound **1u**

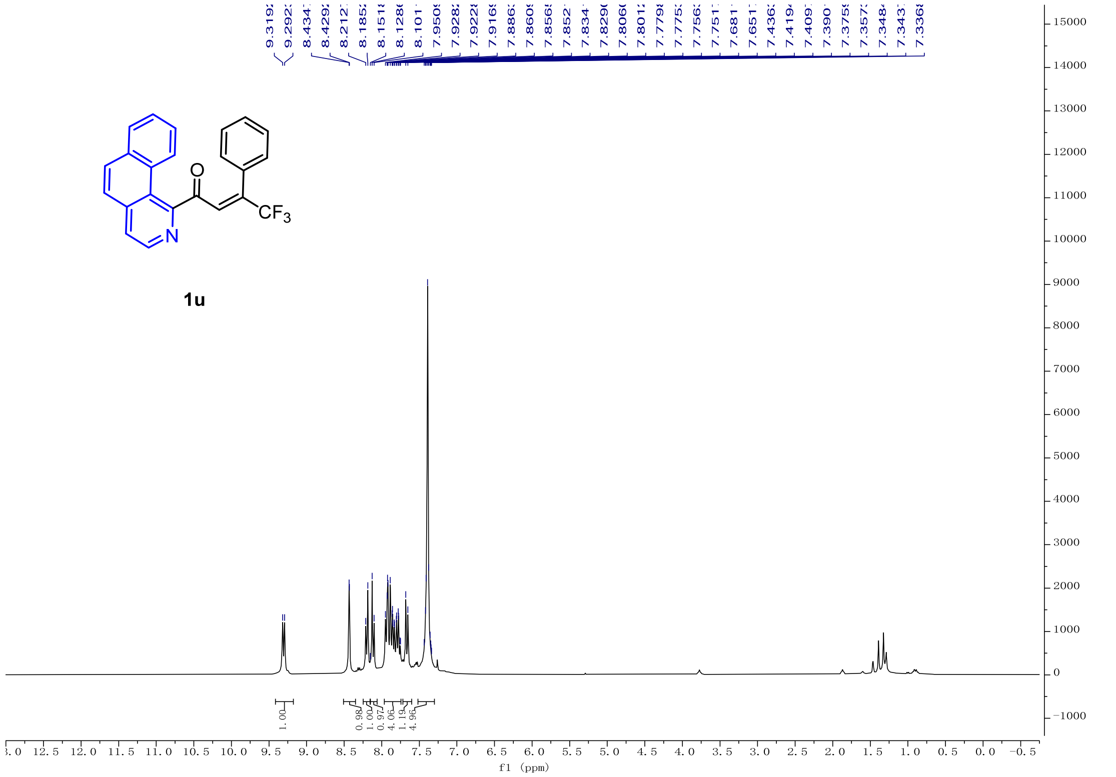

^{13}C NMR (151 MHz, $CDCl_3$) of compound **1u**

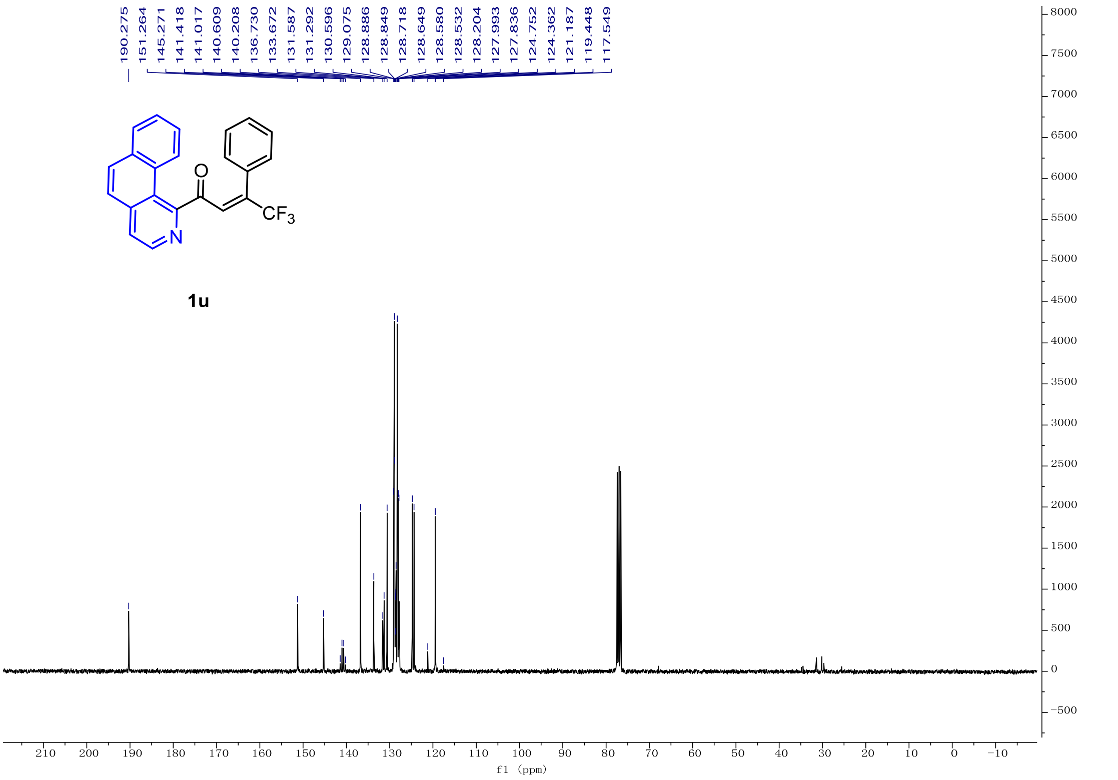

^{19}F NMR (376 MHz, $CDCl_3$) of compound **1u**

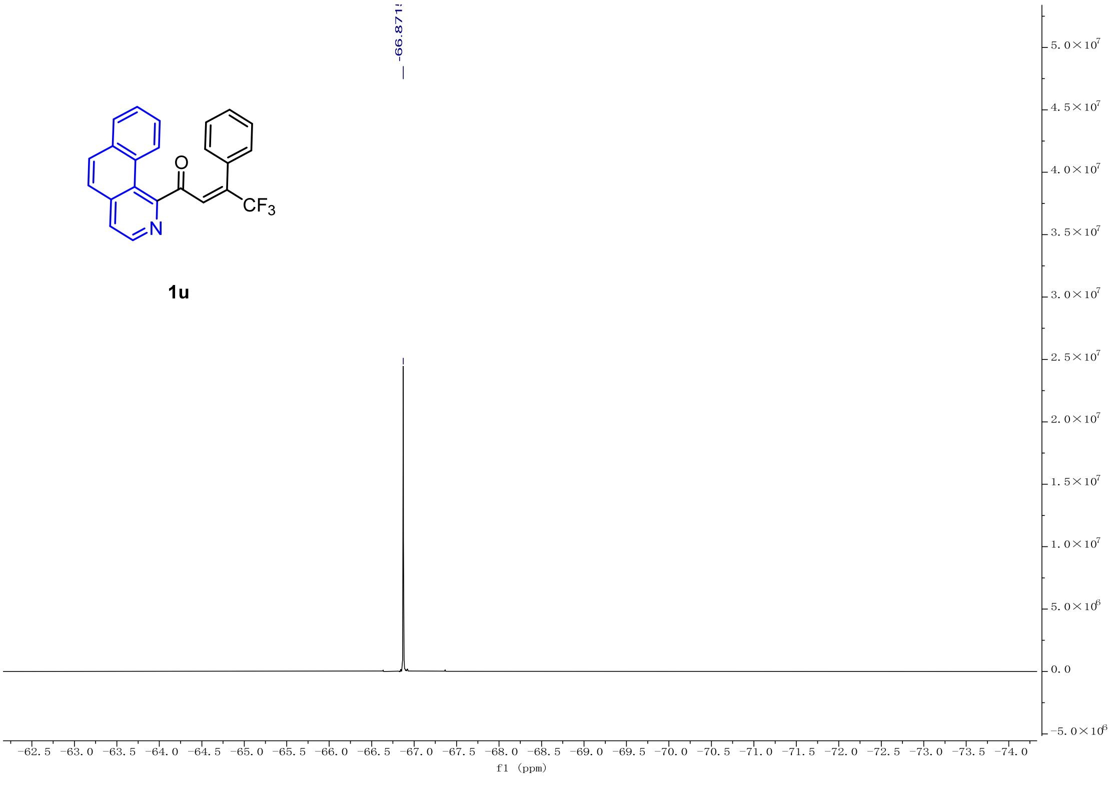

^{1}H NMR (600 MHz, $CDCl_3$) of compound **1v**

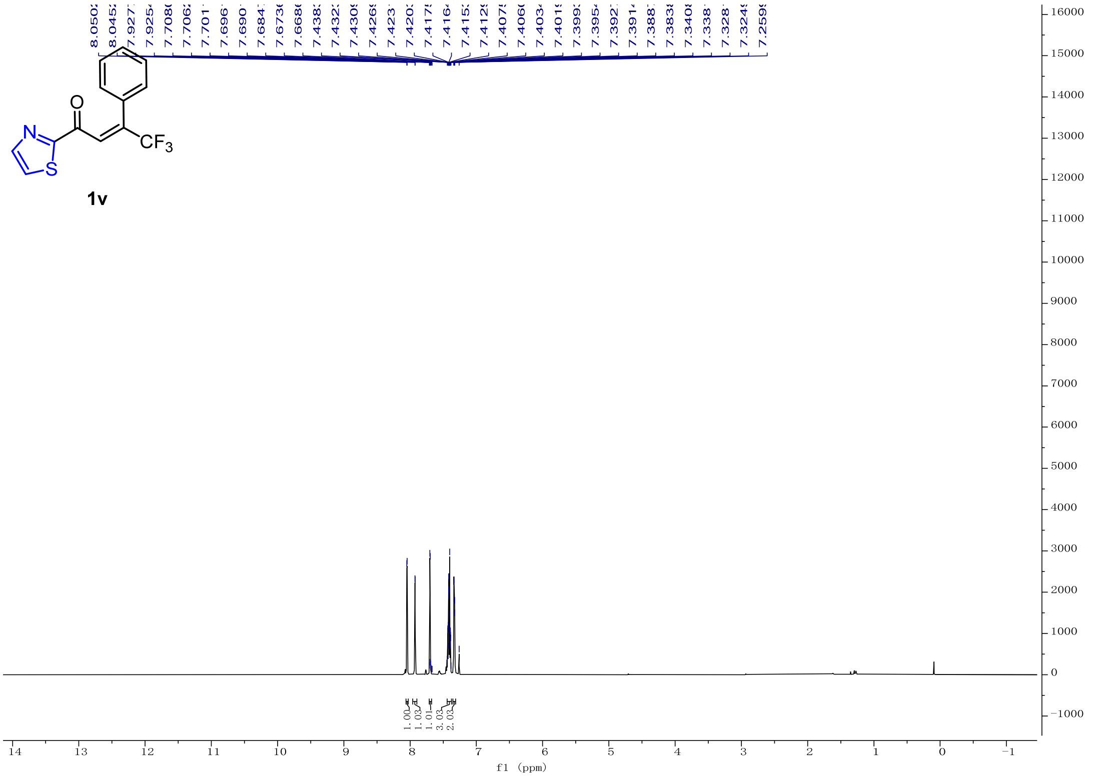

13C NMR (151 MHz, $CDCl_3$) of compound **1v**

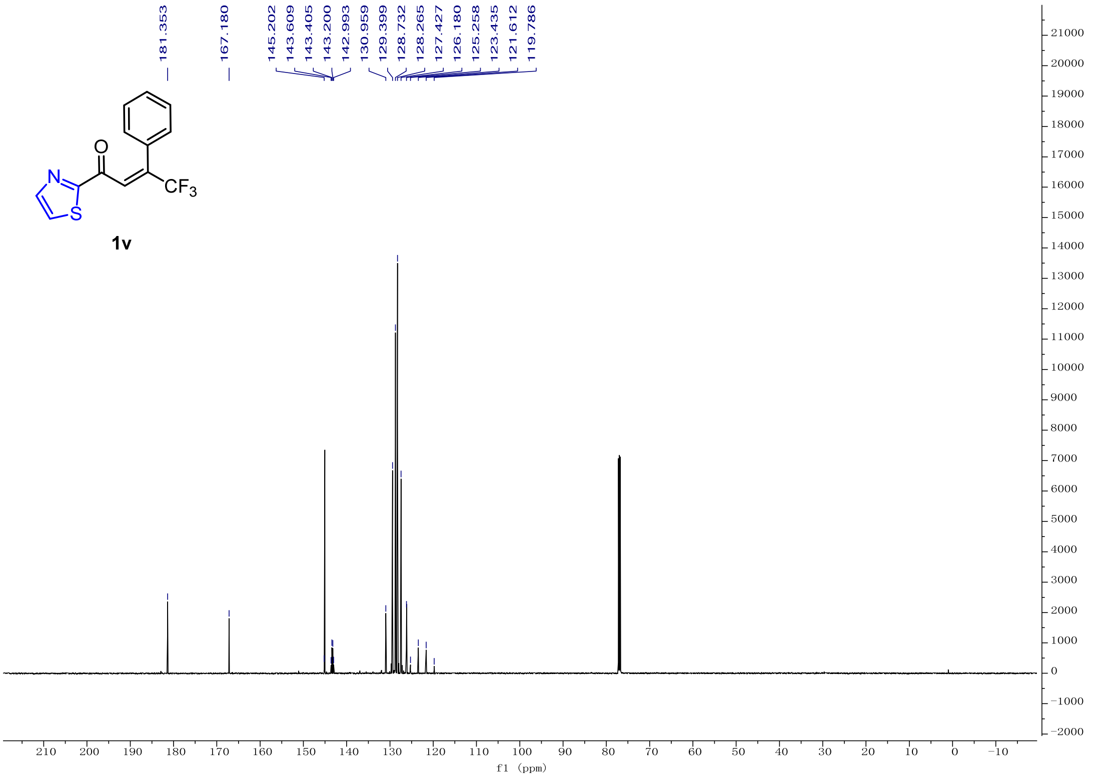

^{19}F NMR (565 MHz, $CDCl_3$) of compound **1v**

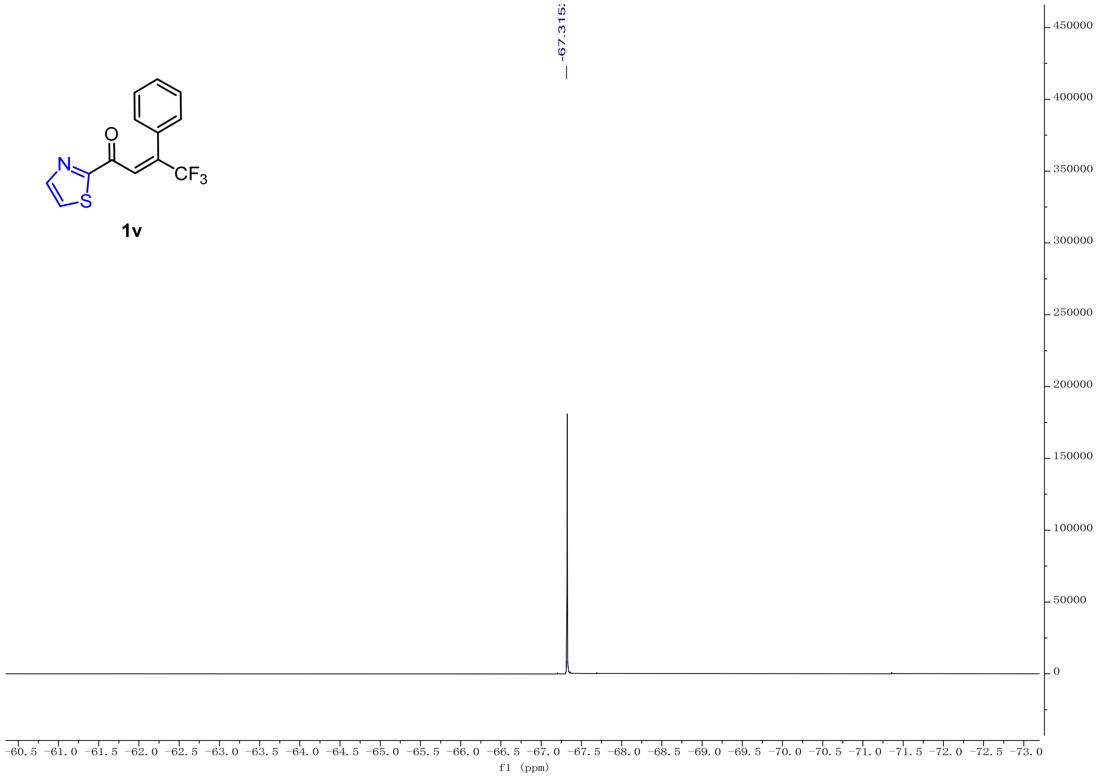

^{1}H NMR (300 MHz, $CDCl_3$) of compound **1w**

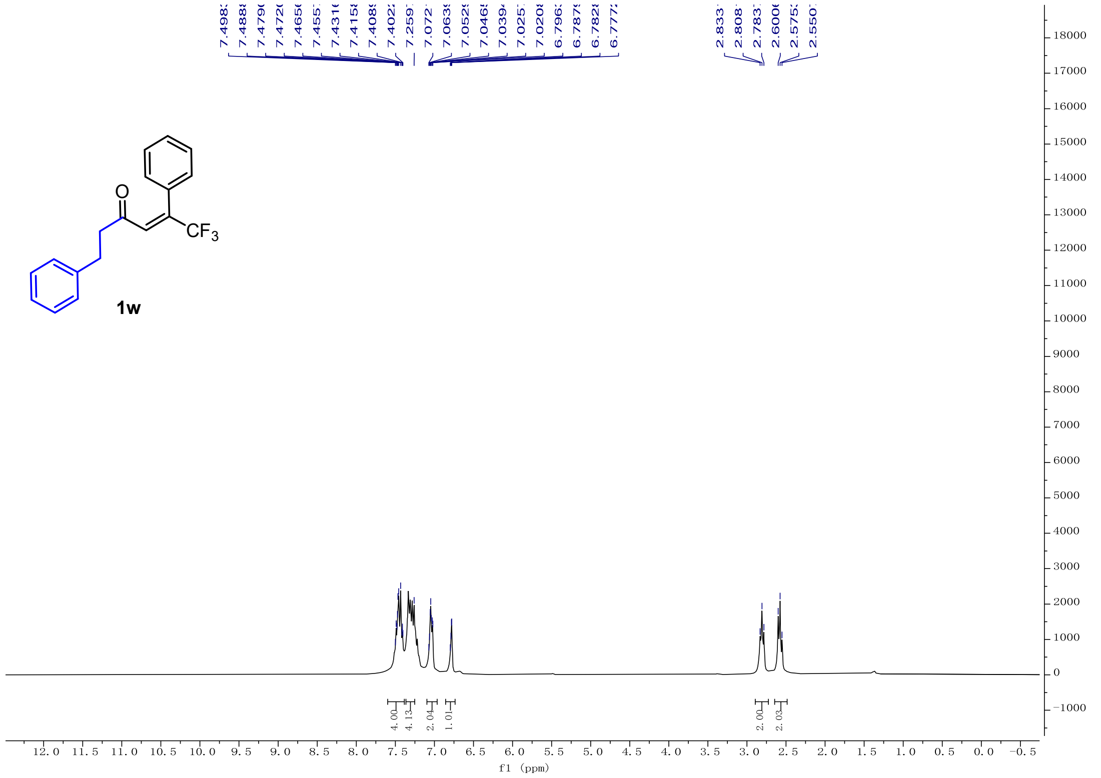

^{13}C NMR (75 MHz, $CDCl_3$) of compound **1w**

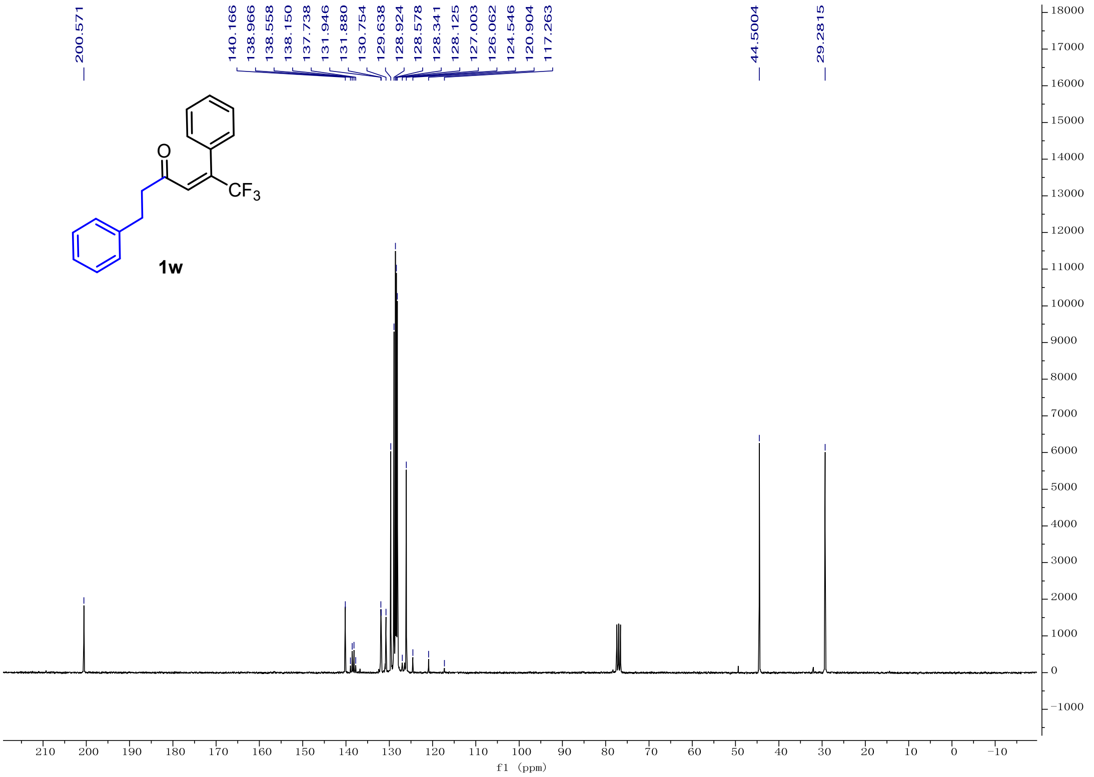

^{19}F NMR (376 MHz, $CDCl_3$) of compound **1w**

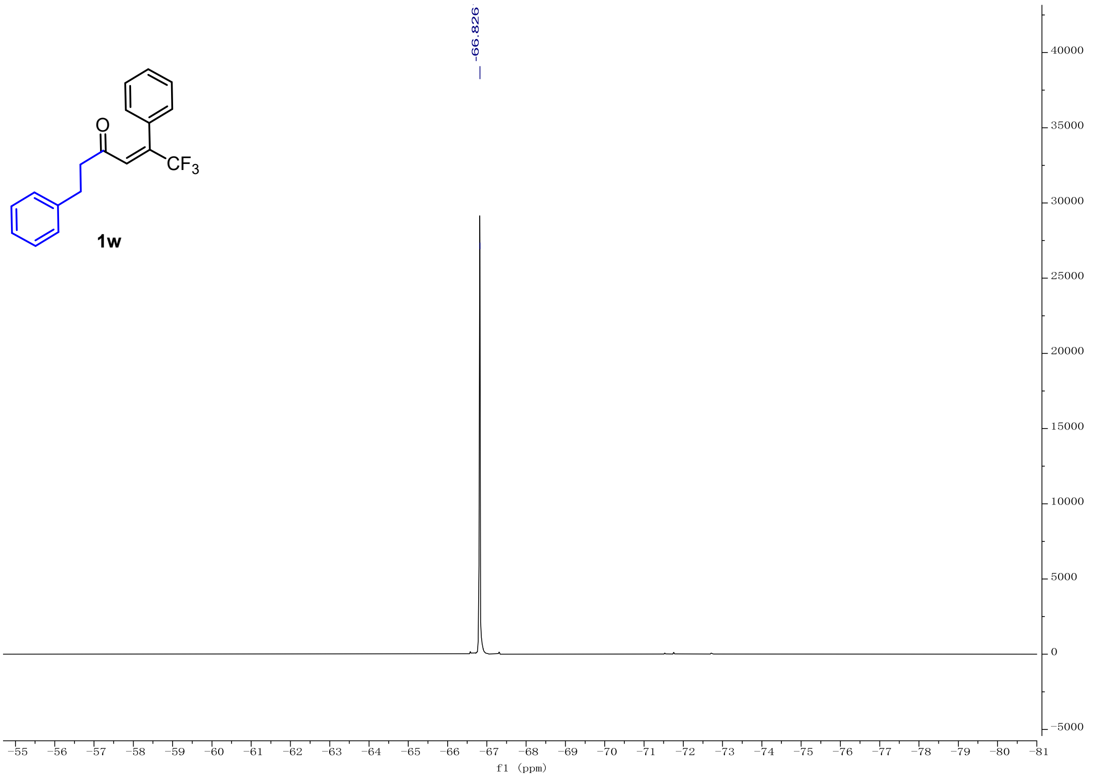

^{1}H NMR (300 MHz, $CDCl_3$) of compound **1x**

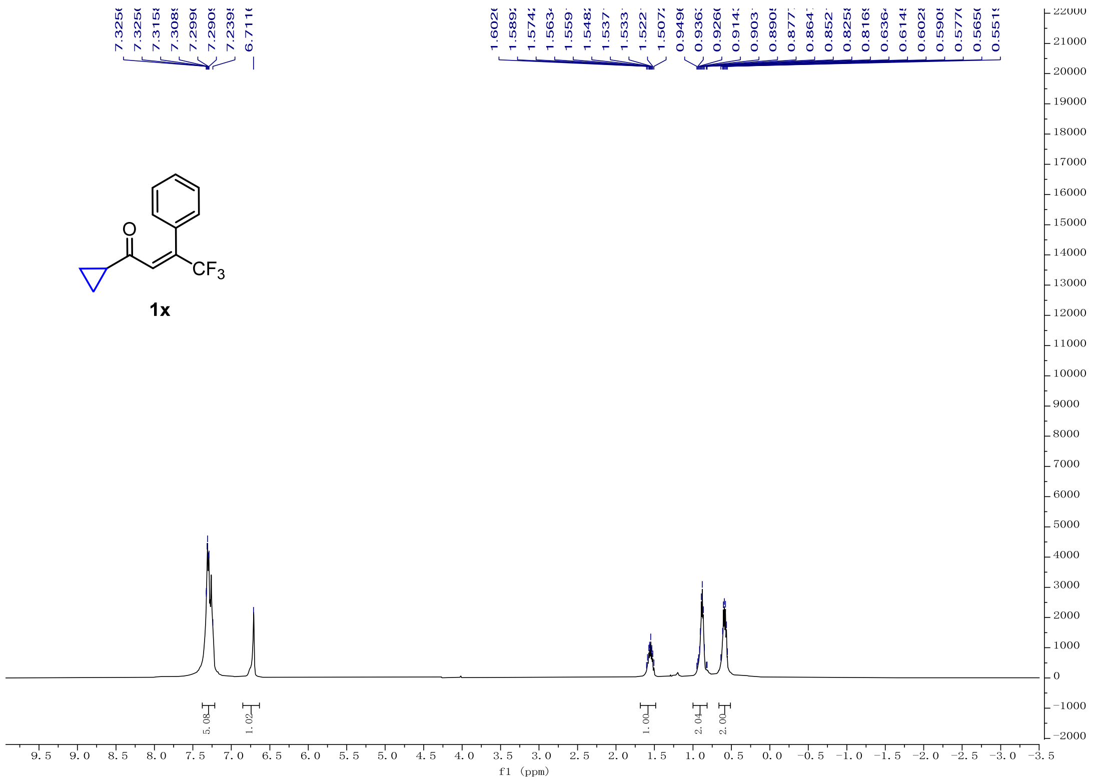

^{13}C NMR (75 MHz, $CDCl_3$) of compound **1x**

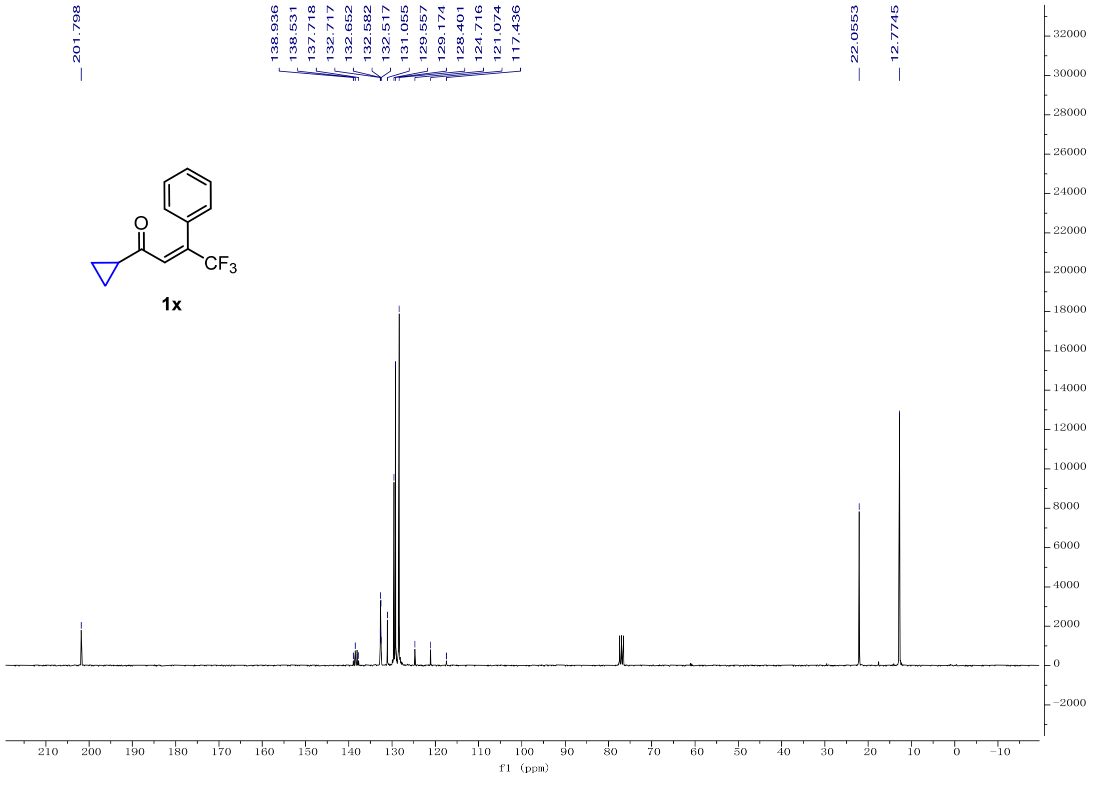

^{19}F NMR (376 MHz, $CDCl_3$) of compound **1x**

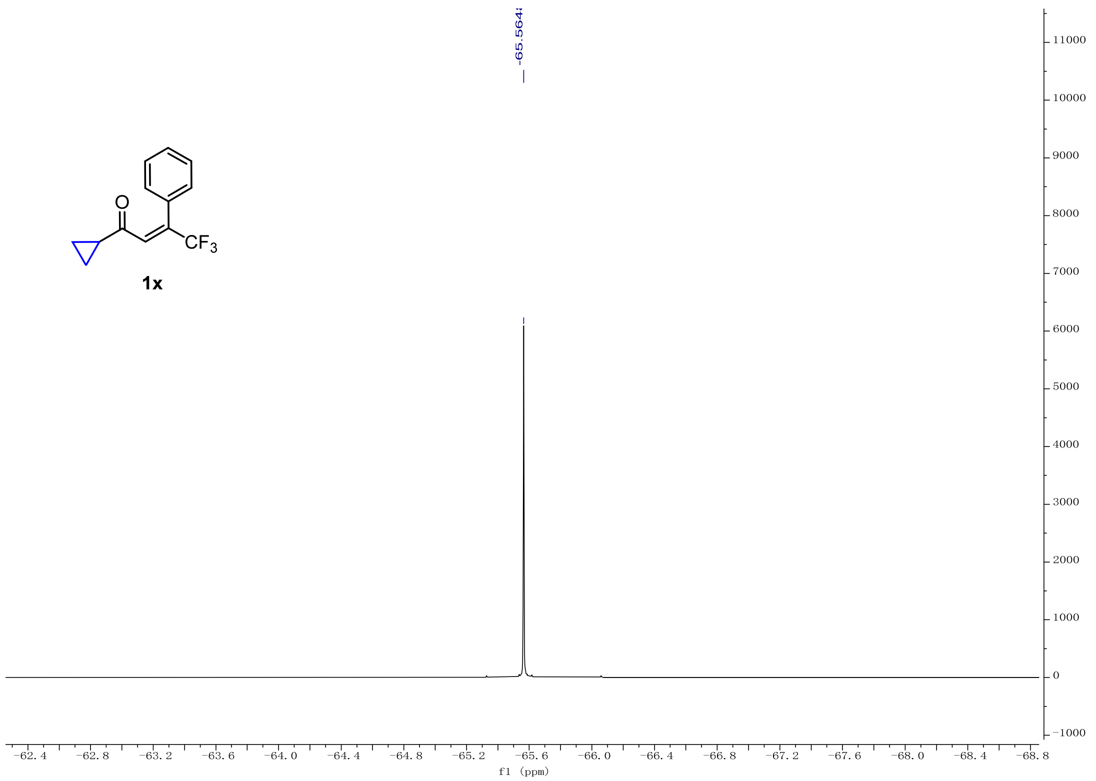

^{1}H NMR (300 MHz, $CDCl_3$) of compound **1y**

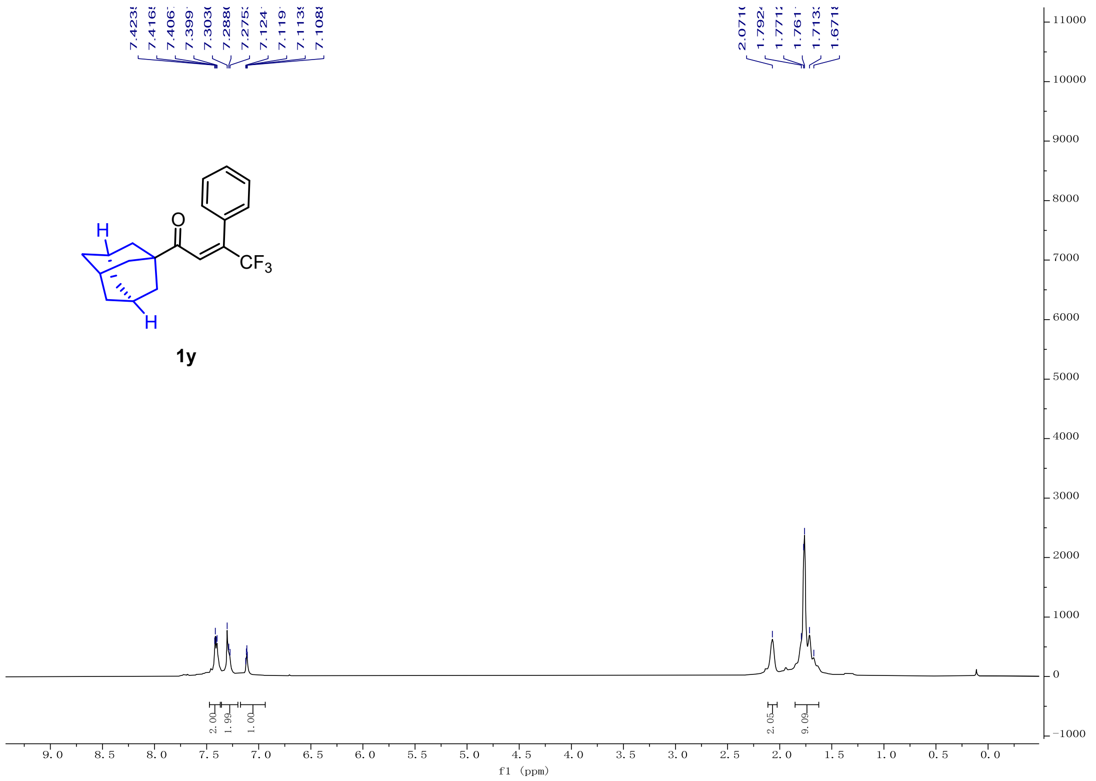

^{13}C NMR (75 MHz, $CDCl_3$) of compound **1y**

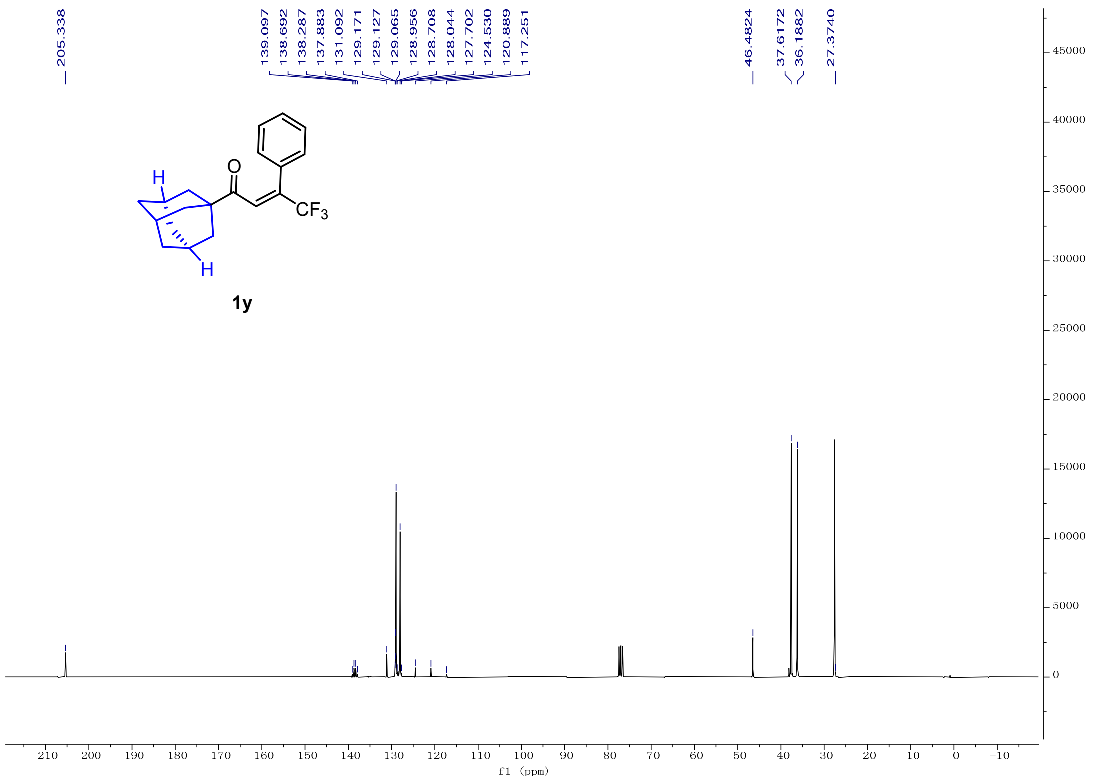

^{19}F NMR (376 MHz, $CDCl_3$) of compound **1y**

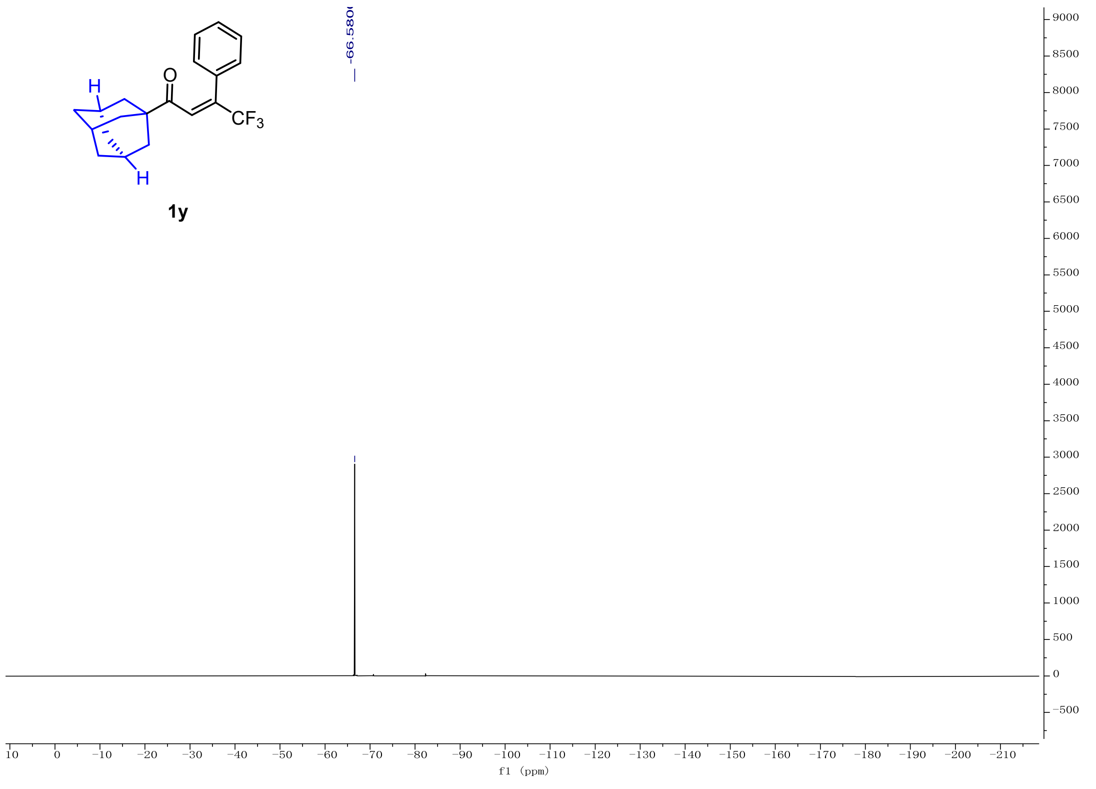

^{1}H NMR (300 MHz, $CDCl_3$) of compound **1za**

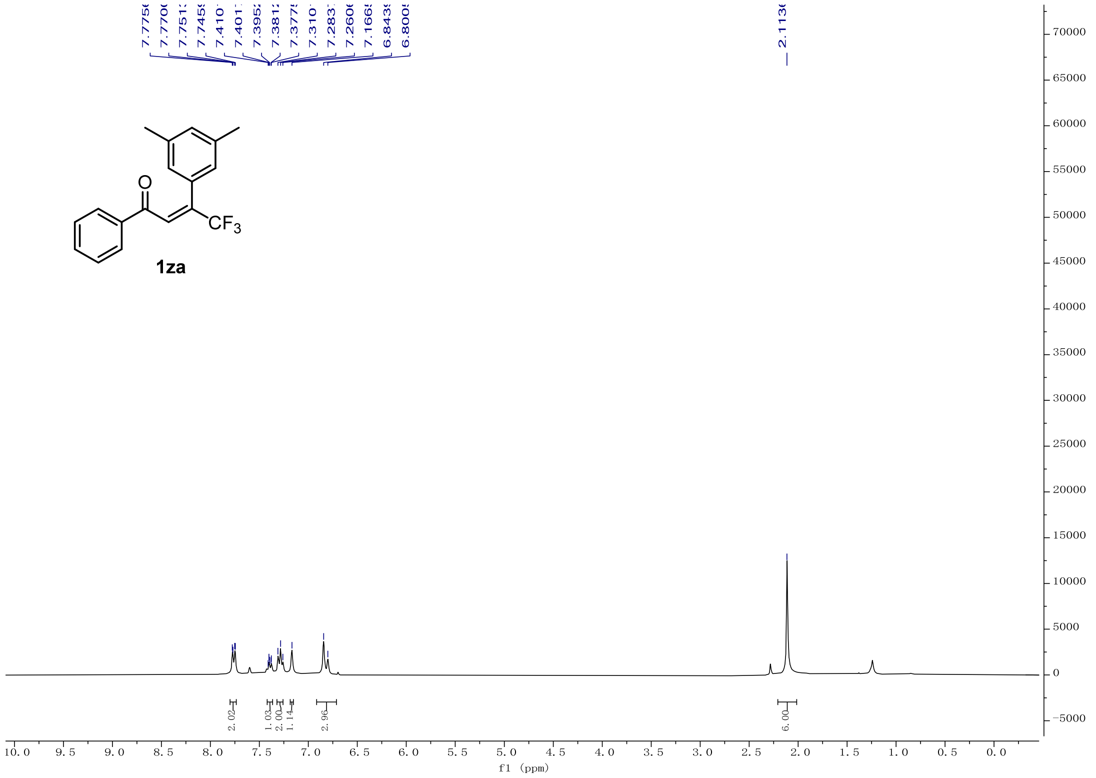

^{13}C NMR (75 MHz, $CDCl_3$) of compound **1za**

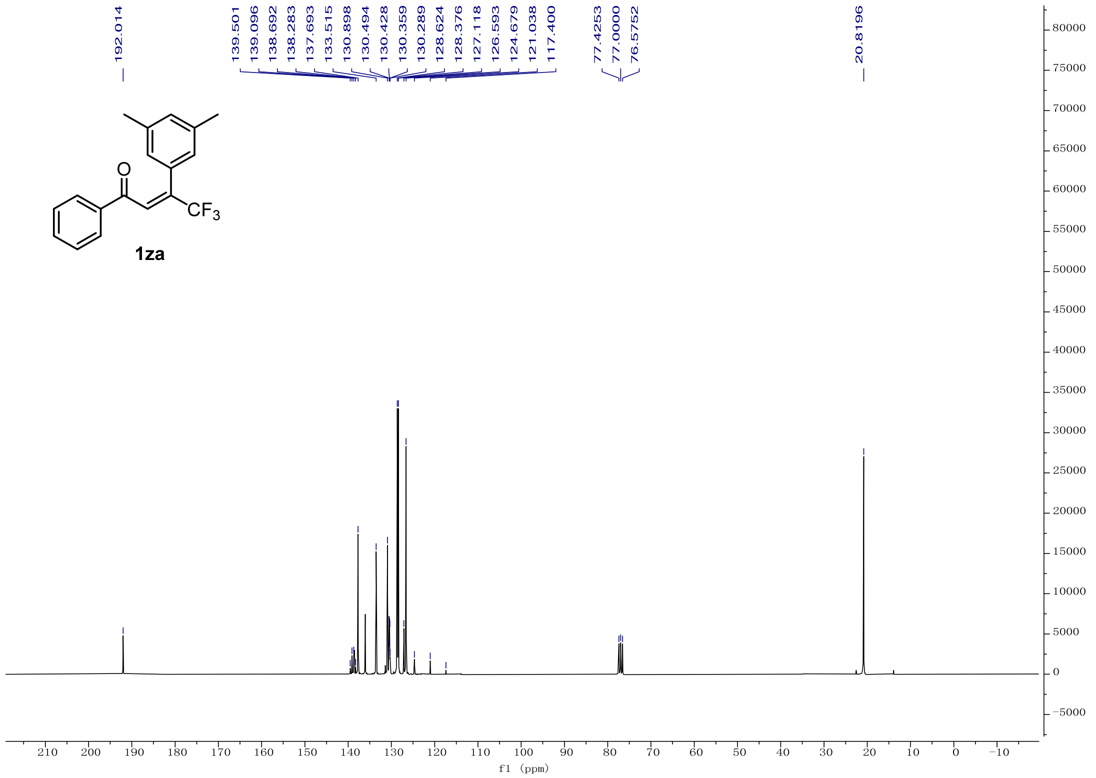

^{19}F NMR (376 MHz, $CDCl_3$) of compound **1za**

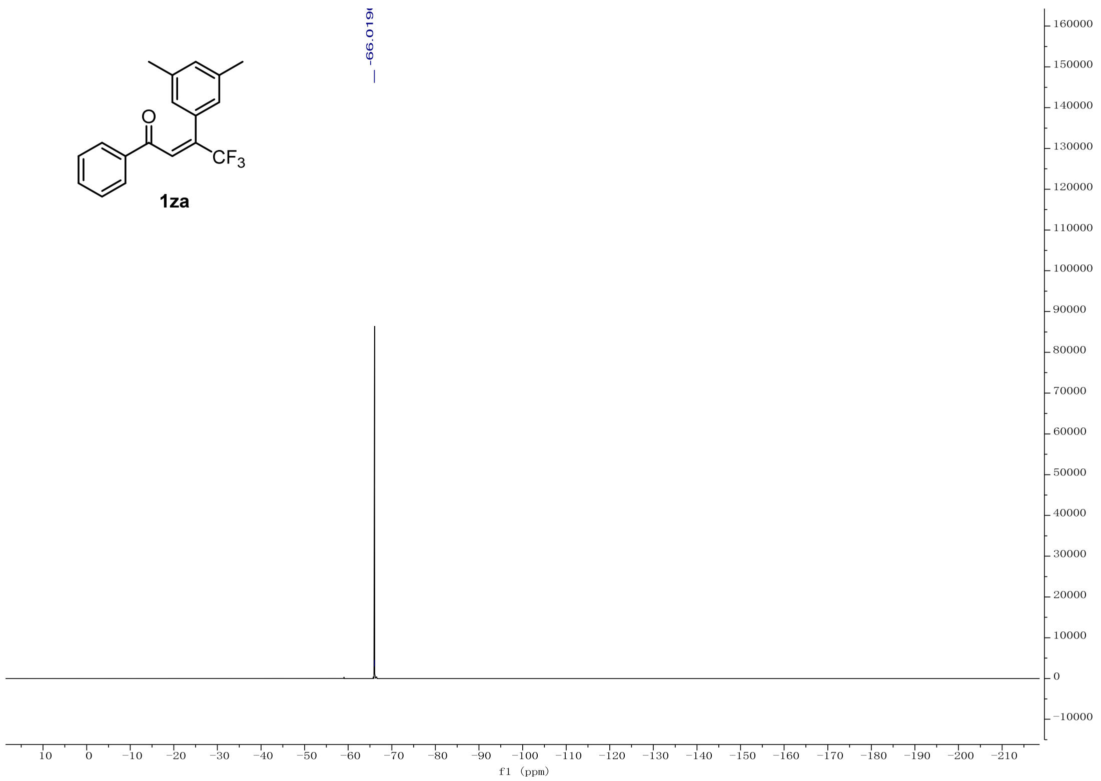

^{1}H NMR (300 MHz, $CDCl_3$) of compound **1zb**

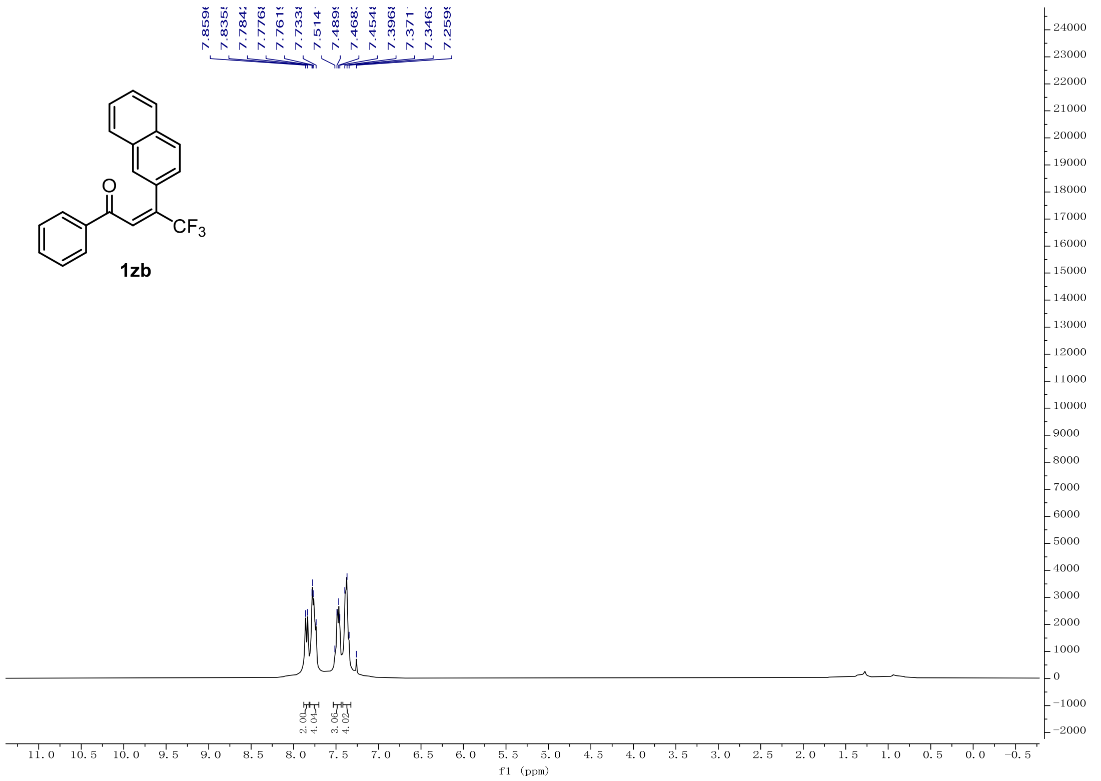

^{13}C NMR (75 MHz, $CDCl_3$) of compound **1zb**

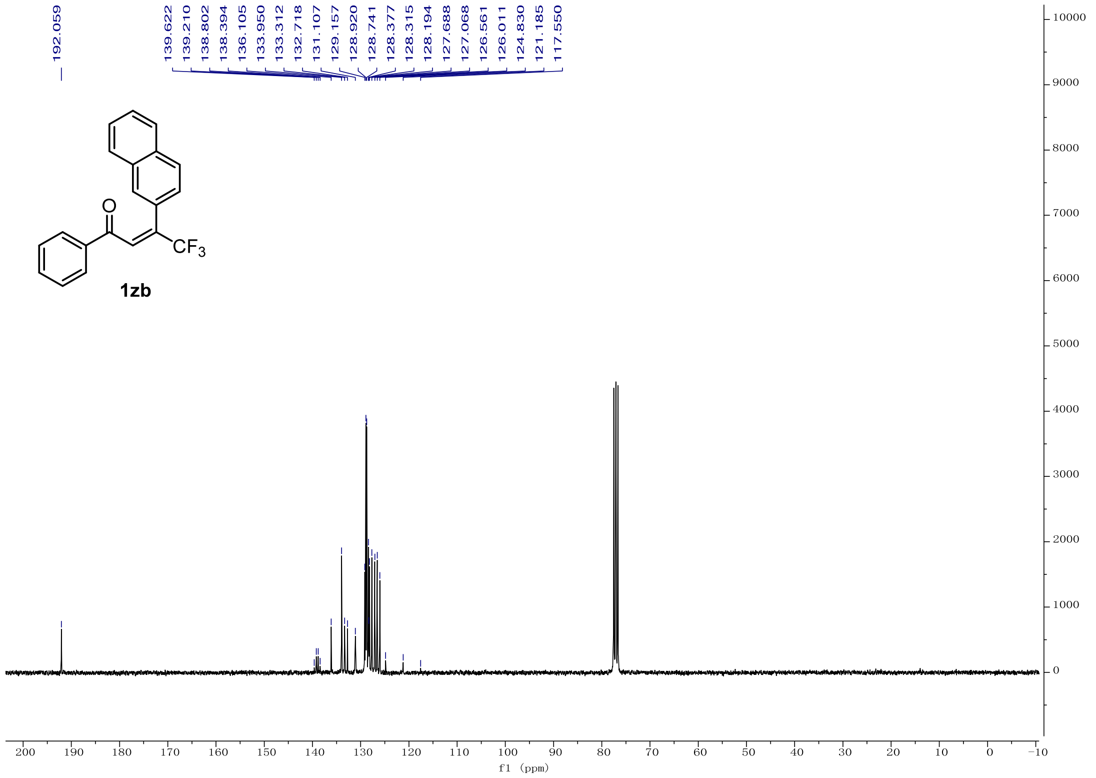

^{19}F NMR (376 MHz, $CDCl_3$) of compound **1zb**

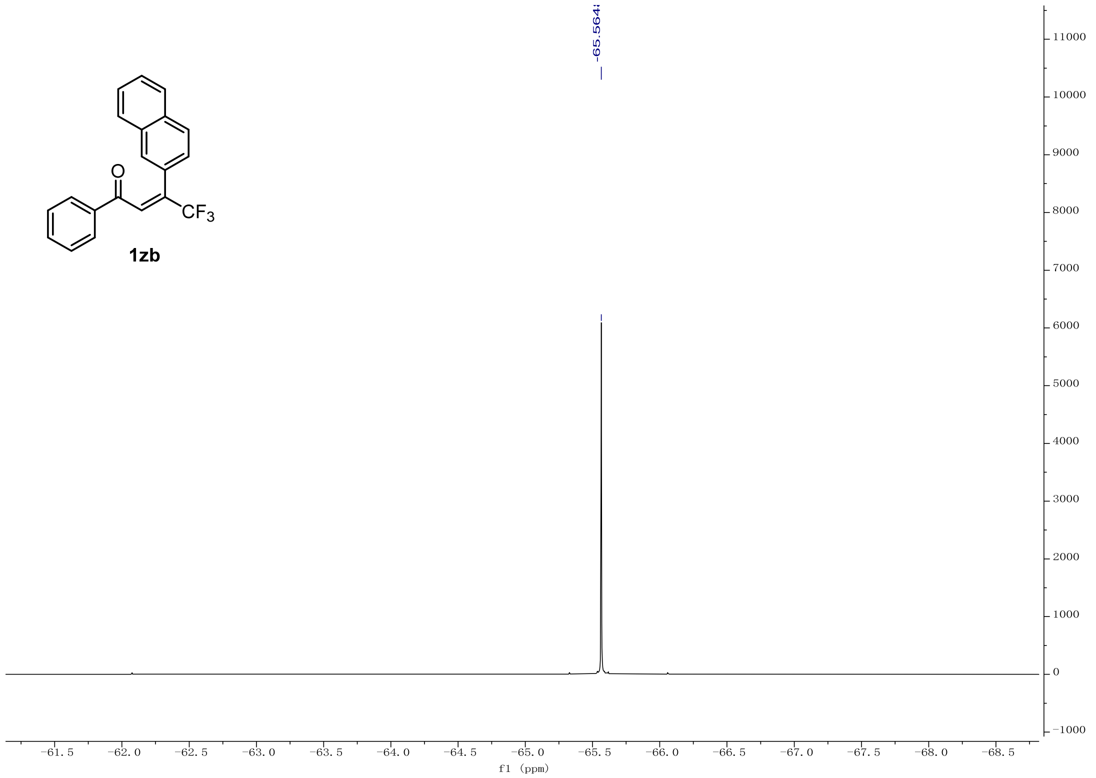

^{1}H NMR (300 MHz, $CDCl_3$) of compound **1zc**

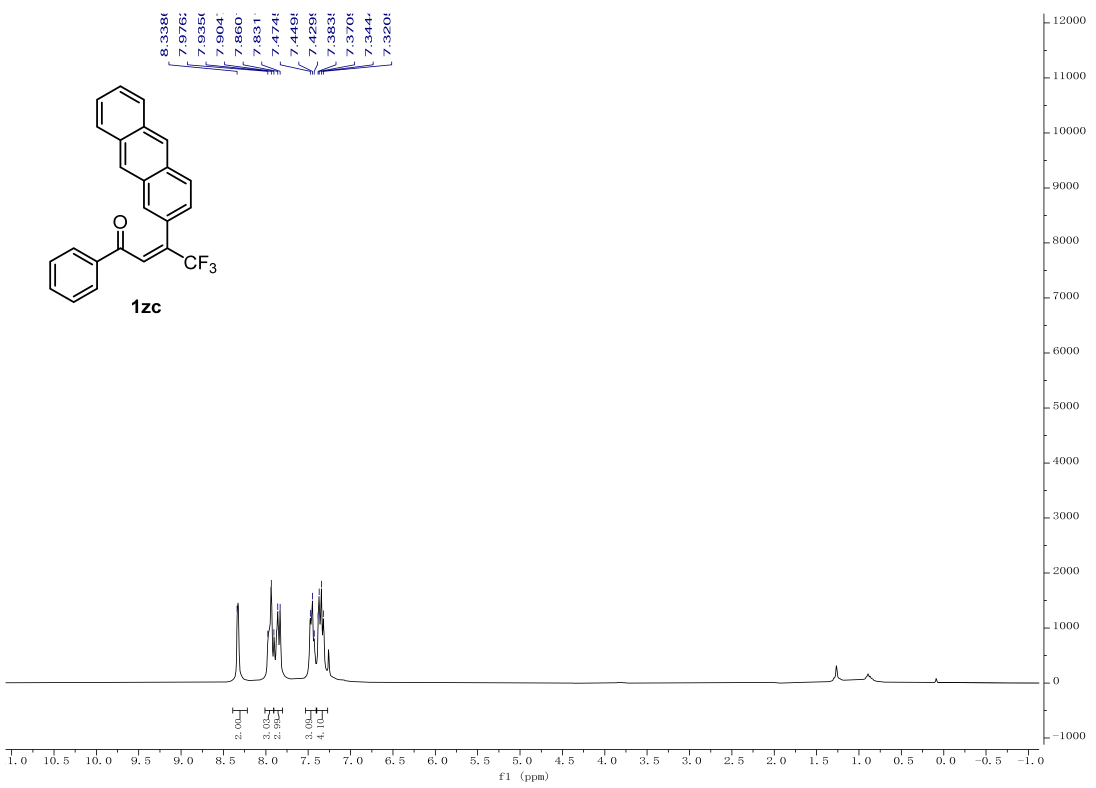

^{13}C NMR (75 MHz, $CDCl_3$) of compound **1zc**

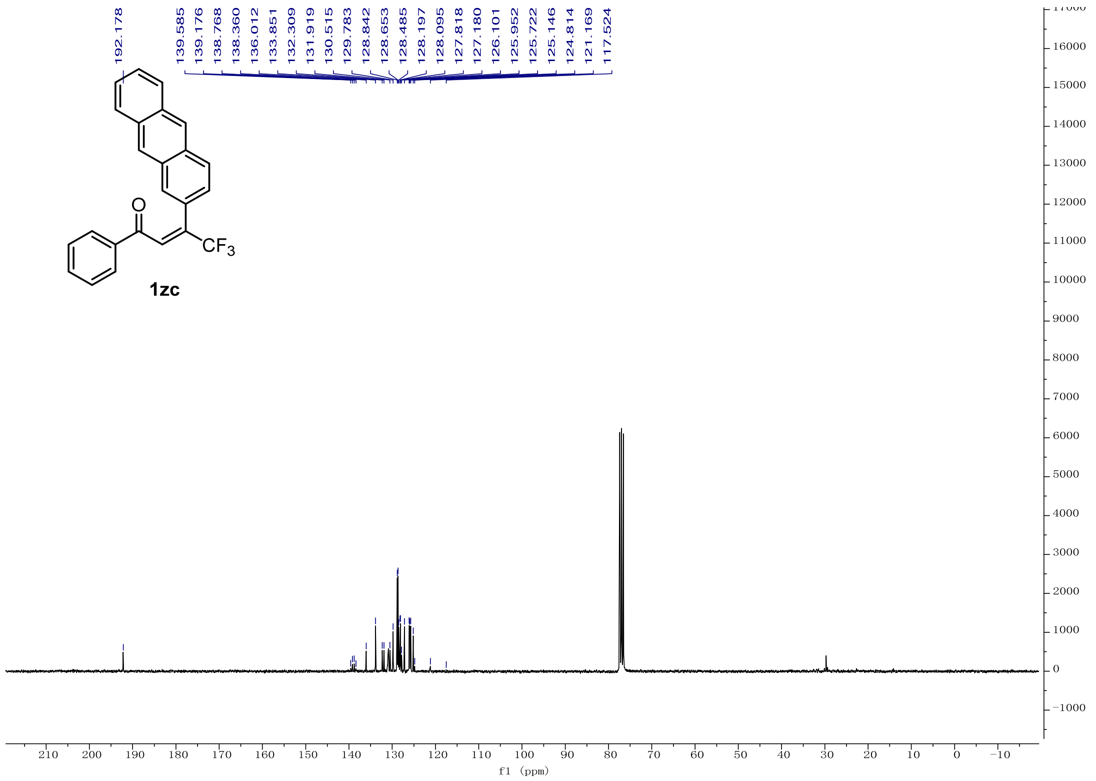

^{19}F NMR (376 MHz, $CDCl_3$) of compound **1zc**

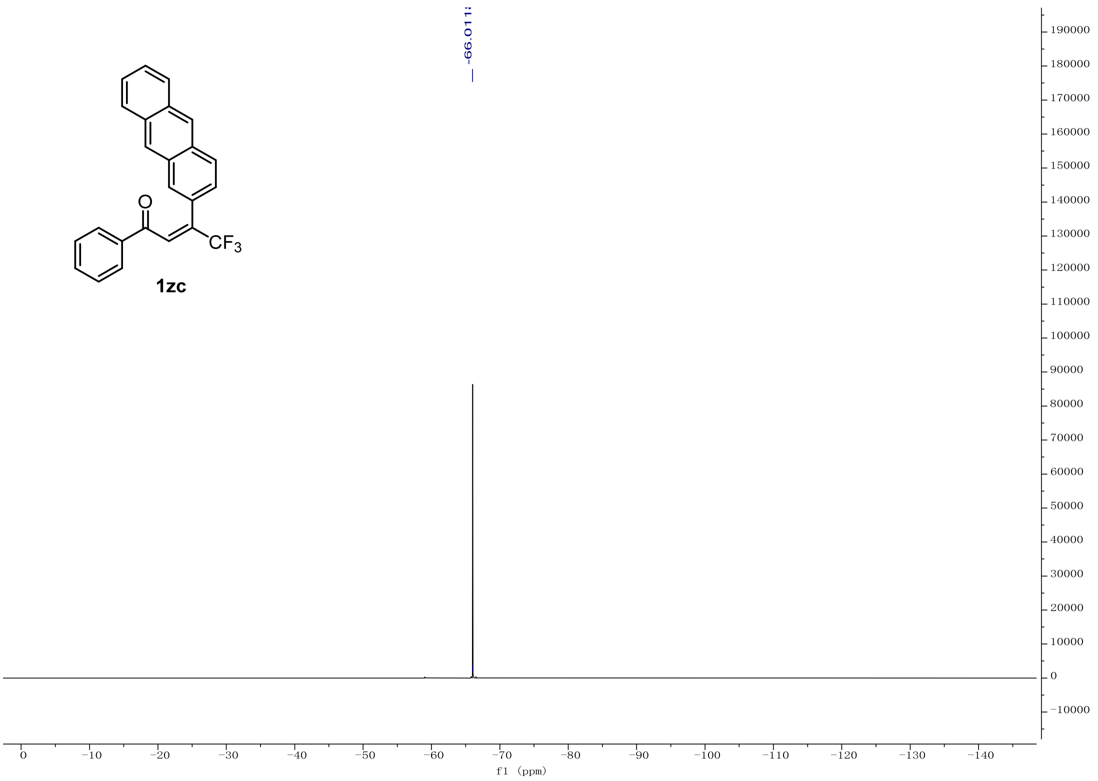

^{1}H NMR (300 MHz, $CDCl_3$) of compound **1zd**

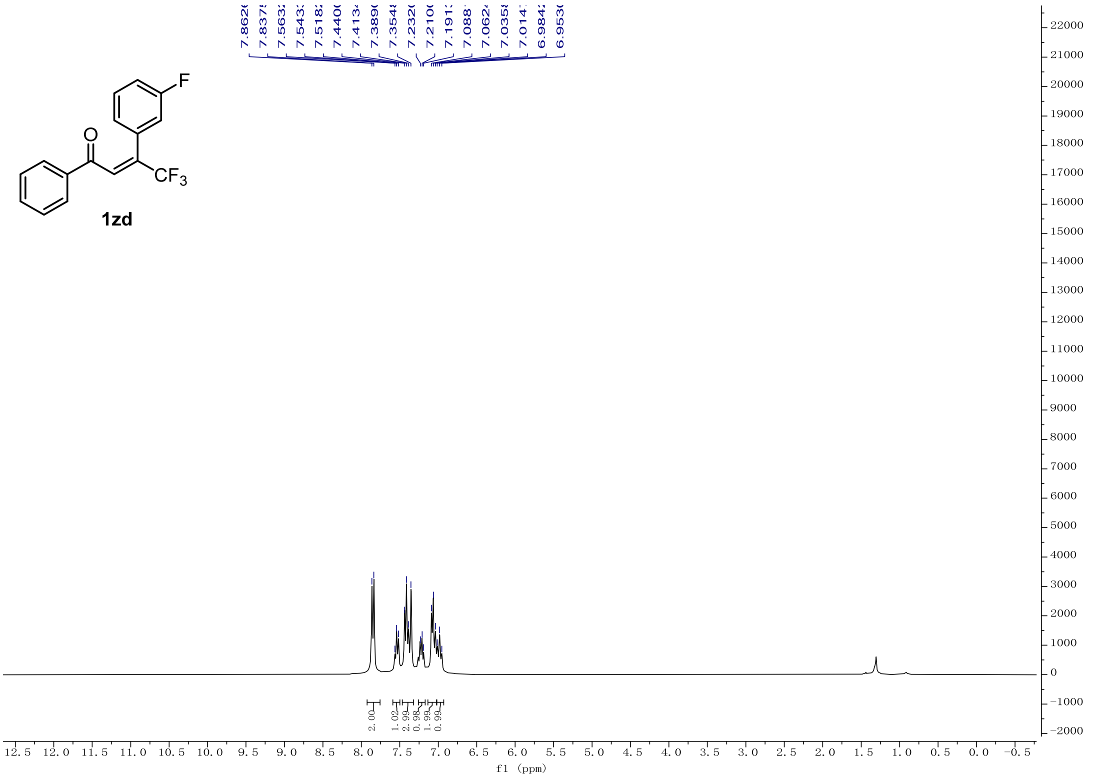

^{13}C NMR (75 MHz, $CDCl_3$) of compound **1zd**

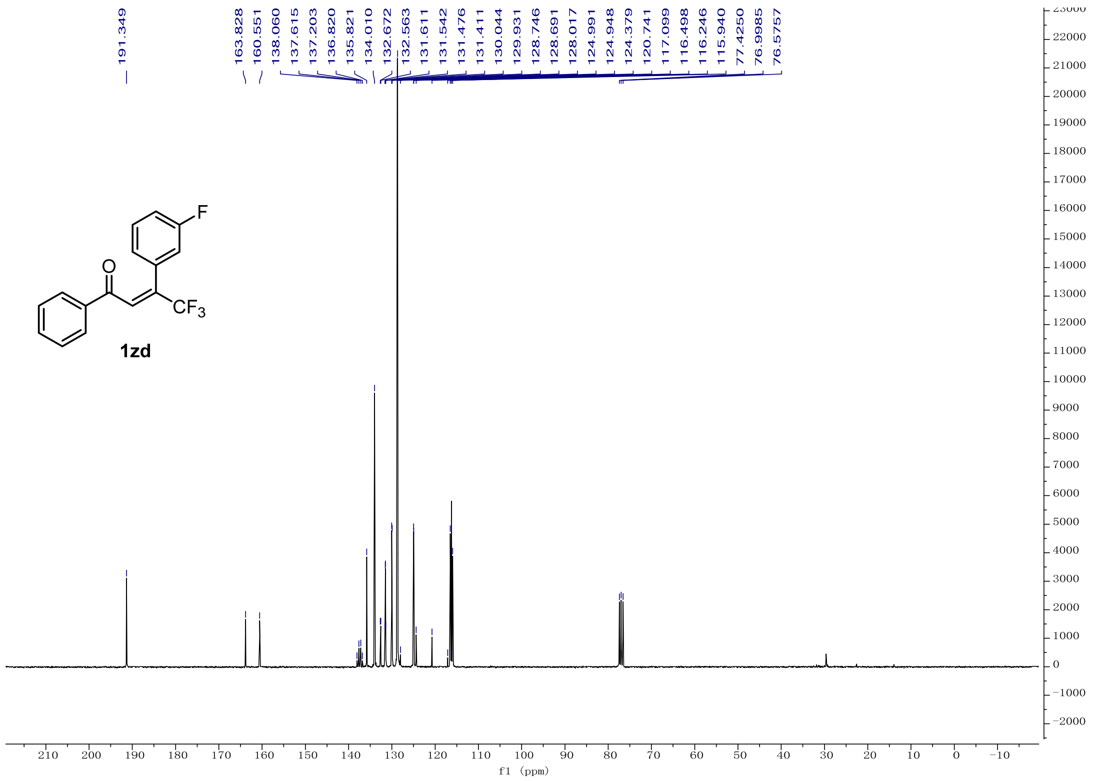

^{19}F NMR (376 MHz, $CDCl_3$) of compound **1zd**

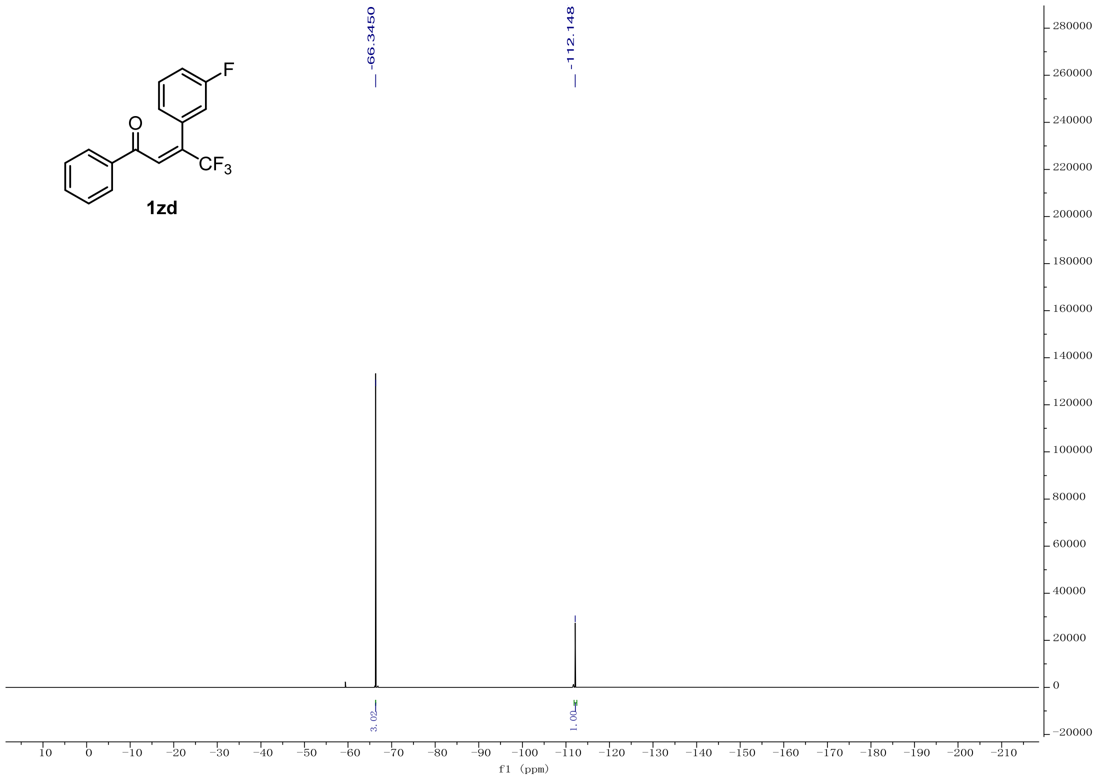

^{1}H NMR (600 MHz, $CDCl_3$) of compound **1ze**

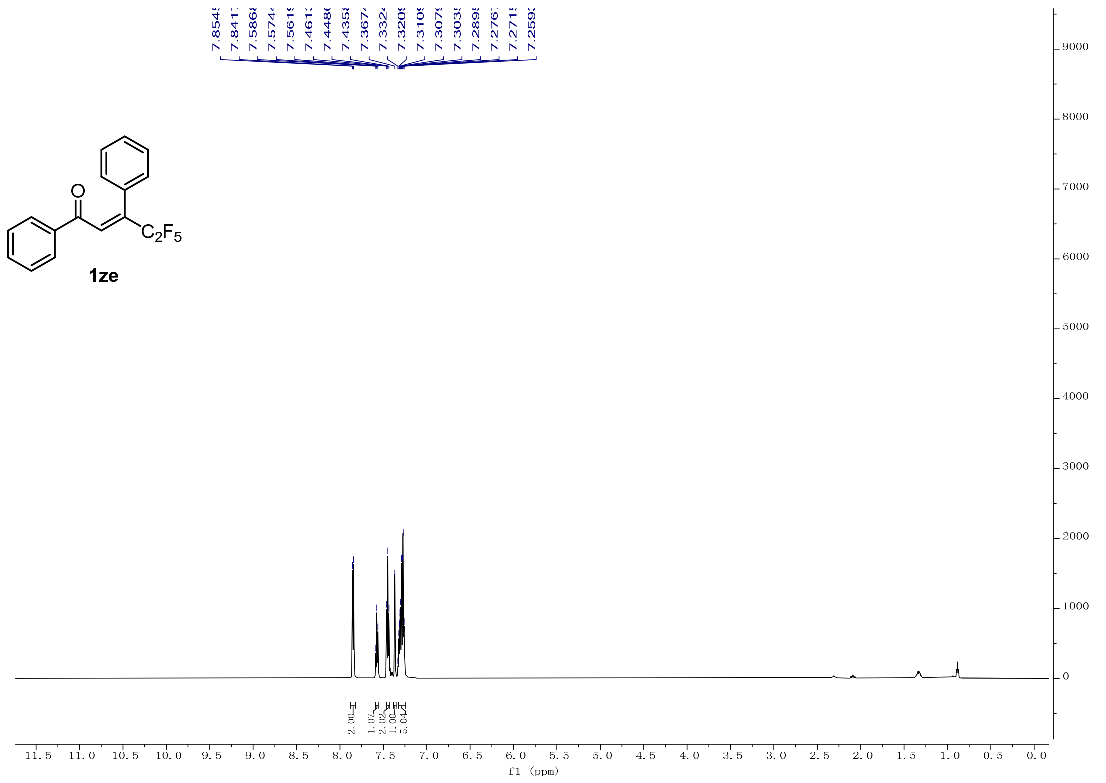

^{13}C NMR (151 MHz, $CDCl_3$) of compound **1ze**

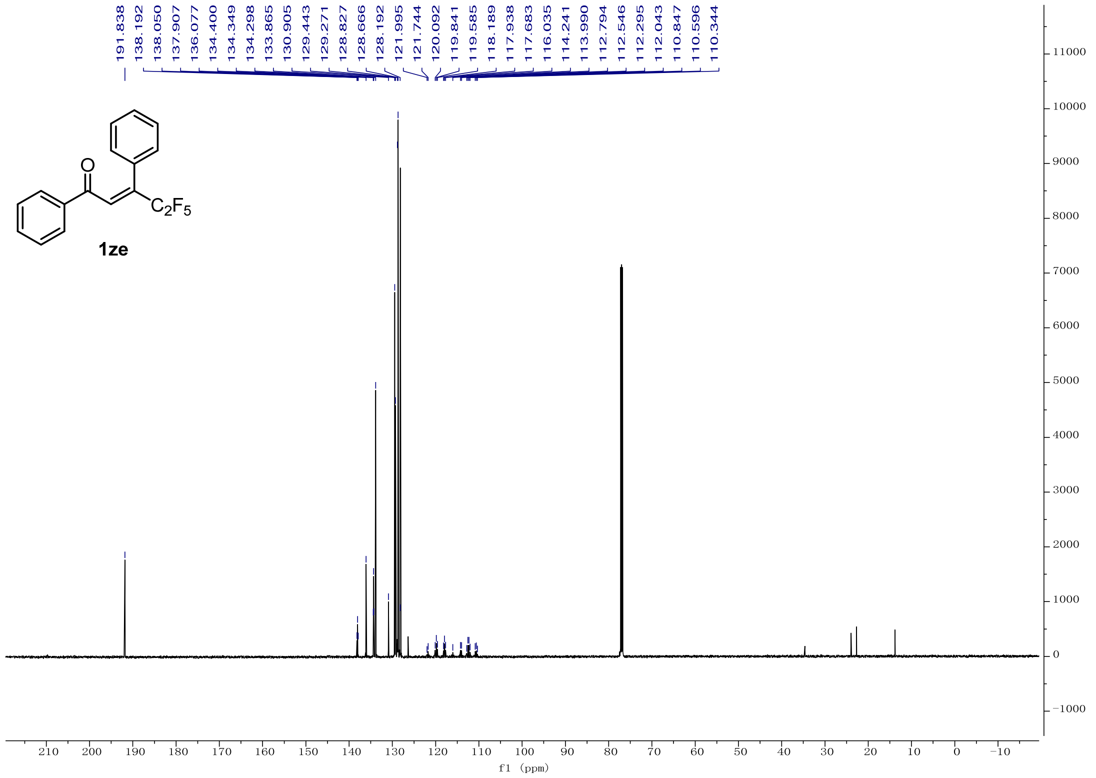

^{19}F NMR (565 MHz, $CDCl_3$) of compound **1ze**

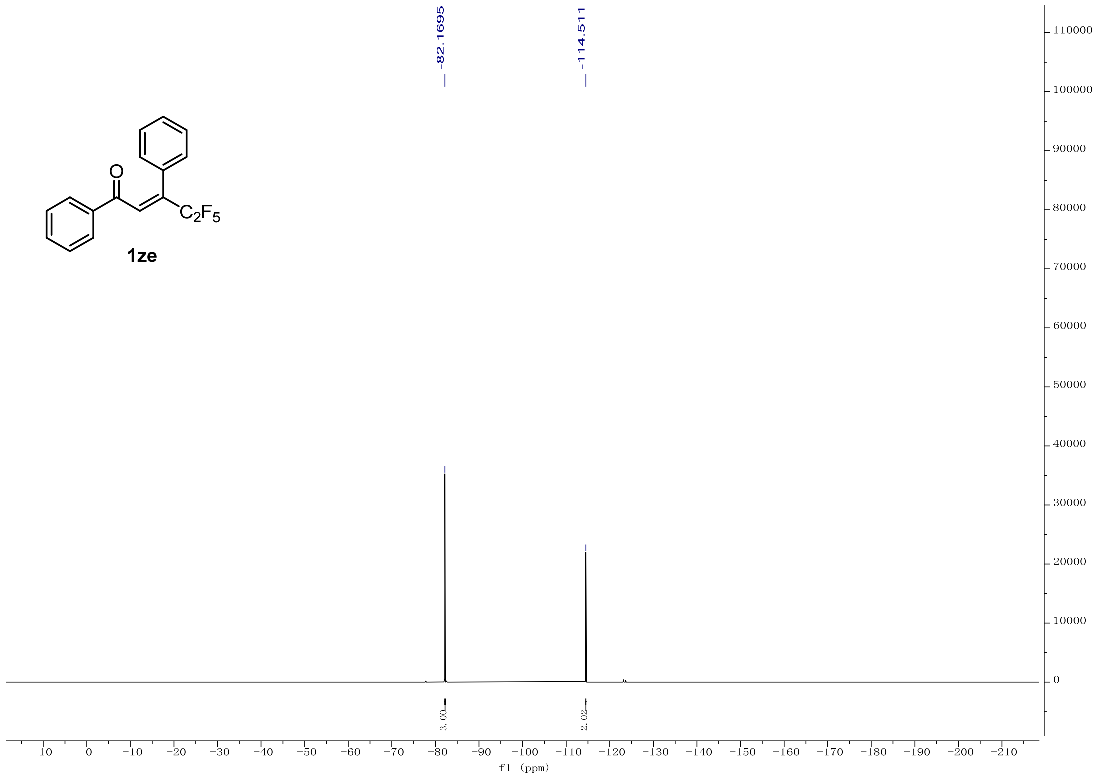

^{1}H NMR (300 MHz, $CDCl_3$) of compound **1zf**

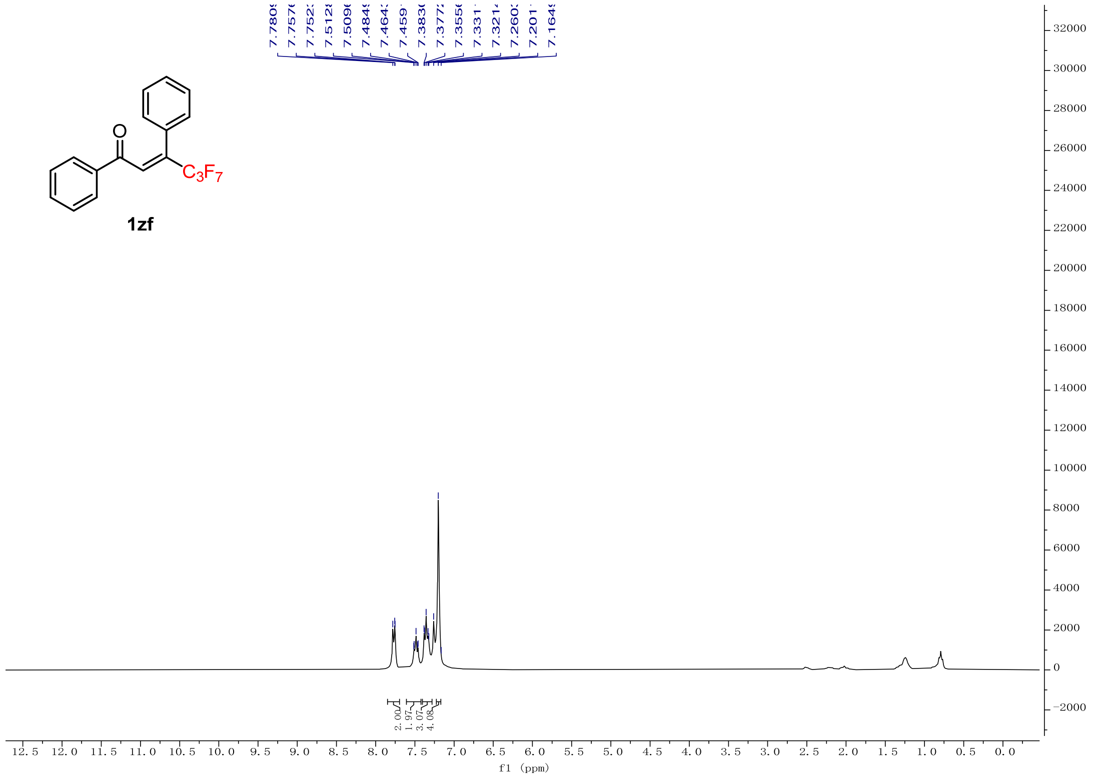

^{13}C NMR (75 MHz, $CDCl_3$) of compound **1zf**

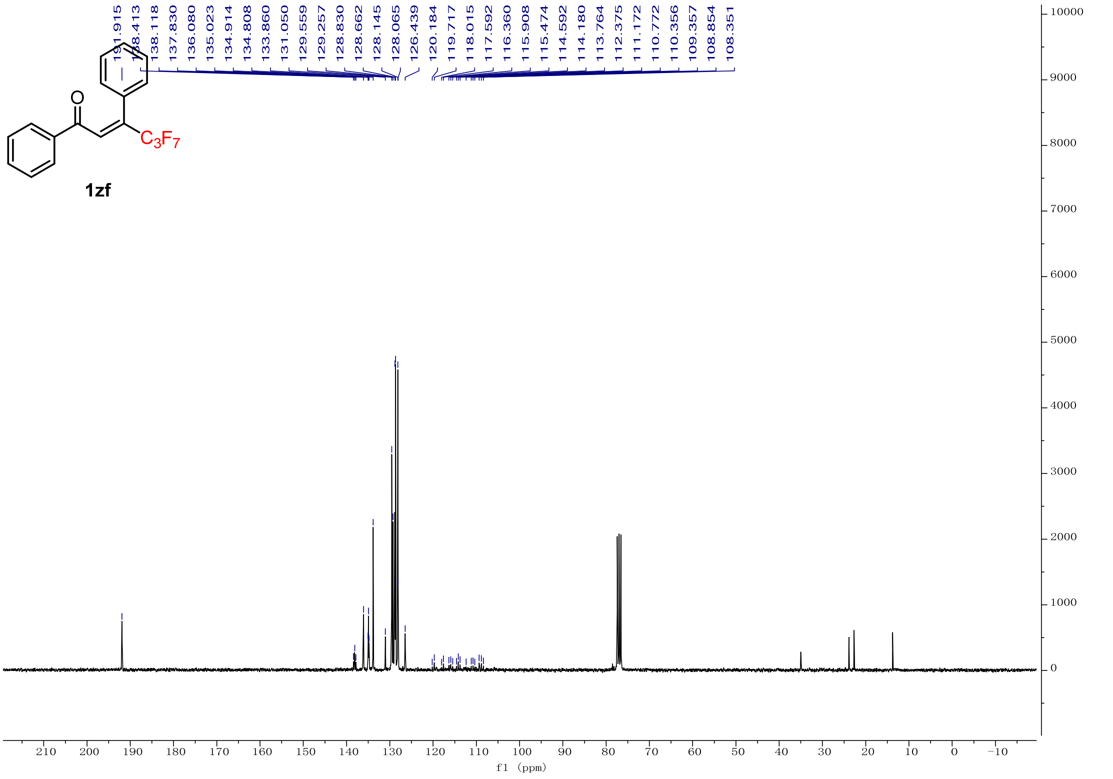

^{19}F NMR (376 MHz, $CDCl_3$) of compound **1zf**

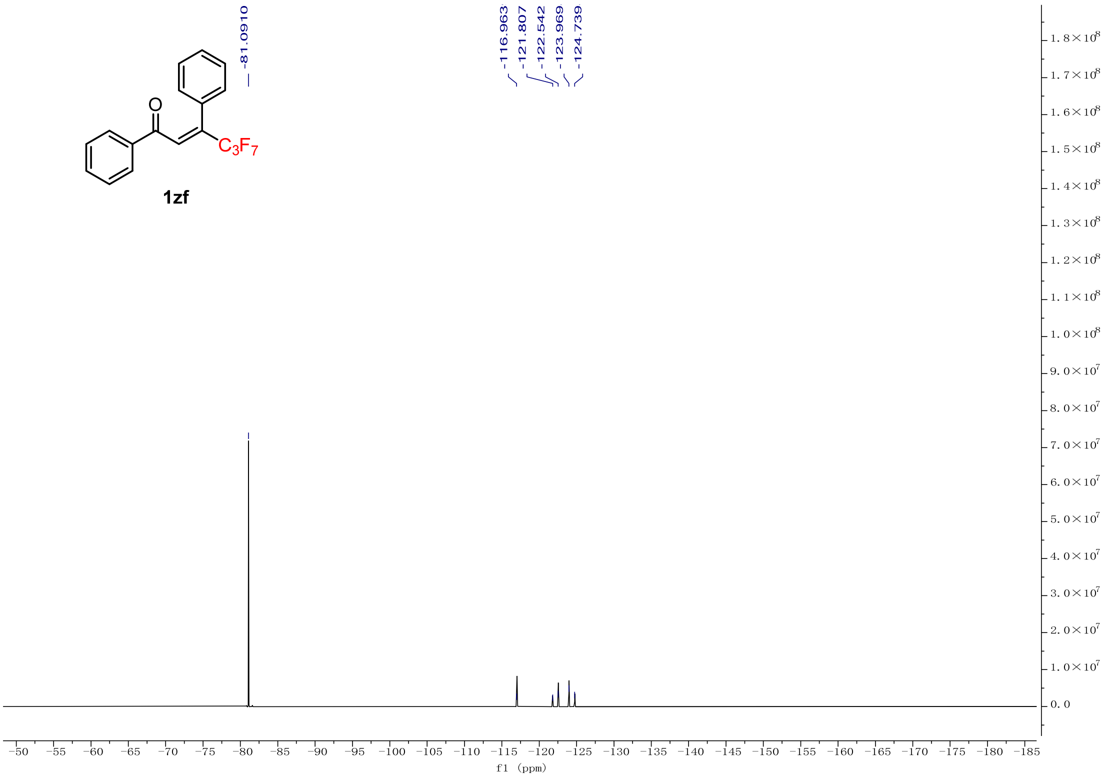

^{1}H NMR (300 MHz, $CDCl_3$) of compound **1zg**

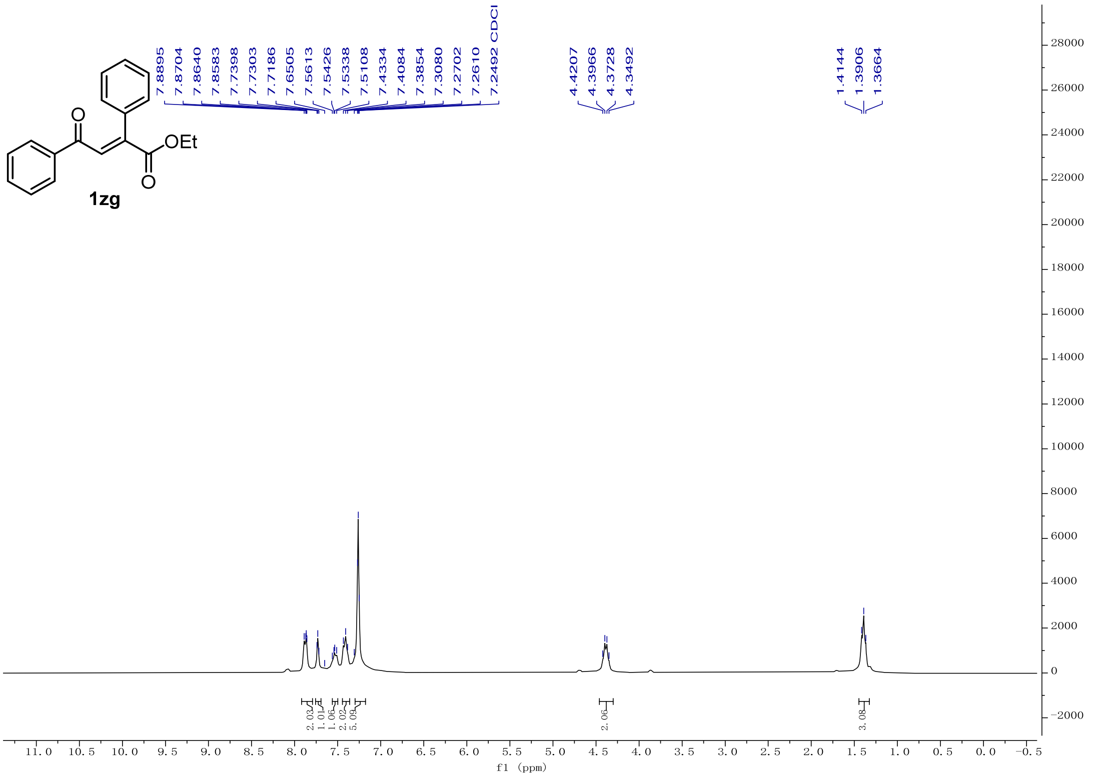

^{13}C NMR (75 MHz, $CDCl_3$) of compound **1zg**

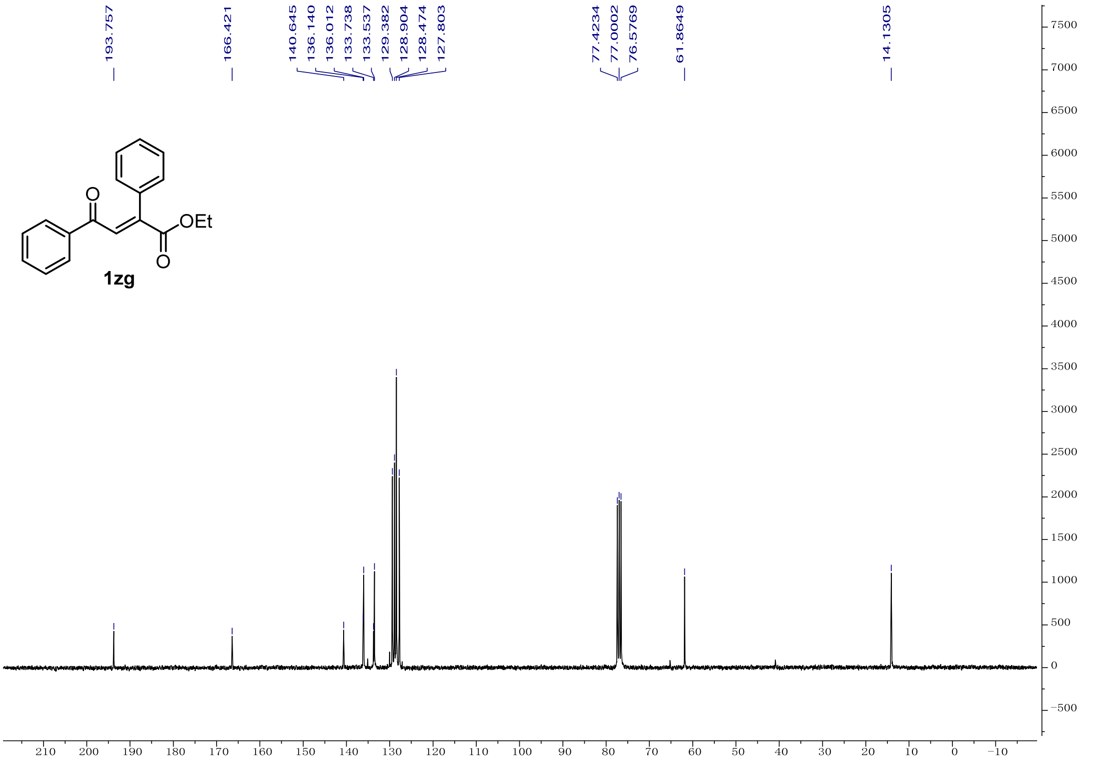

^{1}H NMR (300 MHz, $CDCl_3$) of compound **1zh**

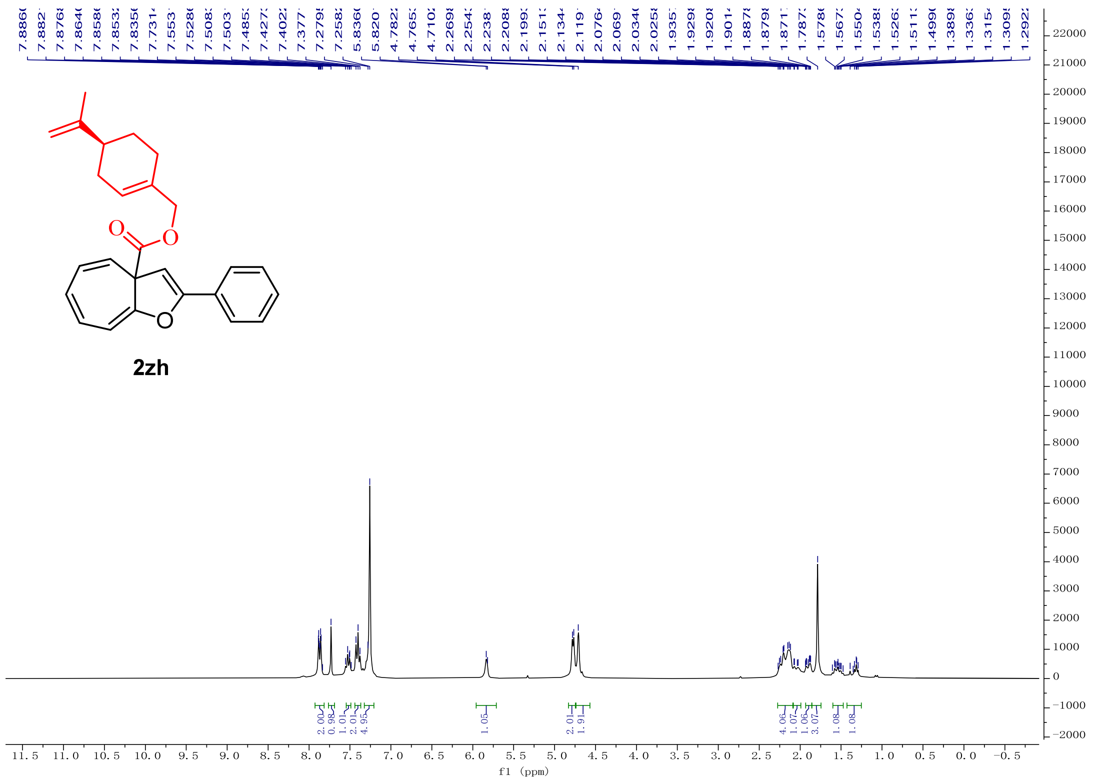

^{13}C NMR (75 MHz, $CDCl_3$) of compound **1zh**

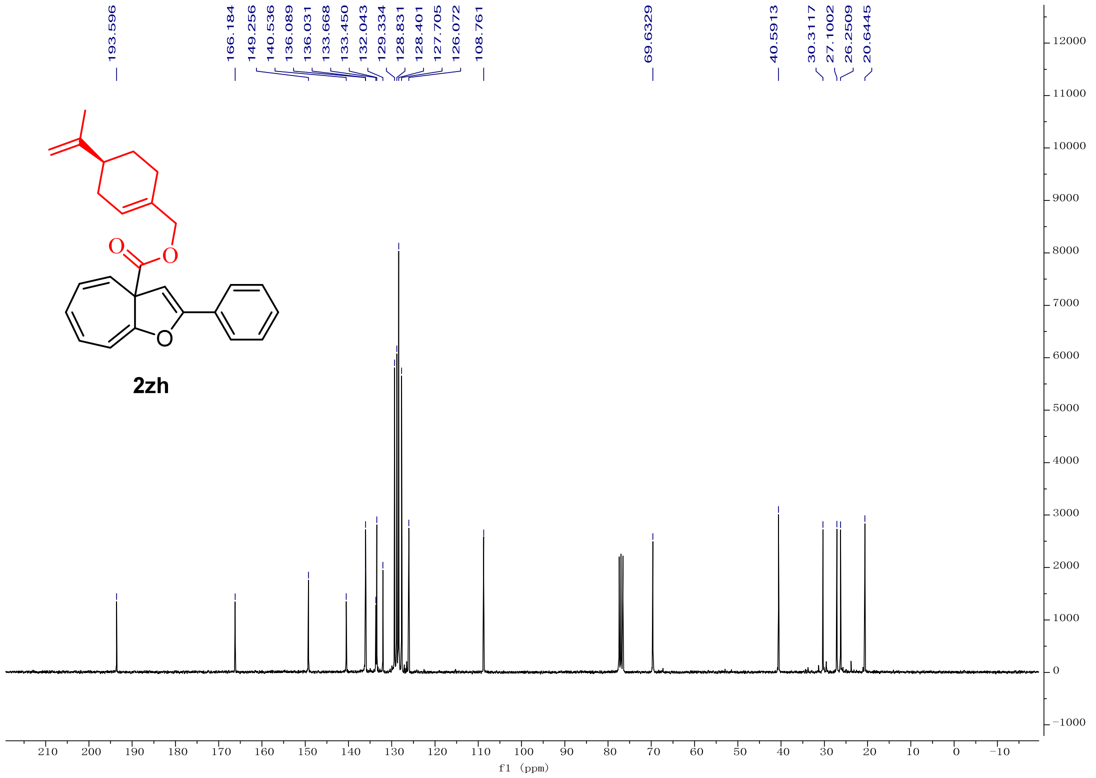

^{1}H NMR (300 MHz, $CDCl_3$) of compound **2zh**

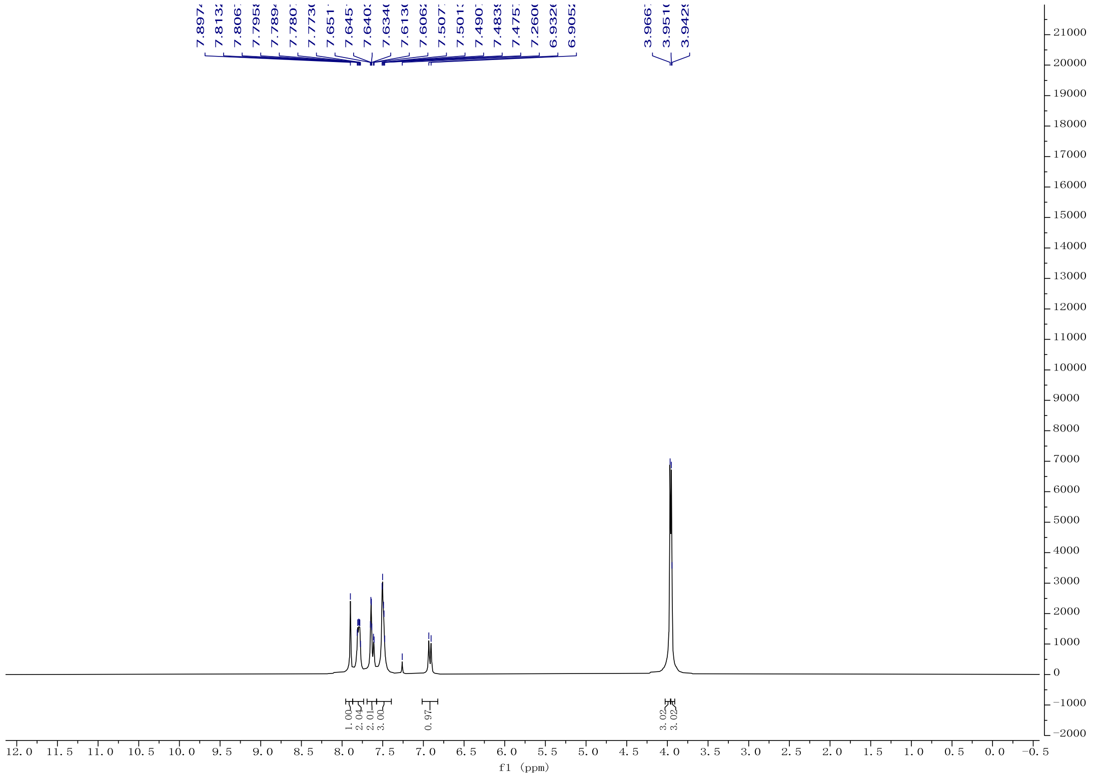

^{1}H NMR (300 MHz, $CDCl_3$) of compound **2zi**

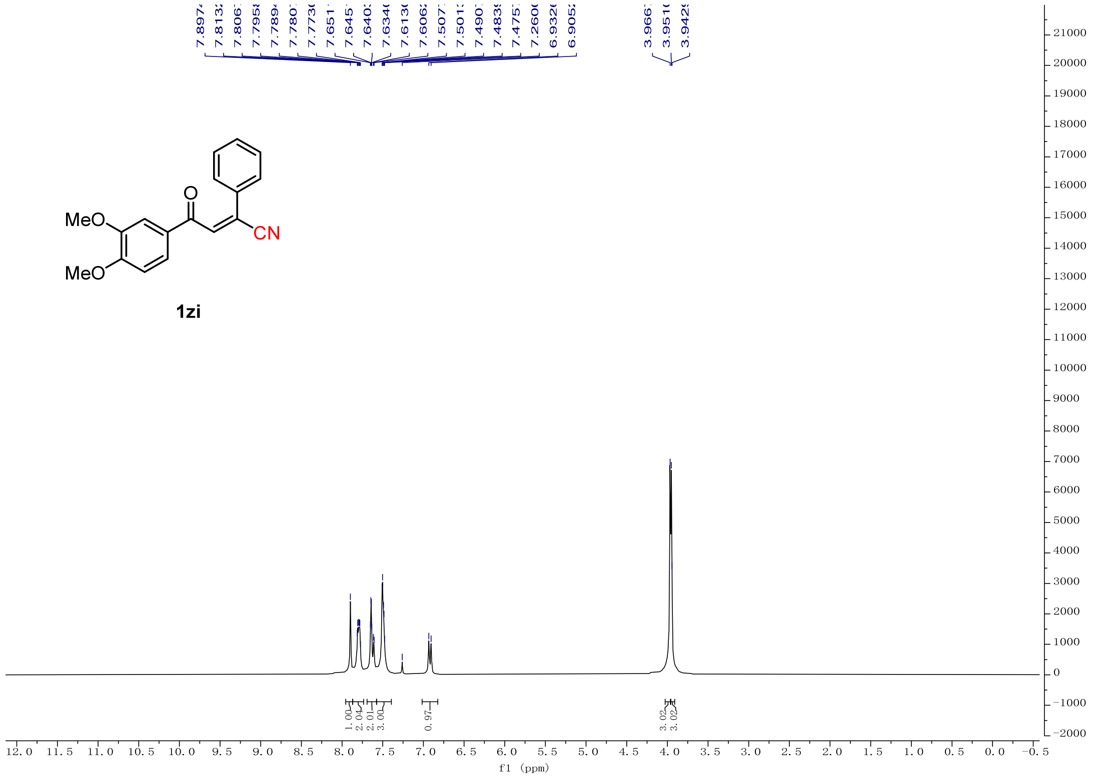

^{13}C NMR (75 MHz, $CDCl_3$) of compound **2zi**

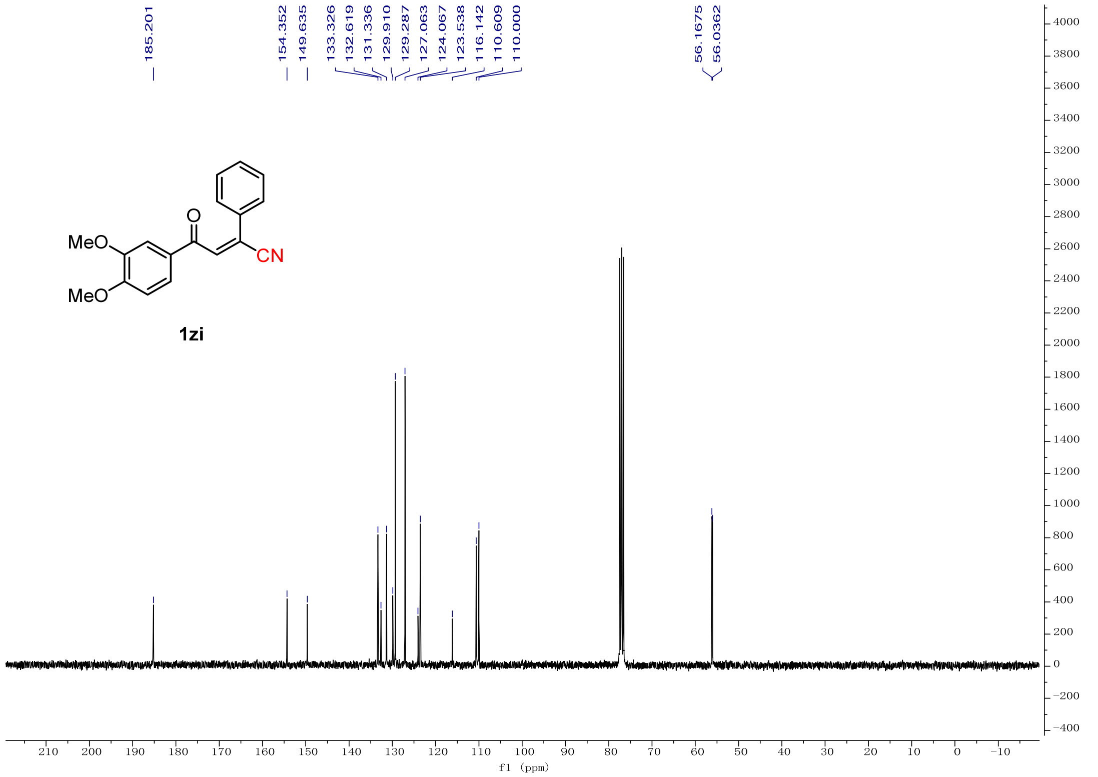

^{1}H NMR (300 MHz, $CDCl_3$) of compound **1zj**

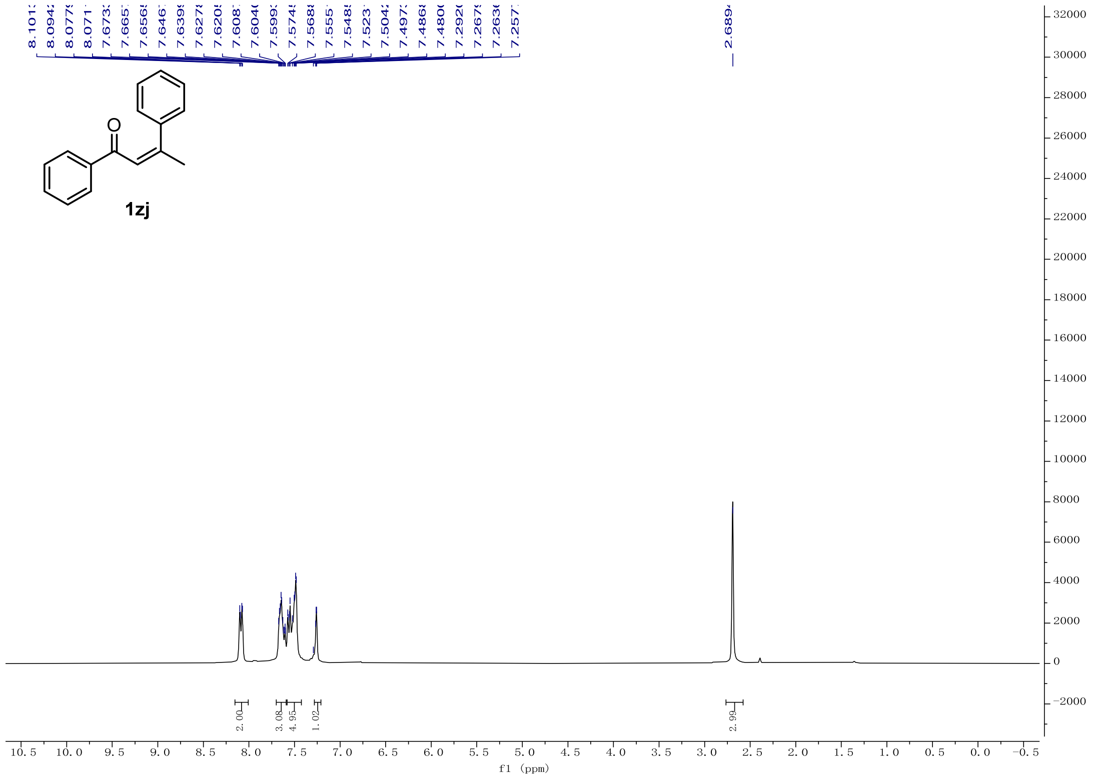

^{13}C NMR (75 MHz, $CDCl_3$) of compound **1zj**

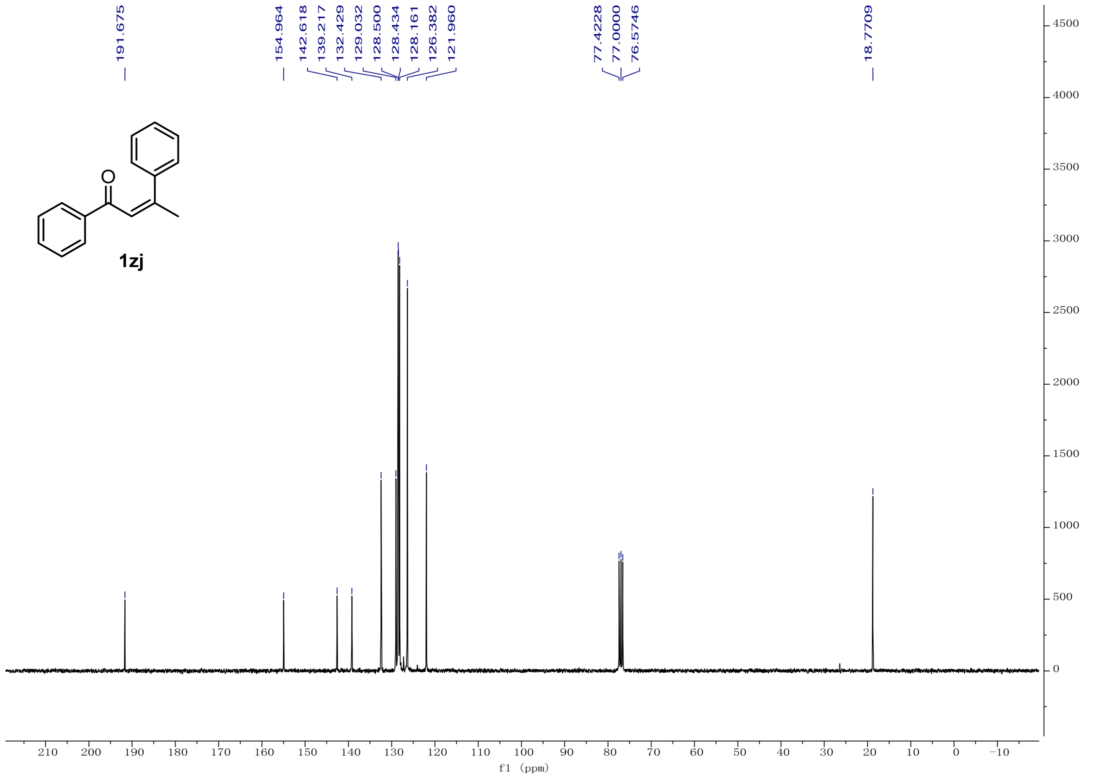

^{1}H NMR (300 MHz, $CDCl_3$) of compound **1zk**

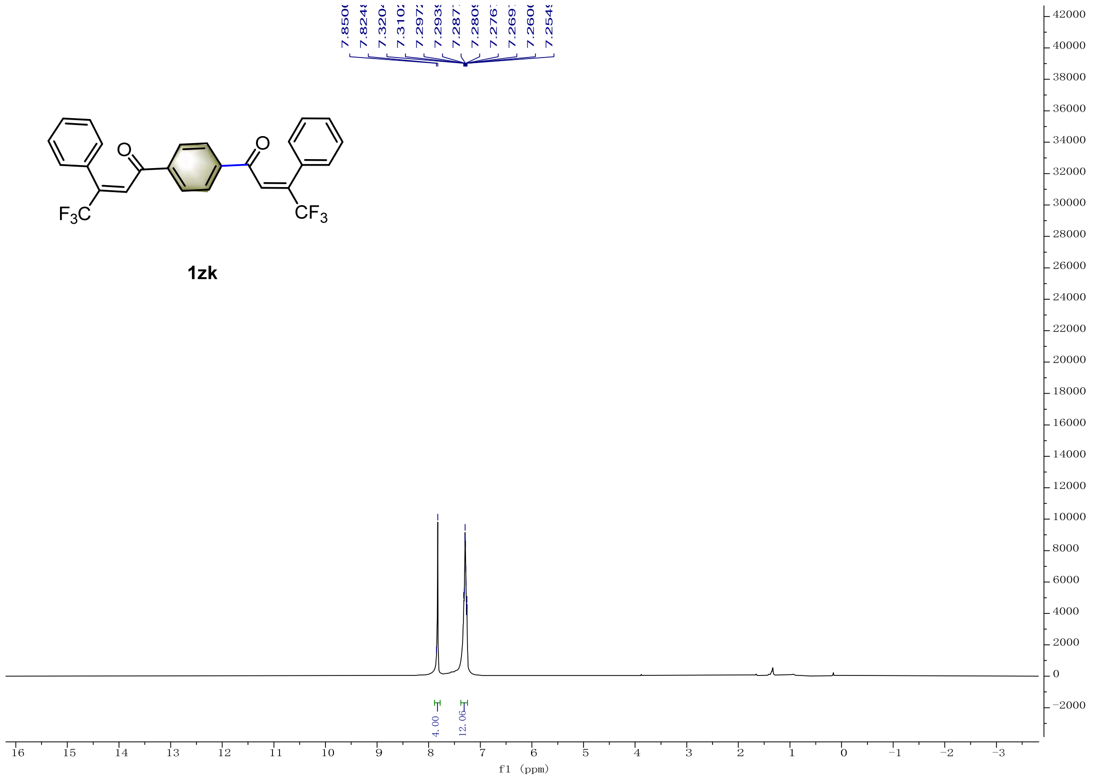

^{13}C NMR (75 MHz, $CDCl_3$) of compound **1zk**

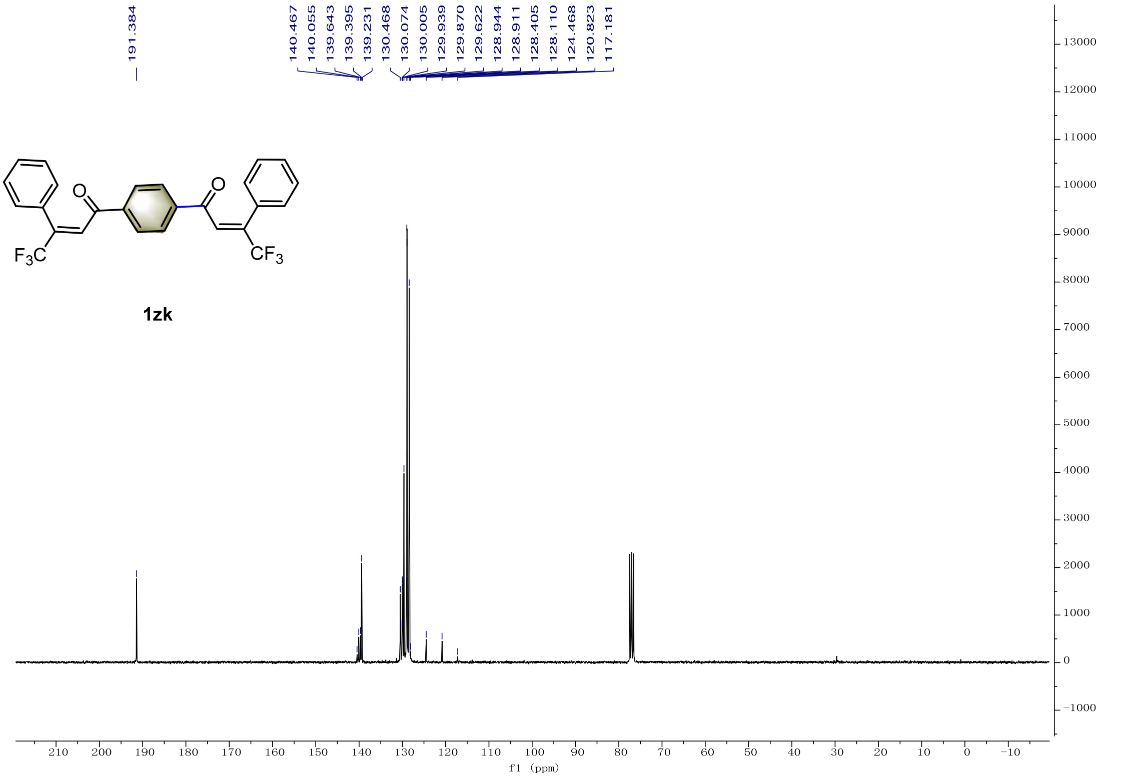

^{19}F NMR (376 MHz, $CDCl_3$) of compound **1zk**

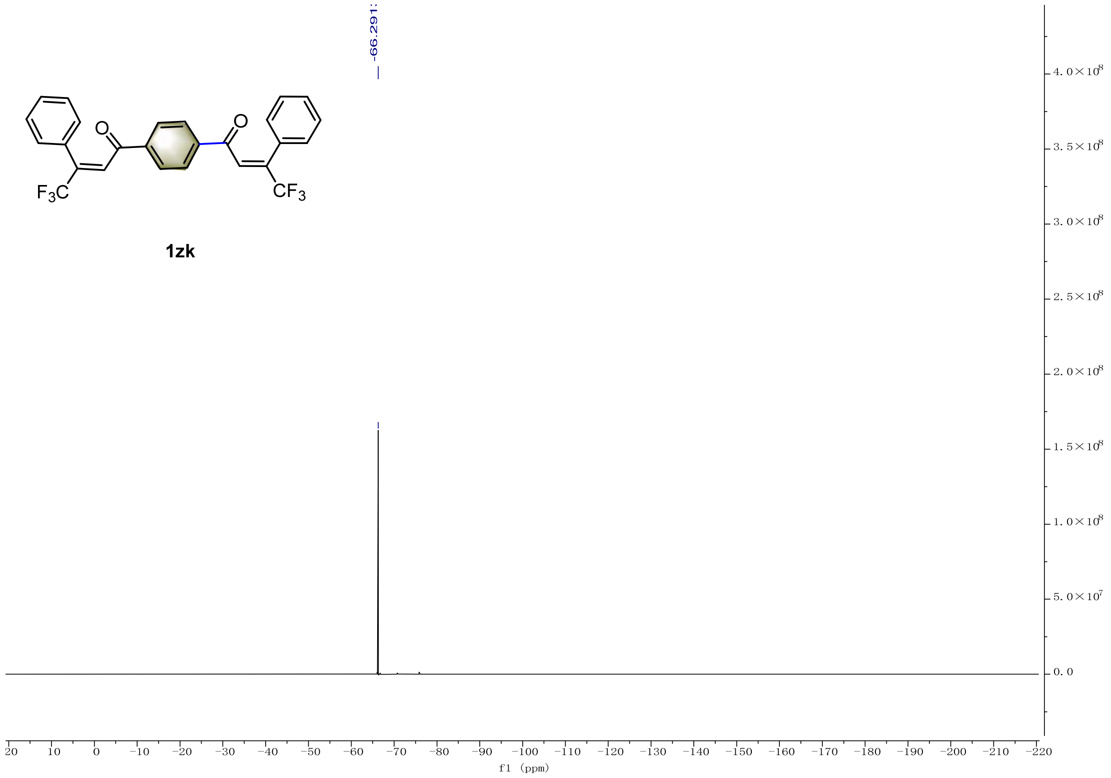

^{1}H NMR (300 MHz, $CDCl_3$) of compound **1zl**

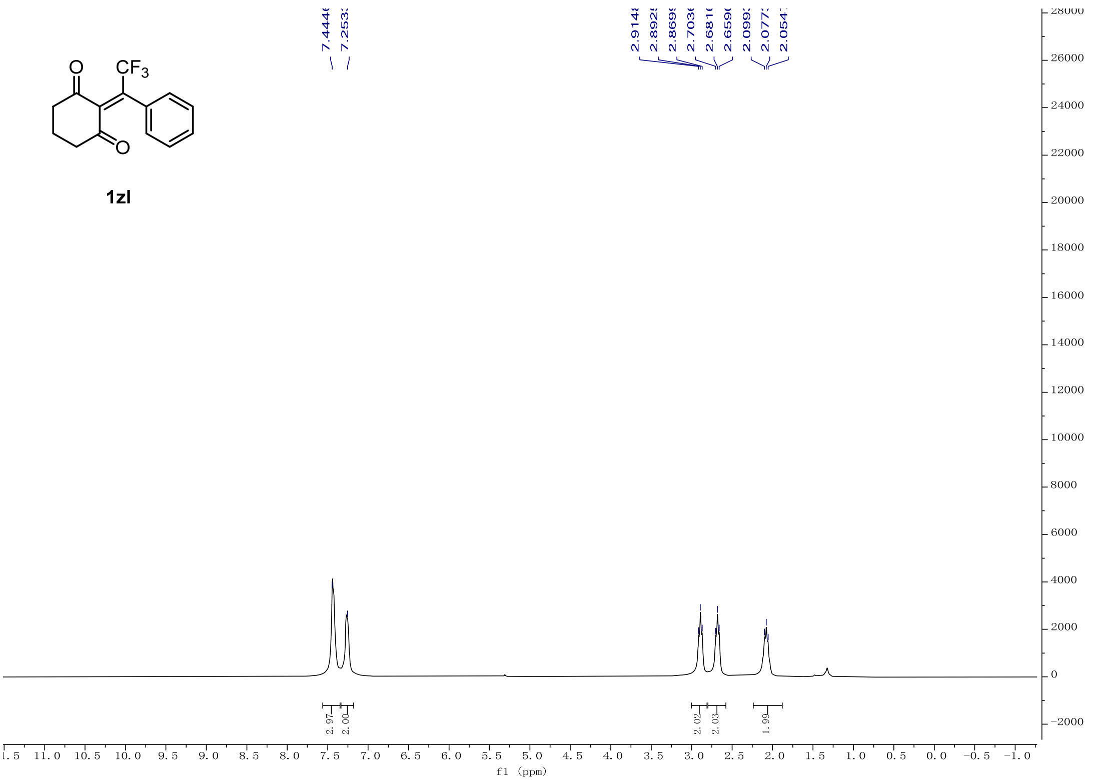

^{13}C NMR (75 MHz, $CDCl_3$) of compound **1zl**

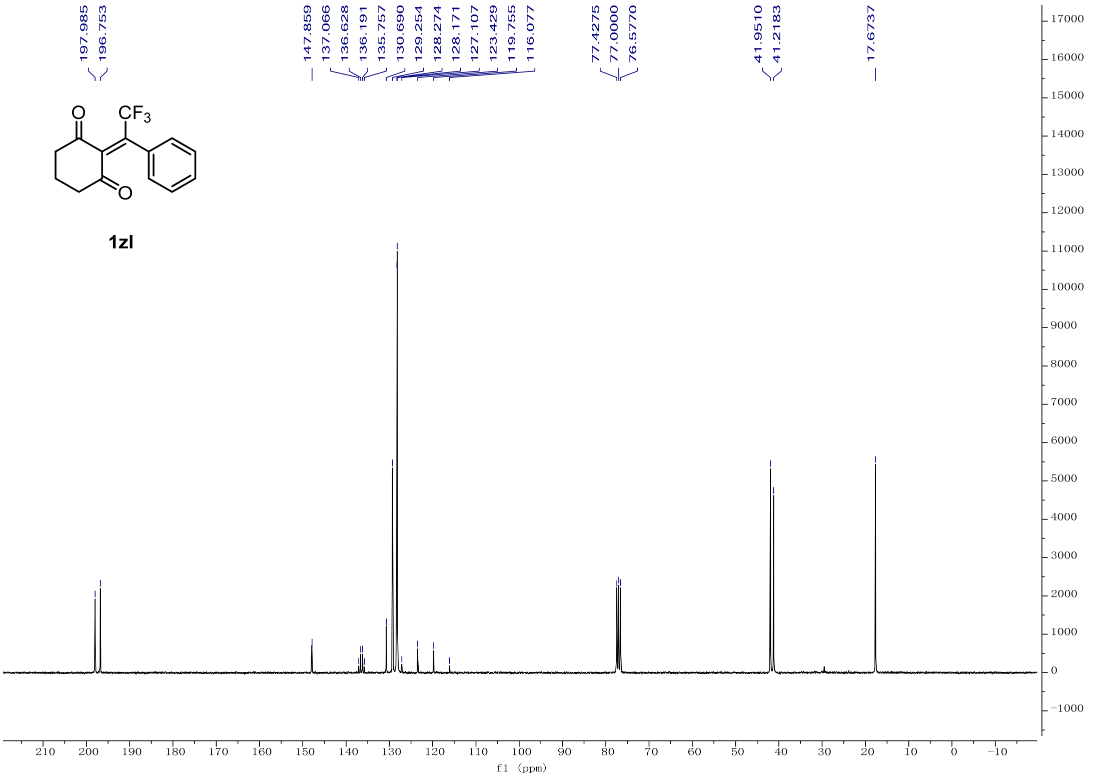

^{19}F NMR (376 MHz, $CDCl_3$) of compound **1zl**

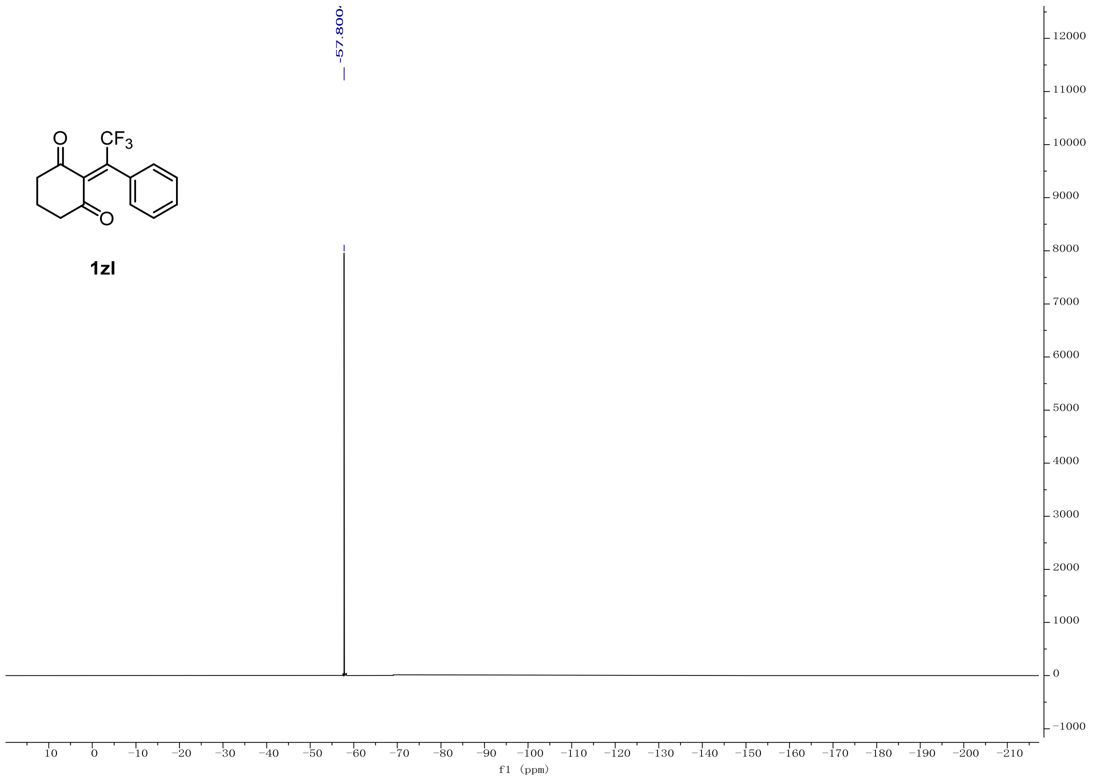

^{1}H NMR (300 MHz, $CDCl_3$) of compound **1zm**

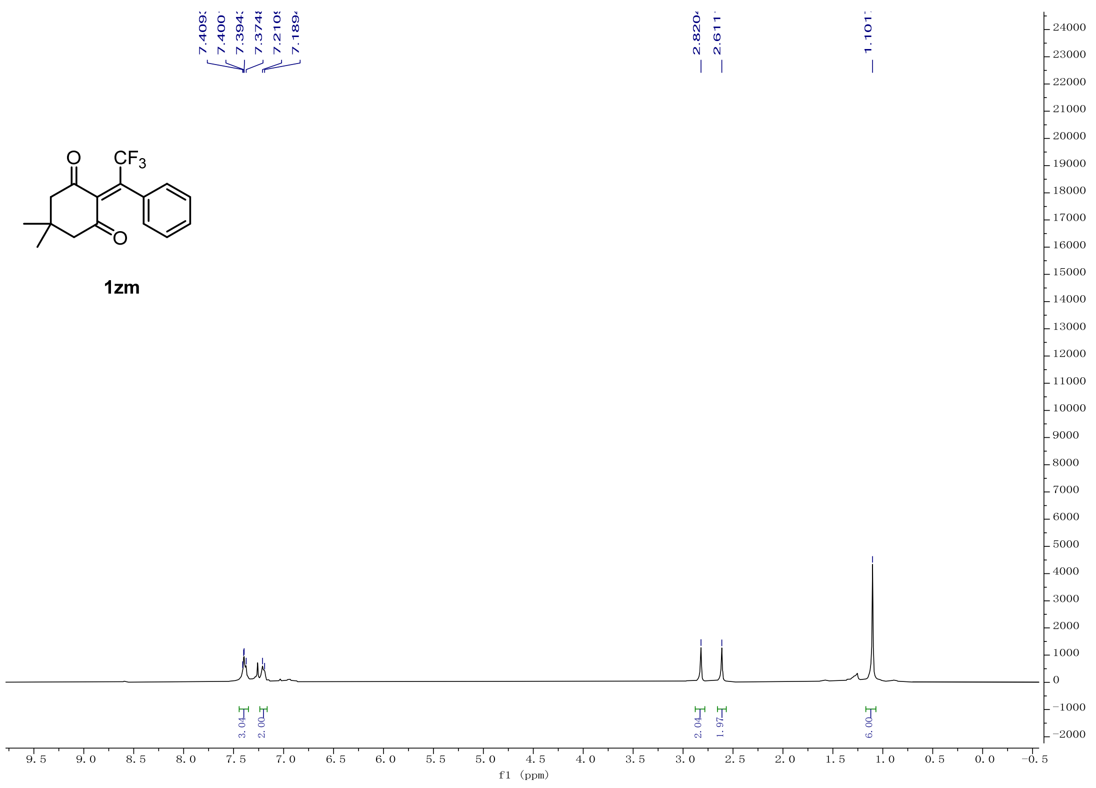

^{13}C NMR (101 MHz, $CDCl_3$) of compound **1zm**

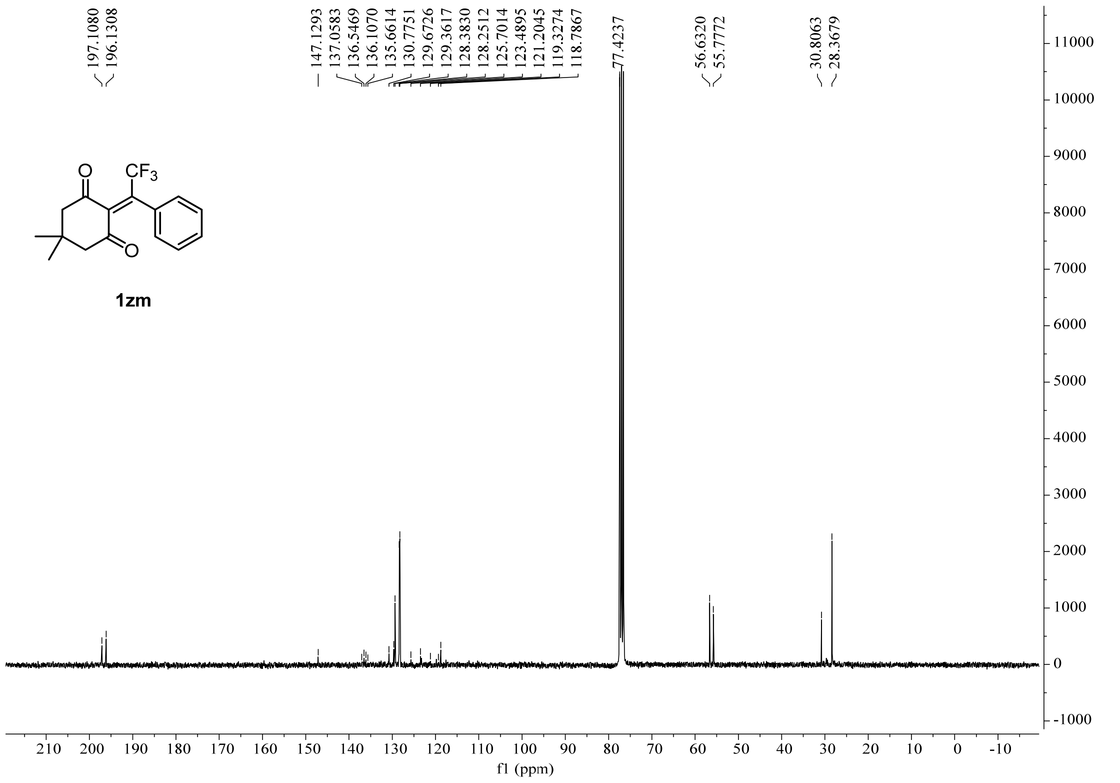

^{19}F NMR (376 MHz, $CDCl_3$) of compound **1zm**

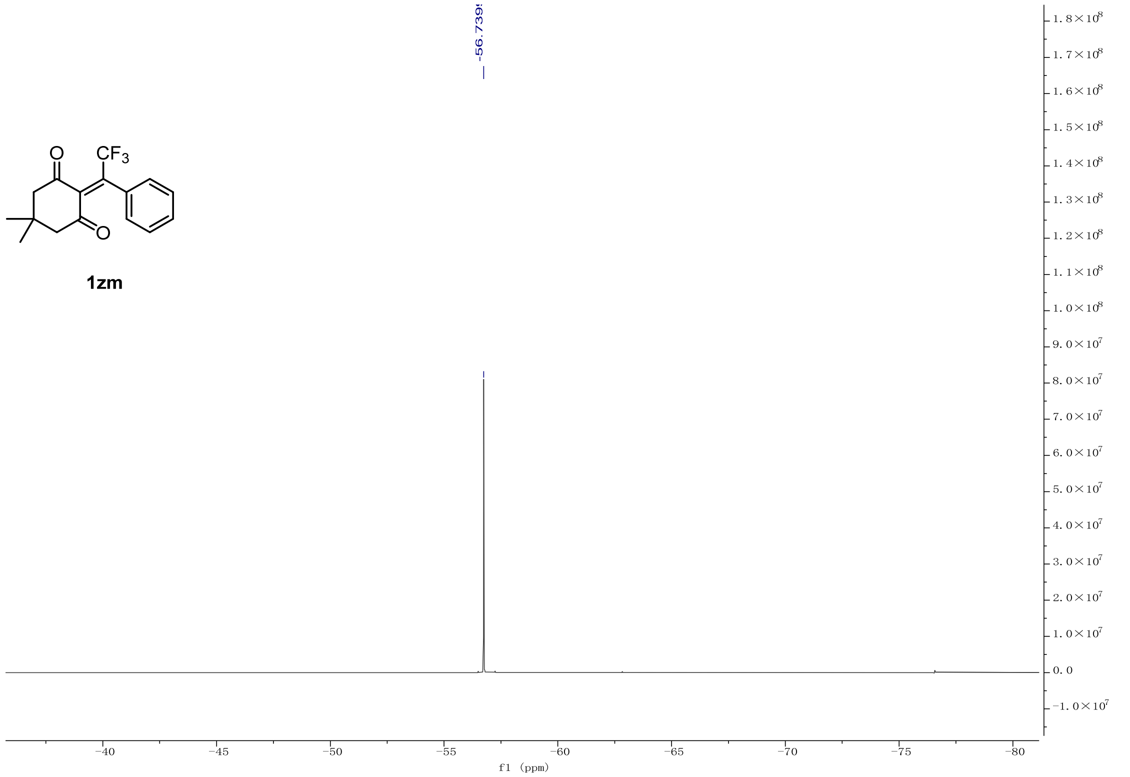

^{1}H NMR (400 MHz, $CDCl_3$) of compound **1zn**

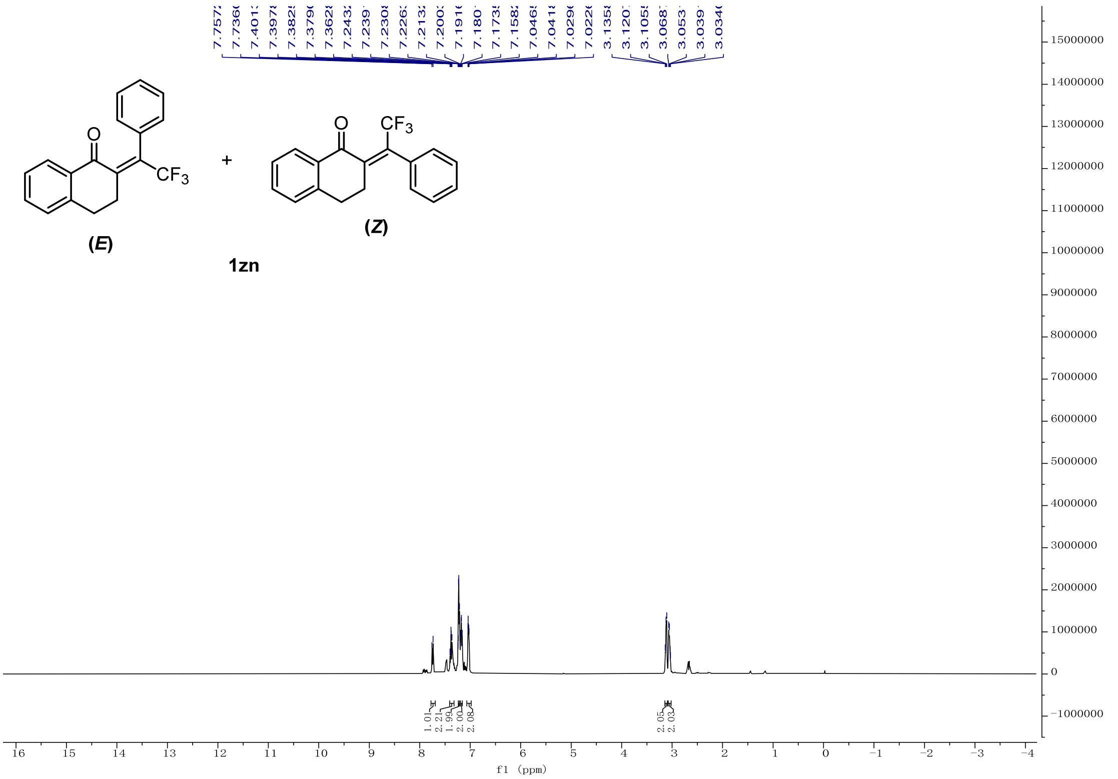

^{13}C NMR (101 MHz, $CDCl_3$) of compound **1zn**

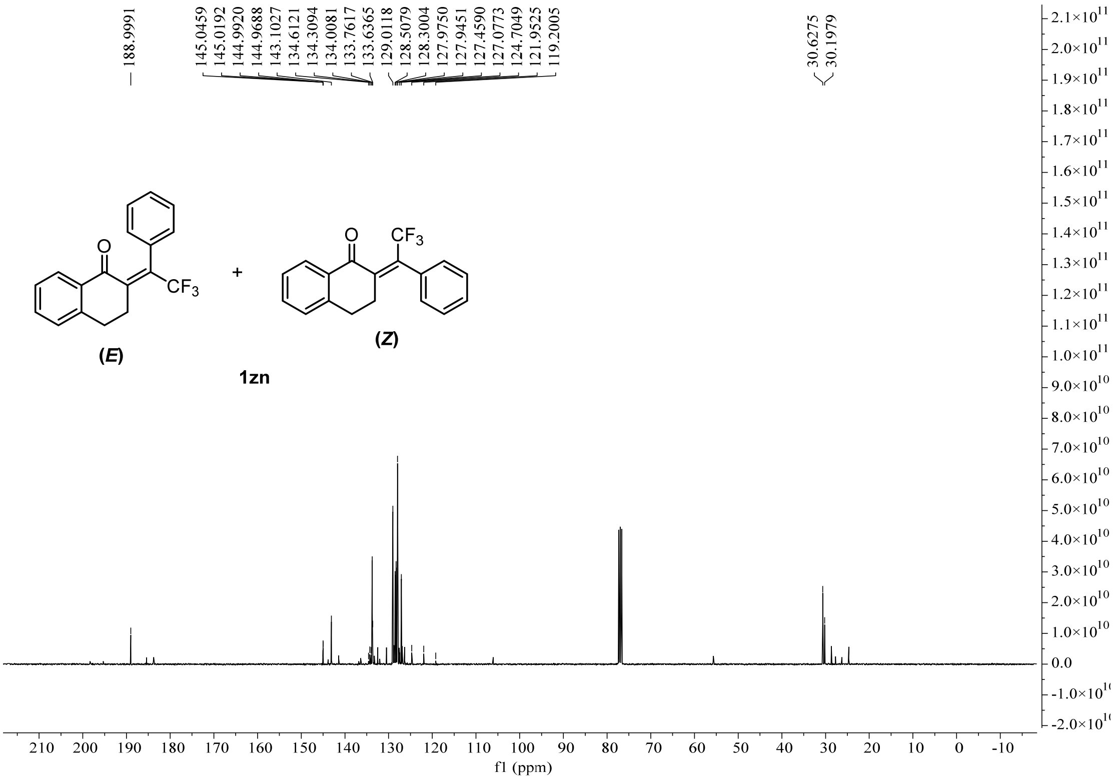

^{19}F NMR (376 MHz, $CDCl_3$) of compound **1zn**

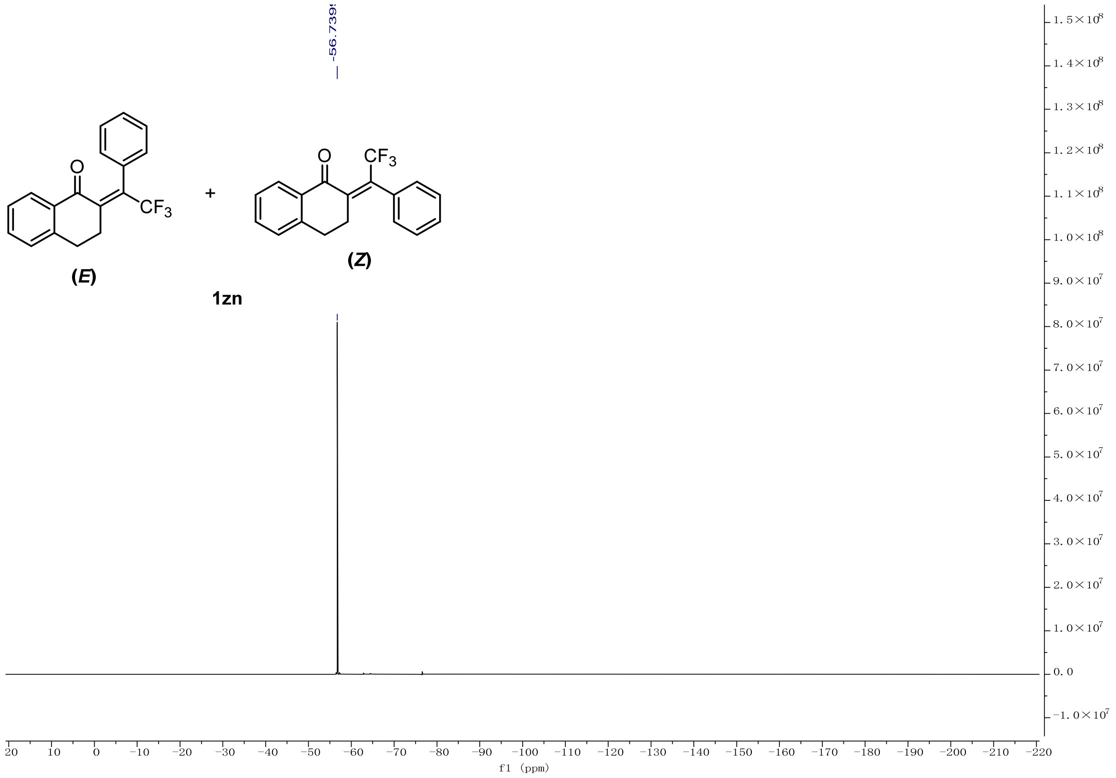

^{1}H NMR (300 MHz, $CDCl_3$) of compound **3a**

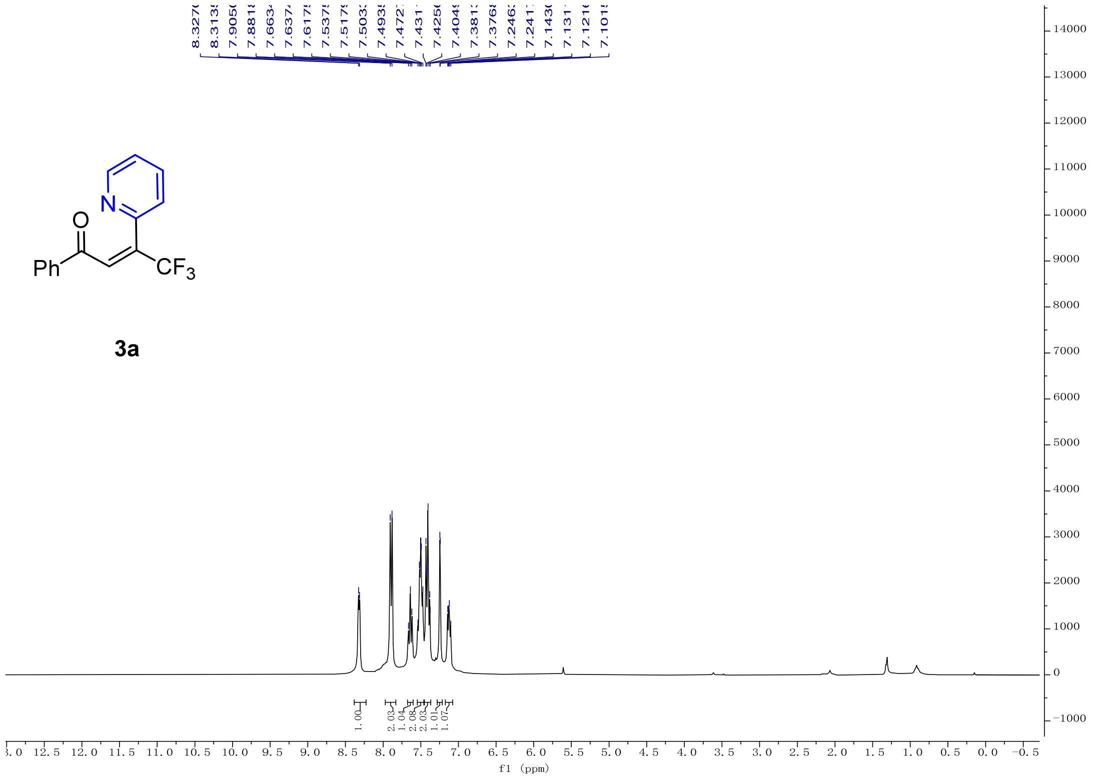

^{13}C NMR (75 MHz, CDCl$_3$) of compound **3a**

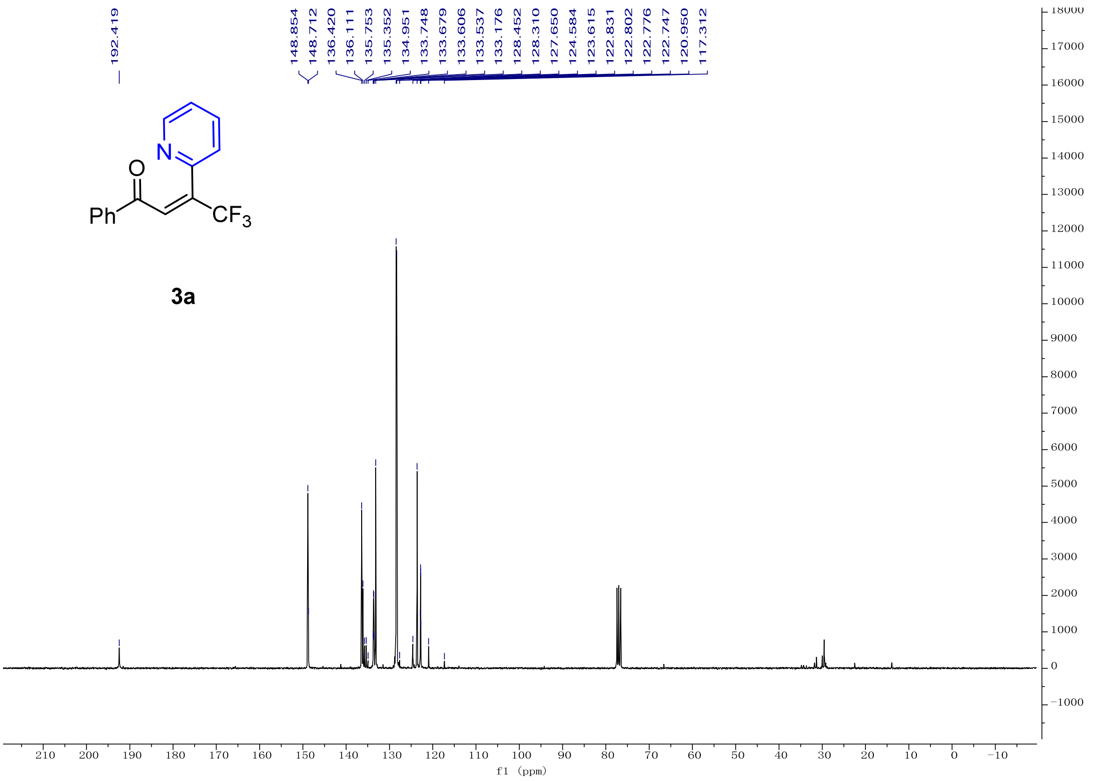

^{19}F NMR (376 MHz, CDCl$_3$) of compound **3a**

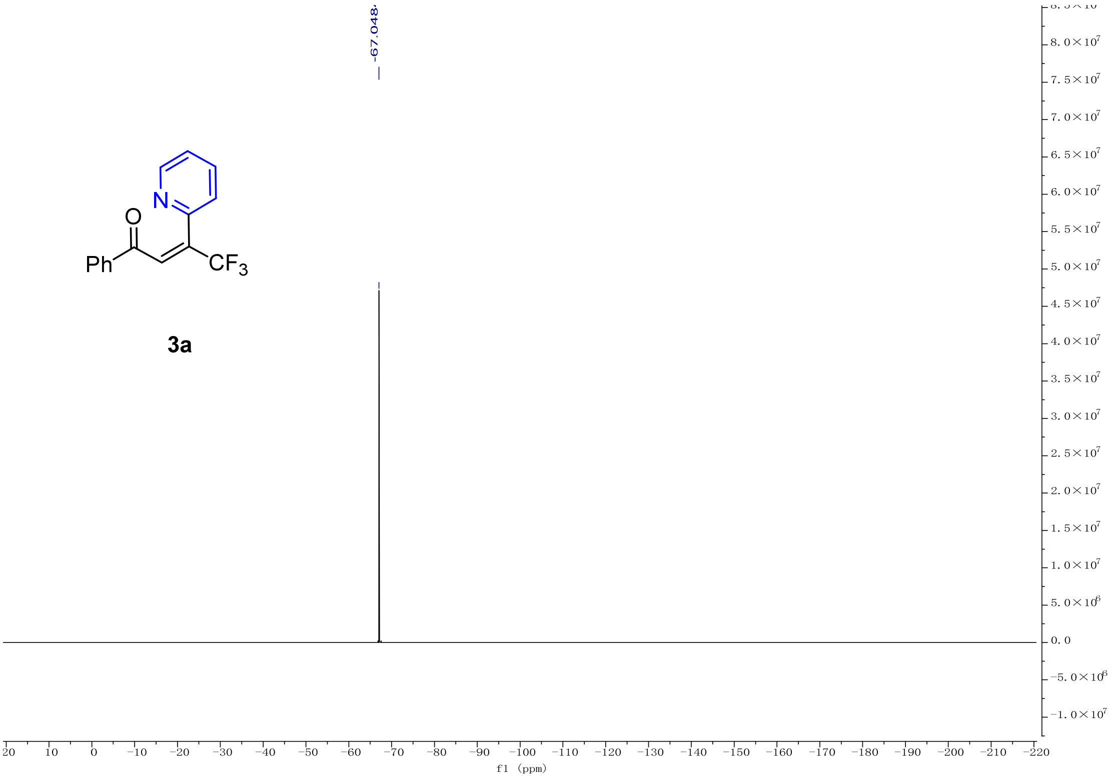

^{1}H NMR (300 MHz, $CDCl_3$) of compound **3b**

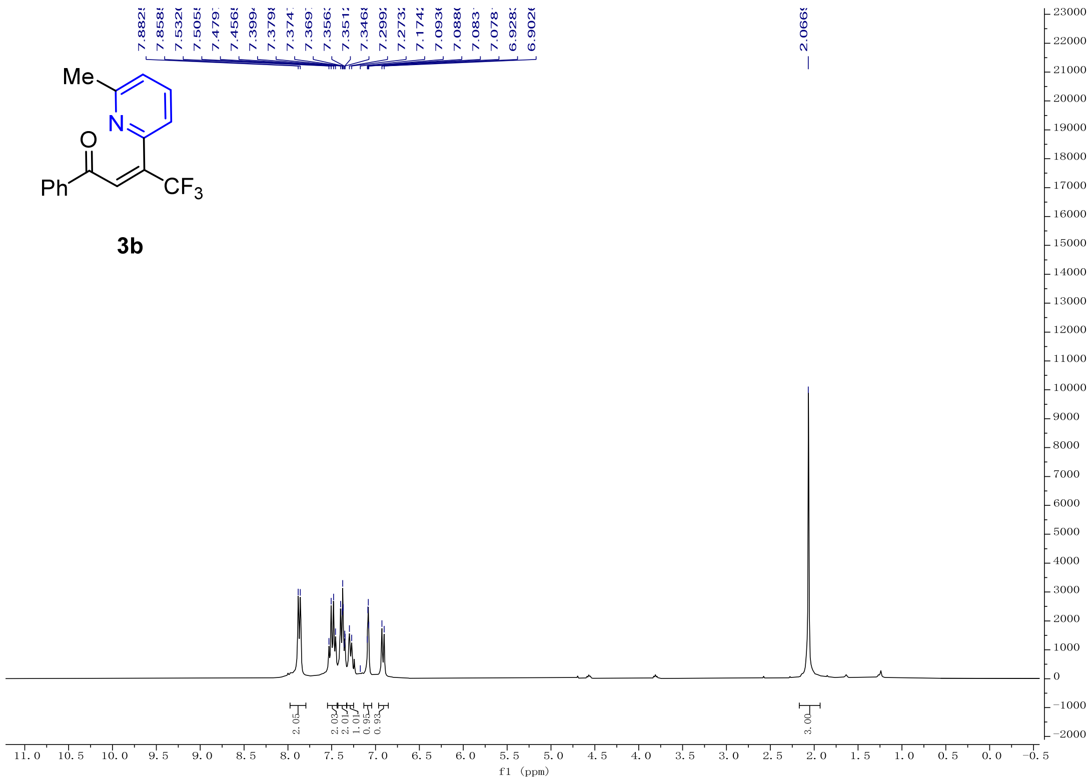

^{13}C NMR (75 MHz, $CDCl_3$) of compound **3b**

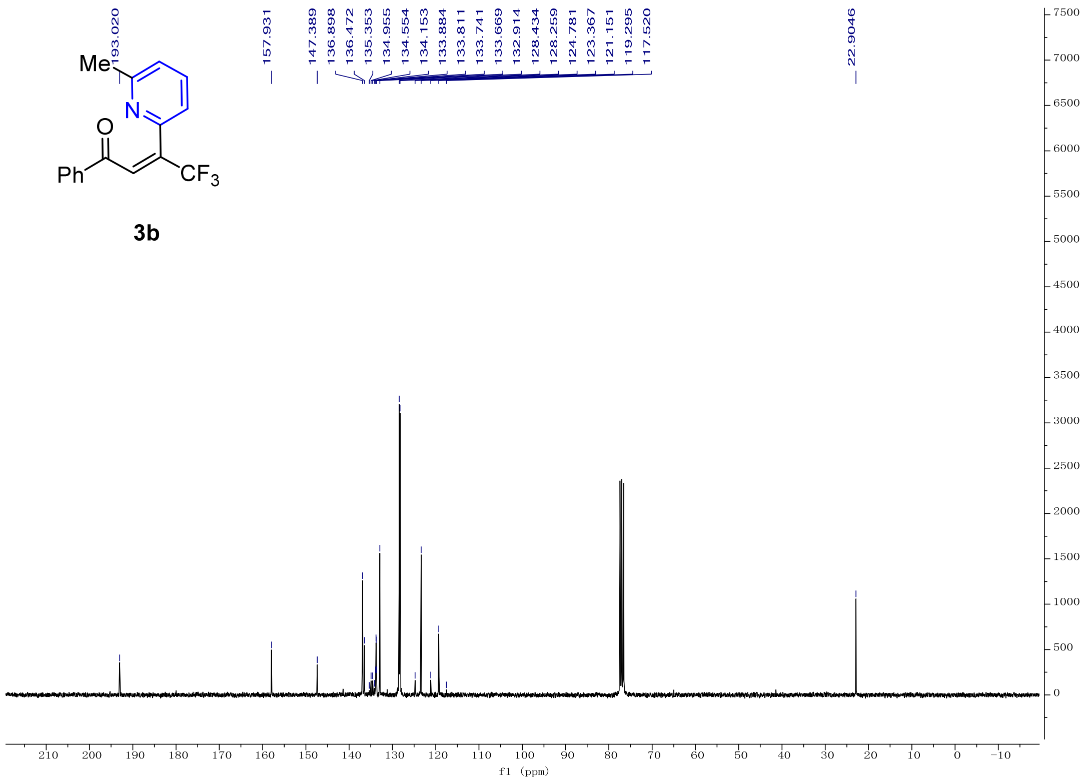

^{19}F NMR (376 MHz, $CDCl_3$) of compound **3b**

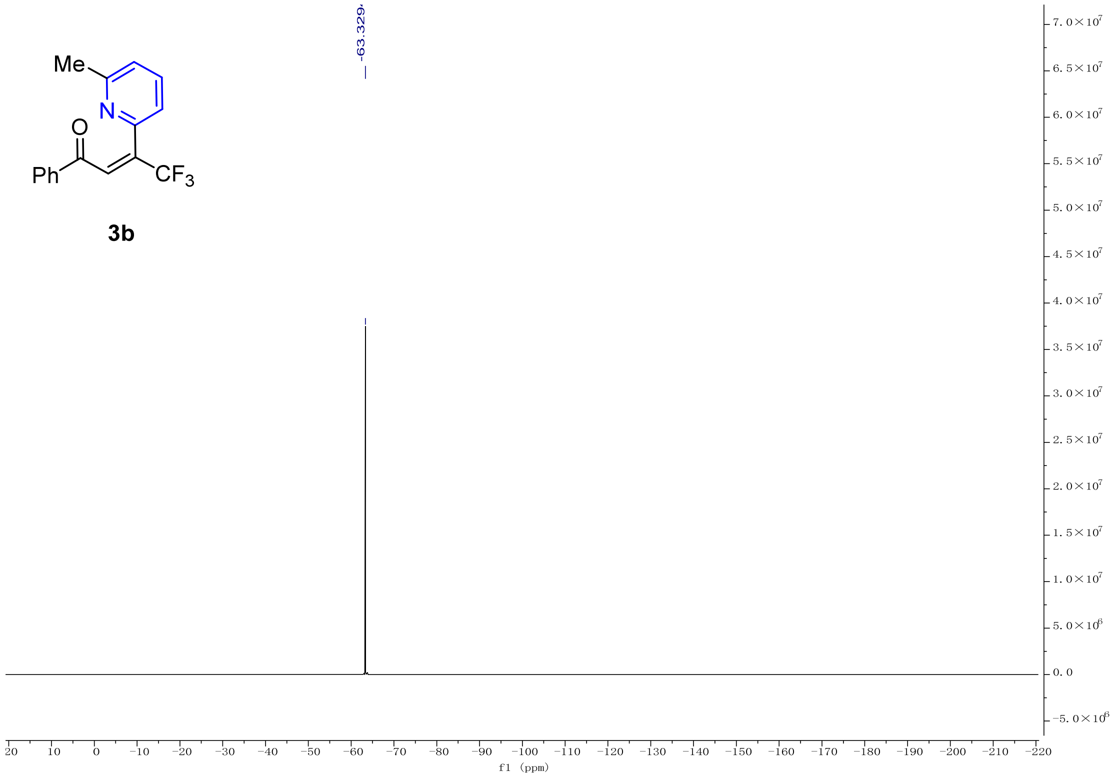

^{1}H NMR (300 MHz, $CDCl_3$) of compound **3c**

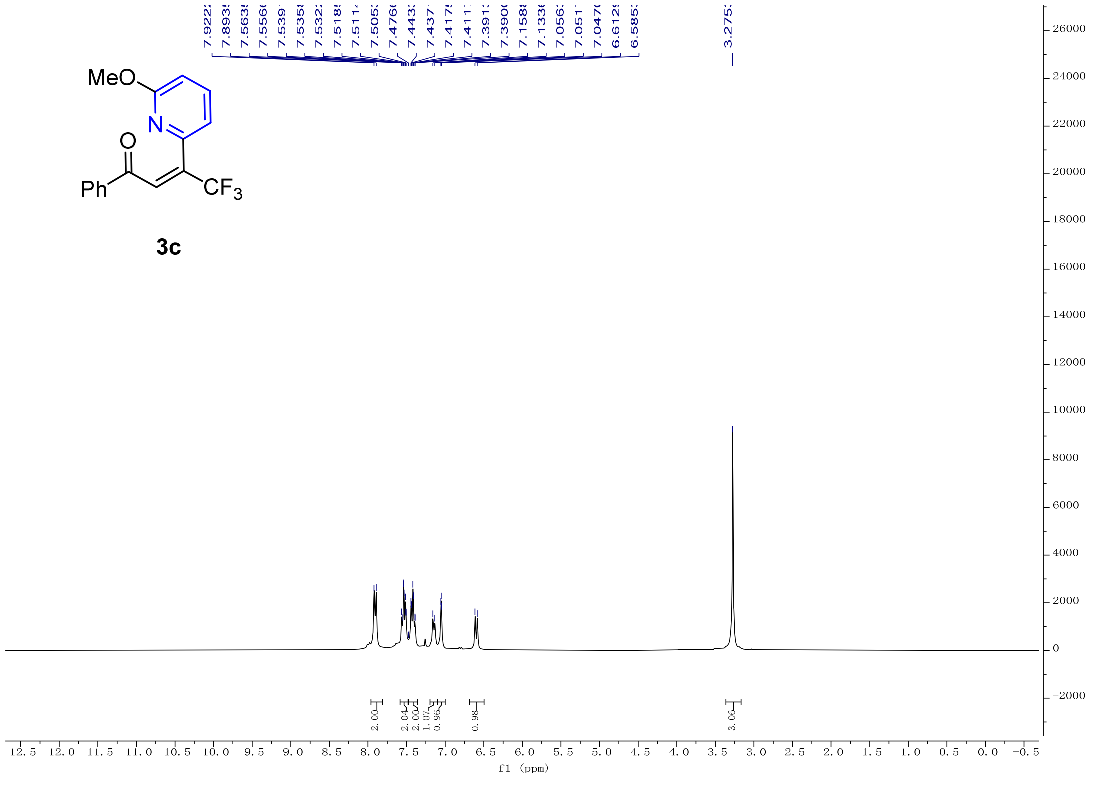

^{13}C NMR (75 MHz, $CDCl_3$) of compound **3c**

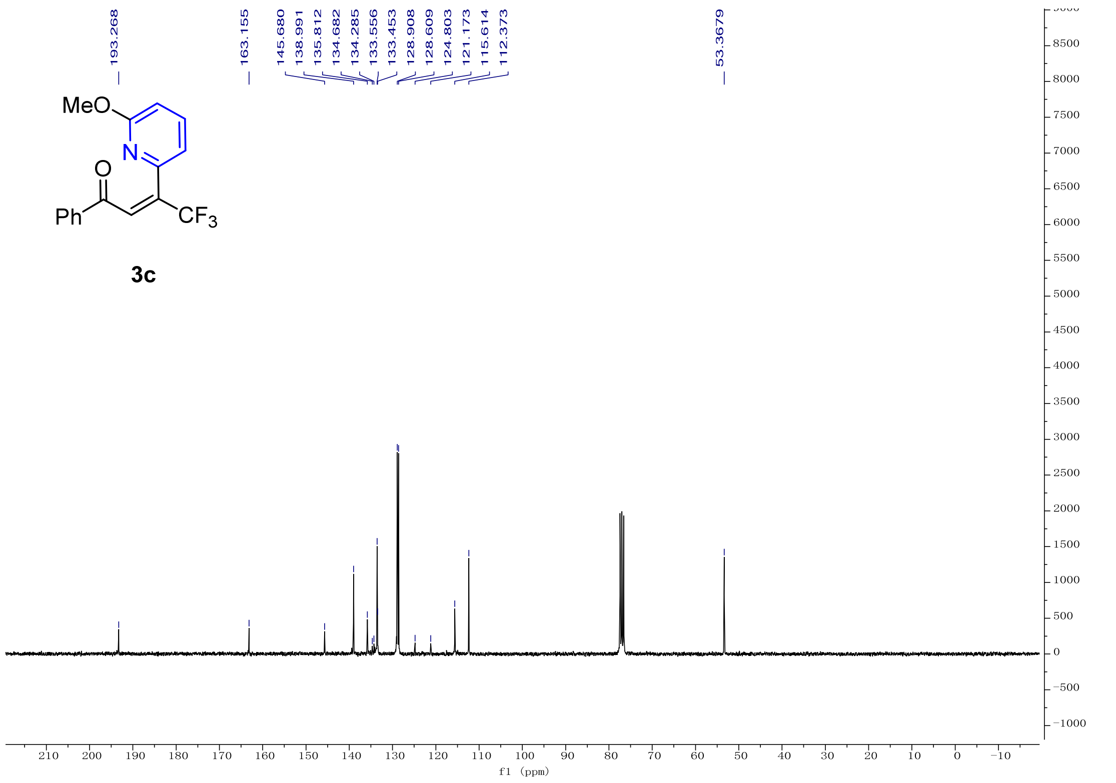

^{19}F NMR (376 MHz, $CDCl_3$) of compound **3c**

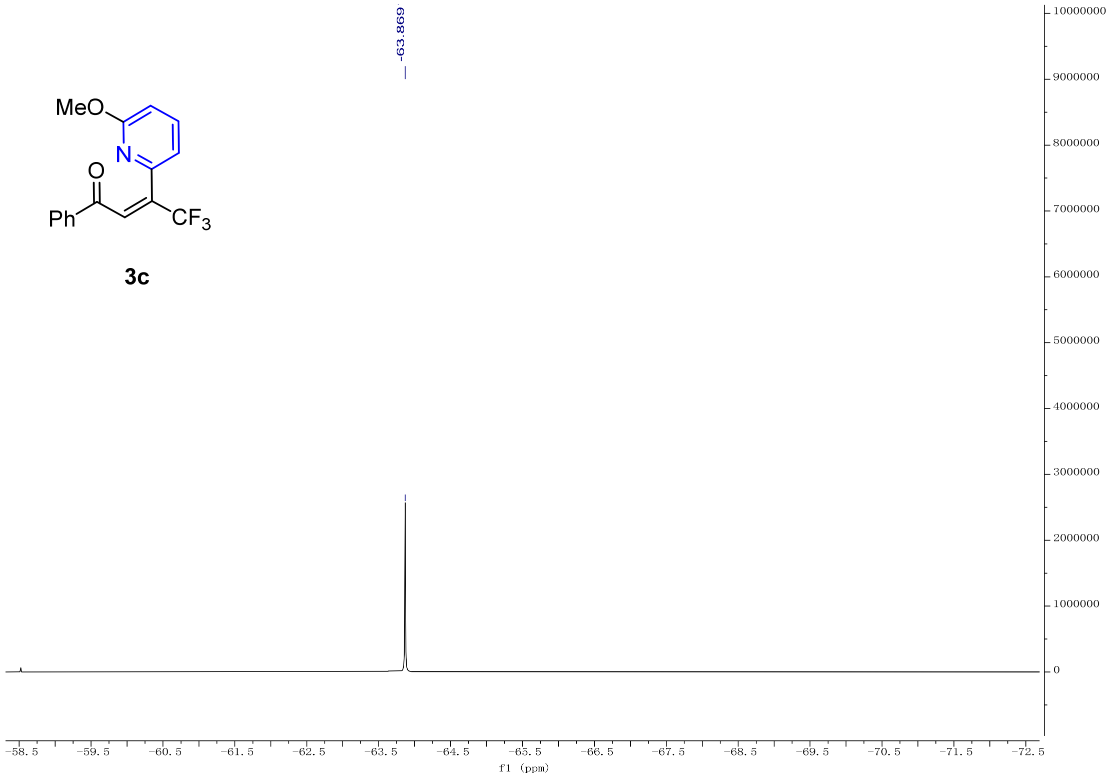

^{1}H NMR (300 MHz, $CDCl_3$) of compound **3d**

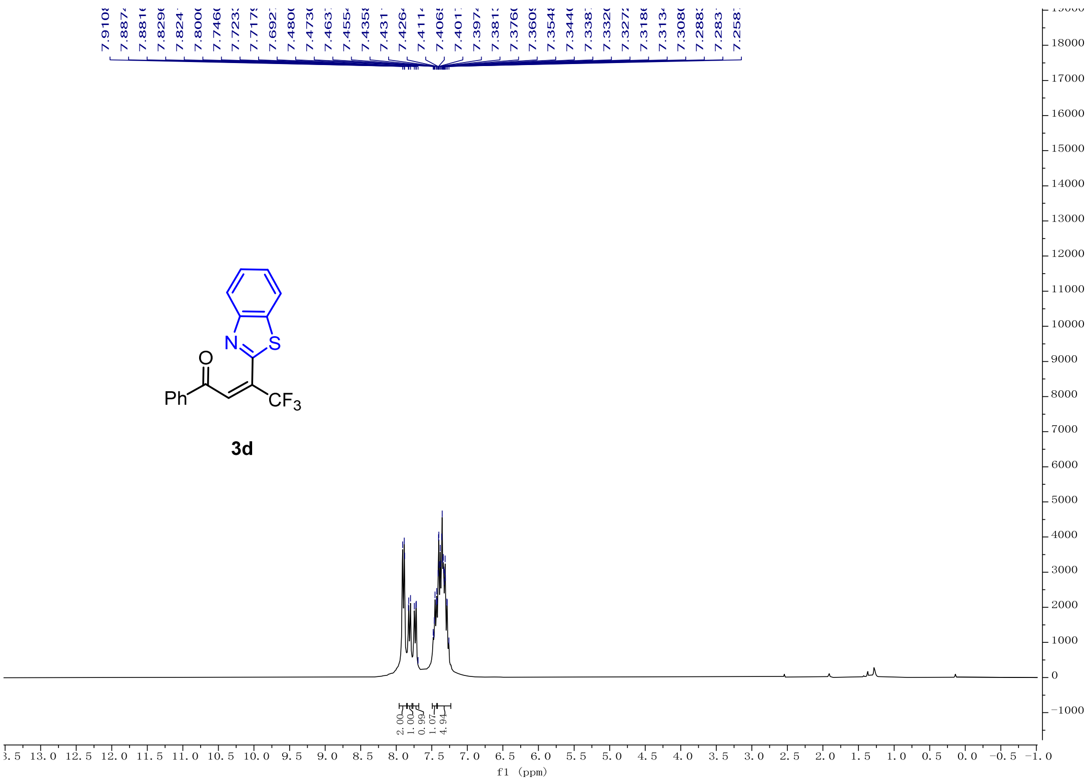

^{13}C NMR (75 MHz, $CDCl_3$) of compound **3d**

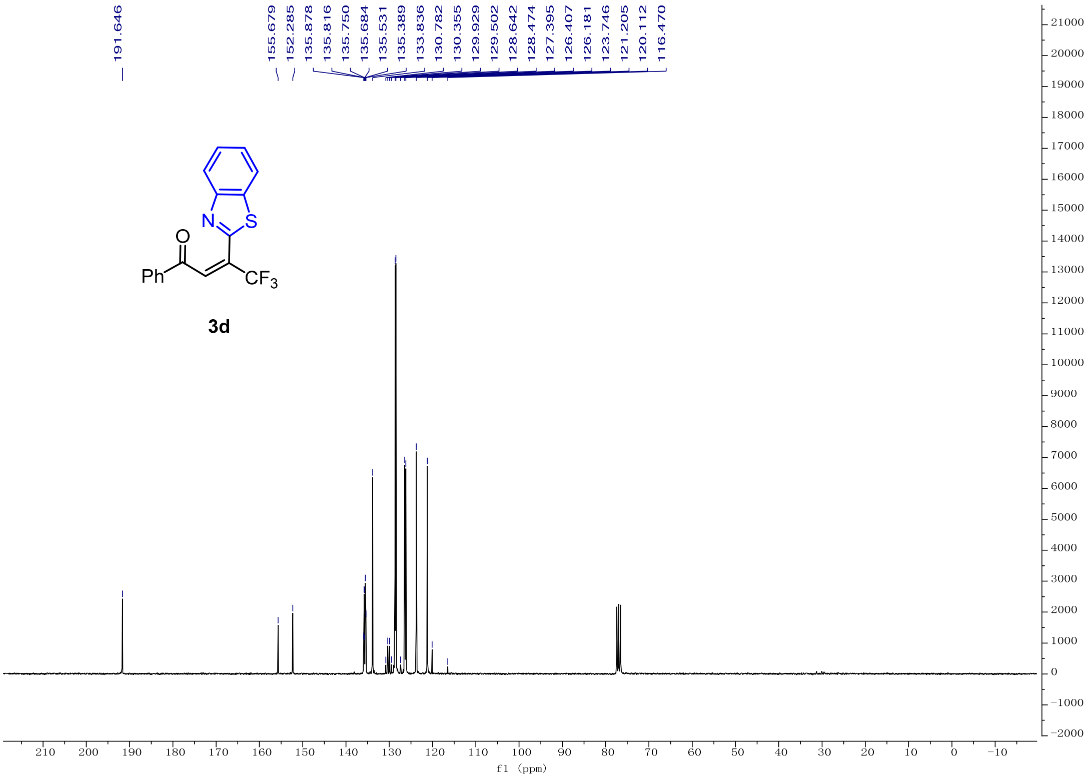

^{19}F NMR (565 MHz, $CDCl_3$) of compound **3d**

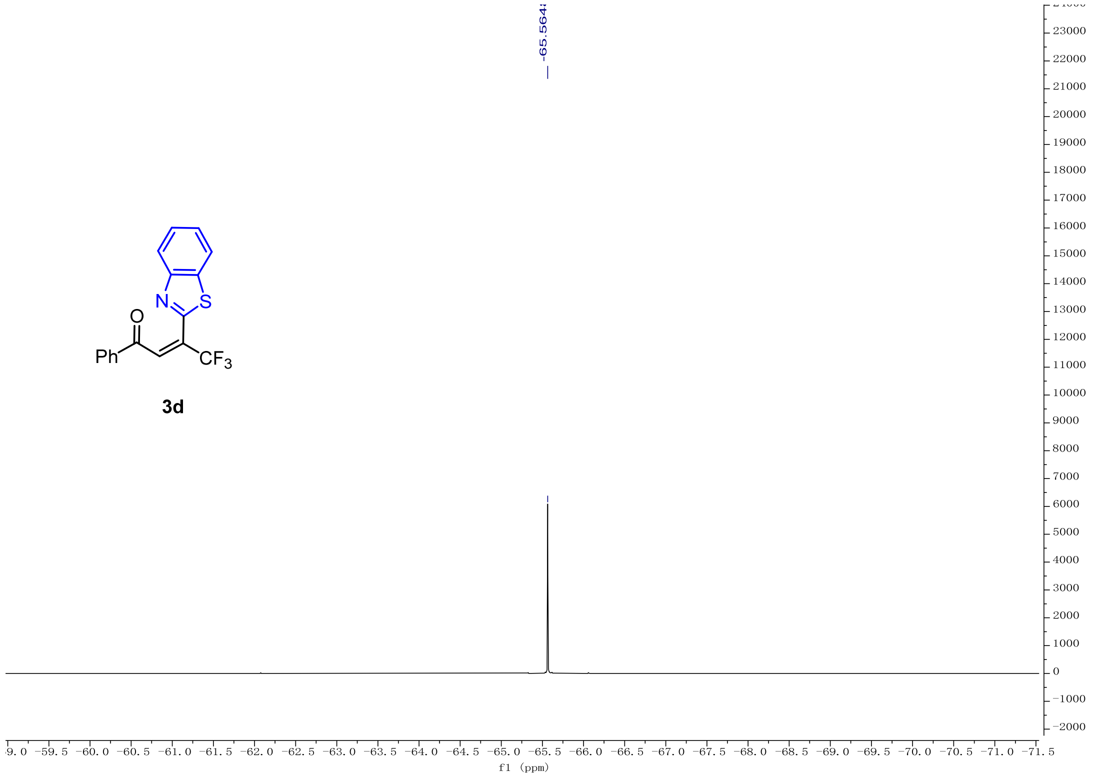

^{1}H NMR (300 MHz, $CDCl_3$) of compound **3e**

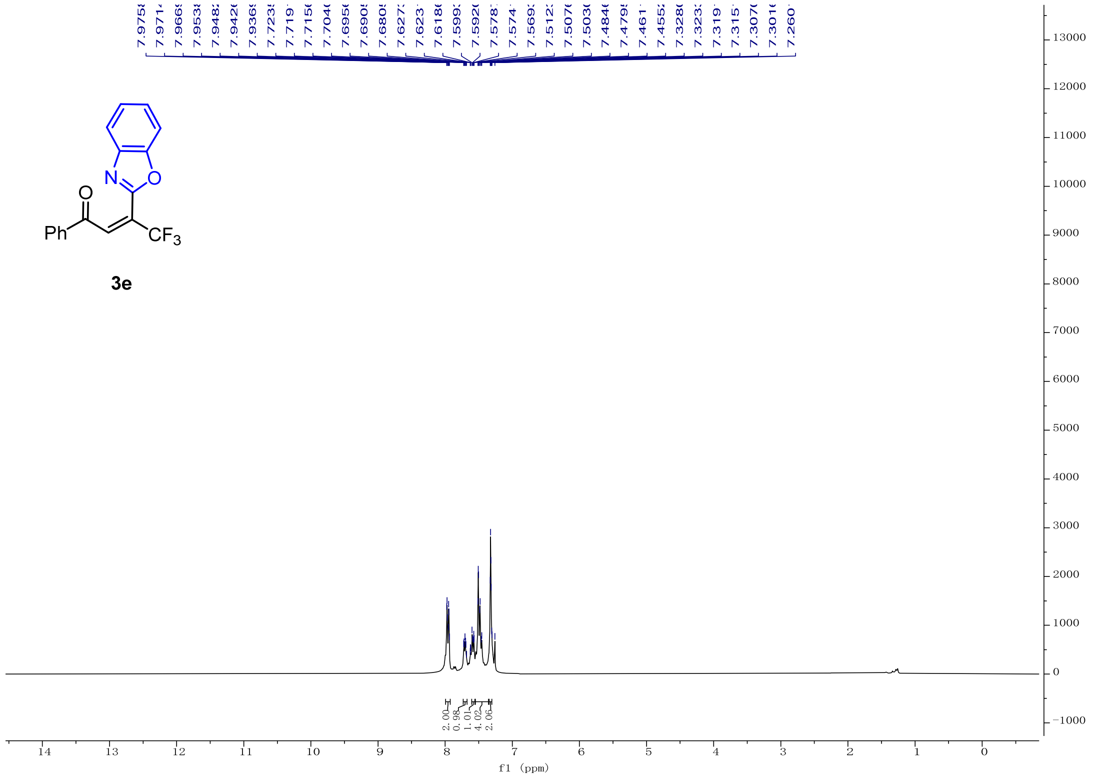

^{13}C NMR (75 MHz, $CDCl_3$) of compound **3e**

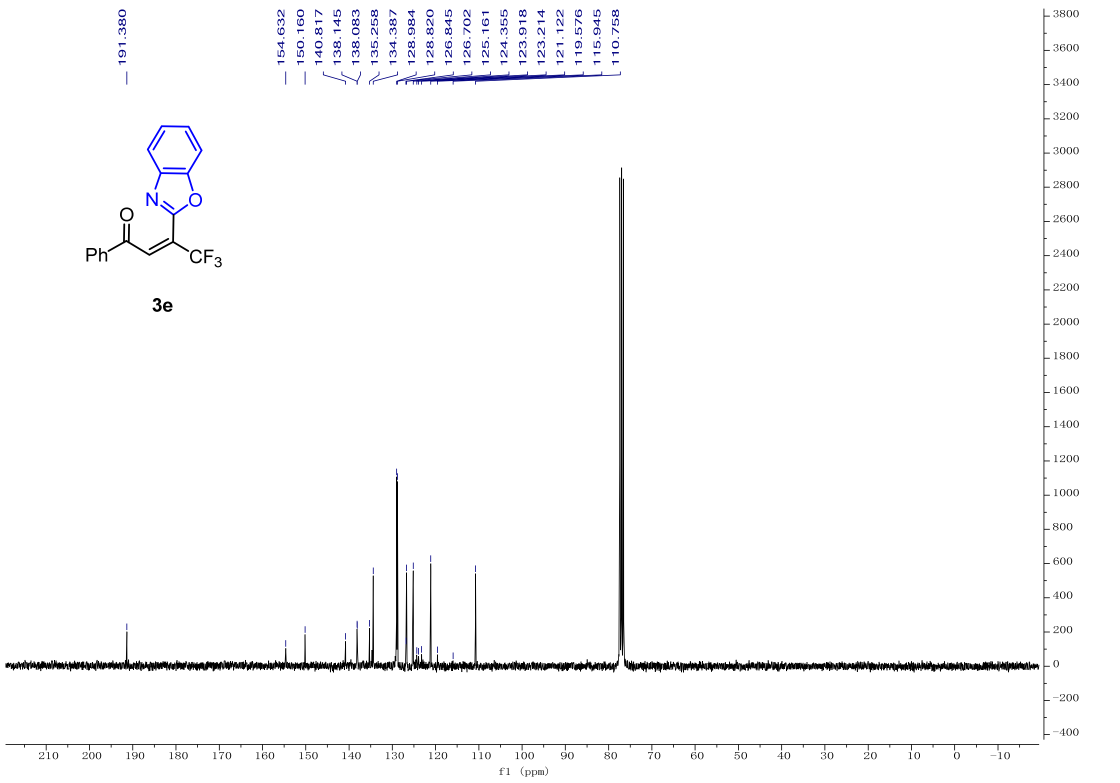

^{19}F NMR (376 MHz, $CDCl_3$) of compound **3e**

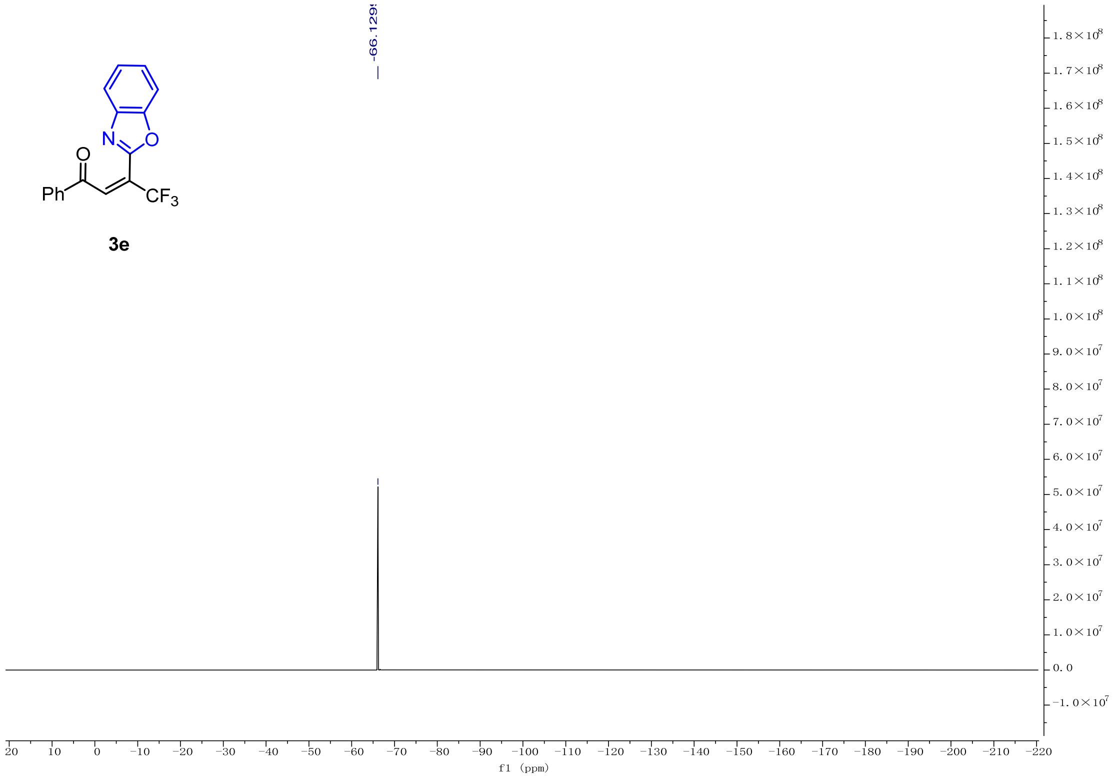

^{1}H NMR (400 MHz, $CDCl_3$) of compound **2a**

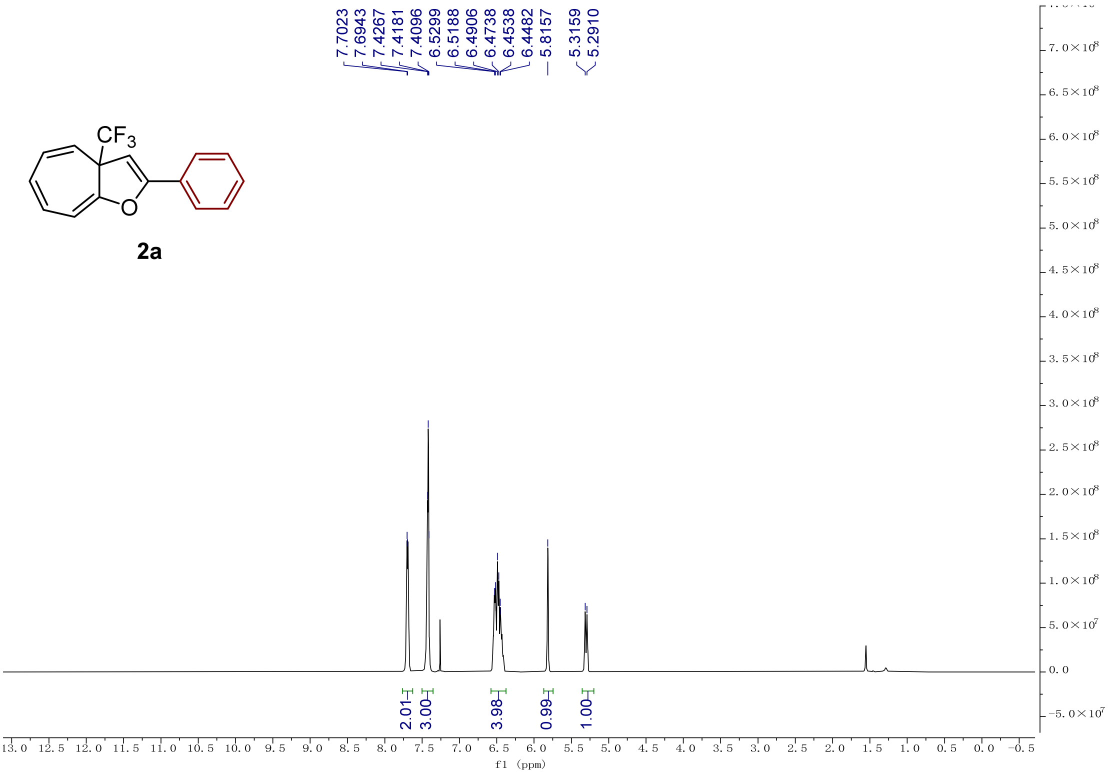

^{13}C NMR (101 MHz, $CDCl_3$) of compound **2a**

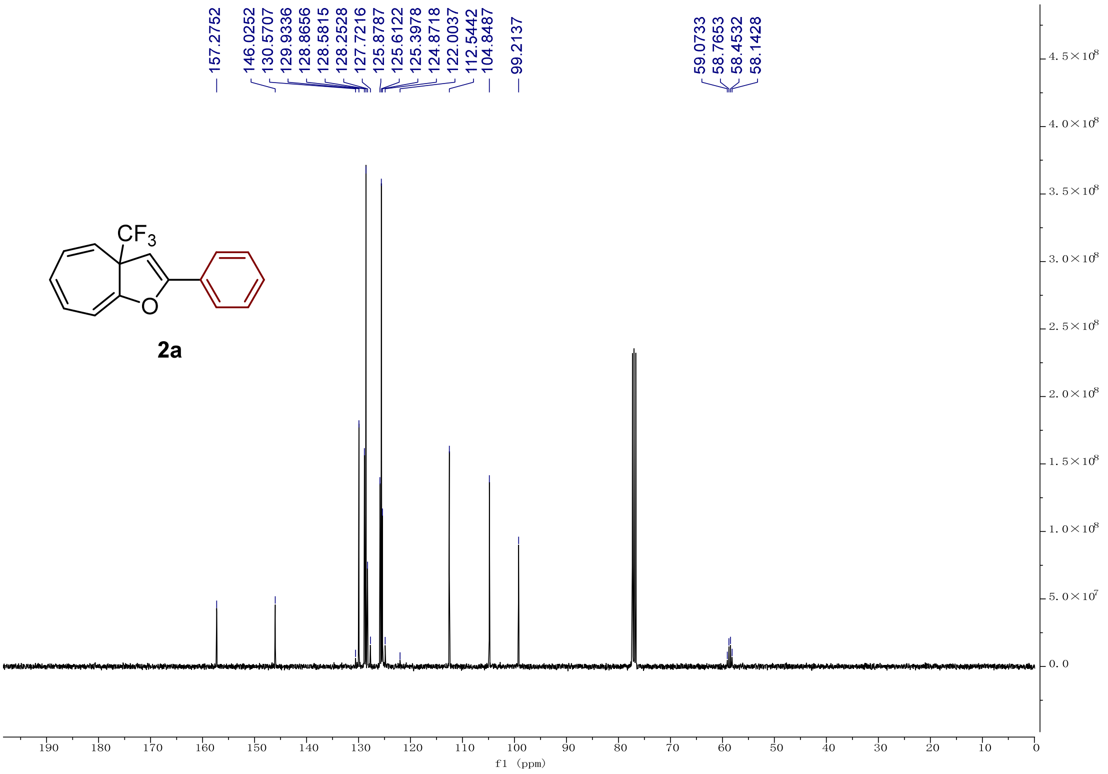

^{19}F NMR (376 MHz, $CDCl_3$) of compound **2a**

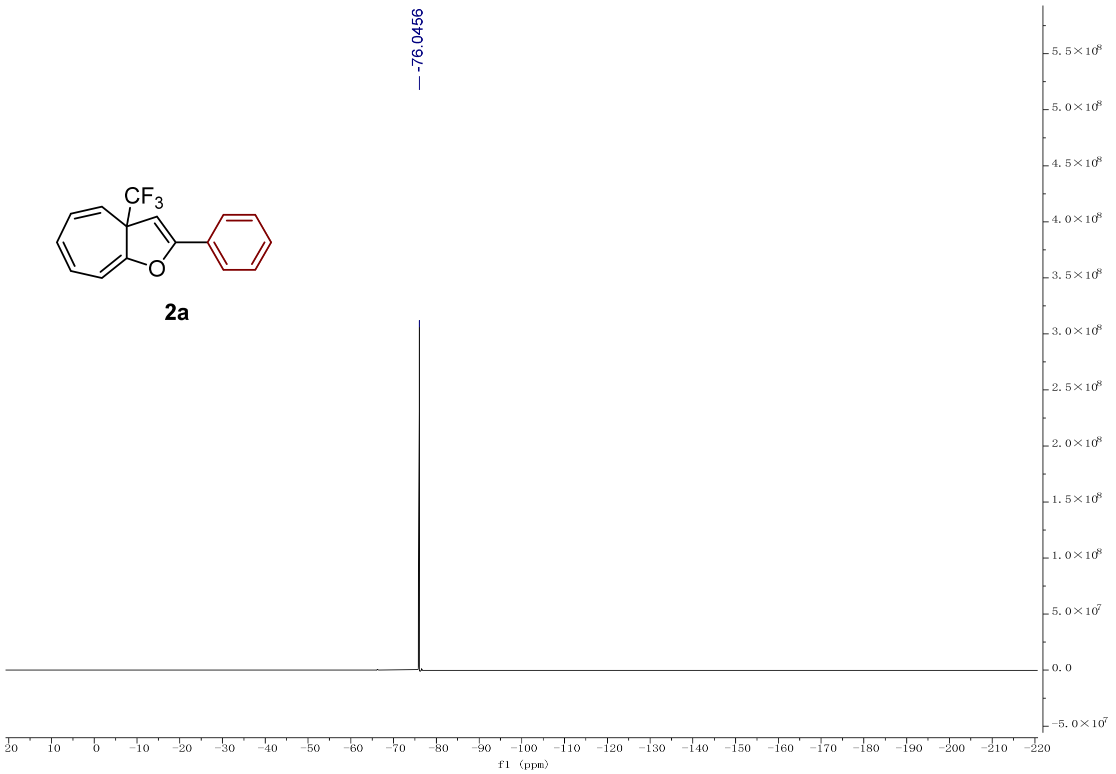

^{1}H NMR (600 MHz, $CDCl_3$) of compound of **2b**

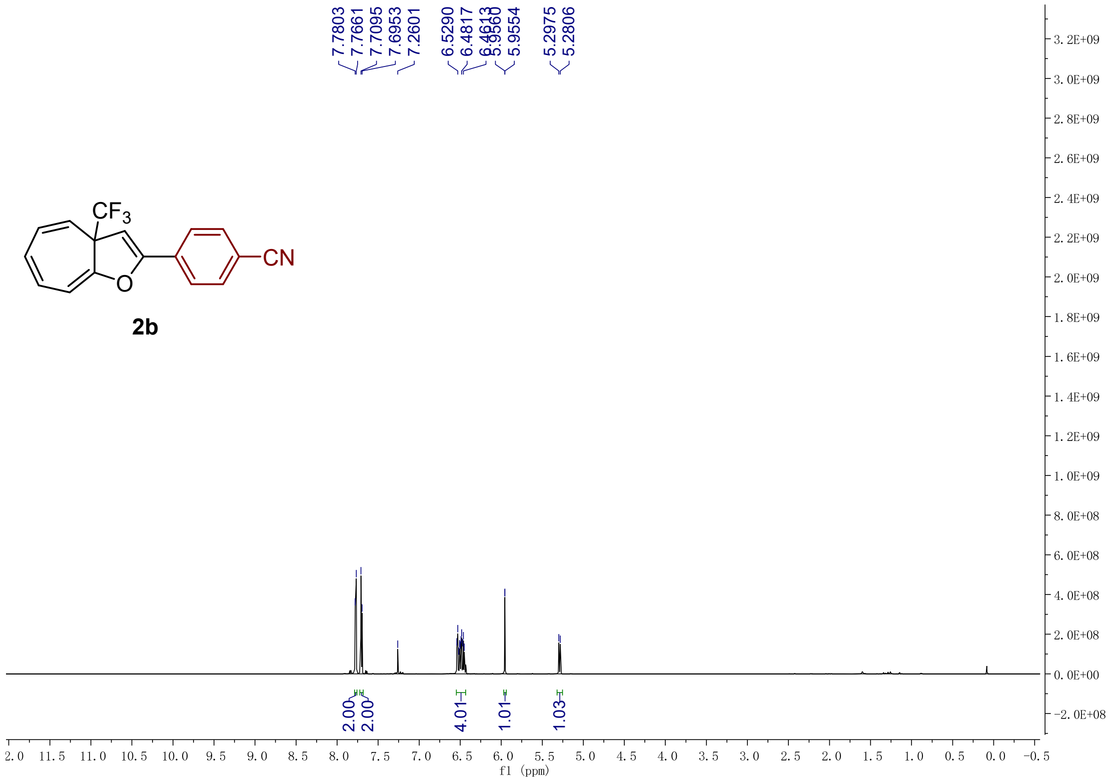

^{13}C NMR (151 MHz, $CDCl_3$) of compound **2b**

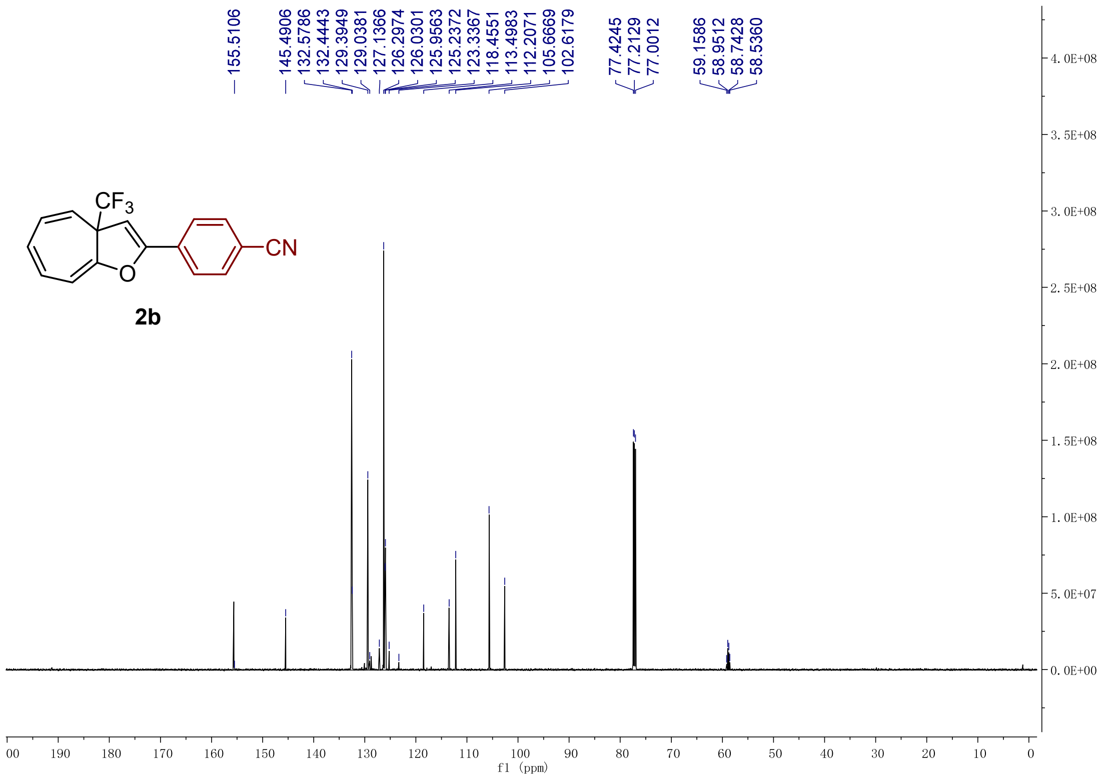

^{19}F NMR (565 MHz, $CDCl_3$) of compound of **2b**

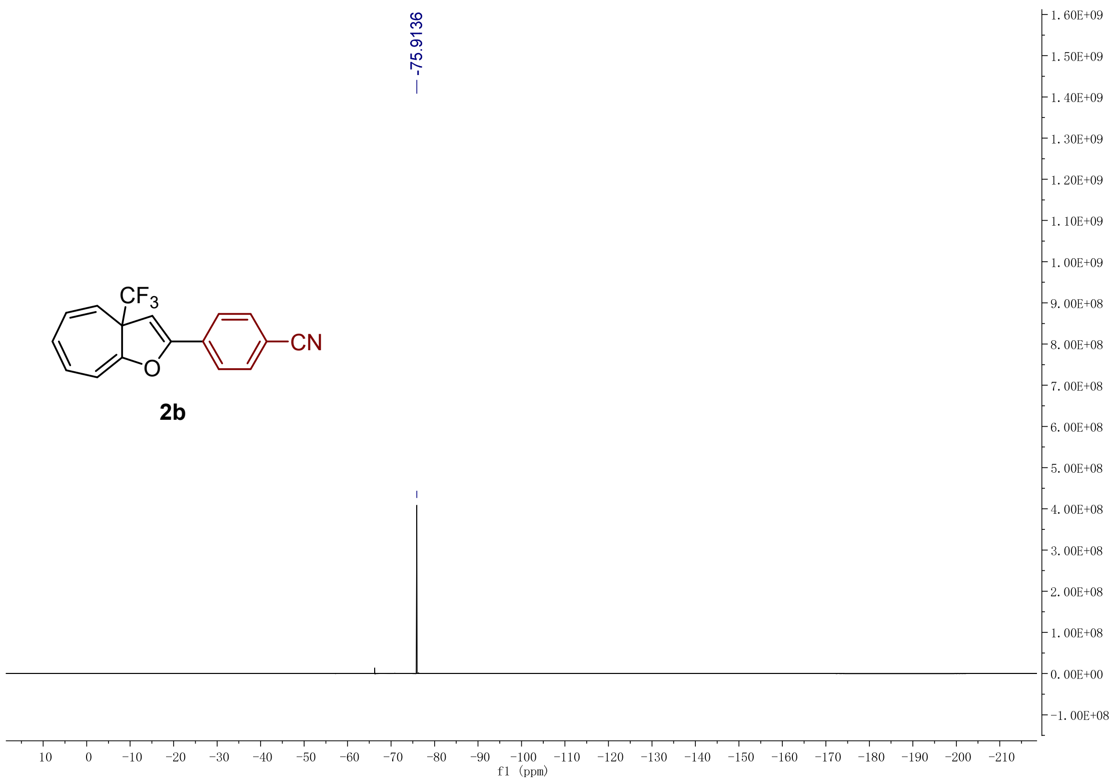

^{1}H NMR (300 MHz, $CDCl_3$) of compound of **2c**

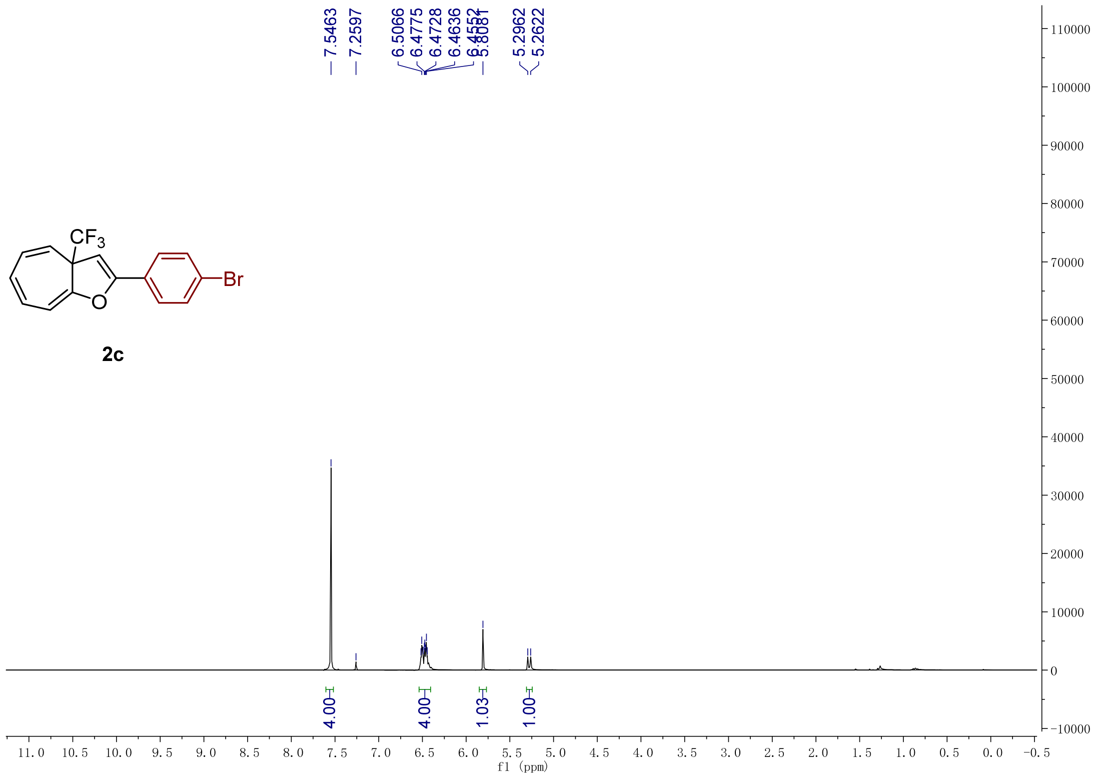

^{13}C NMR (75 MHz, $CDCl_3$) of compound **2c**

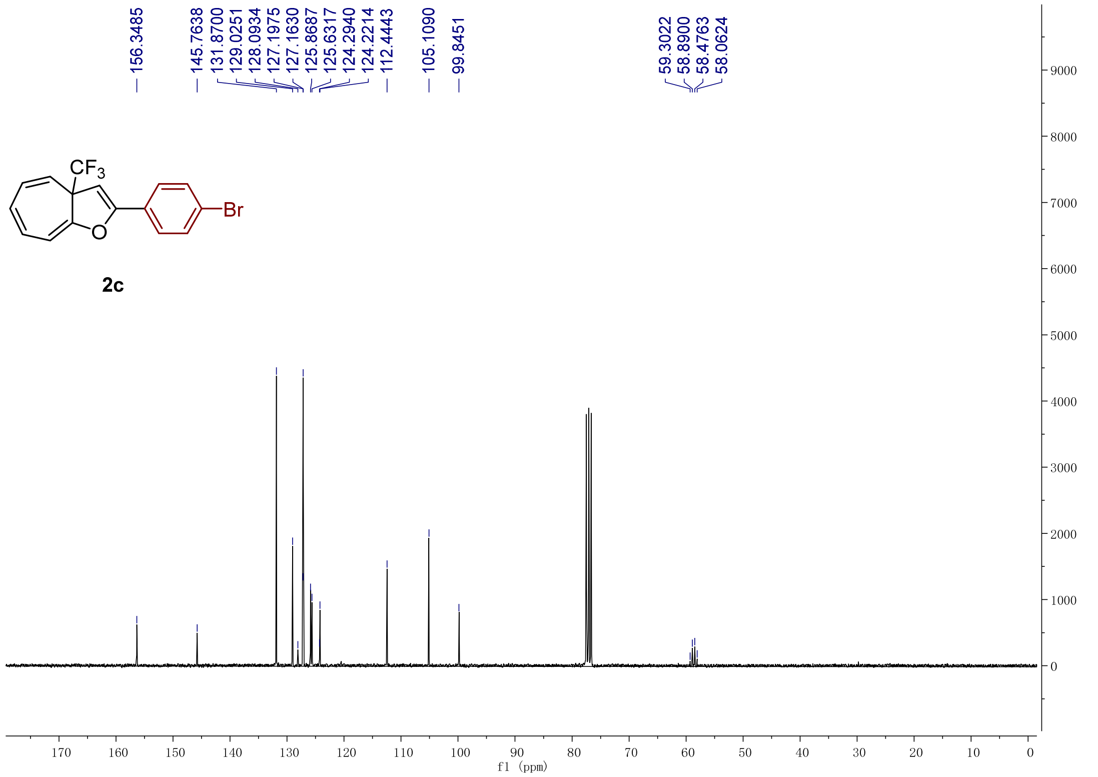

^{19}F NMR (376 MHz, $CDCl_3$) of compound **2c**

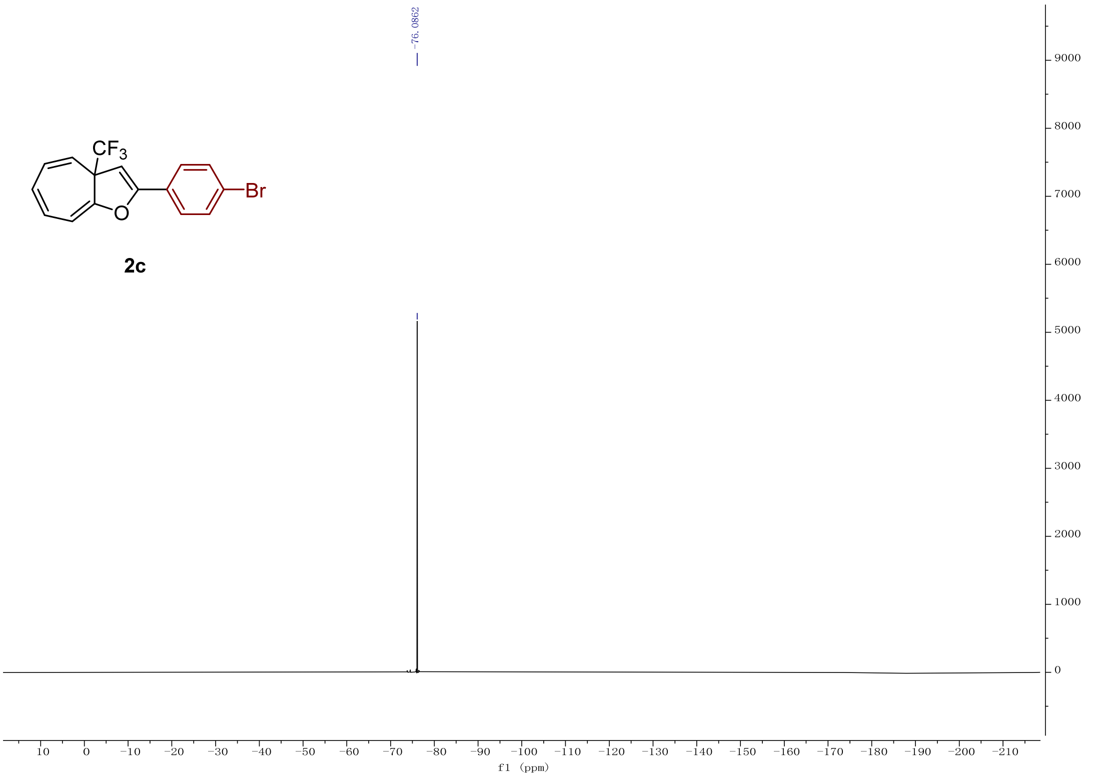

^{1}H NMR (300 MHz, $CDCl_3$) of compound **2d**

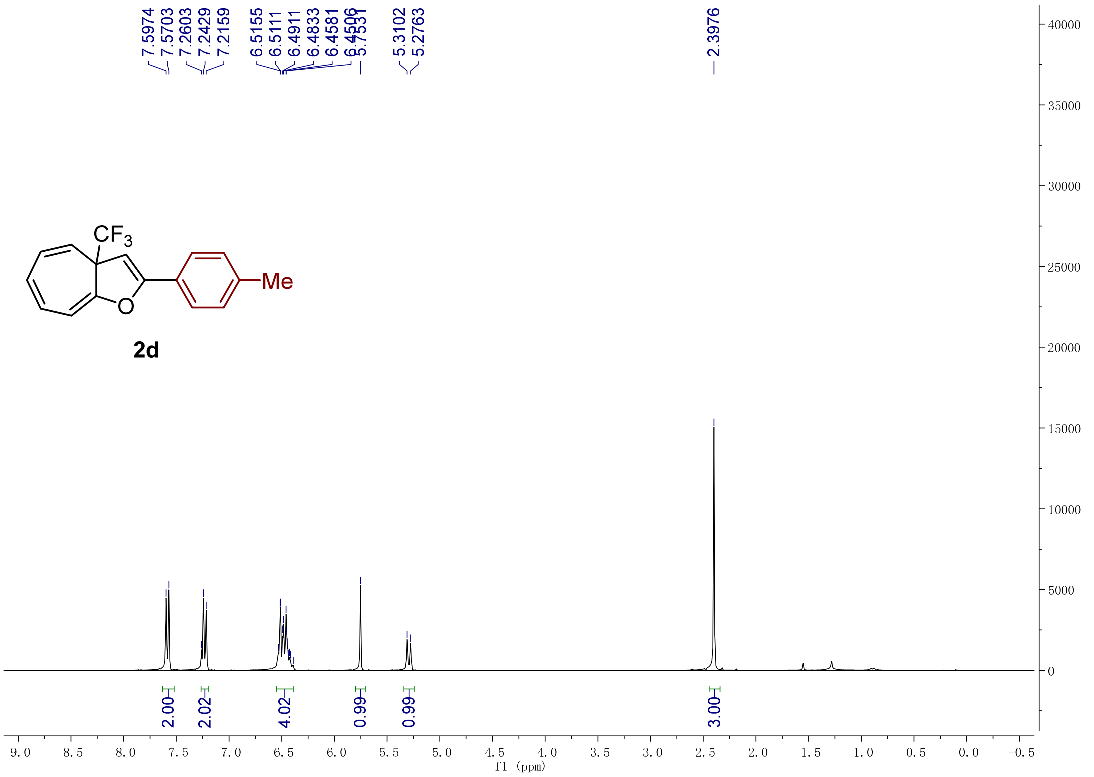

^{13}C NMR (75 MHz, $CDCl_3$) of compound **2d**

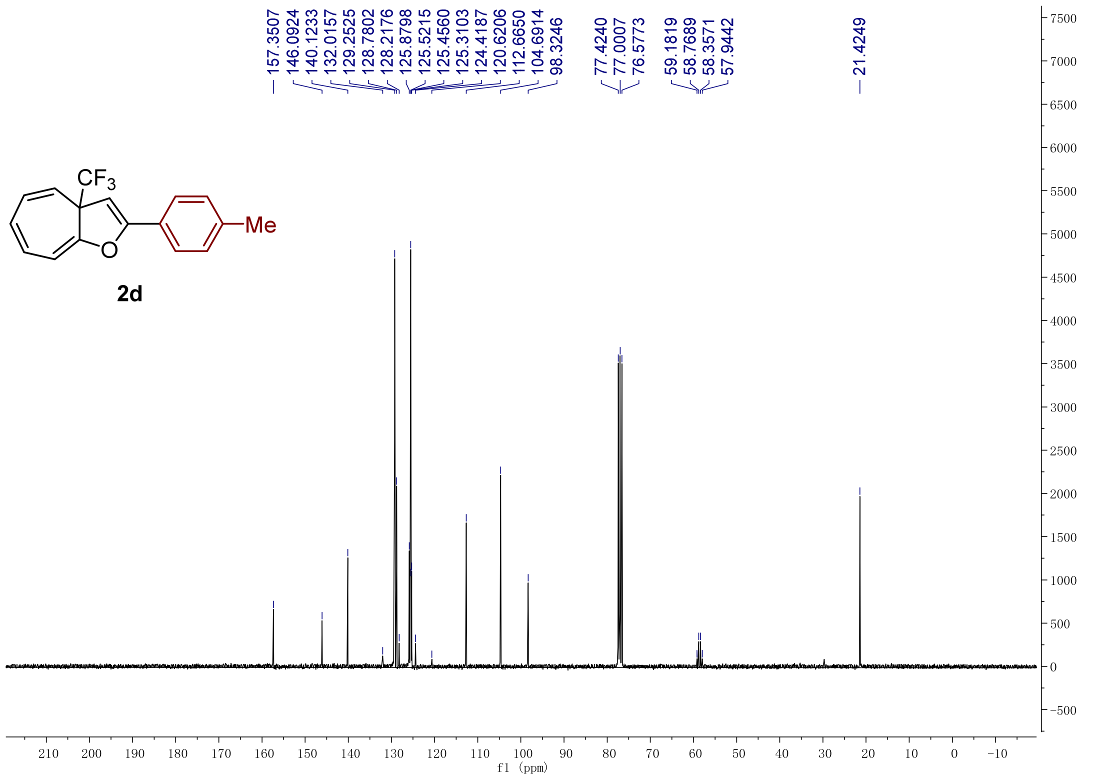

^{19}F NMR (565 MHz, $CDCl_3$) of compound **2d**

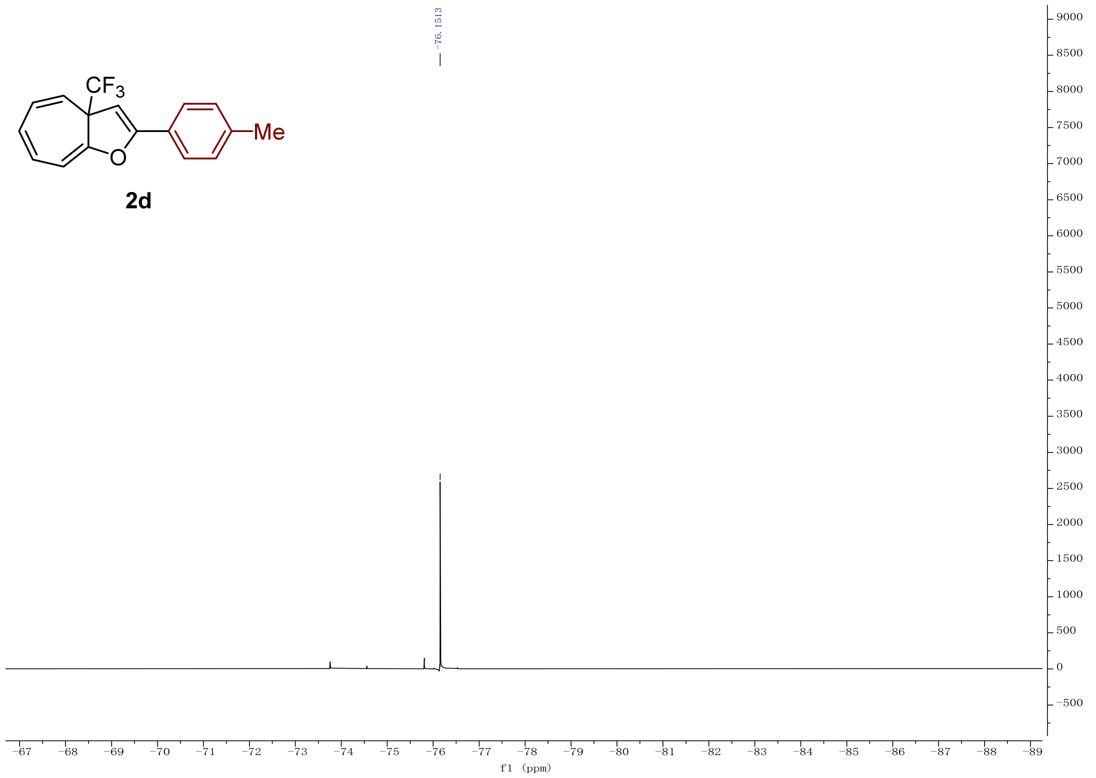

^{1}H NMR (300 MHz, $CDCl_3$) of compound **2e**

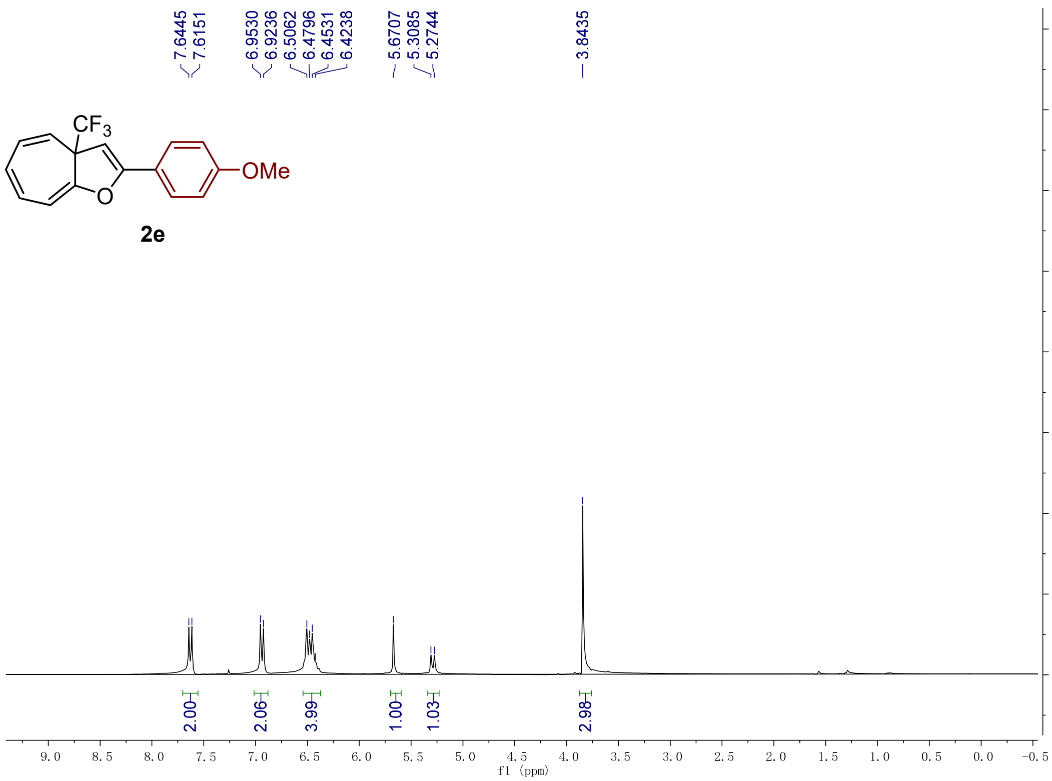

^{13}C NMR (75 MHz, $CDCl_3$) of compound **2e**

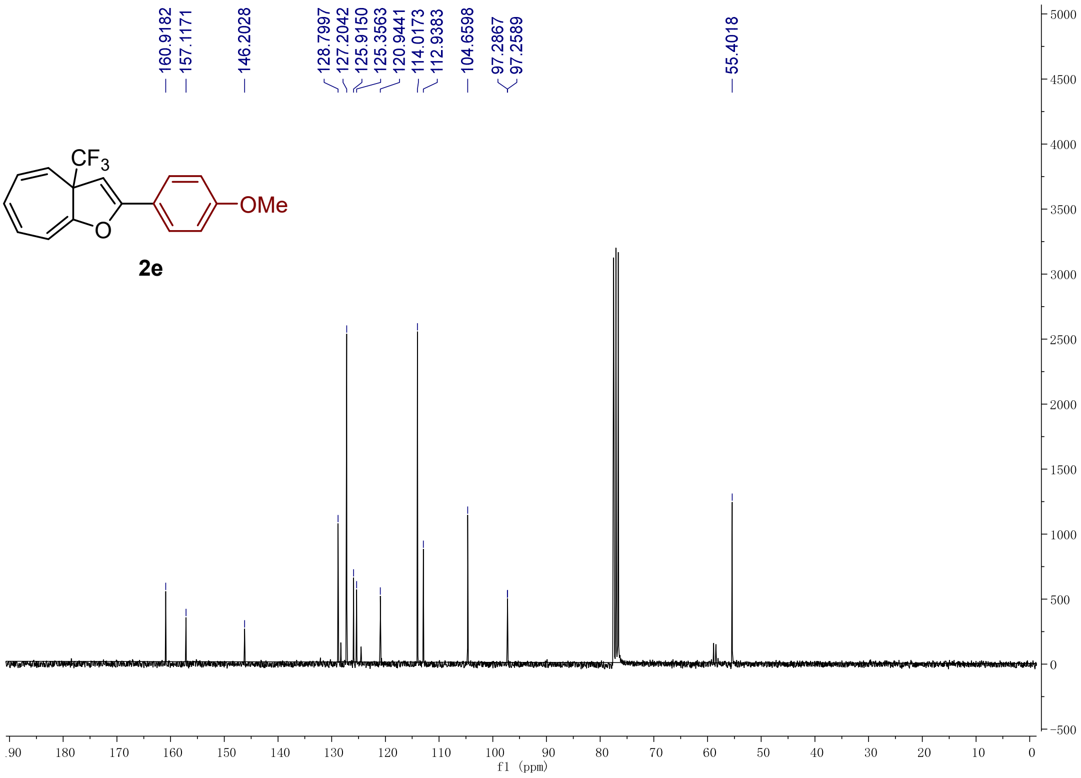

^{19}F NMR (376 MHz, CD_2Cl_2) of compound **2e**

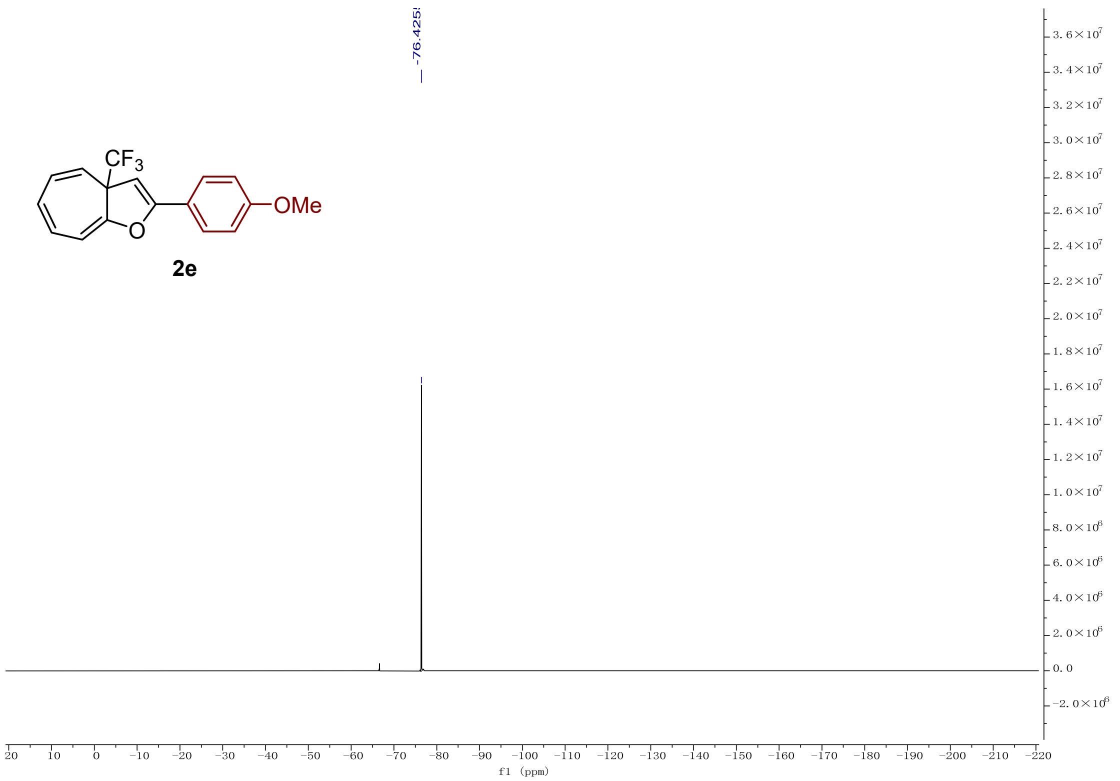

^{1}H NMR (600 MHz, $CDCl_3$) of compound **2f**

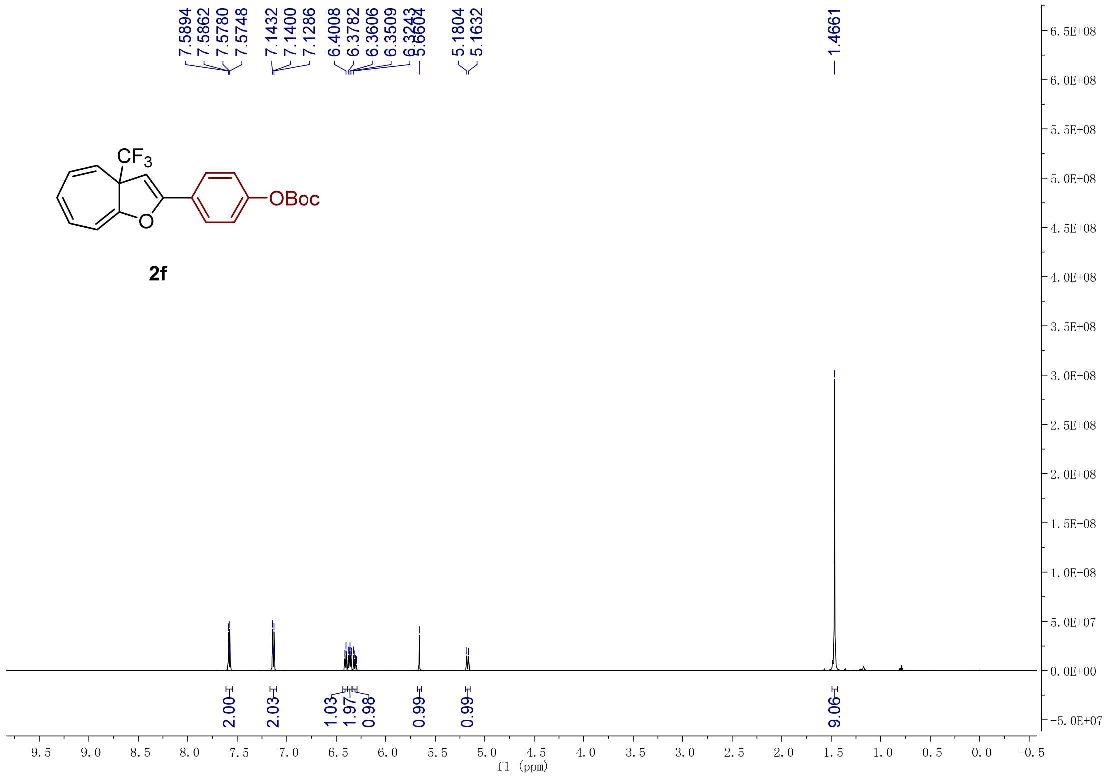

^{13}C NMR (151 MHz, $CDCl_3$) of compound **2f**

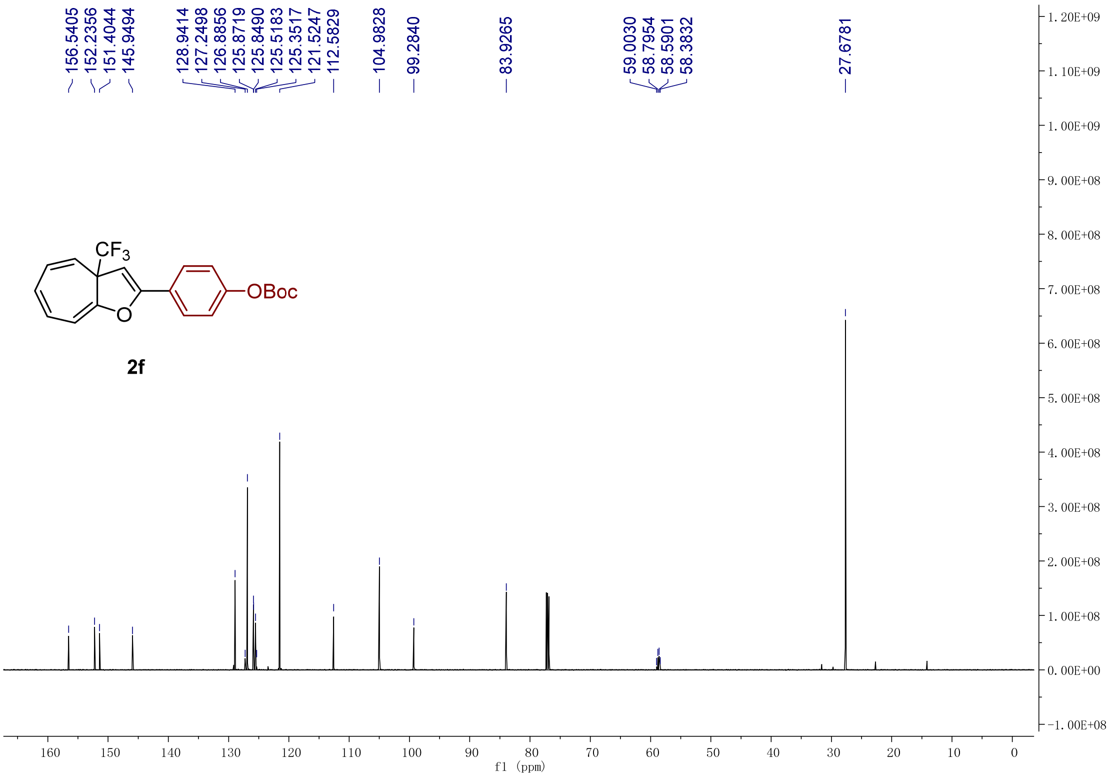

^{19}F NMR (565 MHz, $CDCl_3$) of compound **2f**

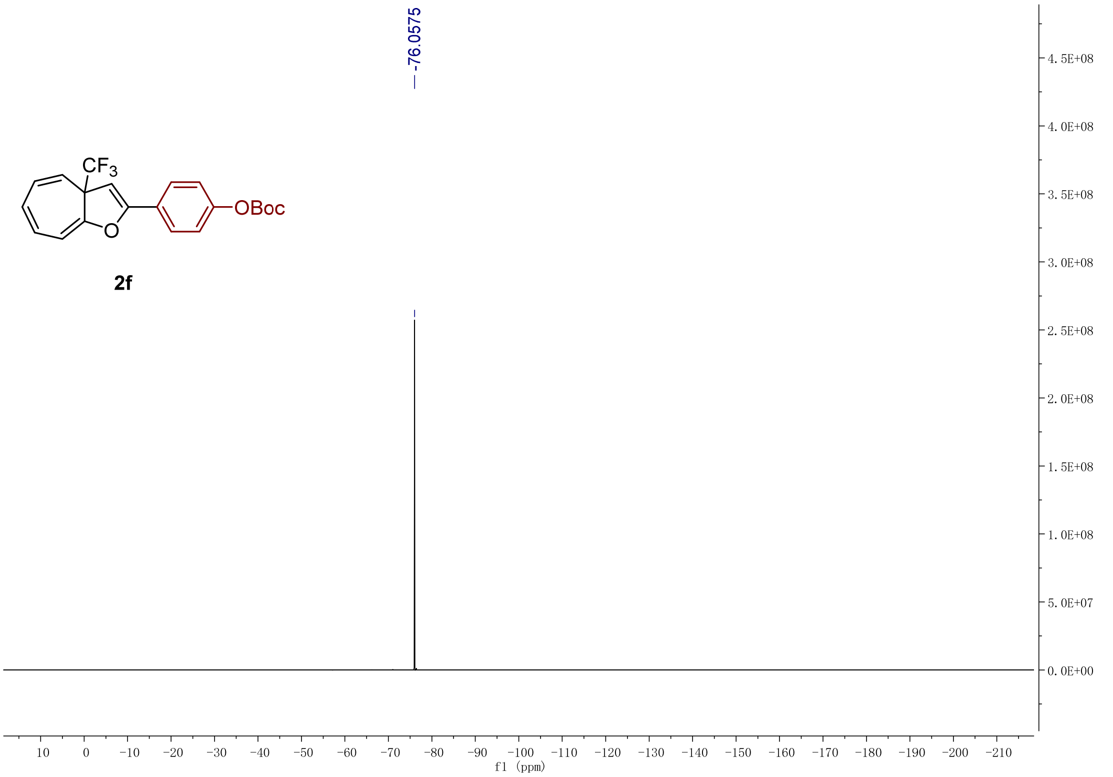

^{1}H NMR (400 MHz, $CDCl_3$) of compound **2g**

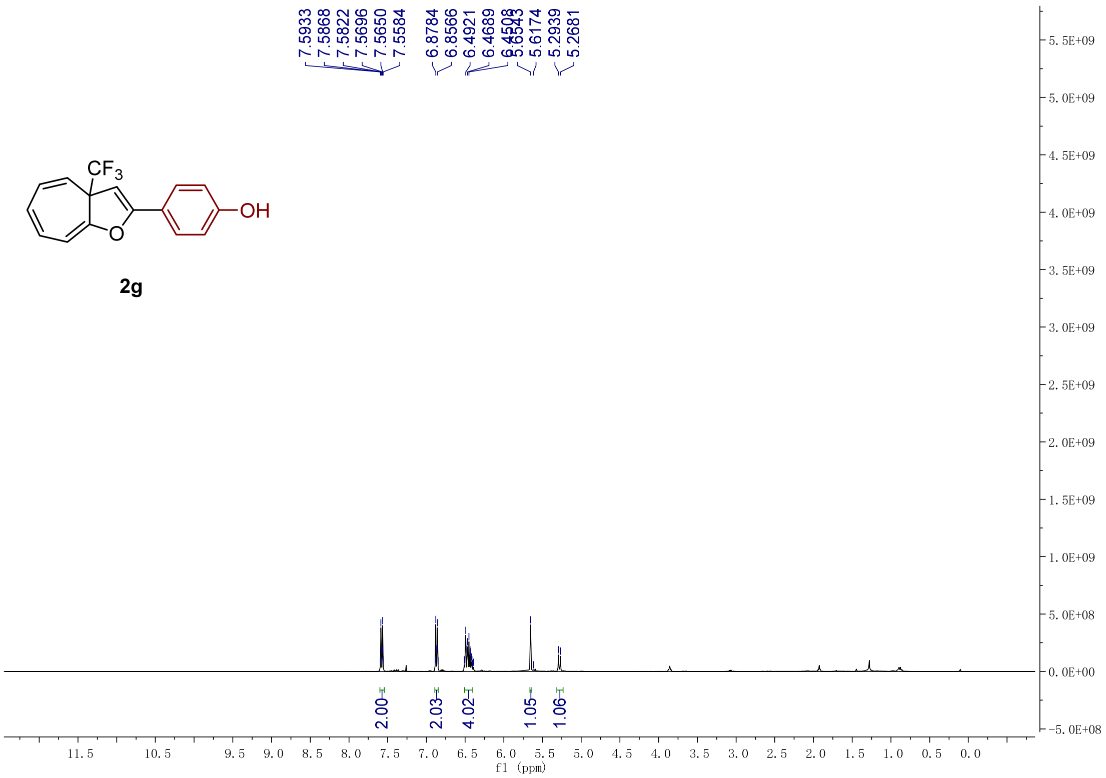

^{13}C NMR (101 MHz, $CDCl_3$) of compound **2g**

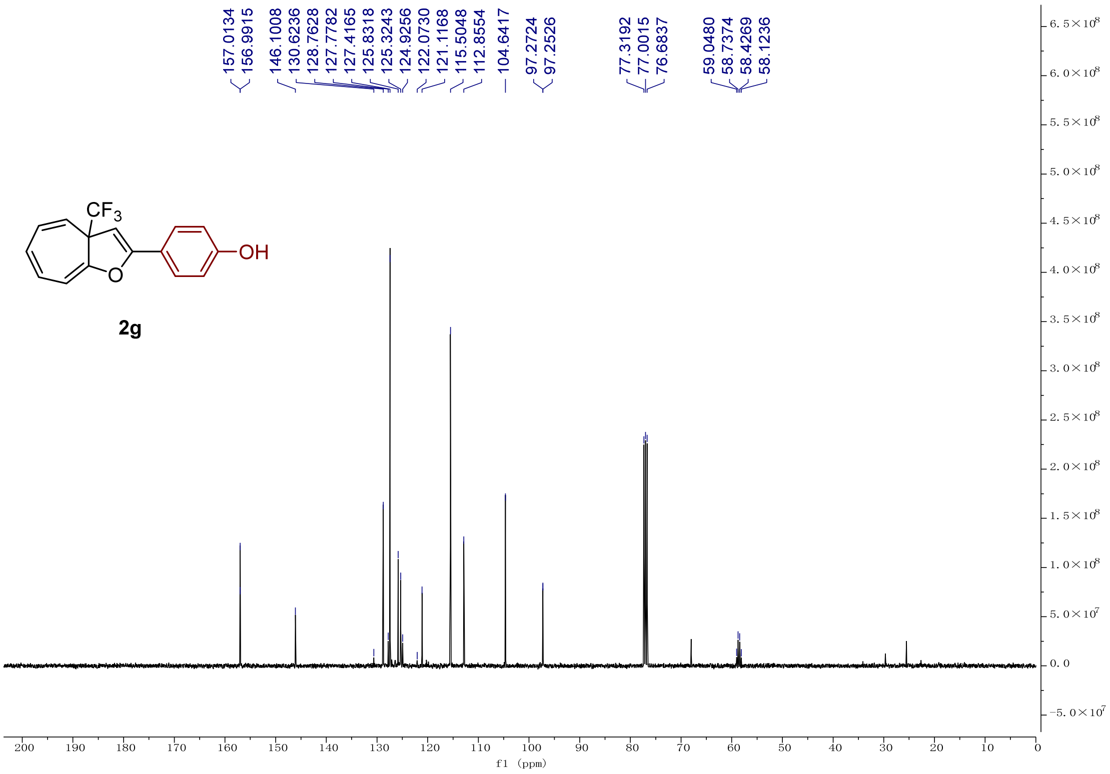

^{19}F NMR (376 MHz, $CDCl_3$) of compound **2g**

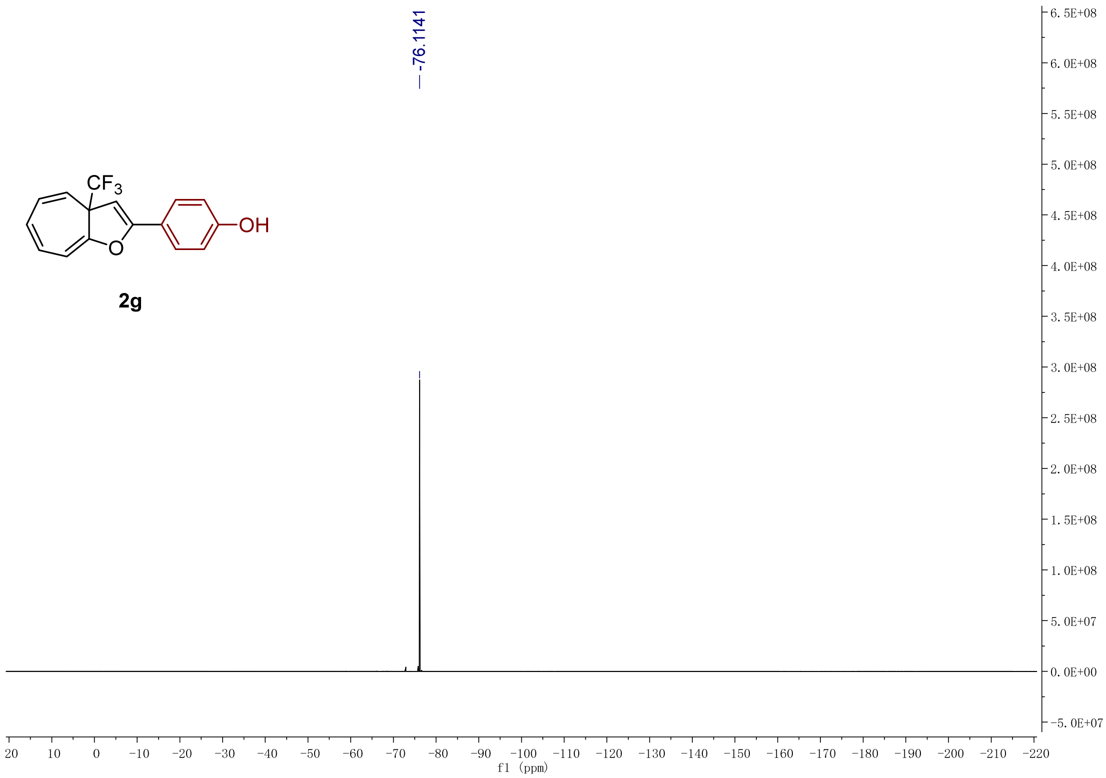

^{1}H NMR (600 MHz, $CDCl_3$) of compound **2h**

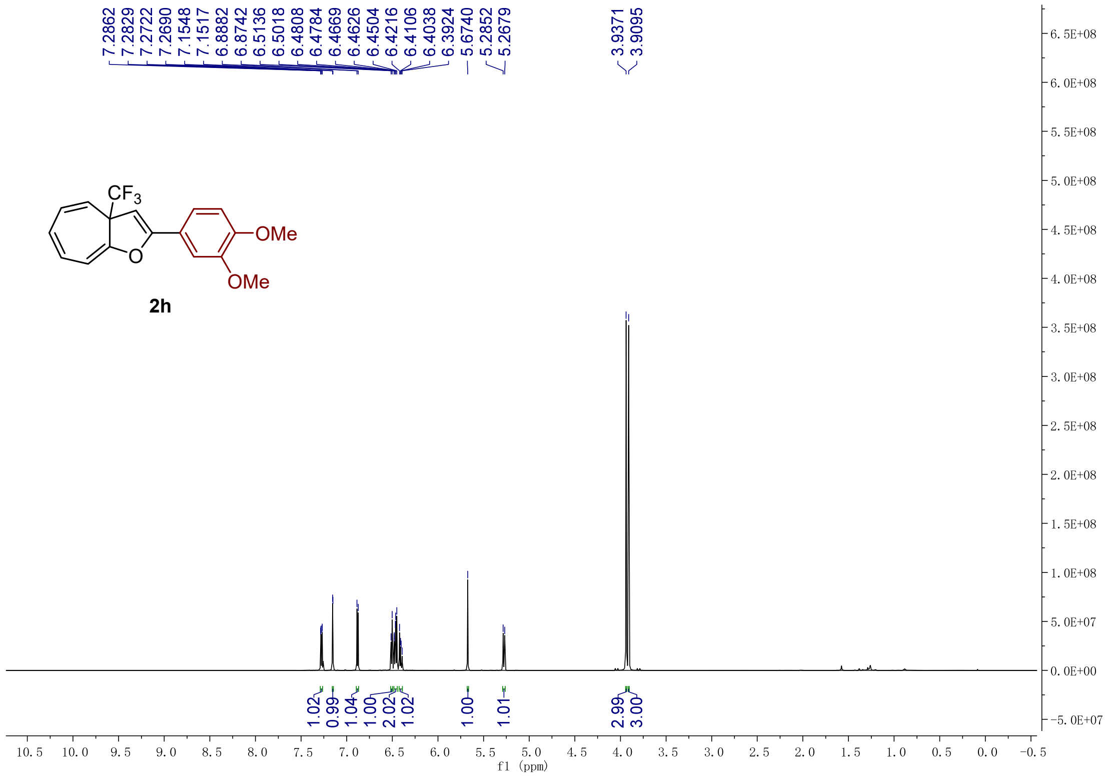

^{13}C NMR (151 MHz, $CDCl_3$) of compound **2h**

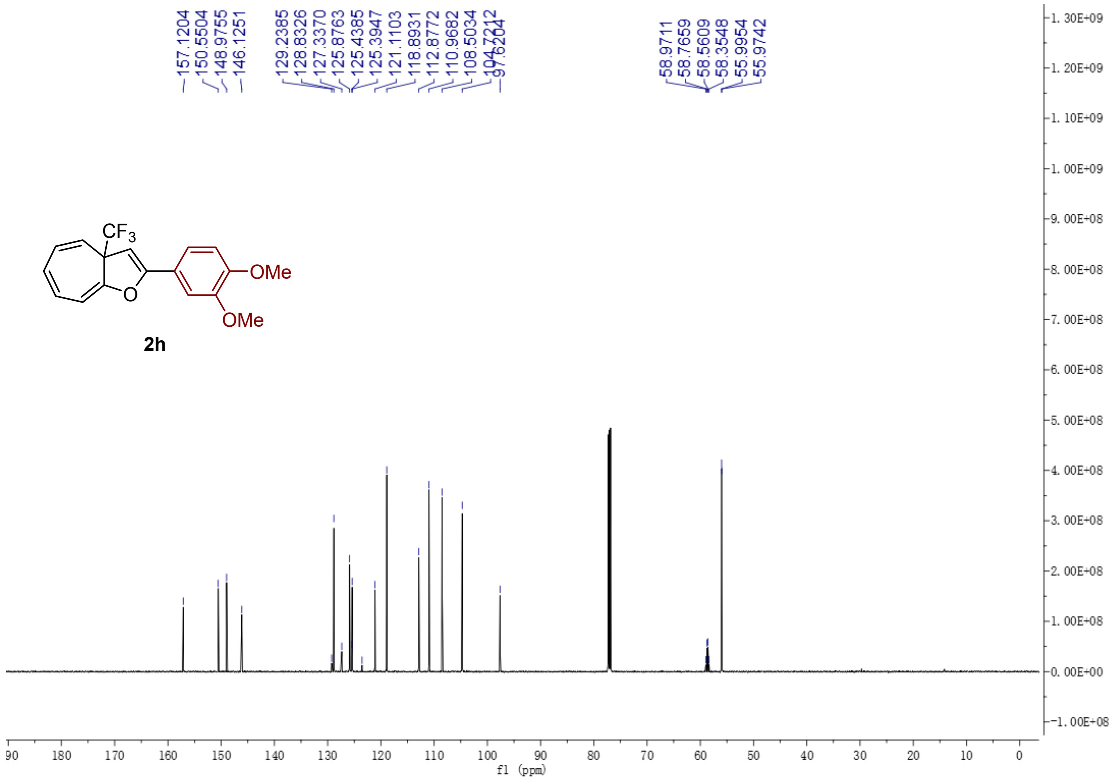

^{19}F NMR (565 MHz, $CDCl_3$) of compound **2h**

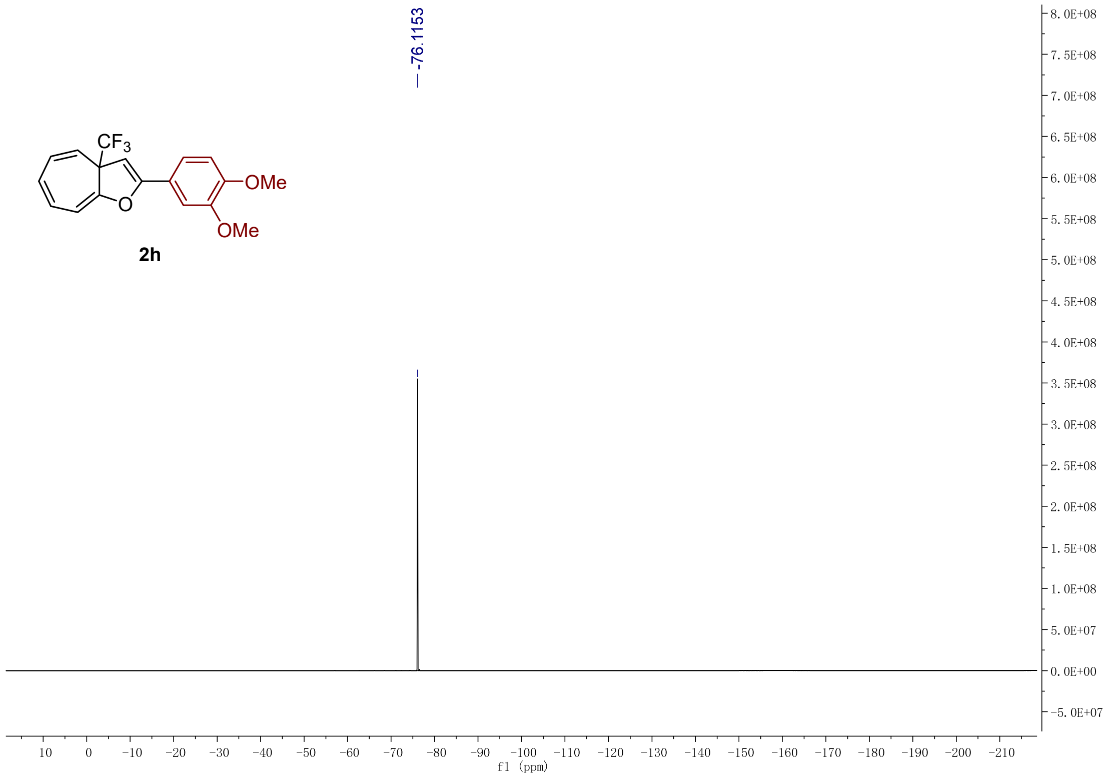

^{1}H NMR (600 MHz, CDCl$_3$) of compound **2i**

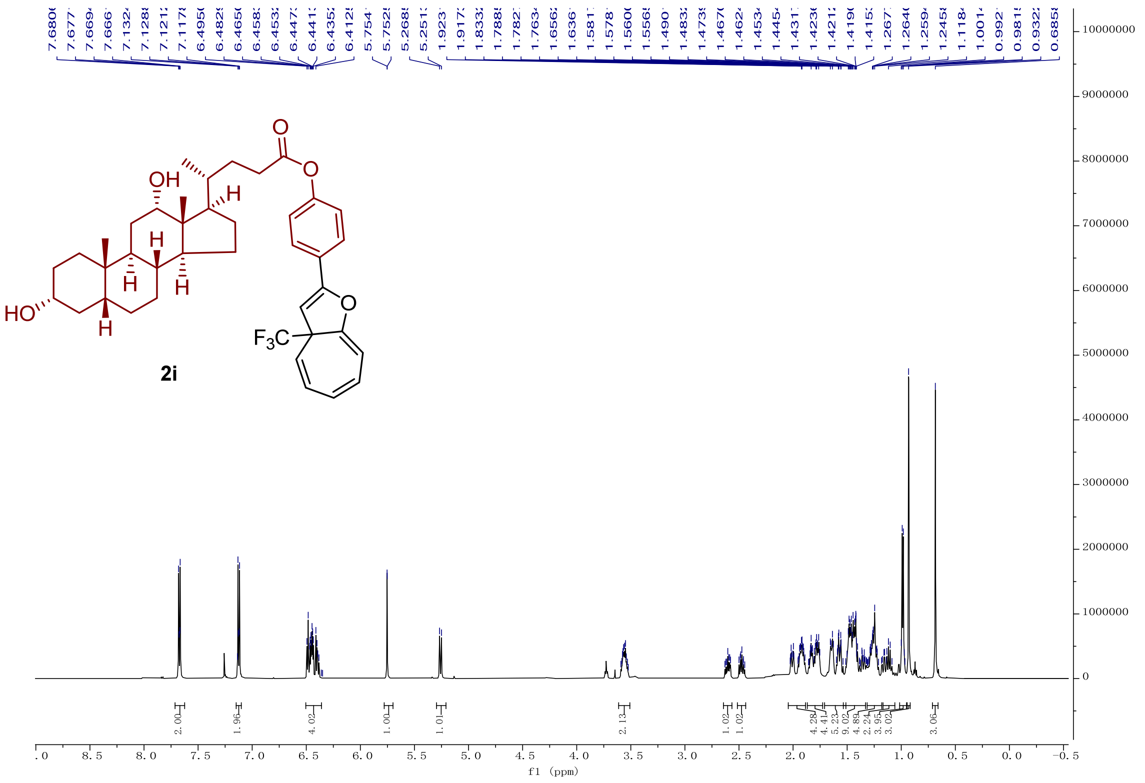

^{13}C NMR (151 MHz, CDCl$_3$) of compound **2i**

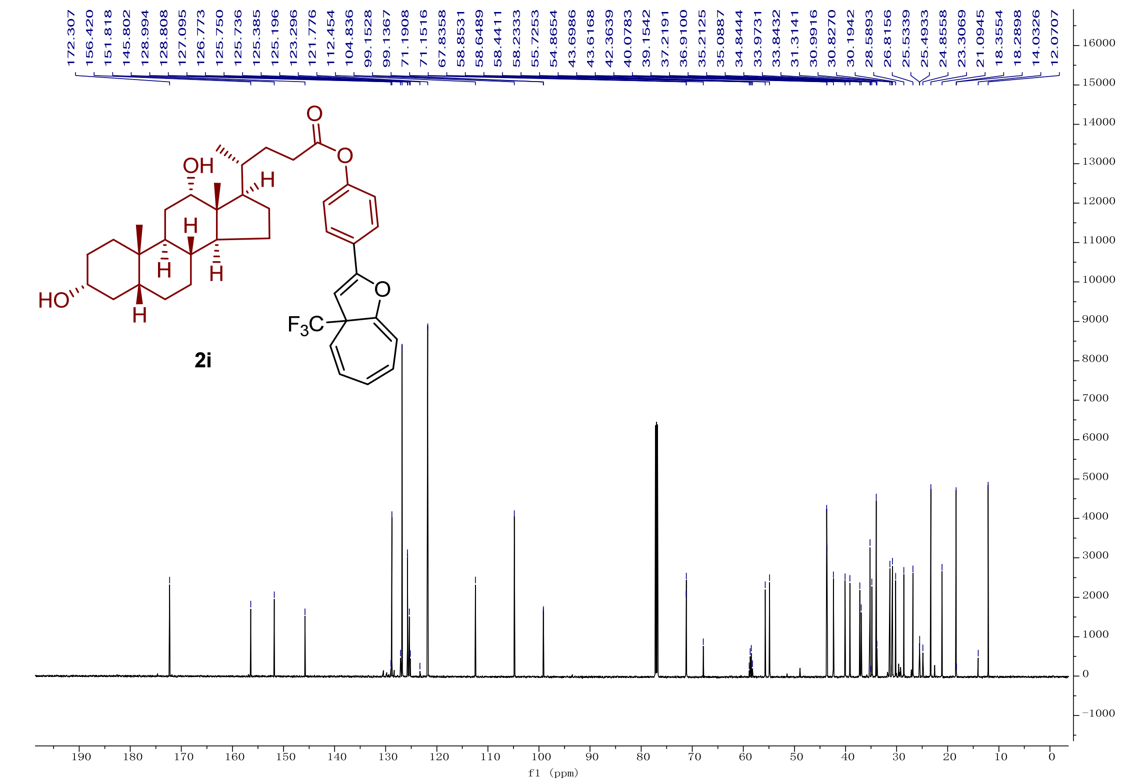

^{19}F NMR (565 MHz, $CDCl_3$) of compound **2i**

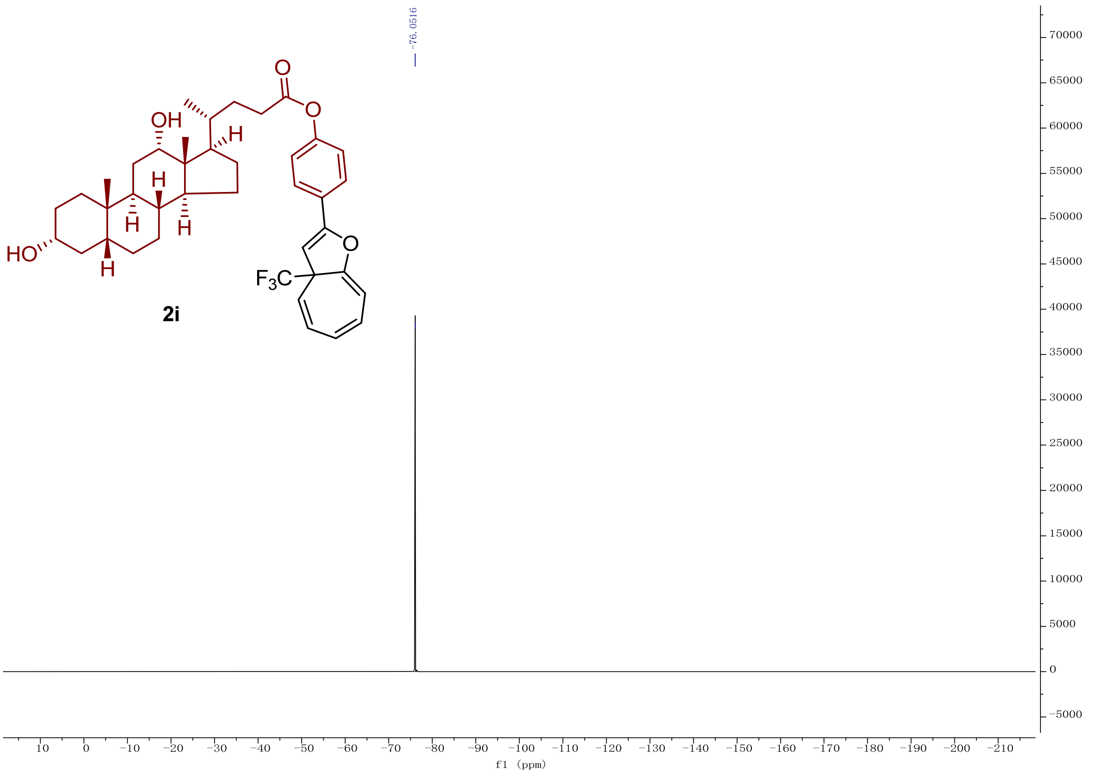

^{1}H NMR (400 MHz, $CDCl_3$) of compound **2j**

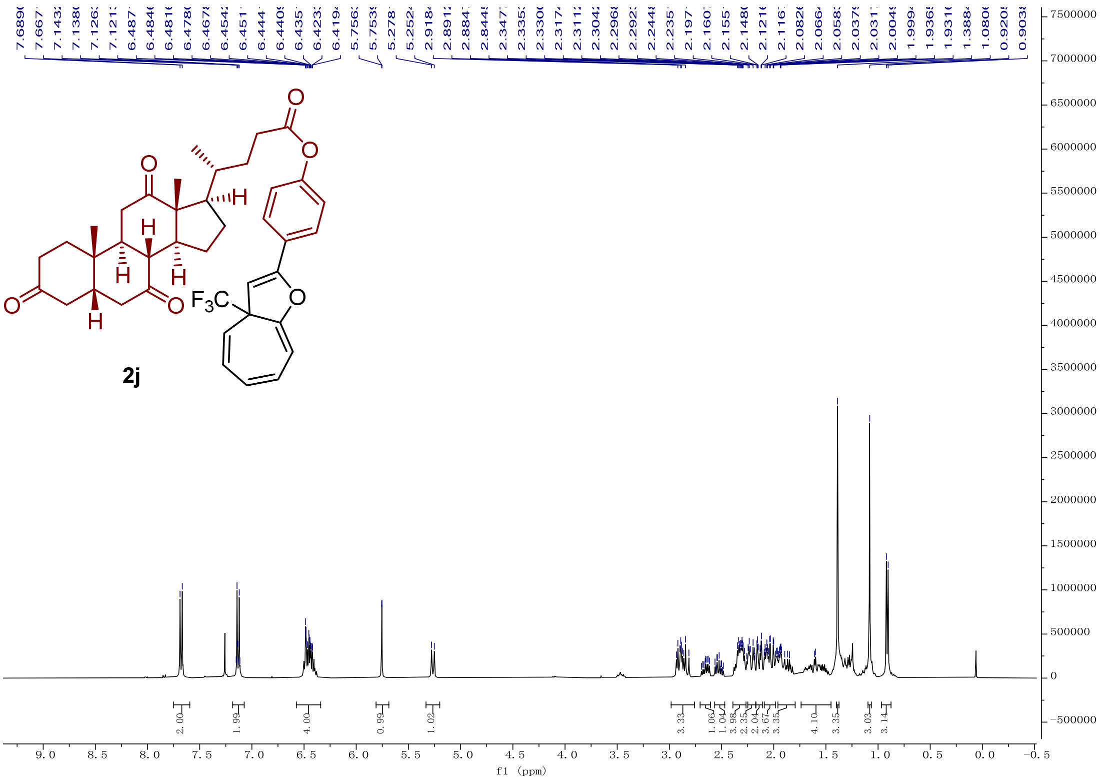

^{13}C NMR (101 MHz, $CDCl_3$) of compound **2j**

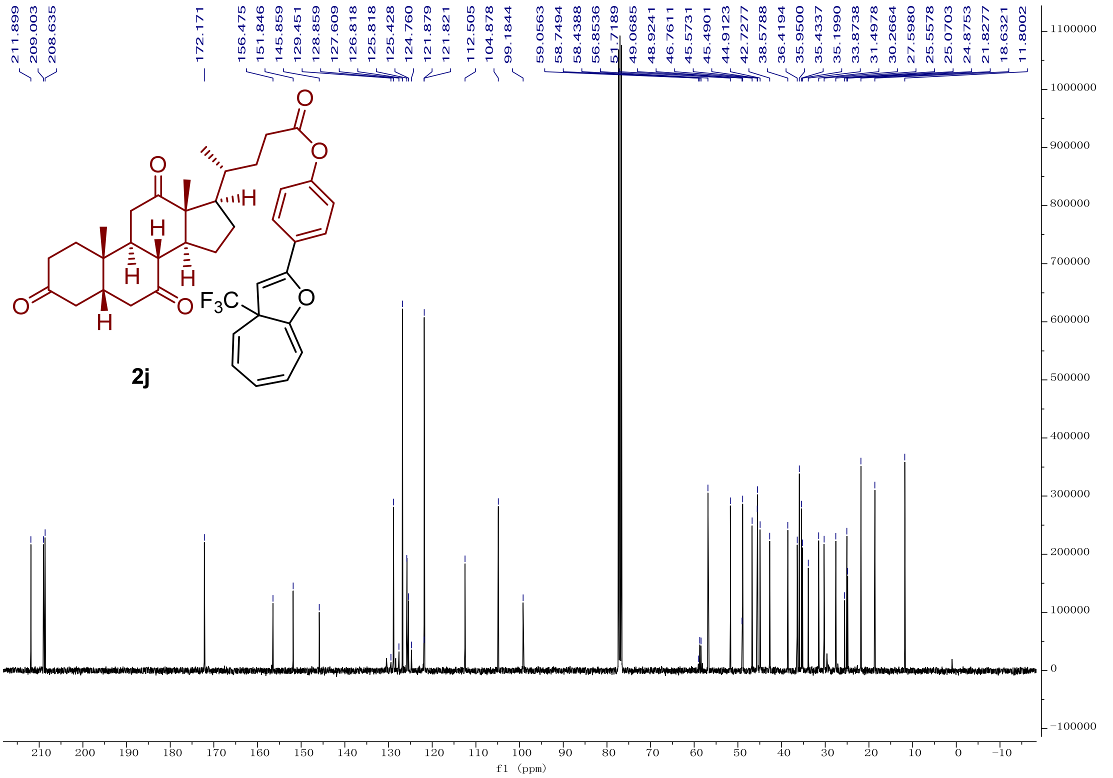

^{19}F NMR (376 MHz, $CDCl_3$) of compound **2j**

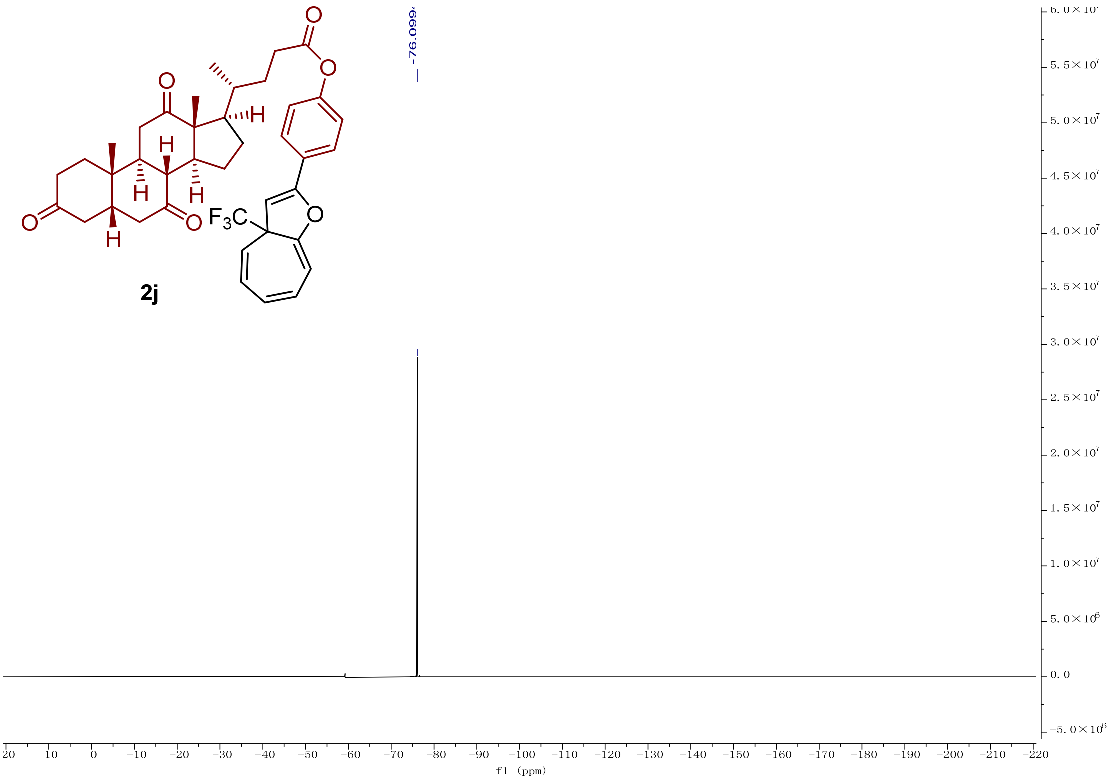

^{1}H NMR (400 MHz, $CDCl_3$) of compound **2k**

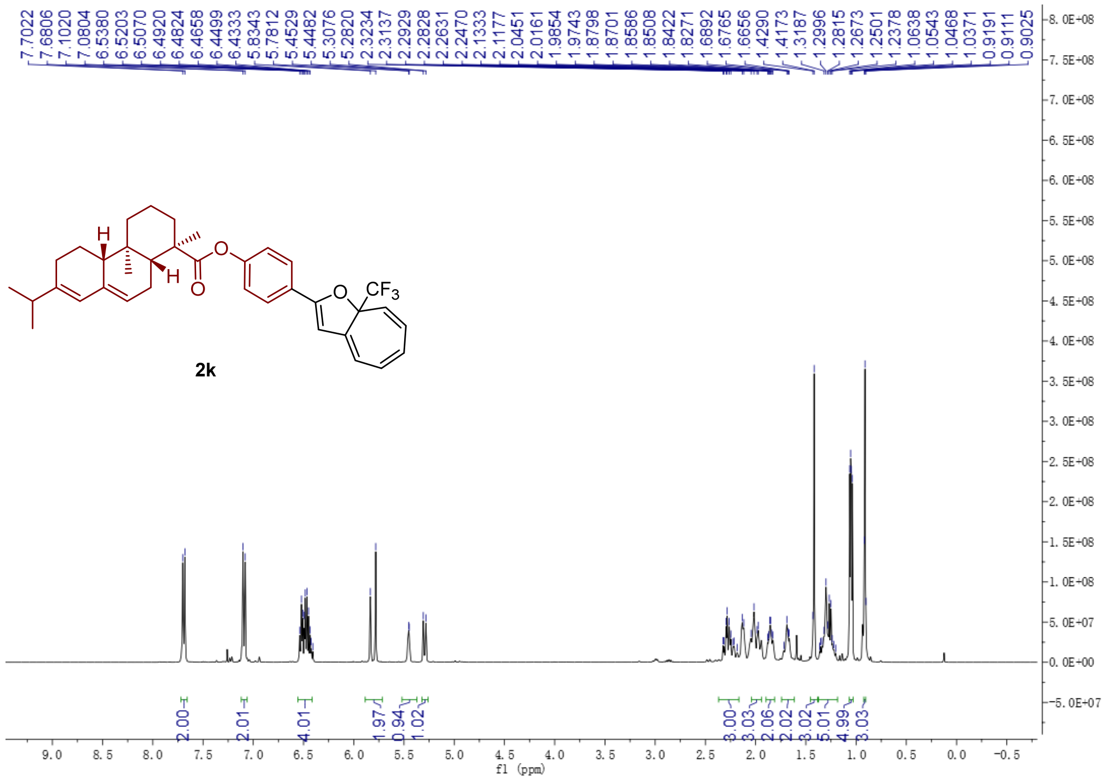

^{13}C NMR (101 MHz, $CDCl_3$) of compound **2k**

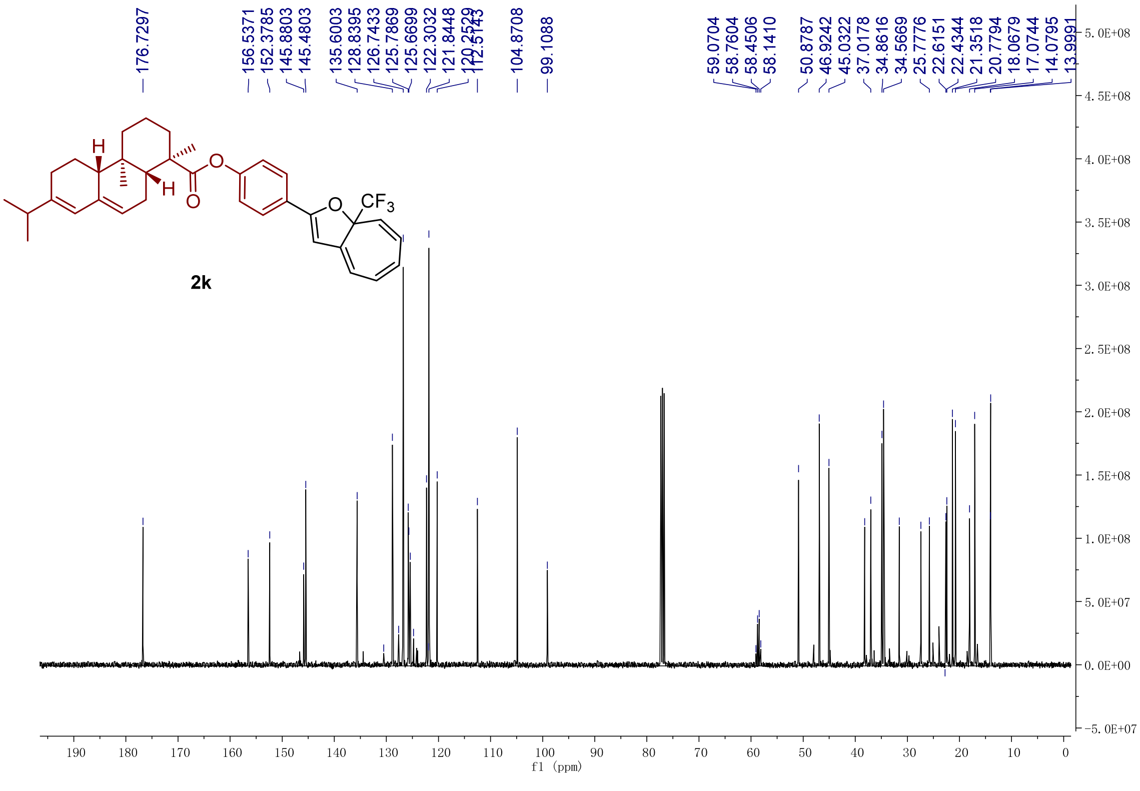

^{19}F NMR (376 MHz, $CDCl_3$) of compound **2k**

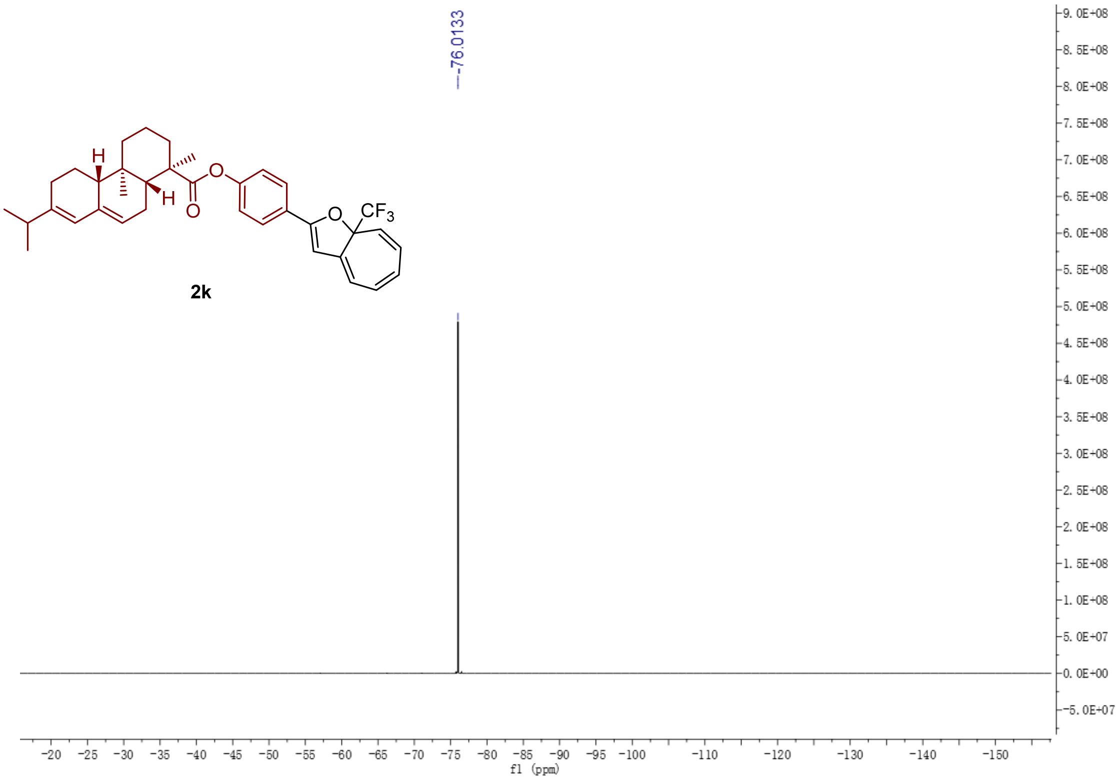

^{1}H NMR (400 MHz, $CDCl_3$) of compound **2l**

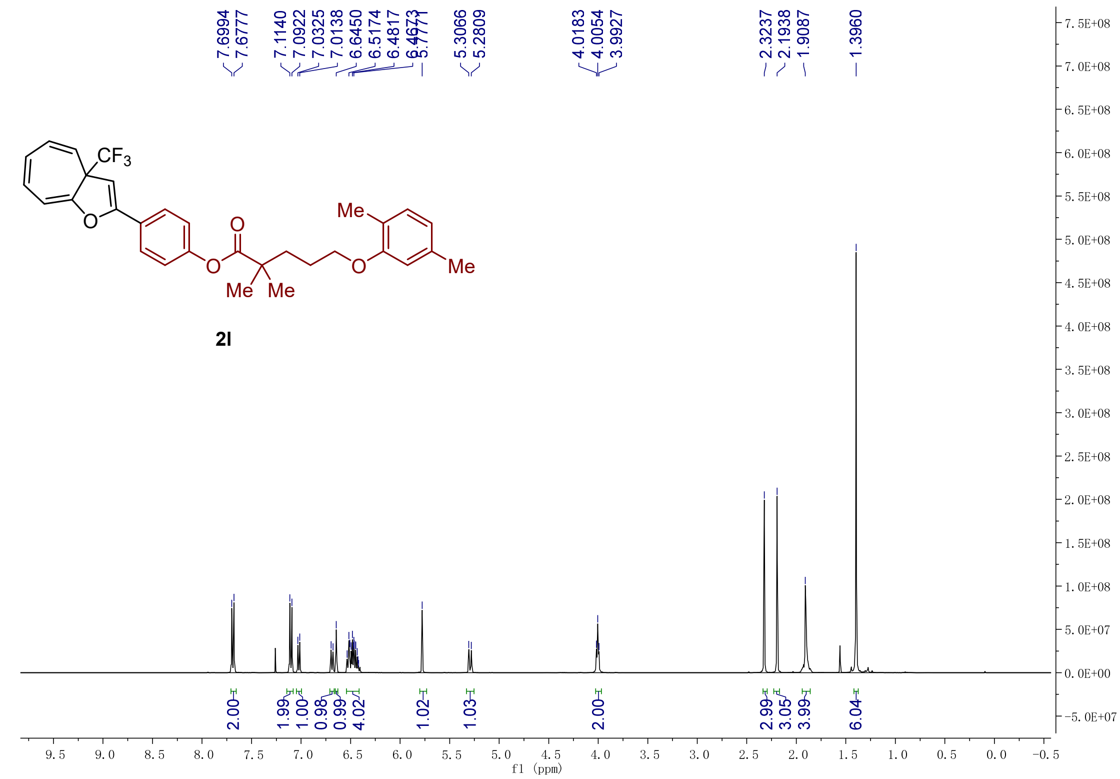

^{13}C NMR (101 MHz, $CDCl_3$) of compound **2l**

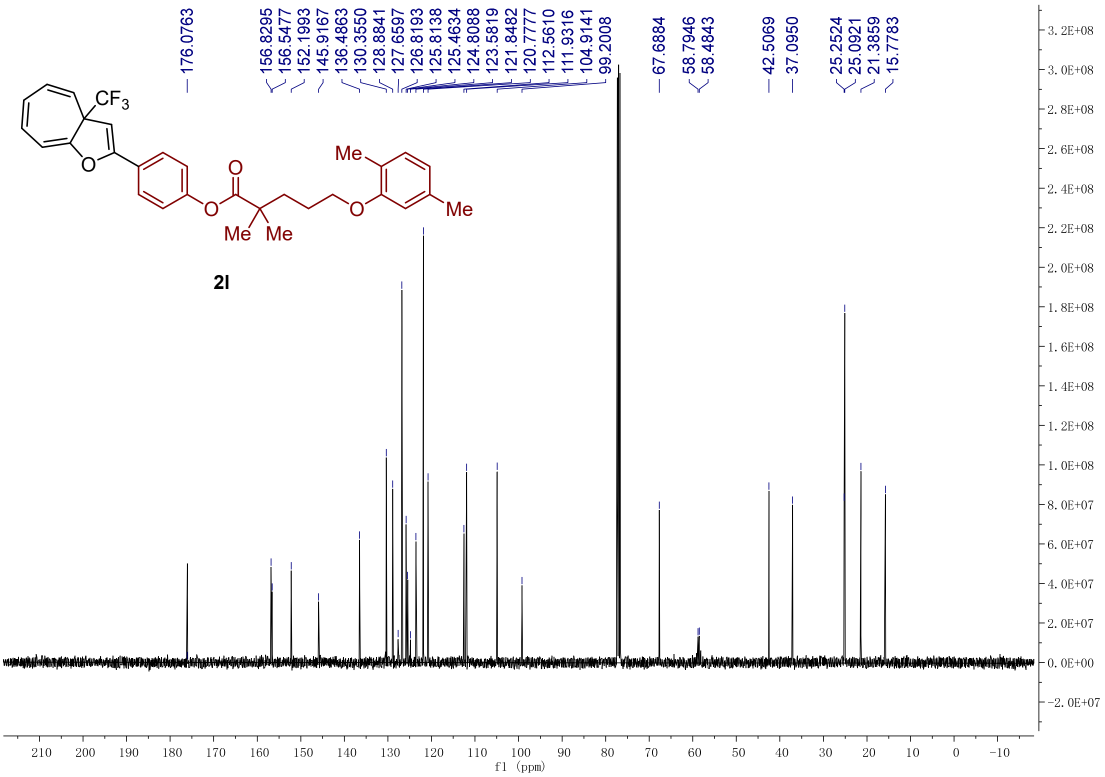

^{19}F NMR (376 MHz, $CDCl_3$) of compound **2l**

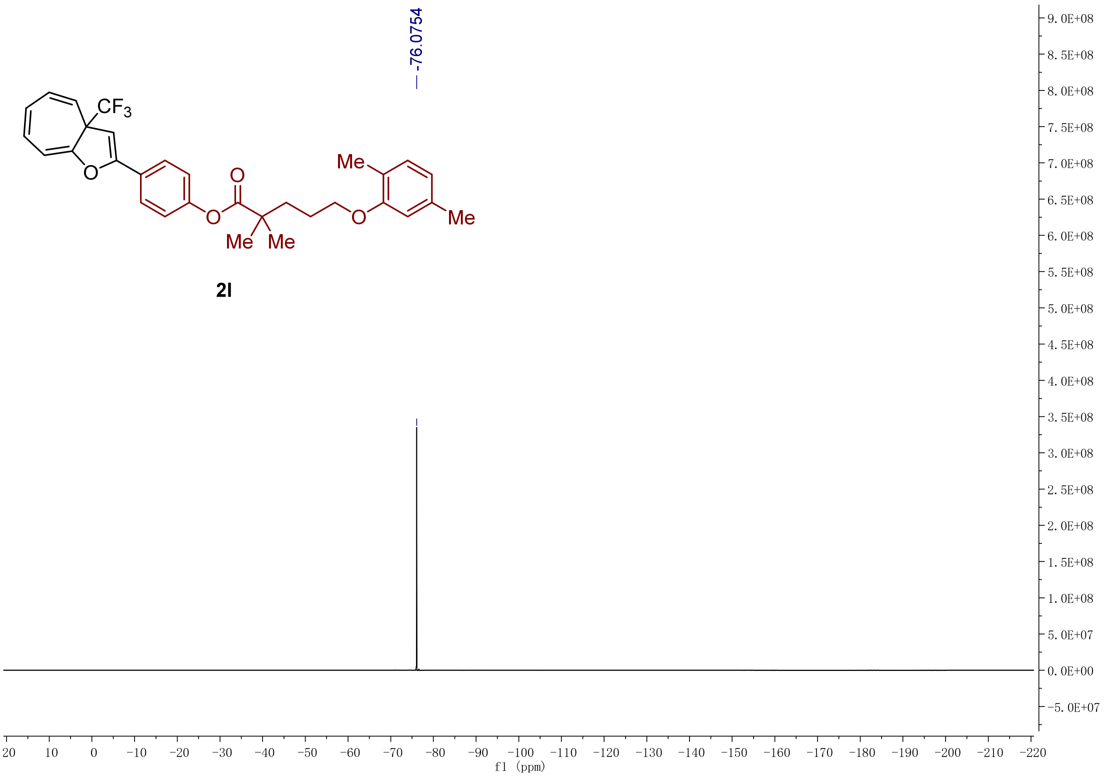

^{1}H NMR (600 MHz, $CDCl_3$) of compound **2m**

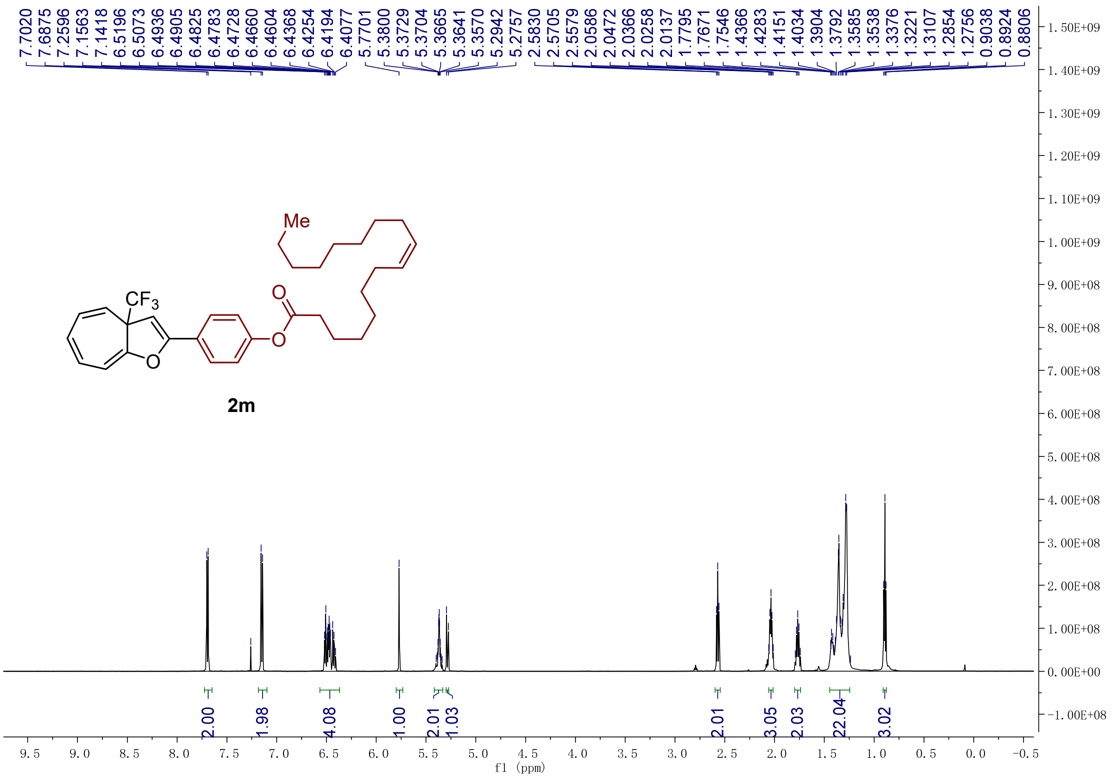

^{13}C NMR (151 MHz, $CDCl_3$) of compound 2m

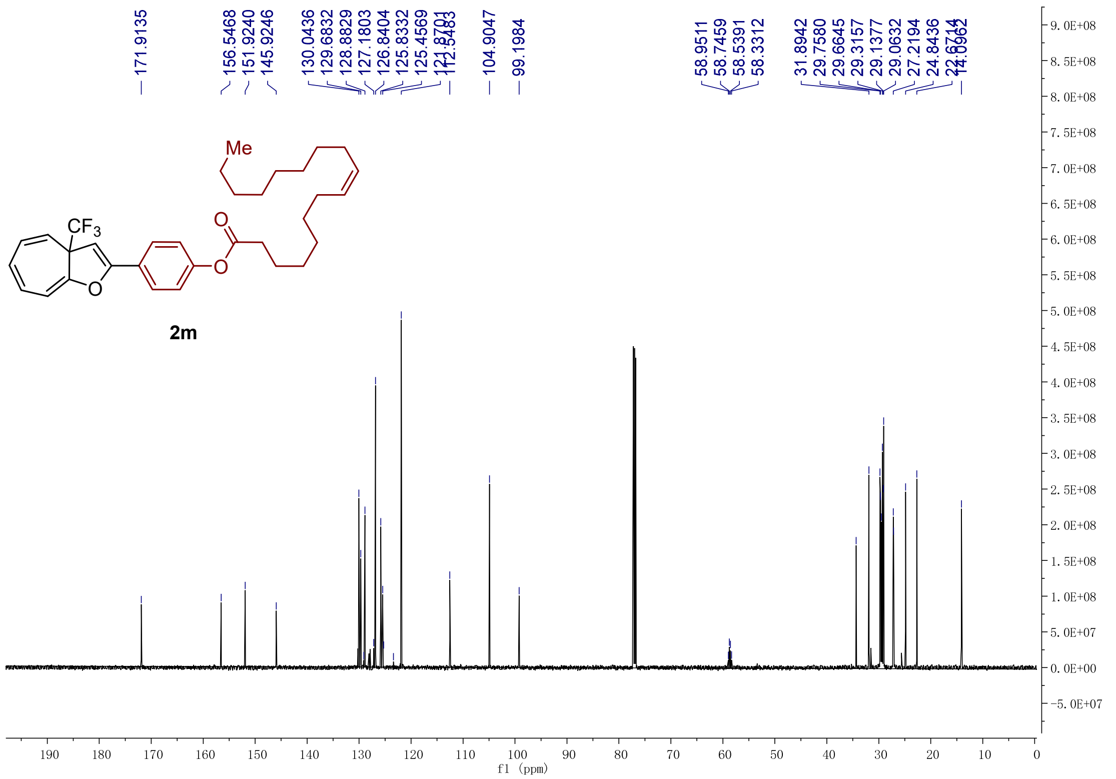

^{19}F NMR (565 MHz, $CDCl_3$) of compound **2m**

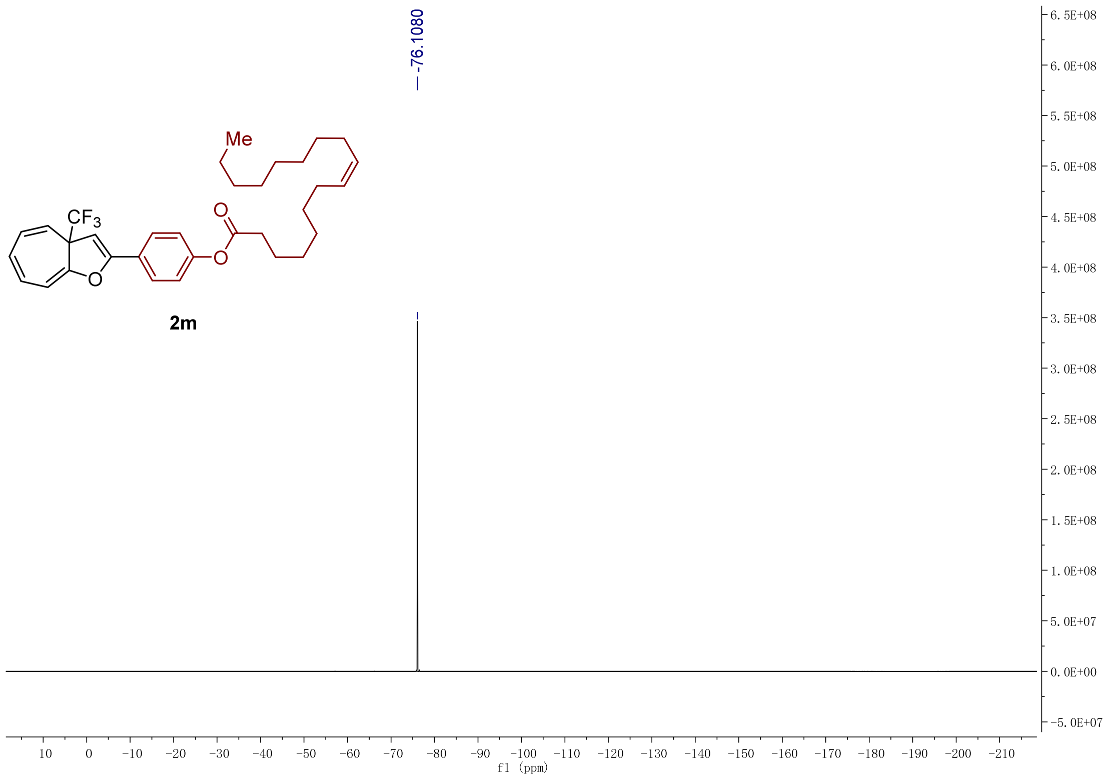

^{1}H NMR (300 MHz, $CDCl_3$) of compound **2n**

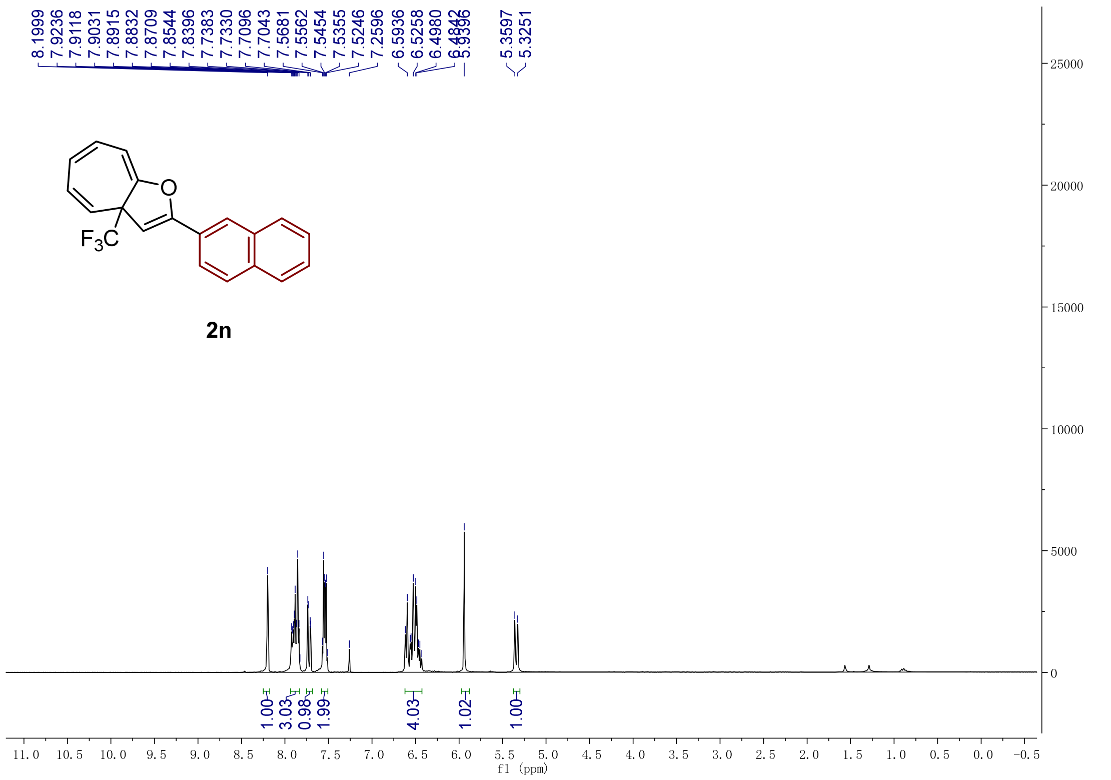

^{13}C NMR (75 MHz, $CDCl_3$) of compound **2n**

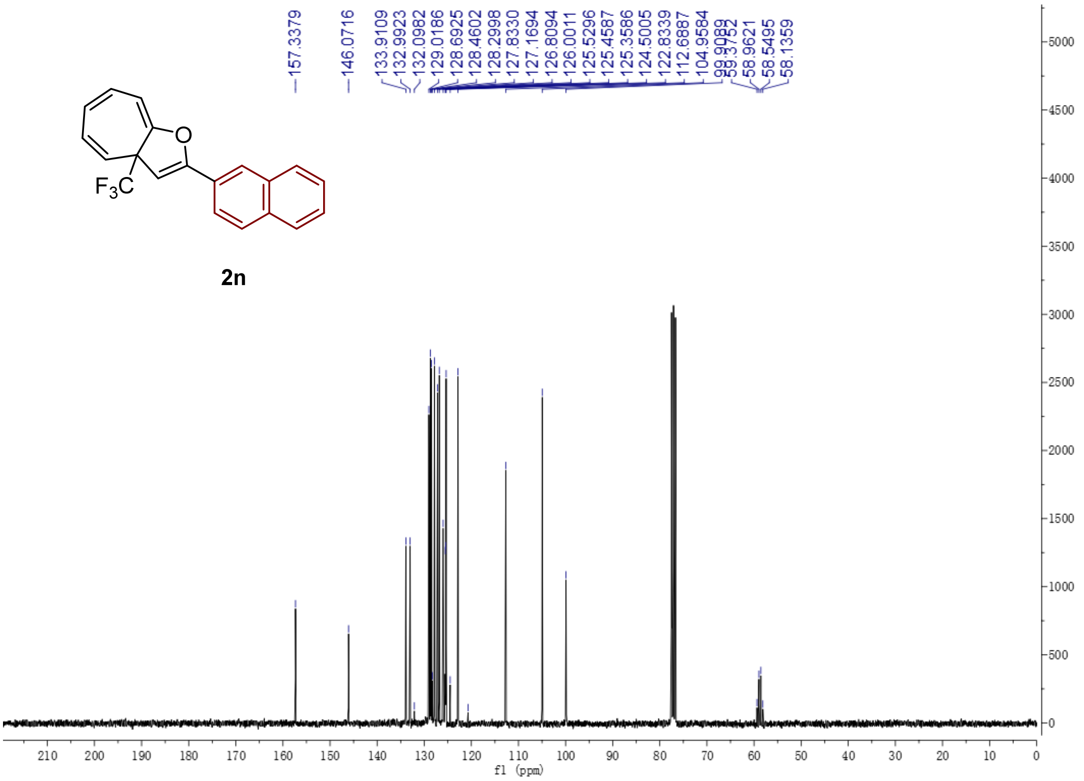

^{19}F NMR (565 MHz, $CDCl_3$) δ -76.0 of compound **2n**

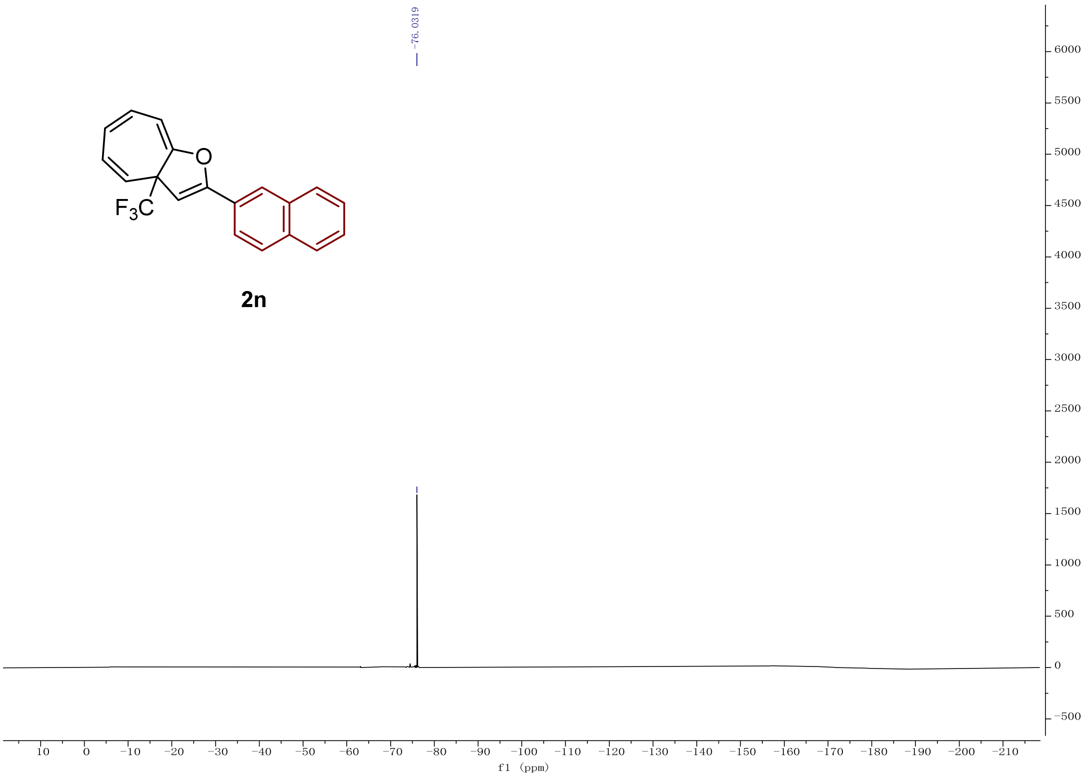

^{1}H NMR (600 MHz, $CDCl_3$) of compound **2o**

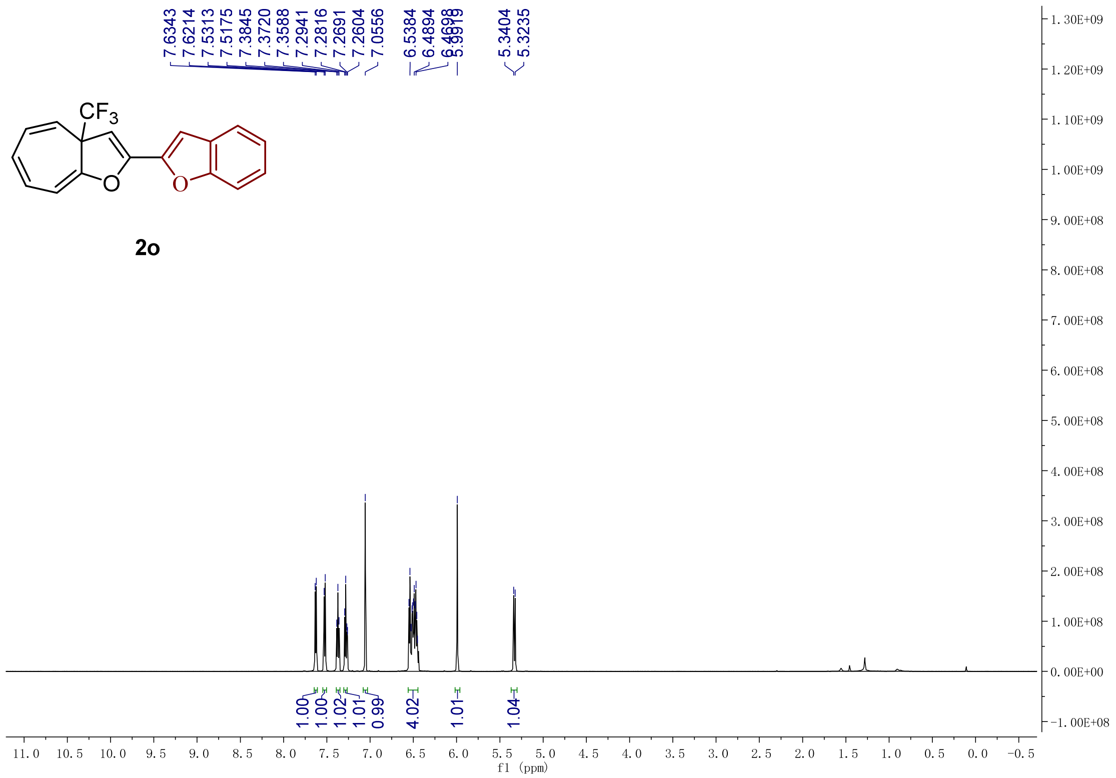

^{13}C NMR (151 MHz, $CDCl_3$) of compound **2o**

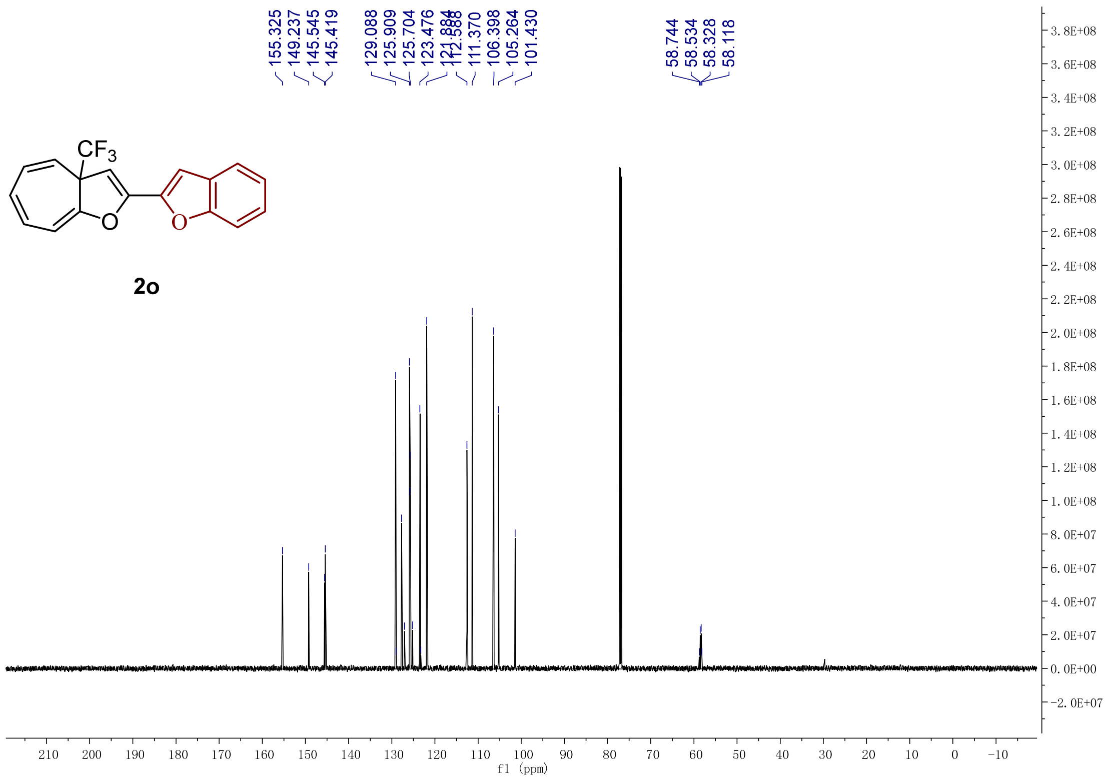

^{19}F NMR (565 MHz, $CDCl_3$) of compound **2o**

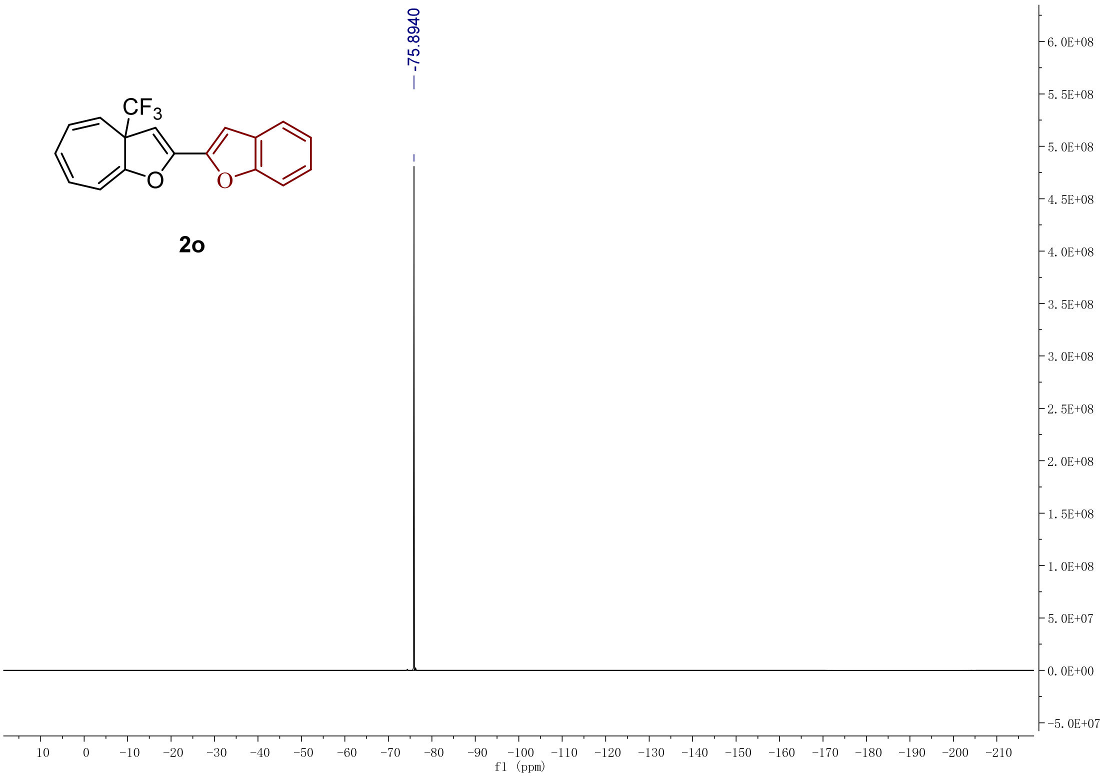

^{1}H NMR (400 MHz, $CDCl_3$) of compound **2p**

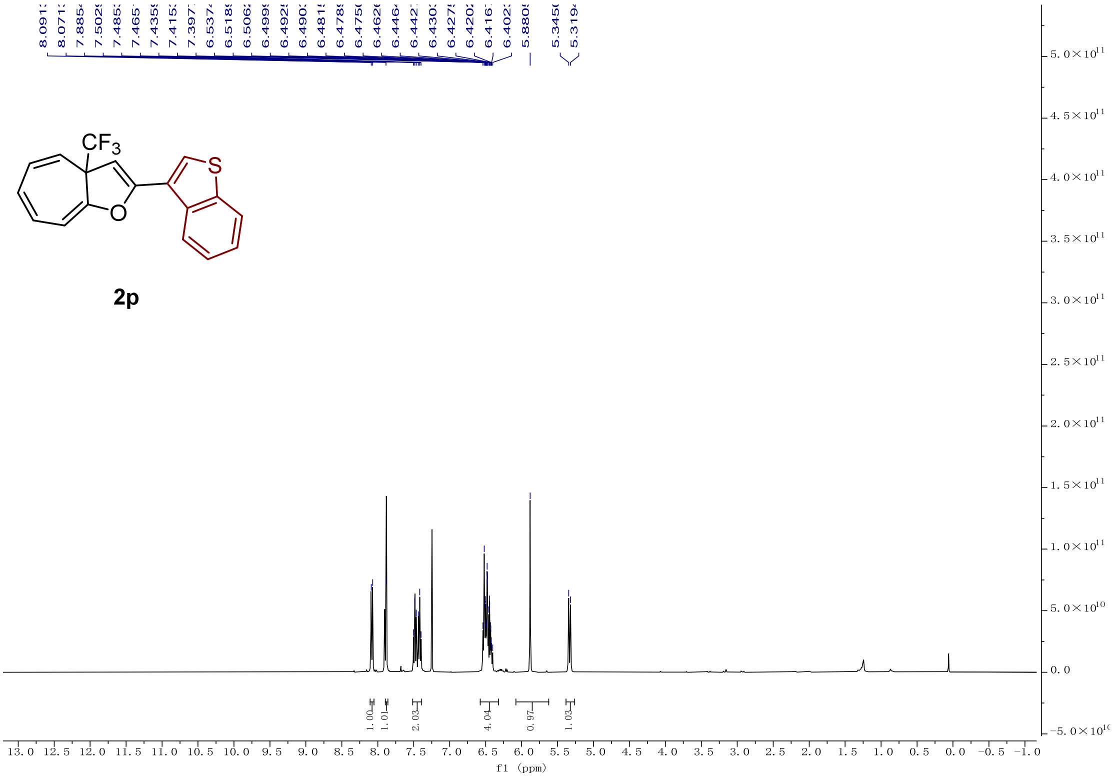

^{13}C NMR (151 MHz, $CDCl_3$) of compound **2p**

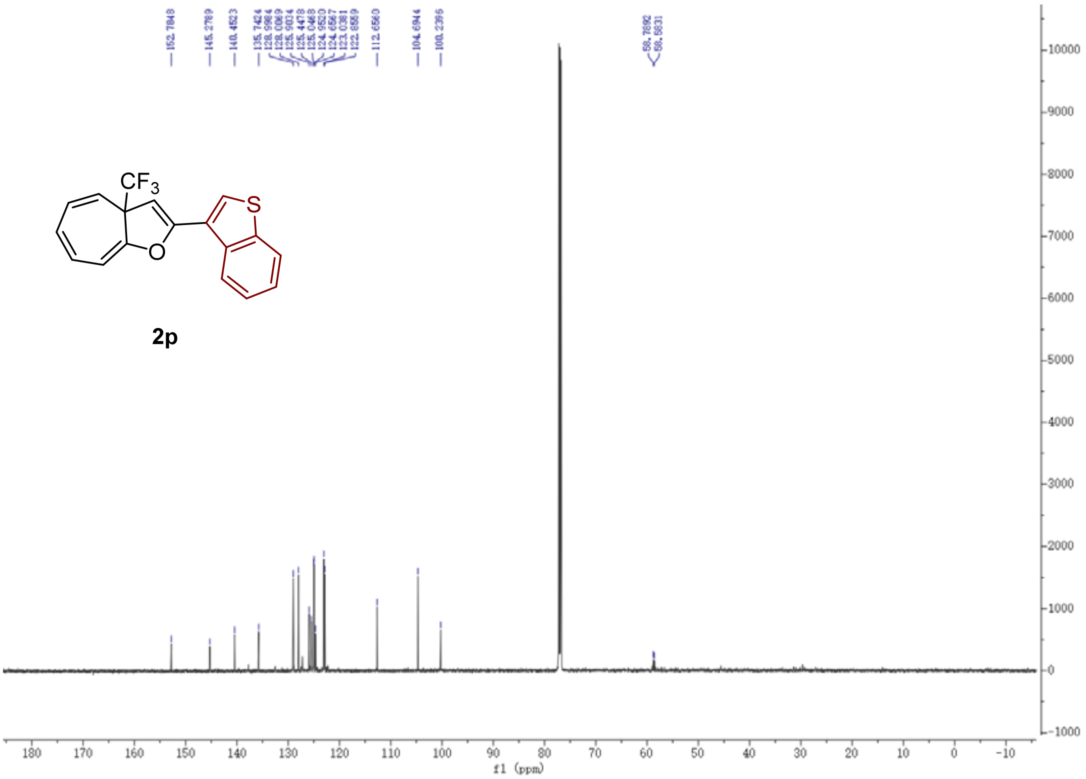

^{19}F NMR (565 MHz, $CDCl_3$) of compound **2p**

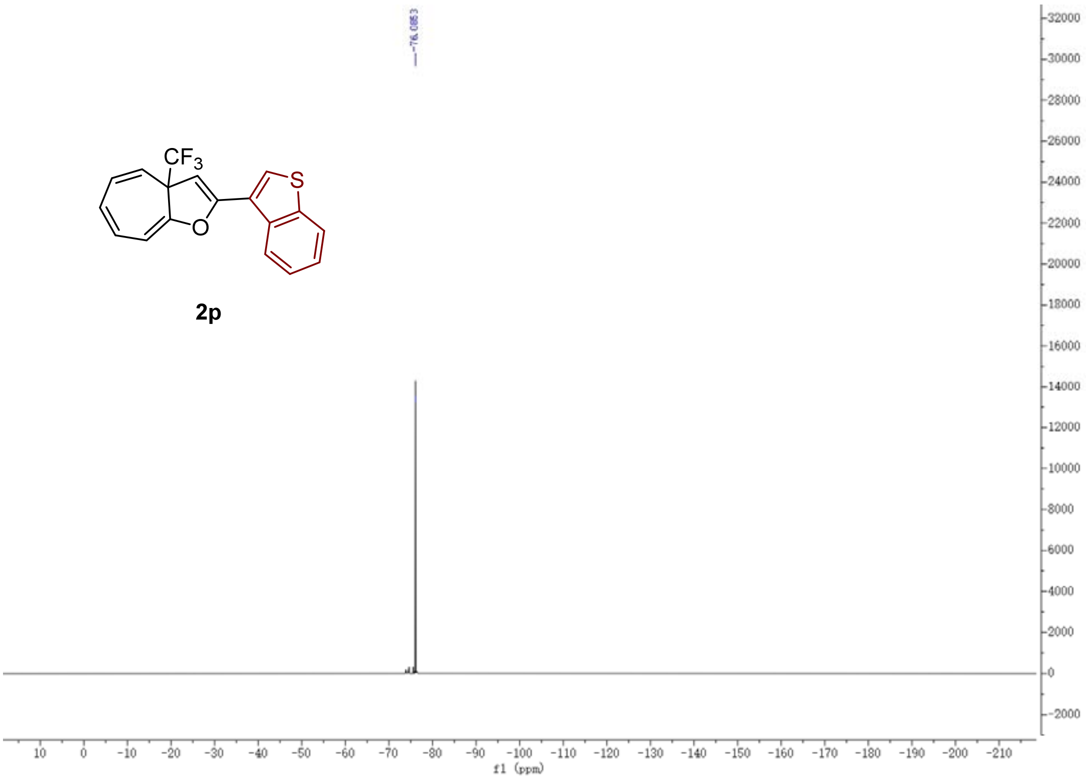

^{1}H NMR (600 MHz, $CDCl_3$) of compound **2q**

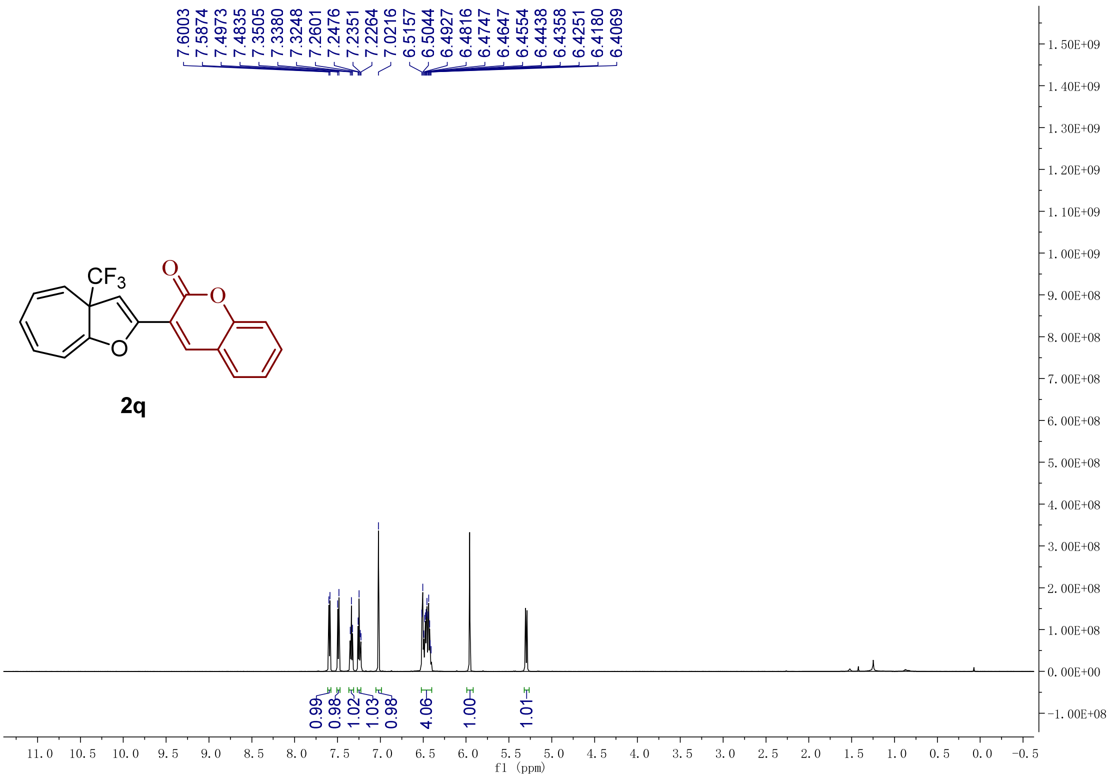

^{13}C NMR (151 MHz, $CDCl_3$) of compound of **2q**

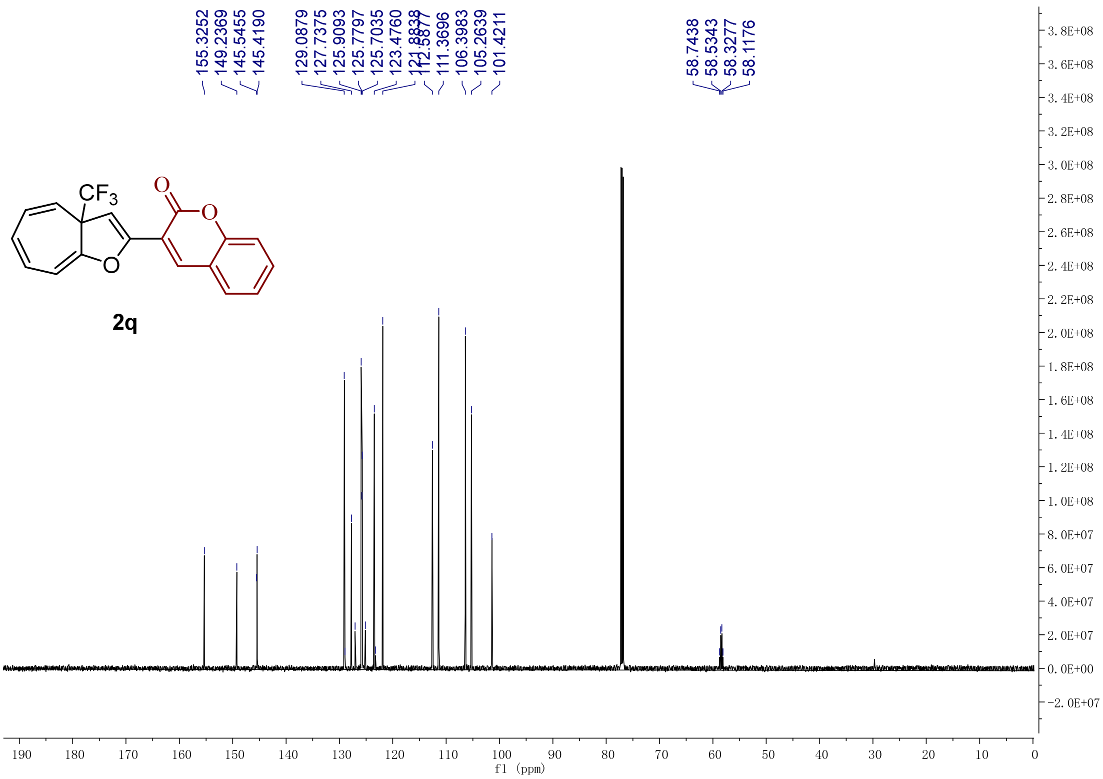

^{19}F NMR (565 MHz, $CDCl_3$) of compound **2q**

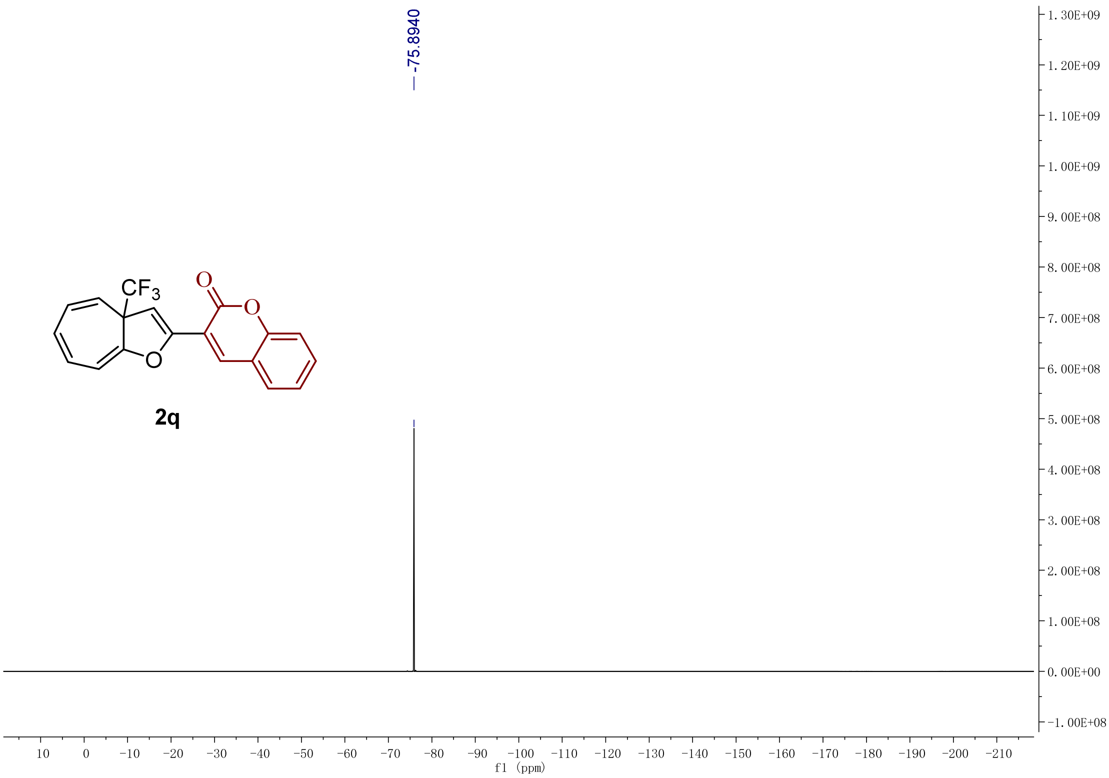

^{1}H NMR (400 MHz, $CDCl_3$) of compound **2r**

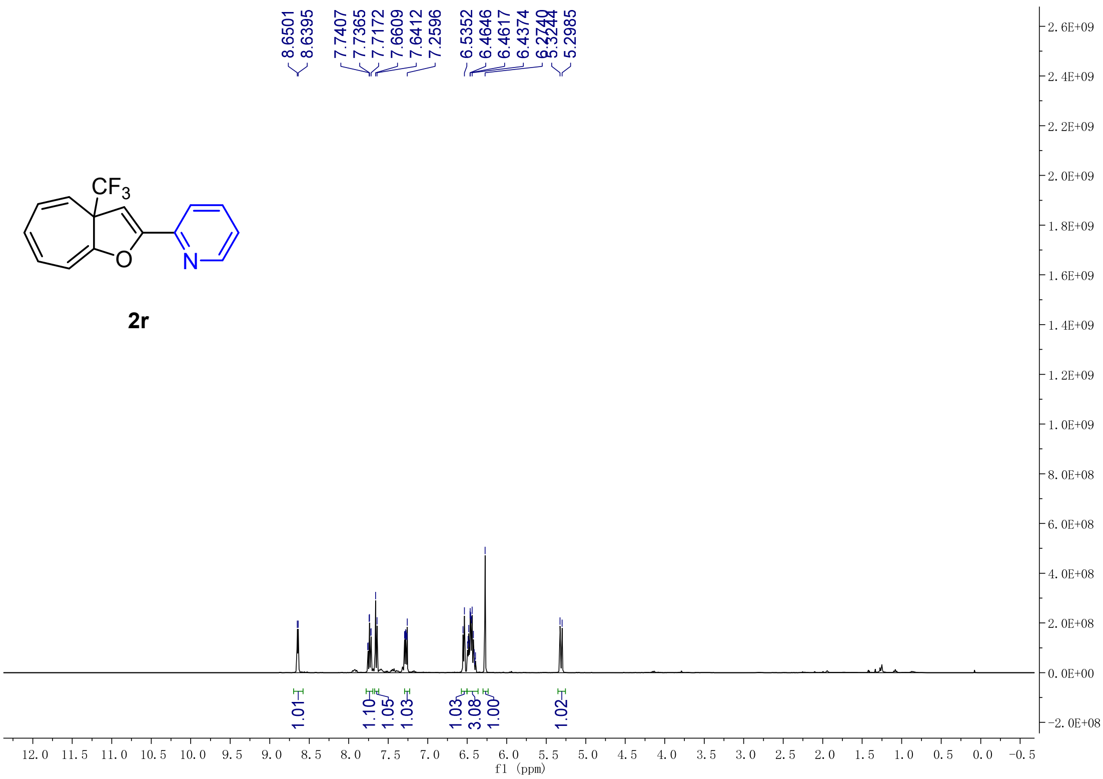

^{13}C NMR (101 MHz, $CDCl_3$) of compound **2r**

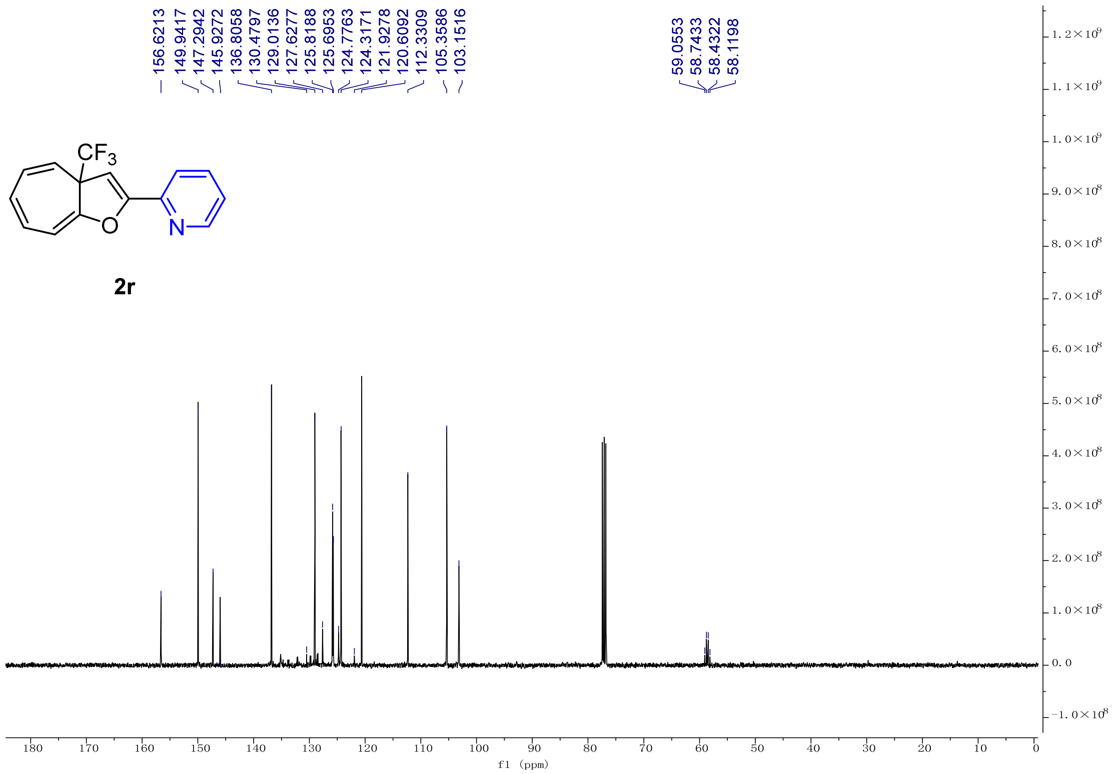

^{19}F NMR (376 MHz, $CDCl_3$) of compound **2r**

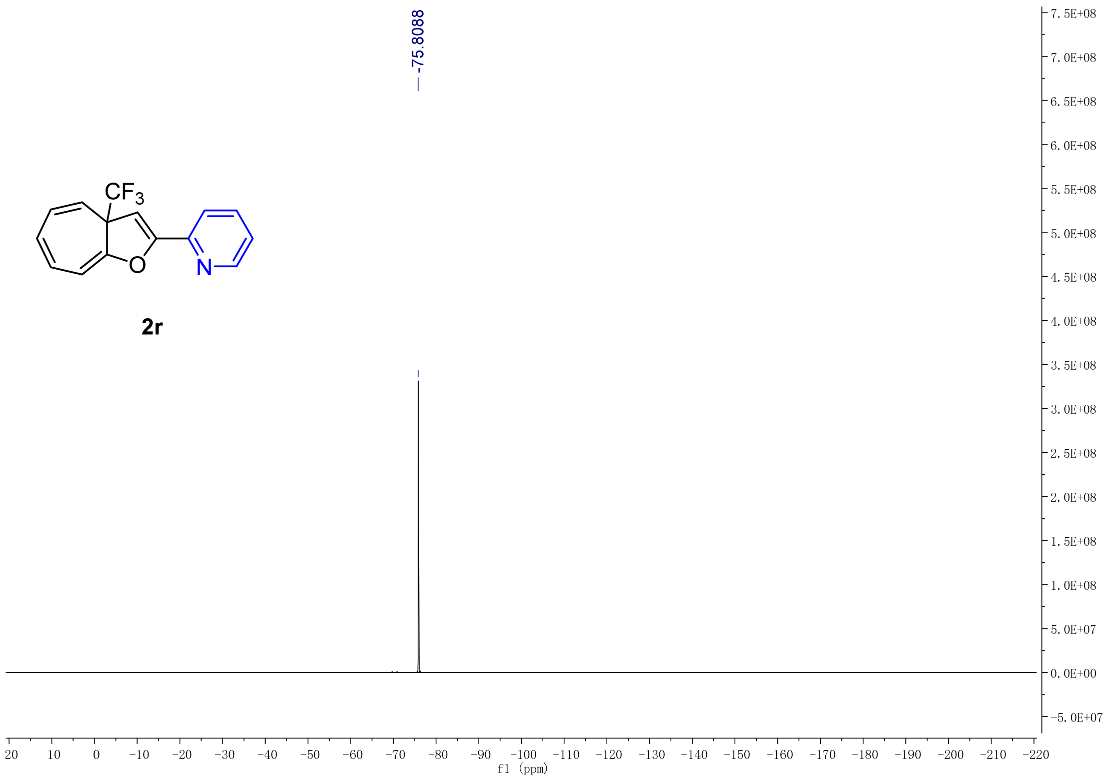

^{1}H NMR (600 MHz, $CDCl_2CDCl_2$) of compound **2s**

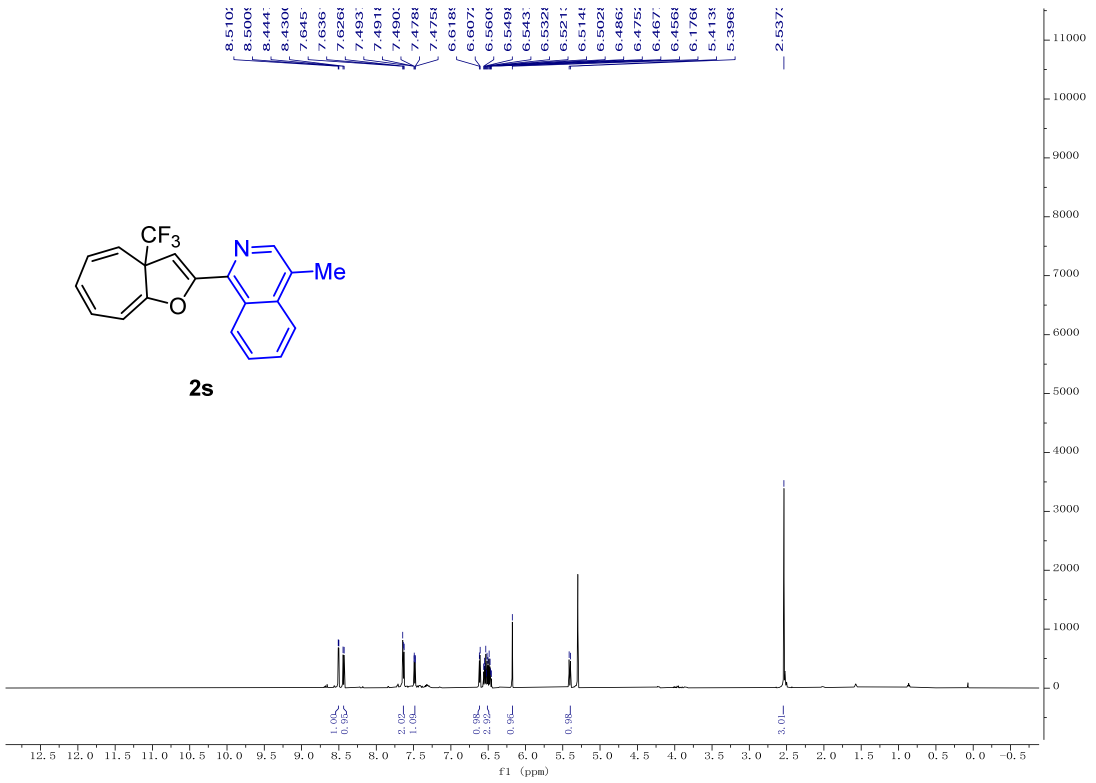

^{13}C NMR (151 MHz, $CDCl_2CDCl_2$) of compound **2s**

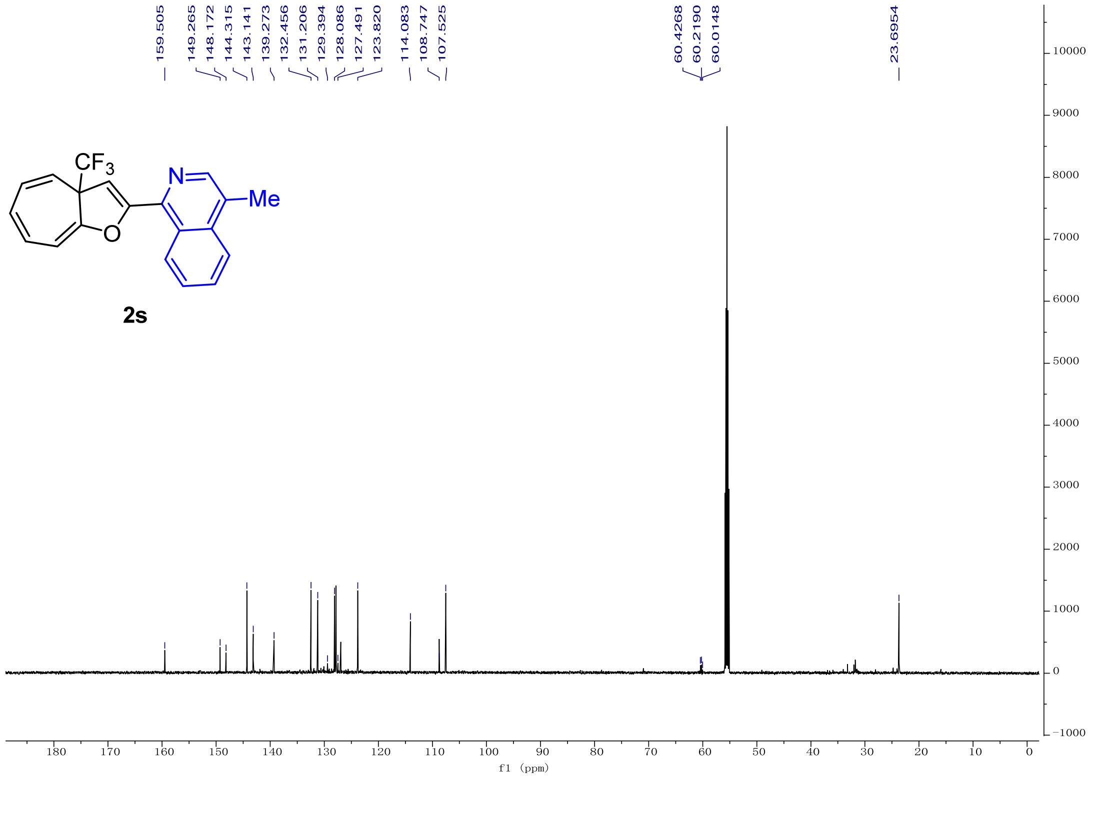

^{19}F NMR (565 MHz, $CDCl_2CDCl_2$) of compound **2s**

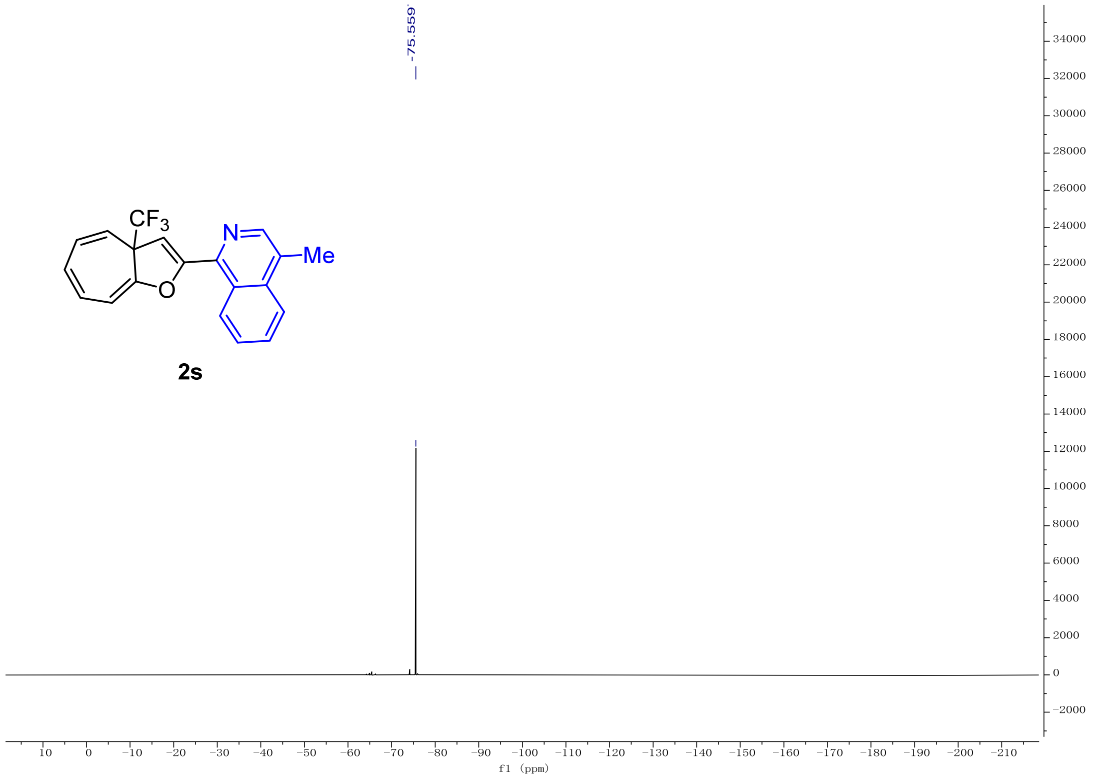

^{1}H NMR (300 MHz, $CDCl_3$) of compound **2t**

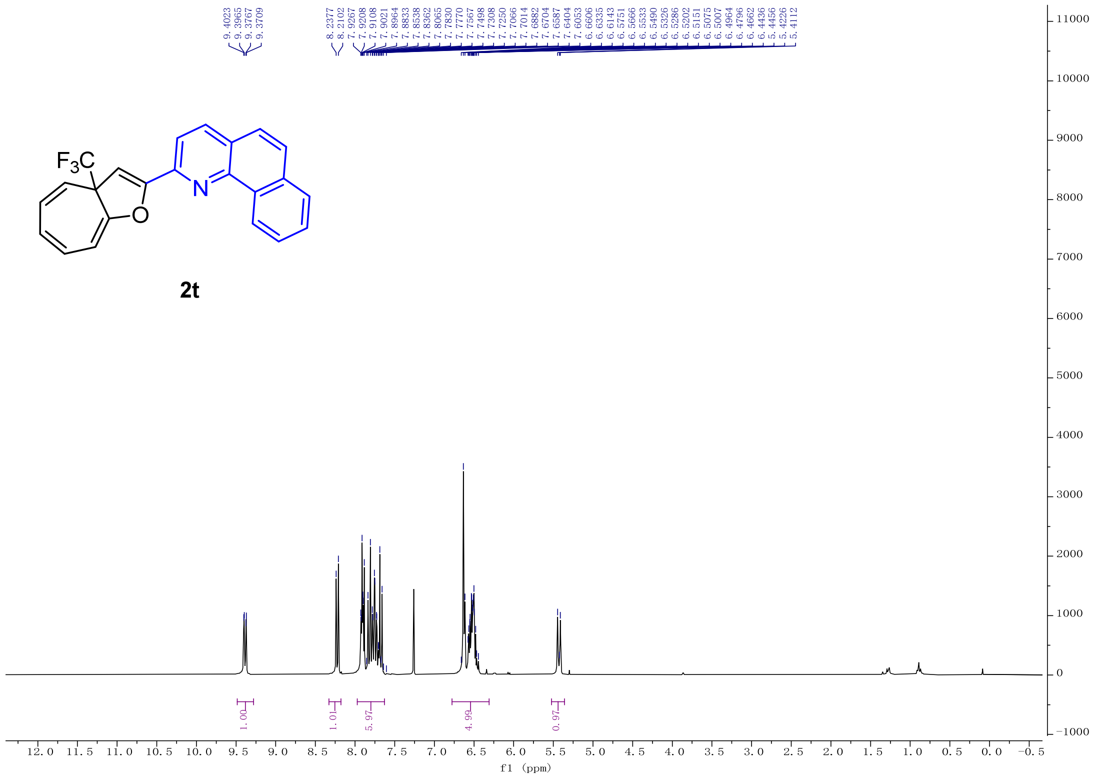

^{13}C NMR (151 MHz, CD_2Cl_2) of compound **2t**

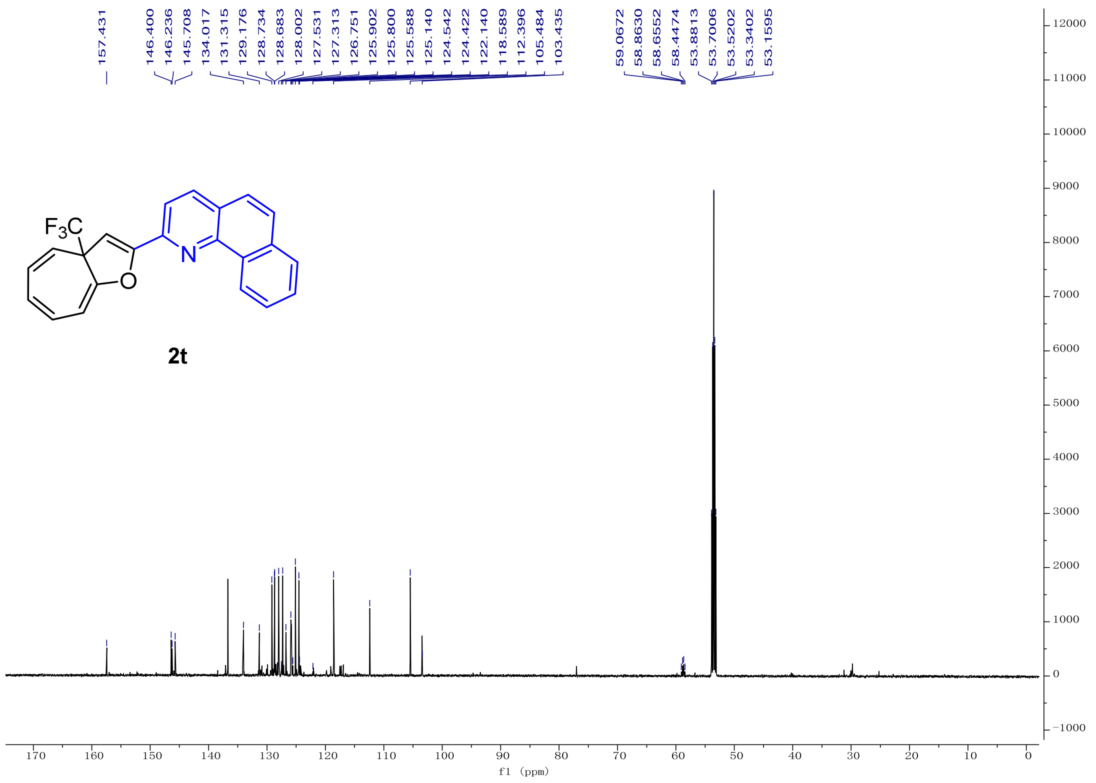

^{19}F NMR (565 MHz, CD_2Cl_2) of compound **2t**

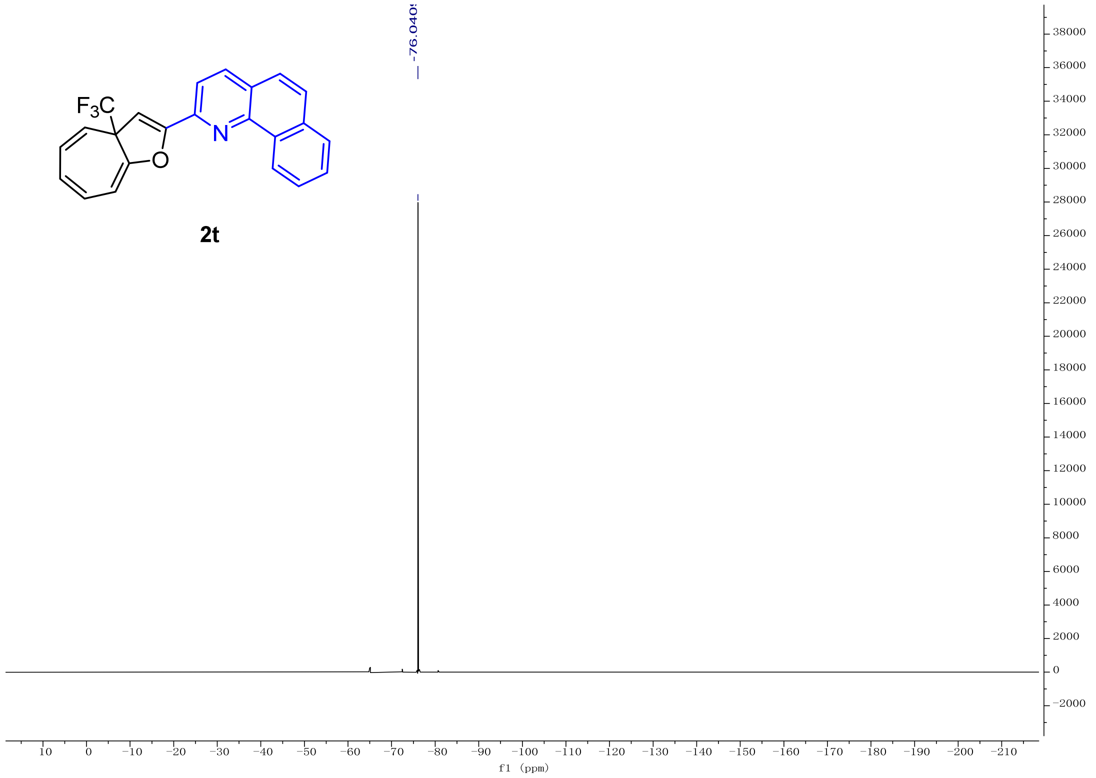

^{1}H NMR (300 MHz, CD_2Cl_2) of compound **2u**

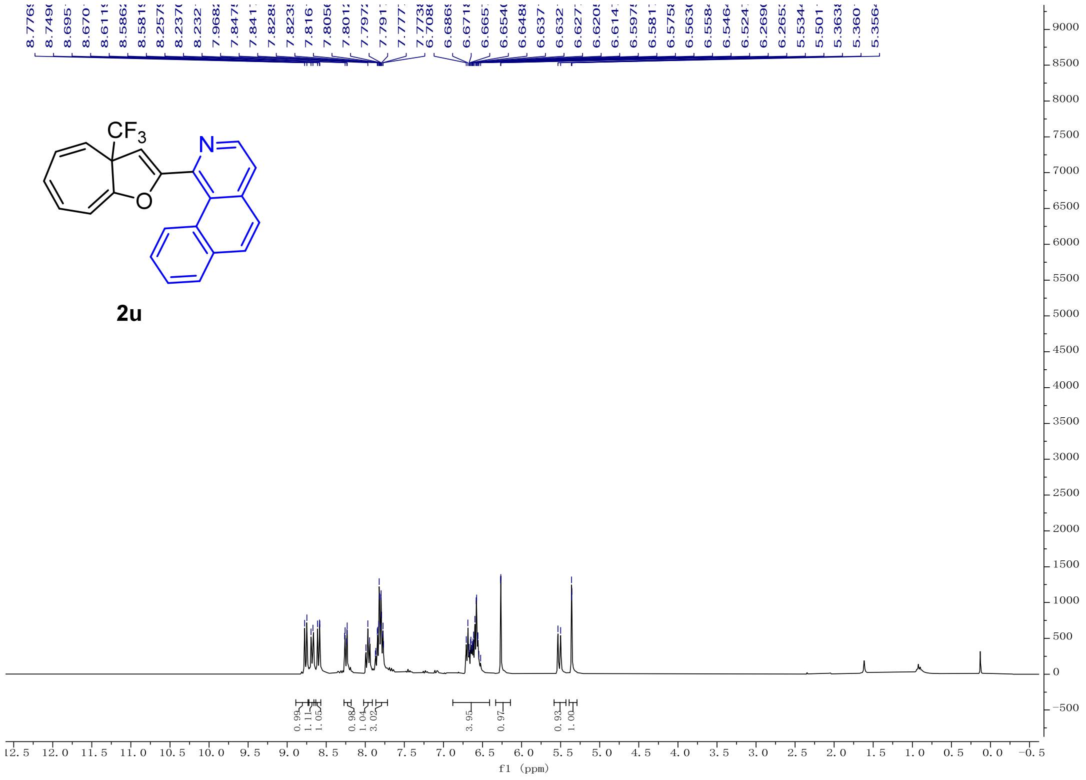

^{13}C NMR (75 MHz, CD_2Cl_2) of compound **2u**

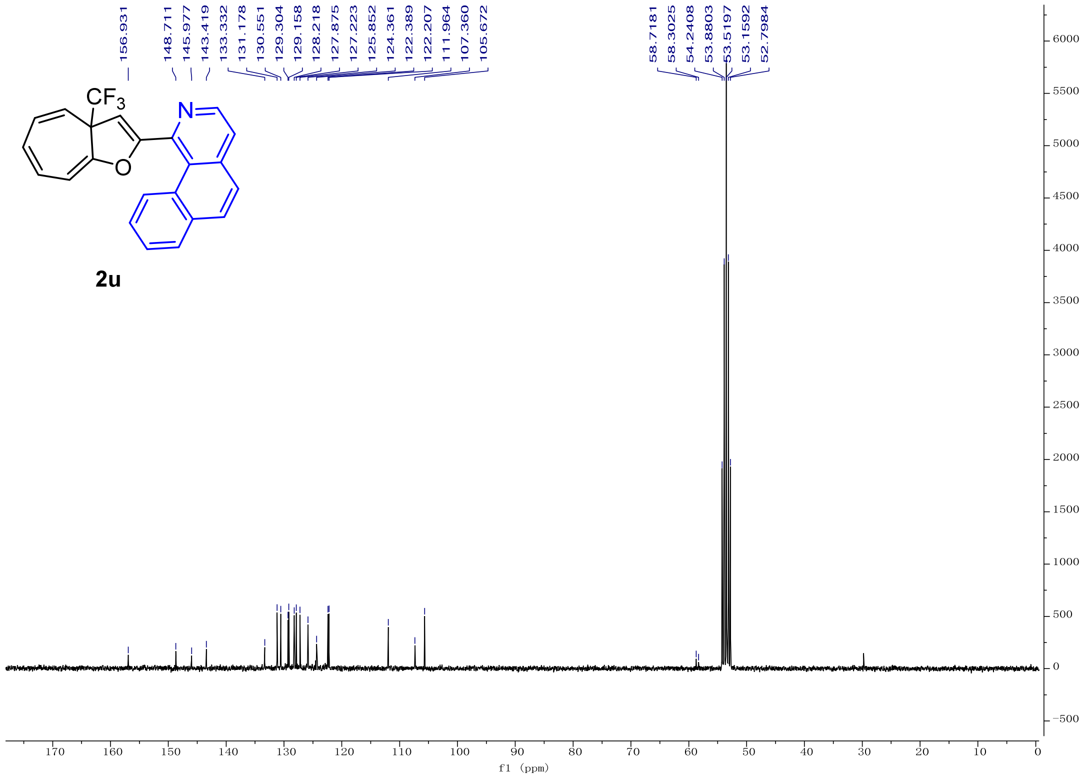

^{19}F NMR (565 MHz, CD_2Cl_2) of compound **2u**

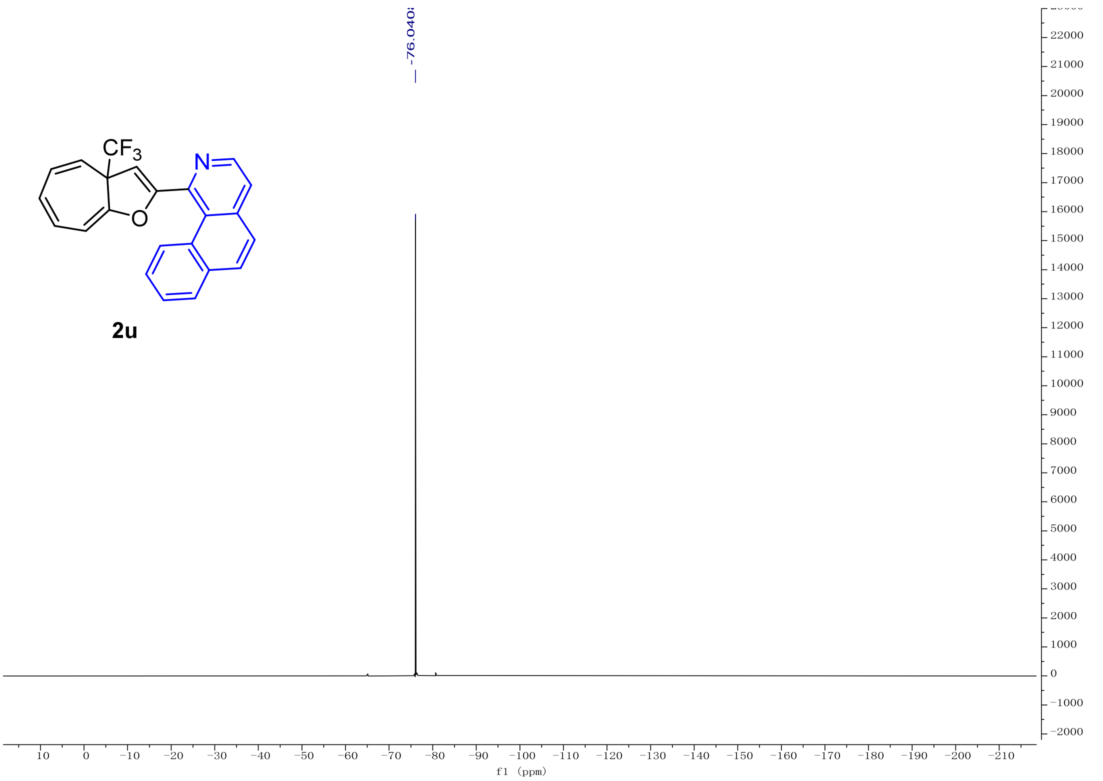

^{1}H NMR (600 MHz, $CDCl_3$) of compound **2v**

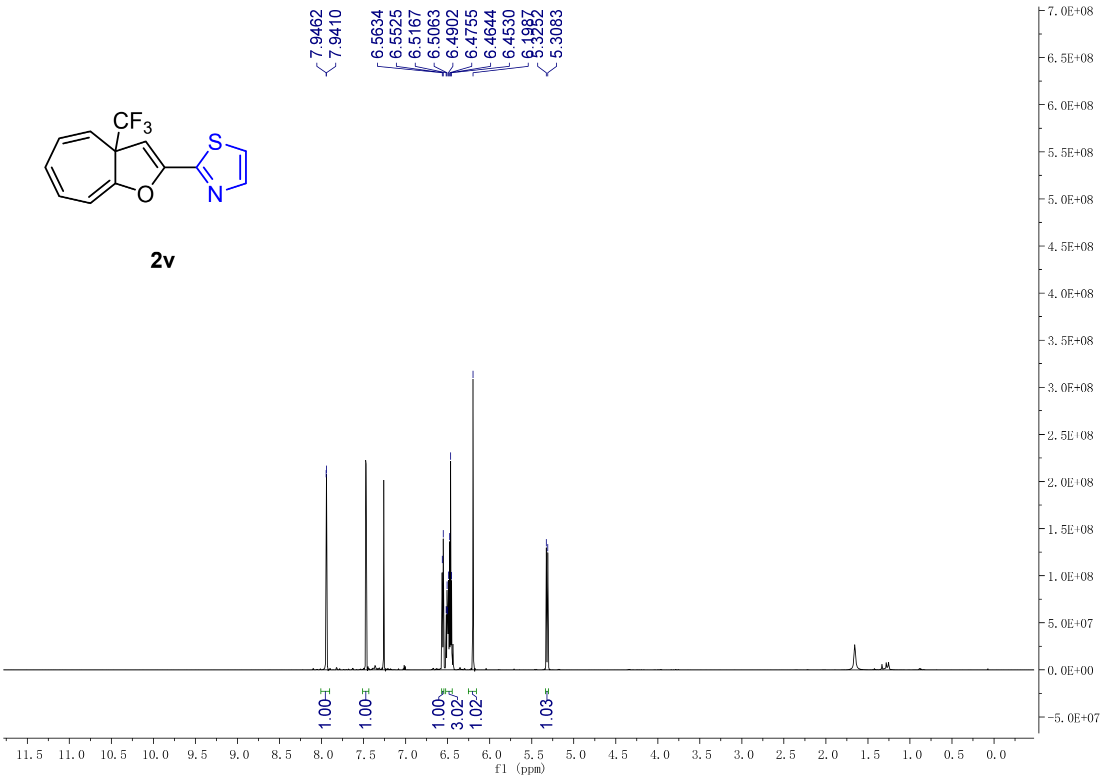

^{13}C NMR (151 MHz, $CDCl_3$) of compound **2v**

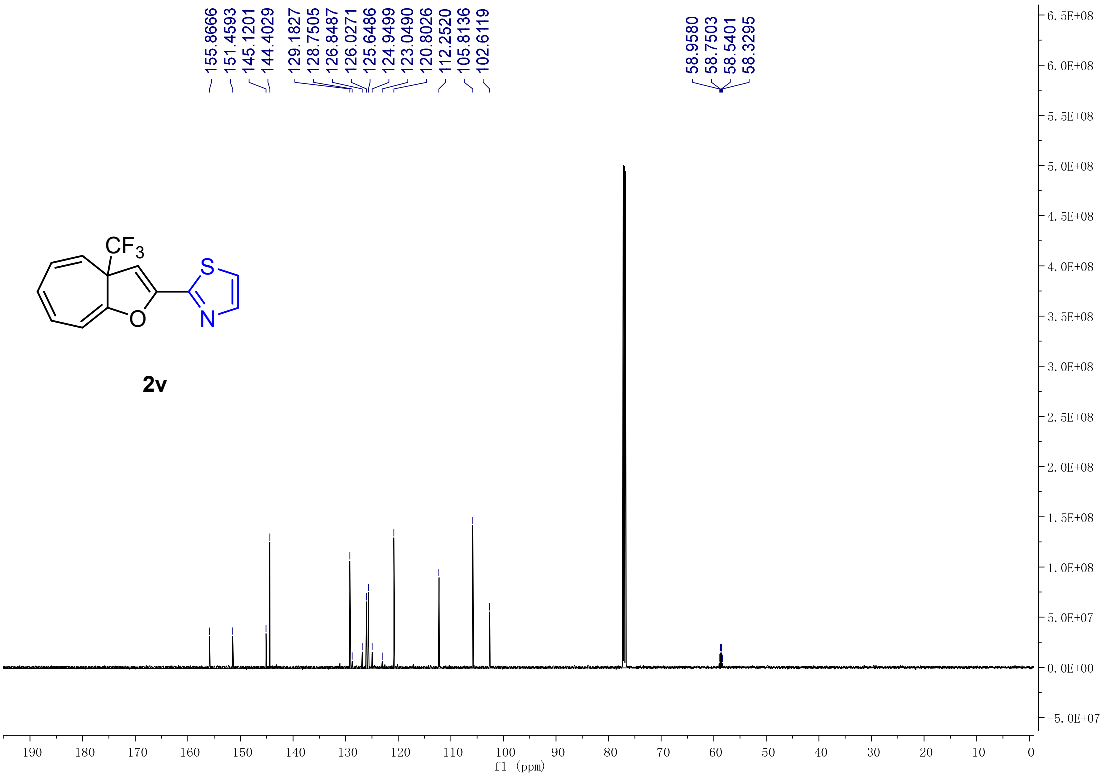

^{19}F NMR (565 MHz, $CDCl_3$) of compound **2v**

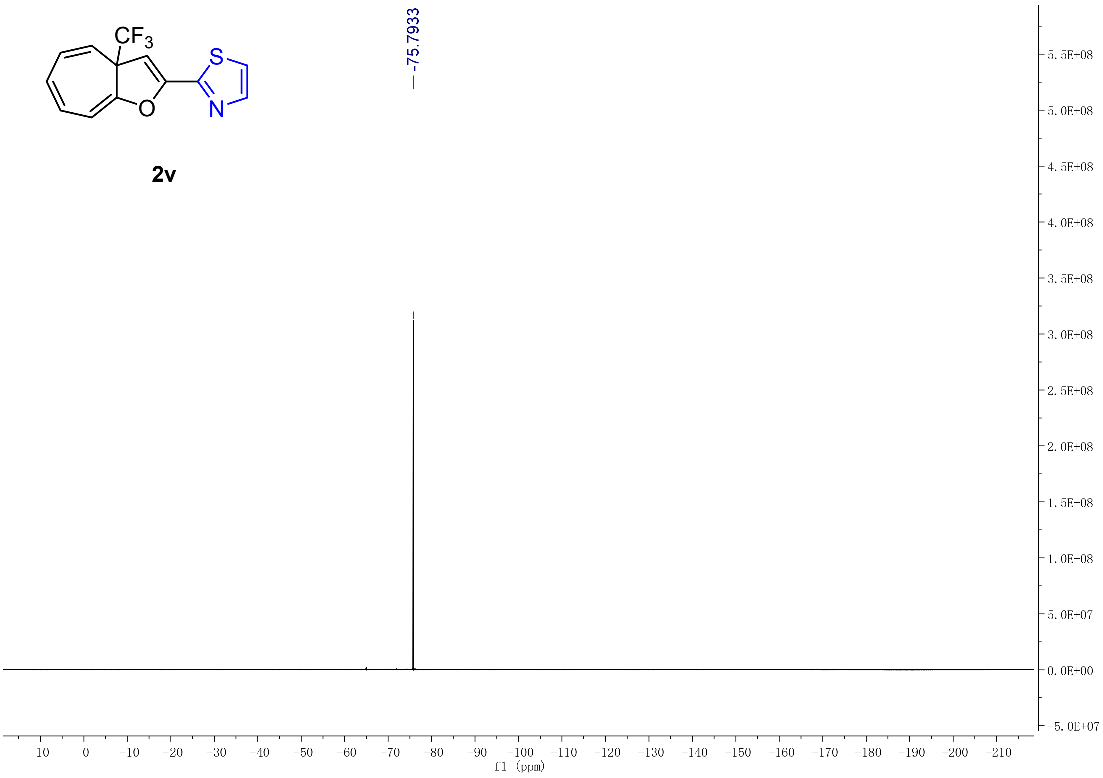

^{1}H NMR (300 MHz, $CDCl_3$) of compound **2w**

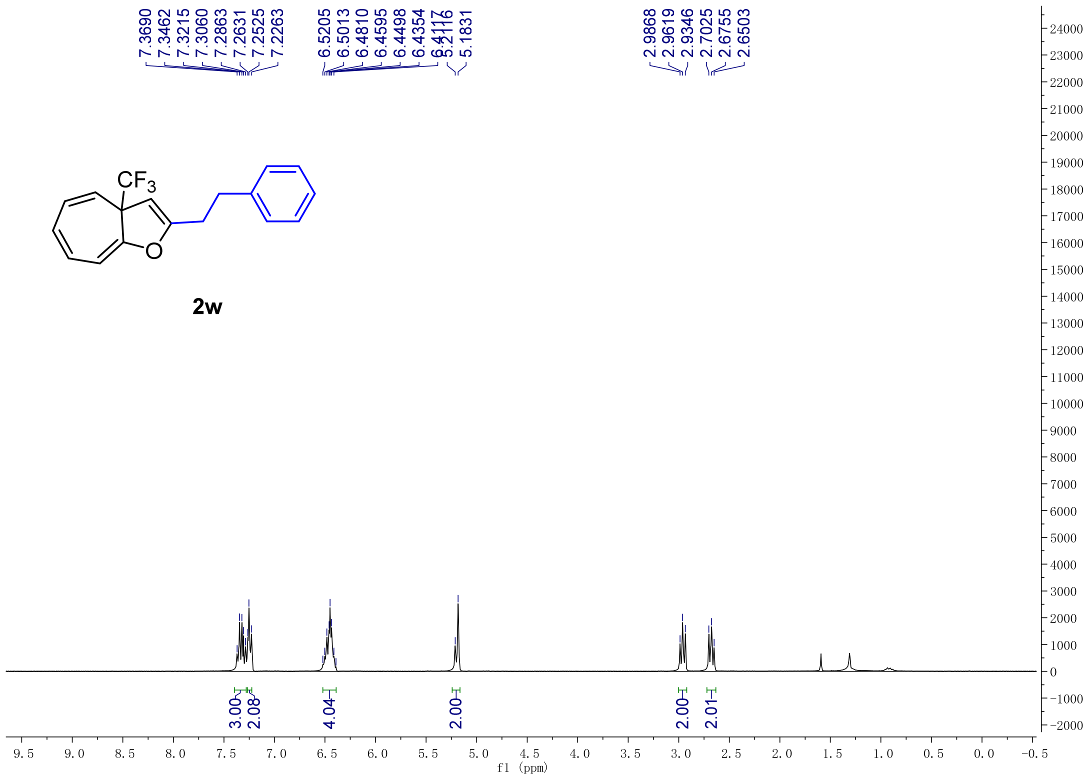

^{13}C NMR (75 MHz, $CDCl_3$) of compound **2w**

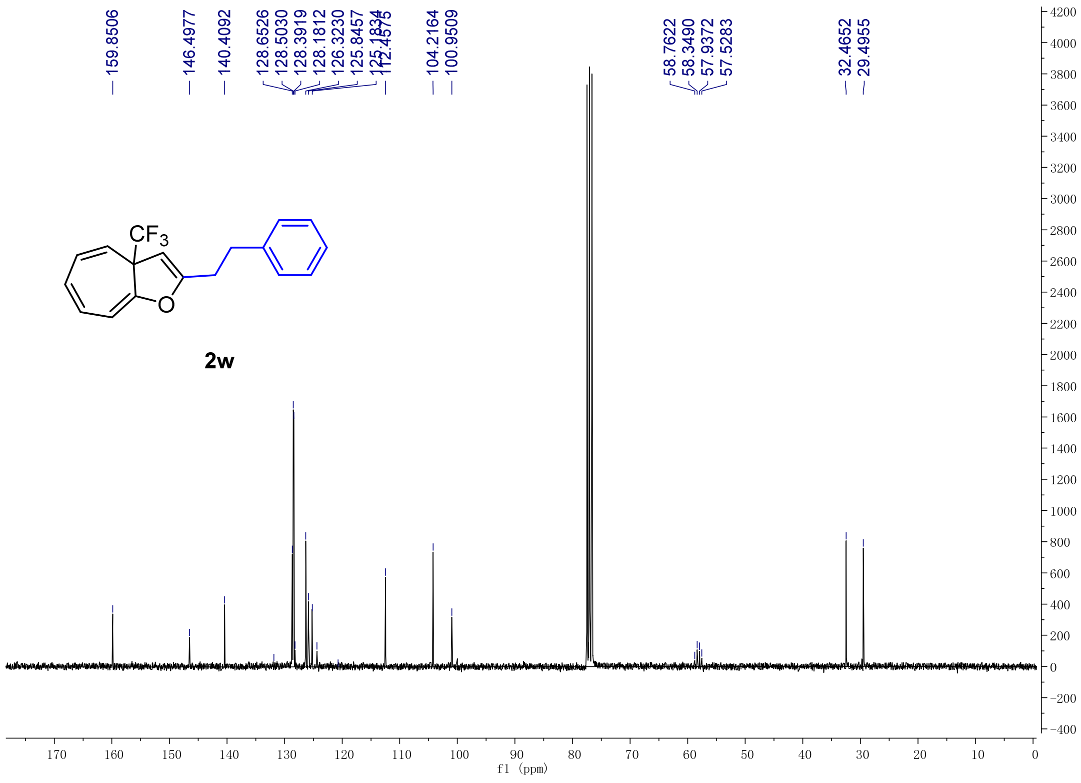

^{19}F NMR (565 MHz, $CDCl_3$) of compound **2w**

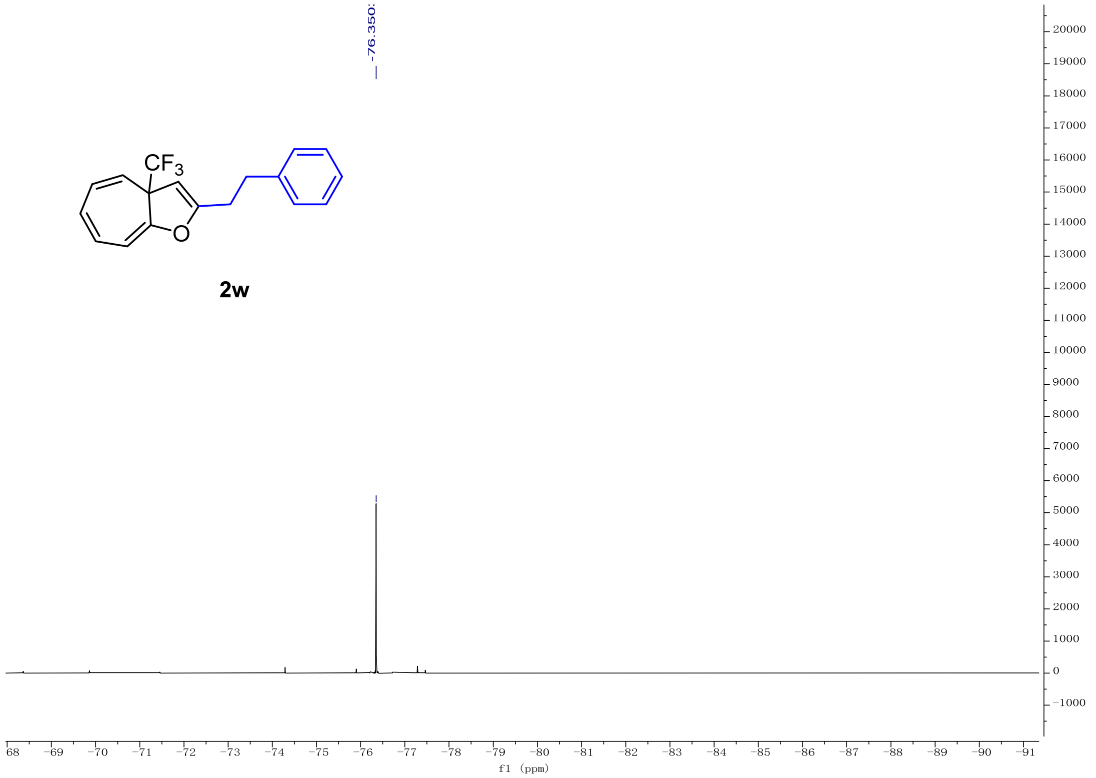

^{1}H NMR (600 MHz, $CDCl_3$) of compound **2x**

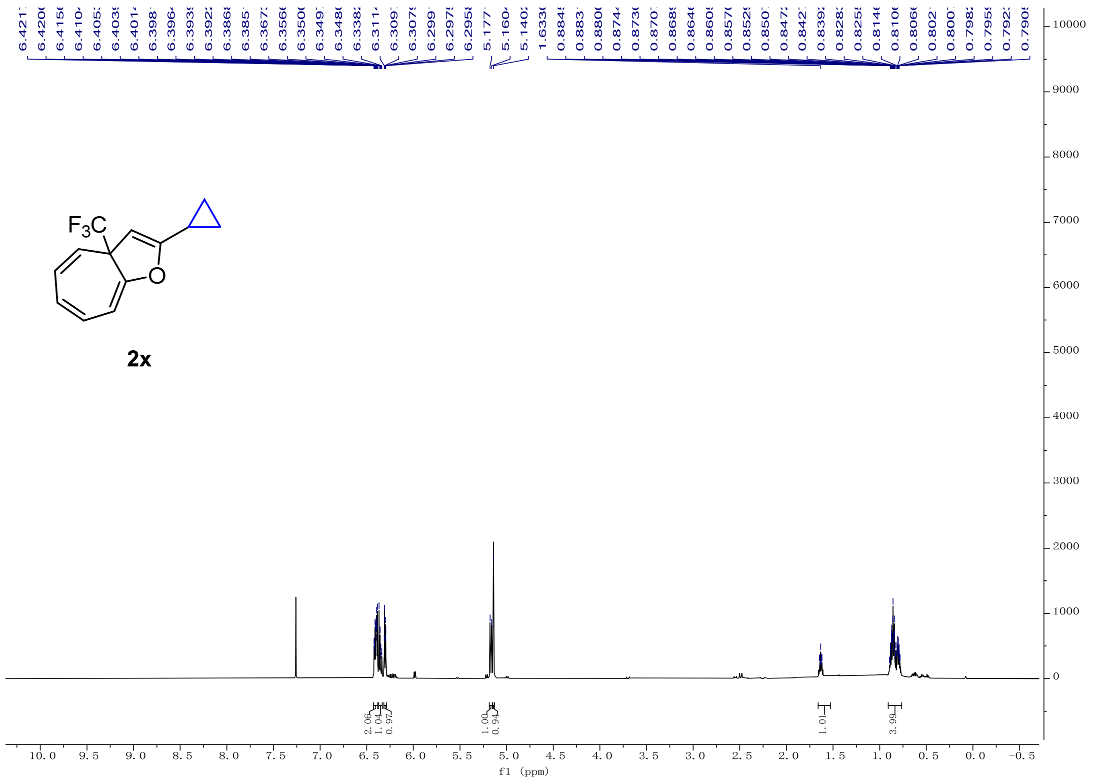

^{13}C NMR (151 MHz, $CDCl_3$) of compound **2x**

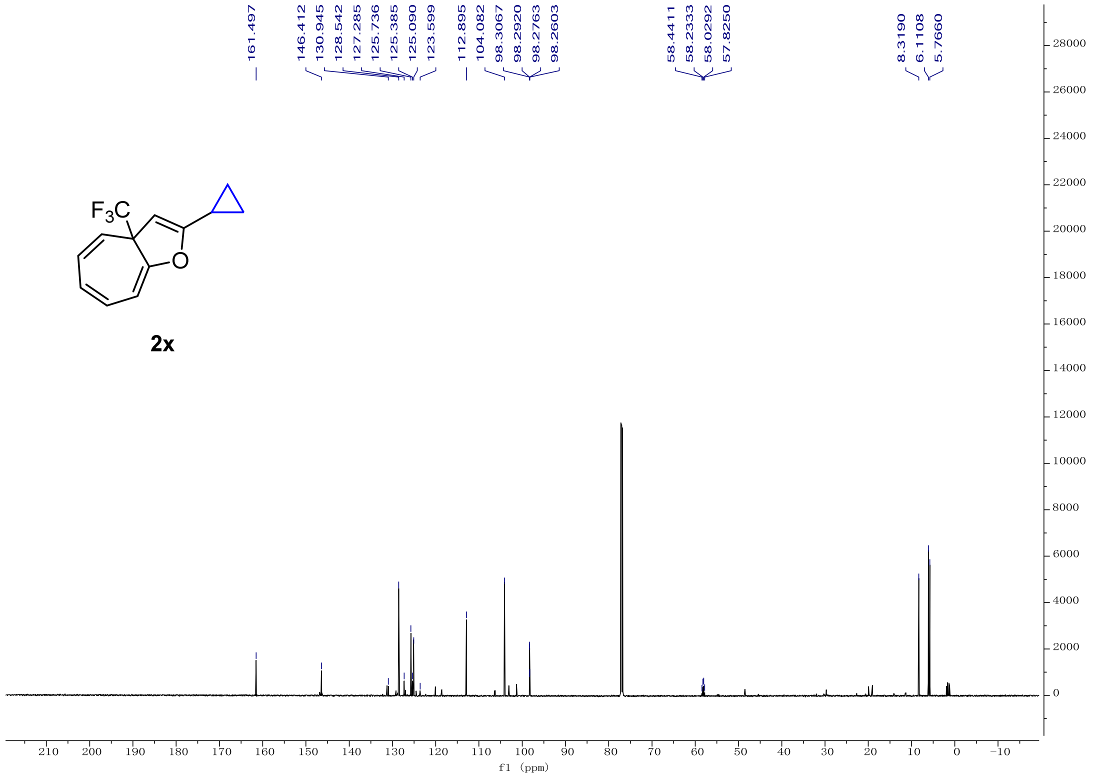

^{19}F NMR (565 MHz, $CDCl_3$) of compound **2x**

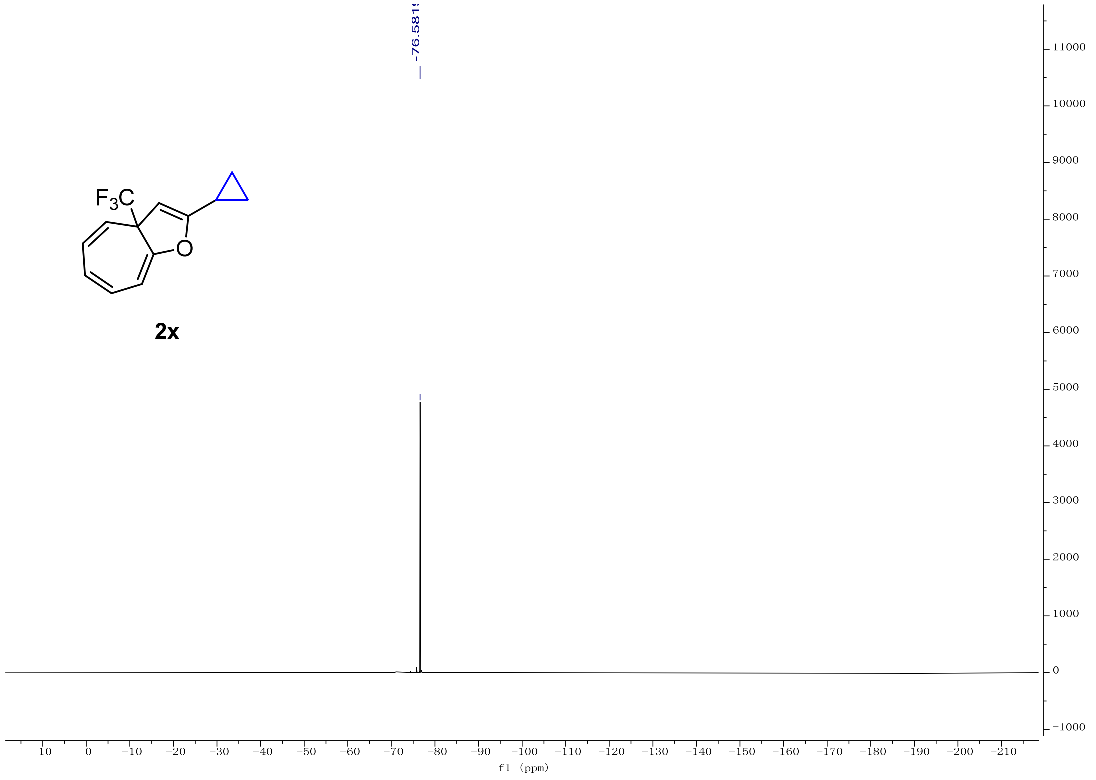

^{1}H NMR (300 MHz, $CDCl_3$) of compound **2y**

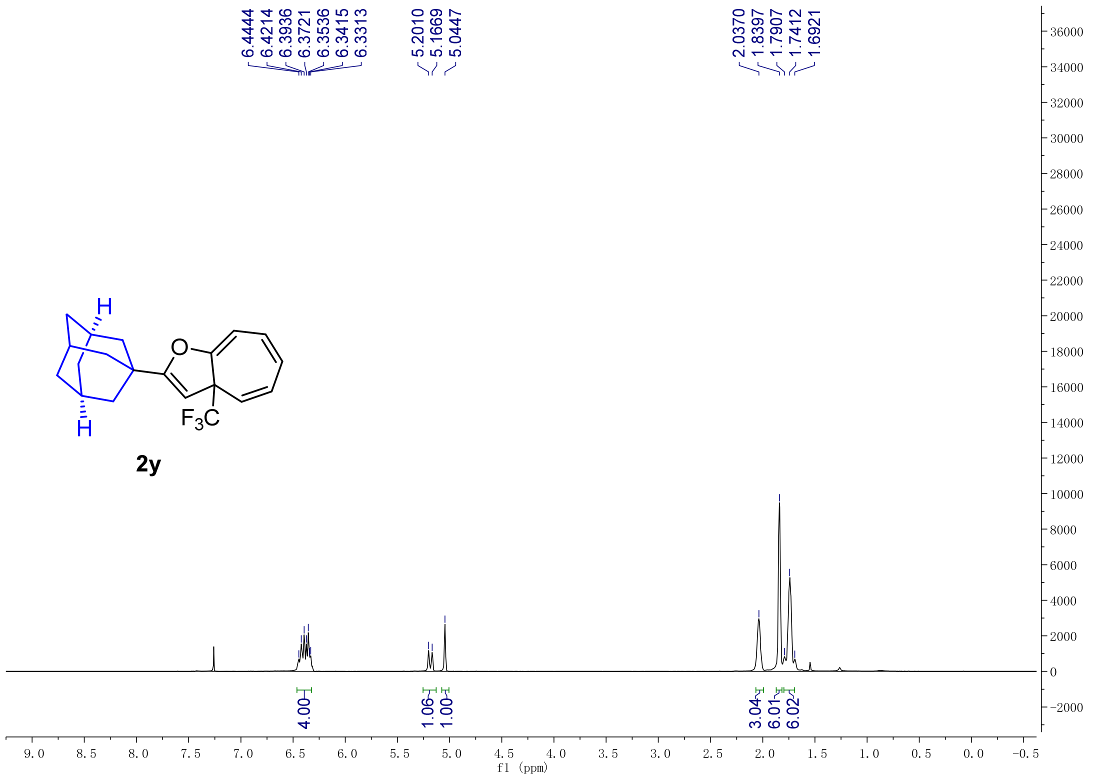

^{13}C NMR (75 MHz, $CDCl_3$) of compound **2y**

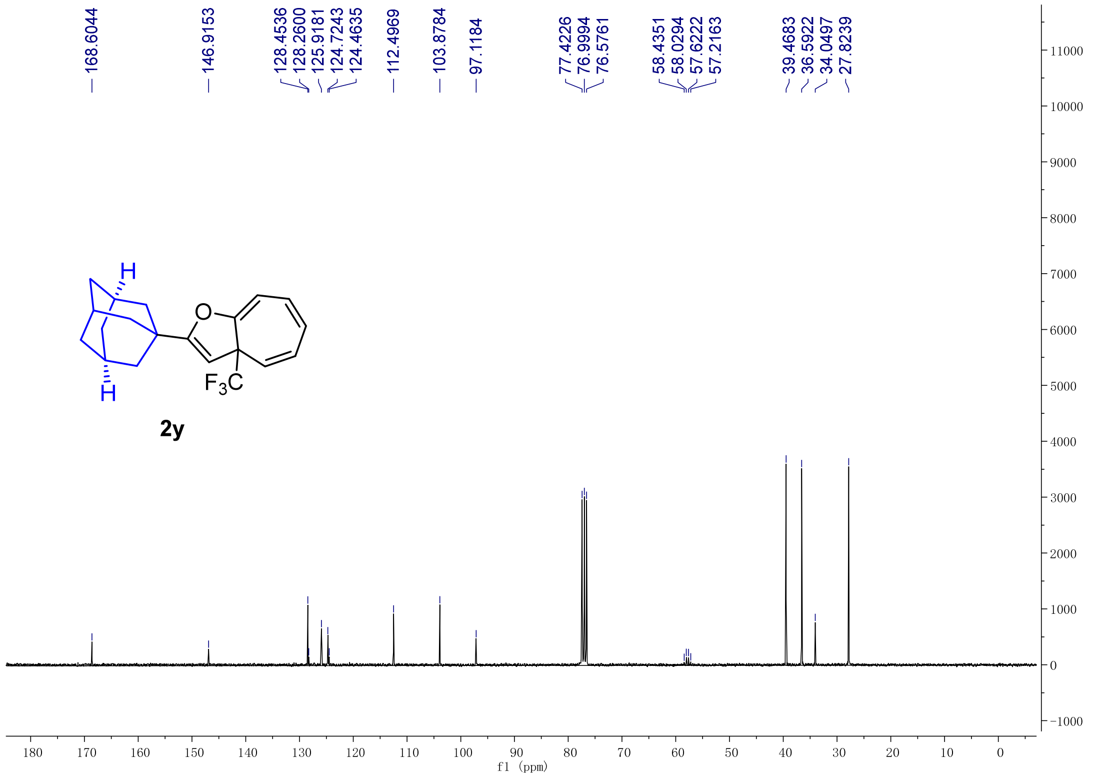

^{19}F NMR (565 MHz, $CDCl_3$) δ -76.7 of compound **2y**

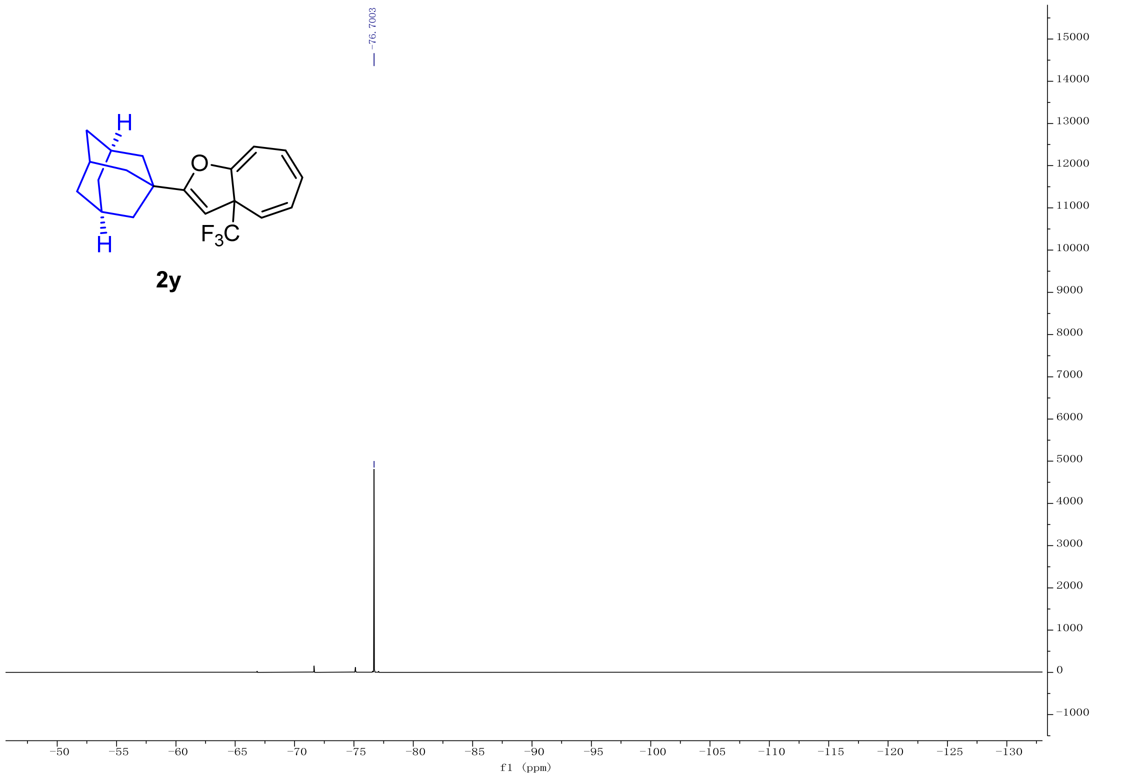

^{1}H NMR (400 MHz, $CDCl_3$) of compound **2za**

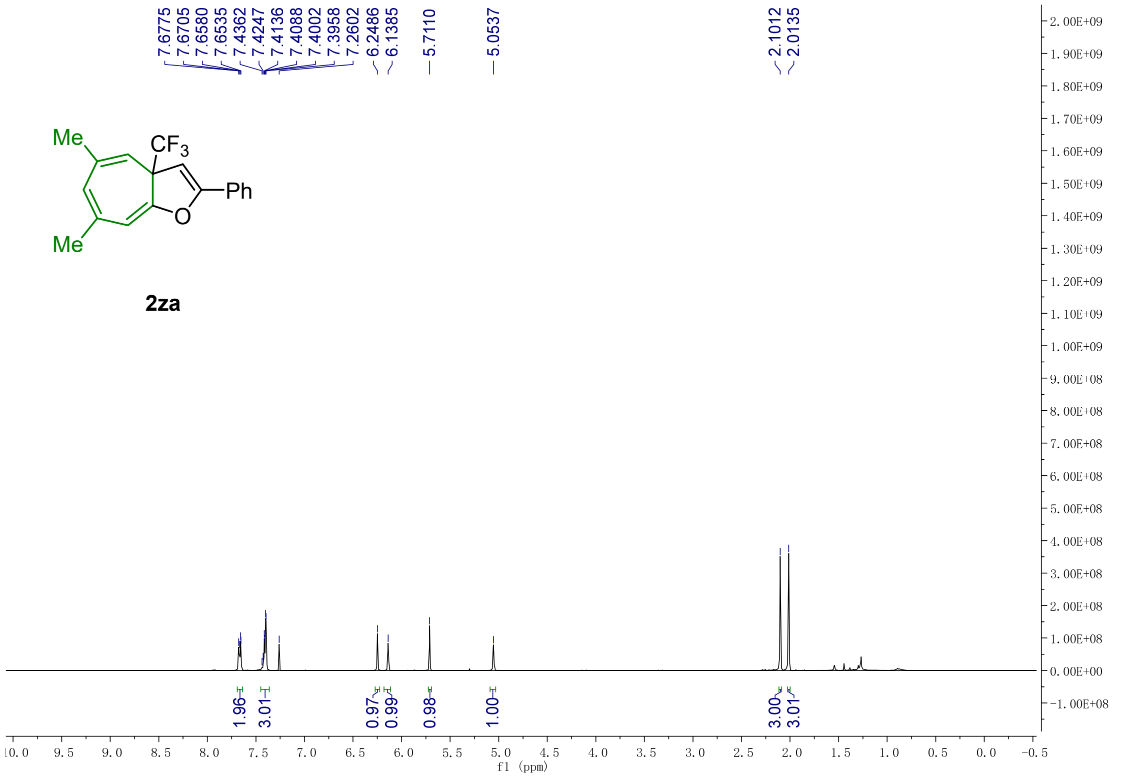

^{13}C NMR (101 MHz, $CDCl_3$) of compound **2za**

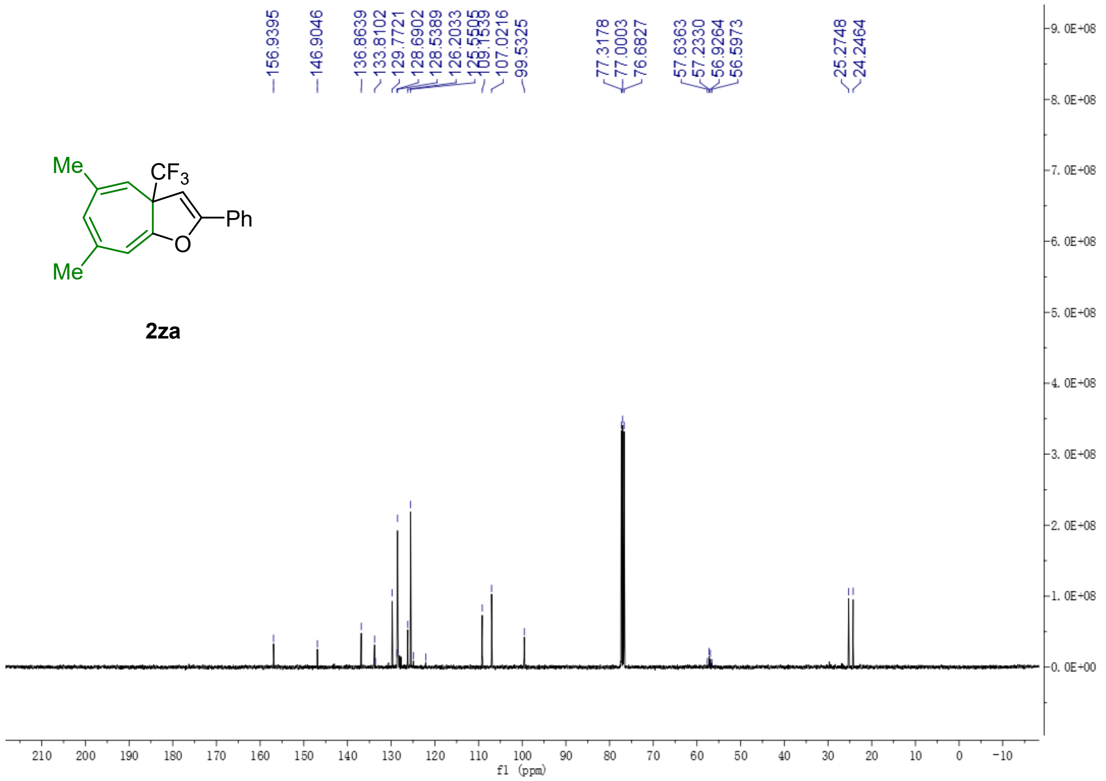

^{19}F NMR (376 MHz, $CDCl_3$) of compound **2za**

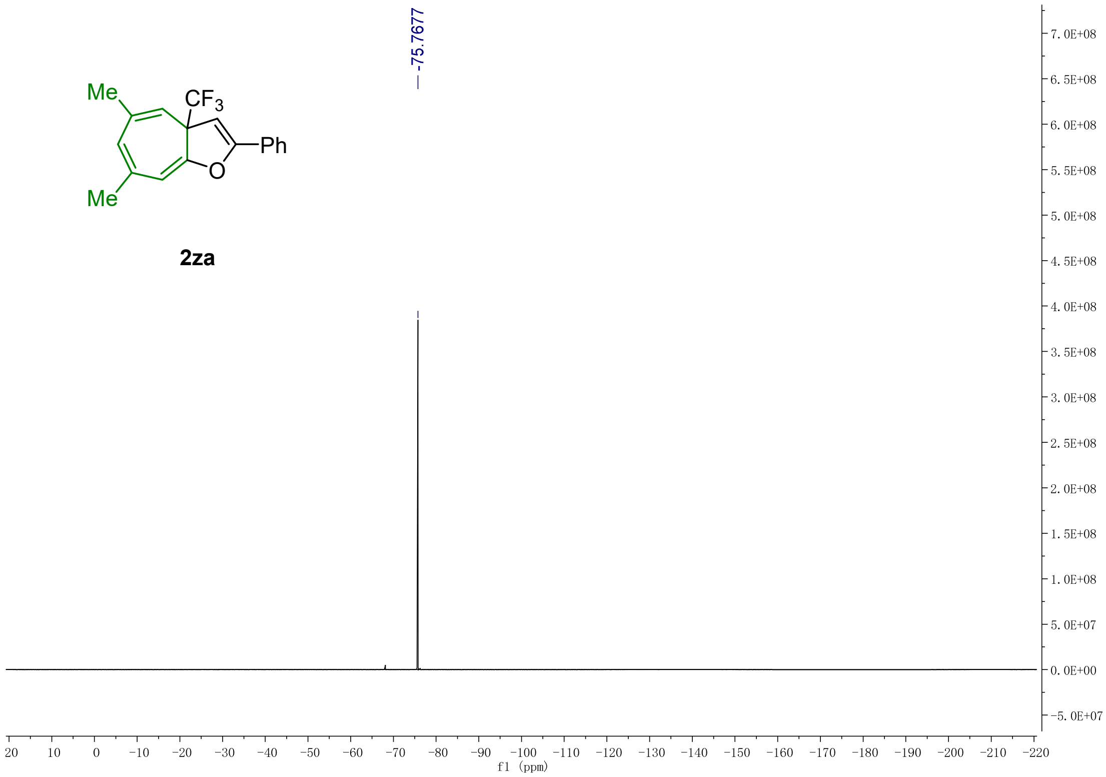

^{1}H NMR (600 MHz, $CDCl_3$) of compound **2zb**

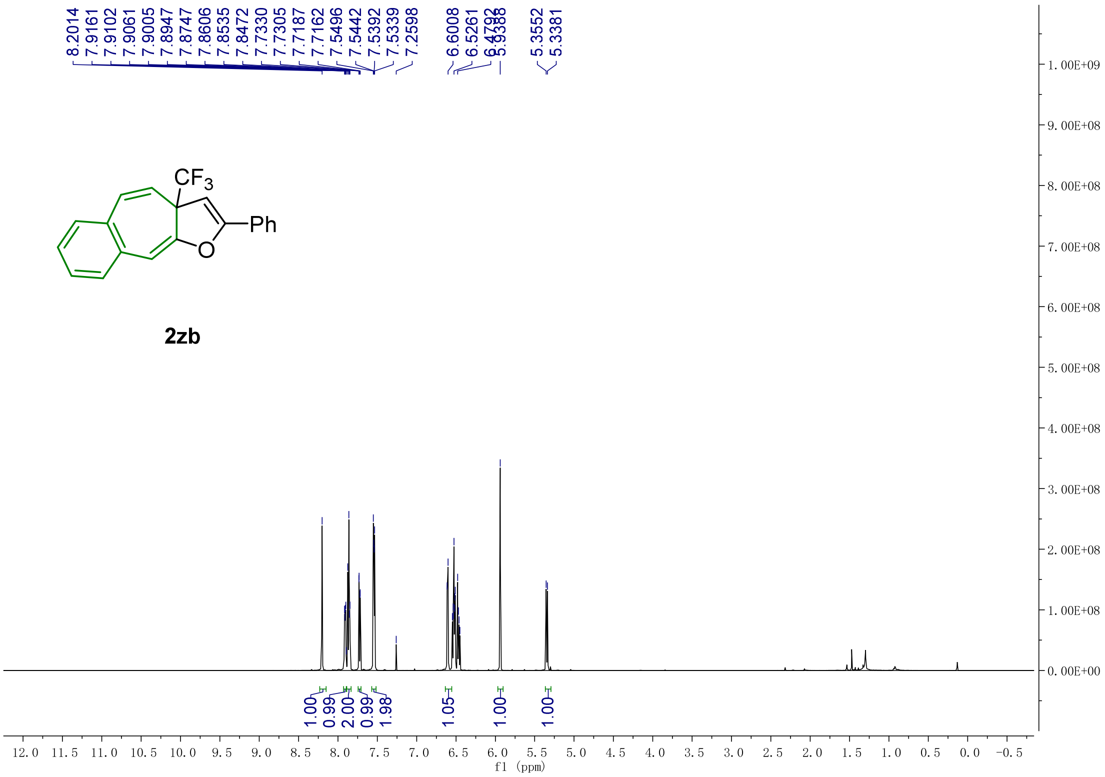

^{13}C NMR (151 MHz, $CDCl_3$) of compound **2zb**

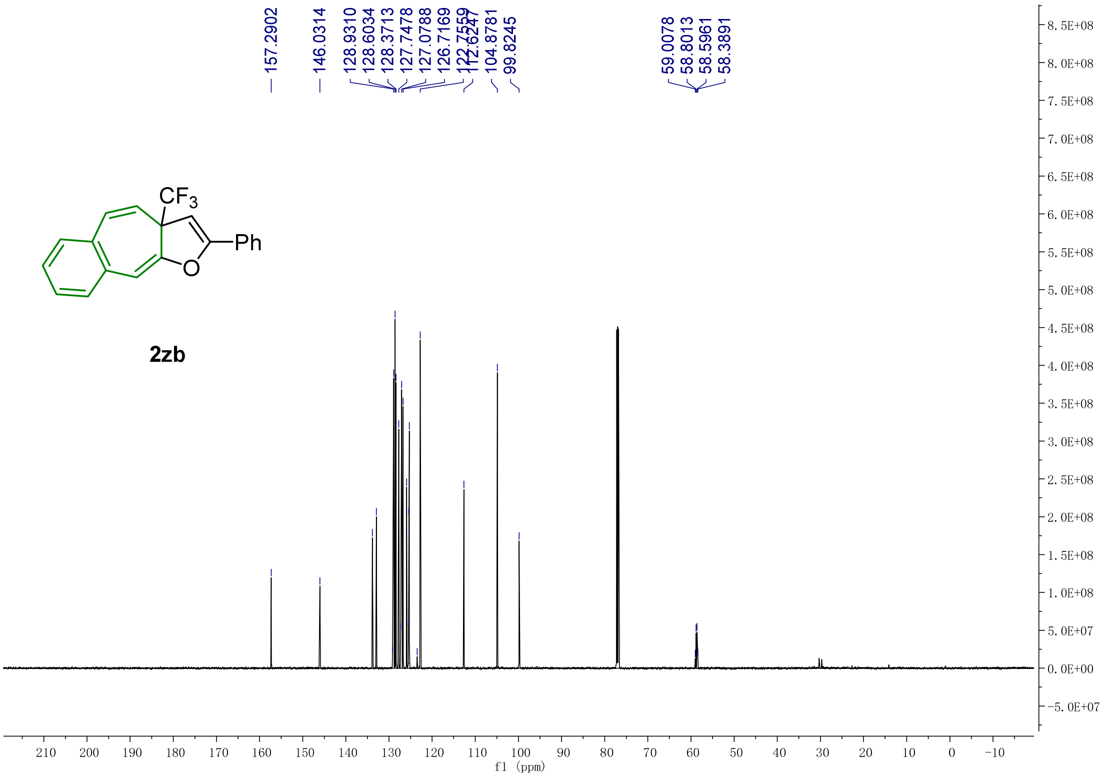

^{19}F NMR (565 MHz, $CDCl_3$) of compound **2zb**

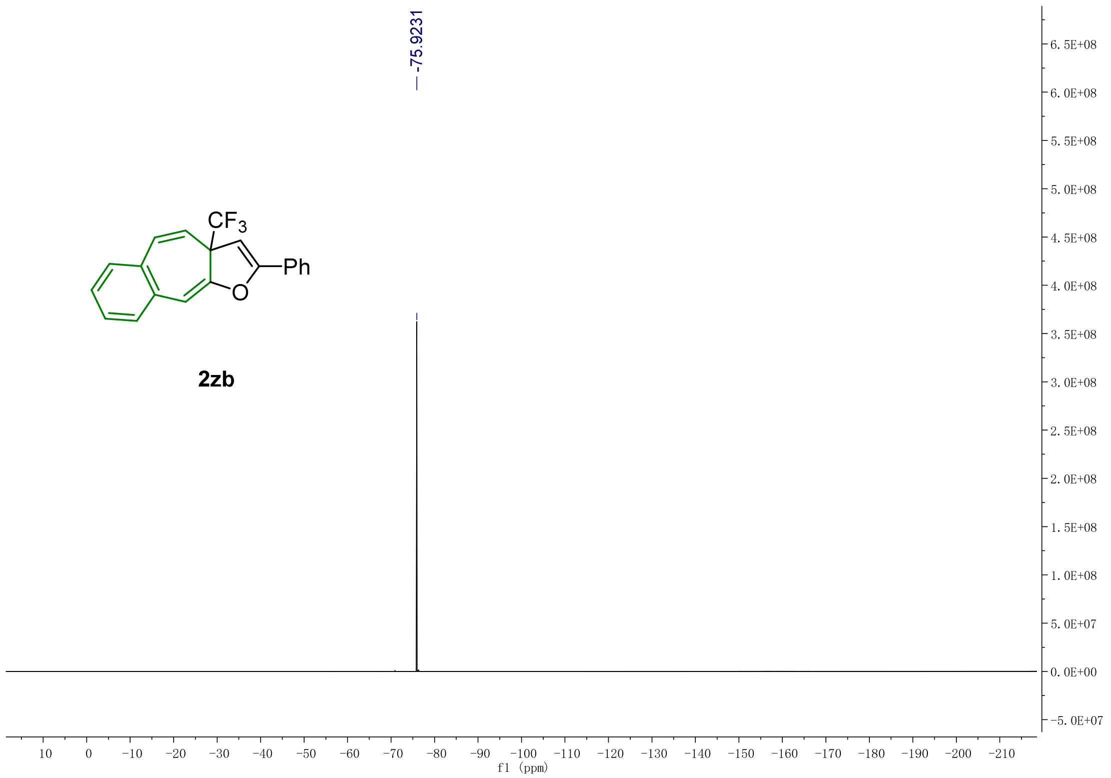

^{1}H NMR (300 MHz, $CDCl_3$) of compound **2zc**

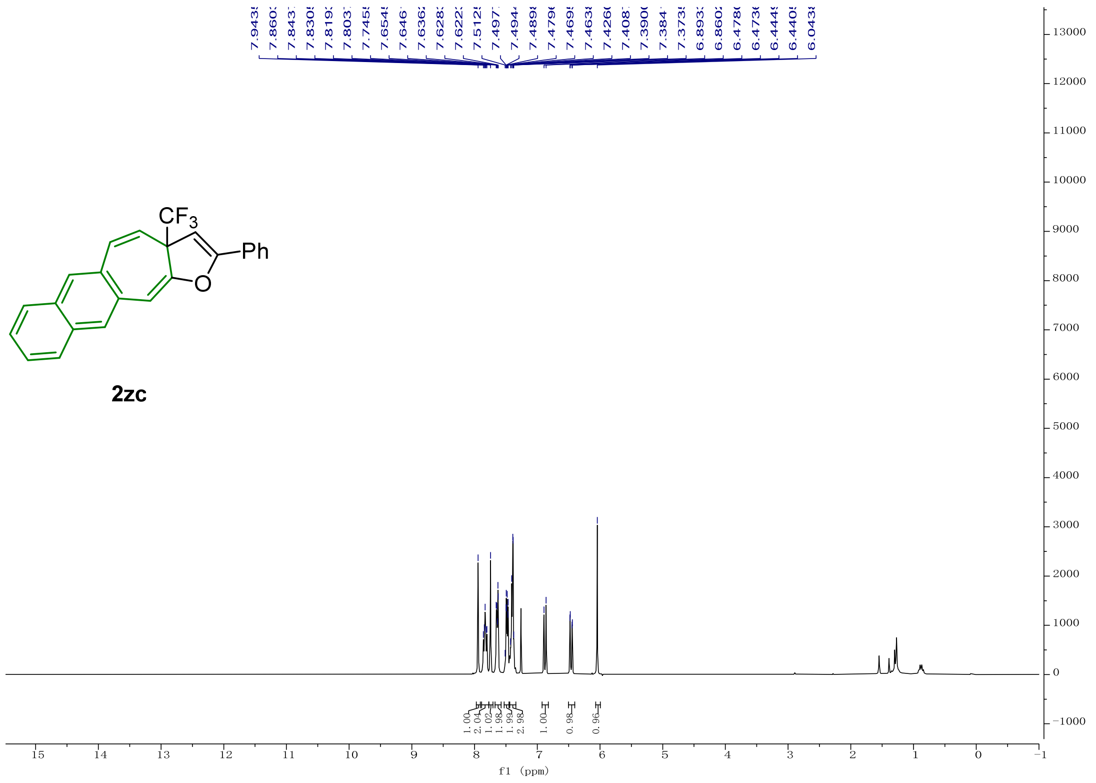

^{13}C NMR (151 MHz, $CDCl_3$) of compound **2zc**

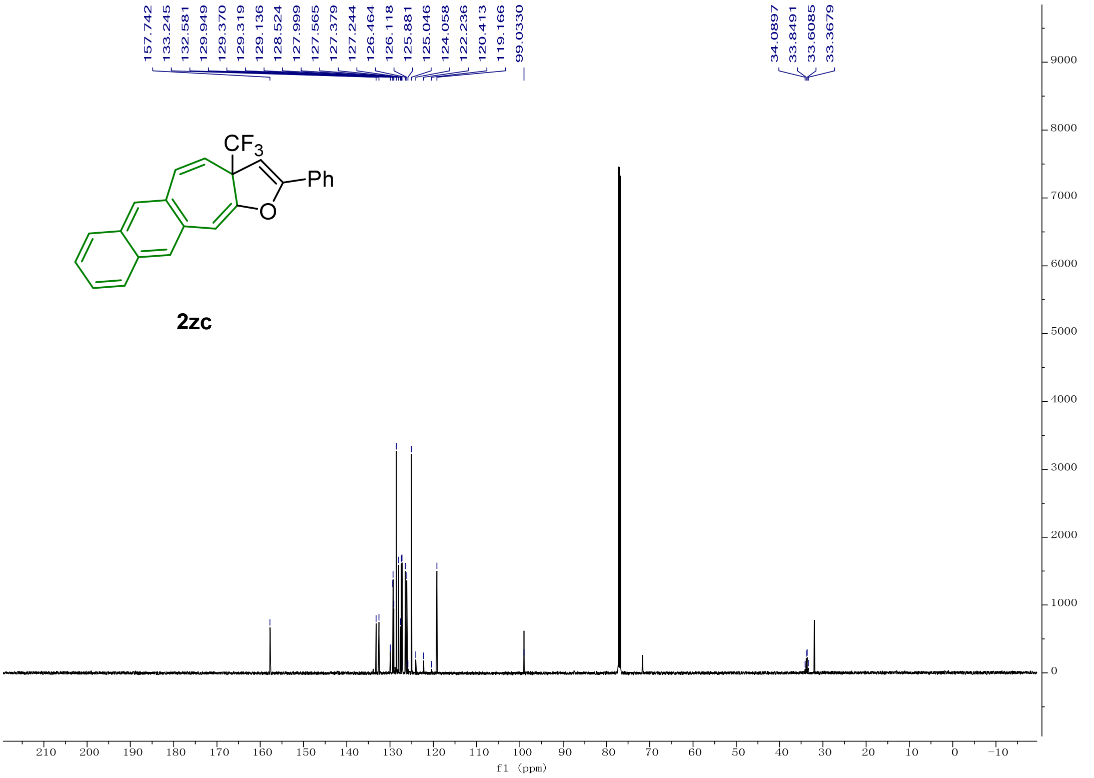

^{19}F NMR (565 MHz, $CDCl_3$ of compound **2zc**

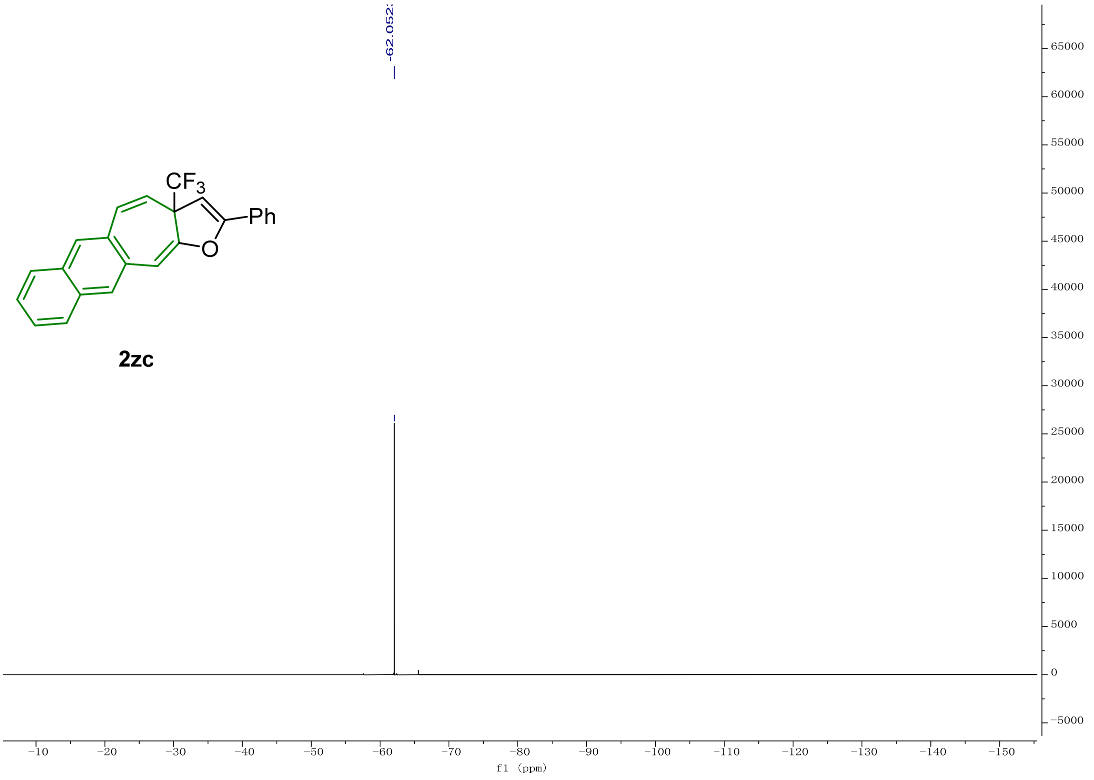

^{1}H NMR (600 MHz, $CDCl_3$) of compound **2zd-1**

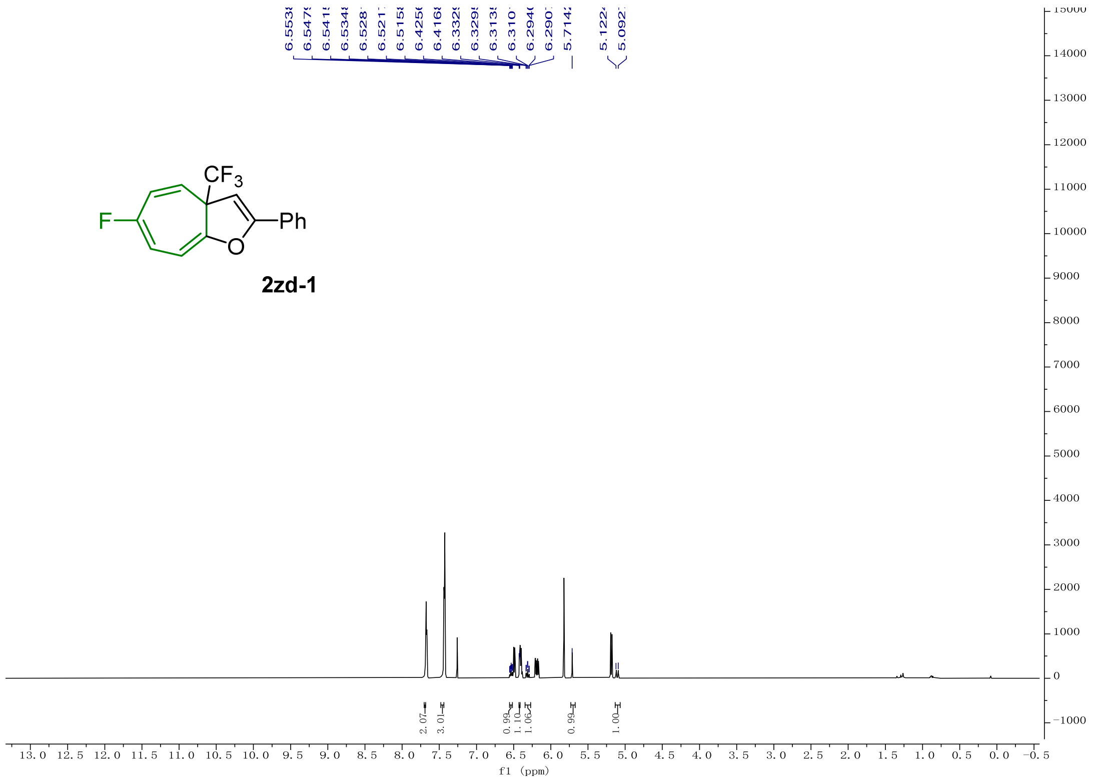

^{1}H NMR (600 MHz, $CDCl_3$) of compound **2zd-2**

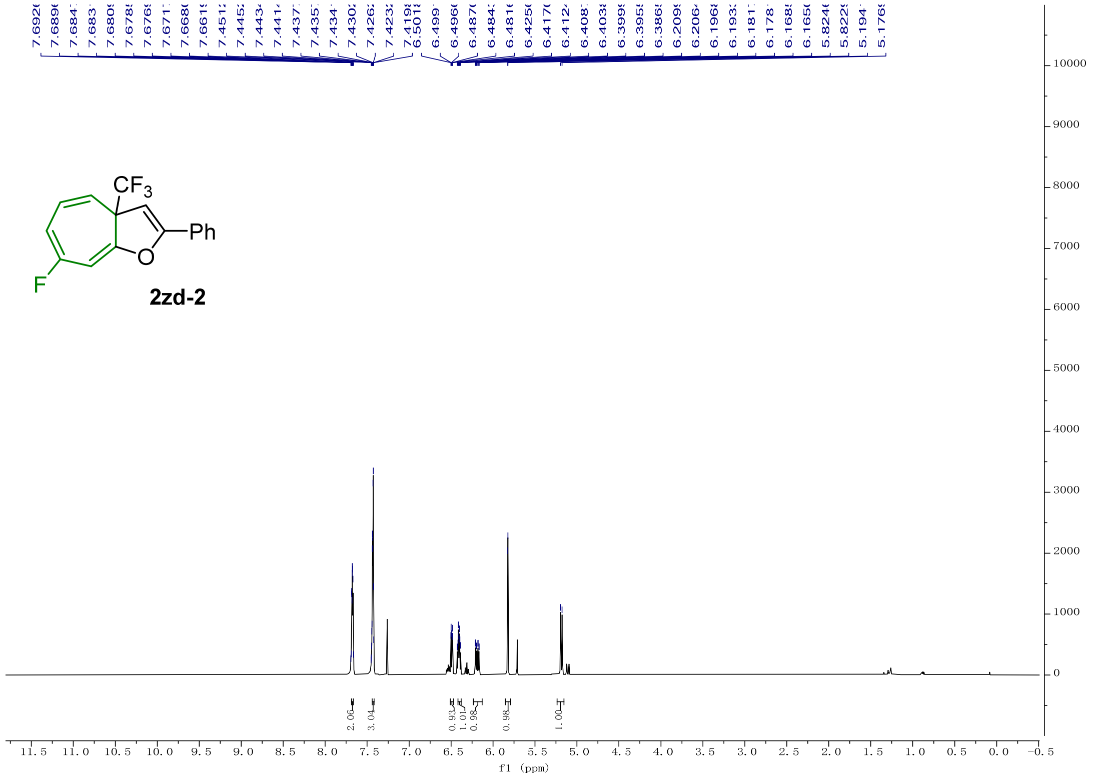

^{13}C NMR (151 MHz, $CDCl_3$) of compound **2zd-1** and **2zd-2**

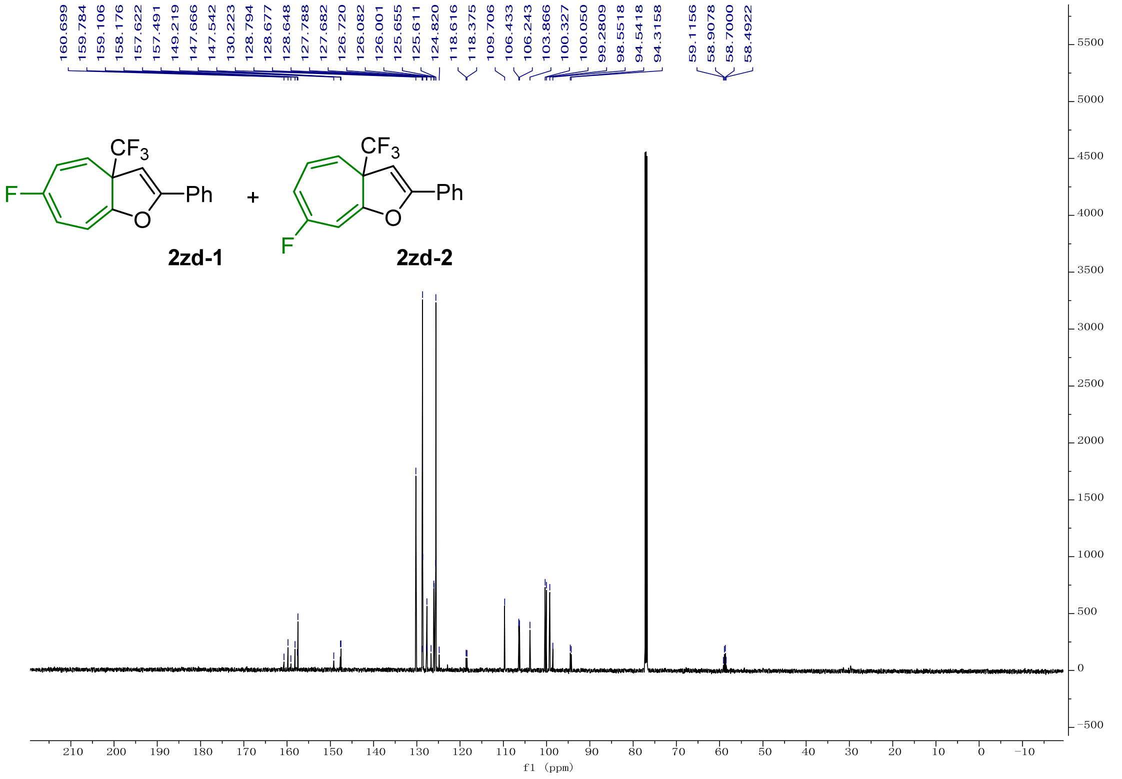

^{19}F NMR (565 MHz, $CDCl_3$) of compound **2zd-1** and **2zd-2**

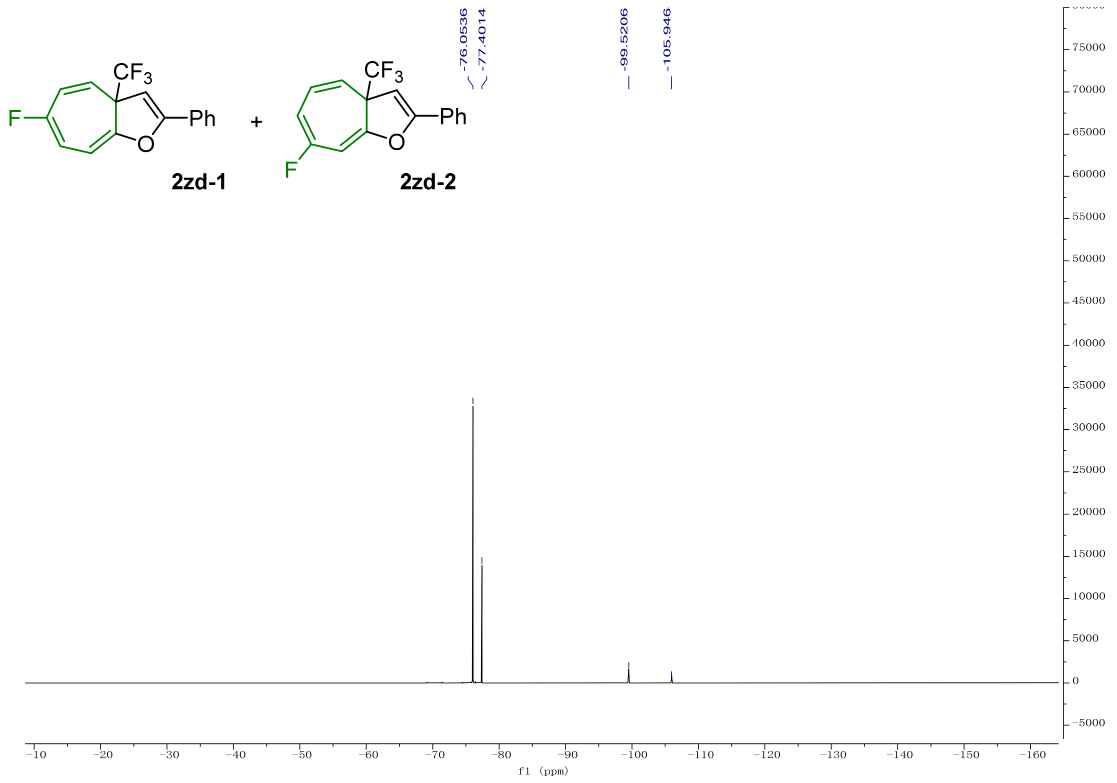

^{1}H NMR (600 MHz, $CDCl_3$) of compound **2ze**

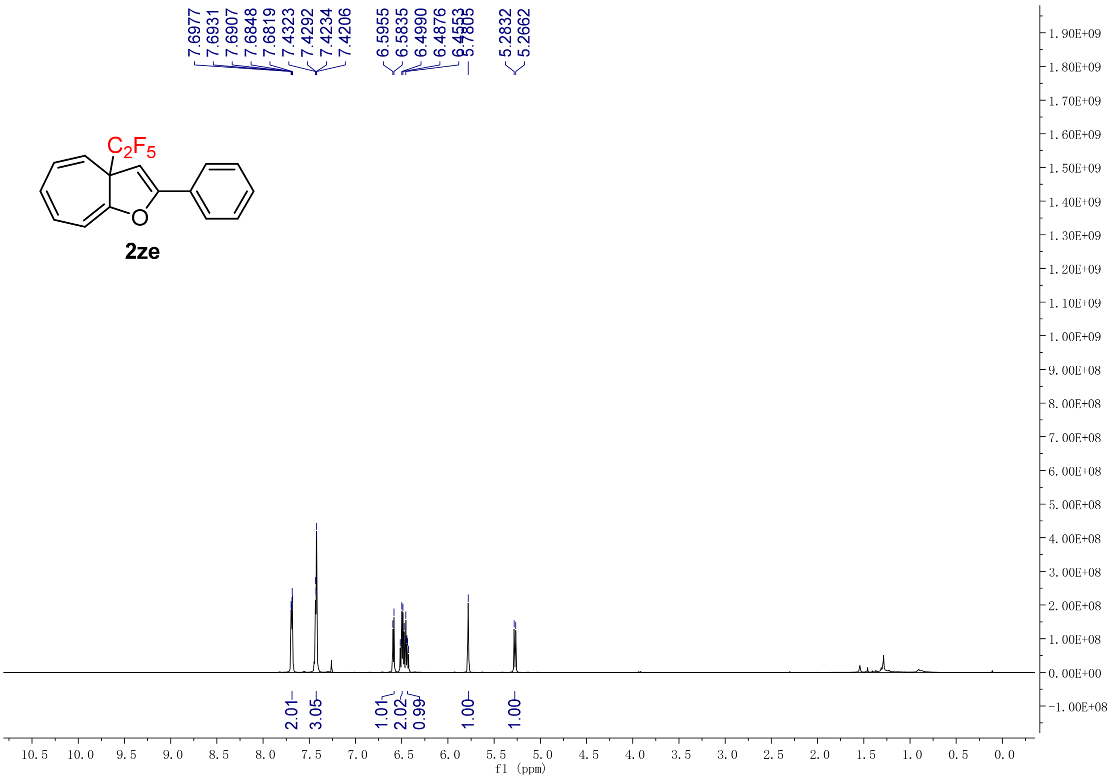

^{13}C NMR (151 MHz, $CDCl_3$) of compound **2ze**

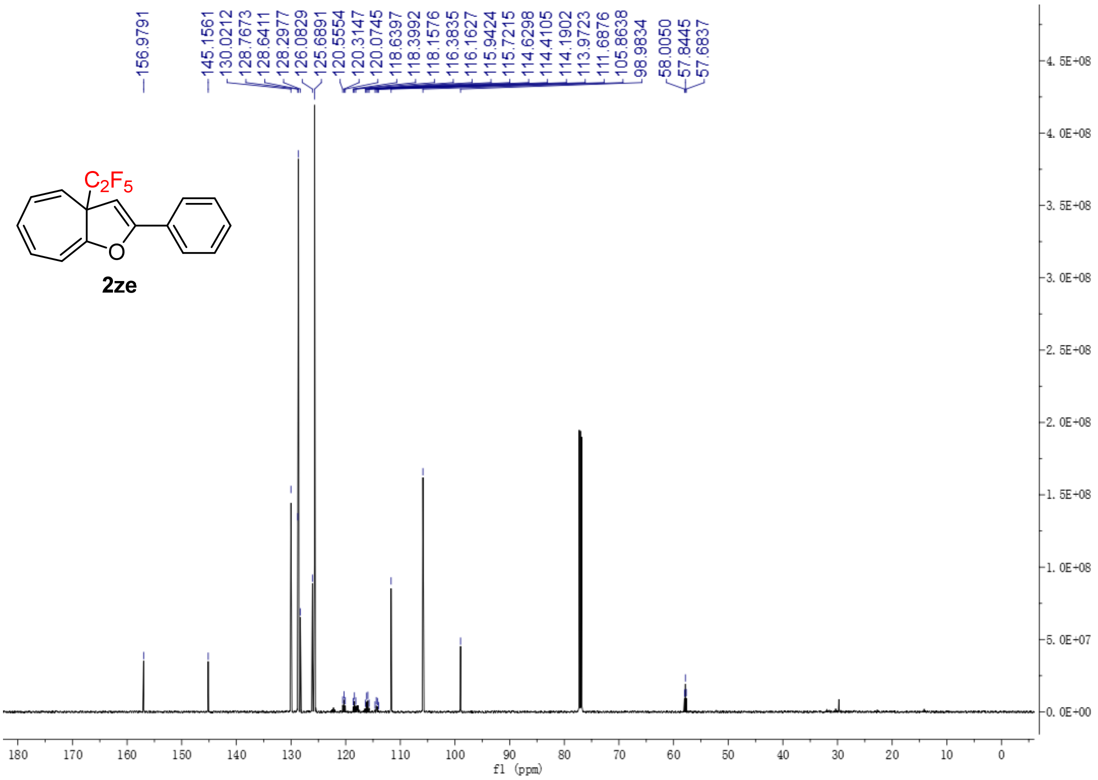

^{19}F NMR (565 MHz, $CDCl_3$) of compound **2ze**

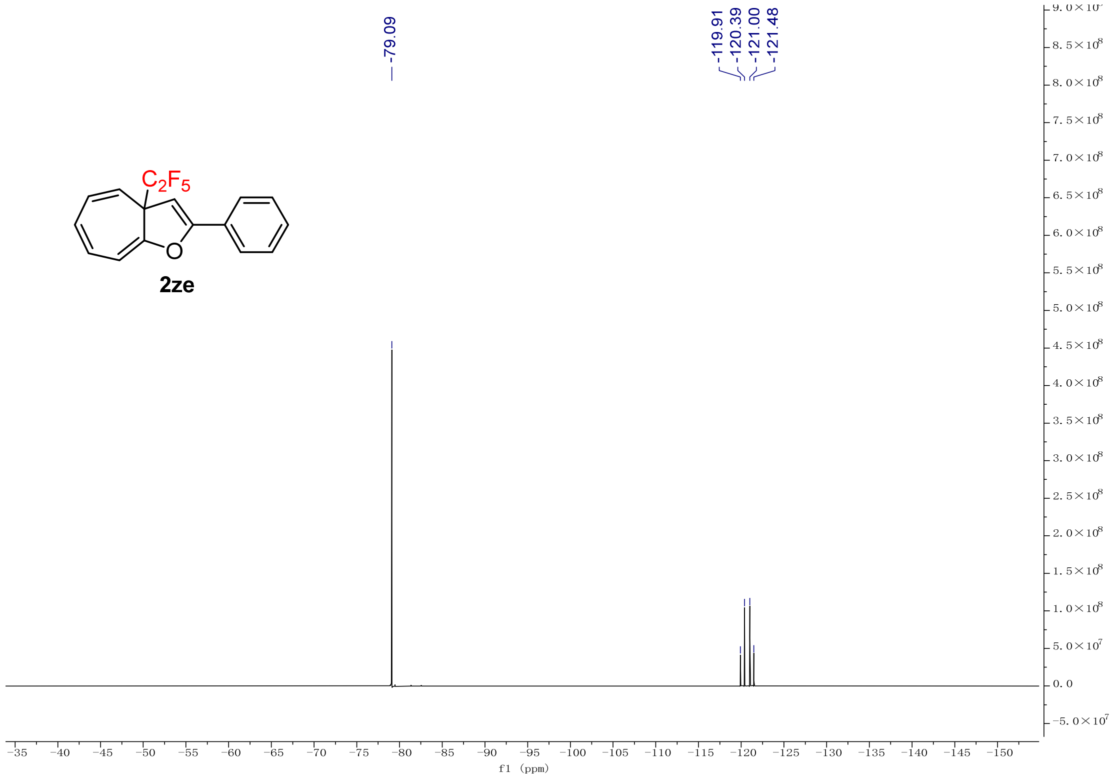

^{1}H NMR (400 MHz, $CDCl_3$) of compound **2zf**

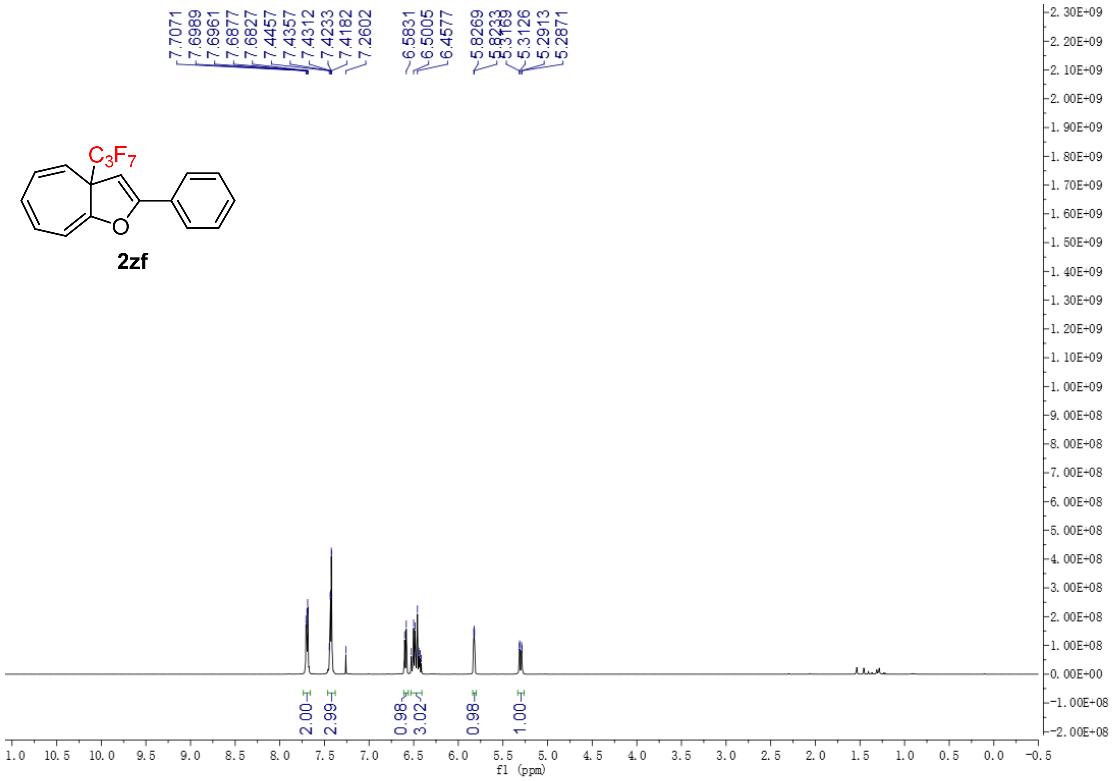

^{13}C NMR (101 MHz, $CDCl_3$) of compound **2zf**

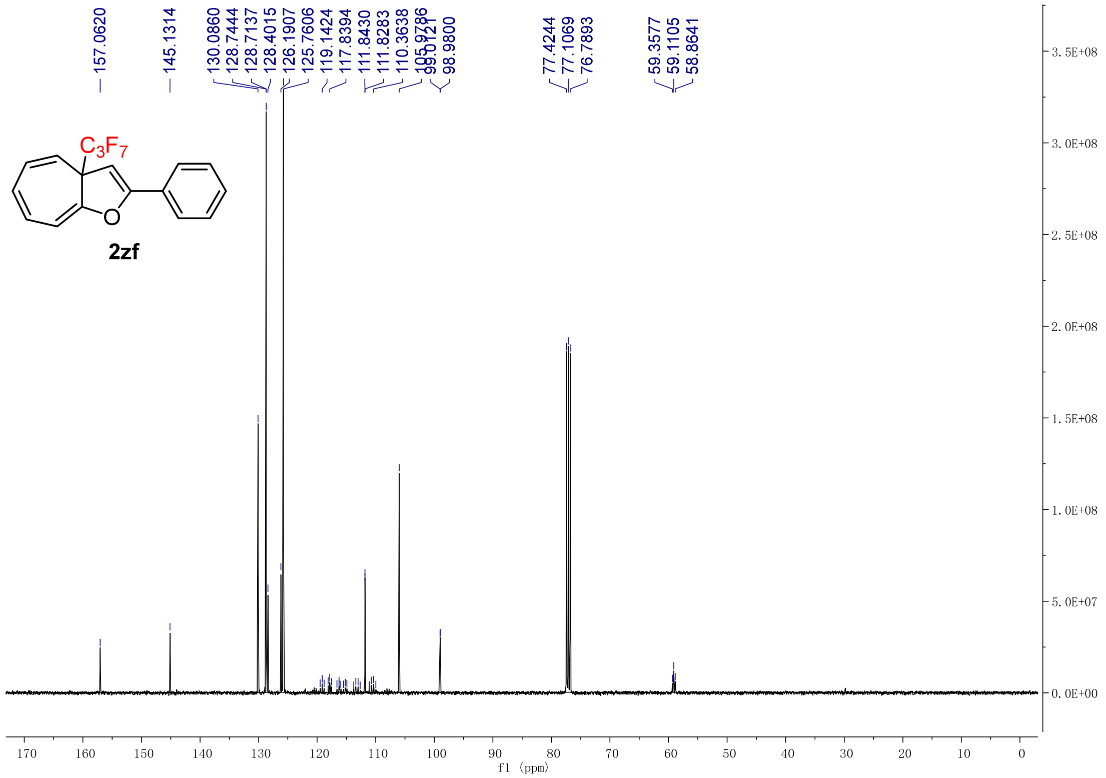

^{19}F NMR (376 MHz, $CDCl_3$) of compound **2zf**

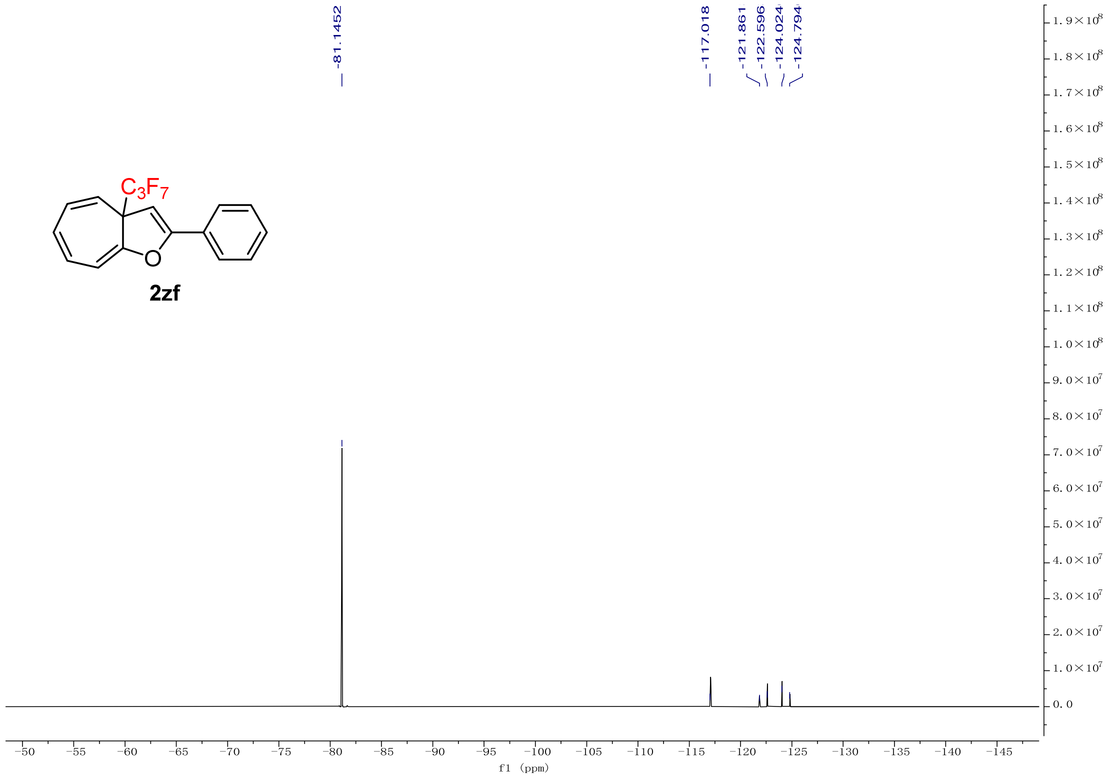

^{1}H NMR (600 MHz, $CDCl_3$) of compound **2zg**

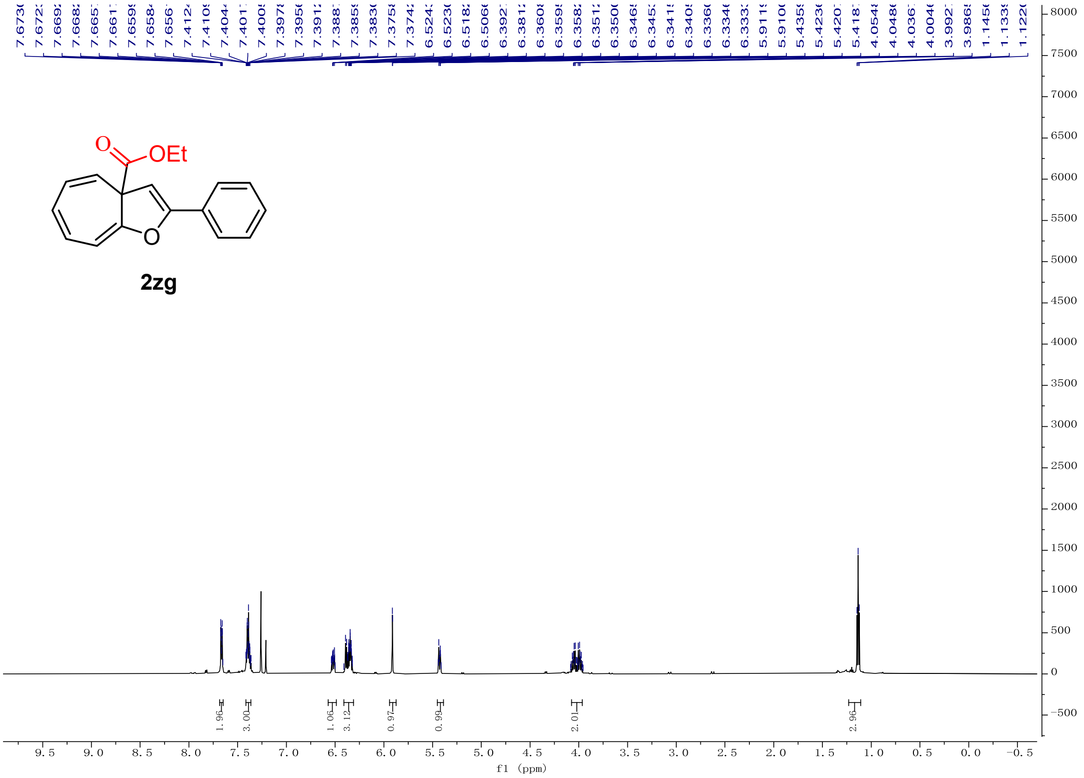

^{13}C NMR (151 MHz, $CDCl_3$) of compound **2zg**

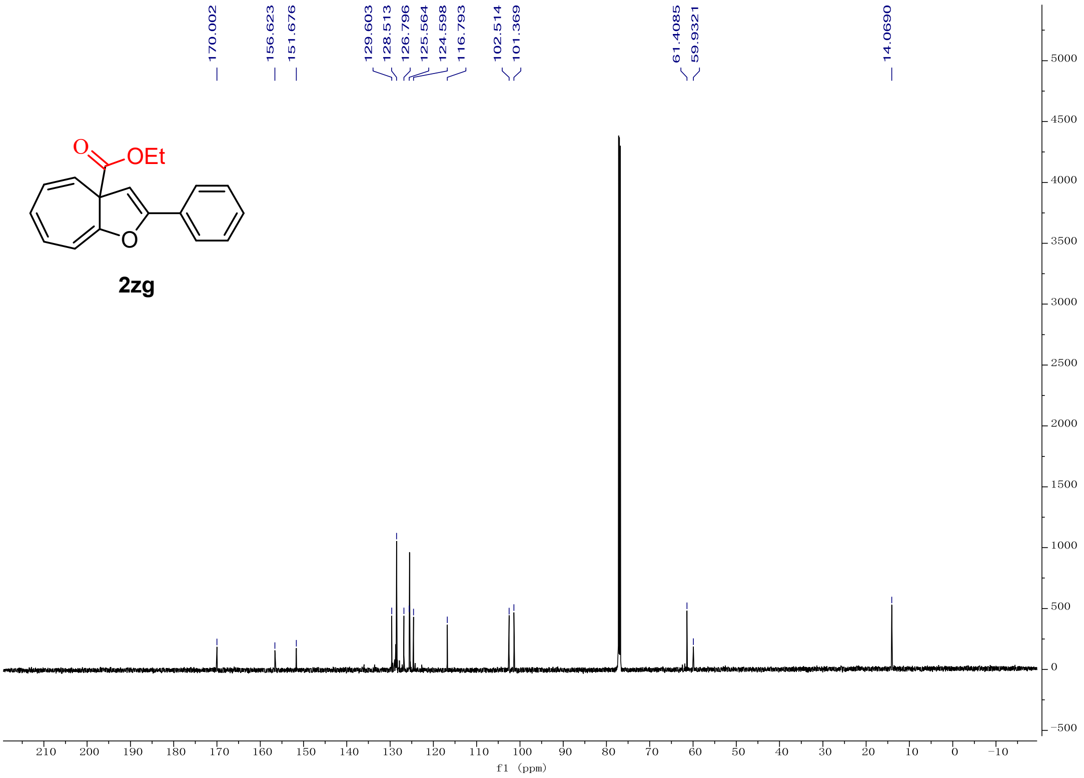

^{1}H NMR (400 MHz, $CDCl_3$) of compound **2zh**

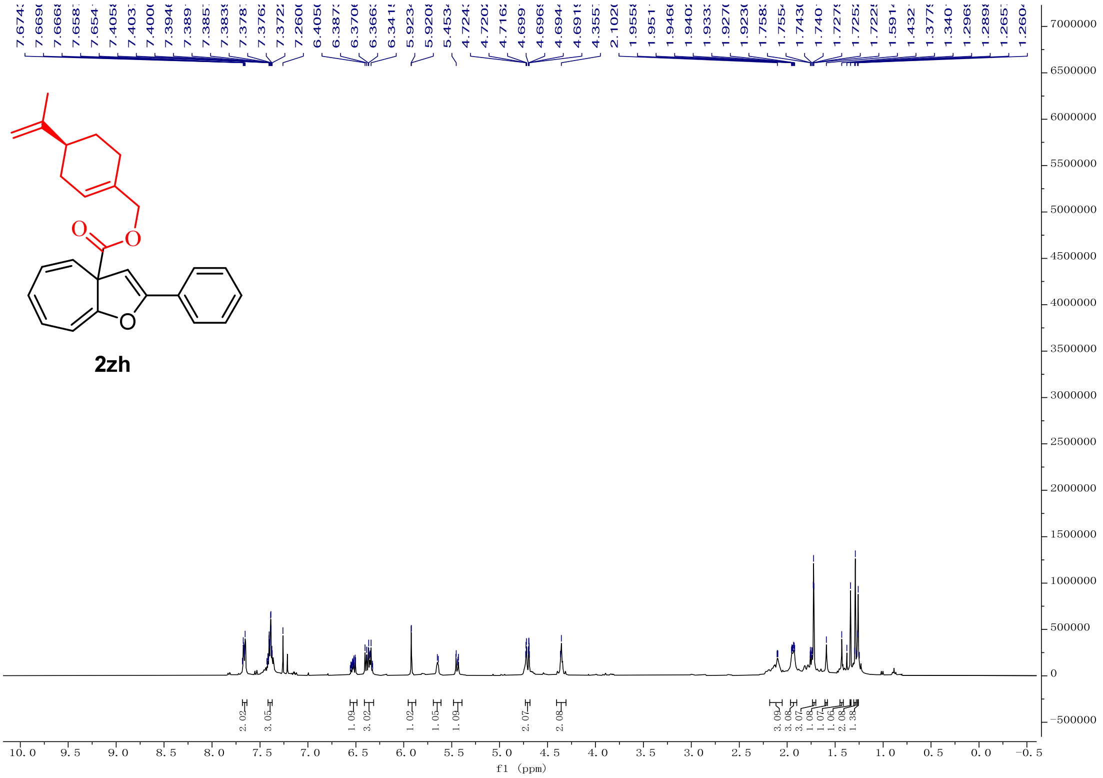

^{13}C NMR (151 MHz, CD_2Cl_2) of compound **2zh**

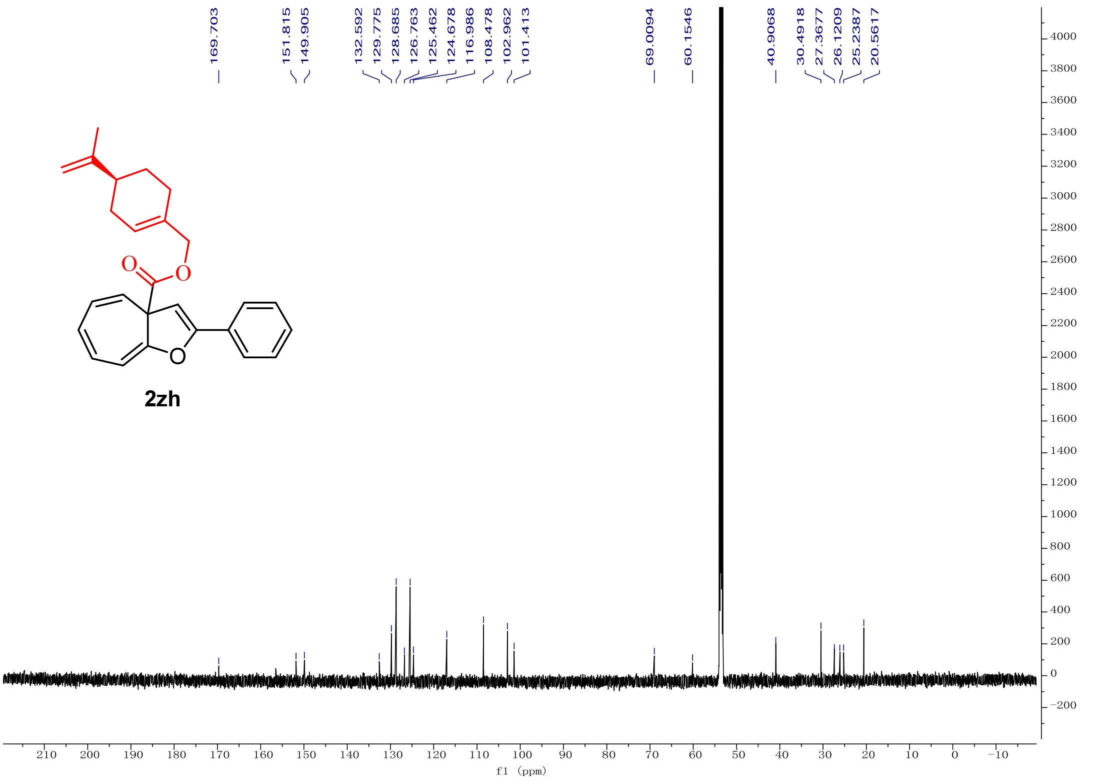

^{1}H NMR (400 MHz, $CDCl_3$) of compound **2zi**

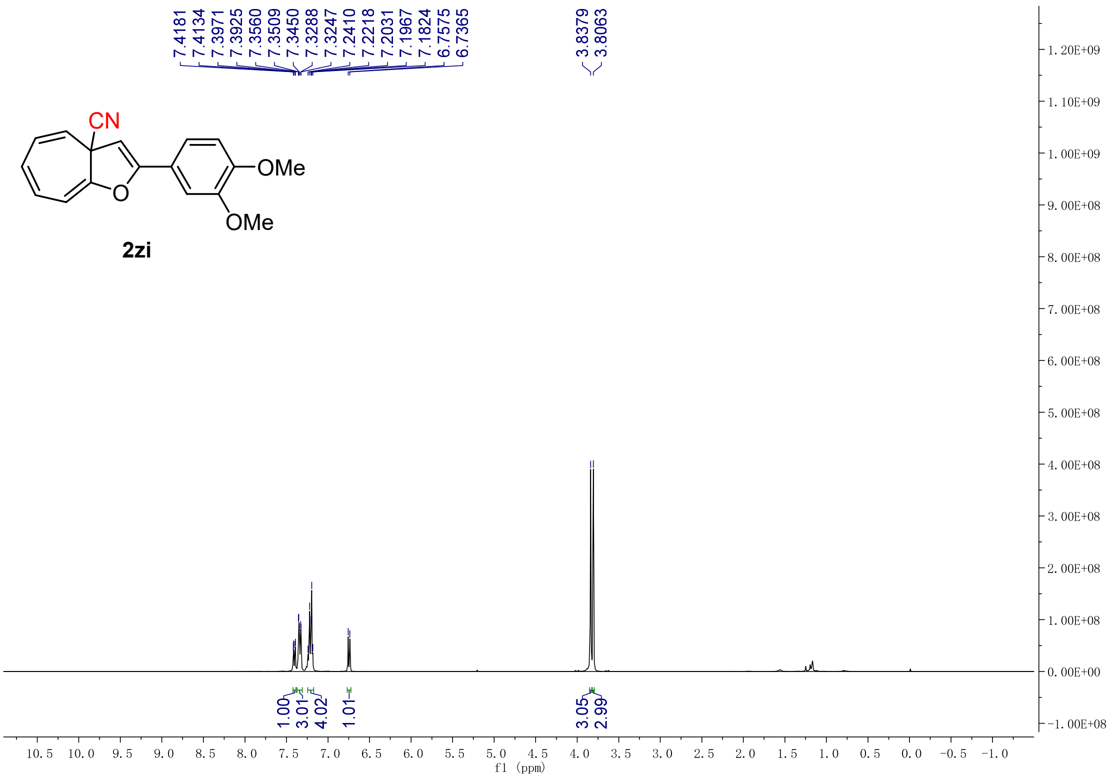

^{13}C NMR (151 MHz, $CDCl_3$) of compound **2zi**

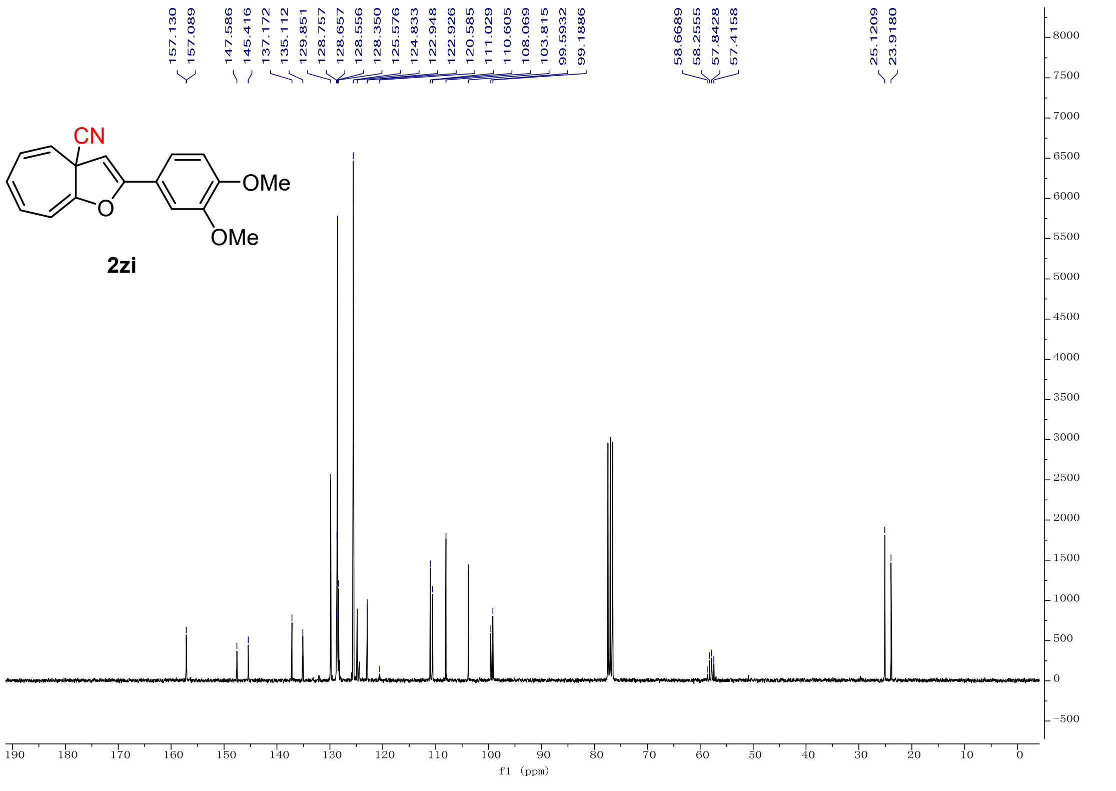

^{1}H NMR (300 MHz, $CDCl_3$) of compound **2zj**

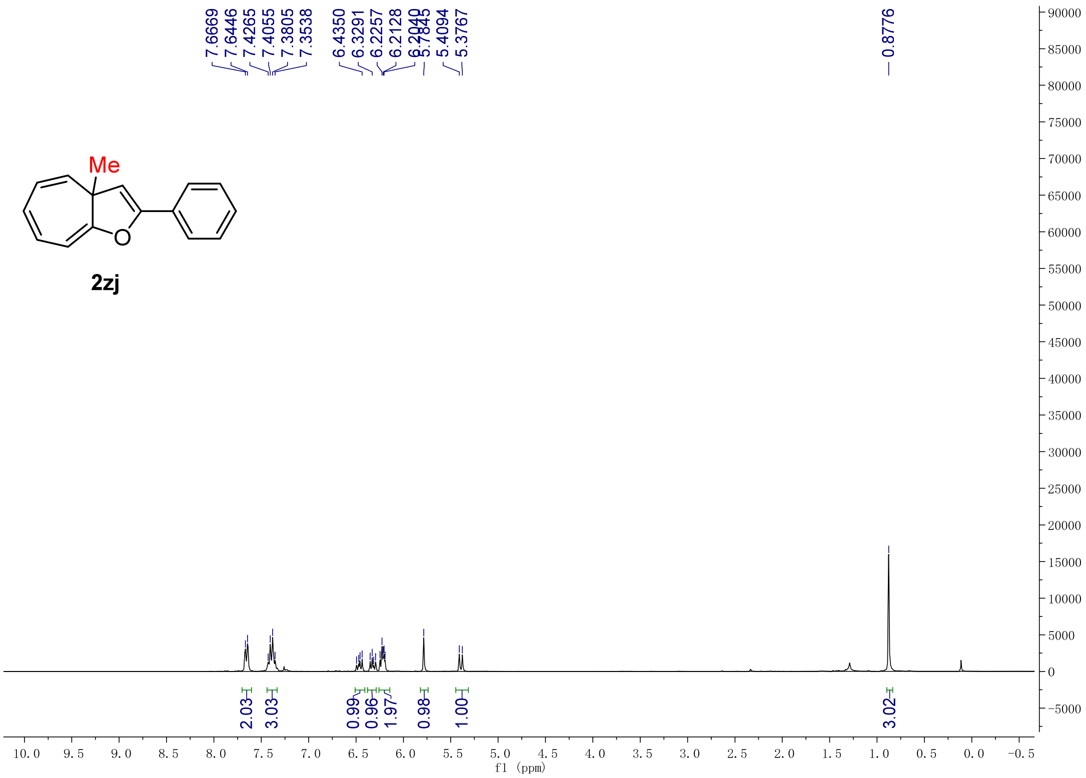

^{13}C NMR (75 MHz, $CDCl_3$) of compound **2zj**

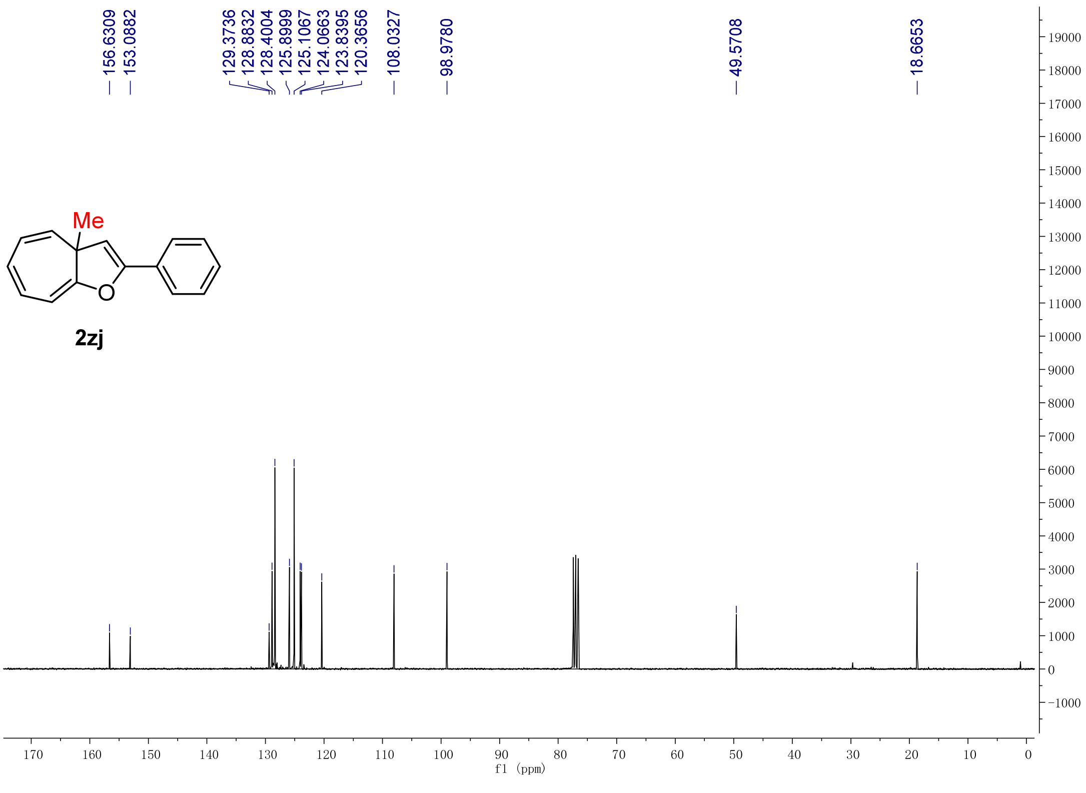

^{1}H NMR (400 MHz, $CDCl_3$) of compound **2zk**

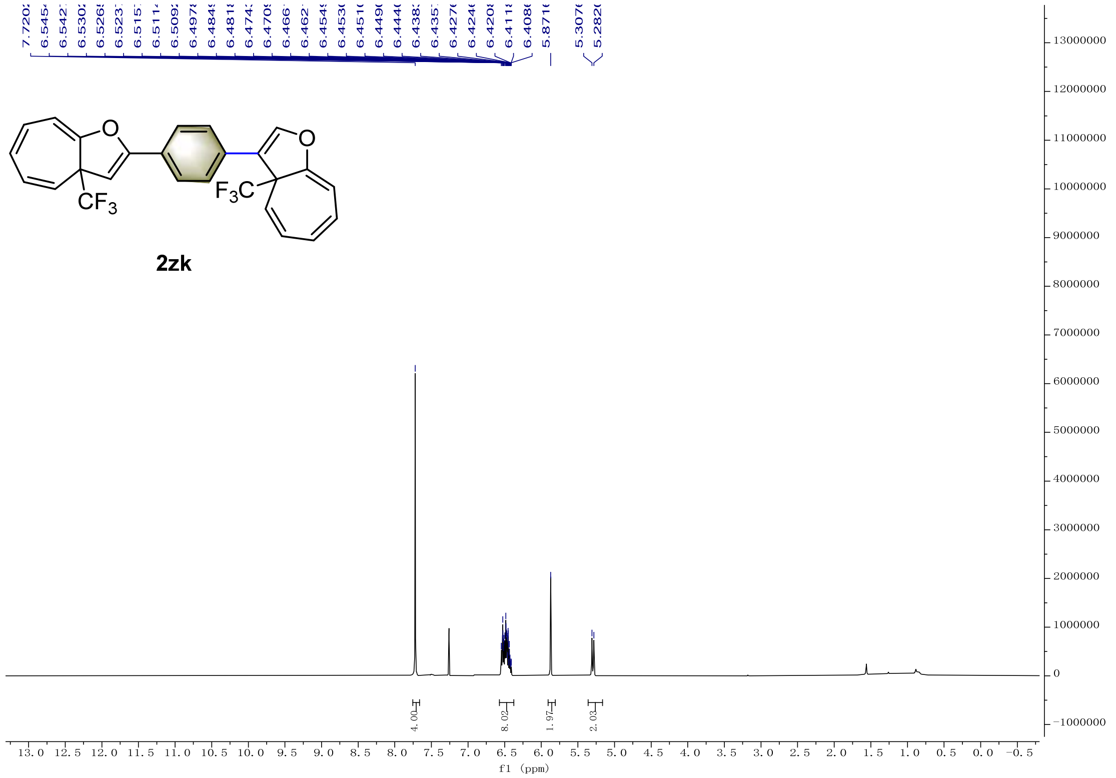

^{13}C NMR (101 MHz, $CDCl_3$) of compound **2zk**

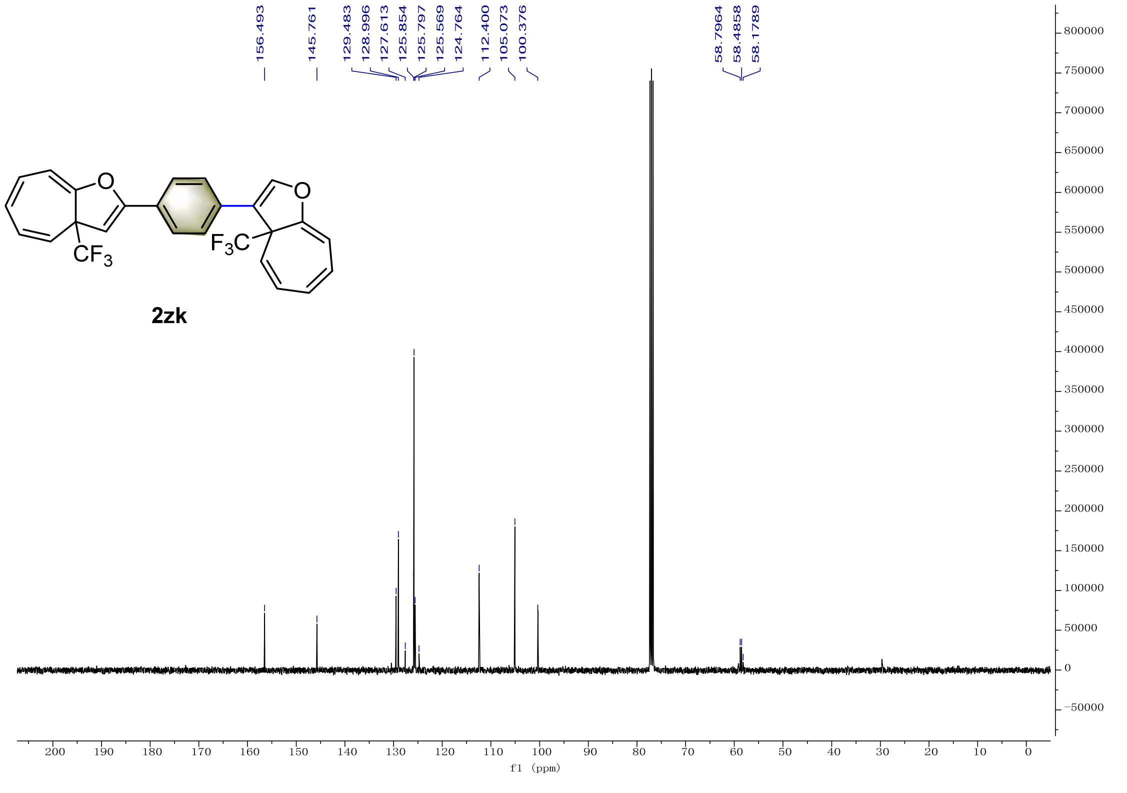

^{19}F NMR (376 MHz, $CDCl_3$) of compound **2zk**

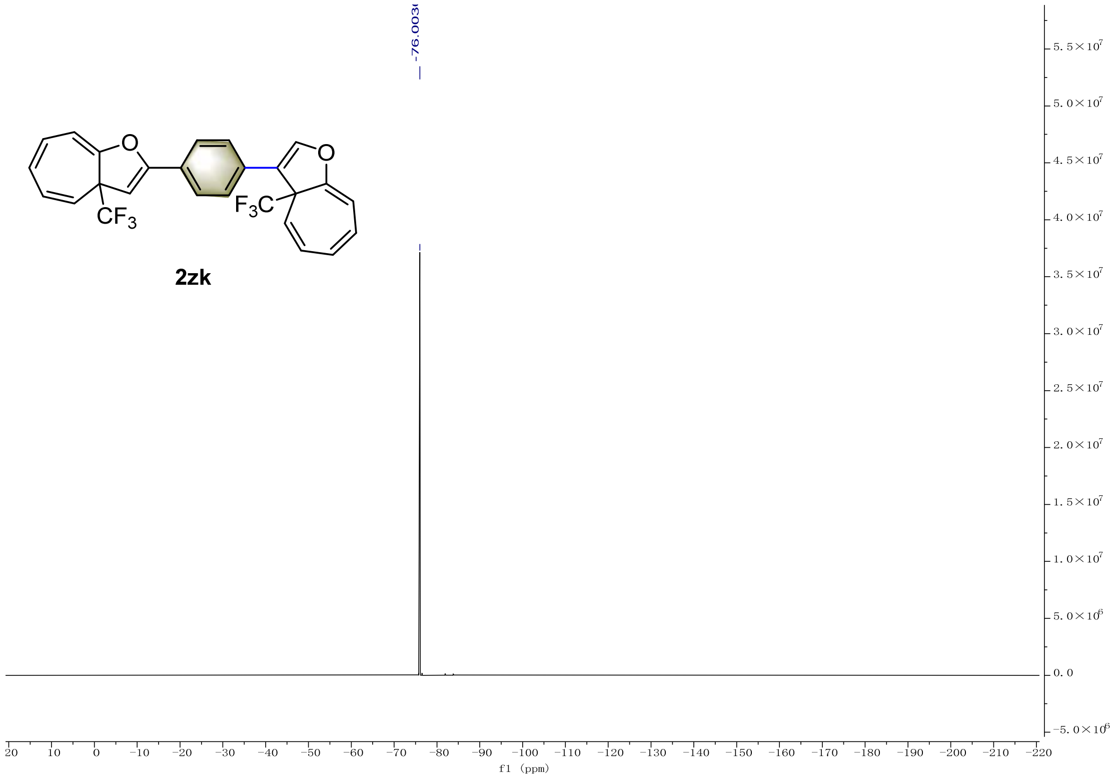

^{1}H NMR (400 MHz, $CDCl_3$) of compound **2zl**

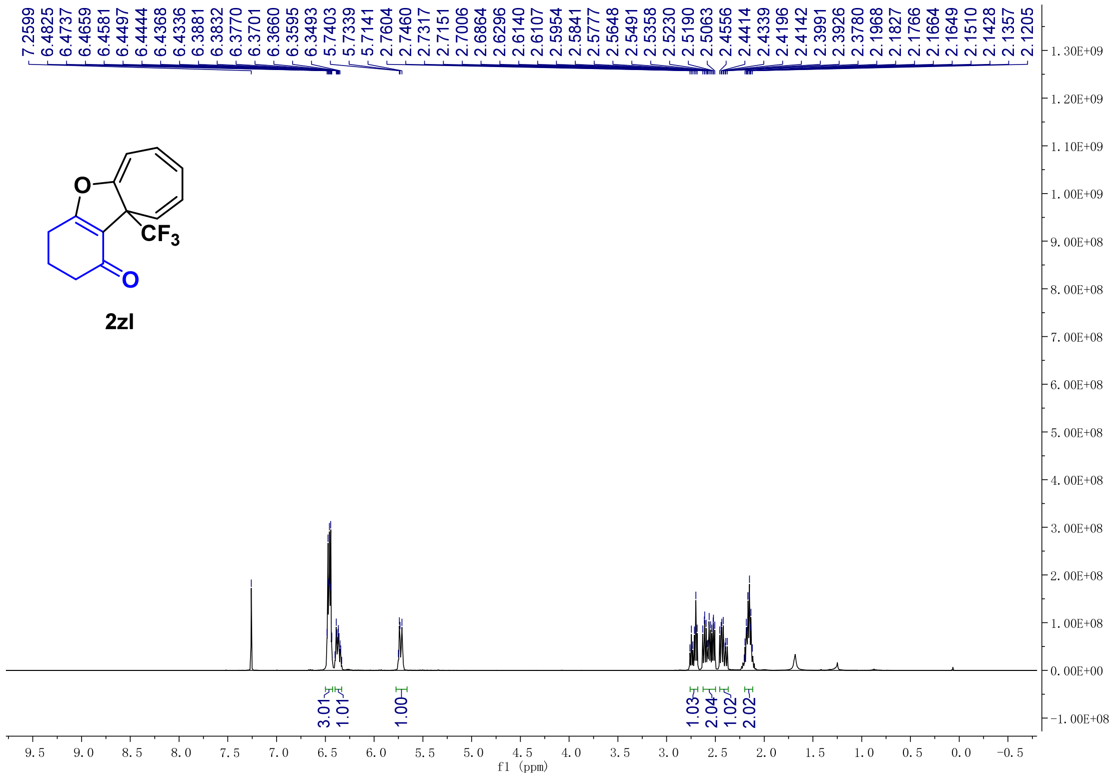

^{13}C NMR (75 MHz, $CDCl_3$) of compound **2zl**

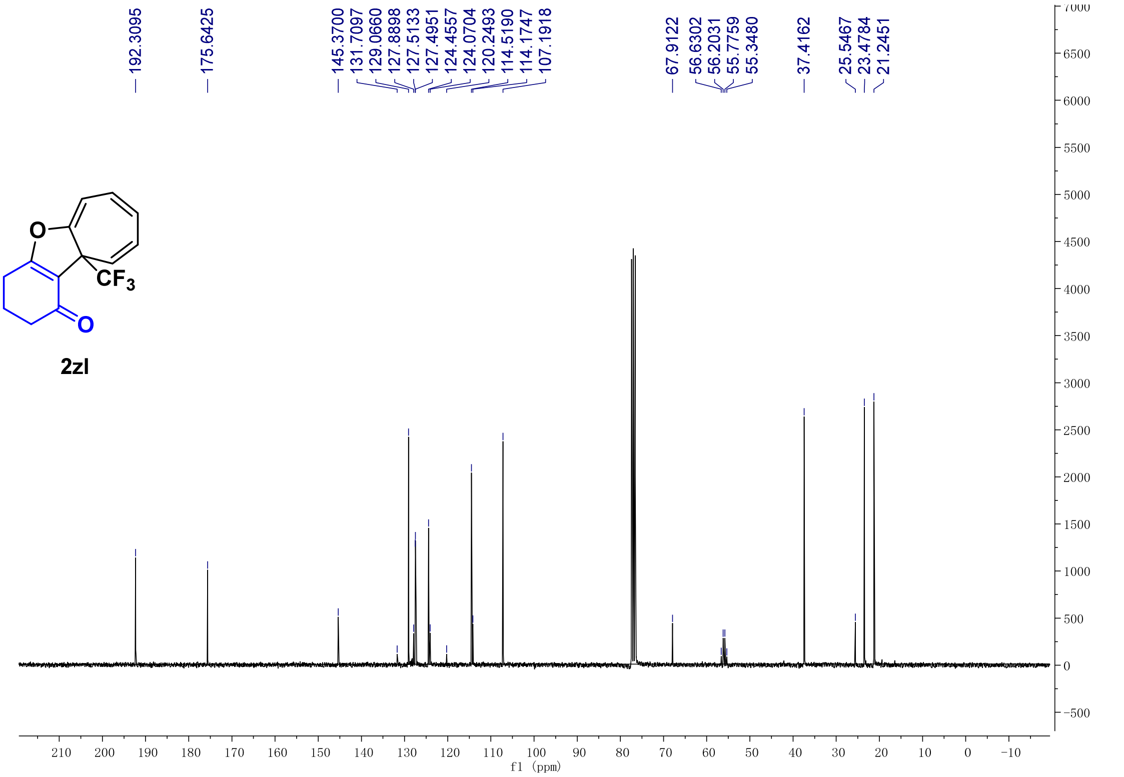

^{19}F NMR (376 MHz, $CDCl_3$) of compound **2zl**

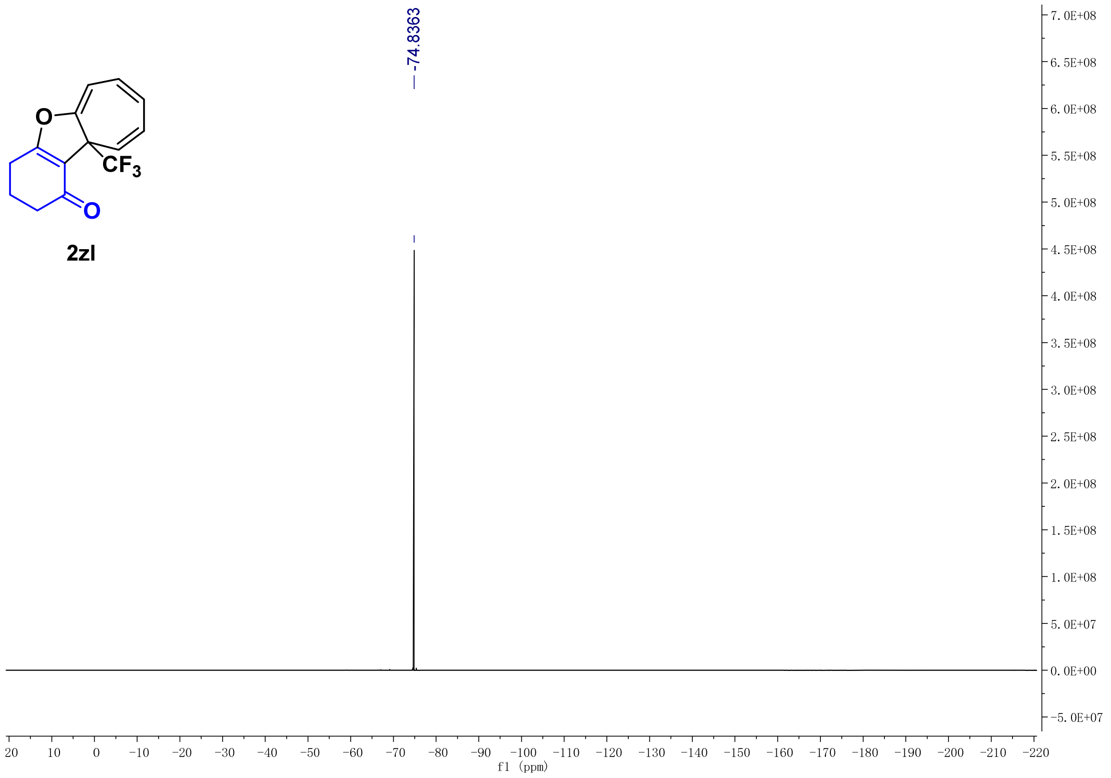

^{1}H NMR (600 MHz, $CDCl_3$) of compound **2zm**

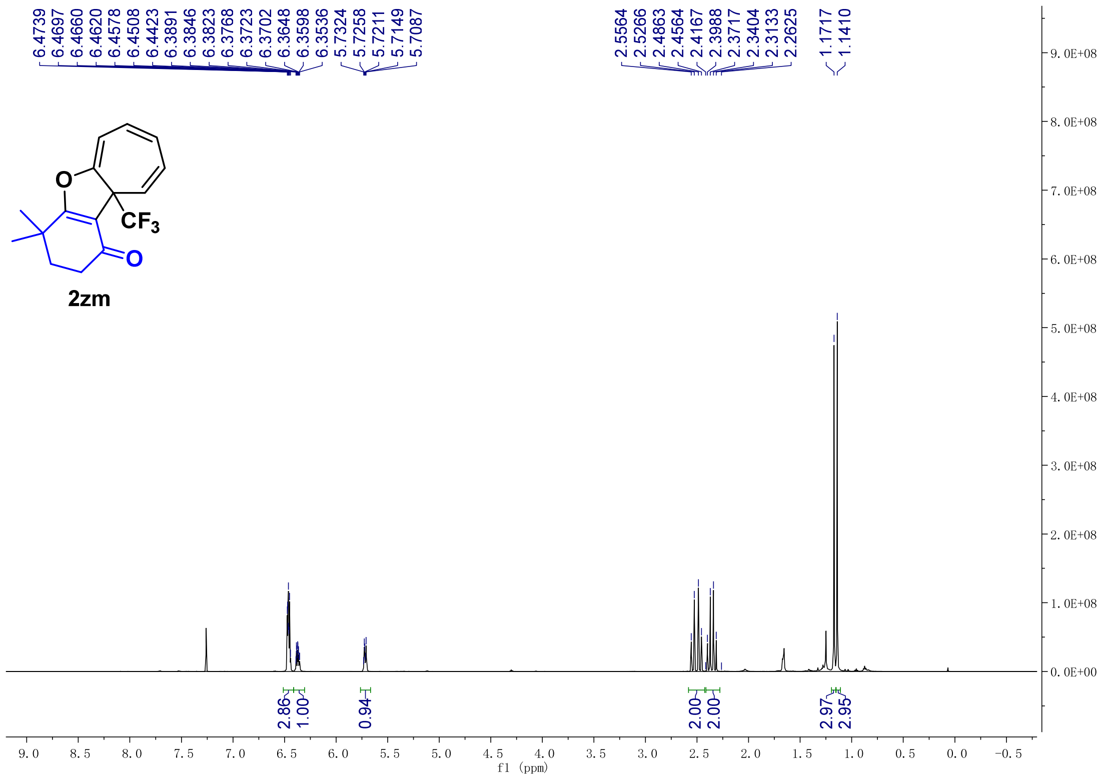

^{13}C NMR (101 MHz, $CDCl_3$) of compound **2zm**

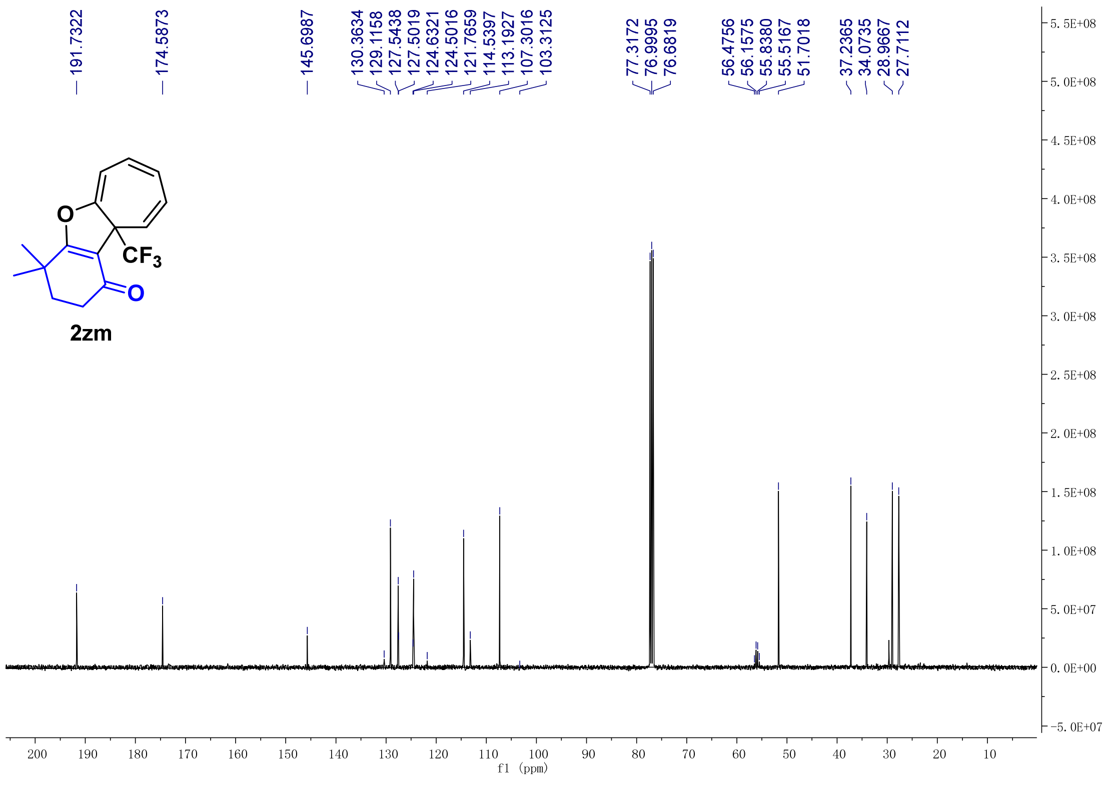

^{19}F NMR (376 MHz, $CDCl_3$)

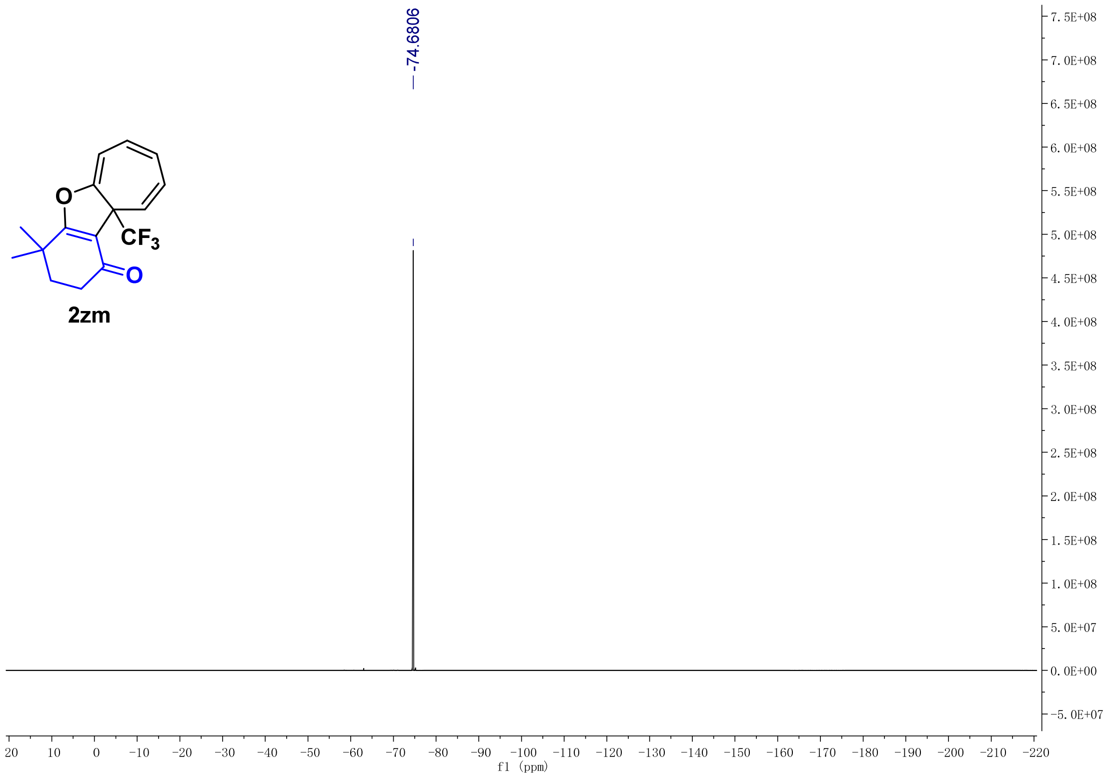

^{1}H NMR (400 MHz, $CDCl_3$) of compound **2zn**

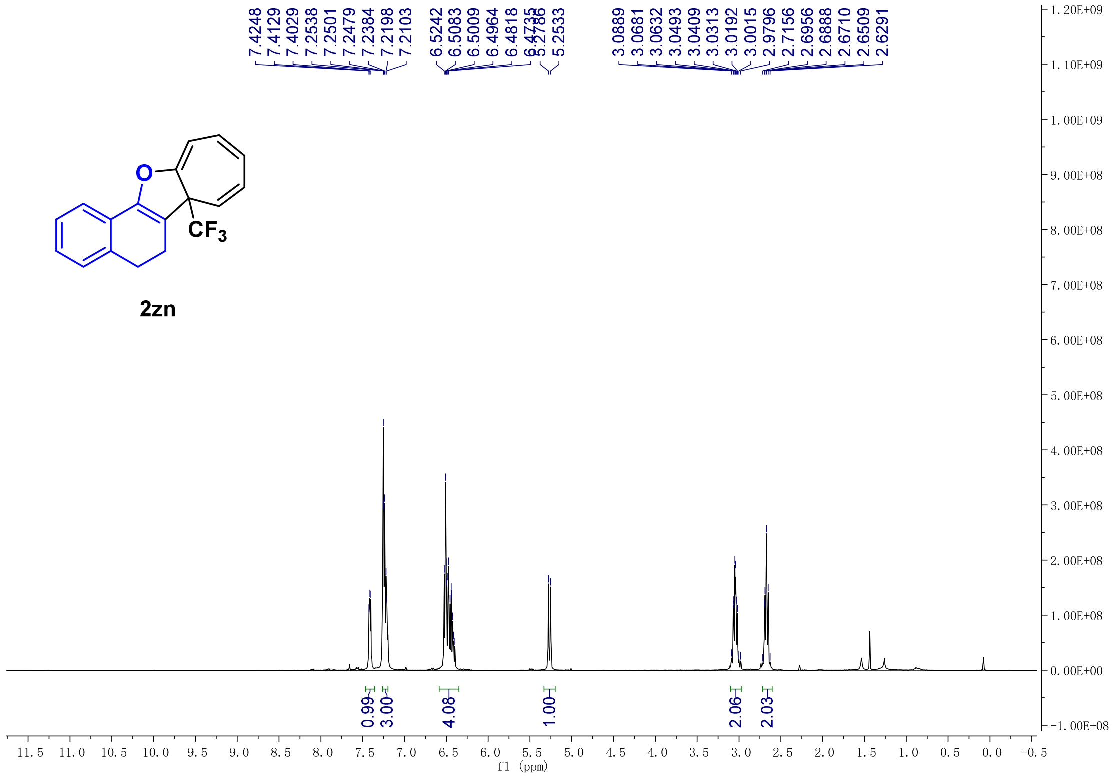

^{13}C NMR (101 MHz, $CDCl_3$) of compound **2zn**

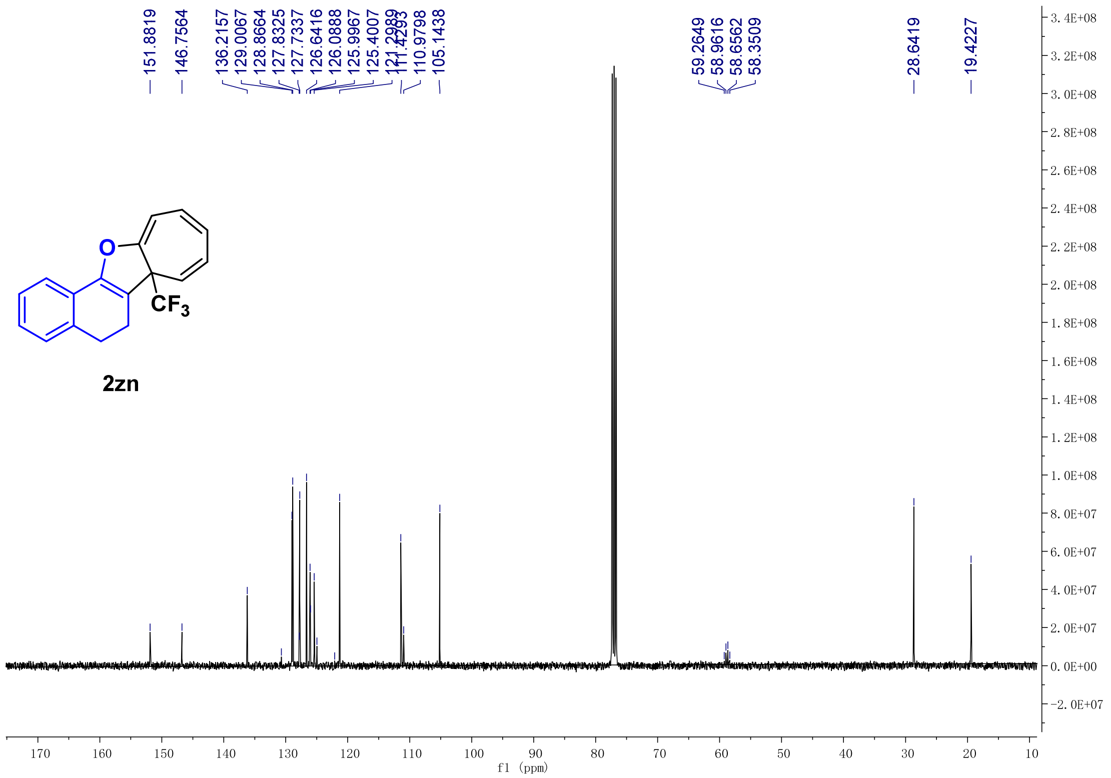

^{19}F NMR (376 MHz, $CDCl_3$) of compound **2zn**

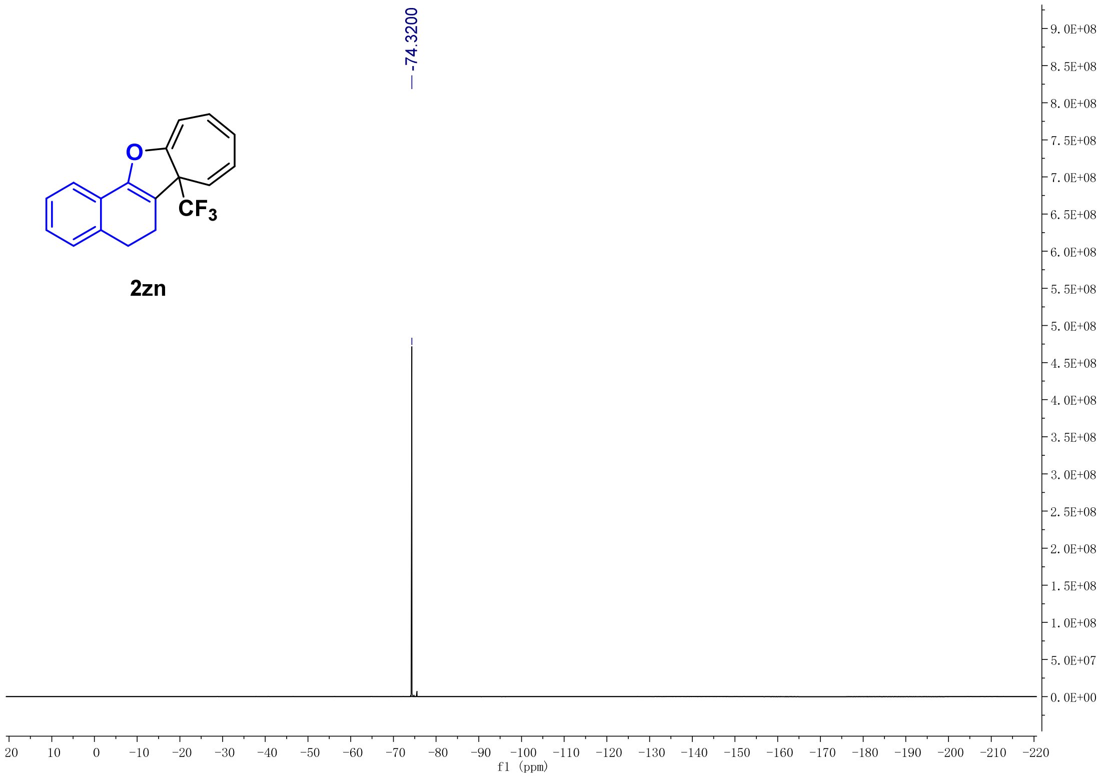

^{1}H NMR (400 MHz, $CDCl_3$) of compound **4a**

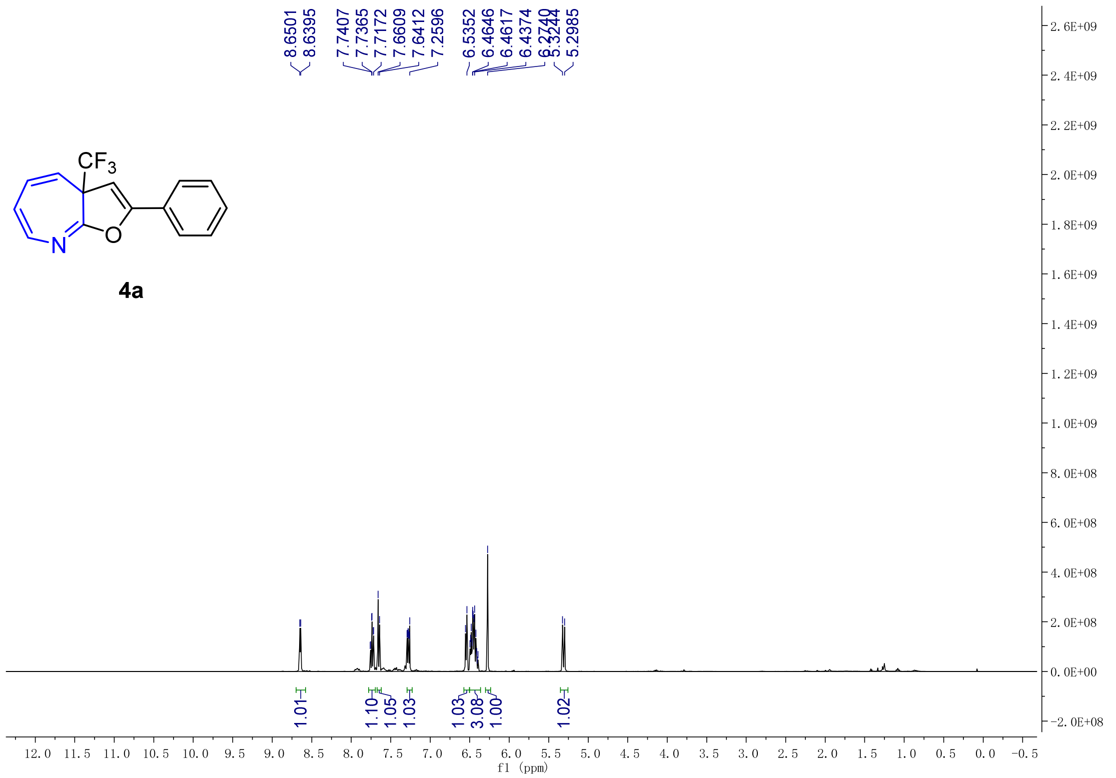

^{13}C NMR (101 MHz, $CDCl_3$) of compound **4a**

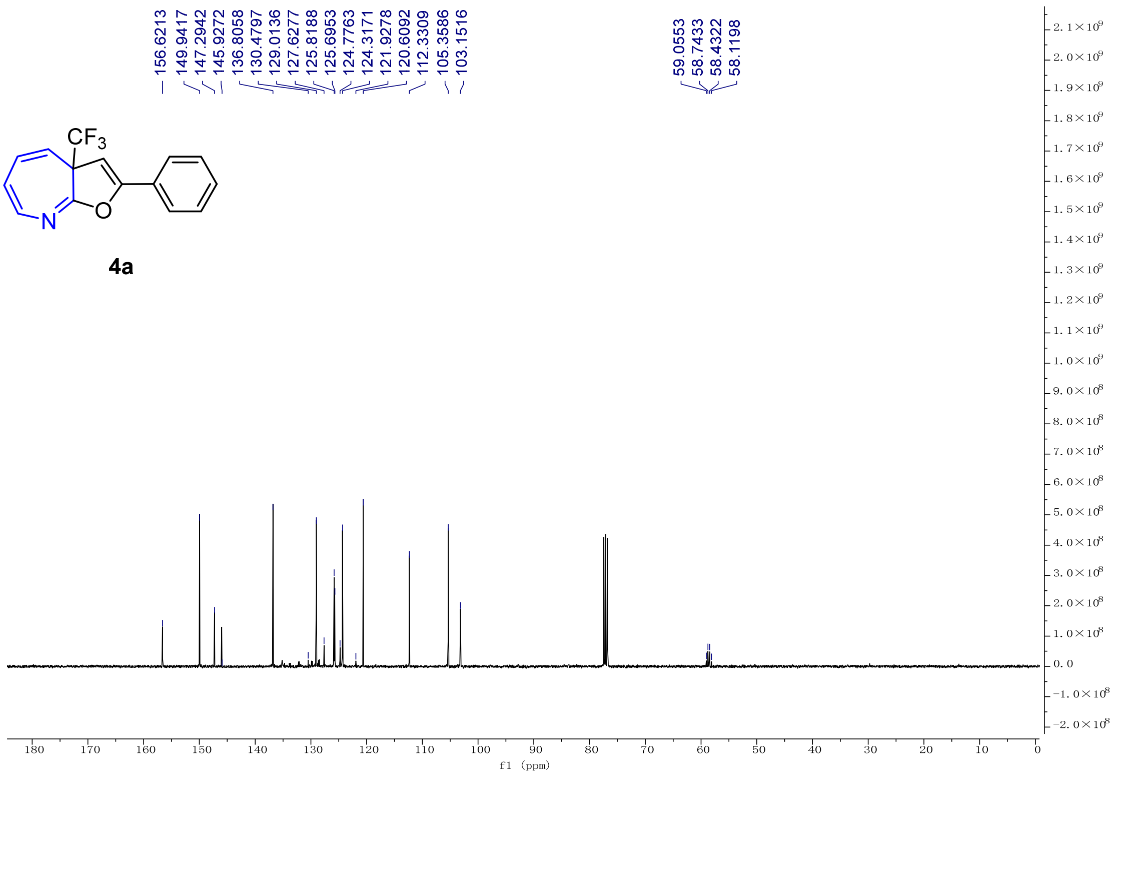

^{19}F NMR (376 MHz, $CDCl_3$) of compound **4a**

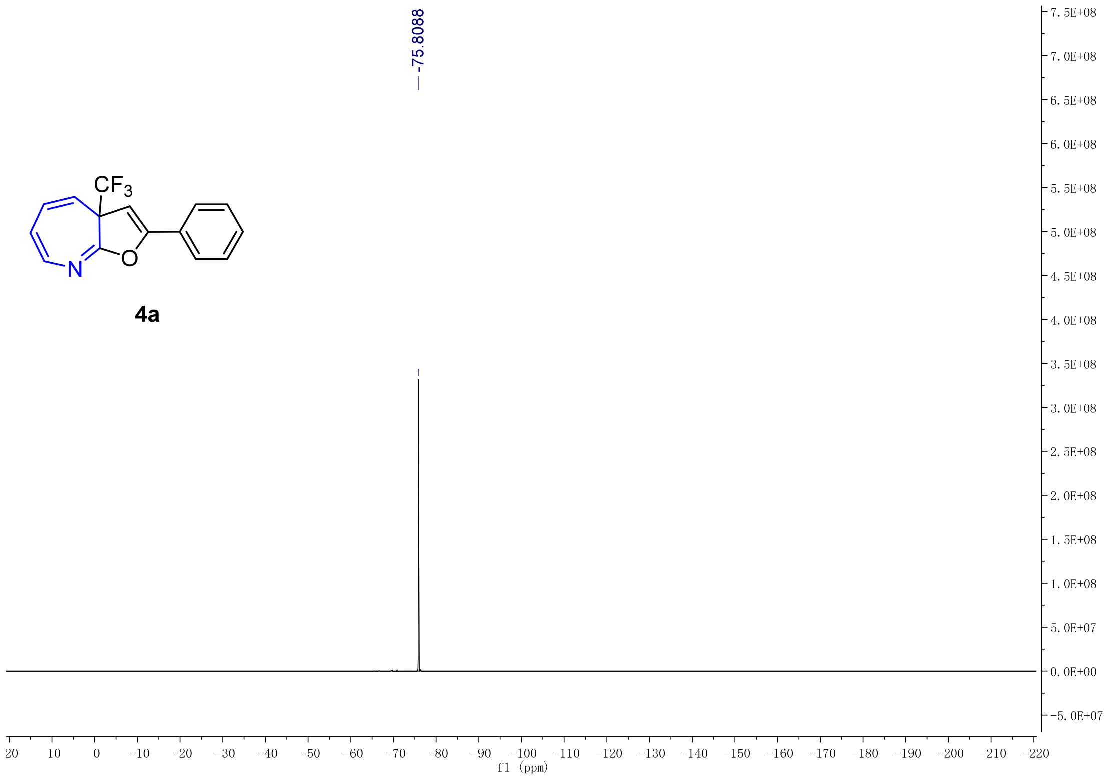

^{1}H NMR (600 MHz, $CDCl_3$) of compound **4b**

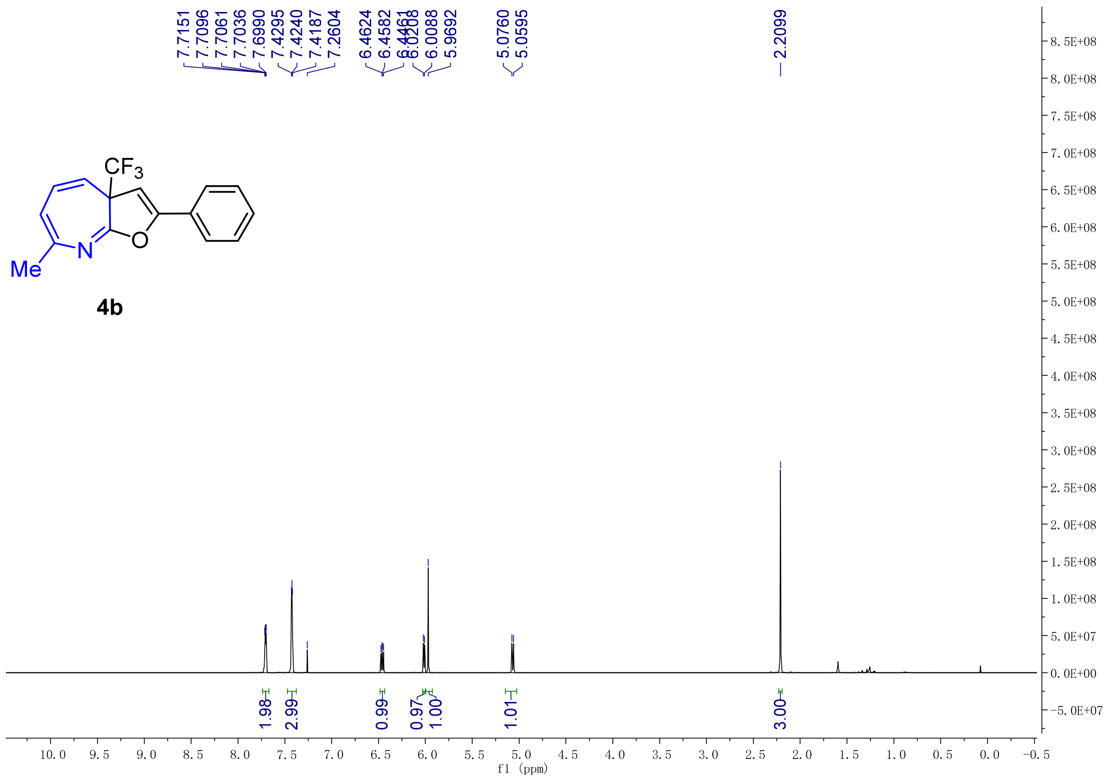

^{13}C NMR (151 MHz, $CDCl_3$) of compound **4b**

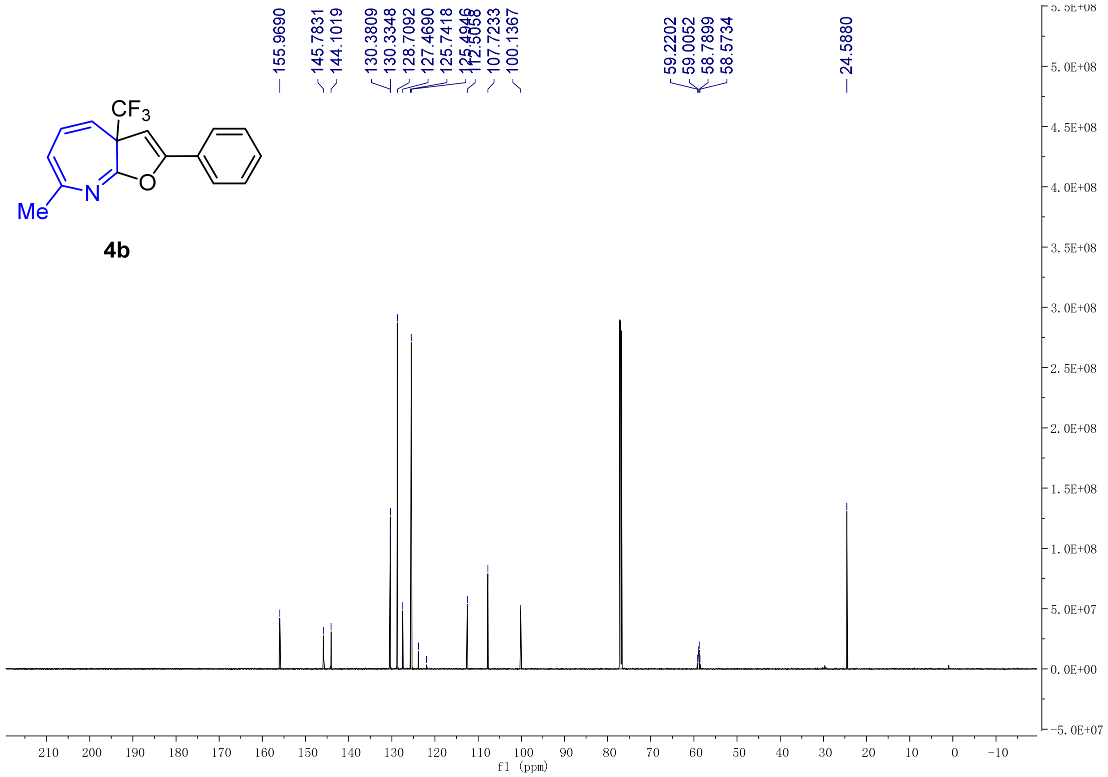

^{19}F NMR (565 MHz, $CDCl_3$) of compound **4b**

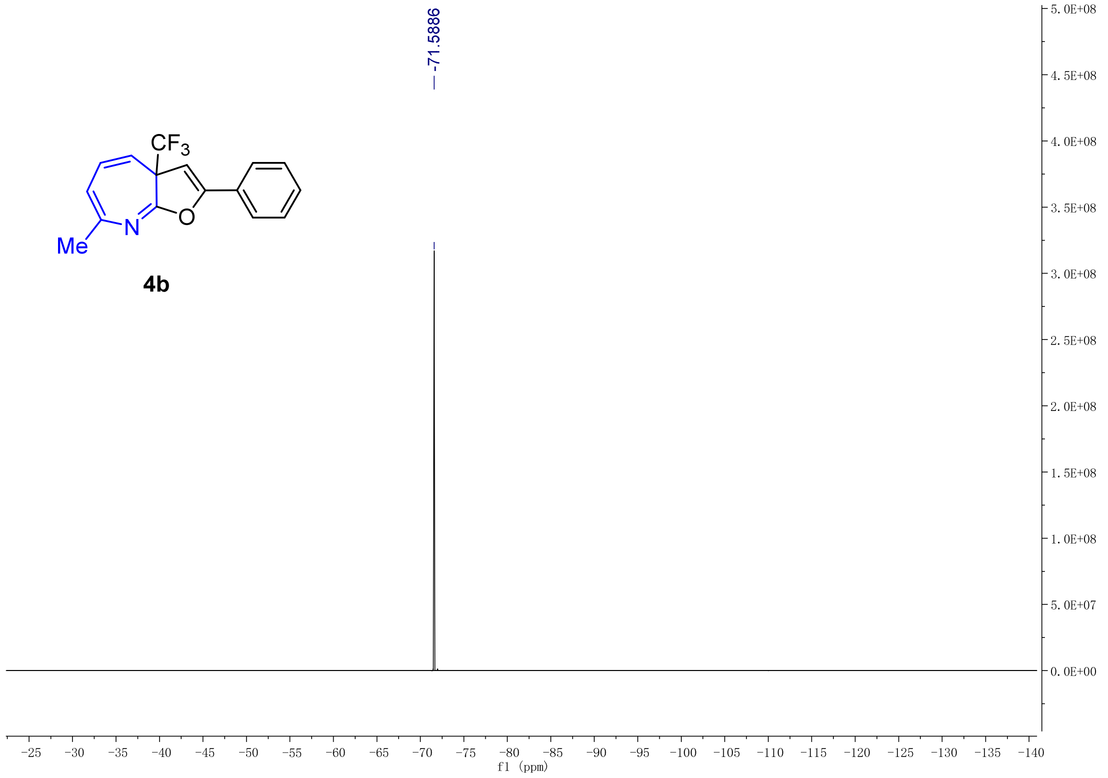

^{1}H NMR (600 MHz, CD_2Cl_2) of compound **4c**

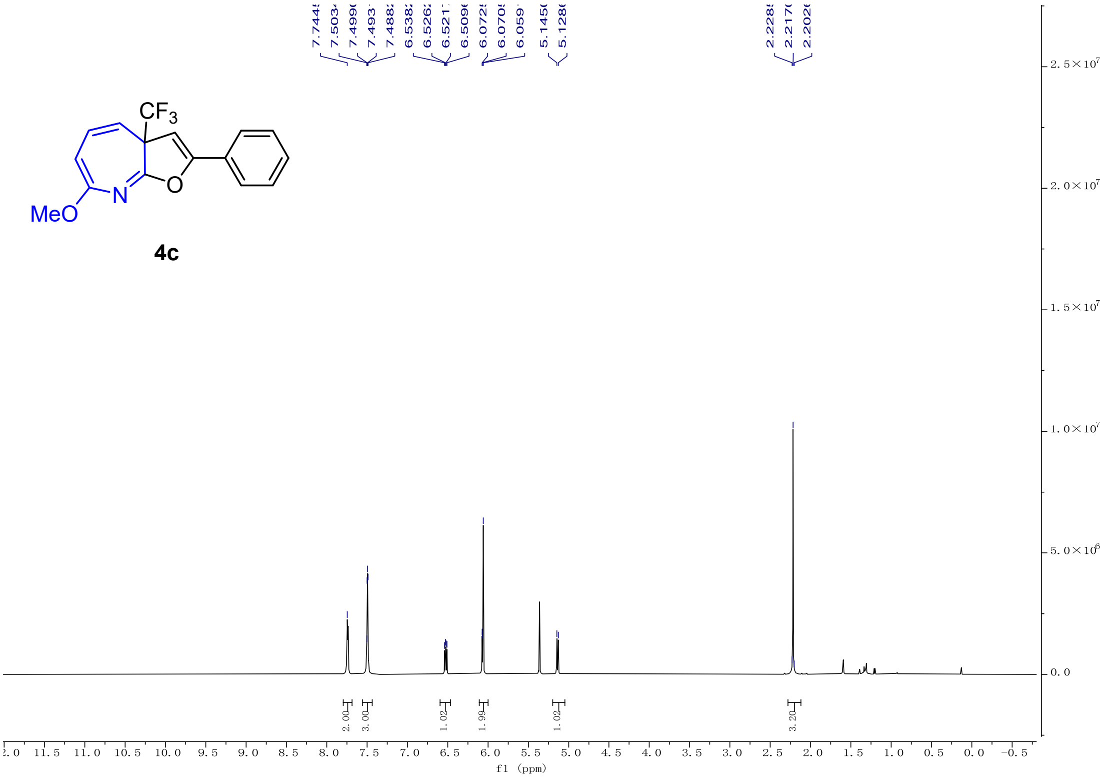

^{13}C NMR (151 MHz, CD_2Cl_2) of compound **4c**

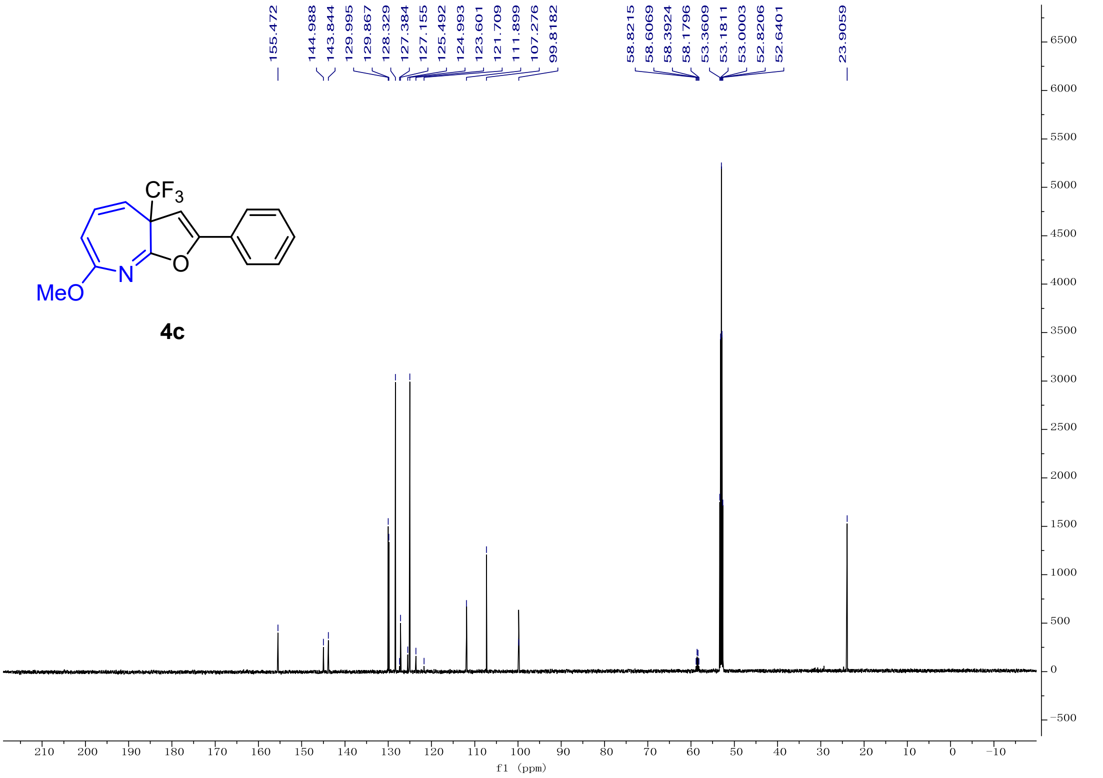

^{19}F NMR (565 MHz, CD_2Cl_2) δ -72.0 of compound **4c**

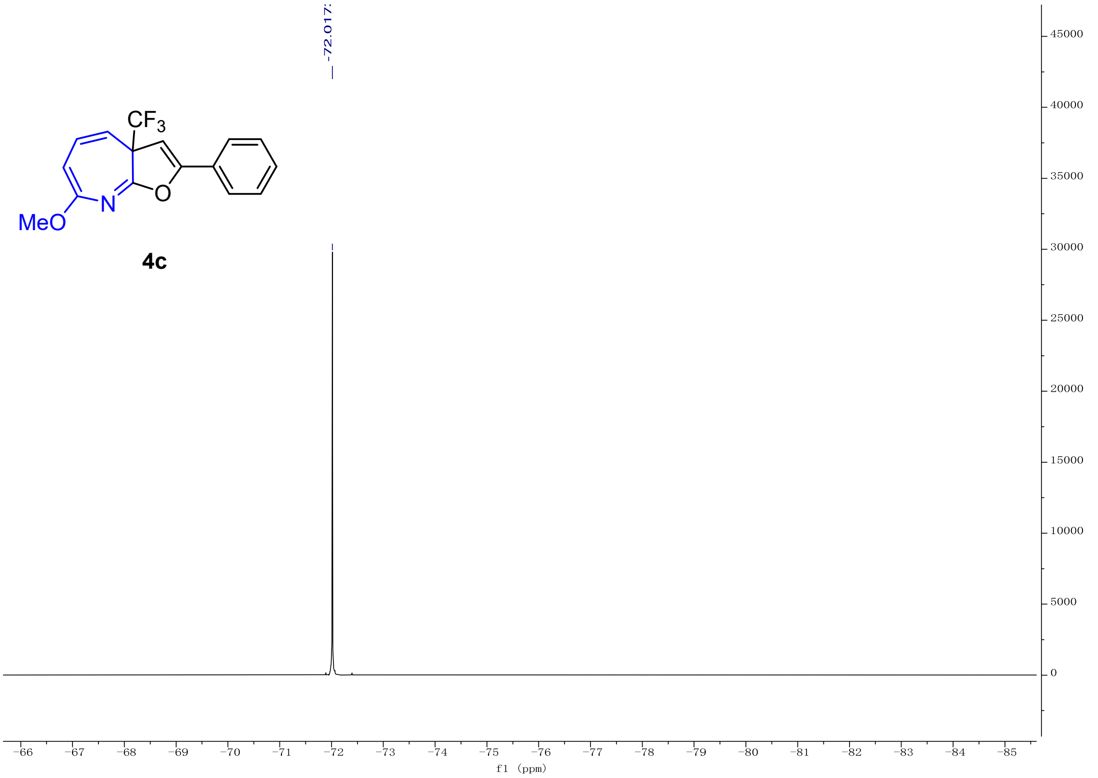

^{1}H NMR (400 MHz, $CDCl_3$) of compound **4d**

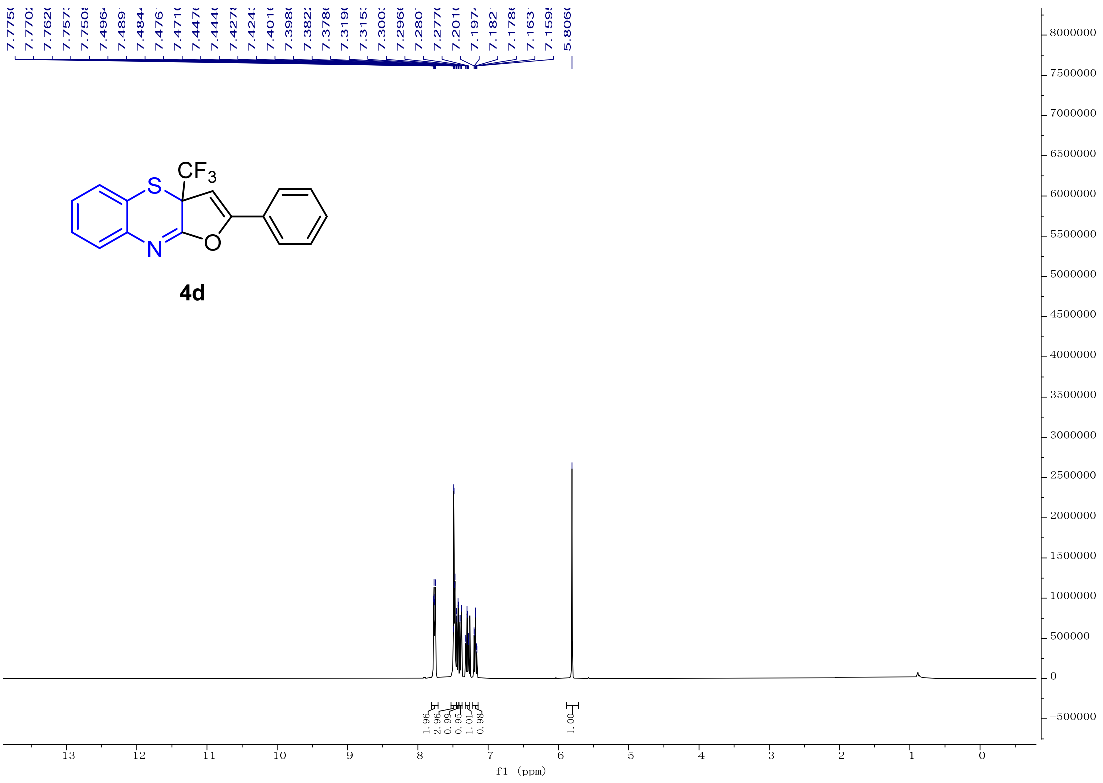

^{13}C NMR (101 MHz, $CDCl_3$) of compound **4d**

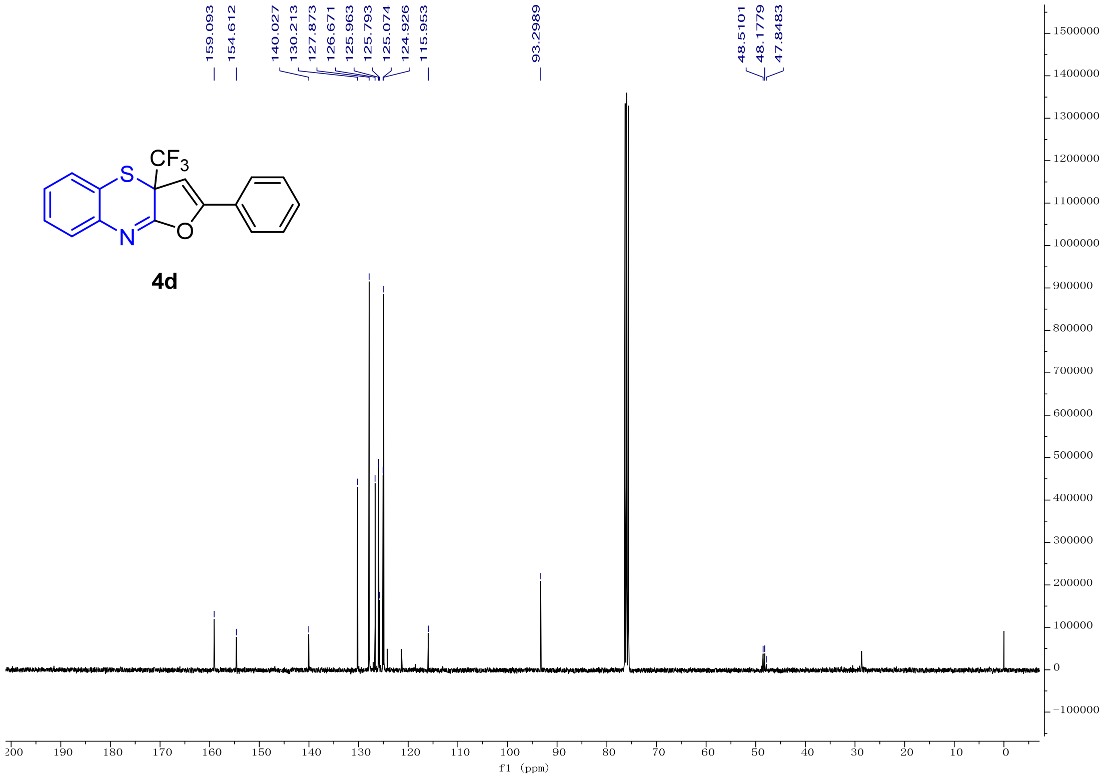

^{19}F NMR (376 MHz, $CDCl_3$) of compound **4d**

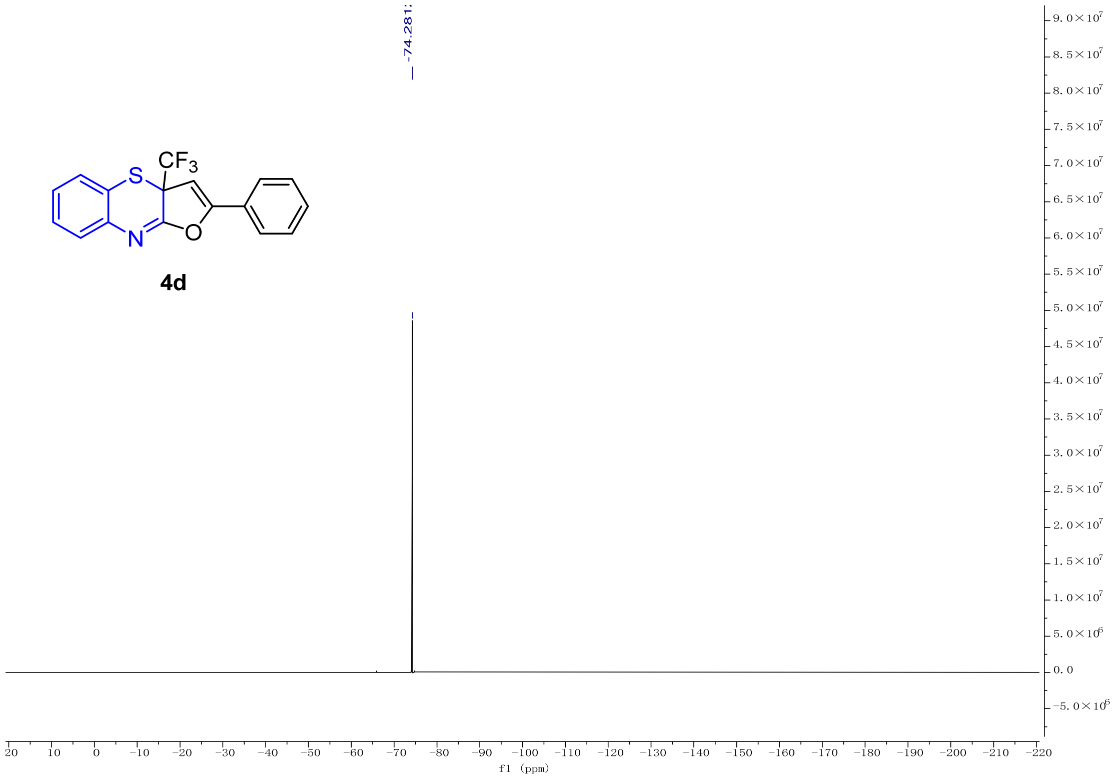

^{1}H NMR (600 MHz, $CDCl_3$) of compound **4e**

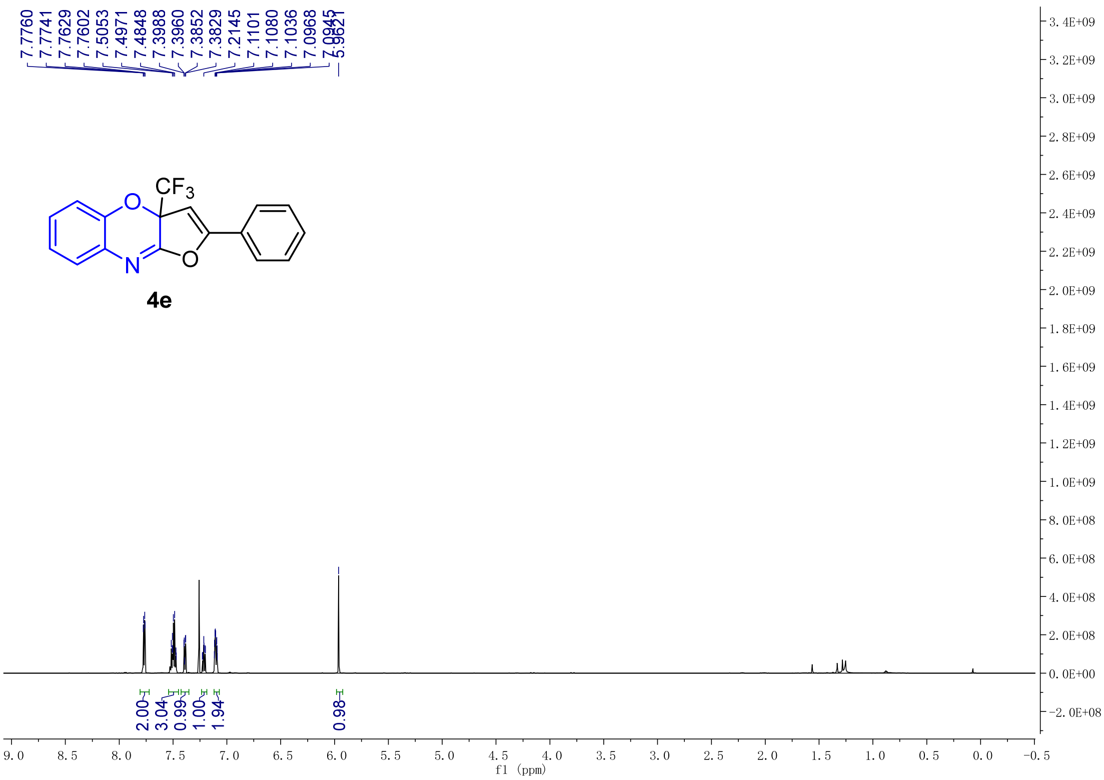

^{13}C NMR (151 MHz, $CDCl_3$) of compound **4e**

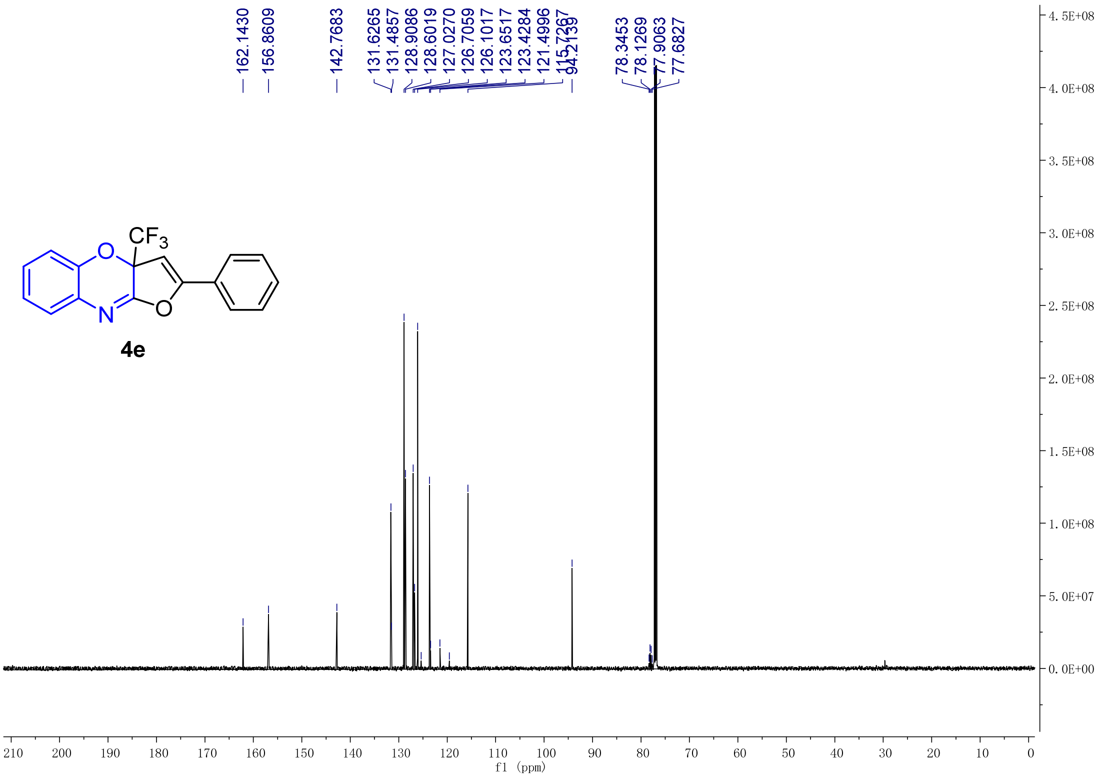

^{19}F NMR (565 MHz, $CDCl_3$) of compound **4e**

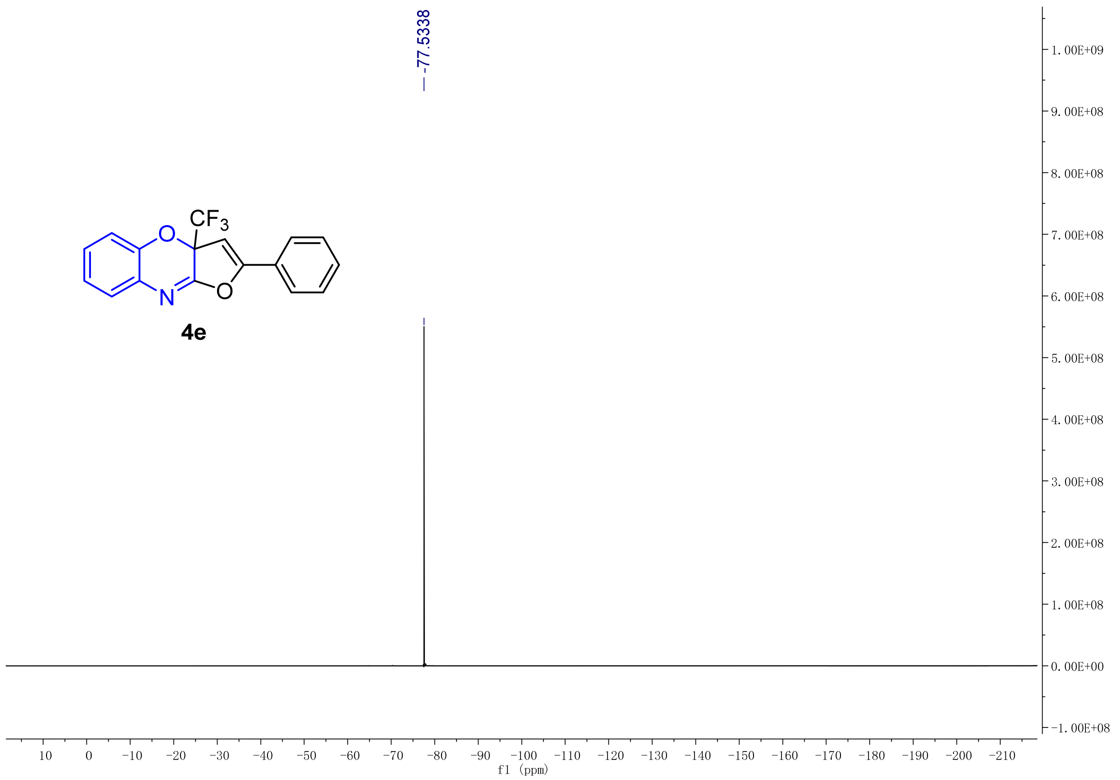

^{1}H NMR (600 MHz, $CDCl_3$) of compound **6a**

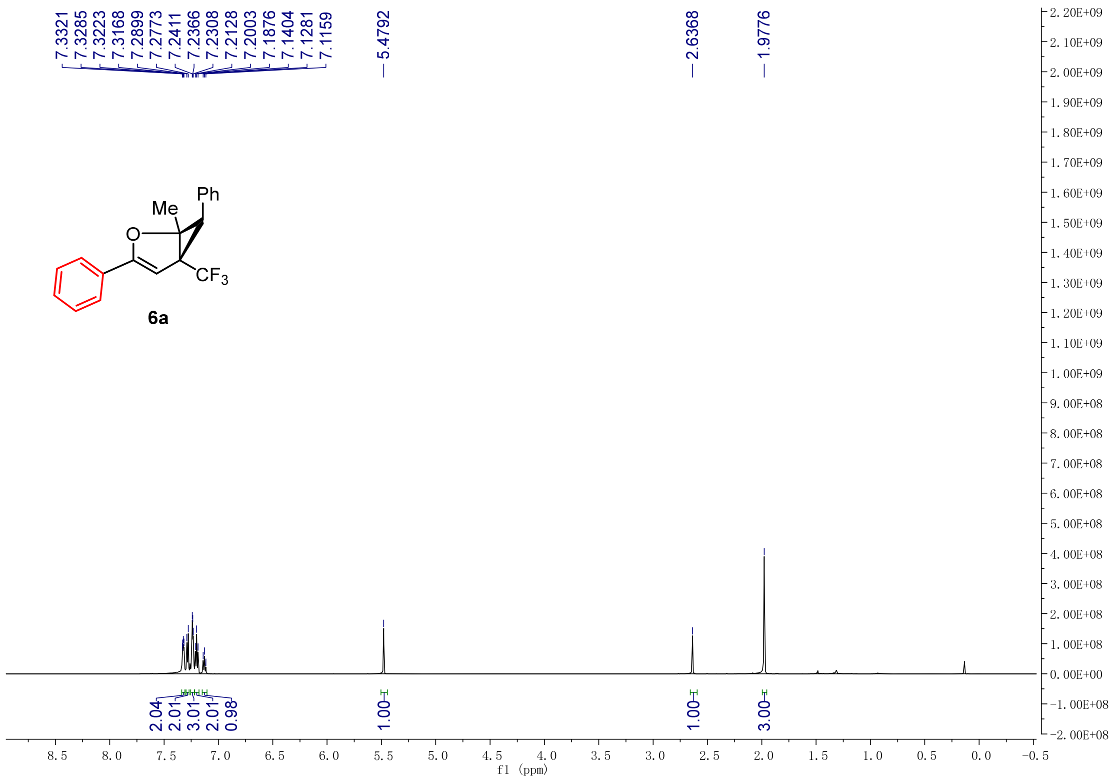

^{13}C NMR (151 MHz, $CDCl_3$) of compound **6a**

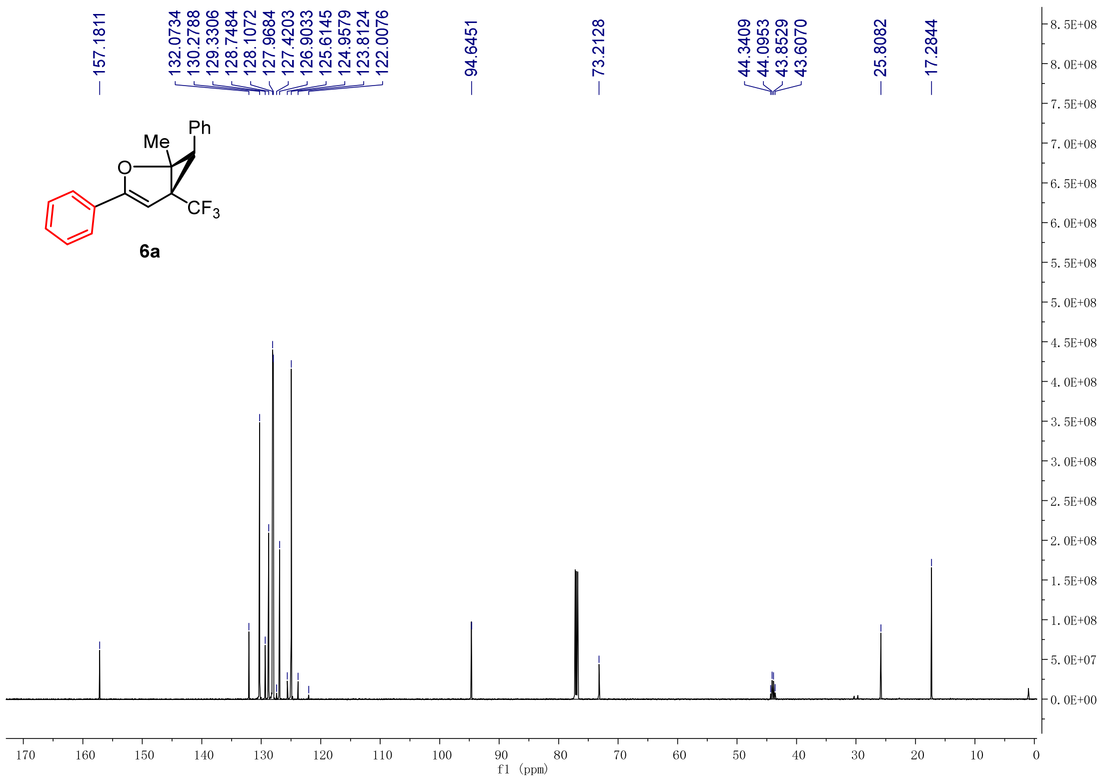

^{19}F NMR (565 MHz, $CDCl_3$) of compound **6a**

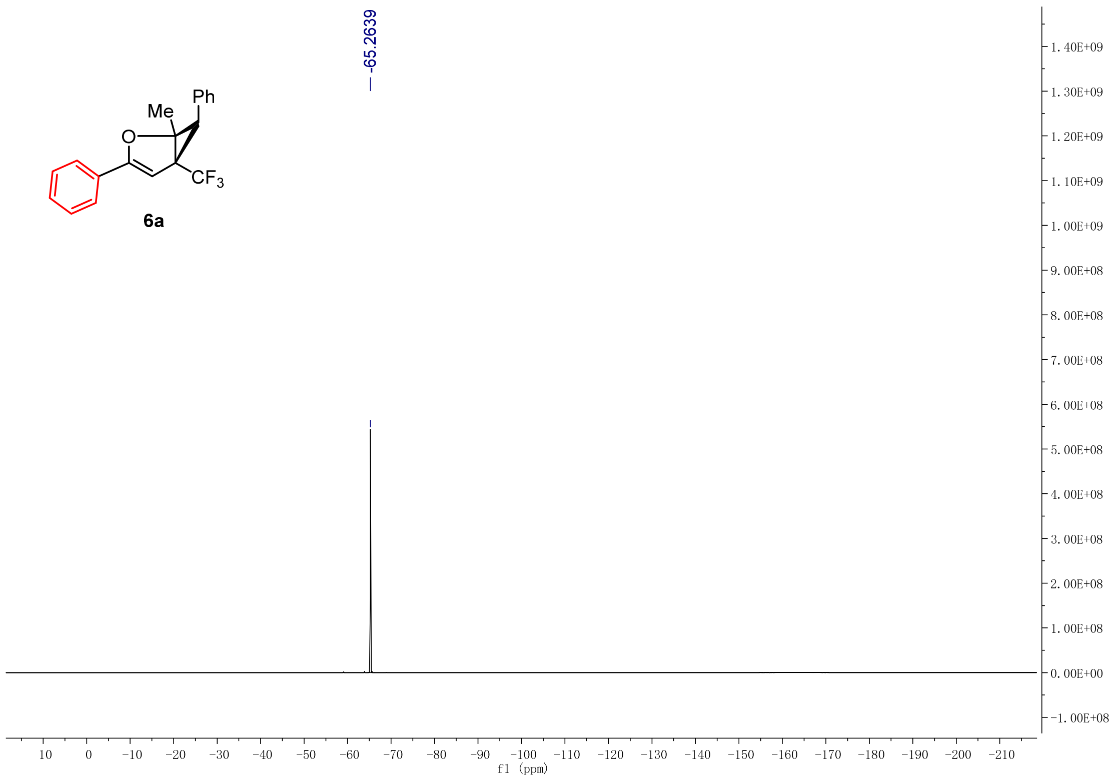

^{1}H NMR (600 MHz, $CDCl_3$) of compound **6b**

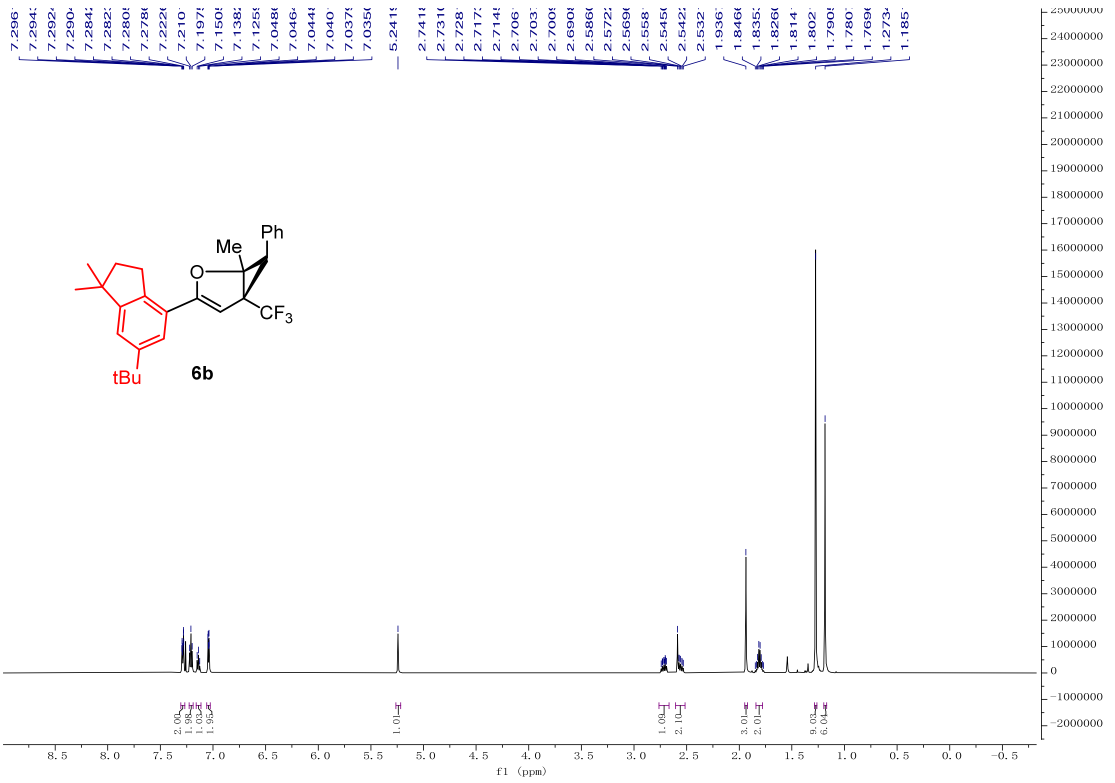

^{13}C NMR (151 MHz, $CDCl_3$) of compound **6b**

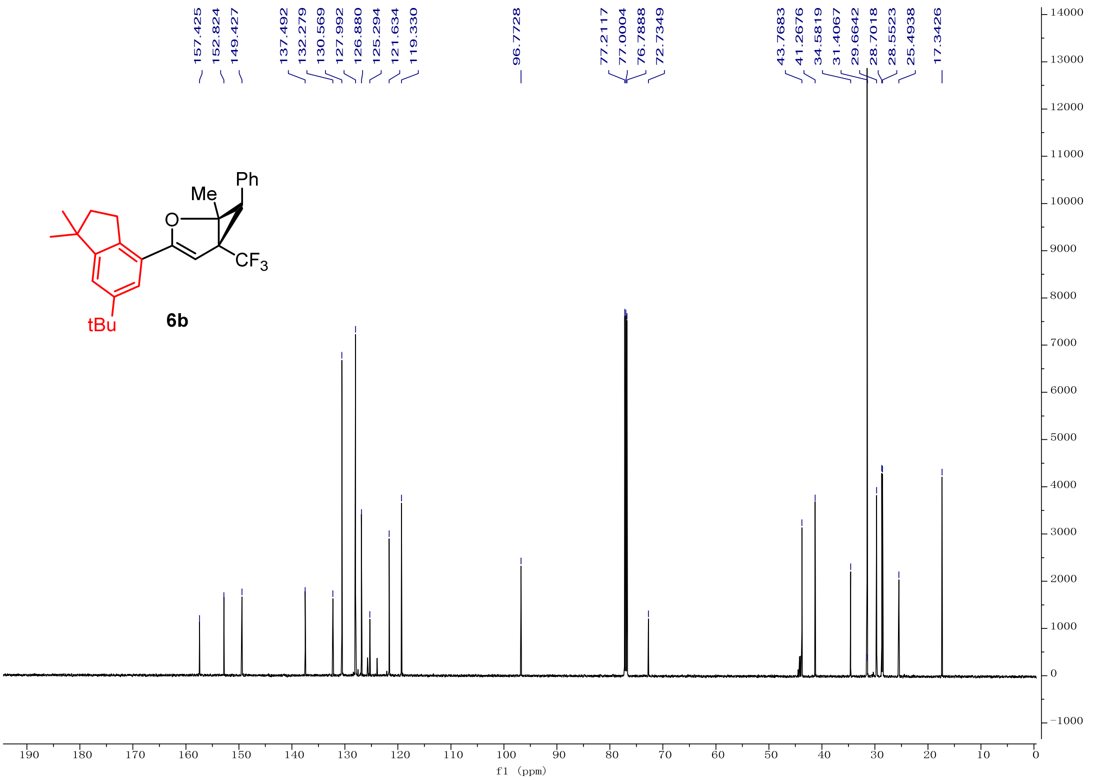

^{19}F NMR (565 MHz, $CDCl_3$) of compound **6b**

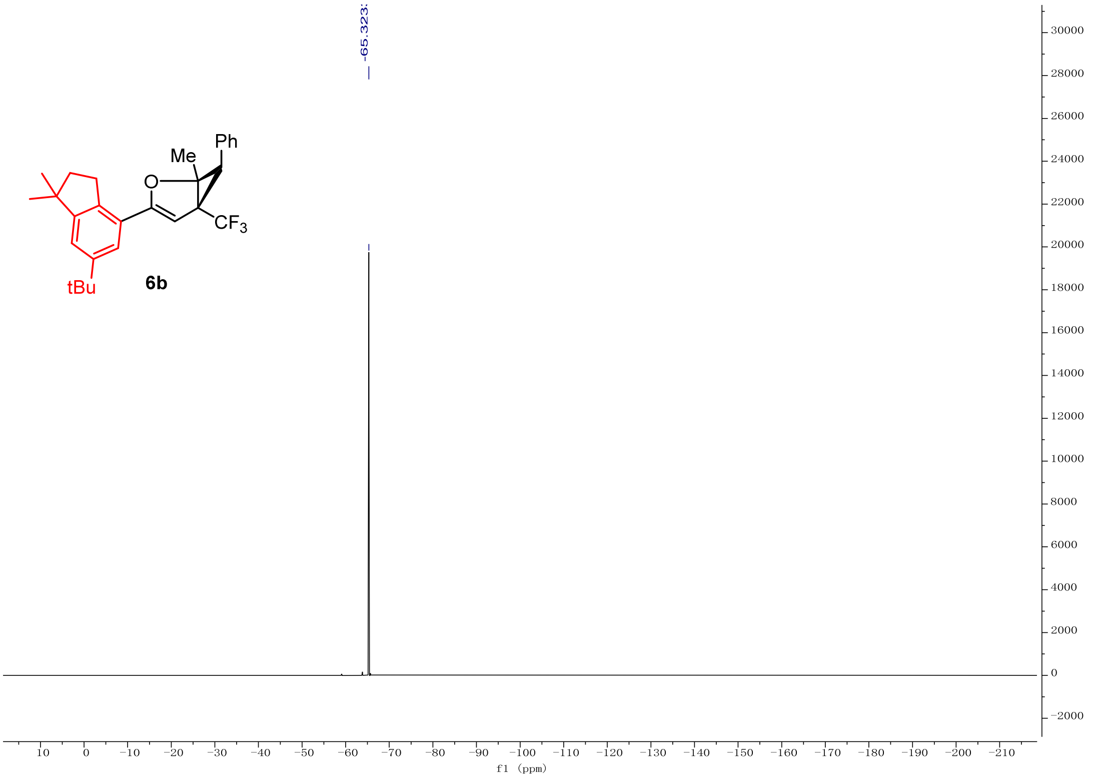

^{1}H NMR (600 MHz, $CDCl_3$) of compound **6c**

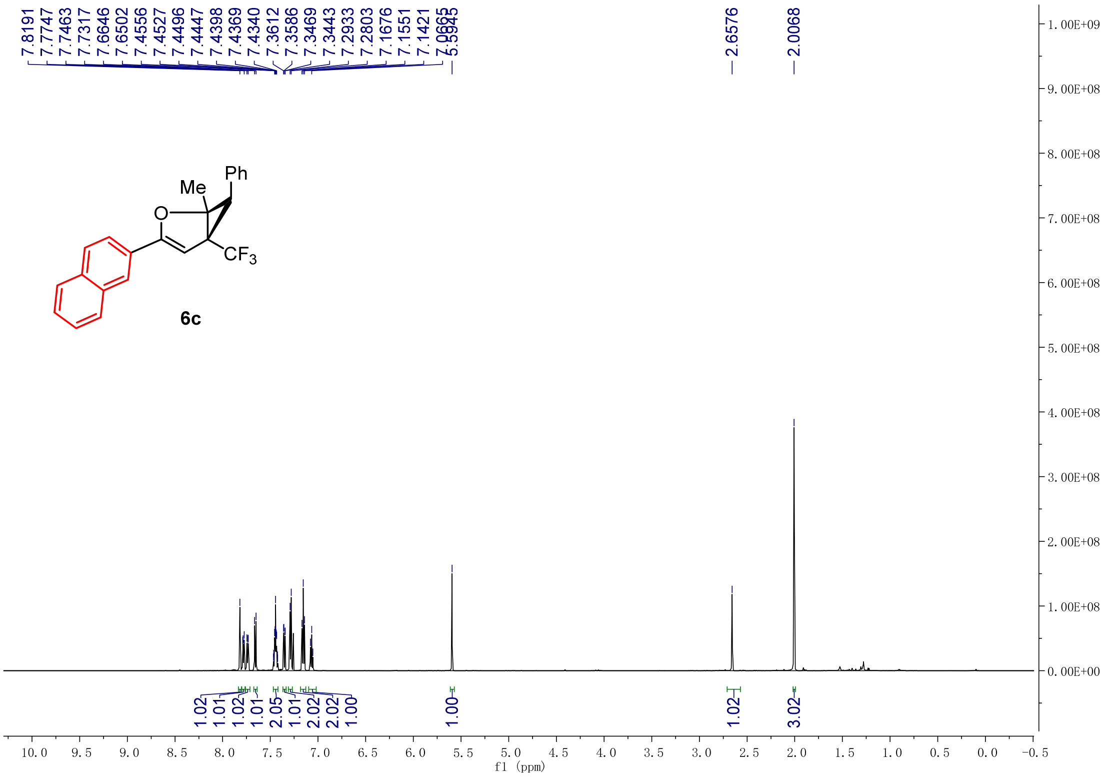

^{13}C NMR (151 MHz, $CDCl_3$) of compound **6c**

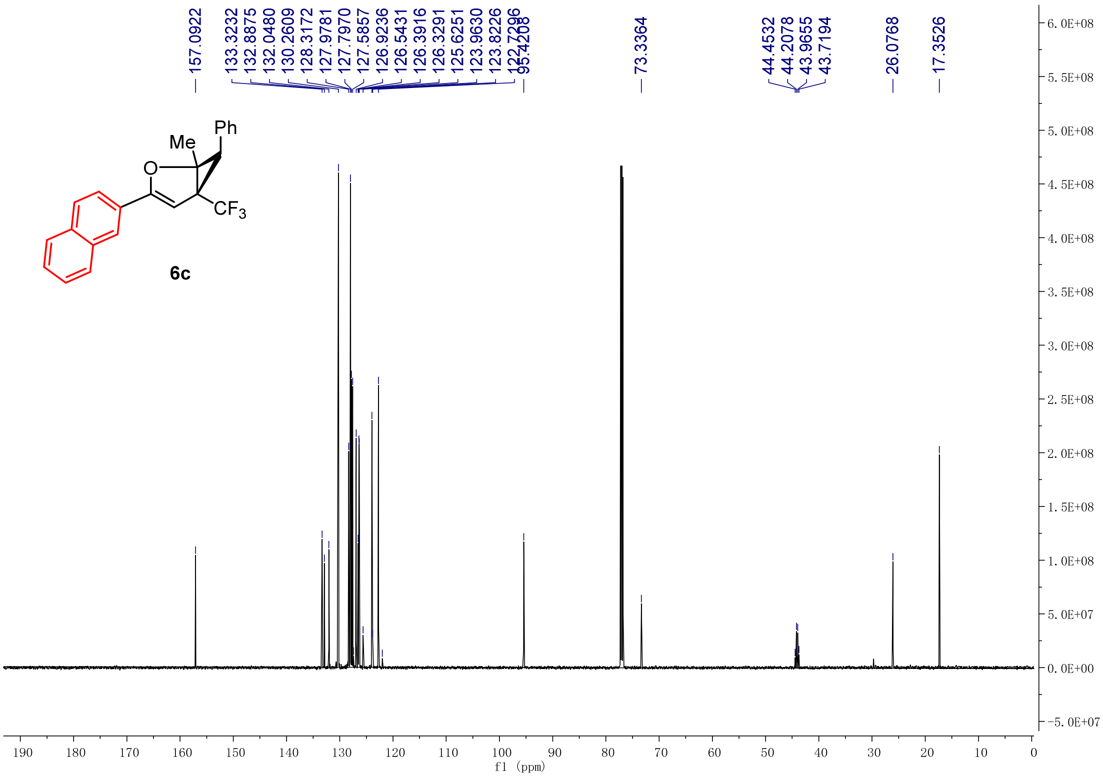

^{19}F NMR (565 MHz, $CDCl_3$) of compound **6c**

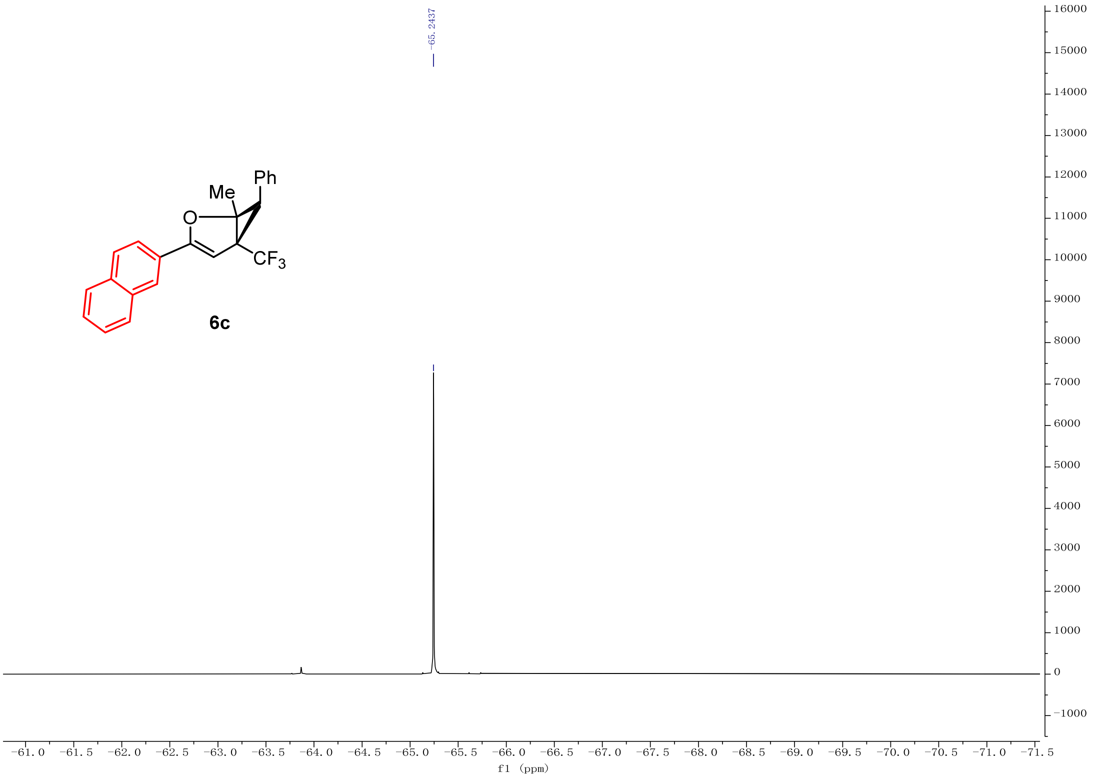

^{1}H NMR (600 MHz, $CDCl_3$) of compound **6d**

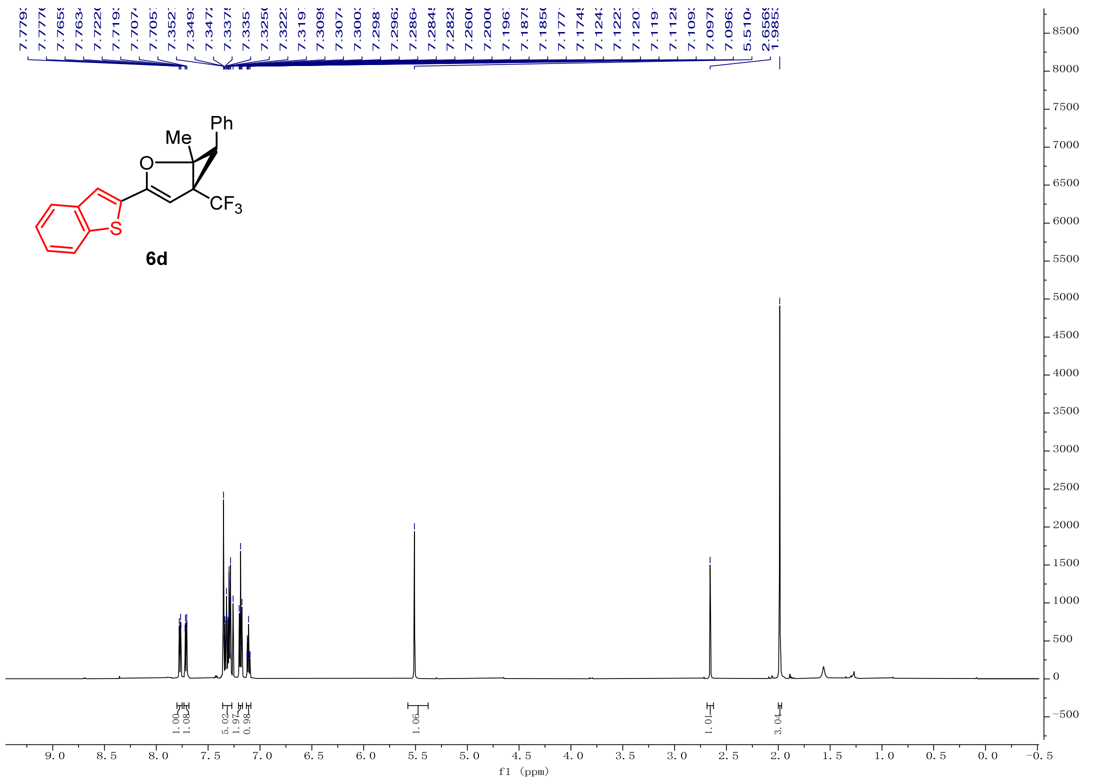

^{13}C NMR (151 MHz, $CDCl_3$) of compound **6d**

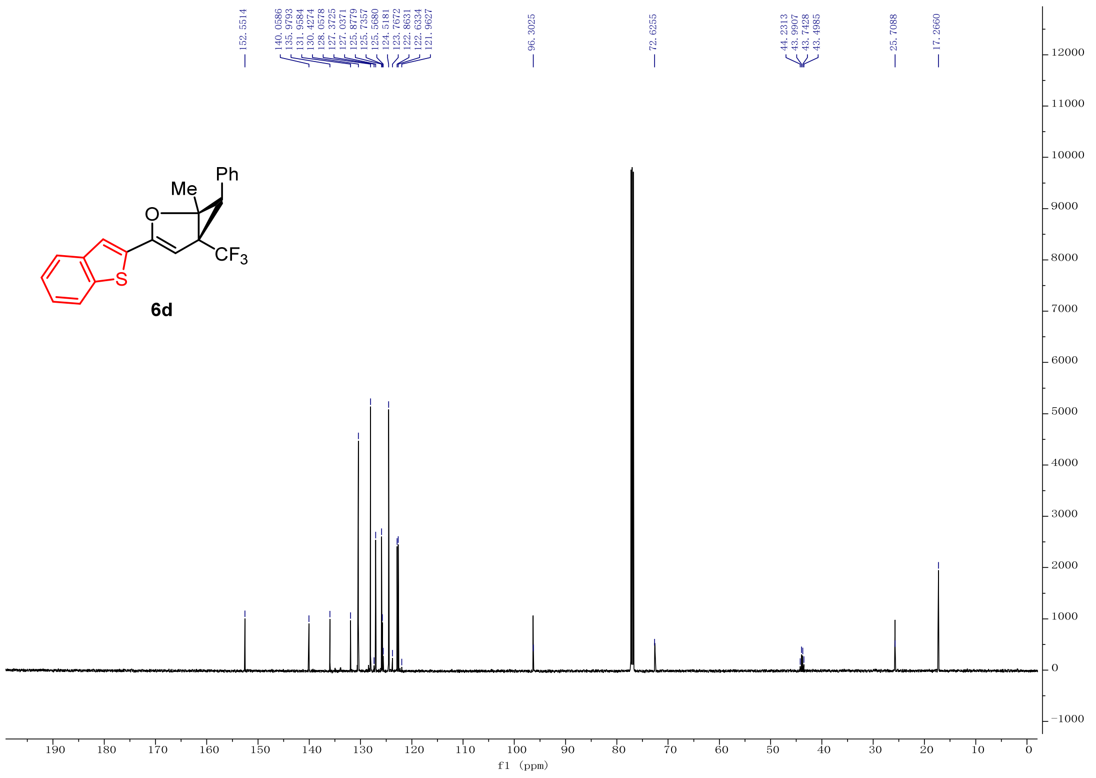

^{19}F NMR (565 MHz, $CDCl_3$) of compound **6d**

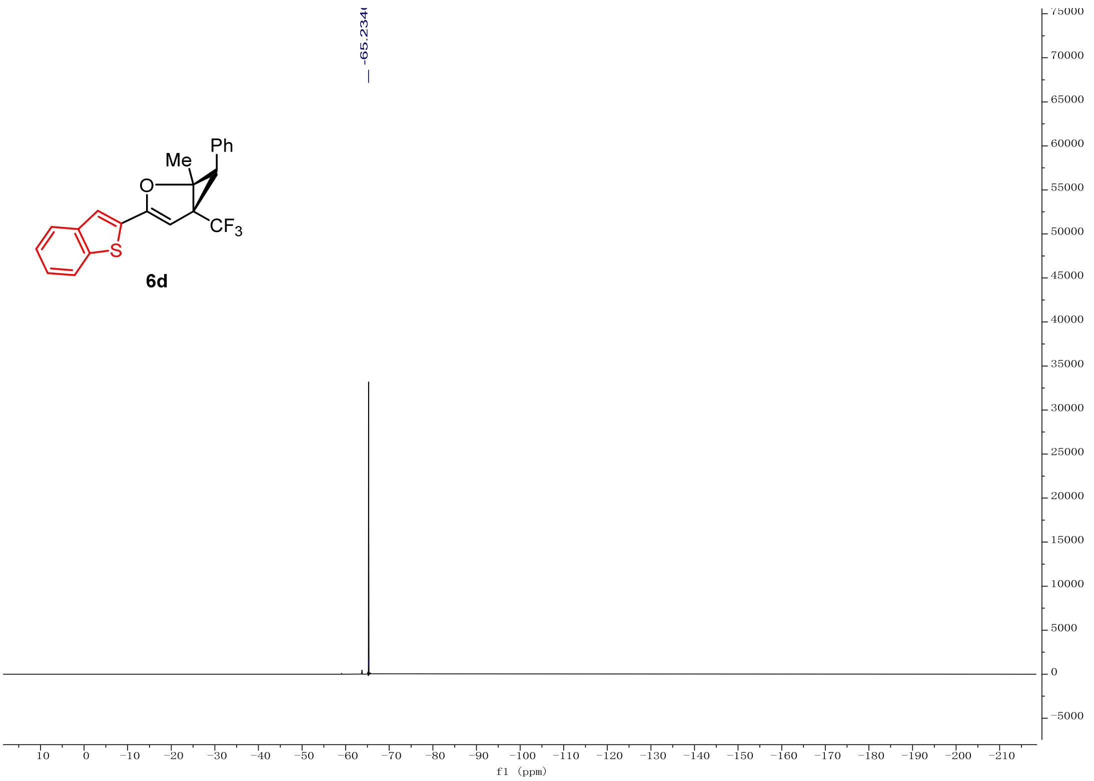

^{1}H NMR (600 MHz, $CDCl_3$) of compound **6e**

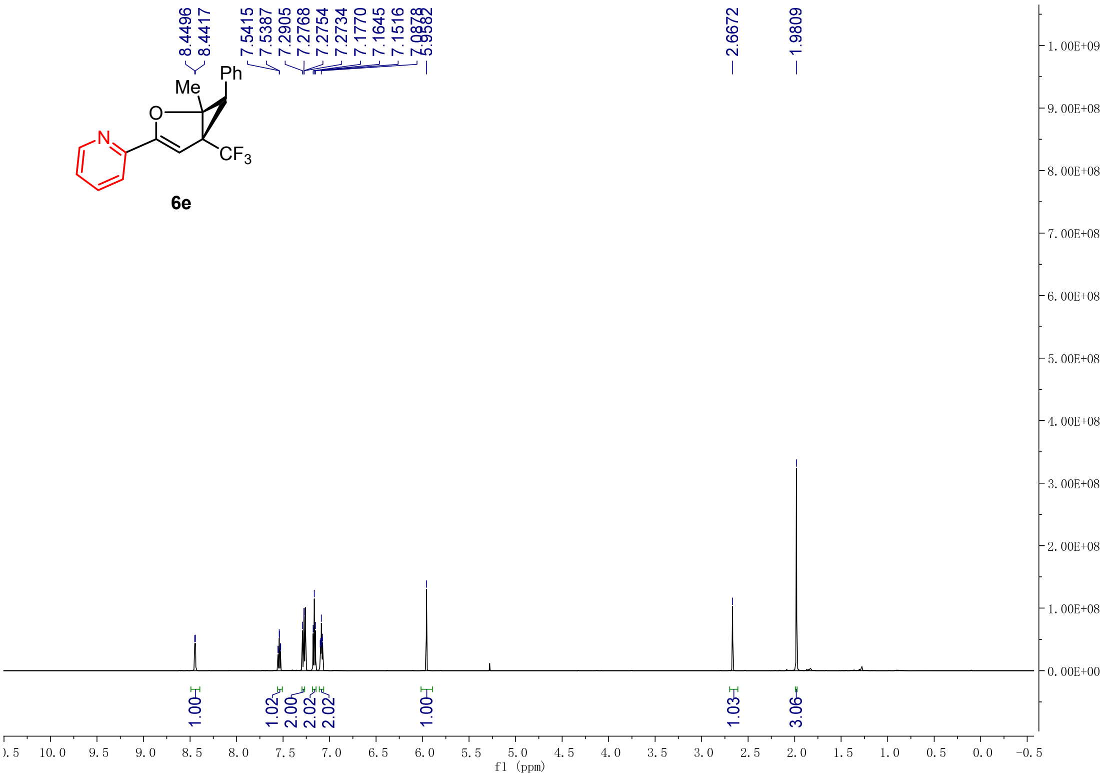

^{13}C NMR (151 MHz, $CDCl_3$) of compound **6e**

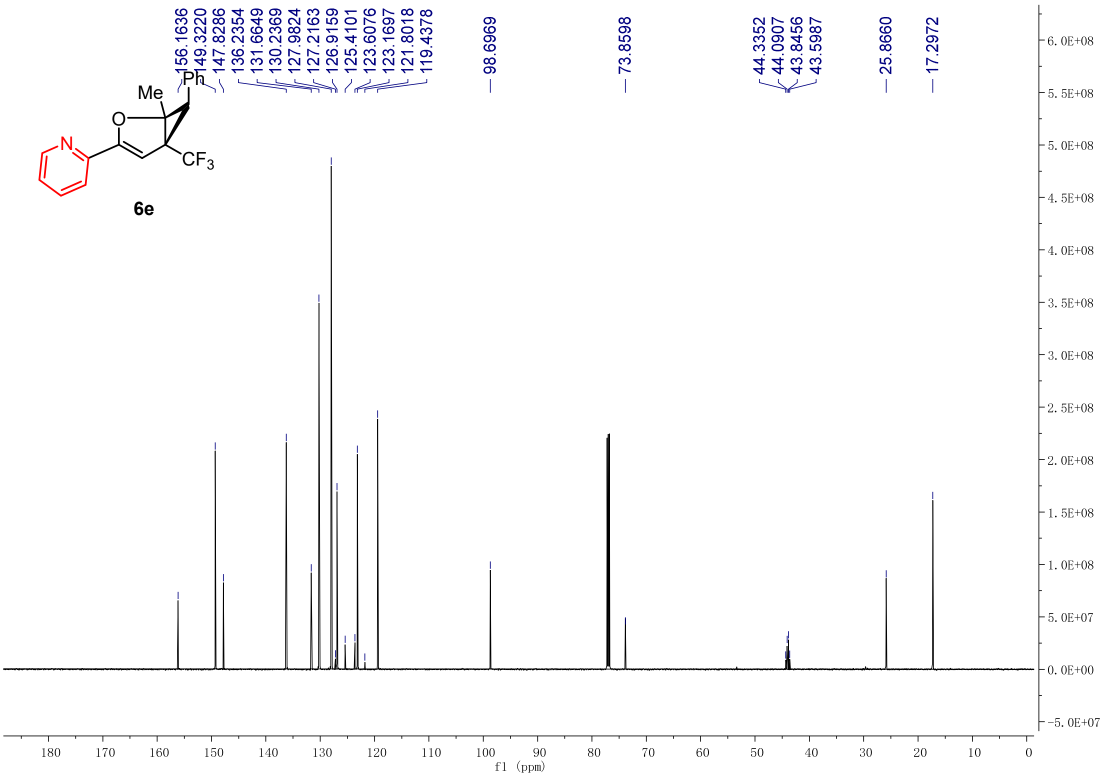

^{19}F NMR (565 MHz, $CDCl_3$) of compound **6e**

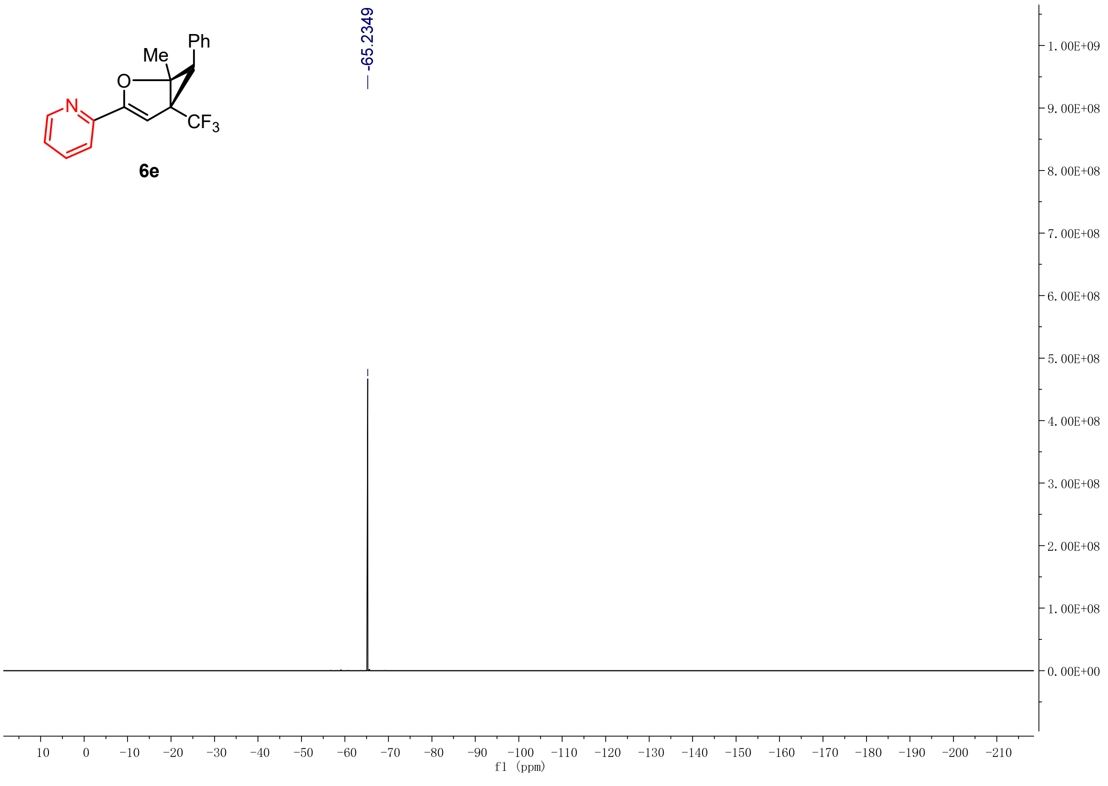

^{1}H NMR (600 MHz, $CDCl_3$) of compound **6f**

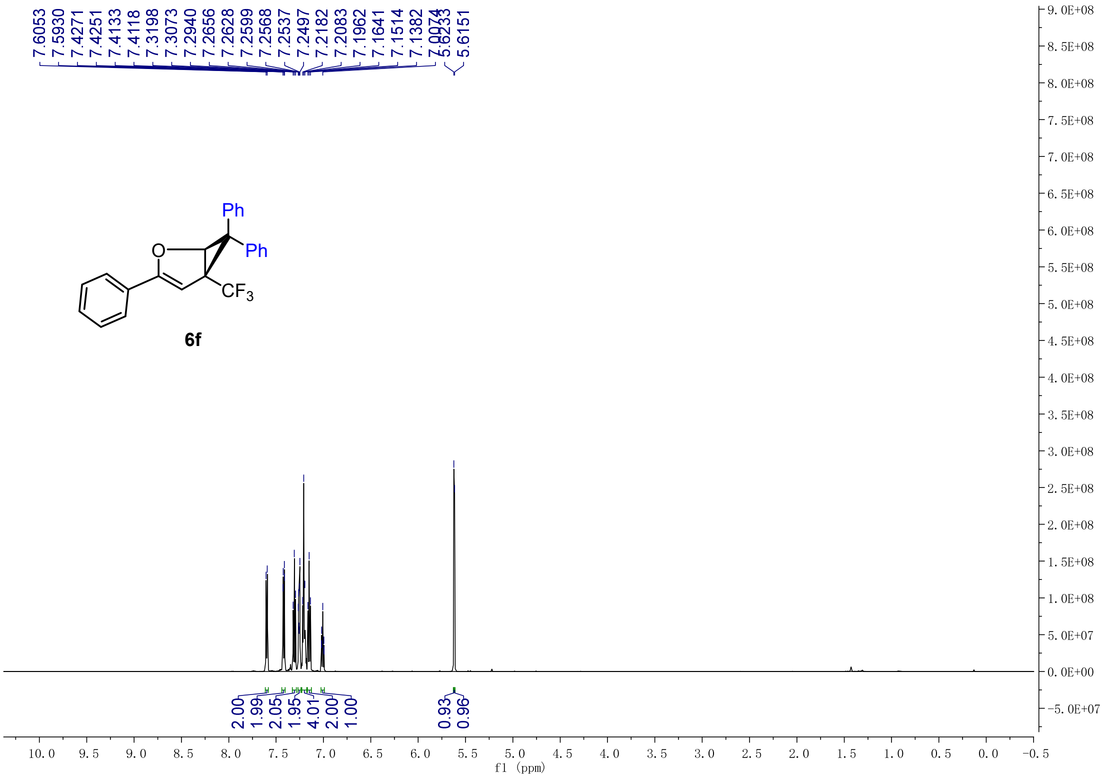

^{13}C NMR (151 MHz, $CDCl_3$) of compound **6f**

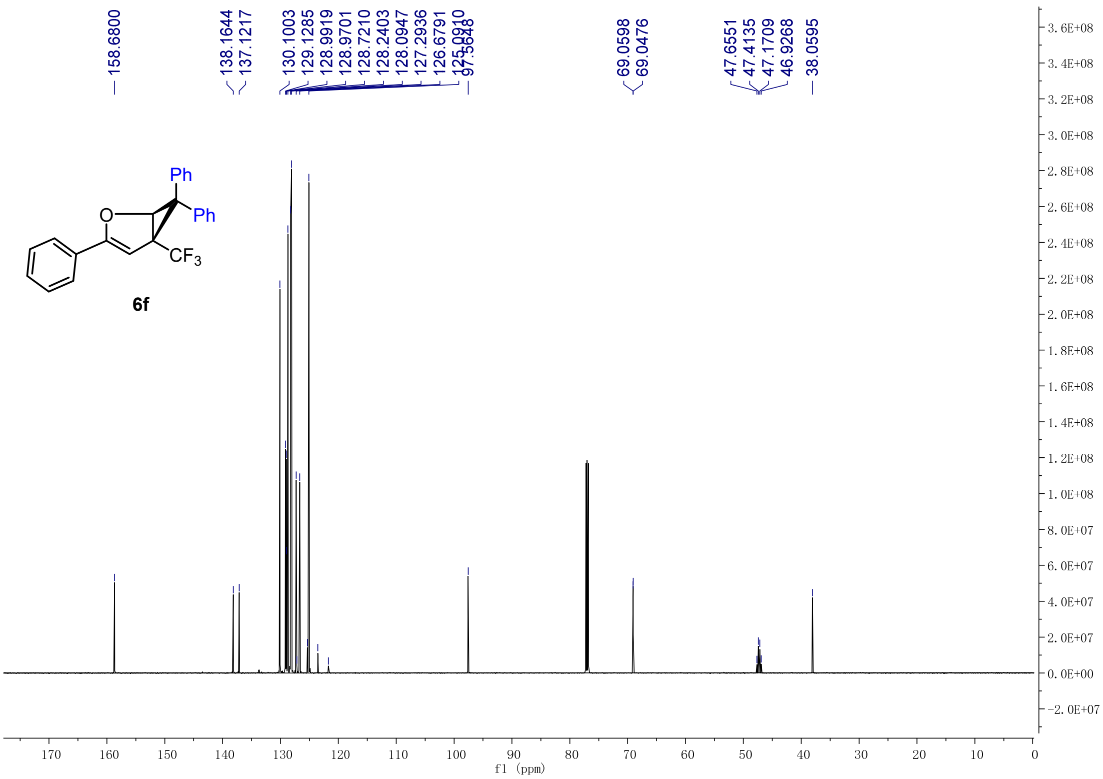

^{19}F NMR (565 MHz, $CDCl_3$) of compound **6f**

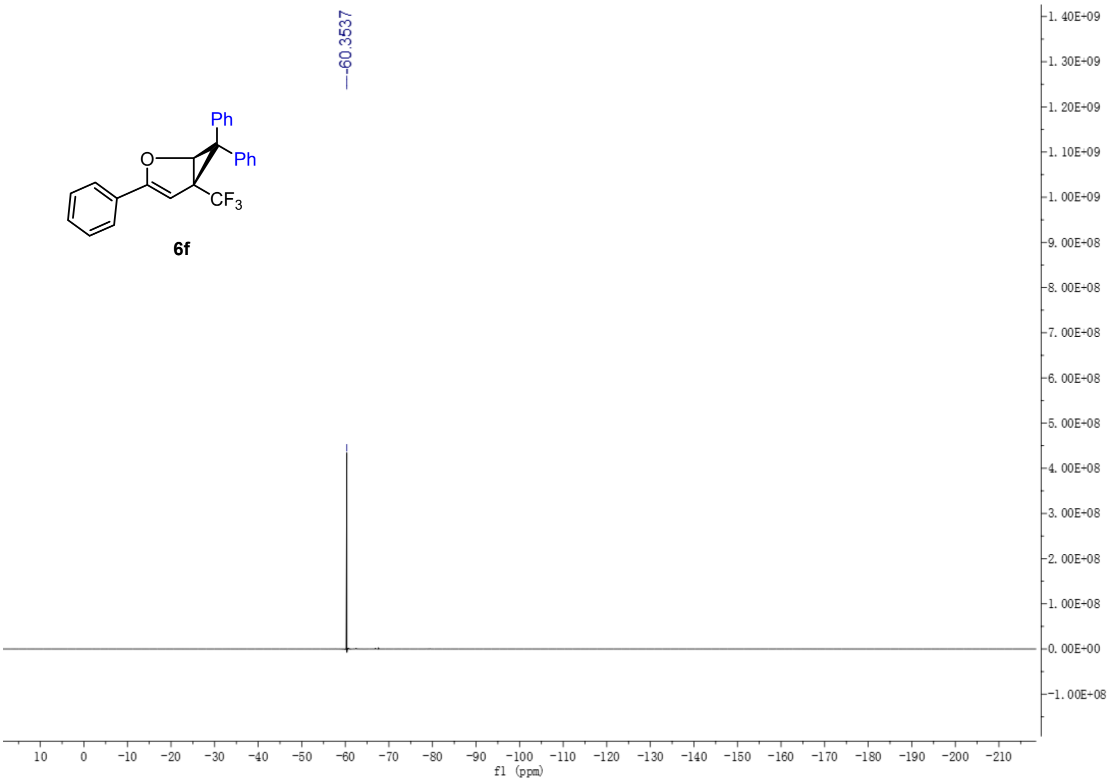

12. References

1. M. J. Frisch, G. W. Trucks, H. B. Schlegel, G. E. Scuseria, M. A. Robb, J. R. Cheeseman, G. Scalmani, V. Barone, G. A. Petersson, H. Nakatsuji, X. Li, M. Caricato, A. V. Marenich, J. Bloino, B. G. Janesko, R. Gomperts, B. Mennucci, H. P. Hratchian, J. V. Ortiz, A. F. Izmaylov, J. L. Sonnenberg, Williams, F. Ding, F. Lipparini, F. Egidi, J. Goings, B. Peng, A. Petrone, T. Henderson, D. Ranasinghe, V. G. Zakrzewski, J. Gao, N. Rega, G. Zheng, W. Liang, M. Hada, M. Ehara, K. Toyota, R. Fukuda, J. Hasegawa, M. Ishida, T. Nakajima, Y. Honda, O. Kitao, H. Nakai, T. Vreven, K. Throssell, J. A. Montgomery Jr., J. E. Peralta, F. Ogliaro, M. J. Bearpark, J. J. Heyd, E. N. Brothers, K. N. Kudin, V. N. Staroverov, T. A. Keith, R. Kobayashi, J. Normand, K. Raghavachari, A. P. Rendell, J. C. Burant, S. S. Iyengar, J. Tomasi, M. Cossi, J. M. Millam, M. Klene, C. Adamo, R. Cammi, J. W. Ochterski, R. L. Martin, K. Morokuma, O. Farkas, J. B. Foresman, D. J. Fox, *Gaussian 16, Revision B.01*; Gaussian, Inc.: Wallingford, CT, 2016.

2. Y. Zhao and D. G. Truhlar, *Theor. Chem. Acc.*, 2008, **120**, 215–241.

3. A. D. Becke, *Phys. Rev. A*, 1988, **38**, 3098-3100.

4. A. D. Becke, *J. Chem. Phys.,* 1993, **98**, 5648-5652.

5. C. Lee, W. Yang and R. G. Parr, *Phys. Rev. B,* 1988, **37**, 785-789.

6. A. V. Marenich, C. J. Cramer and D. G. Truhlar, *J. Phys. Chem. B*, 2009, **113**, 4538–4543.

7. K. Fukui, *Acc. Chem. Res.*, 1981, **14**, 363–368.

8. C. Gonzalez and B. Schlegel, *J. Chem. Phys.*, 1989, **90**, 2154–2161.

9. C. Y. Legault, CYLview, 1.0b; Université de Sherbrooke: Canada, 2009. Available at: http://www.cylview.org (accessed September, 2014).

10. E. Li, J. Chen and Y. Huang, *Angew. Chem. Int. Ed.*, 2022, **61**, e202202040.

11. M. Trost and L. Debien, *J. Am. Chem. Soc.*, 2015, **137**, 11606–11609.

12. Z. C. Litman, Y. Wang, H. Zhao and J. F. Hartwig, *Nature*, 2018, **560**, 355–359.

13. B. B. C. Peters, J. Jongcharoenkamol, S. Krajangsri and P. G. Andersson, *Org. Lett.,* 2020, **23**, 242–246.

14. M. R. Kalbagh, D. Karuturi, S. Kulala, M. Montgomery, P. Kamath and M. Lal, *Synth. Commun.*, 2020, **50**, 3062–3071.

15. K. Matoba, H. Kawai, T. Furukawa, A. Kusuda, E. Tokunaga, S. Nakamura, M. Shiro and N. Shibata, *Angew. Chem. Int. Ed.*, 2010, **49**, 5762-5766.

16. X. Wu, F. Xie, I. D. Gridnev and W. Zhang, *Org. Lett.,* 2018, **20**, 1638–1642.
